# A photochemical strategy for aromatic nitrogen *ortho*-isomerization

**DOI:** 10.1039/d5sc05329c

**Published:** 2025-10-02

**Authors:** Giovanni Lenardon, Xheila Yzeiri, Gael Le Berre, Dilara Berna Yildiz, Daniele Leonori, Alessandro Ruffoni

**Affiliations:** a Institute of Organic Chemistry, RWTH Aachen University Landoltweg 1 Aachen 52074 Germany daniele.leonori@rwth-aachen.de; b Department of Chemistry, Faculty of Science, Gazi University Teknikokullar Ankara 06500 Turkey; c Otto Diels – Institute of Organic Chemistry, Christian Albrecht Universitat zu Kiel Otto-Hahn-Platz 4 24188 Kiel Germany aruffoni@oc.uni-kiel.de

## Abstract

Anilines are essential functional groups in bioactive molecules. Their aromatic substitution pattern governs key physicochemical properties and thus biological activity. Accessing anilines with identical substituents but at alternative aromatic positions is highly desirable, but remains synthetically challenging. Herein, we report a synthetic strategy enabling the *ortho*-isomerization of aromatic nitrogen substituents. This approach leverages the visible light-mediated decomposition of aryl azides in the presence of a tailored thiophenol reagent to generate *ortho*-aminothiophenols. This transformation proceeds *via* nitrene generation and insertion, relocating the nitrogen group to its *ortho* position while installing the sulfur moiety at the *ipso* site. Subsequent oxidative cyclization yields a cyclic sulfonium intermediate, which can be cleaved or exploited as linchpins for divergent functionalization.

## Abstract 2

Anilines with different substitution patterns exhibit distinct physicochemical and biological properties, yet their synthesis remains difficult. We present a visible-light method that enables *ortho*-isomerization of nitrogen substituents.

## Graphical abstract

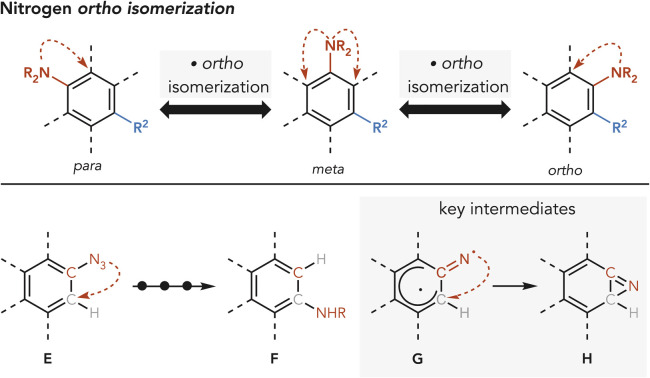

## Introduction

**Scheme 1:**
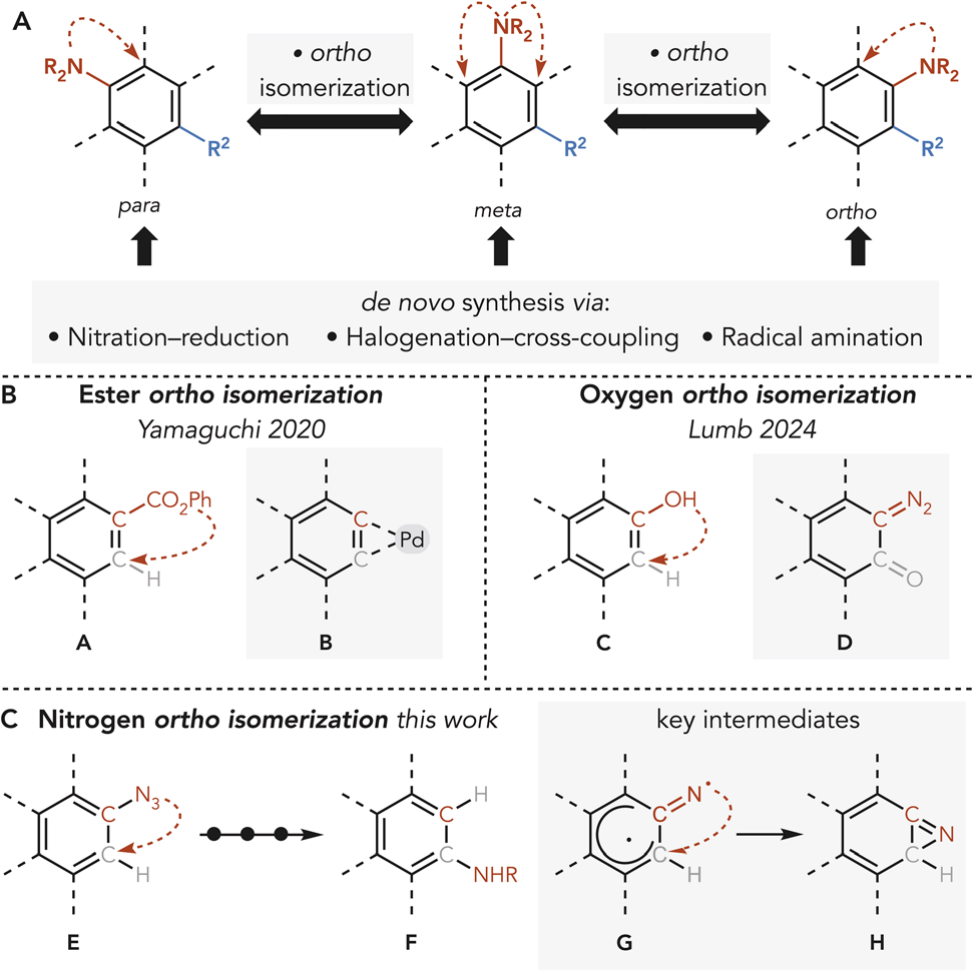
(A) Group translocation-*ortho* isomerization logic. (B) Group translocation-*ortho* isomerization precedent. (C) This work.

Anilines are ubiquitous structural motifs in bioactive molecules.^1,2^ The physicochemical properties and function of anilines are dictated by the nature and the positioning of their substituents.^3–5^ Changes in substitution patterns profoundly impact interactions with biological targets by modulating H-bonding, π-stacking, and other non-covalent interactions.^6,7^ Thus, positional isomers of anilines often display distinct biological profiles, underscoring the importance of accessing such variants during structure–activity relationship campaigns. Despite this relevance, accessing all possible isomeric aromatic compounds can still be challenging and, in most cases, each derivative requires a specifically designed and optimized *de novo* synthesis. This is due to the nature of traditional aromatic amination that follows a linear sequence of synthetic steps where functionality precursors (*e.g.* nitro group for reduction)^8–10^ or reactivity handles (*e.g.* halides for Buchwald-Hartwig cross-coupling) are installed *via* electrophilic aromatic substitution.^11–14^ Although more modern radical-based methods offer a more direct approach, they generally afford only a single isomer (*para*) and do not overcome the limitations in regioisomeric diversification (*ortho* – *meta*).^15–20^ A highly desirable yet underexplored strategy involves the direct translocation of functional groups from one aromatic position to another. This would bypass traditional synthetic limitations and facilitate access to high-value isomers. Unfortunately, methods that achieve such rearrangements are rare.^21–23^ The classical “halogen dance” strategy is limited to heteroaromatics^24,25^ and fails with benzenoids.^26–29^ Very recently, work by Yamaguchi and Lumb has explored new approaches to achieving aromatic *ortho*-isomerization. Yamaguchi developed a Pd-catalyzed process for the reconfiguration of ester groups A leveraging the thermodynamic equilibrium of η^2^-aryne-palladium complexes B during carbonylation and elimination steps (Scheme 1B).^30,31^ Lumb enabled the *para* to *meta* migration of OH groups in phenols C employing a designed sulfonyl hydrazide reagent to selectively generate diazonium intermediates D (Scheme 1B).^32^ These methods are restricted to single positional shifts without enabling further isomerization. Here, we report a synthetic protocol for nitrogen *ortho*-isomerization on benzene rings (Scheme 1C). Our approach uses aryl azides and a tailored thiophenol reagent under visible-light photolysis to generate singlet nitrenes, which undergo regioselective insertion to yield *ortho*-aminothiophenols. These intermediates are converted to sulfonium salts, which serve as both removable handles and diversification platforms. This protocol enables the isomerization of the nitrogen atom from the *para* to the *meta* position, and from the *meta* to the *ortho* or *para* positions, potentially allowing controlled access to all possible isomers. The strategy is compatible with polysubstituted derivatives and has been applied to the isomerization of bioactive molecules.

## Results and discussion

### Design plan

**Scheme 2:**
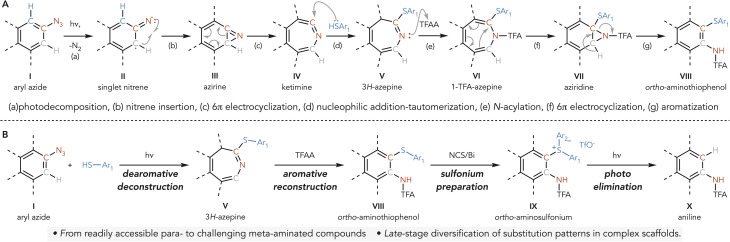
(A) Proposed mechanistic blueprint for the photochemical conversion of aryl azides into *ortho*-aminothiophenols. (B) Synthetic strategy design for nitrogen *ortho* isomerization.

In our earlier studies we demonstrated the synthesis of *ortho*-aminothiophenols,^33^ upon irradiating aryl azides I and sodium thiolates with purple LEDs (*l* = 390 nm) (Scheme 2A).^33–37^ This process generates singlet aryl nitrenes II,^38–43^ which undergo N-insertion to form azirines III.^44^ These intermediates partake in a 6π-electrocyclization event, leading to highly electrophilic ketimines IV. These intermediates can be trapped *in situ* with thiol nucleophiles and the resulting 1*H*-azepines^45–47^ readily isomerize to the thermodynamically stable 3*H*-azepines V.^48–53^ When treated with electrophiles like TFAA, V were converted into VII*via* the generation of antiaromatic VI, followed by 6π-electrocyclization^33,37,54,55^ and aromatization. A key mechanistic aspect of this strategy is that the nitrogen undergoes *ortho*-isomerization, with the thiol being selectively trapped at the original *ipso* position of the starting aryl azide I. Although this methodology effectively relocates the nitrogen atom, it fails to deliver rearranged anilines due to sulfur incorporation in the final product. We envisioned that conversion of these aminothiophenols into sulfonium species followed by visible-light-mediated cleavage could provide a route to the rearranged anilines (Scheme 2B).

### Reaction development

**Scheme 3:**
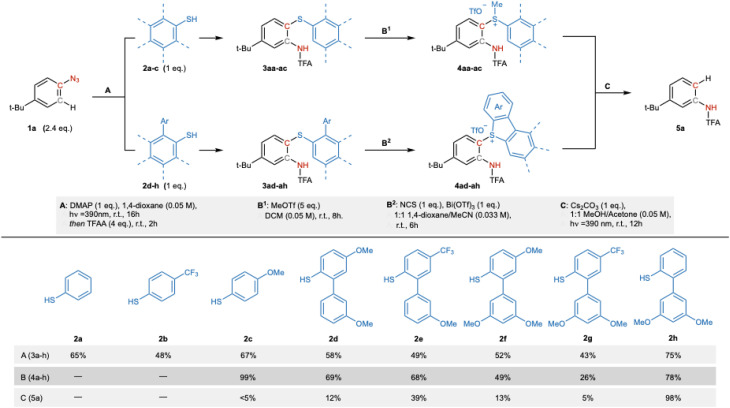
Optimized reaction conditions & thiol reagent design.

The success of this concept hinged on identifying a thiophenol capable of both trapping the ketimine intermediate and supporting subsequent sulfonium formation for the final cleavage step. Building on our previous work, we began by evaluating thiols 2a and 2b, which successfully enabled the formation of *ortho*-aminothiophenols.^33^ However, these intermediates failed to generate the corresponding sulfonium salts, likely due to the insufficient nucleophilicity of the sulfur moiety. In contrast, the more electron-rich thiol 2c afforded the desired sulfonium in high yield, but attempts at photoinduced cleavage were unsuccessful. These results clearly underscored the need for a single thiophenol reagent that could efficiently support all three steps of the sequence. To address this, we explored *ortho*-aryl-substituted thiophenols, which were expected to form cyclic triaryl sulfoniums. These species were of particular interest due to their known propensity for undergoing photofragmentation with release of dibenzothiophene.^56–59^ Accordingly, we evaluated a series of biaryl thiophenols (2d–2h, Scheme 3). However, under standard conditions, these derivatives failed to participate in the nitrene-based insertion reaction. We found that their conversion to the corresponding sodium salts, required to activate the thiol for ketimine trapping, resulted in significant decomposition, presumably due to their high electron density and susceptibility to oxidative degradation. Since these thiophenols showed no reactivity in the absence of a base, we re-optimized the nitrene-insertion step. Rather than using a strong base, which can readily generate easily oxidized thiolates, we investigated the use of a milder base that could potentially also act as a nucleophilic activator. This base may trap the ketimine intermediate, thereby enabling its subsequent displacement by the sulfide. This approach proved effective, and DMAP was identified as the optimal additive to facilitate capture of intermediate IV by thiophenols 2d–2h.

Among these, thiophenol 2h gave the highest conversion, whereas derivatives bearing para electron-donating (OMe, compounds 2d and 2f) or electron-withdrawing (CF_3_, compounds 2e and 2g) substituents displayed reduced reactivity. With this improved protocol, we next examined sulfonium formation: upon treatment with MeOTf in DCM (conditions B^2^) only substrate 3ac was successfully converted to the desired product, which then unfortunately failed to undergo the subsequent step. On the other hand, with NCS as the electrophile and Bi(OTf)_3_ as the Lewis acid (conditions B^1^) we were able to obtain all the corresponding triaryl sulfonium salts, with^60,61^ the derivative derived from thiophenol 2h affording once again the highest yield. The final photocleavage step was evaluated using purple LEDs (*l* = 390 nm) in a MeOH–acetone mixture with Cs_2_CO_3_ as base. Notably, the efficiency of this transformation was highly sensitive to the substitution pattern on the sulfonium ring. While several sulfonium derivatives (2c, 2d, 2f and 2g) gave poor conversions, the compound derived from thiophenol 2h underwent clean photofragmentation to deliver the desired translocated aniline in quantitative yield. Although the origins of this enhanced reactivity remain unclear, thiophenol 2h consistently outperformed all other candidates across each of the three steps and was therefore selected as the optimal reagent for further scope exploration.

### Scope of the nitrogen ring walk

With the optimized reaction conditions in hand, we explored the substrate scope using *para*-substituted aryl azides (1a–1y). These azides, which are readily accessible from the corresponding *para*-substituted anilines as well as other feedstock materials (see SI material for details), serve as valuable precursors to *meta*-functionalized anilines, which are often synthetically challenging to access and, in fact, represent only a small fraction (7%) of commercially available compounds (see SI material for details).

**Scheme 4:**
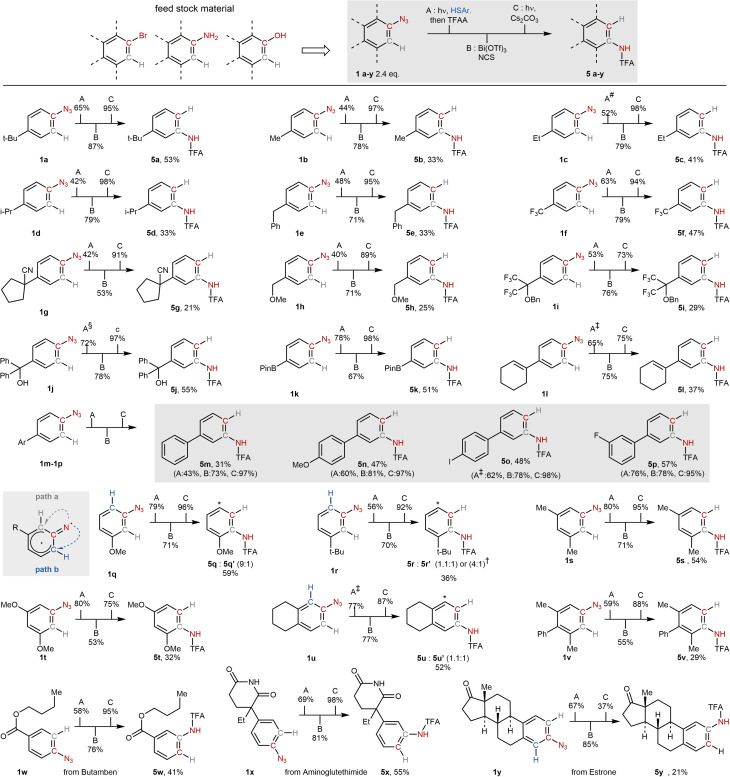
Substrate scope of the nitrogen *ortho* isomerization. Solvent: # hexane, § diethyl ether, ‡ trifluorotoluene, † step A: trifluorotoluene 60 °C. Conditions: A: DMAP (1 eq.), 1,4-dioxane (0.05 M), hν = 390 nm, r.t., 16 h, then TFAA (4 eq.), r.t., 2h. B: NCS (1 eq.), Bi(OTf)3 (1 eq.), 1 : 1 1,4-dioxane/MeCN (0.033 M), r.t., 6 h. C: Cs2CO3 (1 eq.), 1 : 1 MeOH/Acetone (0.05 M), hν = 390 nm, r.t., 12 h. Yield : A = yield calculated based on the amount of thiol used, A and B = NMR yield, C = isolated yield.

**Scheme 5:**
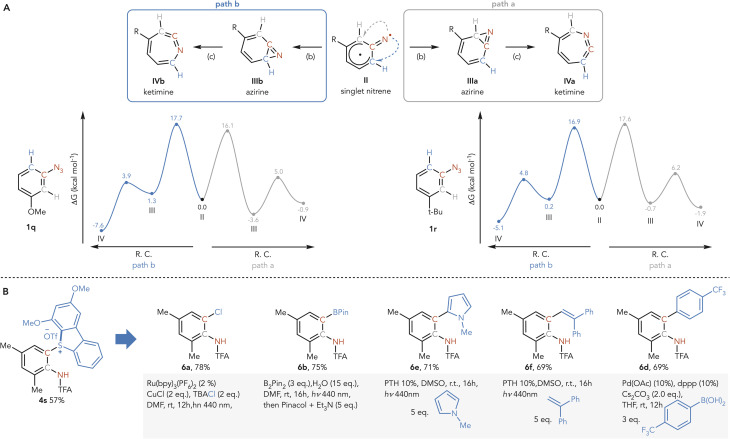
(A) Mechanistic analysis of regioselectivity. The numbers are the relative Gibbs free energies at UM06-2X/def2-QZVPP//def2-TZVP level of theory (kcal mol^−1^). (B) Synthesis of *ortho*-anilines *via* sulfonium diversification.

As shown in Scheme 4A, a series of alkyl substituents were well tolerated, delivering products 5b–5e in overall good yields across the three-step sequence. Importantly, these substituents could incorporate a diverse range of functional groups, including HAT-activated benzyl (1e), α-methoxy (1h), nitrile-substituted quaternary carbons (1g), and trifluoromethyl groups (1f, 1i). These products (5g–5i) were obtained in slightly reduced yields. Free alcohols, as in aryl azide 1j, afford 5j in 55% yield, with no observed self-condensation during the initial step, highlighting the protocol's chemoselectivity for thiol nucleophiles. Notably, the strongly electron-withdrawing *para*-CF_3_ group (1f) was compatible, affording 5f in 47% overall yield. A boronic ester [B(pin)] substituent was also tolerated, furnishing product 5k, which is amenable to downstream cross-coupling functionalization. We next examined substrates bearing alkene (1l) and aromatic (1m–1p) groups. These furnished the corresponding anilines (5l–5p) in moderate to good yields and demonstrated compatibility with para-substituents such as methoxy, iodine, and fluorine. This is particularly noteworthy given that *para*-substituted aromatics are known to suppress nitrene insertion due to electronic deactivation.^62^ With *meta*-substituted azides, mixtures of *ortho*- and *para*-products were observed, consistent with the possibility of nitrene cyclization at either *ortho* position (Scheme 3).^63–66^ Nonetheless, the OMe-containing azide 1q showed high regioselectivity for the ortho-isomer 5q. In contrast, the *tert*-butyl derivative 1r gave a near 1 : 1 mixture of *ortho* and *meta* products. Remarkably, shifting the solvent to trifluorotoluene and conducting the reaction at 60 °C significantly enhanced regioselectivity, yielding the *ortho*-isomer 5r in a 4 : 1 ratio. (See the SI material for details of the optimization and comprehensive studies on other substrates) This observation highlights the decisive influence of solvent polarity and temperature on the reaction coordinate, allowing for fine-tuning of positional selectivity (Scheme 5A).

We further extended the scope to polysubstituted azides. Symmetrical di-*meta*-substituted derivatives 1s and 1t yielded *ortho*–*para* anilines 5s and 5t in 54% and 32% yields, respectively, showcasing the method's tolerance toward electronically rich systems. The bicyclic 6-azido-tetrahydronaphthalene 1u provided both regioisomers in comparable yields (36% and 52%). Trisubstituted azide 1v enabled the synthesis of the tetrasubstituted aniline 5v in 29% yield, an otherwise synthetically demanding structure that lies beyond the reach of conventional S_E_Ar or cross-coupling strategies.^5^ To further demonstrate the synthetic utility of our strategy, we applied it to azide derivatives of bioactive compounds. The local anesthetic butamben was converted into *meta*-butamben 5w in 27% yield. Similarly, the anticancer agent aminoglutethimide underwent clean *para*-to-*meta* isomerization to afford 5x in 55% yield. Estrone-derived azides yielded a 1 : 1 mixture of isomerized aminothiophenol intermediates. However, despite efficient sulfonium formation, photochemical cleavage conditions led exclusively to product 5y in 21% yield, with the other regioisomer undergoing complete decomposition.^67^ It is worth noting that TFA protection is generally removed quantitatively after treatment with aqueous KOH wash, delivering the free anilines. This results in an overall isomerization from *para*-aniline (azide precursors) to *meta*-anilines.

### Mechanistic analysis of regioselectivity

To gain deeper insight into the origins of regioselectivity in *meta*-substituted aryl azides such as 1q and 1r, we carried out DFT calculations on the two pathways (path a and path b) that proceed from the singlet nitrene (II) to the ketimine (IV) *via* the azirine (III), as the regioselectivity of the reaction is determined by the nitrene insertion (Scheme 5A). For 1q, the nitrene-to-azirine conversion (step b) is clearly favored along path a: the computed barrier is lower (16.1 *vs.* 17.1 kcal mol^−1^), and the resulting azirine intermediate is more stabilized (−3.6 *vs.* 1.3 kcal mol^−1^). This dual kinetic and thermodynamic advantage renders path a the dominant trajectory, providing a straightforward rationale for the regioselectivity observed experimentally. In the case of 1r, however, the situation is more nuanced. The two transition states are separated by less than 1 kcal mol^−1^, and the corresponding azirine intermediates differ only slightly in stability (path a favored by 0.9 kcal mol^−1^). The combination of a slightly lower barrier for the path b azirine and the greater stability of the path a azirine establishes a kinetic-thermodynamic dichotomy: the path b azirine can be classified as the kinetic product, while the path a analogue represents the thermodynamic minimum. This balance provides a natural rationale for the temperature-dependent outcome: under ambient conditions both azirines may form in comparable amounts, whereas the elevated temperature equilibration shifts the distribution toward the thermodynamically preferred path a.

### Synthesis of *ortho*-aniline

Aryl sulfoniums are powerful synthetic handles for diverse functionalization *via* cross-coupling reactions and redox chemistry.^68–74^ The sulfonium group can be introduced onto the aromatic ring either by exploiting existing prefunctionalization^68,73^ or directly *via* C–H functionalization, typically with para-selectivity.^69,72,74^ Our protocol allows the selective installation of sulfonium groups *ortho* to aniline functionalities with complete regiocontrol. We envisaged that this intermediate could act as a strategic platform to enable divergent *ortho*-functionalization pathways, expanding the reactivity beyond nitrogen isomerization. This concept was validated as shown in (Scheme 5B). Photoredox-induced fragmentation in the presence of a chlorinating agent provided *ortho*-chloroaniline 6a in 69% yield.^22^ Similarly, blue light irradiation (*λ* = 440 nm) in the presence of B_2_(pin)_2_ afforded *ortho*-borylated product 6b in 75% yield, enabling further downstream diversification through standard cross-coupling protocols.^69^ Furthermore, phenylphenothiazine (PTH)-mediated reduction of sulfonium 4t generated an aryl radical intermediate that was effectively intercepted by methylpyrrole and styrene to afford C–C coupled products 6c and 6d in 71% and 69% yields, respectively. Finally, the versatility of these sulfonium intermediates was showcased through a Suzuki–Miyaura cross-coupling reaction, delivering *ortho*-arylated aniline 6e in 69% yield.

## Conclusions

We have developed a synthetic strategy for *ortho*-isomerization of aromatic nitrogen. This method enables the transformation of easily accessible *para*-substituted anilines into more challenging *meta*-functionalized derivatives. This protocol tolerates many functionalities often encountered in organic synthesis and has proven efficient for late-stage reconfiguration of bioactive molecules. We hope this might find application as a tool for chemical space exploration without the requirement for *de novo* synthesis. Furthermore, the sulfonium intermediates generated as part of the strategy can serve as a powerful linchpin for diverse transformations, including photoredox catalysis and cross-coupling. This can be used to overcome current limitations in aniline functionalization chemistry and expand the scope of accessible derivatives.

## Author contributions

AR and DL conceptualized, designed, and supervised the project. GL, XY and GLB performed all the synthetic experiments. DBY performed the computational experiments. All authors analysed the results and wrote the manuscript.

## Conflicts of interest

There are no conflicts to declare.

## Supplementary Material

SC-OLF-D5SC05329C-s001

SC-OLF-D5SC05329C-s002

## Data Availability

The data that support the findings of this study are available in the supplementary information (SI) of this article. Supplementary information is available. See DOI: https://doi.org/10.1039/d5sc05329c.
