# Supplementary material for: A photochemical strategy for aromatic nitrogen ortho-isomerization
Source: Chem Sci. 2025 Oct 2;16(45):21416–22. doi: 10.1039/d5sc05329c (PMC12530855; doi:10.1039/d5sc05329c)

Supplementary Information (SI) for Chemical Science.
This journal is © The Royal Society of Chemistry 2025

A Photochemical Strategy for Aromatic Nitrogen *ortho*-Isomerization

Giovanni Lenardon,[a] Xheila Yzeiri,[a] Gael Le Berre,[a] Dilara Berna Yıldız,[a,b] Daniele Leonori,*[a] and Alessandro Ruffoni*[c]

[a]*Institute of Organic Chemistry, RWTH Aachen University, Aachen 52056, Germany*

[b]*Department of Chemistry, Faculty of Science, Gazi University, Teknikokullar, Ankara, 06500 Türkiye*

[c]*Otto Diels – Institute of Organic Chemistry, Christian Albrecht Universitat zu Kiel, Otto-Hahn-Platz 4, 24188 Kiel, Germany*

daniele.leonori@rwth-aachen.de and aruffoni@oc.uni-kiel.de

Table of Contents

1. General experimental details 4
2. Database analysis of substitution patterns in commercially available nitrogen-containing molecules 5
3. Reaction optimization for 1-azido-4-(*tert*-butyl)benzene 6
   3.1. Base screening 6
   3.2. Equivalents of DMAP screening 8
   3.3. Stoichiometry and concentration screening 8
   3.4. Reaction time screening 9
   3.5. Air and water screening 10
   3.6. Sodium thiolate screening 11
   3.7. Solvent screening 11
4. Reaction optimization for 1-azido-3,5-dimethylbenzene 13
   4.1. Solvent screening 13
   4.2. Stoichiometry of thiol screening 14
   4.3. Stoichiometry of DMAP screening 14
5. Study of regioselectivity in the ring expansion of meta-substituted azides 16
6. Ni-Raney desulfurization studies 25
7. Reaction optimization for cyclization to sulphonium salt 26
8. Reaction optimization for photochemical sulphonium salt cleavage 28
9. General procedure 29
   9.1. Step 1 for thiol preparation (TP1) 29
   9.2. Step 2 for thiol preparation (TP2) 29
   9.3. Azide preparation from anilines (AP1) 30
   9.4. Azide preparation from aryl bromides (AP2) 31
   9.5. General Procedure 1 (GP1) 31
   9.6. General Procedure 2 (GP2) 32
   9.7. General Procedure 3 (GP3) 33
10. Starting Material preparation 34
11. Substrate Scope 51

12. Failed Substrates 77
12.1. Unable to undergo ring expansion to azepane 77
12.2. Unable to undergo TFAA-mediated ring contraction from azepane 77
13. Mechanistical analysis 78
14. Pictures of Reaction Set-up 87
15. References 88
16. NMR Spectra 91

1. General experimental details

All required fine chemicals were used directly without purification unless stated otherwise. All air and moisture sensitive reactions were carried out under Ar atmosphere using standard Schlenk manifold techniques. All solvents were bought from Acros as 99.8% purity and degassed by Ar bubbling. ^{1}H, ^{13}C and ^{19}F NMR spectra were recorded on Bruker Avance Neo 600 MHz or Varian VNMRS 600 MHz. ^{1}H, ^{13}C and ^{19}F Nuclear Magnetic Resonance (NMR) spectra were acquired at various field strengths as indicated and were referenced to the residual peak solvent (for ^{1}H and ^{13}C) or by the instrument internally after locking and shimming to the deuterated solvent (for ^{19}F). ^{1}H NMR coupling constants (*J*) are reported in Hertz (Hz) and refer to apparent multiplicities and not true coupling constants. Data is reported as follows: chemical shift (δ), integration, multiplicity (s = singlet, br s = broad singlet, d = doublet, t = triplet, q = quartet, qi = quintet, sx = sextet, sp = septet, m = multiplet, dd = doublet of doublets, etc.). High-resolution mass spectra were obtained using a Thermo Scientific LTQ Orbitrap XL spectrometer or a Finnigan MAT 95. Spectra were obtained using electron impact ionization (EI) or positive electrospray (ESI) techniques. Analytical TLC: aluminum backed plates pre-coated (0.25 mm) with Merck Silica Gel 60 F254. Compounds were visualized by exposure to UV-light or by dipping the plates in vanillin or permanganate ($KMnO_4$) stain solutions followed by heating. Column chromatography was performed using Merck Silica Gel 60 (40–63 µm). All mixed solvent eluents are reported as v/v solutions. All the reactions were conducted in CEM 9 mL glass microwave tubes. The LEDs used are Kessil PR 160 390.

2. Database analysis of substitution patterns in commercially available nitrogen-containing molecules

We analyzed the Reaxys database to map substitution patterns across nitrogen-containing functional groups (amines, amides, nitro compounds, azides, sulfonamides, and hydroxylamines). For each substitution pattern, we quantified the number of commercially available compounds and calculated their relative percentage within the broader class of nitrogen-containing molecules.

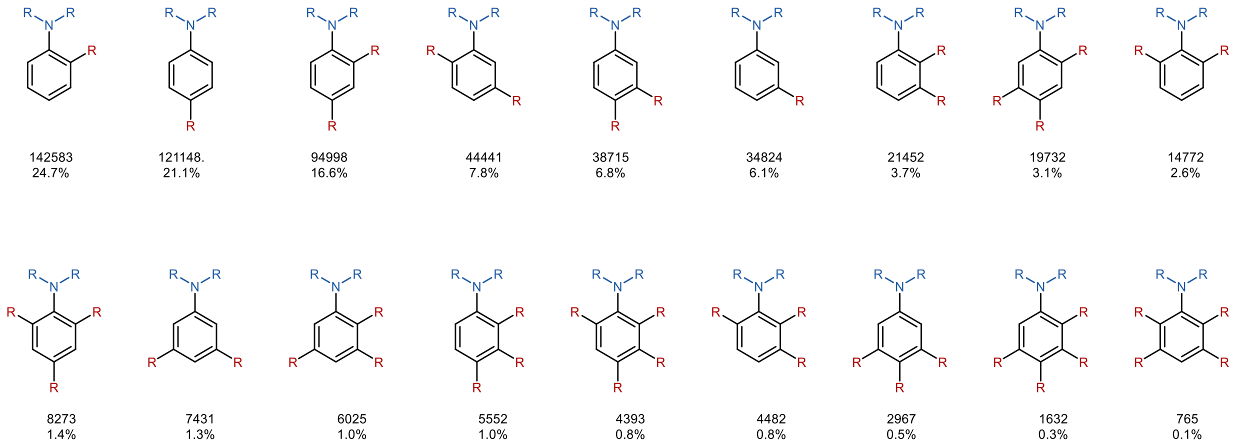

3. Reaction optimization for 1-azido-4-(*tert*-butyl)benzene

3.1. Base screening

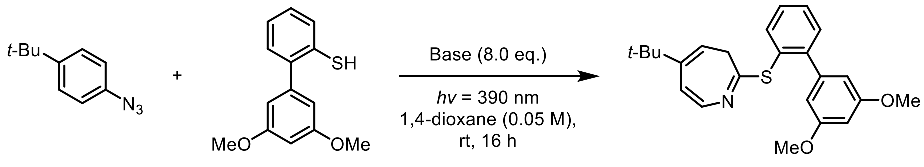

1a **2h** **S1ah**

An oven-dried tube equipped with a stirring bar was charged with aryl azide (0.1 mmol, 1.0 eq.), thiophenol (0.1 mmol, 1 eq.) and base (8.0 eq.). The tube was then capped under air with a Supelco aluminium crimp seal with septum (PTFE/butyl), evacuated and backfilled with argon three times. Anhydrous 1,4-dioxane (2 mL, 0.05 M) was added via syringe. The mixture was then purged with Argon for 5 min. and stirred (600 rpm) under irradiation with purple LEDs (Kessil PR160L-390 nm) for 16 h at room temperature (fan ventilation). Then ethylene carbonate (250 µL, 0.1 M in $CDCl_3$) was added and the solution stirred for 1 min., then 0.3 mL of the solution were placed in an NMR tube, diluted with $CDCl_3$ (0.4 mL) and analyzed by ^{1}H NMR spectroscopy to determine the NMR yield. The crude can be purified *via* Flash column cromatography (Silica gel, Pentane/Et_2O 80:20) to afford a colorless oil.

Table 1

Entry	Base	Yield S1ah (%)
1	NaOEt	-
2	NaOH	-
3	K_3PO_4	-
4	DABCO	-
5	K_2CO_3	-
6	NaOAc	-
7	KF	-
8	DMAP	35%
9	LiOtBu	-
10	Na_2HPO_4	-
11	$AgCO_2CF_3$	-
12	DBU	-
13	NEt_3	-
14	BTMG	-
15	BTTP	-
16	1-methylimidazole	28
17	DIPEA	-
18	Pyridine	-
19	4-(piperidin-1-yl)pyridine	31
20	4-(pyrrolidin-1-yl)pyridine	20
21	4-methylmorpholine	12
22	1-methylpyrrolidine	-
23	1-methyl-1H-benzo[d]imidazole	-
24	1-methyl-1H-pyrrolo[2,3]pyridine	-
25	HOBt	-

3.2. Equivalents of DMAP screening

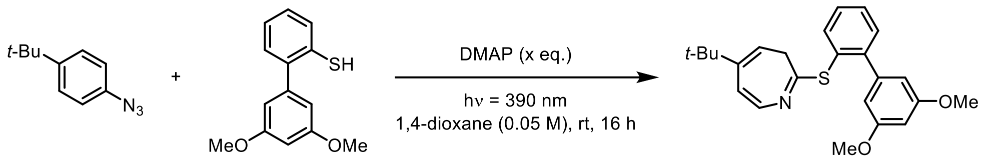

An oven-dried tube equipped with a stirring bar was charged with aryl azide (0.1 mmol, 1.0 eq.), thiophenol (0.1 mmol, 1.0 eq.) and DMAP (x eq.). The tube was then capped under air with a Supelco aluminium crimp seal with septum (PTFE/butyl), evacuated and backfilled with argon three times. Anhydrous 1,4-dioxane (2 mL, 0.05 M) was added via syringe. The mixture was then purged with Argon for 5 min. and stirred (600 rpm) under irradiation with purple LEDs (Kessil PR160L-390 nm) for 16 h at room temperature (fan ventilation). Then ethylene carbonate (250 µL, 0.1 M in $CDCl_3$) was added and the solution stirred for 1 min., then 0.3 mL of the solution were placed in an NMR tube, diluted with $CDCl_3$ (0.4 mL) and analyzed by ^{1}H NMR spectroscopy to determine the NMR yield.

Table 2

Entry	DMAP eq.	Yield S1ah (%)
1	4	35
2	2	35
3	1	37
4	0.2	22

3.3. Stoichiometry and concentration screening

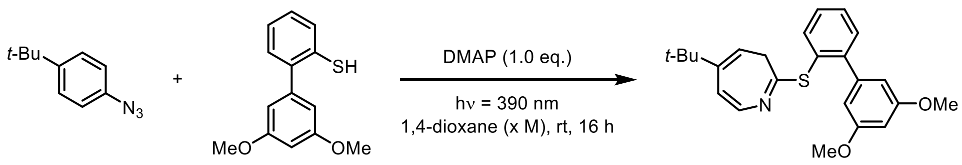

An oven-dried tube equipped with a stirring bar was charged with aryl azide (eq.), thiophenol (eq.) and DMAP (0.1 mmol, 1 eq.). The tube was then capped under air with a Supelco aluminium crimp seal with septum (PTFE/butyl), evacuated and backfilled with argon three times. Anhydrous 1,4-dioxane was added via syringe. The mixture was then purged with Argon for 5 min. and stirred

(600 rpm) under irradiation with purple LEDs (Kessil PR160L-390 nm) for 16 h at room temperature (fan ventilation). Then ethylene carbonate (250 µL, 0.1 M in $CDCl_3$) was added and the solution stirred for 1 min., then 0.3 mL of the solution were placed in an NMR tube, diluted with $CDCl_3$ (0.4 mL) and analyzed by ^1H NMR spectroscopy to determine the NMR yield.

Table 3

Entry	1a:2h eq.	Concentration (M)	Yield S1ah (%)
1	1:1.2	0.05	57
2	1:2.4	0.05	38
3	1.2:1	0.05	53
4	2.4:1	0.05	70
5	2.4:1	0.1	69
6	2.4:1	0.025	27

3.4. Reaction time screening

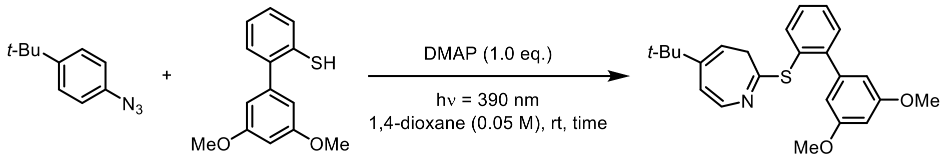

1a 2h S1ah

An oven-dried tube equipped with a stirring bar was charged with aryl azide (0.24 mmol, 2.4 eq.), thiophenol (0.1 mmol, 1.0 eq.) and DMAP (0.1 mmol, 1.0 eq.). The tube was then capped under air with a Supelco aluminium crimp seal with septum (PTFE/butyl), evacuated and backfilled with argon three times. Anhydrous 1,4-dioxane (2 mL, 0.05 M) was added via syringe. The mixture was then purged with Argon for 5 min. and stirred (600 rpm) under irradiation with purple LEDs (Kessil PR160L-390 nm) for x h at room temperature (fan ventilation). Then ethylene carbonate (250 µL, 0.1 M in $CDCl_3$) was added and the solution stirred for 1 min., then 0.3 mL of the solution were placed in an NMR tube, diluted with $CDCl_3$ (0.4 mL) and analyzed by ^1H NMR spectroscopy to determine the NMR yield.

Table 4

Entry	Time	Yield S1ah (%)
1	10´	24
2	30´	45
3	1h	52
4	3h	68
5	6h	73
6	24h	63

3.5. Air and water screening

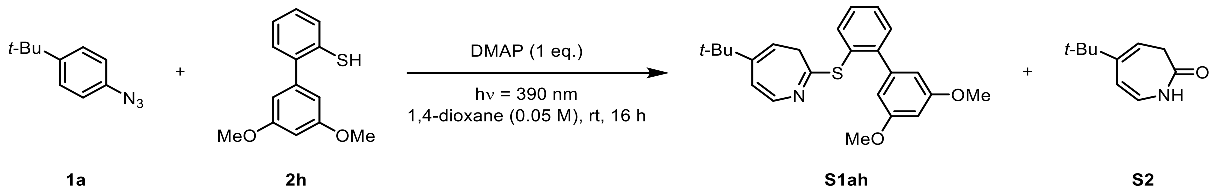

An oven-dried tube equipped with a stirring bar was charged with aryl azide (0.24 mmol, 2.4 eq.), thiophenol (0.1 mmol, 1.0 eq.) and DMAP (x eq.). The tube was then capped under air with a Supelco aluminium crimp seal with septum (PTFE/butyl), evacuated and backfilled with argon three times. Anhydrous 1,4-dioxane (2 mL, 0.05 M) was added via syringe, followed by distilled H_2O (x eq.) when specified. The mixture was then purged with Argon for 5 min. and stirred (600 rpm) under irradiatation with purple LEDs (Kessil PR160L-390 nm) for 16 h at room temperature (fan ventilation). Then ethylene carbonate (250 µL, 0.1 M in $CDCl_3$) was added and the solution stirred for 1 min., then 0.3 mL of the solution were placed in an NMR tube, diluted with $CDCl_3$ (0.4 mL) and analyzed by ^{1}H NMR spectroscopy to determine the NMR yield.

Table 5

Entry	Deviation from Std.	Yield S1ah (%)	Yield S2 (%)
1	Under air	28	-
2	1 equiv. H_2O	63	-
3	10 equiv. H_2O	53	-
4	20 equiv. H_2O	68	-
5	50 equiv. H_2O	73	23
6	100 equiv. H_2O	63	20

3.6. Sodium thiolate screening

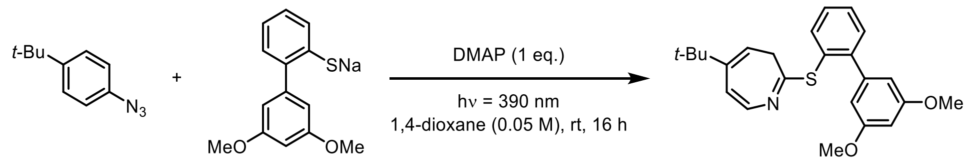

An oven-dried tube equipped with a stirring bar was charged with aryl azide (0.24 mmol, 2.4 eq.), thiophenolate **1c** (0.1 mmol, 1.0 eq.) and DMAP (x eq.). The tube was then capped under air with a Supelco aluminium crimp seal with septum (PTFE/butyl), evacuated and backfilled with argon three times. Anhydrous 1,4-dioxane (2 mL, 0.05 M) was added via syringe. The mixture was then purged with Argon for 5 min. and stirred (600 rpm) under irradiatation with purple LEDs (Kessil PR160L-390 nm) for 16 h at room temperature (fan ventilation). Then ethylene carbonate (250 µL, 0.1 M in $CDCl_3$) was added and the solution stirred for 1 min., then 0.3 mL of the solution were placed in an NMR tube, diluted with $CDCl_3$ (0.4 mL) and analyzed by ^1H NMR spectroscopy to determine the NMR yield (22%).

3.7. Solvent screening

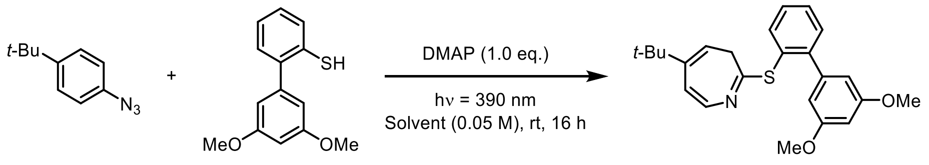

An oven-dried tube equipped with a stirring bar was charged with aryl azide (0.24 mmol, 2.4 eq.), thiophenol (0.1 mmol, 1.0 eq.) and DMAP (0.1 mmol, 1.0 eq.). The tube was then capped under air with a Supelco aluminium crimp seal with septum (PTFE/butyl), evacuated and backfilled with argon three times. Anhydrous solvent (2 mL, 0.05 M) was added via syringe. The mixture was then purged with Argon for 5 min. and stirred (600 rpm) under irradiation with purple LEDs (Kessil PR160L-390 nm) for 16 h at room temperature (fan ventilation). Then ethylene carbonate (250 µL, 0.1 M in CDCl3) was added and the solution stirred for 1 min., then 0.3 mL of the solution were placed in an NMR tube, diluted with CDCl3 (0.4 mL) and analyzed by 1H NMR spectroscopy

to determine the NMR yield. Yield in (*) is referred to the following reaction stoichiometry: aryl azide (0.1 mmol, 1.0 eq.), thiophenol (0.1 mmol, 1 eq.) and DMAP (0.1 mmol, 1.0 eq.).

Table 6

Entry	Solvent	Yield S1ah (%)
1	DMF	-
2	DCE	28 (10*)
3	DCM	35 (19*)
4	MeCN	24 (5*)
5	Toluene	(28*)
6	Et_2O	48 (34*)
7	THF	61 (5*)
8	EtOAc	60 -
9	$PhCF_3$	(33*)
10	MeOH	-
11	HFIP	-
12	MTBE	47 (10*)
13	1,4 – dioxane	70 (35*)
14	DME	0
15	Acetone	45 (10*)
16	MTBE	47
17	CPME	53
18	$PhCF_3$	59
19	Hexane	70

4. Reaction optimization for 1-azido-3,5-dimethylbenzene

4.1. Solvent screening

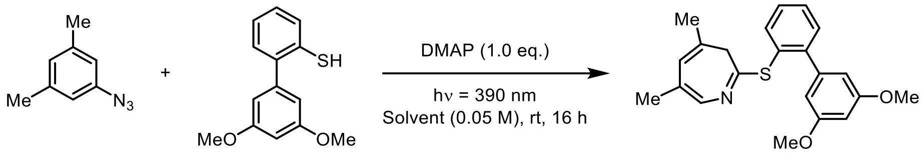

2a **2b** **S1qh**

An oven-dried tube equipped with a stirring bar was charged with aryl azide (0.1 mmol, 0.1 eq.), thiophenol (0.1 mmol, 1.0 eq.) and DMAP (0.1 mmol, 1.0 eq.). The tube was then capped under air with a Supelco aluminium crimp seal with septum (PTFE/butyl), evacuated and backfilled with argon three times. Anhydrous solvent (2 mL, 0.05 M) was added via syringe. The mixture was then purged with Argon for 5 min. and stirred (600 rpm) under irradiation with purple LEDs (Kessil PR160L-390 nm) for 16 h at room temperature (fan ventilation). Then ethylene carbonate (250 µL, 0.1 M in $CDCl_3$) was added and the solution stirred for 1 min., then 0.3 mL of the solution were placed in an NMR tube, diluted with $CDCl_3$ (0.4 mL) and analyzed by ^{1}H NMR spectroscopy to determine the NMR yield

Table 7

Entry	Solvent	Yield S1qh (%)
1	Et_2O	61
2	1,4 - dioxane	61
3	$PhCF_3$	59
4	THF	8
5	Hexane	53
[b]6	1,4 - dioxane	81

[b]Aryl azide (2.4 eq.)

4.2. Stoichiometry of thiol screening

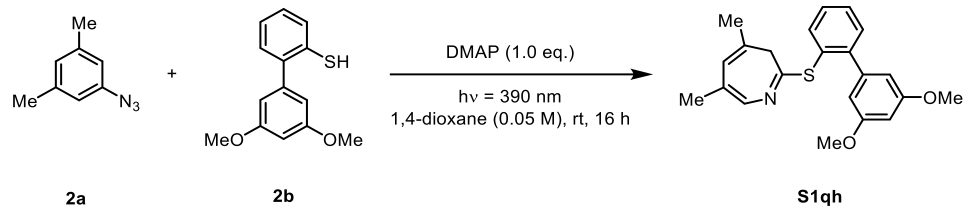

An oven-dried tube equipped with a stirring bar was charged with aryl azide (0.1 mmol, 1.0 eq.) thiophenol (x eq.) and DMAP (0.1 mmol, 1.0 eq.). The tube was then capped under air with a Supelco aluminium crimp seal with septum (PTFE/butyl), evacuated and backfilled with argon three times. Anhydrous 1,4-dioxane (2 mL, 0.05 M) was added via syringe. The mixture was then purged with Argon for 5 min. and stirred (600 rpm) under irradiation with purple LEDs (Kessil PR160L-390 nm) for 16 h at room temperature (fan ventilation). Then ethylene carbonate (250 µL, 0.1 M in $CDCl_3$) was added and the solution stirred for 1 min., then 0.3 mL of the solution were placed in an NMR tube, diluted with $CDCl_3$ (0.4 mL) and analyzed by ^1H NMR spectroscopy to determine the NMR yield.

Table 8

Entry	2b eq.	Yield S1qh (%)
1	1	65
2	2	70
3	3	75
4	4	80
5	5	81

4.3. Stoichiometry of DMAP screening

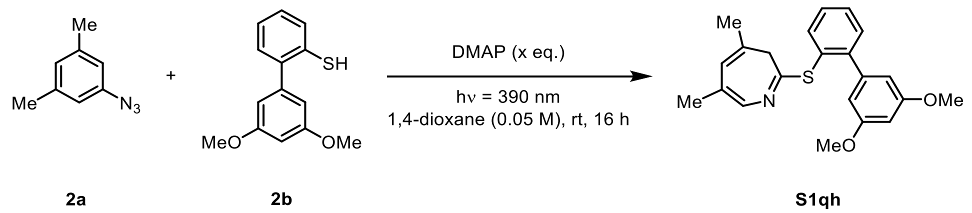

An oven-dried tube equipped with a stirring bar was charged with aryl azide (0.24 mmol, 2.4 eq.) thiophenol (0.1 mmol, 1.0 eq.) and DMAP (x eq.). The tube was then capped under air with a Supelco aluminium crimp seal with septum (PTFE/butyl), evacuated and backfilled with argon

three times. Anhydrous 1,4-dioxane (2 mL, 0.05 M) was added via syringe. The mixture was then purged with Argon for 5 min. and stirred (600 rpm) under irradiation with purple LEDs (Kessil PR160L-390 nm) for 16 h at room temperature (fan ventilation). Then ethylene carbonate (250 µL, 0.1 M in $CDCl_3$) was added and the solution stirred for 1 min., then 0.3 mL of the solution were placed in an NMR tube, diluted with $CDCl_3$ (0.4 mL) and analyzed by ^1H NMR spectroscopy to determine the NMR yield.

Table 9

Entry	x eq.	Yield S1qh (%)
1	0.1	28
2	0.3	36
3	0.5	42
4	0.7	53
5	0.9	66
6	1.5	57
7	2	60
8	3	61
9	4	46
10	5	49
11	6	41
12	10	49

5. Study of regioselectivity in the ring expansion of meta-substituted azides

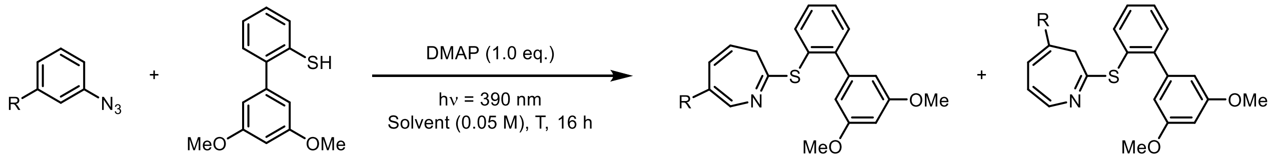

1u 2h A B

Table 10

Entry	R	Solvent	T (°C)	Yield A (%)	Yield B (%)	Ratio
1	F	1,4-dioxane	rt	50	25	2 :1
2	F	1,4-dioxane	70	65	31	2 :1
3	F	TFT	rt	63	28	2.3 :1
4	F	TFT	70	62	36	1.7 :1
5	OMe	1,4-dioxane	rt	71	8	9 :1
6	OMe	1,4-dioxane	70	79	11	7 :1
7	OMe	TFT	rt	74	18	4.1 :1
8	OMe	TFT	70	82	10	8.2 :1
9	CF_3	1,4-dioxane	rt	23	22	1 :1
10	CF_3	1,4-dioxane	70	25	38	1 :1.4
11	CF_3	TFT	rt	23	30	1 :1.3
12	CF_3	TFT	70	24	32	1.3 :1
13	Me	1,4-dioxane	rt	56	28	2 :1
14	Me	1,4-dioxane	70	50	36	1.4 :1
15	Me	TFT	rt	43	27	1.6 :1
16	Me	TFT	70	46	29	1.6 :1
17	*t*-Bu	1,4-dioxane	rt	30	26	1.1 :1
18	*t*-Bu	1,4-dioxane	70	38	18	2 :1
19	*t*-Bu	TFT	rt	49	46	1 :1
20	*t*-Bu	TFT	70	45	11	4.1 :1

Identification of regioisomers: the ratio between the two regioisomers was based on the integral ratio in the crude NMR spectra; after purification (as single isomer or mixture) structural identification was based on multiplicity of the azepane signals. Additionally, for R=F and R=OMe

the assignation was supported by a clear COSY crosspeak of the azepane ring CH_2 in the presence of an adjacent proton. For R=Me and R=CF_3, the ring CH_2 signal is too broad to observe any crosspeak.

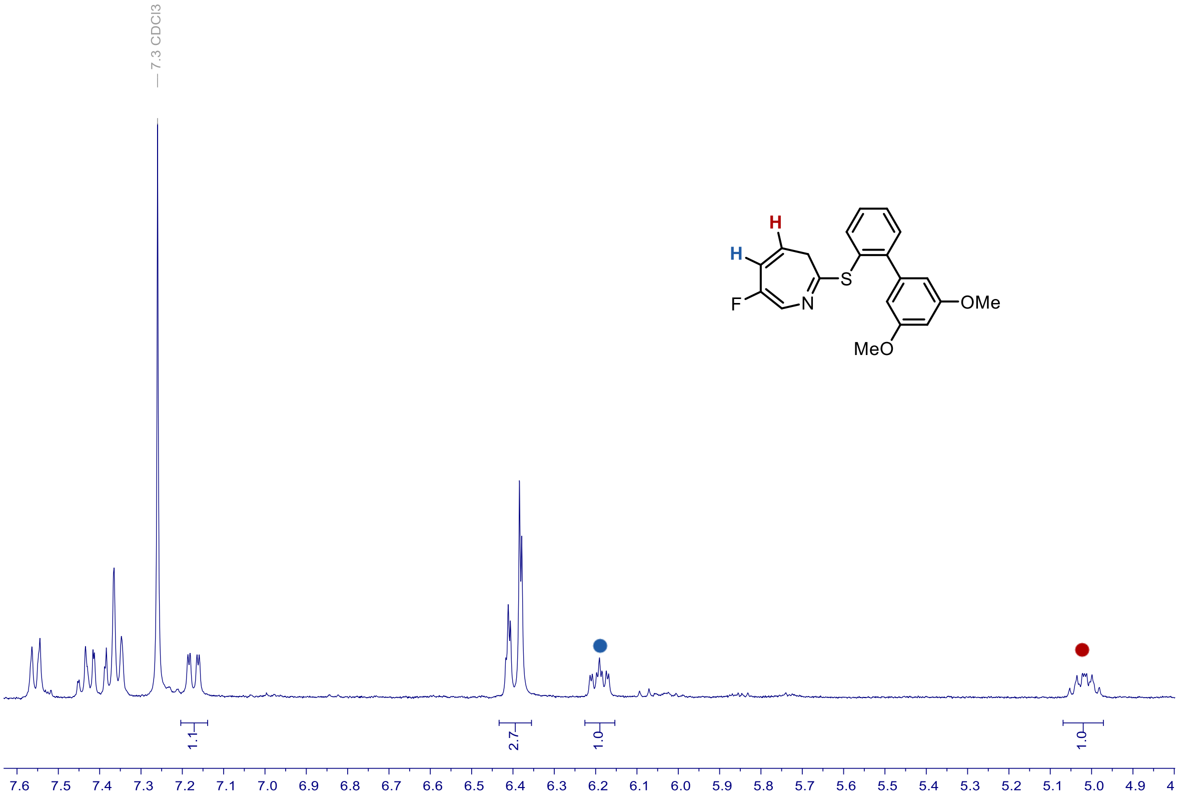

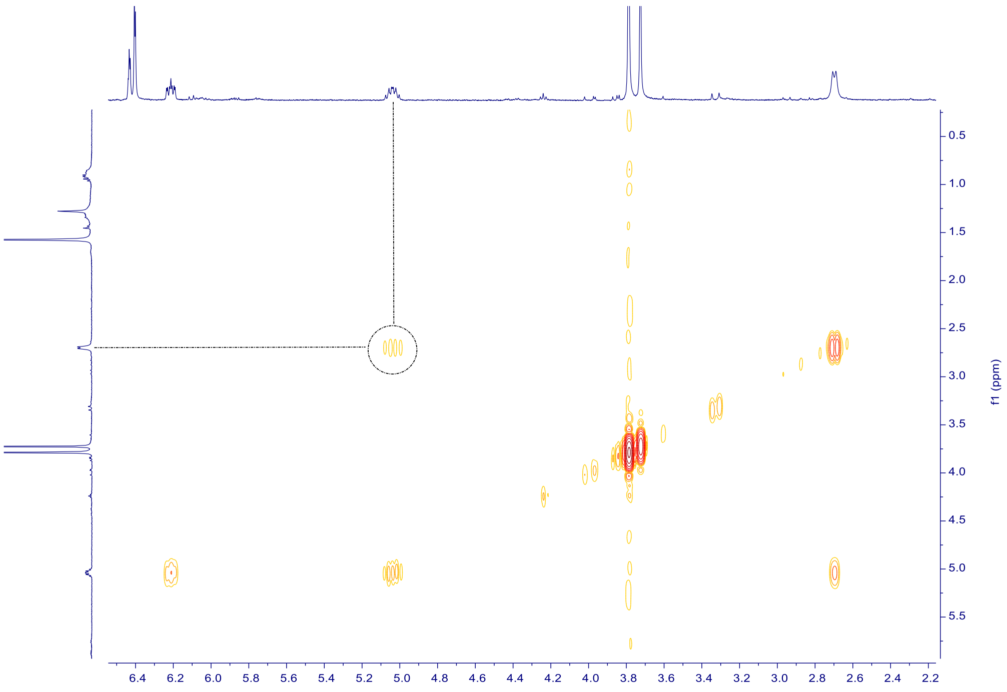

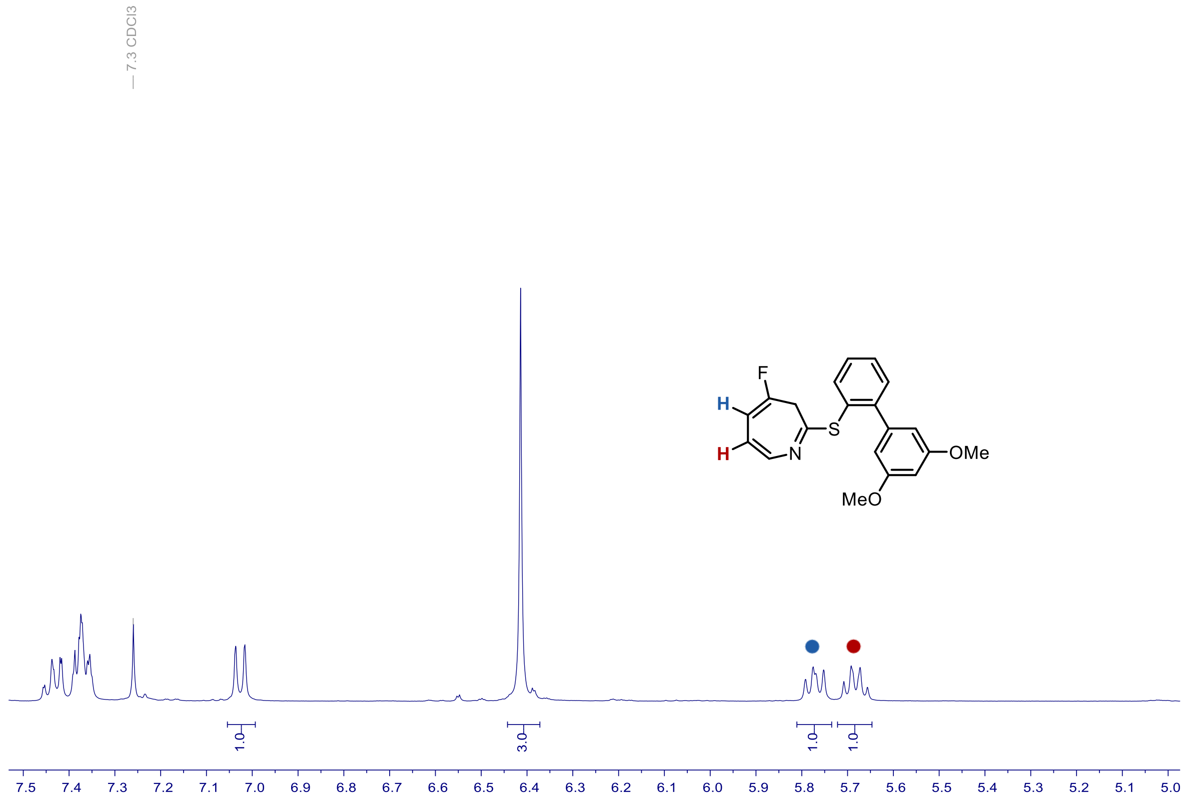
7.3 CDCl3
F
H
H
S
N
OMe
MeO
1.0
3.0
1.0
1.0
7.5 7.4 7.3 7.2 7.1 7.0 6.9 6.8 6.7 6.6 6.5 6.4 6.3 6.2 6.1 6.0 5.9 5.8 5.7 5.6 5.5 5.4 5.3 5.2 5.1 5.0

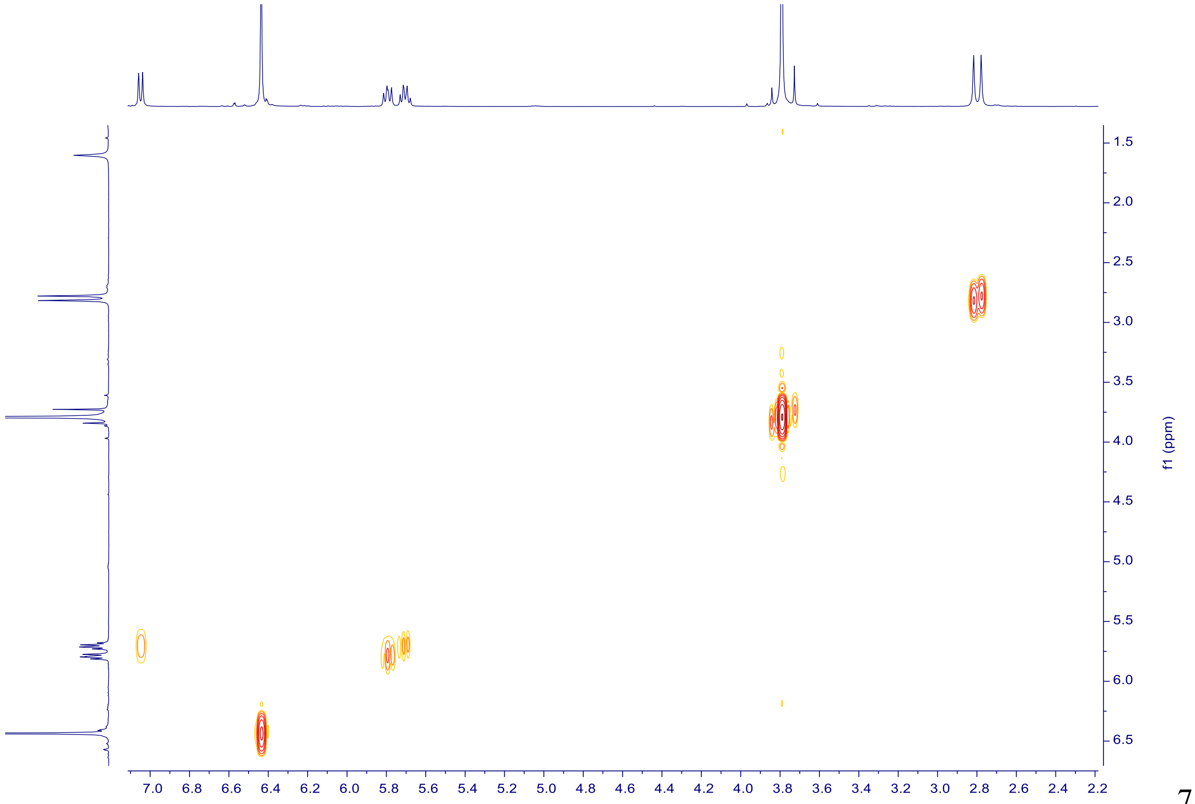
7.0 6.8 6.6 6.4 6.2 6.0 5.8 5.6 5.4 5.2 5.0 4.8 4.6 4.4 4.2 4.0 3.8 3.6 3.4 3.2 3.0 2.8 2.6 2.4 2.2
1.5 2.0 2.5 3.0 3.5 4.0 4.5 5.0 5.5 6.0 6.5
f1 (ppm)

7

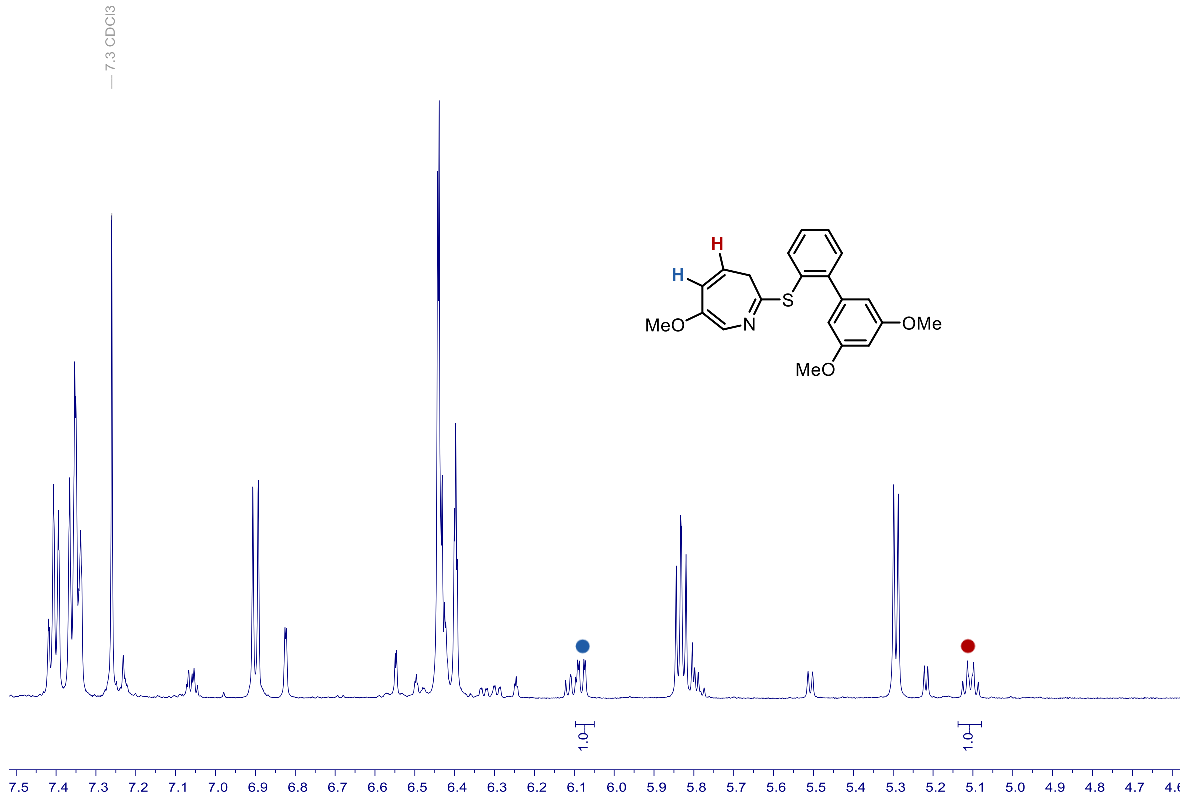

7.3 CDCl3
H
H
MeO
N
S
OMe
MeO
1.0
1.0
7.5 7.4 7.3 7.2 7.1 7.0 6.9 6.8 6.7 6.6 6.5 6.4 6.3 6.2 6.1 6.0 5.9 5.8 5.7 5.6 5.5 5.4 5.3 5.2 5.1 5.0 4.9 4.8 4.7

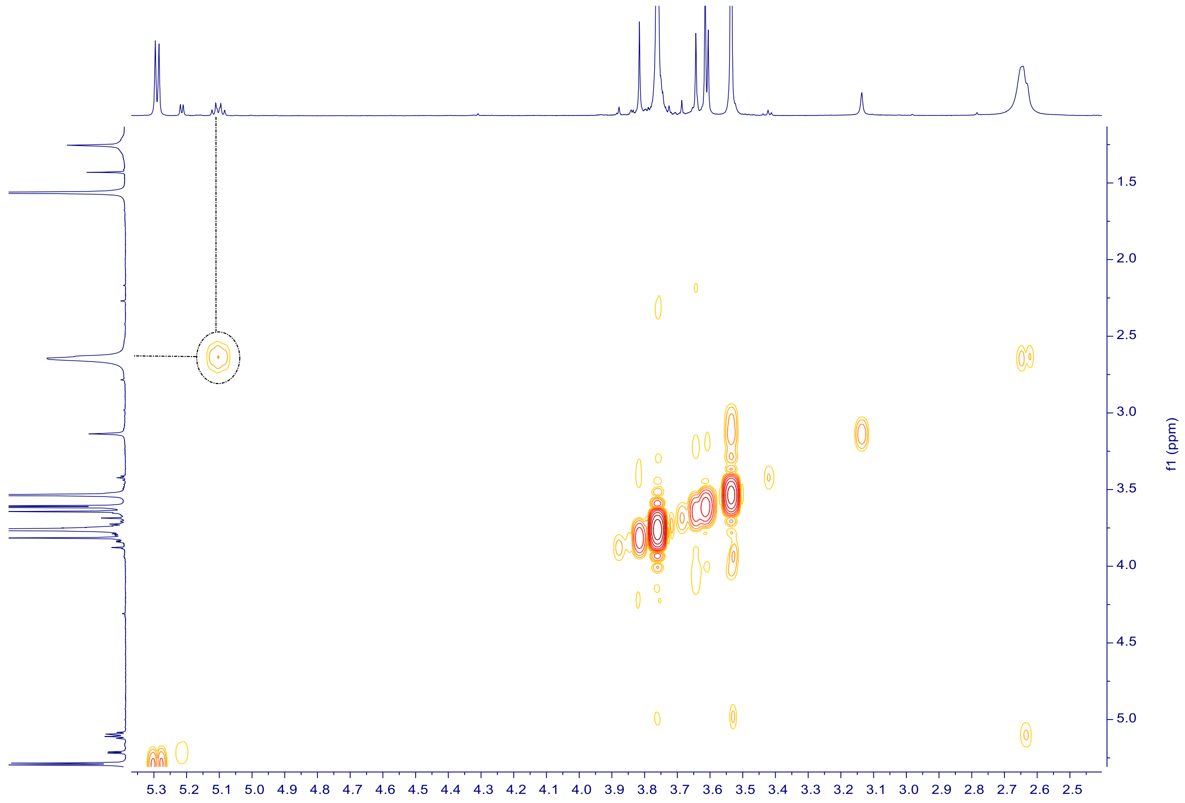

1.5
2.0
2.5
3.0
3.5
4.0
4.5
5.0
f1 (ppm)
5.3 5.2 5.1 5.0 4.9 4.8 4.7 4.6 4.5 4.4 4.3 4.2 4.1 4.0 3.9 3.8 3.7 3.6 3.5 3.4 3.3 3.2 3.1 3.0 2.9 2.8 2.7 2.6 2.5

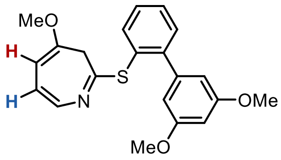
MeO
H
H
N
S
OMe
MeO

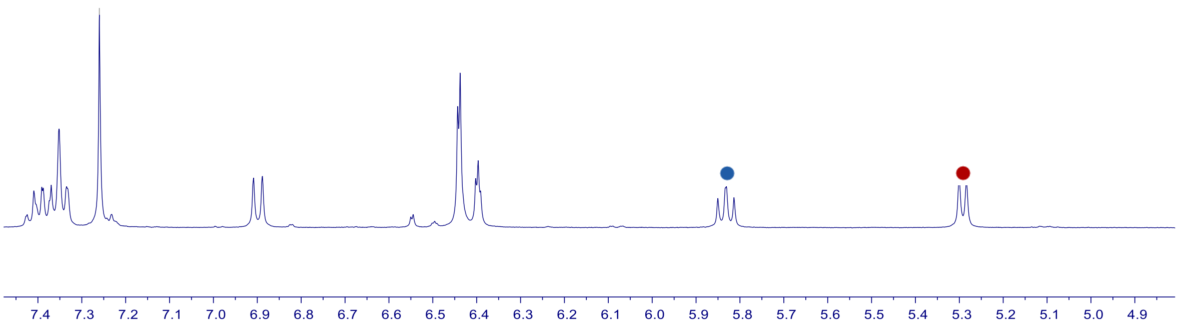
7.3 CDCl3
7.4
7.3
7.2
7.1
7.0
6.9
6.8
6.7
6.6
6.5
6.4
6.3
6.2
6.1
6.0
5.9
5.8
5.7
5.6
5.5
5.4
5.3
5.2
5.1
5.0
4.9

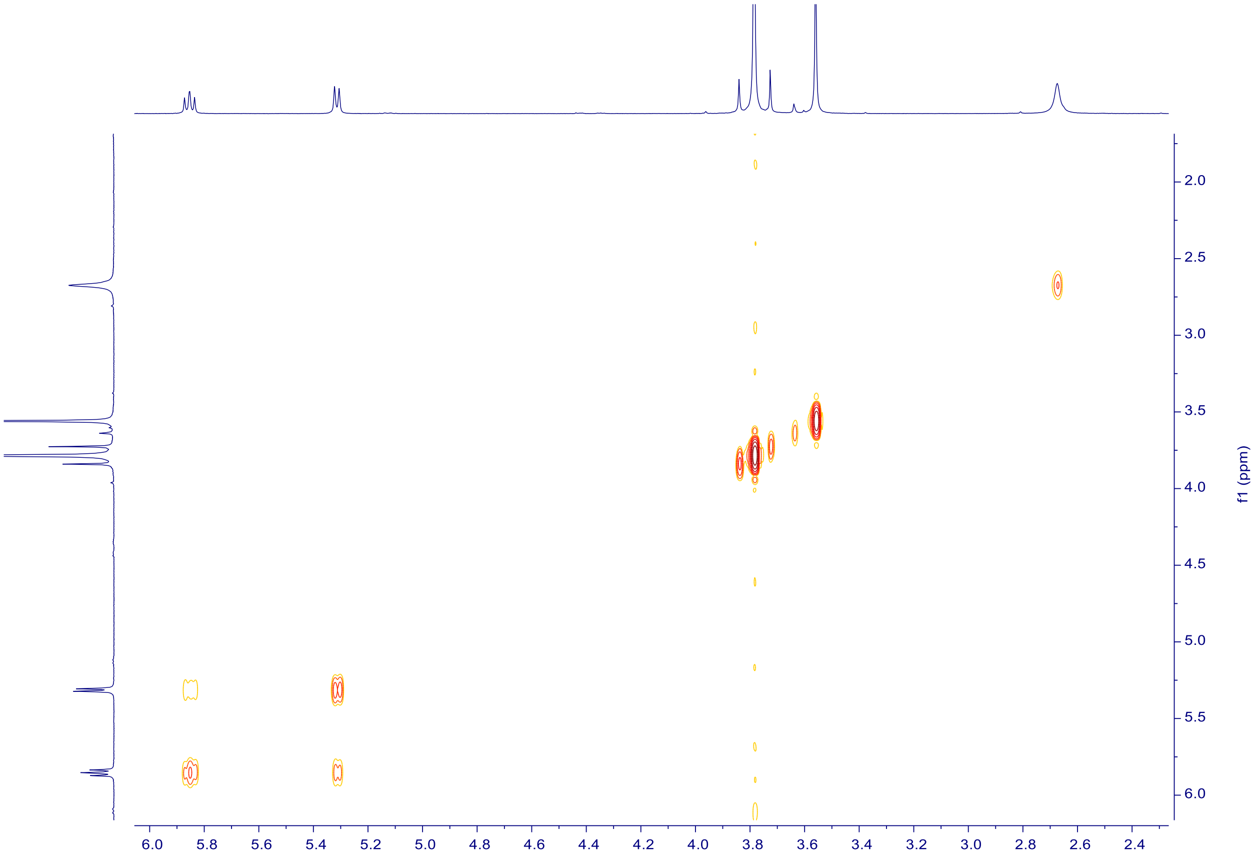
f1 (ppm)
2.0
2.5
3.0
3.5
4.0
4.5
5.0
5.5
6.0
6.0
5.8
5.6
5.4
5.2
5.0
4.8
4.6
4.4
4.2
4.0
3.8
3.6
3.4
3.2
3.0
2.8
2.6
2.4

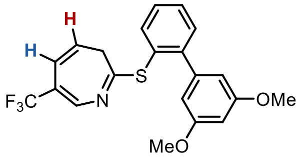
H
H
F_3C
N
S
OMe
MeO

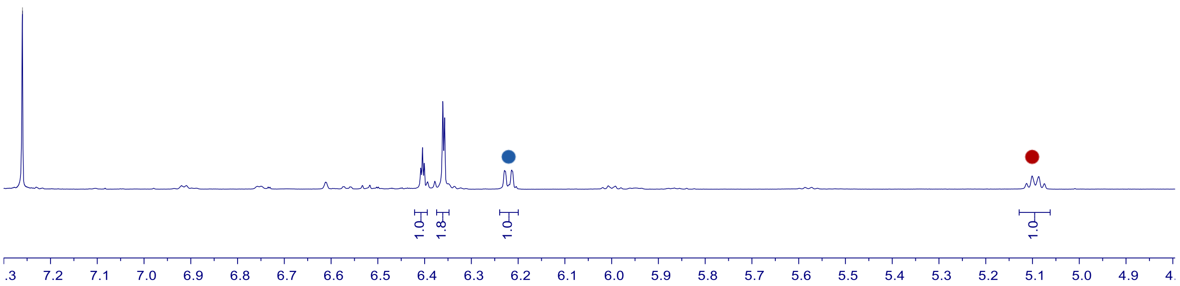
7.3 CDCl3
1.0
1.8
1.0
1.0
.3 7.2 7.1 7.0 6.9 6.8 6.7 6.6 6.5 6.4 6.3 6.2 6.1 6.0 5.9 5.8 5.7 5.6 5.5 5.4 5.3 5.2 5.1 5.0 4.9 4.

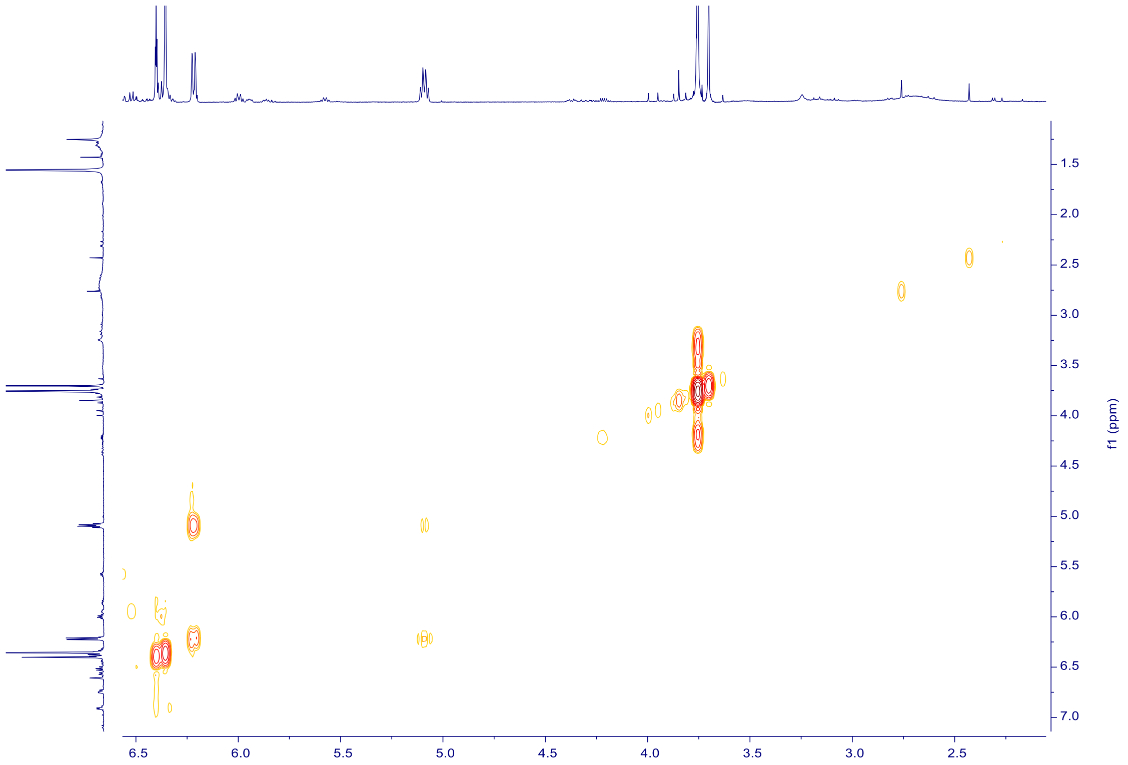
6.5 6.0 5.5 5.0 4.5 4.0 3.5 3.0 2.5
1.5 2.0 2.5 3.0 3.5 4.0 4.5 5.0 5.5 6.0 6.5 7.0
f1 (ppm)

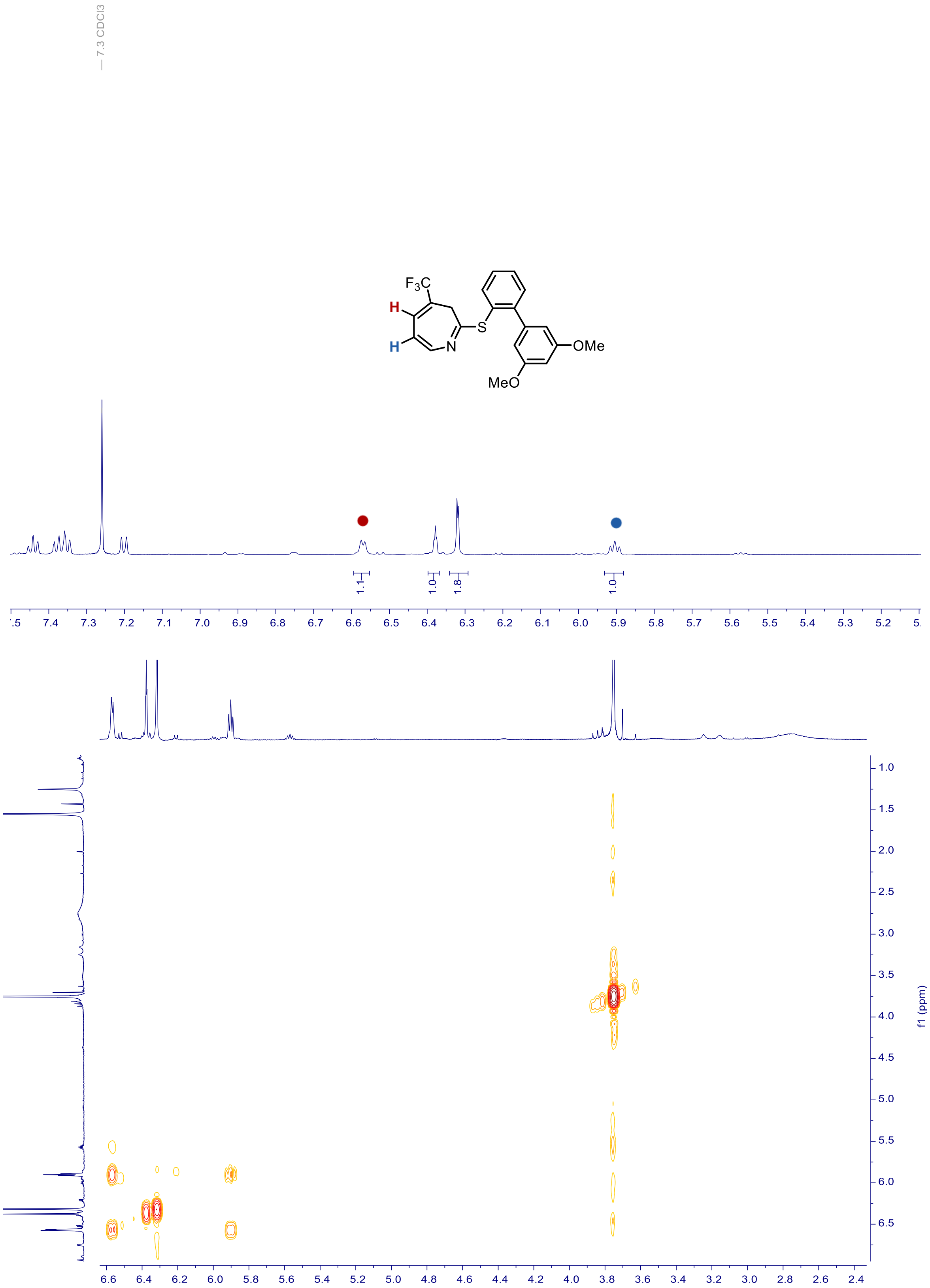
7.3 CDCl3
F3C
H
H
S
N
OMe
MeO
1.1
1.0
1.8
1.0
f1 (ppm)

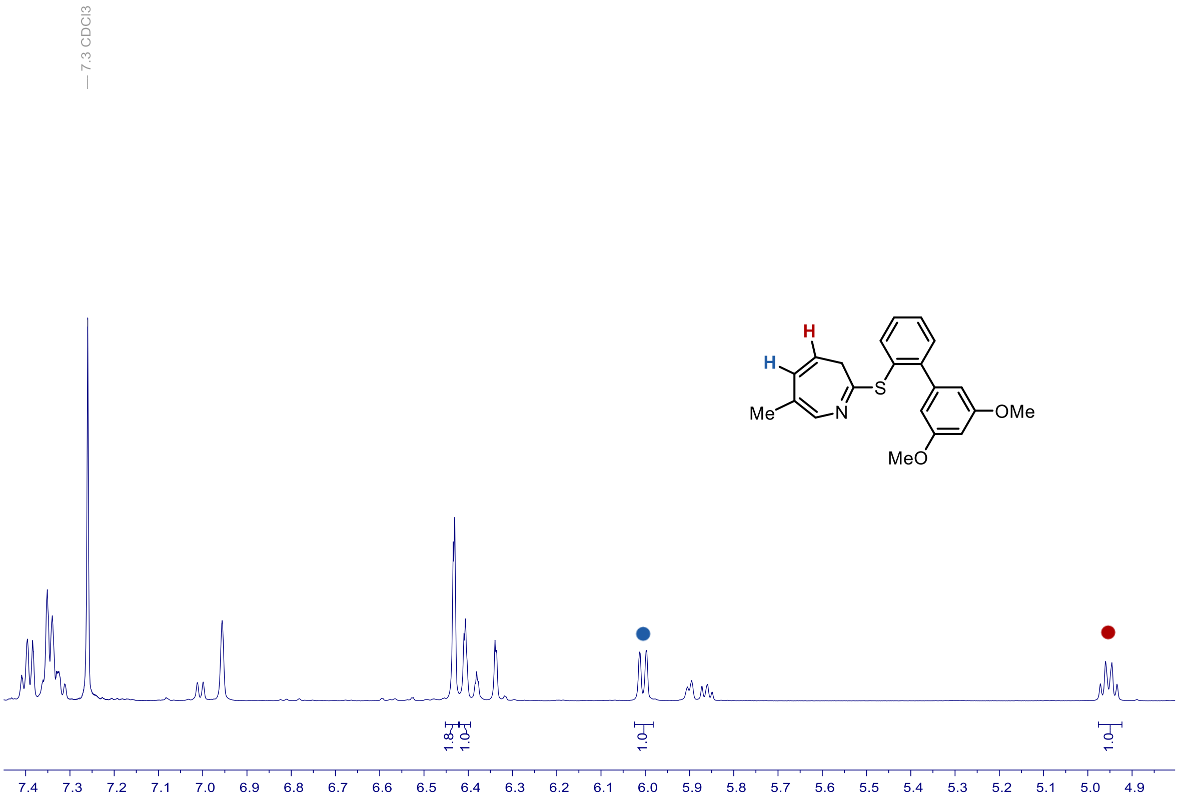
— 7.3 CDCl3
H
H
Me
N
S
OMe
MeO
1.8
1.0
1.0
1.0
7.4
7.3
7.2
7.1
7.0
6.9
6.8
6.7
6.6
6.5
6.4
6.3
6.2
6.1
6.0
5.9
5.8
5.7
5.6
5.5
5.4
5.3
5.2
5.1
5.0
4.9

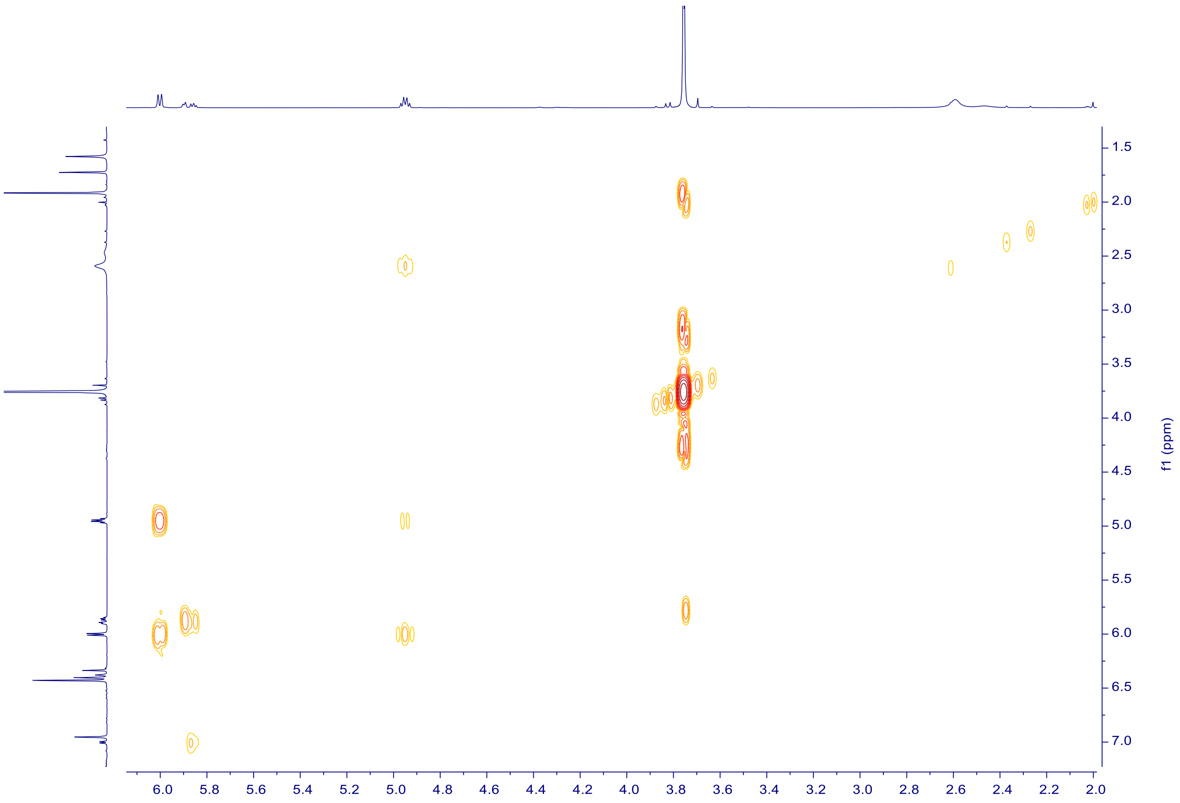
f1 (ppm)

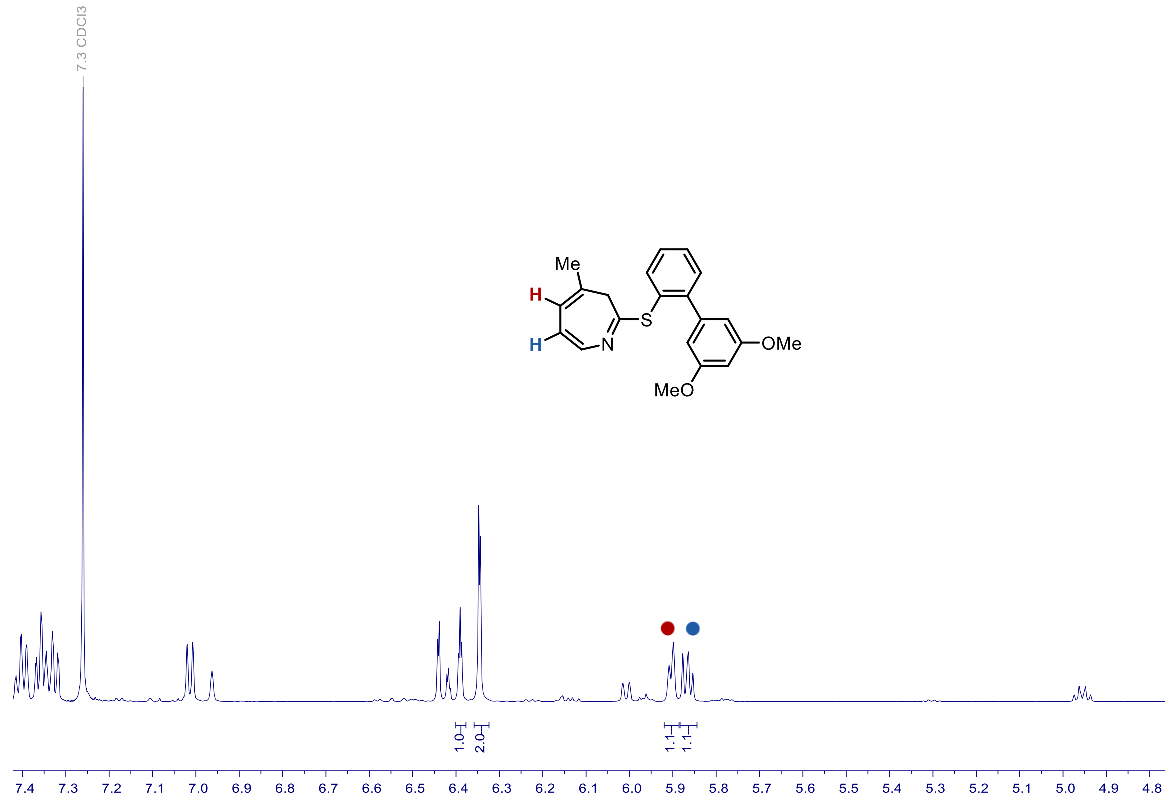
7.3 CDCl3
Me
H
H
S
N
OMe
MeO
1.0
2.0
1.1
1.1

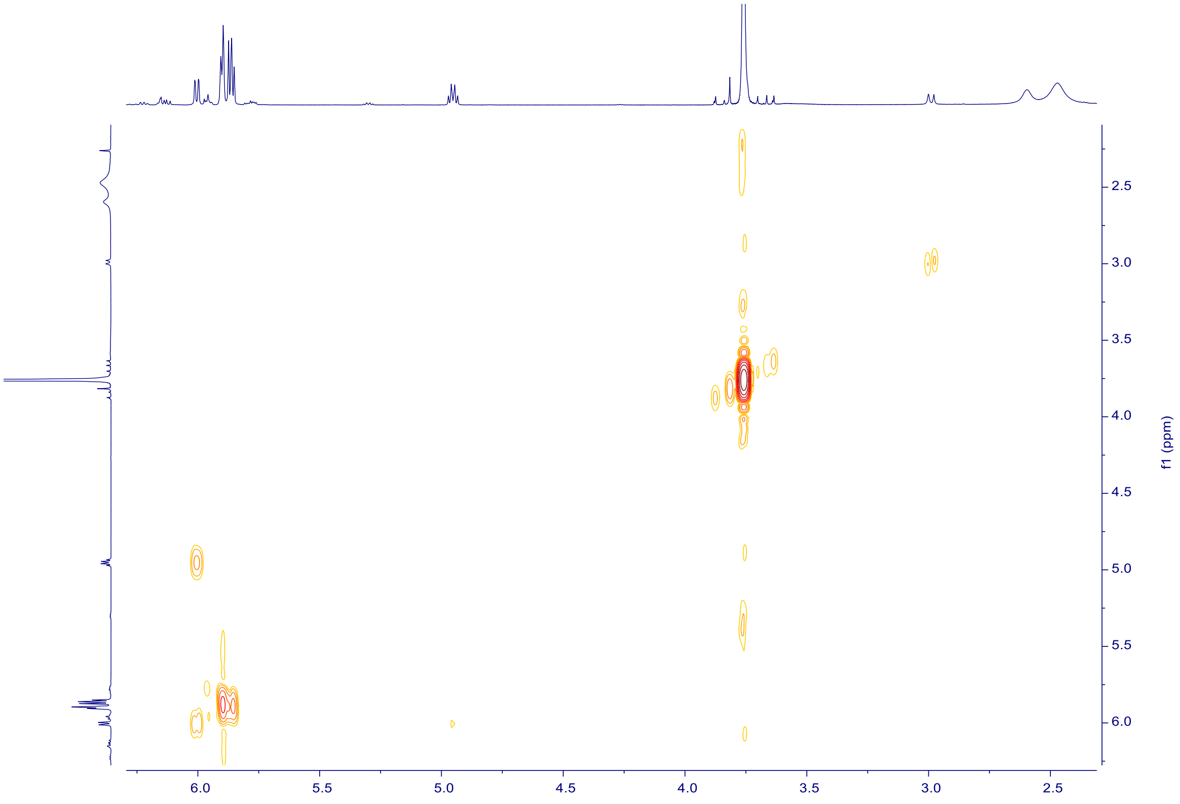
f1 (ppm)

6. Ni-Raney desulfurization studies

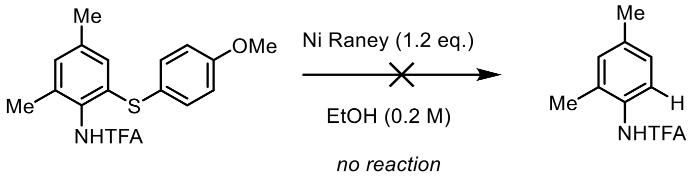

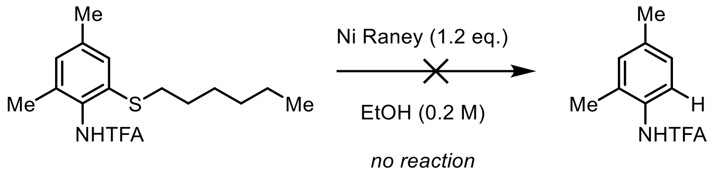

7. Reaction optimization for cyclization to sulphonium salt

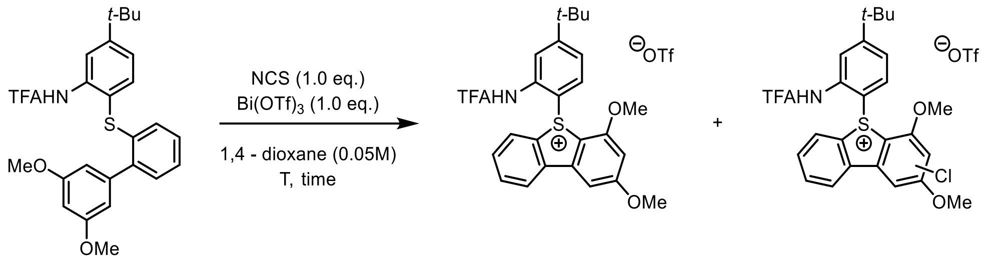

3ah 4ah 4ah´

An oven-dried tube equipped with a stirring bar was charged with NCS (1.0 eq.) and $Bi(OTf)_3$ (1.0 eq.). The tube was then capped under air with a Supelco aluminium crimp seal with septum (PTFE/butyl), evacuated and backfilled with argon three times. After the addition of anhydrous 1,4-dioxane (0.1 M), the mixture was vigorously stirred while a solution of **S2** in dry 1,4-dioxane (0.1 M) was added via syringe. The reaction was then stirred at 1300 rpm for the specified time and at the specified temperature. Upon consumption of the starting material (TLC analysis), the solvent was evaporated and the residue redissolved in CD_3OD (1 mL). Ethylene carbonate (250 µL, 0.1 M in $CDCl_3$) was added and the solution stirred for 1 min., then 0.6 mL of the solution were placed in an NMR tube and analyzed by ^1H NMR spectroscopy to determine the NMR yield.

The sulphonium salt was then purified without Flash column chromatography: the suspension was filtered through Celite® and the filtrate evaporated under vacuum. The residue was dissolved in the minimum amount of $CHCl_3$, and the product was precipitated by addition of Et_2O as an off-white solid. The solid was recollected by filtration and further washed with Et_2O.

Table 11

Entry	NCS:Bi(OTf)$_3$	Time	T (°C)	Yield 4ah (%)	Yield 4ah´ (%)	RSM(%)
1	1:1	6	RT	51	0	28
2	1:1	16	RT	45	0	29
3	1:1	6	40	48	0	25
4	1:1	6	80	64	0	13
5	3:3	6	RT	51	39	0
6	1:3	6	RT	55	12	18
7	3:1	6	RT	51	19	15
8	1.5:1	6	RT	46	0	30
9	1:1.5	6	RT	74	0	0
10	2:2	6	RT	80	0	0
11	1:1[a]	6	RT	87	0	0
12	1:1[b]	6	RT	90	0	0

[a]1,4-dioxane (0.066 M) + MeCN (0.066 M); [b]MeCN (0.033 M).

8. Reaction optimization for photochemical sulphonium salt cleavage

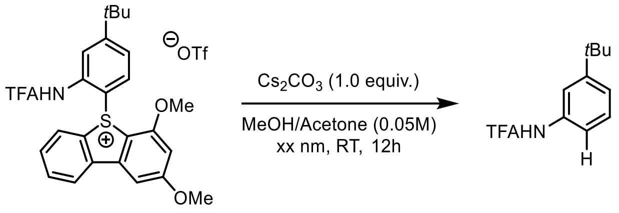

Sulphonium salt (0.08 mmol, 1.0 eq.) was weighed in an oven-dried tube equipped with a stirring bar. Cs_2CO_3 (0.08 mmol, 1.0 eq.) was added and the tube was then capped under air with a Supelco aluminium crimp seal with septum (PTFE/butyl), evacuated and backfilled with argon three times. Anhydrous MeOH (0.1 M) and anhydrous Acetone (0.1 M) were added via syringe. The mixture was then purged with Argon for 5 min. and stirred (600 rpm) under irradiation with purple LEDs (Kessil PR160L-390 nm) for 12 h at room temperature (fan ventilation). Ethylene carbonate (250 µL, 0.1 M in $CDCl_3$) was added and the solution stirred for 1 min., then 0.3 mL of the solution was placed in an NMR tube, diluted with $CDCl_3$ (0.4 mL) and analyzed by ^{1}H NMR spectroscopy to determine the NMR yield.

Table 12

Entry	Wavelength (nm)	Yield 5a (%)
1	390	98
2	427	97
3	440	95
4[a]	390	32
5[a]	427	31
6[a]	440	30

[a]No Cs_2CO_3

9. General procedure

9.1. Step 1 for thiol preparation (TP1)

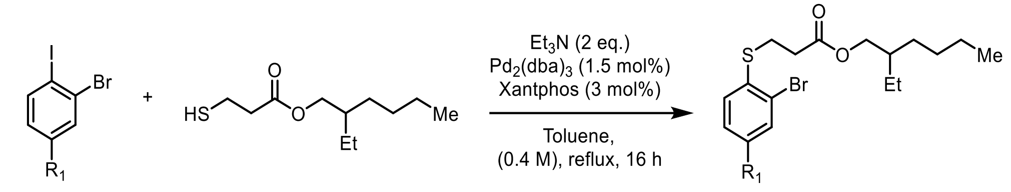

S4a-c 1 eq.
a, R_1=OMe
b, R_1=CF_3
c, R_1=H

S5 1.1 eq.

S6a-c

Under N_2 atmosphere, to a Schlenk tube were added the suitable 2-bromo-1-iodobenzene (1 eq.), tris(dibenzylideneacetone)dipalladium(0) (1.5 mol%), Xantphos (3.0 mol%) and 2-ethylhexyl 3-mercaptopropanoate (1.1 equiv.). The vessel was evacuated and backfilled with argon (x3). Toluene (0.4 M) was added, followed by Et_3N (2.0 eq.). The resulting mixture was degassed with argon for 5 min. and then heated to reflux for 16 h. The reaction was then allowed to cool down to room temperature. The mixture was filtered over a pad of Celite® and concentrated in vacuo. The resulting yellow oils were then purified by flash column chromatography.

9.2. Step 2 for thiol preparation (TP2)

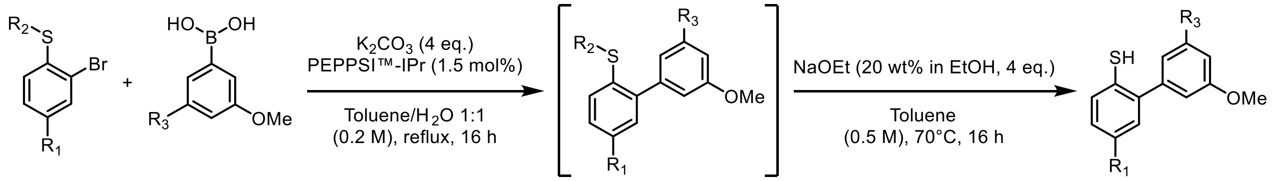

S6a-c 1 eq.
a, R_1=OMe
b, R_1=CF_3
c, R_1=H

S7d-e 1.3 eq.
d, R_3=OMe
e, R_3=H

R_2 =

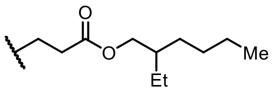

S8ad-cd 1 eq.
ad, R_1=OMe; R_3=OMe;
ae, R_1=OMe; R_3=H ;
bd, R_1=CF_3; R_3=OMe;
be, R_1=CF_3; R_3=H;
cd, R_1=H; R_3=H;

2d-h

Under N_2 atmosphere, to a schlenk tube were added the suitable thioether (1 eq.) and toluene (0.4 M), followed by a solution of potassium carbonate (4 eq.) in water (0.4 M) and the suitable boronic acid (1.3 eq.). The reaction vessel was degassed with argon under vigorous stirring. PEPPSI™-IPr catalyst (1.5 mol%) was added and after additional degassing the resulting mixture was heated to reflux for 16 h. The reaction was allowed to cool down to room temperature. The mixture was

filtered over a pad of Celite®. The organic phase was washed with saturated potassium carbonate aqueous solution (2 × 50 mL), dried over magnesium sulfate and concentrated in vacuo.

Under N_2 atmosphere, to a schlenk tube were added toluene (0.5 M) and the suitable biaryl intermediate (1 eq.), followed by NaOEt (21 wt% in denatured EtOH, 4 eq). The resulting mixture was heated at 70° C for 16 h. After cooling down to room temperature, the reaction mixture was diluted with EtOAc and then acidified to pH 4 using 1 M aq. HCl. After separation, the organic phase was washed with brine, dried over anhydrous magnesium sulfate, filtered and concentrated in vacuo. The crude were purified by flash column chromatography and the products were dried under high vacuum overnight prior to use for further reactions.

The final thiophenols can be purified as well through acid/base extraction using 2M aqueous NaOH and Et_2O as the organic phase.

9.3. Azide preparation from anilines (AP1)

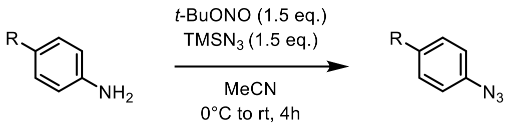

S9 **1a-h, k, m-q, r-x**

A solution of aniline (1.0 equiv.) in MeCN (8.00 mL) in an open flask was cooled to 0°C in an ice bath. To this stirred mixture was added *tert*-butylnitrite (1.5 equiv) followed by trimethylsilyl azide (1.5 equiv) dropwise. After 1 h, the resulting solution was allowed to warm up to room temperature and stirred for an additional 3 h. Upon consumption of the starting material (TLC analysis), the reaction mixture was concentrated in vacuo and the crude residue filtered through a pad of silica gel.

9.4. Azide preparation from aryl bromides (AP2)

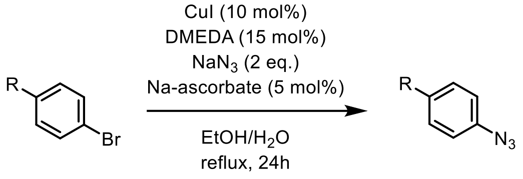

In a round bottom flask under air, to a solution of aryl bromide (1 eq.) in a 3:7 H_2O/EtOH mixture were added DMEDA (15 mol%), CuI (10 mol%), NaN_3 (2 eq.) and Na-ascorbate (5 mol%). The reaction vessel was sealed and the solution was purged with Ar for 5 min. The reaction mixture was heated to reflux for 24 h, then cooled to room temperature and extracted with EtOAc (3x). The organics were washed with brine, dried with $MgSO_4$, concentrated under vacuo and purified by flash column chromatography (Silica gel, Pentane 100% to Pentane/EtoAc 80:10).

9.5. General Procedure 1 (GP1)

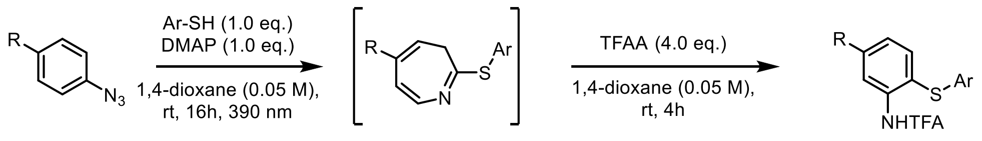

An oven-dried tube equipped with a stirring bar was charged with aryl azide (0.24 mmol, 2.4 eq.), thiophenol (0.1 mmol, 1 eq.) and DMAP (0.1 mmol, 1 eq.). The tube was then capped under air with a Supelco aluminium crimp seal with septum (PTFE/butyl), evacuated and backfilled with argon three times. Anhydrous 1,4-Dioxane (2 mL, 0.05 M) was added via syringe. The mixture was then purged with Argon for 5 min. and stirred (600 rpm) under irradiation with purple LEDs (Kessil PR160L-390 nm) for 16 h at room temperature (fan ventilation). Then TFAA (0.4 mmol, 4 eq.) was added via syringe and the resulting mixture was stirred for another 4 h at room temperature. Ethylene carbonate (250 µL, 0.1 M in $CDCl_3$) was added and the solution stirred for 1 min., then 0.3 mL of the solution were placed in an NMR tube, diluted with $CDCl_3$ (0.4 mL) and analyzed by ^{1}H NMR spectroscopy to determine the NMR yield. The crude mixtures were purified by flash column chromatography (Silica gel, Pentane 100% to Pentane/Et_2O 80:20).

9.6. General Procedure 2 (GP2)

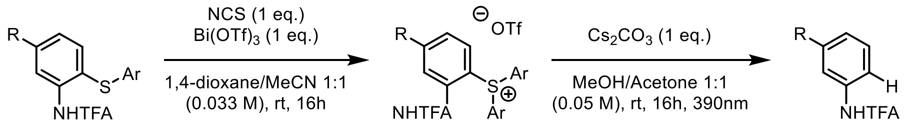

3ad-ah, 3bh-yh 1 eq. **4ad-ah, 4bh-yh** 1 eq. **5a-y**

a. An oven-dried tube equipped with a stirring bar was charged with NCS (1 eq.) and $Bi(OTf)_3$ (1 eq.). The tube was then capped under air with a Supelco aluminium crimp seal with septum (PTFE/butyl), evacuated and backfilled with argon three times. After the addition of anhydrous MeCN (0.066 M), the mixture was vigorously stirred while a solution of in dry 1,4-dioxane (0.066 M) was added via syringe. The reaction was then stirred at 1300 rpm for 6 h at room temperature. Upon consumption of the starting material (TLC analysis), the solvent was evaporated and the residue redissolved in $MeOH/CHCl_3$. The suspension was filtered through Celite® and the filtrate evaporated under vacuum. The residue was dissolved in the minimum amount of $CHCl_3$, and the product was precipitated by addition of Et_2O as an off-white solid. The solid was recollected by filtration and further washed with Et_2O.

b. The sulfonium salt (1 eq.) was weighed in an oven-dried tube equipped with a stirring bar. Cs_2CO_3 (1 eq.) was added and the tube was then capped under air with a Supelco aluminium crimp seal with septum (PTFE/butyl), evacuated and backfilled with argon three times. Anhydrous MeOH (0.1 M) and anhydrous Acetone (0.1 M) were added via syringe. The mixture was then purged with Argon for 5 min. and stirred (600 rpm) and irradiated with purple LEDs (Kessil PR160L-390 nm) for 12 h at room temperature (fan ventilation). Ethylene carbonate (250 μL, 0.1 M in $CDCl_3$) was added and the solution stirred for 1 min., then 0.3 mL of the solution were placed in an NMR tube, diluted with $CDCl_3$ (0.4 mL) and analyzed by ^{1}H NMR spectroscopy to determine the NMR yield. The crude mixtures were purified by flash column chromatography (Silica gel, Pentane 100% to Pentane/Et_2O 80:10).

9.7. General Procedure 3 (GP3)

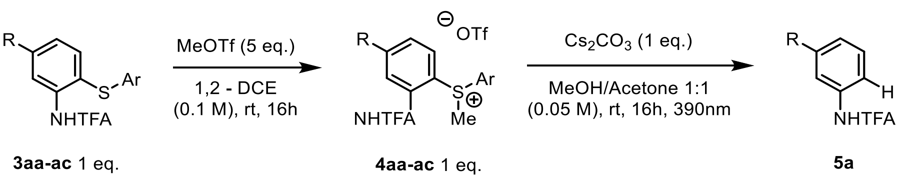

An oven-dried tube equipped with a stirring bar was charged with **S8**. The tube was then capped under air with a Supelco aluminium crimp seal with septum (PTFE/butyl), evacuated and backfilled with argon three times. Then 1,2-DCE (0.1 M) was added by syringe and the mixture was stirred at room temperature for 16 h. Upon consumption of the starting material (TLC analysis), the solvent was evaporated under vacuum. The residue was dissolved in the minimum amount of $CHCl_3$, and the product was precipitated by addition of Et_2O as an off-white solid. The solid was recollected by filtration and further washed with Et_2O.

S9 (1 eq.) was weighed in an oven-dried tube equipped with a stirring bar. Cs_2CO_3 (1 eq.) was added and the tube was then capped under air with a Supelco aluminium crimp seal with septum (PTFE/butyl), evacuated and backfilled with argon three times. Anhydrous MeOH (0.1 M) and anhydrous Acetone (0.1 M) were added via syringe. The mixture was then purged with Argon for 5 min. and stirred (600 rpm) and irradiated with purple LEDs (Kessil PR160L-390 nm) for 12 h at room temperature (fan ventilation). Ethylene carbonate (250 µL, 0.1 M in $CDCl_3$) was added and the solution stirred for 1 min., then 0.3 mL of the solution were placed in an NMR tube, diluted with $CDCl_3$ (0.4 mL) and analyzed by ^1H NMR spectroscopy to determine the NMR yield. The crude mixtures were purified by flash column chromatography (Silica gel, Pentane 100% to Pentane/Et_2O 80:10).

10. Starting Material preparation

2-Ethylhexyl 3-((2-bromo-4-methoxyphenyl)thio)propanoate (S6a)

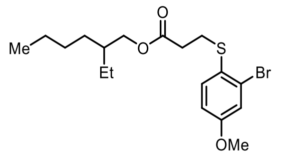

Following **TP1**, 2-bromo-1-iodo-4-methoxybenzene (3.0 g, 9.6 mmol, 1 eq.) gave the title compound after flash column chromatography (Pentane/EtOAc 9:1 to 7:3) as colorless viscous oil (2.6 g, 6.5 mmol, 68%). ^{1}H NMR ($CDCl_3$, 600 MHz) δ 7.39 (1H, d, *J* = 8.6 Hz), 7.17 (1H, d, *J* = 2.8 Hz), 6.82 (1H, dd, *J* = 8.7, 2.8 Hz), 4.04 – 3.96 (2H, m), 3.79 (3H, s), 3.10 (2H, t, *J* = 7.3 Hz), 2.58 (2H, t, *J* = 7.3 Hz), 1.60 – 1.50 (1H, m), 1.40– 1.18 (8H, m), 0.93 – 0.81 (6H, m); ^{13}C NMR ($CDCl_3$, 151 MHz) δ 172.0, 159.7, 134.5, 128.5, 126.4, 119.0, 114.3, 67.3, 55.8, 38.8, 34.4, 30.5, 30.1, 29.0, 23.9, 23.1, 14.2, 11.1. HRMS (ESI): found M^+ 402.0859, $C_{18}H_{27}BrO_3S$ requires 402.0859.

2-Ethylhexyl 3-((2-bromo-4-(trifluoromethyl)phenyl)thio)propanoate (S6b)

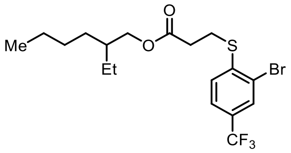

Following **TP1**, 2-bromo-1-iodo-4-methoxybenzene (3.4 g, 9.6 mmol, 1 eq.) gave the title compound after flash column chromatography (Pentane/EtOAc 9:1 to 7:3) as colorless viscous oil (4.2 g, 9.5 mmol, 98%).

^{1}H NMR ($CDCl_3$, 600 MHz) δ 7.78 (1H, s), 7.53 (1H, d, *J* = 8.3 Hz), 7.29 (1H, d, *J* = 8.5 Hz), 4.04 (2H, d, *J* = 5.5 Hz), 3.26 (2H, t, *J* = 7.4 Hz), 2.73 (2H, t, *J* = 7.5 Hz), 1.65 – 1.47 (1H, m), 1.42 – 1.21 (8H, m), 0.89 (6H, t, *J* = 7.5 Hz); ^{13}C NMR ($CDCl_3$, 151 MHz) δ 171.5, 143.1, 130.0 (q, *J* = 4.1 Hz), 128.5 (q, *J* = 33.2 Hz), 126.4, 124.7 (q, *J* = 3.9 Hz), 123.4 (q, *J* = 273.3 Hz), 122.5 (q, *J* = 17.3 Hz), 67.7, 38.9, 33.5, 30.5, 29.1, 27.6, 23.9, 23.1, 14.2, 11.1; ^{19}F NMR ($CDCl_3$, 564 MHz) δ -62.51. HRMS (ESI): found M^+ 440.0629, $C_{18}H_{24}BrF_3O_2S$ requires 440.0627.

2-Ethylhexyl 3-((2-bromophenyl)thio)propanoate (S6c)

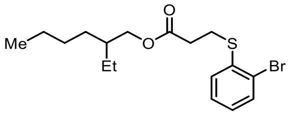

Following **TP1**, 1-bromo-2-iodobenzene (10 g, 35.4 mmol, 1 eq.) gave the title compound after flash column chromatography (Pentane/EtOAc 9:1 to 7:3) as colorless viscous oil (12.1 g, 32.4 mmol, 92%). ^{1}H NMR ($CDCl_3$, 600 MHz) δ 7.56 – 7.53 (1H, m), 7.31 – 7.23 (2H, m), 7.07 – 7.00 (1H, m), 4.05 – 3.97 (2H, m), 3.19 (2H, t, *J* = 7.5 Hz), 2.66 (2H, t, *J* = 7.5 Hz), 1.60 – 1.51 (1H, m), 1.34 (2H, p, *J* = 7.5 Hz), 1.31 – 1.22 (6H, m), 0.87 (6H, t, *J* = 7.4 Hz); ^{13}C NMR ($CDCl_3$, 151 MHz) δ 171.6, 136.8, 133.1, 128.7, 127.8, 127.0, 124.2, 67.3, 38.6, 33.8, 30.3, 28.8, 28.0, 23.7, 22.9, 14.0, 10.9. HRMS (ESI): found MNa^+ 395.0649, $C_{17}H_{25}BrO_2SNa$ requires 395.0656.

3',5-Dimethoxy-[1,1'-biphenyl]-2-thiol (2d)

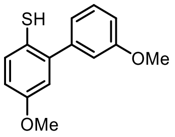

Following **TP2**, 2-ethylhexyl 3-((2-bromo-4-methoxyphenyl)thio)propanoate (1.3 g, 3.2 mmol, 1 eq.) and (3-methoxyphenyl)boronic acid (636 mg, 4.2 mmol, 1.3 eq.) gave the title compound after column chromatography (Pentane to Pentane/EtOAc 8:2) as a colorless viscous oil (660 mg, 2.7 mmol, 83% over two steps). ^{1}H NMR ($CDCl_3$, 600 MHz) δ 7.39 – 7.33 (1H, m), 7.30 (1H, d, *J* = 8.5 Hz), 6.98 (1H, dt, *J* = 7.6, 1.3 Hz), 6.96 – 6.91 (2H, m), 6.84 – 6.83 (1H, m), 6.82 – 6.80 (1H, m), 3.85 (3H, s), 3.80 (3H, s), 3.27 (1H, s); ^{13}C NMR ($CDCl_3$, 151 MHz) δ 159.6, 158.1, 142.5, 142.4, 131.4, 129.6, 121.6, 120.6, 116.0, 114.7, 114.2, 113.5, 55.6, 55.4. HRMS (ESI): found M^+ 246.0708, $C_{14}H_{14}O_2S$ requires 246.0709.

3'-Methoxy-5-(trifluoromethyl)-[1,1'-biphenyl]-2-thiol (2e)

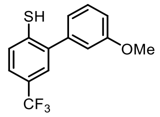

Following **TP2**, 2-ethylhexyl 3-((2-bromo-4-(trifluoromethyl)phenyl)thio)propanoate (2.1 g, 4.8 mmol, 1 eq.) and (3-methoxyphenyl)boronic acid (942 mg, 6.2 mmol, 1.3 eq.) gave the title compound after column chromatography (Pentane to Pentane/EtOAc 8:2) as a colorless viscous oil (260 mg, 1 mmol, 21% over two steps). ^{1}H NMR ($CDCl_3$, 600 MHz) δ 7.48 (1H, s), 7.44 (1H, s), 7.39 (1H, t, J = 7.9 Hz), 7.00 – 6.96 (2H, m), 6.93 (2H, t, J = 2.1 Hz), 3.86 (3H, s), 3.62 (1H, s); ^{13}C NMR ($CDCl_3$, 151 MHz) δ 159.9, 140.9, 140.4, 136.7, 130.0, 129.2, 127.7 (q, J = 32.7 Hz), 127.1 (q, J = 3.8 Hz), 124.6 (q, J = 3.7 Hz), 124.2 (q, J = 271.8 Hz), 121.4, 114.6, 114.1, 55.5; ^{19}F NMR ($CDCl_3$, 564 MHz) δ -62.4. HRMS (ESI): found M^+ 284.0476, $C_{14}H_{11}F_3OS$ requires 284.0477.

3',5,5'-Trimethoxy-[1,1'-biphenyl]-2-thiol (2f)

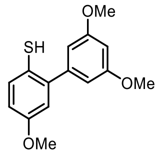

Following **TP2**, 2-ethylhexyl 3-((2-bromo-4-methoxyphenyl)thio)propanoate (1.3 g, 3.2 mmol, 1 eq.) and (3,5-dimethoxyphenyl)boronic acid (762 mg, 4.2 mmol, 1.3 eq.) gave the title compound after column chromatography (Pentane to Pentane/EtOAc 8:2) as a colorless viscous oil (160 mg, 0.6 mmol, 18% over two steps). ^{1}H NMR ($CDCl_3$, 600 MHz) δ 7.46 (1H, d, J = 8.6 Hz), 6.81 – 6.76 (2H, m), 6.44 (1H, t, J = 2.2 Hz), 6.41 (2H, d, J = 2.3 Hz), 3.80 (3H, s), 3.78 (6H, s); ^{13}C NMR ($CDCl_3$, 151 MHz) δ 160.4, 159.2, 144.7, 142.3, 132.9, 126.4, 115.4, 114.3, 107.9, 99.9, 55.6, 55.5. HRMS (ESI): found M^+ 276.0814, $C_{15}H_{16}O_3S$ requires 276.0815.

3',5'-Dimethoxy-5-(trifluoromethyl)-[1,1'-biphenyl]-2-thiol (2g)

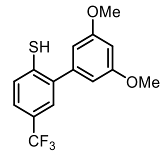

Following **TP2**, 2-ethylhexyl 3-((2-bromo-4-(trifluoromethyl)phenyl)thio)propanoate (2.1 g, 4.8 mmol, 1 eq.) and (3,5-dimethoxyphenyl)boronic acid (1.1 g, 6.2 mmol, 1.3 eq.) gave the title compound after column chromatography (Pentane to Pentane/EtOAc 8:2) as a colorless viscous oil (160 mg, 0.6 mmol, 18% over two steps). ^{1}H NMR ($CDCl_3$, 600 MHz) δ 7.47 (1H, s), 7.43 (2H, s), 6.51 (3H, s), 3.83 (6H, s), 3.67 (1H, s); ^{13}C NMR ($CDCl_3$, 151 MHz) δ 161.2, 141.4, 140.4, 136.7, 129.2, 127.7 (q, J = 32.6 Hz), 127.0 (q, J = 4.1 Hz), 124.7 (q, J = 3.8 Hz), 124.2 (q, J = 273.3 Hz), 107.1, 100.5, 55.6; ^{19}F NMR ($CDCl_3$, 564 MHz) δ -62.40. HRMS (ESI): found M^+ 314.0575, $C_{15}H_{13}F_3O_2S$ requires 314.0583.

3',5'-Dimethoxy-[1,1'-biphenyl]-2-thiol (2h)

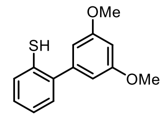

Following **TP2**, 2-ethylhexyl 3-((2-bromo-4-methoxyphenyl)thio)propanoate (12.1 g, 32.4 mmol, 1 eq) and (3,5-dimethoxyphenyl)boronic acid (7.73 g, 15.0 mmol, 1.3 eq) gave the title compound after column chromatography (Pentane to Pentane/EtOAc 8:2) as a colorless viscous oil (3.5 g, 14.1 mmol, 70%). ^{1}H NMR ($CDCl_3$, 600 MHz) δ 7.34 (1H, d, J = 6.6 Hz), 7.26 – 7.12 (3H, m), 6.53 (2H, s), 6.49 (1H, s), 3.82 (6H, s), 3.49 (1H, s); ^{13}C NMR ($CDCl_3$, 151 MHz) δ 160.9, 142.9, 140.4, 131.0, 130.3, 129.4, 128.1, 125.5, 107.3, 100.0, 55.5. HRMS (ESI): found MNa^+ 269.0611, $C_{14}H_{14}O_2SNa$ requires 269.0612.

5-(tert-butyl)-2-((3',5'-dimethoxy-[1,1'-biphenyl]-2-yl)thio)-3H-azepine (S1ah)

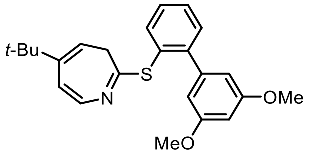

An oven-dried tube equipped with a stirring bar was charged with 1-azido-4-(*tert*-butyl)benzene (42mg, 2.4 mmol, 2.4 eq.), 3',5'-dimethoxy-[1,1'-biphenyl]-2-thiol (24.6 mg, 0.1 mmol, 1.0 eq.) and DMAP (12.2 mg, 1.0 eq.). The tube was then capped under air with a Supelco aluminium crimp seal with septum (PTFE/butyl), evacuated and backfilled with argon three times. Anhydrous 1,4-dioxane (2 mL, 0.05 M) was added via syringe. The mixture was then purged with Argon for 5 min. and stirred (600 rpm) under irradiation with purple LEDs (Kessil PR160L-390 nm) for 16 h at room temperature (fan ventilation). The crude was dried under vacuum and then purified *via* Flash column cromatography (Silica gel, Pentane/Et_2O 80:20 to 60:40) to afford the title compound as a colorless oil (27.5 mg, 70%).

^{1}H NMR ($CDCl_3$, 600 MHz) δ 7.53 (1H, d, *J*=7.0 Hz), 7.42 – 7.37 (2H, m), 7.38 – 7.32 (1H, m), 7.11 (1H, d, *J*=8.5 Hz), 6.54 (2H, d, *J*=2.3 Hz), 6.46 (1H, t, *J*=2.3 Hz), 6.15 (1H, dd, *J*=8.6, 1.2 Hz), 4.99 (1H, t, *J*=6.9 Hz), 3.78 (6H, s), 2.56 (2H, s), 1.10 (9H, s); ^{13}C NMR ($CDCl_3$, 151 MHz) δ 160.2, 151.6, 149.3, 145.8, 142.7, 140.7, 136.5, 130.7, 129.3, 128.6, 128.1, 116.1, 110.7, 107.7, 99.7, 55.5, 37.1, 34.7, 30.2. HRMS (ESI): found MH^+ 394.1834, $C_{24}H_{28}NO_2S$ requires 394.1835.

***N*-(5-(*tert*-butyl)-2-((3',5'-dimethoxy-[1,1'-biphenyl]-2-yl)thio)phenyl)-2,2,2-trifluoroacetamide (3ah)**

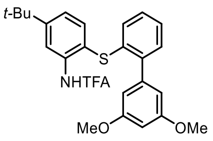

Following **GP1**, 1-azido-4-(tert-butyl)benzene (42 mg, 0.24 mmol, 2.4 eq.) gave the title compound (65%) as an oil. ^{1}H NMR ($CDCl_3$, 600 MHz) δ 8.82 (1H, s), 8.42 (1H, s), 7.47 (1H, d, *J* = 8.1 Hz), 7.29 (1H, t, *J* = 7.8 Hz), 7.26 (2H, t, *J* = 7.7 Hz), 7.22 (1H, t, *J* = 7.6 Hz), 6.89 (1H, d, *J* = 9.0 Hz), 6.53 (1H, s), 6.51 (2H, s), 3.84 (6H, s), 1.38 (9H, s); ^{13}C NMR ($CDCl_3$, 151 MHz) δ 160.7, 154.9, 154.6 (q, *J* = 37.4 Hz), 142.0, 141.8, 136.8, 136.4, 133.7, 130.5, 128.5, 127.7, 126.7,

123.7, 119.3, 118.5, 115.7 (q, J = 289.0 Hz), 107.3, 100.0, 55.5, 35.3, 31.2; ^{19}F NMR ($CDCl_3$, 564 MHz) δ -76.08. HRMS (ESI): found MNa^+ 512.1469, $C_{26}H_{26}F_3NO_3SNa$ requires 512.1478.

5-(4-(*tert*-butyl)-2-(2,2,2-trifluoroacetamido)phenyl)-2,4-dimethoxy-5H-dibenzo[b,d]thiophen-5-ium trifluoromethanesulfonate (4ah)

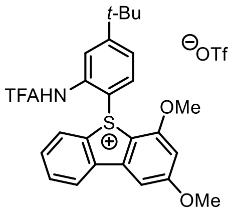

Following **GP2a**, N-(5-(tert-butyl)-2-((3',5'-dimethoxy-[1,1'-biphenyl]-2-yl)thio)phenyl)-2,2,2-trifluoroacetamide (50 mg, 0.1 mmol, 1.0 eq.) gave the title compound as a off-white solid (44.0 mg, 90%). ^{1}H NMR ($CDCl_3$, 600 MHz) δ 11.53 (1H, s), 8.52 (1H, d, J=8.1 Hz), 8.15 (1H, d, J=7.8 Hz), 7.88 (1H, t, J=7.6 Hz), 7.73 (1H, d, J=2.0 Hz), 7.68 (1H, t, J=7.8 Hz), 7.24 (1H, dd, J=8.8, 2.1 Hz), 7.21 (1H, d, J=2.0 Hz), 6.53 (1H, d, J=1.8 Hz), 6.45 (1H, d, J=8.7 Hz), 4.00 (3H, s), 3.87 (3H, s), 1.29 (9H, s); ^{13}C NMR ($CDCl_3$, 151 MHz) δ 167.8, 160.8, 158.8, 158.4 (q, J=39.2 Hz), 141.4, 139.8, 138.1, 134.4, 132.3, 132.1, 129.8, 127.8, 127.0, 126.8, 124.3, 120.7 (q, J=319.9 Hz), 118.6, 116.1 (q, J=287.2 Hz), 108.6, 101.2, 100.3, 57.3, 56.7, 35.8, 30.8; ^{19}F NMR ($CDCl_3$, 564 MHz) δ -75.00, -78.36. HRMS (ESI): found M^+ 488.1489, $C_{26}H_{25}F_3NO_3S^+$ requires 488.1502.

***N*-(3-(*tert*-butyl)phenyl)-2,2,2-trifluoroacetamide (5a)**

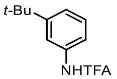

Following **GP2b**, N-(5-(*tert*-butyl)-2-((3',5'-dimethoxy-[1,1'-biphenyl]-2-yl)thio)phenyl)-2,2,2-trifluoroacetamide (47.8 mg, 0.24 mmol, 2.4 eq.) gave the title compound (95%) as an oil. ^{1}H NMR ($CDCl_3$, 600 MHz) δ 7.96 (1H, s), 7.52 (1H, t, J = 2.0 Hz), 7.45 (1H, d, J = 7.9 Hz), 7.32 (1H, t, J = 7.9 Hz), 7.28 (1H, s), 1.32 (9H, s); ^{13}C NMR ($CDCl_3$, 151 MHz) δ 154.8 (q, J = 37.1 Hz), 153.0, 135.0, 129.2, 123.6, 117.9, 117.8, 115.8 (q, J = 288.7 Hz), 35.0, 31.3; ^{19}F NMR ($CDCl_3$, 564 MHz) δ -75.77. HRMS (ESI): found M^+ 245.1022, $C_{12}H_{14}F_3NO$ requires 245.1022.

***N*-(5-(*tert*-butyl)-2-(phenylthio)phenyl)-2,2,2-trifluoroacetamide (3aa)**

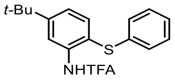

Following **GP1**, 1-azido-4-(*tert*-butyl)benzene (42 mg, 0.24 mmol, 2.4 eq.) gave the title compound (65%) as an oil. ^{1}H NMR ($CDCl_3$, 600 MHz) δ 9.09 (1H, s), 8.49 (1H, d, *J* = 2.1 Hz), 7.60 (1H, d, *J* = 8.2 Hz), 7.35 – 7.23 (3H, m), 7.22 – 7.16 (1H, m), 7.12 (2H, d, *J* = 7.0 Hz), 1.36 (9H, d, *J* = 1.8 Hz); ^{13}C NMR (151 MHz, CD_3CN) δ 155.3, 154.7 (q, *J* = 37.0 Hz), 137.2, 136.4, 135.0, 129.6, 127.9, 127.0, 123.7, 118.8, 118.6, 115.7 (q, *J* = 288.9 Hz), 35.4, 31.3; ^{19}F NMR (376 MHz, CD_3CN) δ –76.65; HRMS (ESI): found MNa^+ 376.0951, $C_{18}H_{18}F_3NOSNa$ requires 376.0953. Data in accordance with literature[1].

***N*-(5-(*tert*-butyl)-2-((4-(trifluoromethyl)phenyl)thio)phenyl)-2,2,2-trifluoroacetamide (3ab)**

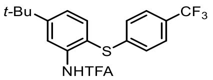

Following **GP1**, 1-azido-4-(*tert*-butyl)benzene (42 mg, 0.24 mmol, 2.4 eq.) gave the title compound (48%) as an oil. ^{1}H NMR ($CDCl_3$, 600 MHz) δ 8.89 (1H, s), 8.44 (1H, d, *J* = 2.1 Hz), 7.52 (1H, d, *J* = 8.2 Hz), 7.41 (2H, d, *J* = 8.1 Hz), 7.25 (1H, dd, *J* = 8.2, 2.1 Hz), 7.05 (2H, d, *J* = 8.0 Hz), 1.30 (9H, s); ^{13}C NMR ($CDCl_3$, 151 MHz) δ 156.2, 154.6 (q, *J* = 37.7 Hz), 140.4, 137.3, 136.8, 128.7 (q, *J* = 32.8 Hz), 126.7, 126.2 (q, *J* = 3.8 Hz), 124.0, 123.8 (q, *J* = 271.8 Hz), 118.8, 116.7, 115.5 (d, *J* = 288.8 Hz), 35.4, 31.1; ^{19}F NMR ($CDCl_3$, 564 MHz) δ -62.7, -76.7. HRMS (ESI): found M^+ 421.0930, $C_{19}H_{17}F_6NOS$ requires 421.0935.

***N*-(5-(*tert*-butyl)-2-((4-methoxyphenyl)thio)phenyl)-2,2,2-trifluoroacetamide (3ac)**

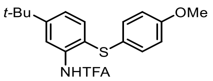

Following **GP1**, 1-azido-4-(*tert*-butyl)benzene (42 mg, 0.24 mmol, 2.4 eq.) gave the title compound (67%) as an oil. ^{1}H NMR ($CDCl_3$, 600 MHz) δ 9.14 (1H, s), 8.42 (1H, d, *J* = 2.1 Hz), 7.54 (1H, d, *J* = 8.2 Hz), 7.23 (1H, dd, *J* = 8.2, 2.1 Hz), 7.17 (2H, d, *J* = 8.8 Hz), 6.82 (2H, d, *J* = 8.8 Hz), 3.77 (3H, s), 1.34 (9H, s); ^{13}C NMR ($CDCl_3$, 151 MHz) δ 159.3, 154.5 (q, *J* = 39.2 Hz),154.4, 136.3, 135.3, 131.2, 124.9, 123.5, 120.9, 118.4, 115.6 (q, *J* = 294.4 Hz), 115.1, 55.4,

35.1, 31.1; ^{19}F NMR ($CDCl_3$, 564 MHz) δ -76.01. HRMS (ESI): found M^+ 383.1161, $C_{19}H_{20}F_3NO_2S$ requires 383.1167. Data in accordance with literature[1].

_N_-(5-(_Tert_-butyl)-2-((3',5-dimethoxy-[1,1'-biphenyl]-2-yl)thio)phenyl)-2,2,2-trifluoroacetamide (3ad)

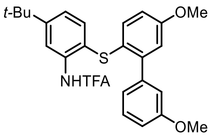

Following **GP1**, 1-azido-4-(_tert_-butyl)benzene (42 mg, 0.24 mmol, 2.4 eq.) gave the title compound (56%) as an oil. ^{1}H NMR ($CDCl_3$, 600 MHz) δ 8.68 (1H, s), 8.28 (1H, d, J = 2.1 Hz), 7.30 (1H, t, J = 7.9 Hz), 7.27 (1H, s), 7.14 (1H, dd, J = 8.2, 2.1 Hz), 7.10 (1H, d, J = 8.6 Hz), 6.91 (1H, dd, J = 8.3, 3.6 Hz), 6.87 (1H, d, J = 8.9 Hz), 6.85 – 6.78 (2H, m), 6.75 – 6.70 (1H, m), 3.79 (3H, s), 3.78 (3H, s), 1.32 (9H, s); ^{13}C NMR ($CDCl_3$, 151 MHz) δ 159.3, 159.1, 154.4 (q, J = 37.4 Hz), 153.7, 144.5, 141.5, 135.9, 135.0, 131.9, 129.2, 123.9, 123.4, 121.3, 121.3, 118.1, 116.4, 115.5 (q, J = 286.9 Hz), 114.6, 114.1, 113.2, 55.4, 55.2, 35.0, 31.1; ^{19}F NMR ($CDCl_3$, 564 MHz) δ -75.91. HRMS (ESI): found MNa^+ 512.1484, $C_{26}H_{26}F_3NO_3SNa$ requires 512.1483.

_N_-(5-(_tert_-butyl)-2-((3'-methoxy-5-(trifluoromethyl)-[1,1'-biphenyl]-2-yl)thio)phenyl)-2,2,2-trifluoroacetamide (3ae)

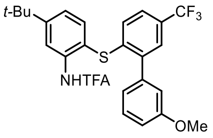

Following **GP1**, 1-azido-4-(_tert_-butyl)benzene (42 mg, 0.24 mmol, 2.4 eq.) gave the title compound (49%) as an oil. ^{1}H NMR ($CDCl_3$, 600 MHz) δ 8.76 (1H, s), 8.46 (1H, s), 7.52 (1H, s), 7.50 (1H, d, J = 8.2 Hz), 7.41 (2H, t, J = 8.2 Hz), 7.30 (1H, dd, J = 8.2, 1.1 Hz), 7.00 (2H, t, J = 8.0 Hz), 6.92 (1H, s), 6.83 (1H, d, J = 8.4 Hz), 3.87 (3H, s), 1.37 (9H, s); ^{13}C NMR ($CDCl_3$, 151 MHz) δ 159.8, 156.0. 154.6 (q, J = 37.6 Hz), 141.2, 140.0, 139.3, 137.2, 136.9, 129.9, 128.6 (q, J = 32.8 Hz), 127.2 (q, J = 3.5 Hz), 126.8, 125.1 (q, J = 3.3 Hz), 124.1, 123.8 (q, J = 271.8 Hz), 121.4, 118.9, 117.5, 115.4 (q, J = 288.4 Hz), 114.7, 114.2, 55.4, 35.5, 31.1; ^{19}F NMR ($CDCl_3$, 564 MHz) δ -62.55, -76.11. HRMS (ESI): found MNa^+ 550.1245, $C_{26}H_{23}F_6NO_2SNa$ requires 550.1246.

***N*-(5-(*Tert*-butyl)-2-((3',5,5'-trimethoxy-[1,1'-biphenyl]-2-yl)thio)phenyl)-2,2,2-trifluoroacetamide (3af)**

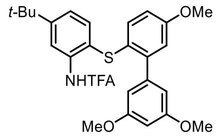

Following **GP1**, 1-azido-4-(*tert*-butyl)benzene (42 mg, 0.24 mmol, 2.4 eq.) gave the title compound (52%) as an oil. ^{1}H NMR ($CDCl_3$, 600 MHz) δ 8.70 (1H, s), 8.28 (1H, d, J = 2.1 Hz), 7.28 (1H, d, J = 8.2 Hz), 7.14 (1H, dd, J = 8.2, 2.1 Hz), 7.08 (1H, d, J = 8.6 Hz), 6.83 (1H, d, J = 2.8 Hz), 6.80 (1H, dd, J = 8.6, 2.9 Hz), 6.46 (1H, t, J = 2.3 Hz), 6.36 (2H, d, J = 2.3 Hz), 3.79 (3H, s), 3.77 (6H, s), 1.31 (9H, s); ^{13}C NMR ($CDCl_3$, 151 MHz) δ 160.6, 159.2, 154.6 (q, J = 37.3 Hz), 153.8, 144.6, 142.2, 136.1, 135.2, 131.9, 123.9, 123.5, 121.4, 118.3, 116.4, 115.5 (q, J = 289.9 Hz), 114.3, 107.2, 99.8, 55.6, 55.4, 31.2, 29.9. ^{19}F NMR ($CDCl_3$, 564 MHz) δ -75.99. HRMS (ESI): found MNa^+ 542.1580, $C_{27}H_{28}F_3NO_4SNa$ requires 542.1583.

***N*-(5-(*tert*-butyl)-2-((3',5'-dimethoxy-5-(trifluoromethyl)-[1,1'-biphenyl]-2-yl)thio)phenyl)-2,2,2-trifluoroacetamide (3ag)**

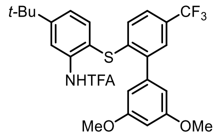

Following **GP1**, 1-azido-4-(*tert*-butyl)benzene (42 mg, 0.24 mmol, 2.4 eq.) gave the title compound (43%) as an oil. ^{1}H NMR ($CDCl_3$, 600 MHz) δ 8.77 (1H, s), 8.45 (1H, d, J = 2.1 Hz), 7.52 (1H, s), 7.50 (1H, d, J = 8.1 Hz), 7.41 (1H, dd, J = 8.5, 2.1 Hz), 7.29 (1H, dd, J = 8.2, 2.1 Hz), 6.82 (1H, d, J = 8.4 Hz), 6.55 (1H, t, J = 2.3 Hz), 6.51 (2H, d, J = 2.3 Hz), 3.84 (6H, s), 1.37 (9H, s); ^{13}C NMR ($CDCl_3$, 151 MHz) δ 161.0, 156.0, 154.6 (q, J = 37.4 Hz), 141.4, 140.5, 139.2, 137.1, 136.9, 128.6 (q, J = 33.0 Hz), 127.0 (q, J = 3.4 Hz), 126.8, 125.2 (q, J = 3.4 Hz), 124.1, 123.9 (q, J = 271.8 Hz), 118.9, 117.5, 115.5 (q, J = 289.1 Hz), 107.2, 100.6, 55.6, 35.5, 31.2; ^{19}F NMR ($CDCl_3$, 564 MHz) δ -62.55, -76.11. HRMS (ESI): found MNa^+ 580.1351, $C_{27}H_{25}F_6NO_3SNa$ requires 580.1352.

1-Azido-4-(*tert*-butyl)benzene (1a)

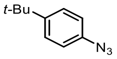

Following **AP1**, 4-(*tert*-butyl)aniline (2.0 g, 13.4 mmol, 1 eq.) gave the title compound (>98%) as an oil. ^{1}H NMR ($CDCl_3$, 600 MHz) δ 7.38 (2 H, d, *J* = 8.6 Hz), 6.97 (2 H, d, *J* = 8.7 Hz), 1.31 (9H, s); ^{13}C NMR ($CDCl_3$, 151 MHz) δ 148.2, 137.2, 126.8, 118.8, 34.6, 31.5. HRMS (ESI): found M^+ 175.1104, $C_{10}H_{13}N_3$ requires 175.1104. Data in accordance with literature[2].

1-Azido-4-methylbenzene (1b)

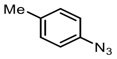

Following **AP1**, p-toluidine (2.0 g, 18.7mmol, 1 eq.) gave the title compound (>98%) as an oil. ^{1}H NMR ($CDCl_3$, 600 MHz) δ 7.21 (2H, d, *J* = 5.3 Hz), 6.90 (2H, d, *J* = 8.1 Hz), 2.35 (3H, s); ^{13}C NMR ($CDCl_3$, 151 MHz) δ 137.2, 134.6, 130.3, 118.8, 20.8. HRMS (ESI): found M^+ 133.0639, $C_7H_7N_3$ requires 133.0640. Data in accordance with literature[3].

1-Azido-4-ethylbenzene (1c)

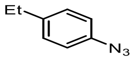

Following **AP1**, 4-ethylaniline (2.0 g, 16.5 mmol, 1 eq.) gave the title compound (>98%) as an oil. ^{1}H NMR ($CDCl_3$, 600 MHz) δ 7.22 (2H, d, *J* = 8.4 Hz), 7.04 (2H, d, *J* = 8.5 Hz), 2.61 (2H, q, *J* = 7.6 Hz), 1.24 (3H, t, *J* = 7.6 Hz); ^{13}C NMR ($CDCl_3$, 151 MHz) δ 141.1, 137.3, 129.2, 118.9, 28.3, 15.6. HRMS (ESI): found M^+ 147.0792, $C_8H_9N_3$ requires 147.0791. Data in accordance with literature[4].

1-Azido-4-isopropylbenzene (1d)

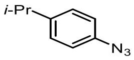

Following **AP1**, 4-isopropylaniline (2.0 g, 14.8 mmol, 1 eq.) gave the title compound (>98%) as an oil. ^{1}H NMR ($CDCl_3$, 600 MHz) δ 7.21 (2H, d, *J* = 8.4 Hz), 6.96 (2H, d, *J* = 8.5 Hz), 2.90 (1H, sp, *J* = 6.9 Hz), 1.24 (6H, d, *J* = 7.0 Hz); ^{13}C NMR ($CDCl_3$, 151 MHz) δ 145.7, 137.4, 127.8, 118.9,

33.6, 24.0. HRMS (ESI): found $[MNH_2]^+$ (mass of the aniline) 134.0964, $C_9H_{13}N$ requires 135.1048. Data in accordance with literature[5].

1-Azido-4-benzylbenzene (1e)

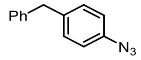

Following **AP1**, 4-benzylaniline (2.0 g, 10.9 mmol, 1 eq.) gave the title compound (>98%) as an oil. ^{1}H NMR ($CDCl_3$, 600 MHz) δ 7.30 (2H, t, J = 7.6 Hz), 7.22 (1H, t, J = 7.4 Hz), 7.18 (4H, d, J = 8.0 Hz), 6.96 (2H, d, J = 8.3 Hz), 3.97 (2H, s); ^{13}C NMR ($CDCl_3$, 151 MHz) δ 140.8, 138.0, 137.9, 130.3, 128.8, 128.6, 126.2, 119.1, 41.3. HRMS (ESI): found $[MNH_2]^+$ (mass of the aniline) 183.1041, $C_{13}H_{13}N$ requires 183.1043. Data in accordance with literature[6].

1-Azido-4-(trifluoromethyl)benzene (1f)

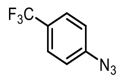

Following **AP1**, 4-(trifluoromethyl)aniline (2.0 g, 12.4 mmol, 1 eq.) gave the title compound (>98%) as an oil. ^{1}H NMR ($CDCl_3$, 600 MHz) δ 7.61 (2H, d, J = 8.4 Hz), 7.11 (2H, d, J = 8.3 Hz); ^{13}C NMR ($CDCl_3$, 151 MHz) δ 143.7, 127.0 (q, J = 31.7 Hz), 127.0 (q, J = 3.7 Hz), 123.9 (d, J = 271.9 Hz), 119.2. HRMS (ESI): found M^+ 187.0355, $C_7H_4F_3N_3$ requires 187.0360. Data in accordance with literature[2].

1-(4-Azidophenyl)cyclopentane-1-carbonitrile (1g)

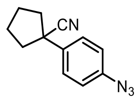

Following **AP1**, 1-(4-aminophenyl)cyclopentane-1-carbonitrile (1.0 g, 5.4 mmol, 1 eq.) gave the title compound (>98%) as an oil. ^{1}H NMR ($CDCl_3$, 600 MHz) δ 7.43 (2H, d, J = 8.6 Hz), 7.03 (2H, d, J = 8.7 Hz), 2.53 – 2.40 (2H, m), 2.11 – 2.00 (4H, m), 1.98 – 1.86 (2H, m); ^{13}C NMR ($CDCl_3$, 151 MHz) δ 139.7, 136.5, 127.5, 124.1, 119.4, 47.3, 40.4, 24.1. HRMS (ESI): found MNa^+ 235.0956, $C_{12}H_{12}N_4Na$ requires 235.0954. Data in accordance with literature[7].

1-Azido-4-(methoxymethyl)benzene (1h)

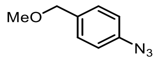

Following **AP1**, 4-(methoxymethyl)aniline (2.0 g, 14.6 mmol, 1 eq.) gave the title compound (>98%) as an oil. ^{1}H NMR ($CDCl_3$, 600 MHz) δ 7.32 (2H, d, *J* = 8.5 Hz), 7.01 (2H, d, *J* = 8.4 Hz), 4.42 (2H, s), 3.38 (3H, s); ^{13}C NMR ($CDCl_3$, 151 MHz) δ 139.5, 135.1, 129.4, 119.1, 74.2, 58.2. HRMS (ESI): found MNa^+ 186.0638, $C_8H_9N_3ONa$ requires 186.0638.

1-Azido-4-(2-(benzyloxy)-1,1,1,3,3,3-hexafluoropropan-2-yl)benzene (1i)

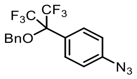

Prepared according to a known procedure[7], 2-(4-azidophenyl)-1,1,1,3,3,3-hexafluoropropan-2-ol (1.0 g, 3.5 mmol, 1 eq.) in DMF (35 mL) was added to a flame dried 100 mL round bottom flask. The flask was purged with Ar and cooled to 0°C. A 60% w/w dispersion in paraffin of NaH (280 mg, 7 mmol, 2 eq.) was added portionwise, followed by BnBr (831 µl, 7 mmol, 2 eq.). The reaction was allowed to warm up to room temperature over the course of 16 h, then quenched with distilled H_2O and extracted with EtOAc (3x). The organics were washed with brine, dried over $MgSO_4$ and concentrated. The crude mixture was purified by flash column chromatography (Silica gel, Pentane 100% to Pentane/EtOAc 80/20) to give the title compound (58%) as an oil. ^{1}H NMR ($CDCl_3$, 600 MHz) δ 7.61 (2H, d, *J* = 8.5 Hz), 7.42 – 7.32 (5H, m), 7.14 – 7.10 (2H, m), 4.63 (2H, s); ^{13}C NMR ($CDCl_3$, 151 MHz) δ 142.6, 136.2, 129.9, 128.8, 128.4, 127.3, 124.6, 122.5 (q, *J* = 291.2 Hz), 119.5, 68.2, 64.8; ^{19}F NMR ($CDCl_3$, 564 MHz) δ -70.83. HRMS (ESI): found MNa^+ 398.0703, $C_{16}H_{11}F_6N_3ONa$ requires 398.0699. Data in accordance with literature[7].

(4-Azidophenyl)diphenylmethanol (1j)

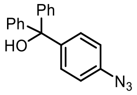

Following **AP2**, (4-bromophenyl)diphenylmethanol (2 g, 5.9 mmol, 1 eq.), prepared according to a known procedure[7], gave the title compound (78%) as an oil. ^{1}H NMR ($CDCl_3$, 600 MHz) δ 7.34 – 7.24 (12H, m), 6.97 (2H, d, *J* = 8.6 Hz), 2.78 (1H, s); ^{13}C NMR ($CDCl_3$, 151 MHz) δ 146.7,

143.8, 139.1, 129.6, 128.2, 127.9, 127.6, 118.6, 81.9. HRMS (ESI): found MNa^+ 324.1109, $C_{19}H_{15}N_3ONa$ requires 324.1107.

2-(4-Azidophenyl)-4,4,5,5-tetramethyl-1,3,2-dioxaborolane (1k)

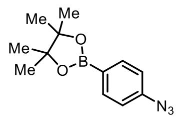

Following **AP1**, 4-(4,4,5,5-tetramethyl-1,3,2-dioxaborolan-2-yl)aniline (2 g, 9.13 mmol, 1 eq.) gave the title compound (>98%) as a solid. ^1H NMR ($CDCl_3$, 600 MHz) δ 7.79 (2H, d, J = 8.4 Hz), 7.02 (2H, d, J = 8.4 Hz), 1.34 (12H, s); ^{13}C NMR ($CDCl_3$, 151 MHz) δ 142.8, 136.4, 118.3, 83.9, 24.8. HRMS (ESI): found $[M-N_2]^+$ 217.1269, $C_{12}H_{16}BNO_2$ requires 217.1269. Data in accordance with literature[8].

((1-(4-Azidophenyl)cyclohexyl)oxy)trimethylsilane (1l)

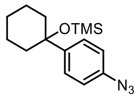

Following **AP2**, 4-(1-((trimethylsilyl)oxy)cyclohexyl)aniline (2.0 g, 11.5 mmol, 1 eq.), prepared according to a known procedure[7], gave the title compound (91%) as an oil. ^1H NMR ($CDCl_3$, 600 MHz) δ 7.44 (2H, d, J = 8.7 Hz), 6.98 (2H, d, J = 8.7 Hz), 1.98 – 1.92 (2H, m), 1.80 – 1.67 (4H, m), 1.67 – 1.62 (1H, m), 1.57 – 1.49 (2H, m), 1.30 – 1.18 (1H, m), -0.11 (9H, s); ^{13}C NMR ($CDCl_3$, 151 MHz) δ 146.0, 138.5, 127.4, 118.6, 75.3, 39.3, 25.9, 22.6, 2.3. HRMS (ESI): found MNa^+ 312.1504, $C_{15}H_{23}N_3OSiNa$ requires 312.1503. Data in accordance with literature[7].

4-Azido-1,1'-biphenyl (1m)

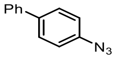

Following **AP1**, [1,1'-biphenyl]-4-amine (2 g, 11.8 mmol, 1 eq.) gave the title compound (>98%) as an oil. ^1H NMR ($CDCl_3$, 600 MHz) δ 7.61 – 7.53 (4H, m), 7.45 (2H, t, J = 7.6 Hz), 7.35 (1H, t, J = 6.8 Hz), 7.11 (2H, d, J = 8.0 Hz); ^{13}C NMR ($CDCl_3$, 151 MHz) δ 140.1, 139.1, 138.0, 128.9, 128.4, 127.4, 126.9, 119.4. HRMS (ESI): found $[M-N_2]^+$ 167.0729, $C_{12}H_9N$ requires 167.0730 Data in accordance with literature[2].

4-Azido-4'-methoxy-1,1'-biphenyl (1n)

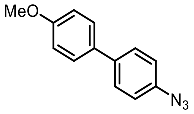

Following **AP1**, 4'-methoxy-[1,1'-biphenyl]-4-amine (2.0 g, 10.0 mmol, 1 eq.) gave the title compound (>98%) as an oil. ^{1}H NMR ($CDCl_3$, 600 MHz) δ 7.55 (2H, d, *J* = 8.5 Hz), 7.50 (2H, d, *J* = 8.7 Hz), 7.10 (2H, d, *J* = 8.5 Hz), 7.01 (2H, d, *J* = 8.7 Hz), 3.94 (3H, s); ^{13}C NMR ($CDCl_3$, 151 MHz) δ 159.2, 138.5, 137.7, 132.7, 128.0, 127.9, 119.4, 114.3, 55.4. HRMS (ESI): found $[MNH_2]^+$ (mass of the aniline) 199.0990, $C_{13}H_{13}NO$ requires 199.0992. Data in accordance with literature[9].

4-Azido-4'-iodo-1,1'-biphenyl (1o)

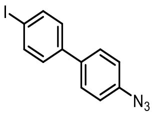

Following **AP1**, 4'-iodo-[1,1'-biphenyl]-4-amine (2.0 mg, 6.8 mmol, 1 eq.) gave the title compound (>98%) as a solid. ^{1}H NMR ($CDCl_3$, 600 MHz) δ 7.76 (2H, d, *J* = 8.5 Hz), 7.54 (2H, d, *J* = 8.6 Hz), 7.29 (2H, d, *J* = 8.5 Hz), 7.10 (2H, d, *J* = 8.5 Hz); ^{13}C NMR ($CDCl_3$, 151 MHz) δ 139.8, 139.8, 138.1, 136.9, 128.8, 128.4, 119.7, 93.2. HRMS (ESI): found $[MNH_2]^+$ (mass of the aniline) 294.9850, $C_{12}H_{10}IN$ requires 294.9852.

4'-Azido-3-fluoro-1,1'-biphenyl (1p)

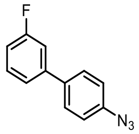

Following **AP1**, 3'-fluoro-[1,1'-biphenyl]-4-amine (2.0 g, 10.7 mmol, 1 eq.) gave the title compound (>98%) as an oil. ^{1}H NMR ($CDCl_3$, 600 MHz) δ 7.56 (2H, d, *J* = 8.6 Hz), 7.43 – 7.36 (1H, m), 7.35 – 7.31 (1H, m), 7.29 – 7.21 (1H, m), 7.11 (2H, d, *J* = 8.5 Hz), 7.04 (1H, td, *J* = 8.4, 1.5 Hz); ^{13}C NMR ($CDCl_3$, 151 MHz) δ 163.2 (d, *J* = 245.8 Hz), 142.4 (d, *J* = 7.5 Hz), 139.8, 136.6, 136.6, 130.3 (d, *J* = 8.5 Hz), 128.4, 122.4 (d, *J* = 2.6 Hz), 114.1 (d, *J* = 21.0 Hz), 113.7 (d, *J* = 22.1 Hz); ^{19}F NMR ($CDCl_3$, 564 MHz) δ -112.98. HRMS (ESI): found $[MNH_2]^+$ (mass of the aniline) 185.0635, $C_{12}H_{10}FN$ requires 185.0635. Data in accordance with literature[10].

1-Azido-3-methoxybenzene (1q)

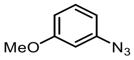

Following **AP1**, 3-methoxyaniline (2.0 g, 16.2 mmol, 1 eq.) gave the title compound (>98%) as an oil. ^{1}H NMR ($CDCl_3$, 600 MHz) δ 7.25 (1H, t, *J* = 8.1 Hz), 6.69 (1H, dd, *J* = 8.3, 2.4 Hz), 6.65 (1H, dd, *J* = 8.0, 2.1 Hz), 6.55 (1H, t, *J* = 2.3 Hz), 3.80 (3H, s); ^{13}C NMR ($CDCl_3$, 151 MHz) δ 160.8, 141.3, 130.4, 111.3, 110.7, 104.9, 55.4. HRMS (ESI): found M$^+$ 149.0585, $C_7H_7N_3O$ requires 149.0584. Data in accordance with literature[11].

1-Azido-3-(*tert*-butyl)benzene (1r)

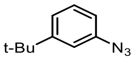

Following **AP1**, 3-(*tert*-butyl)aniline (2.0 g, 13.4 mmol, 1 eq.) gave the title compound (>98%) as an oil. ^{1}H NMR ($CDCl_3$, 600 MHz) δ 7.29 (1H, t, *J* = 7.9 Hz), 7.17 (1H, d, *J* = 10.7 Hz), 7.02 (1H, t, *J* = 2.1 Hz), 6.87 (1H, d, *J* = 11.2 Hz), 1.32 (9H, s); ^{13}C NMR ($CDCl_3$, 151 MHz) δ 153.5, 139.8, 129.5, 122.2, 116.4, 116.1, 35.0, 31.3. HRMS (ESI): found M$^+$ 175.1104, $C_{10}H_{13}N_3$ requires 175.1104. Data in accordance with literature[12].

1-Azido-3,5-dimethylbenzene (1s)

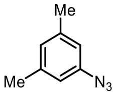

Following **AP1**, 3,5-dimethylaniline (2.0 g, 16.5 mmol, 1 eq.) gave the title compound (>98%) as an oil. ^{1}H NMR ($CDCl_3$, 600 MHz) δ 6.78 (1H, s), 6.65 (2H, s), 2.30 (6H, s); ^{13}C NMR ($CDCl_3$, 151 MHz) δ 140.0, 140.0, 127.1, 117.1, 21.6. HRMS (ESI): found M$^+$ 147.0791, $C_8H_9N_3$ requires 147.0790. Data in accordance with literature[11].

1-Azido-3,5-dimethoxybenzene (1t)

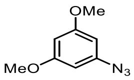

In a 250 mL round bottom flask under air, 3,5-dimethoxyaniline (2.0 g, 13.1 mmol, 1 eq.) was dissolved in 37% HCl in H_2O (3.5 mL) and distilled H_2O (80 mL) and cooled at 0°C. Sodium nitrite (991 mg, 14.4 mmol, 1.1 eq.) was dissolved in distilled H_2O (10 mL) and added to the

solution in 10 min. using a dropping funnel. After addition of K_2CO_3 (2.7 g, 19.6 mmol, 1.5 eq.) a solution of NaN_3 (1.1 g, 17 mmol. 1.3 eq.) in distilled H_2 was added to the mixture in 15 min. The solution was stirred for an hour at 0°C and etracted with Et_2O (3x). The combined organic phases were dried over $MgSO_4$ and the solvent evaporated. The crude mixture was then purified by column chromatography (Silica gel, Pentane/EtOAc 100% to 80/20) to give the title compound (>98%) as solid. ^{1}H NMR ($CDCl_3$, 600 MHz) δ 6.25 (1H, t, J = 2.2 Hz), 6.19 (2H, d, J = 2.2 Hz), 3.78 (6H, s); ^{13}C NMR ($CDCl_3$, 151 MHz) δ 161.8, 142.1, 97.7, 97.4, 55.6. HRMS (ESI): found M^+ 179.0689, $C_8H_9N_3O_2$ requires 179.0689. Data in accordance with literature[11].

6-Azido-1,2,3,4-tetrahydronaphthalene (1u)

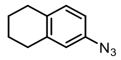

Following **AP1**, 5,6,7,8-tetrahydronaphthalen-2-amine (2.0 g, 13.6 mmol, 1 eq.) gave the title compound (>98%) as an oil. ^{1}H NMR ($CDCl_3$, 600 MHz) δ 7.04 (1H, d, J = 8.1 Hz), 6.76 (1H, d, J = 8.2 Hz), 6.74 (1H, s), 2.82 – 2.65 (4H, m), 1.85 – 1.70 (4H, m); ^{13}C NMR ($CDCl_3$, 151 MHz) δ 138.8, 136.9, 134.0, 130.4, 119.2, 116.3, 29.4, 28.9, 23.1, 22.9. HRMS (ESI): found M^+ 173.0948, $C_{10}H_{11}N_3$ requires 173.0948. Data in accordance with literature[7].

4-Azido-2,6-dimethyl-1,1'-biphenyl (1v)

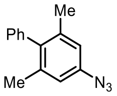

Following **AP1**, 2,6-dimethyl-[1,1'-biphenyl]-4-amine (2.0 g, 10.1 mmol, 1 eq.), prepared according to a known procedure[7], gave the title compound (>98%) as an oil. ^{1}H NMR ($CDCl_3$, 600 MHz) δ 7.43 (2H, t, J = 7.5 Hz), 7.35 (1H, t, J = 7.5 Hz), 7.12 (2H, d, J = 6.8 Hz), 6.79 (2H, s), 2.02 (6H, s); ^{13}C NMR ($CDCl_3$, 151 MHz) δ 140.2, 138.8, 138.3, 138.0, 129.2, 128.5, 126.8, 117.7, 20.9. HRMS (ESI): found $[MNH_2]^+$ (mass of the aniline) 197.1196, $C_{14}H_{15}N$ requires 197.1199. Data in accordance with literature[7].

Butyl 4-Azidobenzoate (1w)

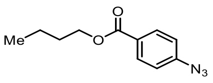

Following **AP1**, butyl 4-aminobenzoate (2.0 g, 10.3 mmol, 1 eq.) gave the title compound (>98%) as an oil. ^{1}H NMR ($CDCl_3$, 600 MHz) δ 8.02 (2H, d, *J* = 8.7 Hz), 7.05 (2H, d, *J* = 8.7 Hz), 4.31 (2H, t, *J* = 6.6 Hz), 1.81 – 1.67 (2H, m), 1.53 – 1.39 (2H, m), 0.97 (3H, t, *J* = 7.4 Hz); ^{13}C NMR ($CDCl_3$, 151 MHz) δ 165.8, 144.6, 131.3, 127.1, 118.8, 64.9, 30.7, 19.3, 13.7. HRMS (ESI): found MNa^+ 242.0902, $C_{11}H_{13}N_3O_2Na$ requires 242.0900. Data in accordance with literature[13].

3-(4-Azidophenyl)-3-ethylpiperidine-2,6-dione (1x)

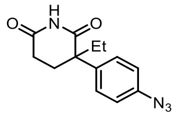

Following **AP1**, 3-(4-aminophenyl)-3-ethylpiperidine-2,6-dione (2.0 g, 8.6 mmol, 1 eq.) gave the title compound after precipitation from DCM/Pentane (>98%) as a solid. ^{1}H NMR ($CDCl_3$, 600 MHz) δ 7.90 (1H, s), 7.27 (2H, d, *J* = 8.7 Hz), 7.03 (2H, d, *J* = 8.6 Hz), 2.65 – 2.57 (1H, m), 2.45 – 2.32 (2H, m), 2.27 – 2.18 (1H, m), 2.09 – 1.84 (2H, m), 0.87 (3H, t, *J* = 7.4 Hz); ^{13}C NMR ($CDCl_3$, 151 MHz) δ 174.9, 172.0, 139.7, 135.5, 127.9, 119.7, 50.8, 33.1, 29.4, 27.1, 9.1. HRMS (ESI): found MNa^+ 281.1012, $C_{13}H_{14}N_4O_2Na$ requires 281.1009. Data in accordance with literature[7].

(8*S*,9*R*,13*R*,14*R*)-3-Azido-13-methyl-6,7,8,9,11,12,13,14,15,16-decahydro-17*H*-cyclopenta[*a*]phenanthren-17-one (1y)

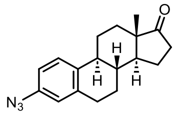

To (8*S*,9*R*,13*R*,14*R*)-13-methyl-3-(4,4,5,5-tetramethyl-1,3,2-dioxaborolan-2-yl)-6,7,8,9,11,12,13,14,15,16-decahydro-17*H*-cyclopenta[a]phenanthren-17-one (2.0 g, 5.26 mmol, 1 eq.), prepared according to a known procedure[14], in MeOH (26 mL) was added NaN_3 (513 mg, 7.89 mmol, 1.5 eq.) and $Cu(OAc)_2$ (96 mg, 0.53 mmol, 0.1 eq.). The solution was stirred at 55°C under air for 24 h. The mixture was then filtered through a pad of Celite®, concentrated under vacuo and purified by flash column chromatography (Silica gel, Pentane/EtoAc 100% to 80/20) to give the title compound (89%) as a waxy solid. ^{1}H NMR ($CDCl_3$, 600 MHz) δ 7.72 (2H, d, *J* = 8.2 Hz), 6.82 (1H dd, *J* = 8.4, 2.7 Hz), 6.76 (1H, s), 2.90 (2H, dd, *J* = 9.0, 4.3 Hz), 2.51 (1H, dd, *J* = 18.6, 9.3 Hz), 2.43 – 2.37 (1H, m), 2.31 – 2.25 (1H, m), 2.20 – 2.10 (1H, m), 2.09 – 2.00 (2H, m),

1.98 – 1.95 (1H, m), 1.67 – 1.58 (2H, m), 1.55 – 1.41 (2H, m), 1.48 – 1.41 (1H, m), 0.92 (3H, m); ^{13}C NMR ($CDCl_3$, 151 MHz) δ 220.9, 138.5, 137.6, 136.8, 126.9, 119.3, 116.6, 50.5, 48.1, 44.3, 38.2, 36.0, 31.7, 29.5, 26.5, 25.9, 21.7, 14.0. HRMS (ESI): found MNa^+ 318.1308, $C_{18}H_{21}N_3ONa$ requires 318.1582. Data in accordance with literature[14].

11. Substrate Scope

_N_-(2-((3',5'-dimethoxy-[1,1'-biphenyl]-2-yl)thio)-5-methylphenyl)-2,2,2-trifluoroacetamide (3bh)

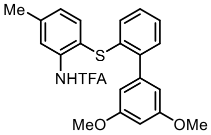

Following **GP1**, 1-azido-4-methylbenzene (31.9 mg, 0.24 mmol, 2.4 eq.) gave the title compound (44%) as an oil. ^{1}H NMR ($CDCl_3$, 600 MHz) δ 8.80 (1H, s), 8.16 (1H, s), 7.42 (1H, d, *J* = 7.9 Hz), 7.27 (1H, dd, *J* = 7.5, 1.7 Hz), 7.23 (1H, t, *J* = 7.5 Hz), 7.17 (1H, td, *J* = 7.6, 1.7 Hz), 7.03 (1H, d, *J* = 7.8 Hz), 6.82 (1H, dd, *J* = 7.9, 1.2 Hz), 6.51 (1H, t, *J* = 2.3 Hz), 6.48 (2H, d, *J* = 2.2 Hz), 3.82 (6H, s), 2.41 (3H, s); ^{13}C NMR ($CDCl_3$, 151 MHz) δ 160.6, 154.4 (q, *J* = 37.3 Hz), 141.8, 141.6, 141.5, 136.8, 136.6, 133.8, 130.3, 128.4, 127.3, 127.2, 126.5, 121.7, 119.1, 115.5 (q, *J* = 289.2 Hz), 107.2, 99.9, 55.3, 21.7; ^{19}F NMR ($CDCl_3$, 564 MHz) δ -76.0. HRMS (ESI): found MNa^+ 470.1000, $C_{23}H_{20}F_3NO_3SNa$ requires 470.1008.

_N_-(2-((3',5'-dimethoxy-[1,1'-biphenyl]-2-yl)thio)-5-ethylphenyl)-2,2,2-trifluoroacetamide (3ch)

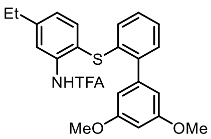

Following **GP1** and using Hexane as a solvent (2 mL, 0.05 M), 1-azido-4-ethylbenzene (35 mg, 0.24 mmol, 2.4 eq.) gave the title compound (52%) as an oil. ^{1}H NMR ($CDCl_3$, 600 MHz) δ 8.84 (1H, s), 8.22 (1H, s), 7.47 (1H, d, *J* = 7.9 Hz), 7.32 – 7.23 (2H, m), 7.21 (1H, td, *J* = 7.5, 1.9 Hz), 7.09 (1H, d, *J* = 7.9 Hz), 6.87 (1H, d, *J* = 7.7 Hz), 6.56 – 6.51 (1H, m), 6.51 (2H, s), 3.85 (6H, s), 2.73 (2H, q, *J* = 7.6 Hz), 1.30 (3H, t, *J* = 7.5 Hz); ^{13}C NMR ($CDCl_3$, 151 MHz) δ 160.8, 154.6 (d, *J* = 37.4 Hz), 148.0, 142.0, 141.7, 137.0, 136.8, 133.9, 130.5, 128.5, 127.5, 126.7, 126.2, 120.7,

119.4, 115.7 (d, J = 289.0 Hz), 107.4, 100.0, 55.5, 29.1, 15.3; ^{19}F NMR ($CDCl_3$, 564 MHz) δ -76.05. HRMS (ESI): found MNa^+ 484.1155, $C_{24}H_{22}F_3NO_3SNa$ requires 484.1165.

***N*-(2-((3',5'-dimethoxy-[1,1'-biphenyl]-2-yl)thio)-5-ethylphenyl)-2,2,2-trifluoroacetamide (3dh)**

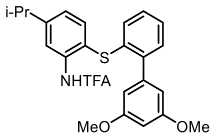

Following **GP1**, 1-azido-4-isopropylbenzene (39 mg, 0.24 mmol, 2.4 eq.) gave the title compound (42%) as an oil. ^{1}H NMR ($CDCl_3$, 600 MHz) δ 8.81 (1H, s), 8.22 (1H, s), 7.44 (1H, d, J = 7.9 Hz), 7.30 – 7.21 (2H, m), 7.18 (1H, td, J = 7.5, 1.9 Hz), 7.07 (1H, dd, J = 8.0, 1.9 Hz), 6.87 – 6.83 (1H, m), 6.50 (1H, d, J = 2.1 Hz), 6.48 (2H, d, J = 2.2 Hz), 3.81 (6H, s), 2.95 (1H, p, J = 6.9 Hz), 1.28 (6H, d, J = 6.9 Hz); ^{13}C NMR ($CDCl_3$, 151 MHz) δ 160.8, 154.5 (q, J = 37.4 Hz), 152.6, 142.0, 141.7, 137.0, 136.8, 133.8, 130.5, 128.5, 127.6, 126.7, 124.7, 121.3, 119.4, 115.7 (q, J = 289.0 Hz), 107.3, 100.0, 55.5, 34.4, 23.8; ^{19}F NMR ($CDCl_3$, 564 MHz) δ -76.07. HRMS (ESI): found M^+ 475.1426, $C_{25}H_{24}F_3NO_3S$ requires 475.1424.

***N*-(5-benzyl-2-((3',5'-dimethoxy-[1,1'-biphenyl]-2-yl)thio)phenyl)-2,2,2-trifluoroacetamide (3eh)**

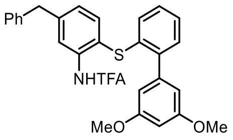

Following **GP1**, 1-azido-4-benzylbenzene (50 mg, 0.24 mmol, 2.4 eq.) gave the title compound (48%) as an oil. ^{1}H NMR ($CDCl_3$, 600 MHz) δ 8.78 (1H, s), 8.23 (1H, s), 7.42 (1H, d, J = 8.0 Hz), 7.32 (2H, t, J = 7.6 Hz), 7.28 – 7.24 (2H, m), 7.22 (3H, t, J = 6.6 Hz), 7.18 (1H, td, J = 7.6, 1.7 Hz), 7.00 (1H, dd, J = 8.0, 1.9 Hz), 6.85 (1H, dd, J = 7.9, 1.3 Hz), 6.50 (1H, t, J = 2.3 Hz), 6.46 (2H, d, J = 2.3 Hz), 4.01 (2H, s), 3.80 (6H, s); ^{13}C NMR ($CDCl_3$, 151 MHz) δ 160.8, 154.6 (q, J = 37.4 Hz), 144.8, 142.0, 141.9, 140.0, 137.1, 136.9, 133.6, 130.5, 129.1, 128.8, 128.6, 127.8, 127.0, 126.8, 126.7, 121.6, 120.2, 115.6 (q, J = 289.0 Hz), 107.3, 100.0, 55.5, 42.1; ^{19}F NMR ($CDCl_3$, 564 MHz) δ -76.05. HRMS (ESI): found MNa^+ 546.1326, $C_{29}H_{24}F_3NO_3SNa$ requires 546.1326.

***N*-(2-((3',5'-dimethoxy-[1,1'-biphenyl]-2-yl)thio)-5-(trifluoromethyl)phenyl)-2,2,2 trifluoroacetamide (3fh)**

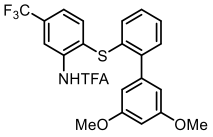

Following **GP1**, 1-azido-4-(trifluoromethyl)benzene (44.9 mg, 0.24 mmol, 2.4 eq.) gave the title compound (63%) as an oil. ^{1}H NMR ($CDCl_3$, 600 MHz) δ 8.69 (1H, s), 8.50 (1H, d, *J* = 1.9 Hz), 7.48 (1H, d, *J* = 8.1), 7.39 – 7.32 (2H, m), 7.31 – 7.27 (2H, m), 7.19 (1H, dd, *J* = 7.9, 1.3 Hz), 6.44 (1H, t, *J* = 2.3 Hz), 6.31 (2H, d, *J* = 2.3 Hz), 3.75 (6H, s); ^{13}C NMR ($CDCl_3$, 151 MHz) δ 160.8, 154.7 (q, *J* = 37.9 Hz), 143.5, 141.9, 136.6, 135.8, 132.0 (q, *J* = 37.7 Hz), 131.9, 131.1, 130.6, 128.8, 128.4, 125.5, 123.4 (q, *J* = 273.0 Hz), 122.7 (q, *J* = 3.7 Hz), 117.9 (q, *J* = 4.0 Hz), 115.4 (q, *J* = 289.0 Hz), 107.2, 99.6, 55.4; ^{19}F NMR ($CDCl_3$, 564 MHz) δ -63.03, -76.01. HRMS (ESI): found MNa^+ 524.0718, $C_{23}H_{17}F_6NO_3SNa$ requires 524.0725.

***N*-(5-(1-cyanocyclopentyl)-2-((3',5'-dimethoxy-[1,1'-biphenyl]-2-yl)thio)phenyl)-2,2,2-trifluoroacetamide (3gh)**

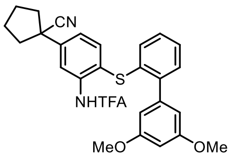

Following **GP1**, 1-(4-azidophenyl)cyclopentane-1-carbonitrile (50.9 mg, 0.24 mmol, 2.4 eq.) gave the title compound (42%) as an solid. ^{1}H NMR ($CDCl_3$, 600 MHz) δ 8.75 (1H, s), 8.35 (1H, d, *J* = 2.1 Hz), 7.47 (1H, d, *J* = 8.1 Hz), 7.35 (1H, dd, *J* = 8.2, 2.1 Hz), 7.29 – 7.27 (2H, d), 7.25 – 7.21 (1H, m), 6.99 (1H, d, *J* = 7.8 Hz), 6.47 (1H, t, *J* = 2.2 Hz), 6.40 (2H, d, *J* = 2.2 Hz), 3.79 (6H, s), 2.50 (2H, m), 2.13 – 2.01 (4H, m), 1.97 (2H, m); ^{13}C NMR ($CDCl_3$, 151 MHz) δ 160.8, 154.8 (q, *J* = 37.6 Hz), 142.9, 142.5, 141.9, 137.2, 136.6, 132.8, 130.8, 128.9, 128.7, 127.5, 124.6, 123.8, 123.0, 118.2, 115.5 (q, *J* = 288.6 Hz), 107.3, 99.8, 55.5, 47.9, 40.7, 24.4. ^{19}F NMR ($CDCl_3$, 564 MHz) δ -76.1. HRMS (ESI): found MNa^+ 549.1435, $C_{28}H_{25}F_3N_2O_3SNa$ requires 549.1436.

***N*-(2-((3',5'-dimethoxy-[1,1'-biphenyl]-2-yl)thio)-5-(methoxymethyl)phenyl)-2,2,2-trifluoroacetamide (3hh)**

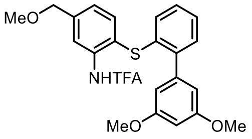

Following **GP1**, 1-azido-4-(methoxymethyl)benzene (39.2 mg, 0.24 mmol, 2.4 eq.) gave the title compound (40%) as an oil. ^{1}H NMR ($CDCl_3$, 600 MHz) δ 8.79 (1H, s), 8.28 (1H, d, *J* = 1.9 Hz), 7.56 – 6.81 (6H, m), 6.48 (3H, dd, *J* = 18.2, 2.3 Hz), 4.48 (2H, s), 3.81 (6H, s), 3.44 (3H, s); ^{13}C NMR ($CDCl_3$, 151 MHz) δ 160.8, 154.6 (q, *J* = 37.6 Hz), 141.9, 141.9, 141.9, 137.0, 136.8, 133.5, 130.5, 128.6, 127.9, 126.9, 125.3, 121.8, 120.1, 115.6 (q, *J* = 288.9 Hz), 107.3, 100.0, 74.0, 58.7, 55.5. ^{19}F NMR ($CDCl_3$, 564 MHz) δ -76.04. HRMS (ESI): found MNa^+ 500.1115, $C_{24}H_{22}F_3NO_4SNa$ requires 500.1119.

***N*-(5-(2-(benzyloxy)-1,1,1,3,3,3-hexafluoropropan-2-yl)-2-((3',5'-dimethoxy-[1,1'-biphenyl]-2-yl)thio)phenyl)-2,2,2-trifluoroacetamide (3ih)**

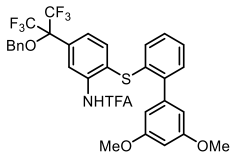

Following **GP1**, 1-azido-4-(2-(benzyloxy)-1,1,1,3,3,3-hexafluoropropan-2-yl)benzene (90 mg, 0.24 mmol, 2.4 eq.) gave the title compound (53%) as an oil. ^{1}H NMR ($CDCl_3$, 600 MHz) δ 8.59 (1H, s), 8.54 (1H, s), 7.44 (2H, m), 7.42 – 7.39 (3H, m), 7.38 – 7.33 (3H, m), 7.32 – 7.27 (3H, m), 6.37 (1H, t, *J* = 2.3 Hz), 6.34 (2H, d, *J* = 2.3 Hz), 4.65 (2H, s), 3.71 (6H, s); ^{13}C NMR ($CDCl_3$, 151 MHz) 160.7, 154.7 (d, *J* = 38.1 Hz), 143.6, 142.0, 136.3, 136.0, 135.1, 131.6, 131.4, 131.2, 129.8, 128.8, 128.5, 128.5, 127.8, 127.8, 125.7, 122.4 (d, *J* = 291.7 Hz), 121.0, 116.5, 114.6, 107.3, 99.3, 68.6, 55.4. ^{19}F NMR ($CDCl_3$, 564 MHz) δ -70.52, -75.97. HRMS (ESI): found MNa^+ 712.1163, $C_{32}H_{24}F_9NO_4SNa$ requires 712.1174.

***N*-(2-((3',5'-dimethoxy-[1,1'-biphenyl]-2-yl)thio)-5-(hydroxydiphenylmethyl)phenyl)-2,2,2-trifluoroacetamide (3jh)**

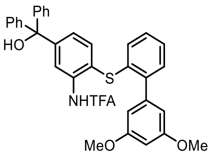

Following **GP1** and using Et_2O as a solvent (2 mL, 0.05 M), (4-azidophenyl)diphenylmethanol (72 mg, 0.24 mmol, 2.4 eq.) gave the title compound (72%) as an oil. ^{1}H NMR ($CDCl_3$, 600 MHz) δ 8.67 (1H, s), 8.27 (1H, d, J = 2.0 Hz), 7.38 (1H, d, J = 8.2 Hz), 7.36 – 7.32 (4H, m), 7.31 – 7.26 (8H, m), 7.25 – 7.21 (1H, m), 7.11 (1H, dd, J = 8.2, 2.0 Hz), 7.04 (1H, d, J = 7.7 Hz), 6.47 (1H, t, J = 2.3 Hz), 6.42 (2H, d, J = 2.3 Hz), 3.79 (6H, s), 2.97 (1H, s); ^{13}C NMR ($CDCl_3$, 151 MHz) δ 160.7, 154.5 (q, J = 37.1 Hz), 149.8, 146.2, 142.5, 142.0, 136.3, 135.6, 133.1, 130.7, 129.1, 128.6, 128.3, 127.9, 127.8, 127.4, 126.1, 122.4, 120.8, 115.6 (q, J = 289.1 Hz), 107.4, 99.8, 81.8, 55.5; ^{19}F NMR ($CDCl_3$, 564 MHz) δ -76.02. HRMS (ESI): found MNa^+ 638.1573, $C_{35}H_{28}F_3NO_4SNa$ requires 638.1583.

2,4-dimethoxy-5-(4-(4,4,5,5-tetramethyl-1,3,2-dioxaborolan-2-yl)-2 (2,2,2trifluoroacetamido)phenyl) -5H-dibenzo[b,d]thiophen-5-ium trifluoromethanesulfonate (4kh)

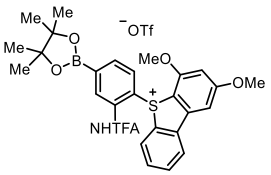

Following **GP2**, 2-(4-azidophenyl)-4,4,5,5-tetramethyl-1,3,2-dioxaborolane (59 mg, 0.24 mmol, 2.4 eq.) was converted into N-(2-((3',5'-dimethoxy-[1,1'-biphenyl]-2-yl)thio)-5-(4,4,5,5-tetramethyl-1,3,2-dioxaborolan-2-yl)phenyl)-2,2,2-trifluoroacetamide **(3kh)**, which was then subjected without any purification to **GP2a** yielding the title compound (78%) as an oil. ^{1}H NMR ($CDCl_3$, 600 MHz) δ 11.41 (1H, s), 8.53 (1H, d, J = 8.1 Hz), 8.18 (1H, d, J = 7.8 Hz), 8.08 (1H, s), 7.88 (1H, t, J = 7.7 Hz), 7.66 (1H, t, J = 8.0 Hz), 7.59 (1H, d, J = 8.2 Hz), 7.24 (1H, s), 6.53 – 6.45 (2H, m), 3.99 (3H, s), 3.84 (3H, s), 1.28 (12H, s); ^{13}C NMR ($CDCl_3$, 151 MHz) δ 167.9, 158.7, 158.2 (q, J = 39.2 Hz), 141.4, 140.0, 137.6, 136.0, 135.8, 134.6, 132.3, 131.4, 130.0, 126.3, 125.4, 124.5, 120.6 (q, J = 319.7 Hz), 116.1 (q, J = 287.4 Hz), 108.3, 101.3, 100.3, 85.0, 57.2,

56.8, 29.8, 24.9; ^{19}F NMR ($CDCl_3$, 564 MHz) δ -72.71, -76.18. HRMS (ESI): found $[M-OTf]^+$ 558.1729, $C_{28}H_{28}BF_3NO_5S^+$ requires 558.1733.

***N*-(4-((3',5'-dimethoxy-[1,1'-biphenyl]-2-yl)thio)-2',3',4',5'-tetrahydro-[1,1'-biphenyl]-3-yl)-2,2,2-trifluoroacetamide (3lh)**

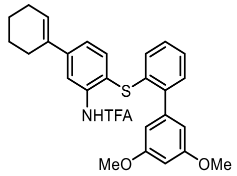

Following **GP1**, 4'-azido-2,3,4,5-tetrahydro-1,1'-biphenyl (47.8 mg, 0.24 mmol, 2.4 eq.) gave the title compound (65%) as an oil. ^{1}H NMR ($CDCl_3$, 600 MHz) δ 8.76 (1H, s), 8.35 (1H, d, *J* = 1.9 Hz), 7.43 (1H, d, *J* = 8.3 Hz), 7.28 – 7.20 (3H, m), 7.17 (1H, td, *J* = 7.6, 1.7 Hz), 6.86 (1H, dd, *J* = 7.9, 1.3 Hz), 6.49 (1H, t, *J* = 2.3 Hz), 6.46 (2H, d, *J* = 2.2 Hz), 6.26 (1H, dt, *J* = 4.1, 2.4 Hz), 3.80 (6H, d, *J* = 1.3 Hz), 2.41 (2H, td, *J* = 6.2, 3.8 Hz), 2.30 – 2.19 (2H, m), 1.84 – 1.73 (2H, m), 1.72 – 1.62 (2H, m); ^{13}C NMR ($CDCl_3$, 151 MHz) δ 160.61, 154.31, 145.37, 141.84, 141.63, 136.80, 136.38, 135.34, 133.71, 130.32, 128.38, 127.55, 127.33, 126.59, 122.78, 120.06, 117.50, 107.17, 99.86, 55.33, 27.10, 25.93, 22.86, 21.94; ^{19}F NMR ($CDCl_3$, 564 MHz) δ -76.08. HRMS (ESI): found M^+ 513.1578, $C_{28}H_{26}F_3NO_3S$ requires 513.1580.

***N*-(4-((3',5'-dimethoxy-[1,1'-biphenyl]-2-yl)thio)-[1,1'-biphenyl]-3-yl)-2,2,2-trifluoroacetamide (3mh)**

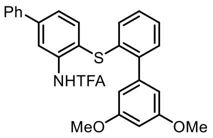

Following **GP1**, 4-azido-2,6-dimethyl-1,1'-biphenyl (46.8 mg, 0.24 mmol, 2.4 eq.) gave the title compound (43%) as an oil. ^{1}H NMR ($CDCl_3$, 600 MHz) δ 8.81 (1H, s), 8.56 (1H, d, *J* = 2.0 Hz), 7.63 (2H, d, *J* = 7.2), 7.55 (1H, d, *J* = 8.1 Hz), 7.49 – 7.44 (2H, m), 7.44 – 7.35 (2H, m), 7.30 – 7.26 (2H, m), 7.23 (1H, ddd, *J* = 7.8, 6.9, 2.0 Hz), 7.00 (1H, dd, *J* = 7.9, 1.2 Hz), 6.50 (1H, t, *J* = 2.3 Hz), 6.45 (2H, d, *J* = 2.3 Hz), 3.79 (6H, s); ^{13}C NMR ($CDCl_3$, 151 MHz) δ 160.8, 154.7 (q, *J* = 37.5 Hz), 143.8, 142.2, 142.0, 139.5, 137.3, 136.9, 133.5, 130.6, 129.1, 128.6, 128.4, 128.4, 127.3, 127.1, 125.0, 121.8, 119.7, 115.7 (q, *J* = 288.9 Hz), 107.3, 99.9, 55.5; ^{19}F NMR ($CDCl_3$, 564 MHz) δ -76.01. HRMS (ESI): found MNa^+ 532.1170, $C_{28}H_{22}F_3NO_3SNa$ requires 532.1170.

***N*-(4-((3',5'-dimethoxy-[1,1'-biphenyl]-2-yl)thio)-4'-methoxy-[1,1'-biphenyl]-3-yl)-2,2,2-trifluoroacetamide (3nh)**

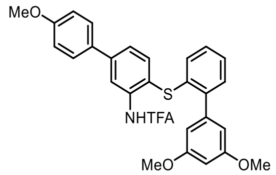

Following **GP1**, 4-azido-4'-methoxy-1,1'-biphenyl (54 mg, 0.24 mmol, 2.4 eq.) gave the title compound (60%) as an oil. ^{1}H NMR ($CDCl_3$, 600 MHz) δ 8.81 (1H, s), 8.53 (1H, d, *J* = 2.0 Hz), 7.57 (2H, d, *J* = 8.7 Hz), 7.53 (1H, d, *J* = 8.1 Hz), 7.38 (1H, dd, *J* = 8.1, 2.0 Hz), 7.28 (1H, dd, *J* = 7.5, 1.9 Hz), 7.27 – 7.24 (2H, m), 7.21 (1H, td, *J* = 7.5, 1.9 Hz), 7.00 – 6.93 (3H, m), 6.50 (1H, t, *J* = 2.3 Hz), 6.46 (2H, d, *J* = 2.3 Hz), 3.86 (3H, s), 3.79 (6H, s); ^{13}C NMR ($CDCl_3$, 151 MHz) δ 160.8, 160.0, 154.7 (q, *J* = 37.5 Hz), 143.5, 142.0, 142.0, 137.3, 137.0, 133.7, 131.9, 130.6, 128.6, 128.4, 128.1, 127.0, 124.5, 120.8, 119.1, 115.7 (q, *J* = 288.8 Hz), 114.5, 107.3, 100.0, 55.5, 55.5; ^{19}F NMR ($CDCl_3$, 564 MHz) δ -76.02. HRMS (ESI): found MH^+ 540.1447, $C_{29}H_{25}F_3NO_4S$ requires 540.1451.

***N*-(4-((3',5'-dimethoxy-[1,1'-biphenyl]-2-yl)thio)-4'-iodo-[1,1'-biphenyl]-3-yl)-2,2,2-trifluoroacetamide (3oh)**

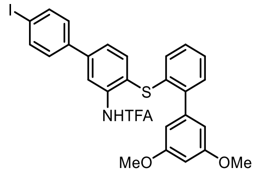

Following **GP1** and using TFT as a solvent (2 mL, 0.05 M), 4'-azido-3-fluoro-1,1'-biphenyl (77.4 mg, 0.24 mmol, 2.4 eq.) gave the title compound (62%) as an oil. ^{1}H NMR ($CDCl_3$, 600 MHz) δ 8.78 (1H, s), 8.50 (1H, d, *J* = 2.0 Hz), 7.78 (2H, d, *J* = 8.5 Hz), 7.53 (1H, d, *J* = 8.1 Hz), 7.38 – 7.32 (3H, m), 7.27 (2H, d, *J* = 16.0 Hz), 7.23 (1H, ddd, *J* = 7.9, 6.3, 2.7 Hz), 7.04 – 6.98 (1H, m), 6.48 (1H, t, *J* = 2.3 Hz), 6.43 (2H, d, *J* = 2.3 Hz), 3.77 (6H, s); ^{13}C NMR ($CDCl_3$, 151 MHz) δ 160.8, 142.5, 142.3, 142.0, 139.0, 138.2, 138.2, 137.3, 136.9, 133.3, 130.7, 129.1, 128.6, 127.3, 124.7, 122.6, 119.4, 107.3, 99.9, 94.4, 55.5; ^{19}F NMR ($CDCl_3$, 564 MHz) δ -76.02. HRMS (ESI): found MNa^+ 658.0122, $C_{28}H_{21}F_3INO_3SNa$ requires 658.0131.

***N*-(4-((3',5'-dimethoxy-[1,1'-biphenyl]-2-yl)thio)-3'-fluoro-[1,1'-biphenyl]-3-yl)-2,2,2-trifluoroacetamide (3ph)**

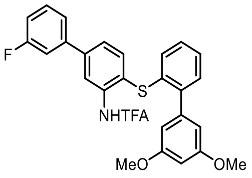

Following **GP1**, 4'-azido-3-fluoro-1,1'-biphenyl (51.1 mg, 0.24 mmol, 2.4 eq.) gave the title compound (76%) as an oil. ^{1}H NMR ($CDCl_3$, 600 MHz) δ 8.79 (1H, s), 8.53 (1H, d, *J* = 2.0 Hz), 7.54 (1H, d, *J* = 8.1 Hz), 7.46 – 7.36 (3H, m), 7.34 – 7.18 (4H, m), 7.08 (1H, ddt, *J* = 9.0, 6.8, 2.3 Hz), 7.03 (1H, d, *J* = 7.8 Hz), 6.49 (1H, t, *J* = 2.3 Hz), 6.43 (2H, d, *J* = 2.3 Hz), 3.78 (6H, s); ^{13}C NMR ($CDCl_3$, 151 MHz) δ 160.8, 142.4 (q, *J* = 54.4 Hz), 142.0, 141.8 (d, *J* = 7.8 Hz), 137.3, 136.8, 133.3, 130.7, 130.6 (d, *J* = 8.5 Hz), 128.7, 128.6, 127.3, 124.9, 123.0, 123.0, 122.8, 119.7, 115.2 (d, *J* = 21.2 Hz), 114.3 (d, *J* = 22.4 Hz), 107.3, 99.9, 55.5; ^{19}F NMR ($CDCl_3$, 564 MHz) δ -73.61, -112.60. HRMS (ESI): found MNa^+ 550.1064, $C_{28}H_{21}F_4NO_3SNa$ requires 550.1076.

***N*-(2-((3',5'-dimethoxy-[1,1'-biphenyl]-2-yl)thio)-6-methoxyphenyl)-2,2,2-trifluoroacetamide (3qh´)**

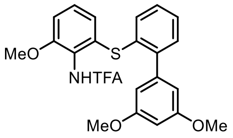

Following **GP1**, 1-azido-3-methoxybenzene (36 mg, 0.24 mmol, 2.4 eq.) gave the title compound (71%) as an oil. ^{1}H NMR ($CDCl_3$, 600 MHz) δ; ^{13}C NMR ($CDCl_3$, 151 MHz) δ 8.52 (1H, s), 8.16 (1H, d, *J* = 9.0 Hz), 7.29 (1H, dd, *J* = 7.5, 1.9 Hz), 7.28 – 7.25 (1H, m), 7.21 (1H, td, *J* = 7.5, 1.9 Hz), 7.03 (1H, d, *J* = 2.9 Hz), 6.99 – 6.94 (2H, m), 6.49 (1H, t, *J* = 2.3 Hz), 6.45 (2H, d, *J* = 2.2 Hz), 3.81 (6H, s), 3.78 (3H, s); ^{13}C NMR ($CDCl_3$, 151 MHz) δ 160.7, 157.4, 154.3 (q, *J* = 37.2 Hz), 142.2, 142.0, 133.2, 130.6, 129.9, 128.6, 128.4, 127.1, 124.8, 122.7, 121.3, 115.9, 115.8 (q, *J* = 288.9 Hz), 107.3, 100.0, 55.7, 55.5; ^{19}F NMR ($CDCl_3$, 564 MHz) δ -75.94. HRMS (ESI): found M^+ 463.1058, $C_{23}H_{20}F_3NO_4S$ requires 463.1060.

***N*-(4-(*tert*-butyl)-2-((3',5'-dimethoxy-[1,1'-biphenyl]-2-yl)thio)phenyl)-2,2,2-trifluoroacetamide (3rh)**

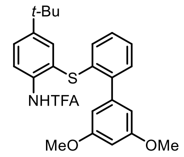

Following **GP1**, 1-azido-3-(*tert*-butyl)benzene (42 mg, 0.24 mmol, 2.4 eq.) gave the title compound (27%) as an oil. ^{1}H NMR ($CDCl_3$, 600 MHz) δ 8.70 (1H, s), 8.21 (1H, d, *J* = 8.7 Hz), 7.55 (1H, d, *J* = 2.3 Hz), 7.48 (1H, dd, *J* = 8.6, 2.3 Hz), 7.29 (1H, dd, *J* = 7.5, 1.6 Hz), 7.24 (1H, td, *J* = 7.4, 1.3 Hz), 7.19 (1H, td, *J* = 7.6, 1.7 Hz), 6.82 (1H, dd, *J* = 7.9, 1.3 Hz), 6.50 (3H, s), 3.82 (6H, s), 1.30 (9H, s); ^{13}C NMR ($CDCl_3$, 151 MHz) δ 160.8, 154.5 (q, *J* = 37.4 Hz), 149.9, 142.0, 141.6, 134.4, 133.7, 133.7, 130.5, 128.6, 128.0, 127.4, 126.7, 122.2, 121.0, 115.7 (q, *J* = 289.0 Hz), 107.3, 100.1, 55.5, 34.8, 31.3; ^{19}F NMR ($CDCl_3$, 564 MHz) δ -76.02. HRMS (ESI): found MNa^+ 512.1466, $C_{26}H_{26}F_3NO_3SNa$ requires 512.1478.

***N*-(2-(*tert*-butyl)-6-((3',5'-dimethoxy-[1,1'-biphenyl]-2-yl)thio)phenyl)-2,2,2-trifluoroacetamide (3rh′)**

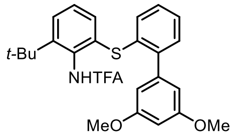

Following **GP1**, 1-azido-3-(*tert*-butyl)benzene (42 mg, 0.24 mmol, 2.4 eq.) gave the title compound (29%) as an oil. ^{1}H NMR ($CDCl_3$, 600 MHz) δ 7.54 (1H, dd, *J* = 7.6, 1.5 Hz), 7.36 (1H, td, *J* = 7.5, 1.6 Hz), 7.32 (1H, td, *J* = 7.5, 1.7 Hz), 7.22 (3H, s), 7.12 (1H, t, *J* = 7.9 Hz), 7.06 (1H, dd, *J* = 7.8, 1.4 Hz), 6.37 (1H, t, *J* = 2.3 Hz), 6.29 (2H, d, *J* = 2.2 Hz), 3.70 (6H, s), 1.31 (9H, s); ^{13}C NMR ($CDCl_3$, 151 MHz) δ 160.0, 156.6 (q, *J* = 36.9 Hz), 148.3, 144.8, 143.0, 138.7, 134.1, 133.9, 131.4, 131.4, 130.9, 129.1, 128.4 (q, *J* = 5.1 Hz), 126.1, 116.1 (d, *J* = 289.1 Hz), 107.3, 99.8, 55.4, 35.6, 30.9, 29.9; ^{19}F NMR ($CDCl_3$, 564 MHz) δ -75.64. HRMS (ESI): found MNa^+ 512.1467, $C_{26}H_{26}F_3NO_3SNa$ requires 512.1478.

***N*-(2-((3',5'-dimethoxy-[1,1'-biphenyl]-2-yl)thio)-4,6-dimethylphenyl)-2,2,2-trifluoroacetamide (3sh)**

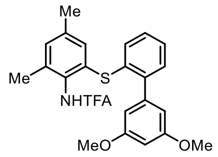

Following **GP1**, 1-azido-3,5-dimethylbenzene (35.9 mg, 0.24 mmol, 2.4 eq.) gave the title compound (80%) as an oil. ^{1}H NMR ($CDCl_3$, 600 MHz) δ 7.51 (1H, s), 7.29 – 7.24 (2H, m), 7.23 – 7.19 (1H, m), 7.11 (1H, dd, J = 7.7, 1.0 Hz), 7.07 (1H, d, J = 1.9 Hz), 7.03 (1H, d, J = 2.0 Hz), 6.46 (1H, t, J = 2.3 Hz), 6.39 (2H, d, J = 2.3 Hz), 3.77 (6H, s), 2.26 (3H, s), 2.15 (3H, s); ^{13}C NMR ($CDCl_3$, 151 MHz) δ 160.4, 154.8 (q, J = 37.0 Hz), 142.5, 142.4, 138.5, 136.0, 133.7, 133.3, 132.3, 130.8, 130.5, 130.4, 129.7, 128.4, 127.1, 115.7 (q, J = 288.9 Hz), 107.1, 99.7, 55.3, 20.8, 18.5; ^{19}F NMR ($CDCl_3$, 564 MHz) δ -75.50. HRMS (ESI): found MNa^+ 484.1157, $C_{24}H_{22}F_3NO_3SNa$ requires 484.1164.

***N*-(2-((3',5'-dimethoxy-[1,1'-biphenyl]-2-yl)thio)-4,6-dimethoxyphenyl)-2,2,2-trifluoroacetamide (3th)**

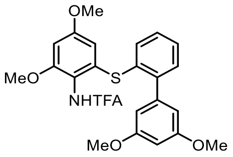

Following **GP1**, 1-azido-3,5-dimethoxybenzene (43 mg, 0.24 mmol, 2.4 eq.) gave the title compound (80%) as an oil. ^{1}H NMR ($CDCl_3$, 600 MHz) δ 7.36 – 7.27 (5H, m), 7.17 (1H, s), 6.42 (1H, s), 6.39 (3H, s), 3.79 (3H, s), 3.76 (6H, s), 3.69 (3H, s); ^{13}C NMR ($CDCl_3$, 151 MHz) δ 160.4, 160.2, 155.8, 155.3 (q, J = 36.2 Hz), 143.6, 142.5, 135.3, 133.4, 131.8, 130.6, 128.5, 127.8, 116.3, 116.0 (q, J = 288.9 Hz), 109.4, 107.3, 99.8, 99.2, 56.2, 55.7, 55.4; ^{19}F NMR ($CDCl_3$, 564 MHz) δ -75.38. HRMS (ESI): found MNa^+ 516.1049, $C_{24}H_{22}F_3NO_5SNa$ requires 516.1063.

***N*-(3-((3',5'-dimethoxy-[1,1'-biphenyl]-2-yl)thio)-5,6,7,8-tetrahydronaphthalen-2-yl)-2,2,2-trifluoroacetamide (3uh)**

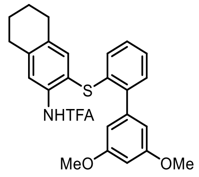

Following **GP1**, 5-azido-1,2,3,4-tetrahydronaphthalene (41.5 mg, 0.24 mmol, 2.4 eq.) gave the title compound (37%) as an oil. ^{1}H NMR ($CDCl_3$, 600 MHz) δ 8.73 (1H, s), 8.03 (1H, s), 7.40 – 7.14 (4H, m), 6.87 (1H, dd, *J* = 7.7, 1.5 Hz), 6.52 (1H, d, *J* = 2.1 Hz), 6.51 (2H, d, *J* = 2.2 Hz), 3.84 (6H, s), 2.83 (2H, t, *J* = 3.5 Hz), 2.72 (2H, d, *J* = 5.2 Hz), 1.82 (4H, p, *J* = 3.2 Hz); ^{13}C NMR ($CDCl_3$, 151 MHz) δ 160.7, 154.4 (q, *J* = 37.2 Hz), 142.0, 141.6, 140.8, 137.4, 135.9, 134.3, 130.4, 128.5, 128.2, 127.9, 126.6, 121.7, 119.0, 115.7 (q, *J* = 289.1 Hz), 107.3, 100.1, 55.5, 29.7, 28.9, 22.9, 22.9; ^{19}F NMR ($CDCl_3$, 564 MHz) δ -75.50. HRMS (ESI): found MNa$^+$ 510.1314, $C_{26}H_{24}F_3NO_3SNa$ requires 510.1321.

***N*-(2-((3',5'-dimethoxy-[1,1'-biphenyl]-2-yl)thio)-5,6,7,8-tetrahydronaphthalen-1-yl)-2,2,2-trifluoroacetamide (3uh´)**

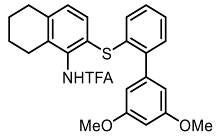

Following **GP1**, 6-azido-1,2,3,4-tetrahydronaphthalene (41.5 mg, 0.24 mmol, 2.4 eq.) gave the title compound (40%) as an oil. ^{1}H NMR ($CDCl_3$, 600 MHz) δ 7.40 (1H, s), 7.24 – 7.10 (3H, m), 7.07 (2H, d, *J* = 7.0 Hz), 6.93 (1H, d, *J* = 8.0 Hz), 6.36 (1H, d, *J* = 2.3 Hz), 6.29 (2H, d, *J* = 2.3 Hz), 3.68 (6H, s), 2.70 (2H, t, *J* = 6.1 Hz), 2.42 (2H, t, *J* = 6.0 Hz), 1.69 (4H, q, *J* = 7.2 Hz); ^{13}C NMR ($CDCl_3$, 151 MHz) δ 160.5, 155.1 (q, *J* = 37.0 Hz), 142.7, 142.7, 139.7, 135.8, 134.4, 133.4, 132.1, 130.5, 130.0, 129.9, 128.4, 128.2, 127.1, 115.9 (q, *J* = 288.9 Hz), 107.2, 99.7, 55.4, 29.7, 25.5, 22.5, 22.4; ^{19}F NMR ($CDCl_3$, 564 MHz) δ -75.97. HRMS (ESI): found MNa$^+$ 510.1319, $C_{26}H_{24}F_3NO_3SNa$ requires 510.1321.

***N*-(4-((3',5'-dimethoxy-[1,1'-biphenyl]-2-yl)thio)-2,6-dimethyl-[1,1'-biphenyl]-3-yl)-2,2,2-trifluoroacetamide (3vh)**

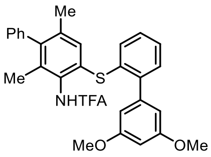

Following **GP1**, 4-azido-2,6-dimethyl-1,1'-biphenyl (53.5 mg, 0.24 mmol, 2.4 eq.) gave the title compound (59%) as an oil. ^{1}H NMR ($CDCl_3$, 600 MHz) δ 7.58 (1H, s), 7.44 (2H, t, *J* = 7.6 Hz), 7.36 (1H, t, *J* = 7.3 Hz), 7.32 – 7.25 (4H, m), 7.13 (1H, d, *J* = 7.3 Hz), 7.12 (2H, s), 6.44 (1H, d, *J* = 2.3 Hz), 6.42 (2H, d, *J* = 2.3 Hz), 3.78 (6H, s), 1.93 (3H, s), 1.80 (3H, s); ^{13}C NMR ($CDCl_3$, 151 MHz) δ 160.5, 155.2 (q, *J* = 37.1 Hz), 143.6, 142.8, 142.7, 140.0, 137.1, 134.6, 133.9, 133.6, 131.2, 130.7, 130.5, 129.9, 129.0, 128.8, 128.5, 127.4, 127.3, 116.0 (q, *J* = 288.8 Hz), 107.4, 99.5, 55.5, 20.9, 16.8; ^{19}F NMR ($CDCl_3$, 564 MHz) δ -75.46. HRMS (ESI): found MNa^+ 560.1467, $C_{30}H_{26}F_3NO_3SNa$ requires 560.1477.

Butyl 4-((3',5'-dimethoxy-[1,1'-biphenyl]-2-yl)thio)-3-(2,2,2-trifluoroacetamido)benzoate (3wh)

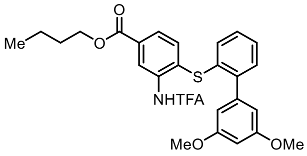

Following **GP1**, butyl 4-azidobenzoate (52.6 mg, 0.24 mmol, 2.4 eq.) gave the title compound (58%) as an oil. ^{1}H NMR ($CDCl_3$, 600 MHz) δ 8.79 (1H, d, *J* = 1.8 Hz), 8.58 (1H, s), 7.81 (1H, dd, *J* = 8.1, 1.8 Hz), 7.46 (1H, d, *J* = 8.1 Hz), 7.34 – 7.22 (3H, m), 7.11 (1H, dd, *J* = 7.8, 1.2 Hz), 6.45 (1H, t, *J* = 2.3 Hz), 6.35 (2H, d, *J* = 2.3 Hz), 4.34 (2H, t, *J* = 6.7 Hz), 3.76 (6H, s), 1.79 – 1.63 (2H, m), 1.52 – 1.44 (2H, m), 0.98 (3H, t, *J* = 7.4 Hz); ^{13}C NMR ($CDCl_3$, 151 MHz) δ 165.5, 160.7, 154.7 (q, *J* = 37.8 Hz), 143.2, 141.9, 136.1, 135.3, 132.2, 130.9, 130.2, 130.0, 128.7, 128.0, 127.2, 122.1, 115.6 (q, *J* = 289.0 Hz), 107.3, 99.7, 67.2, 65.5, 55.4, 30.8, 19.3, 13.9; ^{19}F NMR ($CDCl_3$, 564 MHz) δ -75.97. HRMS (ESI): found MNa^+ 556.1369, $C_{27}H_{26}F_3NO_5SNa$ requires 556.1376.

***N*-(2-((3',5'-dimethoxy-[1,1'-biphenyl]-2-yl)thio)-5-(3-ethyl-2,6-dioxopiperidin-3-yl)phenyl)-2,2,2-trifluoroacetamide (3xh)**

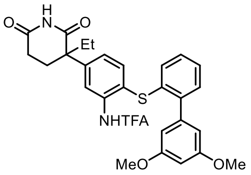

Following **GP1**, 3-(4-azidophenyl)-3-ethylpiperidine-2,6-dione (62 mg, 0.24 mmol, 2.4 eq.) gave the title compound (69%) as an oil. ^{1}H NMR ($CDCl_3$, 600 MHz) δ 8.73 (1H, s), 8.30 (1H, d, *J* = 2.2 Hz), 7.84 (1H, s), 7.43 (1H, d, *J* = 8.2 Hz), 7.32 – 7.26 (2H, m), 7.26 – 7.21 (1H, m), 7.09 (1H, dd, *J* = 8.2, 2.1 Hz), 7.01 (1H, d, *J* = 8.7 Hz), 6.46 (1H, t, *J* = 2.3 Hz), 6.39 (2H, d, *J* = 2.2 Hz), 3.79 (6H, s), 2.71 – 2.62 (1H, m), 2.49 – 2.41 (1H, m), 2.40 – 2.33 (1H, m), 2.26 (1H, td, *J* = 14.0, 4.4 Hz), 2.13 – 2.04 (1H, m), 1.98 – 1.88 (1H, m), 0.89 (3H, t, *J* = 7.4 Hz); ^{13}C NMR ($CDCl_3$, 151 MHz) δ 174.5, 172.0, 160.8, 154.9 (q, *J* = 37.8 Hz), 142.7, 142.0, 141.9, 137.2, 136.6, 132.6, 130.8, 129.3, 128.7, 127.9, 127.6, 124.7, 123.1, 115.5 (q, *J* = 37.8 Hz), 107.4, 99.7, 55.5, 51.4, 32.9, 29.3, 27.1, 9.2; ^{19}F NMR ($CDCl_3$, 564 MHz) δ -76.05. HRMS (ESI): found MNa^+ 595.1476, $C_{29}H_{27}F_3N_2O_5SNa$ requires 595.1485.

***N*-((8*R*,9*S*,13*S*,14*S*)-3-((3',5'-dimethoxy-[1,1'-biphenyl]-2-yl)thio)-13-methyl-17-oxo-7,8,9,11,12,13,14,15,16,17-decahydro-6*H*-cyclopenta[a]phenanthren-2-yl)-2,2,2-trifluoroacetamide (3yh)**

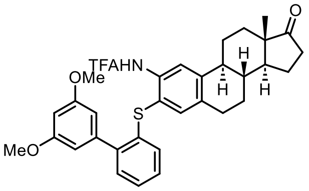

Following **GP1**, (8*R*,9*S*,13*S*,14*S*)-3-azido-13-methyl-6,7,8,9,11,12,13,14,15,16-decahydro-17*H*-cyclopenta[a]phenanthren-17-one (71 mg, 0.24 mmol, 2.4 eq.) gave the title compound (34%) as an oil. ^{1}H NMR ($CDCl_3$, 600 MHz) δ 8.68 (1H, s), 8.25 (1H, s), 7.29 – 7.23 (1H, m), 7.23 (1H, d, *J* = 5.7 Hz), 7.18 (1H, t, *J* = 7.1 Hz), 6.87 (1H, d, *J* = 7.9 Hz), 6.49 (1H, s), 6.47 (2H, s), 3.82 (6H, s), 2.86 (2H, d, *J* = 7.5 Hz), 2.52 (1H, dd, *J* = 19.0, 8.8 Hz), 2.44 (1H, dd, *J* = 13.5, 3.6 Hz), 2.32 (1H, td, *J* = 11.8, 11.2, 3.9 Hz), 2.22 – 2.11 (1H, m), 2.10 – 2.01 (2H, m), 1.99 (1H, d, *J* = 12.9 Hz), 1.69 – 1.40 (7H, m), 0.93 (3H, s); ^{13}C NMR ($CDCl_3$, 151 MHz) δ 220.6, 160.7, 143.2, 142.0, 141.7, 137.0, 135.3, 134.7, 133.8, 130.5, 128.5, 127.6, 126.7, 119.7, 118.5, 107.3, 100.0, 55.5,

50.6, 48.1, 44.8, 37.9, 36.0, 31.6, 28.9, 26.3, 25.7, 21.7, 14.0; ^{19}F NMR ($CDCl_3$, 564 MHz) δ -76.06. HRMS (ESI): found MNa^+ 632.2043, $C_{34}H_{34}F_3NO_4SNa$ requires 632.2052.

N-((8R,9S,13S,14S)-3-((3',5'-dimethoxy-[1,1'-biphenyl]-2-yl)thio)-13-methyl-17-oxo-7,8,9,11,12,13,14,15,16,17-decahydro-6H-cyclopenta[a]phenanthren-4-yl)-2,2,2-trifluoroacetamide (3yh′)

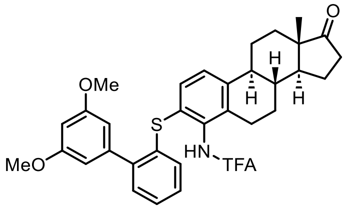

Following **GP1**, (8*R*,9*S*,13*S*,14*S*)-3-azido-13-methyl-6,7,8,9,11,12,13,14,15,16-decahydro-17*H*-cyclopenta[a]phenanthren-17-one (71 mg, 0.24 mmol, 2.4 eq.) gave the title compound (33%) as an oil. ^{1}H NMR ($CDCl_3$, 600 MHz) δ 7.49 (1H, s), 7.29 – 7.14 (6H, m), 6.44 (1H, t, *J* = 2.3 Hz), 6.36 (2H, d, *J* = 2.3 Hz), 3.76 (6H, s), 2.72 – 2.57 (2H, m), 2.51 (1H, dd, *J* = 19.2, 8.7 Hz), 2.42 – 2.32 (1H, m), 2.32 – 2.23 (1H, m), 2.20 – 2.10 (1H, m), 2.09 – 1.99 (2H, m), 1.98 – 1.95 (1H, m), 1.68 – 1.44 (6H, m), 0.93 (3H, s); ^{13}C NMR ($CDCl_3$, 151 MHz) δ 220.7, 160.5, 155.1 (q, *J* = 36.8 Hz), 142.8, 142.6, 142.2, 135.3, 134.0, 133.0, 132.2, 130.5, 130.4, 129.1, 128.5, 127.3, 126.4, 115.9 (q, *J* = 289.0 Hz), 107.2, 99.7, 55.5, 50.5, 48.0, 44.9, 37.4, 35.9, 31.7, 25.9, 25.9, 25.5, 21.7, 14.0; ^{19}F NMR ($CDCl_3$, 564 MHz) δ -75.42. HRMS (ESI): found MNa^+ 632.2043, $C_{34}H_{34}F_3NO_4SNa$ requires 632.2053.

2,2,2-Trifluoro-*N*-(*m*-tolyl)acetamide (5b)

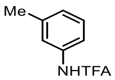

Following **GP2**, N-(2-((3',5'-dimethoxy-[1,1'-biphenyl]-2-yl)thio)-5-methylphenyl)-2,2,2-trifluoroacetamide (47.8 mg, 0.24 mmol, 2.4 eq.) gave the title compound (97%) as n oil. ^{1}H NMR ($CDCl_3$, 600 MHz) δ 7.80 (1H, s), 7.40 (1H, s), 7.35 (1H, d, *J* = 8.2 Hz), 7.28 (1H, t, *J* = 7.8 Hz), 7.06 (1H, d, *J* = 7.5), 2.37 (3H, s); ^{13}C NMR ($CDCl_3$, 151 MHz) δ 154.8 (q, *J* = 37.4 Hz), 139.7, 135.1, 129.4, 127.3, 121.2, 117.7, 115.9 (q, *J* = 288.9 Hz), 21.6; ^{19}F NMR ($CDCl_3$, 564 MHz) δ -75.65. HRMS (ESI): found MNa^+ 226.0445, $C_9H_8F_3NONa$ requires 226.0450.

***N*-(3-ethylphenyl)-2,2,2-trifluoroacetamide (5c)**

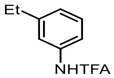

Following **GP2**, *N*-(2-((3',5'-dimethoxy-[1,1'-biphenyl]-2-yl)thio)-5-ethylphenyl)-2,2,2-trifluoroacetamide (47.8 mg, 0.24 mmol, 2.4 eq.) gave the title compound (98%) as an oil. ^{1}H NMR ($CDCl_3$, 600 MHz) δ 8.03 (1H, s), 7.41 (1H, s), 7.39 (1H, d, *J* = 8.03 Hz), 7.30 (1H, t, *J* = 7.8 Hz), 7.09 (1H, d, *J* = 7.6), 2.65 (2H, q, *J* = 7.6), 1.24 (3H, t, *J* = 7.6 Hz); ^{13}C NMR ($CDCl_3$, 151 MHz) δ 155.0 (q, *J* = 37.1 Hz), 146.0, 135.2, 129.4, 126.1, 120.2, 118.0, 115.9 (q, *J* = 288.8 Hz), 28.9, 15.5; ^{19}F NMR ($CDCl_3$, 564 MHz) δ -75.78. HRMS (ESI): found MNa^+ 240.0601, $C_{10}H_{10}F_3NONa$ requires 240.0607.

2,2,2-Trifluoro-*N*-(3-isopropylphenyl)acetamide (5d)

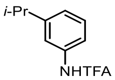

Following **GP2**, *N*-(2-((3',5'-dimethoxy-[1,1'-biphenyl]-2-yl)thio)-5-isopropylphenyl)-2,2,2-trifluoroacetamide (47.8 mg, 0.24 mmol, 2.4 eq.) gave the title compound (98%) as an oil. ^{1}H NMR ($CDCl_3$, 600 MHz) δ 8.00 (1H, s), 7.43 – 7.39 (2H, m), 7.31 (1H, t, *J* = 8.2 Hz), 7.12 (1H, d, *J* = 7.8 Hz), 2.91 (1H, hept, *J* = 6.9 Hz), 1.25 (6H, d, *J* = 7.0 Hz); ^{13}C NMR ($CDCl_3$, 151 MHz) δ 154.9 (q, *J* = 37.3 Hz), 150.6, 135.2, 129.4, 124.7, 118.8, 118.2, 115.9 (q, *J* = 288.7 Hz), 34.2, 23.9; ^{19}F NMR ($CDCl_3$, 564 MHz) δ -75.69. HRMS (ESI): found M^+ 231.0867, $C_{11}H_{12}F_3NO$ requires 231.0866.

***N*-(3-benzylphenyl)-2,2,2-trifluoroacetamide (5e)**

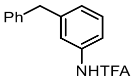

Following **GP2**, *N*-(5-benzyl-2-((3',5'-dimethoxy-[1,1'-biphenyl]-2-yl)thio)phenyl)-2,2,2-trifluoroacetamide (47.8 mg, 0.24 mmol, 2.4 eq.) gave the title compound (95%) as an oil. ^{1}H NMR ($CDCl_3$, 600 MHz) δ 7.76 (1H, s), 7.47 (1H, d, *J* = 6.8 Hz), 7.35 – 7.27 (4H, m), 7.23 (1H, d, *J* = 7.5 Hz), 7.19 (2H, d, *J* = 7.0 Hz), 7.09 (1H, d, *J* = 7.7 Hz), 3.99 (2H, s); ^{13}C NMR ($CDCl_3$, 151 MHz) δ 154.8 (q, *J* = 37.8 Hz), 143.0, 140.4, 135.4, 129.7, 129.1, 128.8, 127.1, 126.5, 120.9,

118.4, 115.8 (q, J = 288.2 Hz), 41.9; ^{19}F NMR ($CDCl_3$, 564 MHz) δ -70.8. HRMS (ESI): found MNa^+ 302.0768, $C_{15}H_{12}F_3NONa$ requires 302.0769.

2,2,2-Trifluoro-*N*-(3-(trifluoromethyl)phenyl)acetamide (5f)

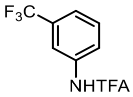

Following **GP2**, *N*-(2-((3',5'-dimethoxy-[1,1'-biphenyl]-2-yl)thio)-5-(trifluoromethyl)phenyl)-2,2,2 trifluoroacetamide (47.8 mg, 0.24 mmol, 2.4 eq.) gave the title compound (94%) as an oil. ^{1}H NMR ($CDCl_3$, 600 MHz) δ 7.98 (1H, s), 7.87 (1H, s), 7.80 (1H, dt, J = 7.9, 1.8 Hz), 7.57 – 7.51 (2H, m); ^{13}C NMR ($CDCl_3$, 151 MHz) δ 155.1 (q, J = 37.8 Hz), 135.8, 132.1 (q, J = 33.2 Hz), 130.3, 123.7, 123.4 (q, J = 273.3 Hz), 123.2 (q, J = 4.5 Hz), 117.5 (q, J = 4.5 Hz), 115.6 (q, J = 288.4 Hz); ^{19}F NMR ($CDCl_3$, 564 MHz) δ -62.95, -75.84. HRMS (ESI): found MNa^+ 280.0165, $C_9H_5F_6NONa$ requires 280.0158.

***N*-(3-(1-cyanocyclopentyl)phenyl)-2,2,2-trifluoroacetamide (5g)**

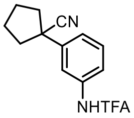

Following **GP2**, *N*-(5-(1-cyanocyclopentyl)-2-((3',5'-dimethoxy-[1,1'-biphenyl]-2-yl)thio)phenyl)-2,2,2-trifluoroacetamide (47.8 mg, 0.24 mmol, 2.4 eq.) gave the title compound (91%) as an oil. ^{1}H NMR ($CDCl_3$, 600 MHz) δ 8.05 (1H, s), 7.67 (1H, t, J = 2.0 Hz), 7.59 (1H, d, J = 8.1 Hz), 7.42 (1H, t, J = 8.0 Hz), 7.34 (1H, d, J = 4.9 Hz), 2.52 – 2.46 (2H, m), 2.12 – 2.01 (4H, m), 1.99 – 1.93 (2H, m); ^{13}C NMR ($CDCl_3$, 151 MHz) δ 154.9 (q, J = 37.7 Hz), 141.6, 135.9, 130.1, 124.1, 124.0, 120.0, 118.3, 115.8 (q, J = 288.9 Hz), 47.9, 40.7, 24.4; ^{19}F NMR ($CDCl_3$, 564 MHz) δ -75.71. HRMS (ESI): found M^+ 282.0973, $C_{14}H_{13}F_3N_2O$ requires 282.0975.

2,2,2-Trifluoro-*N*-(3-(methoxymethyl)phenyl)acetamide (5h)

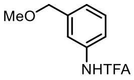

Following **GP2**, *N*-(2-((3',5'-dimethoxy-[1,1'-biphenyl]-2-yl)thio)-5-(methoxymethyl)phenyl)-2,2,2-trifluoroacetamide (47.8 mg, 0.24 mmol, 2.4 eq.) gave the title compound (89%) as an oil. ^{1}H NMR ($CDCl_3$, 600 MHz) δ 8.45 (1H, s), 7.53 – 7.48 (2H, m), 7.33 (1H, t, J = 7.8 Hz), 7.18 (1H, d, J = 7.6 Hz), 4.45 (2H, s), 3.41 (3H, s); ^{13}C NMR ($CDCl_3$, 151 MHz) δ 155.1 (q, J = 37.4 Hz), 139.4, 135.5, 129.4, 125.6, 120.2, 120.0, 115.9 (q, J = 288.5 Hz), 74.2, 58.3; ^{19}F NMR ($CDCl_3$, 564 MHz) -75.80 δ. HRMS (ESI): found M^+ 233.0659, $C_{10}H_{10}F_3NO_2$ requires 233.0658.

***N*-(3-(2-(benzyloxy)-1,1,1,3,3,3-hexafluoropropan-2-yl)phenyl)-2,2,2-trifluoroacetamide (5i)**

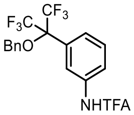

Following **GP2**, *N*-(5-(2-(benzyloxy)-1,1,1,3,3,3-hexafluoropropan-2-yl)-2-((3',5'-dimethoxy-[1,1'-biphenyl]-2-yl)thio)phenyl)-2,2,2-trifluoroacetamide (47.8 mg, 0.24 mmol, 2.4 eq.) gave the title compound (73%) as an oil. ^{1}H NMR ($CDCl_3$, 600 MHz) δ 7.91 (1H, s), 7.88 – 7.80 (1H, m), 7.73 (1H, s), 7.53 (2H, d, J = 5.1 Hz), 7.45 – 7.33 (5H, m), 4.68 (2H, s); ^{13}C NMR ($CDCl_3$, 151 MHz) δ 136.1, 135.8, 130.2, 130.0, 128.8, 128.5, 127.6, 126.2, 122.7, 122.4 (q, J = 286.9 Hz), 120.5, 68.6; ^{19}F NMR ($CDCl_3$, 564 MHz) δ -70.57, -75.69. HRMS (ESI): found MNa^+ 468.0609, $C_{18}H_{12}F_9NO_2Na$ requires 468.0616.

2,2,2-Trifluoro-*N*-(3-(hydroxydiphenylmethyl)phenyl)acetamide (5j)

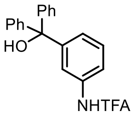

Following **GP2**, *N*-(2-((3',5'-dimethoxy-[1,1'-biphenyl]-2-yl)thio)-5-(hydroxydiphenylmethyl)phenyl)-2,2,2-trifluoroacetamide (47.8 mg, 0.24 mmol, 2.4 eq.) gave the title compound (97%) as an oil. ^{1}H NMR ($CDCl_3$, 600 MHz) δ 7.84 (1H, s), 7.66 (1H, d, J = 5.9 Hz), 7.36 – 7.18 (12H, m), 7.10 (1H, d, J = 7.9 Hz); ^{13}C NMR ($CDCl_3$, 151 MHz) δ 154.9 (q, J = 37.4 Hz), 148.5, 146.4, 135.0, 129.1, 128.3, 128.0, 127.7, 126.1, 119.9, 119.6, 115.8 (q, J = 288.8

Hz), 81.9; ^{19}F NMR ($CDCl_3$, 564 MHz) δ -75.77. HRMS (ESI): found MNa^+ 394.1028, $C_{21}H_{16}F_3NO_2Na$ requires 394.1030.

2,2,2-Trifluoro-*N*-(3-(4,4,5,5-tetramethyl-1,3,2-dioxaborolan-2-yl)phenyl)acetamide (5k)

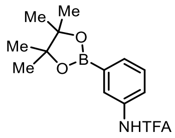

Following **GP2**, 2,4-dimethoxy-5-(4-(4,4,5,5-tetramethyl-1,3,2-dioxaborolan-2-yl)-2-(2,2,2-trifluoroacetamido)phenyl)-5*H*-dibenzo[b,d]thiophen-5-ium trifluoromethanesulfonate (47.8 mg, 0.24 mmol, 2.4 eq.) gave the title compound (98%) as an oil. ^1H NMR ($CDCl_3$, 600 MHz) δ 7.90 (1H, d, J = 10.8 Hz), 7.84 (1H, s), 7.73 (1H, s), 7.67 (1H, d, J = 7.3 Hz), 7.42 (1H, t, J = 7.7 Hz), 1.35 (12H, s); ^{13}C NMR ($CDCl_3$, 151 MHz) δ 154.9 (q, J = 37.2 Hz), 134.7, 132.7, 129.1, 126.5, 123.5, 115.9 (q, J = 289.2 Hz), 84.4, 25.0; ^{19}F NMR ($CDCl_3$, 564 MHz) δ -75.87. HRMS (ESI): found M^+ 315.1245, $C_{14}H_{17}BF_3NO_3$ requires 315.1249.

2,2,2-Trifluoro-*N*-(2',3',4',5'-tetrahydro-[1,1'-biphenyl]-3-yl)acetamide (5l)

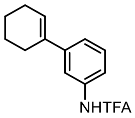

Following **GP2**, *N*-(4-((3',5'-dimethoxy-[1,1'-biphenyl]-2-yl)thio)-2',3',4',5'-tetrahydro-[1,1'-biphenyl]-3-yl)-2,2,2-trifluoroacetamide (47.8 mg, 0.24 mmol, 2.4 eq.) gave the title compound (75%) as an oil. ^1H NMR ($CDCl_3$, 600 MHz) δ 7.91 (1H, s), 7.55 (1H, s), 7.43 (1H, d, J = 8.1 Hz), 7.32 (1H, t, J = 7.9 Hz), 7.26 (1H, d, J = 8.0 Hz), 6.18 – 6.13 (1H, m), 2.41 – 2.34 (2H, m), 2.25 – 2.17 (2H, m), 1.83 – 1.75 (2H, m), 1.69 – 1.60 (2H, m); ^{13}C NMR ($CDCl_3$, 151 MHz) δ 154.9 (q, J = 37.4 Hz), 144.3, 135.8, 135.1, 129.3, 126.3, 123.2, 118.7, 117.3, 115.9 (q, J = 288.9 Hz), 27.5, 26.0, 23.1, 22.1; ^{19}F NMR ($CDCl_3$, 564 MHz) δ -75.78. HRMS (ESI): found MNa^+ 292.0911, $C_{14}H_{14}F_3NONa$ requires 292.0925.

***N*-([1,1'-biphenyl]-3-yl)-2,2,2-trifluoroacetamide (5m)**

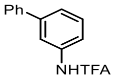

Following **GP2**, *N*-(4-((3',5'-dimethoxy-[1,1'-biphenyl]-2-yl)thio)-[1,1'-biphenyl]-3-yl)-2,2,2-trifluoroacetamide (47.8 mg, 0.24 mmol, 2.4 eq.) gave the title compound (97%) as an oil. ^{1}H NMR (CDCl$_3$, 600 MHz) δ 7.95 (1H, s), 7.80 (1H, s), 7.59 (2H, d, *J* = 8.1 Hz), 7.58 – 7.54 (1H, m), 7.50 – 7.43 (4H, m), 7.38 (1H, t, *J* = 6.9 Hz); ^{13}C NMR (CDCl$_3$, 151 MHz) δ 154.7 (q, *J* = 37.6 Hz), 142.8, 140.1, 135.7, 129.9, 129.0, 128.0, 127.3, 125.3, 119.3, 118.0 (q, *J* = 362.4 Hz) ; ^{19}F NMR (CDCl$_3$, 564 MHz) δ -75.71. HRMS (ESI): found M$^+$ 265.0709, $C_{14}H_{10}F_3NO$ requires 265.0709.

2,2,2-Trifluoro-*N*-(4'-methoxy-[1,1'-biphenyl]-3-yl)acetamide (5n)

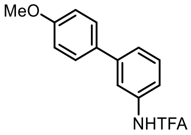

Following **GP2**, *N*-(4-((3',5'-dimethoxy-[1,1'-biphenyl]-2-yl)thio)-4'-methoxy-[1,1'-biphenyl]-3-yl)-2,2,2-trifluoroacetamide (47.8 mg, 0.24 mmol, 2.4 eq.) gave the title compound (97%) as an oil. ^{1}H NMR (CDCl$_3$, 600 MHz) δ 7.94 (1H, s), 7.76 (1H, s), 7.52 (2H, d, *J* = 8.7 Hz), 7.51 – 7.48 (1H, m), 7.46 – 7.41 (2H, m), 6.98 (2H, d, *J* = 8.7 Hz), 3.86 (3H, s); ^{13}C NMR (CDCl$_3$, 151 MHz) δ 159.7, 154.9 (q, *J* = 37.3 Hz), 142.4, 135.6, 132.6, 129.9, 128.4, 124.9, 118.9, 118.7, 115.9 (q, *J* = 288.5 Hz), 114.5, 55.5; ^{19}F NMR (CDCl$_3$, 564 MHz) δ -76.1. HRMS (ESI): found MNa$^+$ 318.0705, $C_{15}H_{12}F_3NO_2Na$ requires 318.0712.

2,2,2-Trifluoro-*N*-(4'-iodo-[1,1'-biphenyl]-3-yl)acetamide (5o)

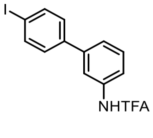

Following **GP2**, *N*-(4-((3',5'-dimethoxy-[1,1'-biphenyl]-2-yl)thio)-4'-iodo-[1,1'-biphenyl]-3-yl)-2,2,2-trifluoroacetamide (47.8 mg, 0.24 mmol, 2.4 eq.) gave the title compound (98%) as an oil. ^{1}H NMR (CDCl$_3$, 600 MHz) δ 8.00 (1H, s), 7.80 (1H, s), 7.58 (2H, d, *J* = 7.1 Hz), 7.56 (1H, dt, *J* = 6.8, 2.2 Hz), 7.49 – 7.43 (3H, m), 7.38 (1H, t, *J* = 7.4 Hz); ^{13}C NMR (CDCl$_3$, 151 MHz) δ 155.0 (q, *J* = 37.3 Hz), 142.8, 140.1, 135.7, 129.9, 129.0, 128.0, 127.3, 125.3, 120.9, 119.4, 115.9 (q, *J*

= 288.7 Hz); ^{19}F NMR ($CDCl_3$, 564 MHz) δ -75.71. HRMS (ESI): found MNa^+ 413.9573, $C_{14}H_9F_3INONa$ requires 413.9579.

2,2,2-Trifluoro-*N*-(3'-fluoro-[1,1'-biphenyl]-3-yl)acetamide (5p)

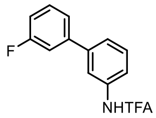

Following **GP2**, *N*-(4-((3',5'-dimethoxy-[1,1'-biphenyl]-2-yl)thio)-3'-fluoro-[1,1'-biphenyl]-3-yl)-2,2,2-trifluoroacetamide (47.8 mg, 0.24 mmol, 2.4 eq.) gave the title compound (95%) as an oil. ^{1}H NMR ($CDCl_3$, 600 MHz) δ 7.94 (1H, s), 7.79 (1H, s), 7.57 – 7.49 (3H, m), 7.46 (1H, t, J = 7.8 Hz), 7.42 (1H, d, J = 7.6 Hz), 7.14 (2H, t, J = 8.6 Hz); ^{13}C NMR ($CDCl_3$, 151 MHz) δ 162.9 (d, J = 247.5 Hz), 155.0 (q, J = 37.4 Hz), 141.9, 136.3, 135.7, 130.0, 129.0, 128.9, 125.2, 119.3, 119.2, 116.0, 115.9, 115.6 (q, J = 289.9 Hz); ^{19}F NMR ($CDCl_3$, 564 MHz) δ -75.72, -114.71. HRMS (ESI): found M^+ 283.0613, $C_{14}H_9F_4NO$ requires 283.0615.

2,2,2-Trifluoro-*N*-(2-methoxyphenyl)acetamide (5q′)

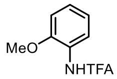

Following **GP2**, *N*-(2-((3',5'-dimethoxy-[1,1'-biphenyl]-2-yl)thio)-6-methoxyphenyl)-2,2,2-trifluoroacetamide (47.8 mg, 0.24 mmol, 2.4 eq.) gave the title compound (96%) as an oil. ^{1}H NMR ($CDCl_3$, 600 MHz) δ 8.57 (1H, s), 8.32 (1H, dd, J = 8.0, 1.6 Hz), 7.17 (1H, td, J = 7.9, 1.6 Hz), 7.01 (1H, td, J = 7.8, 1.3 Hz), 6.94 (1H, dd, J = 8.3, 1.3 Hz), 3.93 (3H, s); ^{13}C NMR ($CDCl_3$, 151 MHz) δ 154.5 (q, J = 37.2 Hz), 148.4, 126.1, 125.2, 121.4, 120.3, 115.9 (q, J = 288.6 Hz), 110.4, 56.0; ^{19}F NMR ($CDCl_3$, 564 MHz) δ -75.84. HRMS (ESI): found M^+ 219.0501, $C_9H_8F_3NO_2$ requires 219.0507.

***N*-(4-(*tert*-butyl)phenyl)-2,2,2-trifluoroacetamide (5r)**

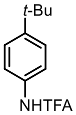

Following **GP2**, *N*-(4-(*tert*-butyl)-2-((3',5'-dimethoxy-[1,1'-biphenyl]-2-yl)thio)phenyl)-2,2,2-trifluoroacetamide (47.8 mg, 0.24 mmol, 2.4 eq.) gave the title compound (92%) as an oil. ^{1}H

NMR ($CDCl_3$, 600 MHz) δ 8.02 (1H, s), 7.49 (2H, d, J = 7.2 Hz), 7.40 (2H, d, J =7.4 Hz), 1.32 (9H, s); ^{13}C NMR ($CDCl_3$, 151 MHz) δ 155.0 (q, J = 37.1 Hz), 149.7, 132.6, 126.3, 120.5, 115.9 (q, J = 288.6 Hz), 34.7, 31.4; ^{19}F NMR ($CDCl_3$, 564 MHz) δ -75.73. HRMS (ESI): found M^+ 245.1021, $C_{12}H_{14}F_3NO$ requires 245.1022.

***N*-(2-(*tert*-butyl)phenyl)-2,2,2-trifluoroacetamide (5r′)**

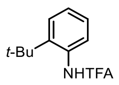

Following **GP2**, *N*-(2-(*tert*-butyl)-6-((3',5'-dimethoxy-[1,1'-biphenyl]-2-yl)thio)phenyl)-2,2,2-trifluoroacetamide (47.8 mg, 0.24 mmol, 2.4 eq.) gave the title compound (92%) as an oil. ^{1}H NMR ($CDCl_3$, 600 MHz) δ 8.01 (1H, s), 7.67 (1H, d, J = 7.7 Hz), 7.50 – 7.41 (1H, m), 7.29 (1H, td, J = 7.6, 1.8 Hz), 7.29 – 7.23 (1H, m), 1.43 (9H, s); ^{13}C NMR ($CDCl_3$, 151 MHz) δ 155.2 (q, J = 39.2) 142.8, 132.5, 127.8, 127.4, 127.2, 126.8, 116.3 (q, J = 289.9) 34.6, 30.8; ^{19}F NMR ($CDCl_3$, 564 MHz) δ -75.95. HRMS (ESI): found M^+ 245.1021, $C_{12}H_{14}F_3NO$ requires 245.1022.

***N*-(2,4-dimethylphenyl)-2,2,2-trifluoroacetamide (5s)**

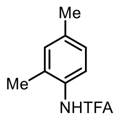

Following **GP2**, *N*-(2-((3',5'-dimethoxy-[1,1'-biphenyl]-2-yl)thio)-4,6-dimethylphenyl)-2,2,2-trifluoroacetamide (47.8 mg, 0.24 mmol, 2.4 eq.) gave the title compound (95%) as an oil. ^{1}H NMR ($CDCl_3$, 600 MHz) δ 7.63 (1H, s), 7.60 (1H, d, J = 6.0 Hz), 7.07 (1H, d, J = 6.5 Hz), 7.06 (1H, s), 2.32 (3H, s), 2.25 (3H, s); ^{13}C NMR ($CDCl_3$, 151 MHz) δ 137.2, 131.7, 130.3, 130.2, 127.8, 123.5, 116.0 (q, J = 286.9), 21.1, 17.5; ^{19}F NMR ($CDCl_3$, 564 MHz) δ -75.63. HRMS (ESI): found MNa^+ 240.0603, $C_{10}H_{10}F_3NONa$ requires 240.0607.

***N*-(2,4-dimethoxyphenyl)-2,2,2-trifluoroacetamide (5t)**

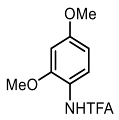

Following **GP2**, *N*-(2-((3',5'-dimethoxy-[1,1'-biphenyl]-2-yl)thio)-4,6-dimethoxyphenyl)-2,2,2-trifluoroacetamide (47.8 mg, 0.24 mmol, 2.4 eq.) gave the title compound (75%) as an oil. ^{1}H NMR ($CDCl_3$, 600 MHz) δ 8.37 (1H, s), 8.19 (1H, d, J = 9.2 Hz), 6.51 – 6.49 (2H, m), 3.89 (3H,

s), 3.81 (3H, s); ^{13}C NMR ($CDCl_3$, 151 MHz) δ 158.1, 154.1 (q, J = 37.0 Hz), 149.8, 121.2, 118.6, 116.0 (q, J = 288.2 Hz), 104.1, 98.8, 56.0, 55.7; ^{19}F NMR ($CDCl_3$, 564 MHz) δ -75.75. HRMS (ESI): found M$^+$ 249.0605, $C_{10}H_{10}F_3NO_3$ requires 249.0607.

2,2,2-Trifluoro-*N*-(5,6,7,8-tetrahydronaphthalen-2-yl)acetamide (5u)

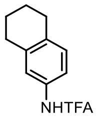

Following **GP2**, *N*-(3-((3',5'-dimethoxy-[1,1'-biphenyl]-2-yl)thio)-5,6,7,8-tetrahydronaphthalen-2-yl)-2,2,2-trifluoroacetamide (47.8 mg, 0.24 mmol, 2.4 eq.) gave the title compound (87%) as an oil. ^{1}H NMR ($CDCl_3$, 600 MHz) δ 7.85 (1H, s), 7.28 (1H, s), 7.24 (1H, dd, J = 8.2, 2.4 Hz), 7.06 (1H, d, J = 8.2 Hz), 2.77 – 2.71 (4H, m), 1.88 – 1.73 (4H, m); ^{13}C NMR ($CDCl_3$, 151 MHz) δ 154.8 (q, J = 37.0 Hz), 138.5, 135.8, 132.4, 130.0, 121.2, 118.0, 115.9 (q, J = 288.7 Hz), 29.6, 29.1, 23.1, 23.0; ^{19}F NMR ($CDCl_3$, 564 MHz) δ -75.75. HRMS (ESI): found MNa$^+$ 266.0759, $C_{12}H_{12}F_3NONa$ requires 266.0763.

2,2,2-Trifluoro-*N*-(5,6,7,8-tetrahydronaphthalen-1-yl)acetamide (5u′)

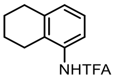

Following **GP2**, *N*-(2-((3',5'-dimethoxy-[1,1'-biphenyl]-2-yl)thio)-5,6,7,8-tetrahydronaphthalen-1-yl)-2,2,2-trifluoroacetamide (47.8 mg, 0.24 mmol, 2.4 eq.) gave the title compound (87%) as an oil. ^{1}H NMR ($CDCl_3$, 600 MHz) δ 7.77 (1H, s), 7.56 (1H, d, J = 7.9 Hz), 7.15 (1H, t, J = 7.8 Hz), 7.03 (1H, d, J = 7.7 Hz), 2.80 (2H, t, J = 6.3 Hz), 2.60 (2H, t, J = 6.4 Hz), 1.90 – 1.83 (2H, m), 1.82 – 1.72 (2H, m); ^{13}C NMR ($CDCl_3$, 151 MHz) δ 155.2 (q, J = 36.9 Hz), 138.8, 132.6, 129.3, 128.3, 126.2, 120.9, 116.1 (q, J = 289.0 Hz), 29.8, 24.3, 22.7, 22.5; ^{19}F NMR ($CDCl_3$, 564 MHz) δ -75.66. HRMS (ESI): found MNa$^+$ 266.0757, $C_{12}H_{12}F_3NONa$ requires 266.0763.

N-(2,6-dimethyl-[1,1'-biphenyl]-3-yl)-2,2,2-trifluoroacetamide (5v)

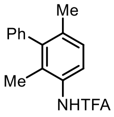

Following **GP2**, *N*-(4-((3',5'-dimethoxy-[1,1'-biphenyl]-2-yl)thio)-2,6-dimethyl-[1,1'-biphenyl]-3-yl)-2,2,2-trifluoroacetamide (47.8 mg, 0.24 mmol, 2.4 eq.) gave the title compound (88%) as an oil. ^{1}H NMR ($CDCl_3$, 600 MHz) δ 7.73 (1H, s), 7.60 (1H, d, *J* = 8.2 Hz), 7.45 (2H, t, *J* = 7.4 Hz), 7.37 (1H, t, *J* = 7.5 Hz), 7.18 (1H, d, *J* = 8.3 Hz), 7.11 (2H, d, *J* = 6.8 Hz), 2.02 (3H, s), 1.94 (3H, s); ^{13}C NMR ($CDCl_3$, 151 MHz) δ 155.4 (q, *J* = 36.9 Hz), 143.2, 140.4, 135.8, 130.6, 129.1, 128.8, 128.1, 127.3, 122.9, 116.2 (q, *J* = 289.0 Hz), 21.1, 15.3; ^{19}F NMR ($CDCl_3$, 564 MHz) δ -75.60. HRMS (ESI): found M^+ 293.1019, $C_{16}H_{14}F_3NO$ requires 293.1022.

Butyl 3-(2,2,2-trifluoroacetamido)benzoate (5w)

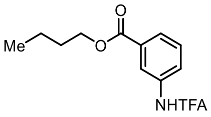

Following **GP2**, Butyl 4-((3',5'-dimethoxy-[1,1'-biphenyl]-2-yl)thio)-3-(2,2,2-trifluoroacetamido)benzoate (47.8 mg, 0.24 mmol, 2.4 eq.) gave the title compound (95%) as an oil. ^{1}H NMR ($CDCl_3$, 600 MHz) δ 8.53 (1H, s), 8.14 (1H, s), 8.00 (1H, d, *J* = 8.1 Hz), 7.90 (1H, d, *J* = 7.8 Hz), 7.48 (1H, t, *J* = 8.0 Hz), 4.32 (2H, t, *J* = 6.6 Hz), 1.74 (2H, qi, *J* = 6.8 Hz), 1.46 (2H, sx, *J* = 7.4 Hz), 0.97 (3H, t, *J* = 7.4 Hz); ^{13}C NMR ($CDCl_3$, 151 MHz) δ 166.2, 155.3 (q, *J* = 37.7 Hz), 135.7, 131.7, 129.7, 127.3, 125.1, 121.7, 115.8 (q, *J* = 288.7 Hz), 65.5, 30.8, 19.3, 13.8; ^{19}F NMR ($CDCl_3$, 564 MHz) δ -75.65. HRMS (ESI): found MNa^+ 312.0812, $C_{13}H_{14}F_3NO_3Na$ requires 312.0818.

N-(3-(3-ethyl-2,6-dioxopiperidin-3-yl)phenyl)-2,2,2-trifluoroacetamide (5x)

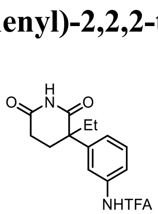

Following **GP2**, *N*-(2-((3',5'-dimethoxy-[1,1'-biphenyl]-2-yl)thio)-5-(3-ethyl-2,6-dioxopiperidin-3-yl)phenyl)-2,2,2-trifluoroacetamide (47.8 mg, 0.24 mmol, 2.4 eq.) gave the title compound (98%) as an oil. ^{1}H NMR ($CDCl_3$, 600 MHz) δ 8.07 (1H, s), 8.03 (1H, s), 7.61 (1H, d, *J* = 8.0 Hz),

7.51 (1H, s), 7.42 (1H, t, J = 8.0 Hz), 7.17 (1H, d, J = 7.9 Hz), 2.64 (1H, d, J = 17.2 Hz), 2.41 (2H, d, J = 14.2 Hz), 2.27 (1H, d, J = 13.5 Hz), 2.11 – 1.91 (2H, m), 0.88 (3H, t, J = 7.4 Hz); ^{13}C NMR ($CDCl_3$, 151 MHz) δ 174.9, 172.0, 140.6, 136.2, 130.3, 124.2, 119.9, 118.5, 51.3, 33.1, 29.4, 27.0, 9.2; ^{19}F NMR ($CDCl_3$, 564 MHz) δ -75.70. HRMS (ESI): found MNa^+ 351.0922, $C_{15}H_{15}F_3N_2O_3Na$ requires 351.0927.

2,2,2-Trifluoro-*N*-((8*R*,9*S*,13*S*,14*S*)-13-methyl-17-oxo-7,8,9,11,12,13,14,15,16,17-decahydro-6*H*-cyclopenta[a]phenanthren-2-yl)acetamide (5y)

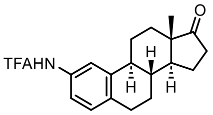

Following **GP2**, *N*-((8*R*,9*S*,13*S*,14*S*)-3-((3',5'-dimethoxy-[1,1'-biphenyl]-2-yl)thio)-13-methyl-17-oxo-7,8,9,11,12,13,14,15,16,17-decahydro-6*H*-cyclopenta[a]phenanthren-2-yl)-2,2,2-trifluoroacetamide (47.8 mg, 0.24 mmol, 2.4 eq.) gave the title compound (95%) as an oil. ^{1}H NMR ($CDCl_3$, 600 MHz) δ 7.76 (1H, s), 7.48 (1H, s), 7.32 (1H, d, J = 8.3 Hz), 7.12 (1H, d, J = 8.2 Hz), 2.99 – 2.85 (2H, m), 2.51 (1H, dd, J = 19.1, 8.8 Hz), 2.44 – 2.37 (1H, m), 2.31 (1H, t, J = 11.3 Hz), 2.19 – 2.11 (1H, m), 2.08 – 2.01 (2H, m), 1.98 (1H, d, J = 12.5 Hz), 1.71 – 1.56 (3H, m), 1.53 – 1.40 (3H, m), 0.88 (3H, t, J = 7.2 Hz); ^{13}C NMR ($CDCl_3$, 151 MHz) δ 220.8, 141.2, 135.1, 132.9, 130.0, 118.4, 117.9, 118.4, (q, J = 332.2 Hz), 50.6, 46.1, 44.5, 38.0, 36.0, 31.6, 29.0, 26.5, 25.9, 21.7, 14.0; ^{19}F NMR ($CDCl_3$, 564 MHz) δ -75.74. HRMS (ESI): found MNa^+ 388.1499, $C_{20}H_{22}F_3NO_2Na$ requires 388.1500.

5-(3,5-Dimethyl-2-(2,2,2-trifluoroacetamido)phenyl)-2,4-dimethoxy-5*H*-dibenzo[*b,d*]thiophen-5-ium trifluoromethanesulfonate (4qh)

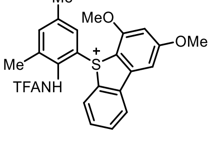

Following **GP2a**, N-(2-((3',5'-dimethoxy-[1,1'-biphenyl]-2-yl)thio)-5-(3-ethyl-2,6-dioxopiperidin-3-yl)phenyl)-2,2,2-trifluoroacetamide (50 mg, 0.11 mmol, 1 eq.) gave the title compound (71%) as a brown solid. ^{1}H NMR ($MeOD_4$, 600 MHz) δ 8.43 (1H, d, J = 7.4 Hz), 8.22 (1H, s), 7.96 (1H, t, J = 7.6 Hz), 7.75 (2H, t, J = 7.8 Hz), 7.60 (1H, d, J = 2.0 Hz), 7.54 (1H, s), 6.85 (1H, d, J = 2.0 Hz), 6.47 (1H, s), 4.04 (3H, s), 3.91 (3H, s), 2.39 (3H, s), 2.15 (3H, s); ^{13}C

NMR ($CDCl_3$, 151 MHz) δ 169.5, 159.9, 143.8, 143.5, 141.1, 139.6, 135.4, 134.7, 132.9, 128.7, 126.3, 122.8, 120.7, 118.5, 116.6, 103.1, 101.7, 57.7, 57.3, 20.8, 17.9; ^{19}F NMR ($CDCl_3$, 564 MHz) δ -76.49, -80.14. HRMS (ESI): found [M-OTf]$^+$ 460.1181, $C_{24}H_{21}F_3NO_3S$ requires 460.1189.

***N*-(2-chloro-4,6-dimethylphenyl)-2,2,2-trifluoroacetamide (6a)**

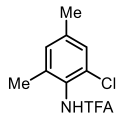

Following a known procedure[15], 5-(3,5-dimethyl-2-(2,2,2-trifluoroacetamido)phenyl)-2,4-dimethoxy-5*H*-dibenzo[*b,d*]thiophen-5-ium trifluoromethanesulfonate (100 mg, 0.16 mmol, 1 eq.) gave the title compound (78%) as an oil. ^{1}H NMR ($CDCl_3$, 600 MHz) δ 7.58 (1H, s), 7.14 (1H, s), 7.01 (1H, s), 2.32 (3H, s), 2.24 (3H, s); ^{13}C NMR ($CDCl_3$, 151 MHz) δ 155.6 (q, J = 37.8 Hz), 139.9, 137.5, 131.0, 130.5, 128.0, 126.8, 21.1, 18.6; ^{19}F NMR ($CDCl_3$, 564 MHz) δ -75.38. HRMS (ESI): found MNa$^+$ 274.0212, $C_{10}H_9ClF_3NONa$ requires 274.0217.

***N*-(2,4-dimethyl-6-(4,4,5,5-tetramethyl-1,3,2-dioxaborolan-2-yl)phenyl)-2,2,2-trifluoroacetamide (6b)**

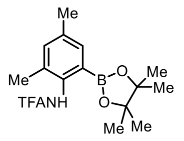

Following a known procedure[15], 5-(3,5-dimethyl-2-(2,2,2-trifluoroacetamido)phenyl)-2,4-dimethoxy-5*H*-dibenzo[*b,d*]thiophen-5-ium trifluoromethanesulfonate (100 mg, 0.16 mmol, 1 eq.) gave the title compound (75%) as an oil. ^{1}H NMR ($CDCl_3$, 600 MHz) δ 8.76 (1H, s), 7.48 (1H, s), 7.19 (1H, s), 2.32 (3H, s), 2.22 (3H, s), 1.33 (12H, s); ^{13}C NMR ($CDCl_3$, 151 MHz) δ 155.5 (q, J = 36.4 Hz), 137.0, 135.7, 135.4, 134.6, 134.2, 116.4 (q, J = 289.0 Hz), 84.5, 25.0, 20.9, 18.6; ^{19}F NMR ($CDCl_3$, 564 MHz) δ -75.67. HRMS (ESI): found MNa$^+$ 366.1455, $C_{16}H_{21}BF_3NO_3Na$ requires 366.1459.

N-(2,4-dimethyl-6-(1-methyl-1H-pyrrol-2-yl)phenyl)-2,2,2-trifluoroacetamide (6c)

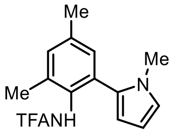

Following a known procedure[16], 5-(3,5-dimethyl-2-(2,2,2-trifluoroacetamido)phenyl)-2,4-dimethoxy-5*H*-dibenzo[*b,d*]thiophen-5-ium trifluoromethanesulfonate (100 mg, 0.16 mmol, 1 eq.) gave the title compound (71%) as an oil. ^{1}H NMR ($CDCl_3$, 600 MHz) δ 7.12 (1H, s), 7.01 (1H, s), 6.72 (1H, t, J = 2.1 Hz), 6.18 (1H, t, J = 2.8 Hz), 6.03 (1H, dd, J = 3.7, 1.6 Hz), 3.39 (3H, s), 2.35 (3H, s), 2.25 (3H, s); ^{13}C NMR ($CDCl_3$, 151 MHz) δ 155.5 (q, J = 36.5 Hz), 138.0, 135.6, 131.9, 130.3, 130.1, 129.2, 129.2, 123.4, 116.0 (q, J = 288.7 Hz), 109.2, 108.0, 34.4, 21.1, 18.6; ^{19}F NMR ($CDCl_3$, 564 MHz) δ -75.60. HRMS (ESI): found MNa^+ 319.1023, $C_{15}H_{15}F_3N_2ONa$ requires 319.1029.

N-(2-(2,2-diphenylvinyl)-4,6-dimethylphenyl)-2,2,2-trifluoroacetamide (6d)

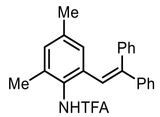

Following a known procedure[16], 5-(3,5-dimethyl-2-(2,2,2-trifluoroacetamido)phenyl)-2,4-dimethoxy-5*H*-dibenzo[*b,d*]thiophen-5-ium trifluoromethanesulfonate (100 mg, 0.16 mmol, 1 eq.) gave the title compound (69%) as an oil. ^{1}H NMR ($CDCl_3$, 600 MHz) δ 7.34 – 7.32 (3H, m), 7.33 – 7.28 (2H, m), 7.28 – 7.23 (3H, m), 7.09 (2H, dd, J = 7.9, 1.7 Hz), 6.96 (1H, s), 6.93 (1H, s), 6.85 (1H, s), 6.81 (1H, s), 2.20 (3H, s), 2.08 (3H, s); ^{13}C NMR ($CDCl_3$, 151 MHz) δ 155.6 (q, J = 36.6 Hz), 145.4, 142.8, 139.7, 138.1, 135.4, 135.3, 130.7, 130.4, 129.3, 128.6, 128.4, 128.4, 128.1, 126.9, 126.2, 124.4, 116.1 (q, J = 288.5 Hz), 21.1, 18.2; ^{19}F NMR ($CDCl_3$, 564 MHz) δ -75.17. HRMS (ESI): found MNa^+ 418.1390, $C_{24}H_{20}F_3NONa$ requires 418.1395.

***N*-(3,5-dimethyl-4'-(trifluoromethyl)-[1,1'-biphenyl]-2-yl)-2,2,2-trifluoroacetamide (6e)**

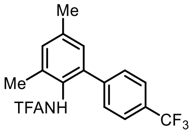

Following a known procedure[17], 5-(3,5-dimethyl-2-(2,2,2-trifluoroacetamido)phenyl)-2,4-dimethoxy-5*H*-dibenzo[*b,d*]thiophen-5-ium trifluoromethanesulfonate (100 mg, 0.16 mmol, 1 eq.) gave the title compound (69%) as an oil. ^{1}H NMR ($CDCl_3$, 600 MHz) δ 7.75 (1H, s), 7.39 (2H, d, *J* = 8.5 Hz), 7.18 (2H, d, *J* = 8.0 Hz), 6.80 (1H, s), 6.26 (1H, s), 2.10 (3H, s), 2.08 (3H, s); ^{13}C NMR ($CDCl_3$, 151 MHz) δ 155.2 (q, *J* = 36.8 Hz), 148.6, 144.8, 139.5, 137.9, 135.3, 133.9, 128.7 (q, *J* = 32.1 Hz), 128.2, 127.4, 124.8 (q, *J* = 3.7 Hz), 124.4 (q, *J* = 271.8 Hz), 116.1 (q, *J* = 288.7 Hz), 21.1, 18.5; ^{19}F NMR ($CDCl_3$, 564 MHz) δ -62.37, -75.23. HRMS (ESI): found MNa^+ 384.0805, $C_{17}H_{13}F_6NONa$ requires 384.0799.

Characterized as a mixture with inseparable byproduct : **2,4-Dimethoxydibenzo[*b,d*]thiophene 5-oxide (6e´)**

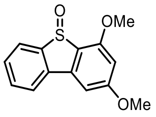

12. Failed Substrates

12.1. Unable to undergo ring expansion to azepane

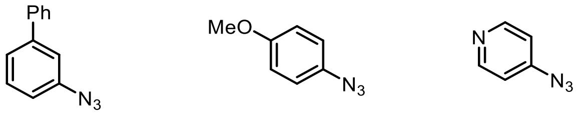

12.2. Unable to undergo TFAA-mediated ring contraction from azepane

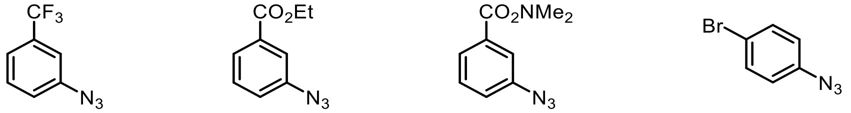

13. Mechanistical analysis

Computational Details

To investigate the temperature-dependent product distribution of **1q** and **1r** substrates, DFT[18] calculations were carried out. The singlet nitrene (**II**), azirine (**III**), and ketimine (**IV**) species were considered, along with the transition states connecting them (**II→III**, step b; **III→IV**, step c). These calculations were conducted along both *path a* and *path b* (**Scheme 1**).

Figure 1. Studied reaction mechanism

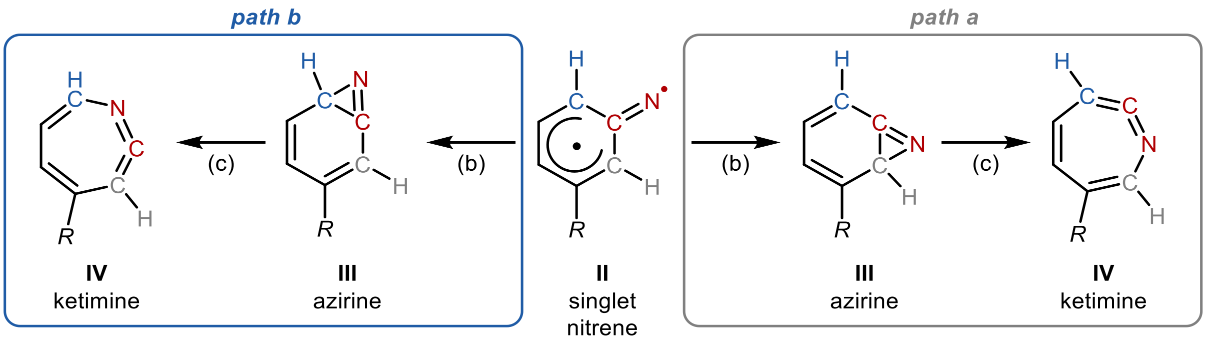

All calculations were performed with Gaussian 16 C.01 software package[19]. Geometry optimizations were carried out using the unrestricted formalism of M06-2X functional[20] in combination with the def2-TZVP basis set[21], as implemented in Gaussian 16. The same level of theory was employed for vibrational frequency analyses to confirm that no negative eigenvalues in the Hessian matrix, with only one negative eigenvalue for transition states. No symmetry restrictions were applied.

Further single-point energy calculations were performed at the UM06-2X/def2-QZVPP[22] level of theory both in the gas phase and with three different solvents: 1,4-dioxane ($\varepsilon = 2.2099$), THF ($\varepsilon = 7.4257$), and TFT ($\varepsilon = 9.40$), using the implicit solvation model SMD[23]. Since Gaussian 16 does not provide specific solvent parameters for TFT, the parameters of toluene were employed, with the following adjustments: dielectric constant ($\varepsilon = 9.40$), the square of the index of refraction at optical frequencies ($n^2 = 2.00$), and electronegative halogenicity parameter (ElectronegativeHalogenicity = 0.500).

Thermodynamic data were obtained via quasi-harmonic corrections to entropy using the rigid rotor/harmonic oscillator (RRHO) approximation[24], in which vibrational modes below 100 cm^{-1}

were treated with a free rotor approximation interpolated with a damping function. These corrections were applied with the *Goodvibes.py* script, assuming a solution-phase standard state (c = 1 mol-1). In addition, Gibbs free energies were corrected for temperature effects between 298 K and 333 K in 5 K increments.

All energy values are reported in kcal mol^{-1}. Cartesian coordinates and thermodynamic data are provided in the electronic supplementary material.

Electronic Structure of Singlet Nitrene

The singlet nitrene intermediates (**II**) were found to exhibit an open-shell singlet character. Calculations were performed within the unrestricted formalism, and spin contamination was observed, with $\langle S^2 \rangle$ value of 1.03. This significant deviation from the ideal singlet value indicates a pronounced open-shell character. Mulliken spin density analysis consistently reveals that the nitrene nitrogen atom carries the highest spin population (0.480-0.517), confirming its central role as the reactive site. In addition, the aromatic π-framework accommodates substantial delocalization, with spin densities on the ring carbons spanning a wide range (approximately -050 to +0.45). By contrast, the substituents (–OMe and –*t*-Bu) exhibit spin densities close to zero, indicating that they do not contribute significantly to the electronic structure. Taken together, these results highlight the dual character of the intermediate: a highly localized radical centre at nitrogen, electronically stabilized by extensive delocalization within the aromatic π-system.

The total spin density distribution is shown in **Figure 2**, where localization at the nitrogen centre and delocalization over the aromatic π-system are clearly visible. This dual character underscores the reactivity of the open-shell singlet, in which a strongly localized nitrene centre is electronically stabilized by interaction with the aromatic framework.

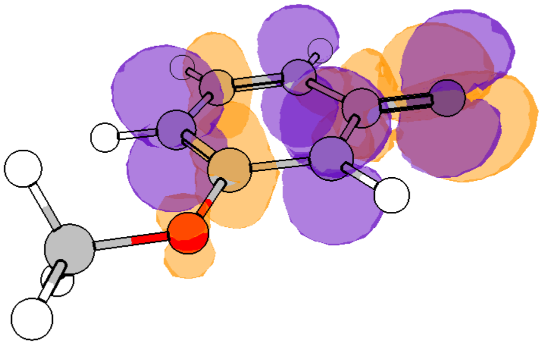

1q

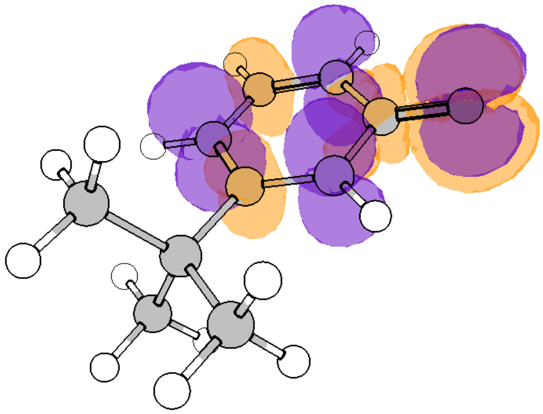

1r

Figure 2. Total spin density of singlet nitrenes (**II**) at UM06-2X/def2-QZVPP//def2-TZVP level of theory

Calculated Reaction Profiles

The mechanistic landscape for substrates **1q** and **1r** was explored along two distinct pathways, denoted as *path a* and *path b*, starting from the singlet nitrene intermediate (**II**). In both cases, the key transformation corresponds to the conversion of the nitrene into the respective azirine, which constitutes the rate-determining step of the process (**Figure 3-4**).

In the gas phase, the calculated energy profile for substrate **1q** shows that the nitrene-to-azirine conversion is favoured along *path a*, both kinetically and thermodynamically at the azirine level (**Figure 3**). The activation barrier for *step b* is 16.1 kcal mol^{-1} on *path a* versus 17.7 kcal mol^{-1} on *path b*. The resulting azirine intermediates (**III**) lie at -3.6 kcal mol^{-1} (*path a*) and +1.3 kcal mol-1 (*path b*), respectively. Subsequent **III**→ **IV** barriers are modest (8.6 kcal mol^{-1} for *path a*; 2.6 kcal mol^{-1} for *path b*). Taken together, these data indicate that formation of the azirine along *path a* dominates the reactivity landscape for **1q**, providing a straightforward rationale for the observed regioselectivity.

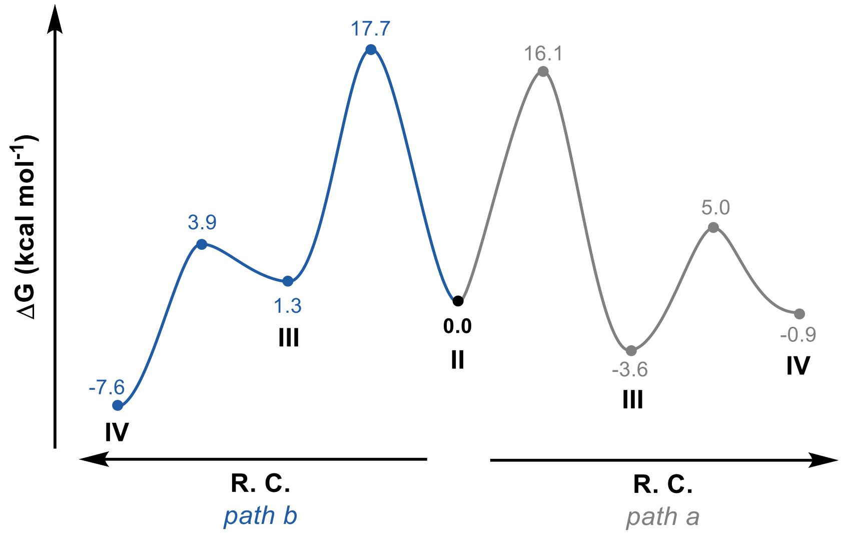

Figure 3. Calculated reaction energy profile for **1q**. The numbers are the relative Gibbs free energies at UM06-2X/def2-QZVPP//def2-TZVP level of theory (kcal mol^{-1})

To evaluate the influence of the environment, solvent corrections were applied using the SMD model in 1,4-dioxane and TFT. The relative trends observed in the gas phase were preserved across both solvents. The activation barriers for step b remained within ~1 kcal mol-1 of the gas-phase values. Importantly, the *path a* azirine continued to be more stable than its *path b* analogue. Thus, both in the gas phase and in solution, the mechanism of substrate **1q** is dominated by *path a*, and this preference is robust to solvation (**Table 13**).

Table 13. Calculated Gibbs free energies (kcal mol^{-1}) of intermediates and transition states along path a and path b for substrate **1q**, obtained at the UM06-2X/def2-QZVPP(SMD,solvent)//def2-TZVP(gas) level of theory. Energies are reported relative to **II**.

		**Gas**	**1,4-Dioxane**	**TFT**
	**II**	0.0	0.0	0.0
**path a**	**TS(II-III)**	16.1	15.8	15.6
	**III**	-3.6	-4.3	-4.2
	**TS(III-IV)**	5.0	4.6	5.1
	**IV**	-0.9	-0.6	0.1
**path b**	**TS(II-III)**	17.7	17.2	16.8
	**III**	1.3	0.4	0.5
	**TS(III-IV)**	3.9	3.4	3.9
	**IV**	-7.6	-7.5	-7.1

In the gas phase, the calculated activation barriers for *step b* of substrate **1r** were found to be very close in energy (**Figure 4**). *Path a* proceeds through a barrier of 17.6 kcal mol^{-1}, while the corresponding barrier for *path b* is slightly lower, at 16.9 kcal mol^{-1}. The resulting azirine intermediates (**III**) are likewise close in stability, with the *path a* azirine being favoured by 0.9 kcal mol^{-1}. These results show that the two pathways are energetically competitive, differing only marginally in both activation and intermediate energies.

On this basis, the azirine of *path b* can be described as the kinetic intermediate, since it is accessed through the lower activation barrier, whereas the azirine of *path a* corresponds to the thermodynamic intermediate, reflecting its greater stability. This energetic dichotomy provides a rationale for the observed temperature dependence. At room temperature, the similar activation barriers and near-degeneracy of the azirines suggest that both intermediates may be formed in comparable amounts. At elevated temperatures, however, the greater stability of the *path a* azirine is expected to drive the equilibrium towards this thermodynamically favoured intermediate, despite the slightly lower kinetic barrier of *path b*.

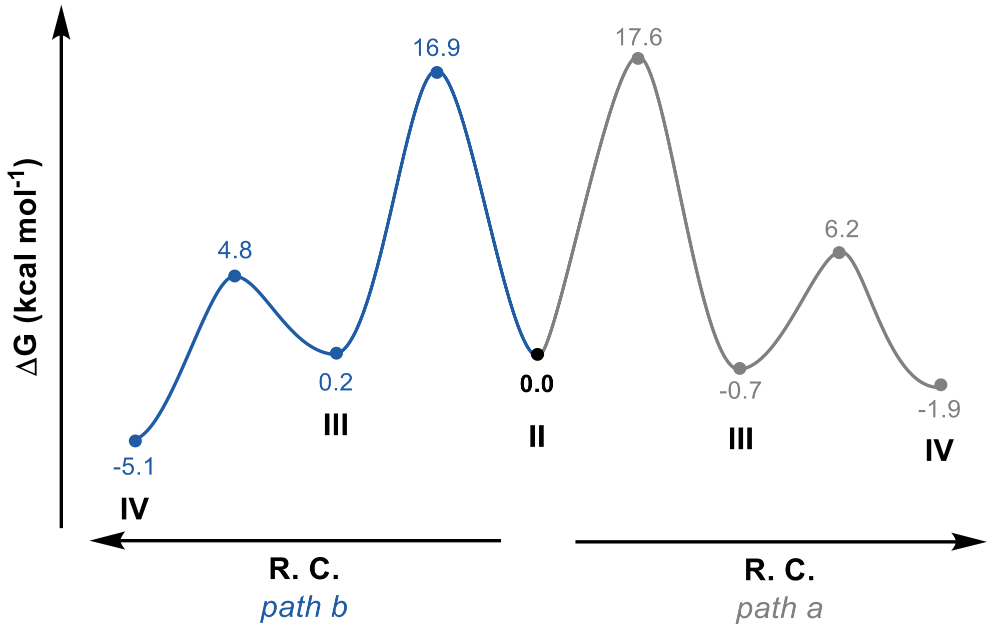

Figure 4. Calculated reaction energy profile for **1r**. The numbers are the relative Gibbs free energies at UM06-2X/def2-QZVPP//def2-TZVP level of theory (kcal mol^{-1})

To assess the impact of the environment, solvent corrections were included using the SMD model with 1,4-dioxane, THF and TFT. In all cases, the relative trends observed in the gas phase were preserved. The activation barriers for step b remained within ~1 kcal mol^{-1} of the gas-phase values, and the relative stability of the *path a* azirine over the path b analogue was consistently maintained. Thus, the kinetic versus thermodynamic balance identified in the gas-phase calculations is robust and not significantly altered by solvation (**Table 14**).

Scheme 1. Studied reaction mechanism

Table 14. Calculated Gibbs free energies (kcal mol^{-1}) of intermediates and transition states along path a and path b for substrate **1r**, obtained at the UM06-2X/def2-QZVPP(SMD,solvent)//def2-TZVP(gas) level of theory. Energies are reported relative to **II**.

		**Gas**	**1,4-Dioxane**	**THF**	**TFT**
	**II**	0.0	0.0	0.0	0.0
**path a**	**TS(II-III)**	17.6	17.3	17.0	17.0
	**III**	-0.7	-1.2	-1.1	-1.0
	**TS(III-IV)**	6.2	5.8	6.2	6.2
	**IV**	-1.9	-1.7	-1.2	-1.2
**path b**	**TS(II-III)**	16.9	15.5	16.2	16.1
	**III**	0.2	-0.6	-0.5	-0.5
	**TS(III-IV)**	4.8	4.3	4.7	4.7
	**IV**	-5.1	-4.9	-4.4	-4.5

Temperature dependence

To evaluate the effect of temperature, Gibbs free energies were corrected from 298 K to 333 K in 5 K increments using *Goodvibes.py*. The relative energies of intermediates and transition states remained essentially unchanged for **1q** (**Table 15**) and **1r** (**Table 16**).

Table 15. Calculated Gibbs free energies (kcal mol^{-1}) of intermediates and transition states for substrate **1q** at different temperatures. Relative energies remain consistent across the tested range. (UM06-2X/def2-QZVPP(SMD,solvent)//def2-TZVP(gas) level of theory)

			**298**	**303**	**308**	**313**	**318**	**323**	**328**	**333**
**Gas phase**		**II**	0.0	0.0	0.0	0.0	0.0	0.0	0.0	0.0
	**path a**	**TS(II-III)**	16.1	16.1	16.1	16.1	16.1	16.1	16.1	16.2
		**III**	-3.6	-3.6	-3.6	-3.6	-3.5	-3.5	-3.5	-3.5
		**TS(III-IV)**	5.0	5.0	5.0	5.0	5.0	5.1	5.1	5.1
		**IV**	-0.9	-0.9	-0.9	-0.9	-0.9	-0.9	-0.9	-0.9
	**path b**	**TS(II-III)**	17.7	17.7	17.7	17.7	17.7	17.7	17.7	17.7
		**III**	1.3	1.3	1.3	1.3	1.3	1.3	1.3	1.3
		**TS(III-IV)**	3.9	4.0	4.0	4.0	4.0	4.0	4.0	4.0
		**IV**	-7.6	-7.6	-7.6	-7.6	-7.6	-7.6	-7.5	-7.5
**1,4-Dioxane**		**II**	0.0	0.0	0.0	0.0	0.0	0.0	0.0	0.0
	**path a**	**TS(II-III)**	15.8	15.8	15.8	15.8	15.8	15.8	15.8	15.8
		**III**	-4.3	-4.3	-4.3	-4.3	-4.3	-4.3	-4.3	-4.3
		**TS(III-IV)**	4.6	4.6	4.7	4.7	4.7	4.7	4.7	4.7
		**IV**	-0.6	-0.6	-0.6	-0.6	-0.6	-0.6	-0.6	-0.6
	**path b**	**TS(II-III)**	17.2	17.2	17.2	17.2	17.2	17.2	17.2	17.2
		**III**	0.4	0.4	0.4	0.4	0.4	0.5	0.5	0.5
		**TS(III-IV)**	3.4	3.4	3.4	3.4	3.4	3.5	3.5	3.5
		**IV**	-7.5	-7.5	-7.5	-7.5	-7.5	-7.5	-7.5	-7.5
**TFT**		**II**	0.0	0.0	0.0	0.0	0.0	0.0	0.0	0.0
	**path a**	**TS(II-III)**	15.6	15.6	15.6	15.6	15.6	15.6	15.6	15.6
		**III**	-4.2	-4.2	-4.2	-4.2	-4.2	-4.2	-4.2	-4.2
		**TS(III-IV)**	5.1	5.1	5.1	5.1	5.1	5.2	5.2	5.2
		**IV**	0.1	0.1	0.1	0.1	0.1	0.1	0.1	0.1
	**path b**	**TS(II-III)**	16.8	16.8	16.8	16.8	16.8	16.8	16.8	16.8
		**III**	0.5	0.5	0.5	0.5	0.5	0.5	0.5	0.5
		**TS(III-IV)**	3.9	3.9	3.9	3.9	3.9	3.9	3.9	3.9
		**IV**	-7.1	-7.1	-7.0	-7.0	-7.0	-7.0	-7.0	-7.0

Table 16. Calculated Gibbs free energies (kcal mol^{-1}) of intermediates and transition states for substrate **1r** at different temperatures. Relative energies remain consistent across the tested range. (UM06-2X/def2-QZVPP(SMD,solvent)//def2-TZVP(gas) level of theory)

			298	303	308	313	318	323	328	333
Gas phase		II	0.0	0.0	0.0	0.0	0.0	0.0	0.0	0.0
	path a	TS(II-III)	17.6	17.6	17.6	17.6	17.6	17.7	17.7	17.7
		III	-0.7	-0.7	-0.7	-0.7	-0.7	-0.7	-0.7	-0.7
		TS(III-IV)	6.2	6.2	6.2	6.2	6.2	6.2	6.3	6.3
		IV	-1.9	-1.9	-1.9	-1.8	-1.8	-1.8	-1.8	-1.8
	path b	TS(II-III)	16.9	16.9	17.0	17.0	17.0	17.0	17.0	17.0
		III	0.2	0.2	0.2	0.2	0.2	0.2	0.2	0.2
		TS(III-IV)	4.8	4.8	4.8	4.8	4.8	4.8	4.8	4.8
		IV	-5.1	-5.1	-5.1	-5.1	-5.1	-5.1	-5.1	-5.1
1,4-Dioxane		II	0.0	0.0	0.0	0.0	0.0	0.0	0.0	0.0
	path a	TS(II-III)	17.3	17.3	17.3	17.3	17.3	17.3	17.3	17.3
		III	-1.2	-1.2	-1.2	-1.2	-1.2	-1.2	-1.2	-1.2
		TS(III-IV)	5.8	5.9	5.9	5.9	5.9	5.9	5.9	5.9
		IV	-1.7	-1.7	-1.7	-1.7	-1.7	-1.7	-1.6	-1.6
	path b	TS(II-III)	16.5	16.5	16.5	16.5	16.5	16.5	16.5	16.6
		III	-0.6	-0.6	-0.6	-0.6	-0.6	-0.5	-0.5	-0.5
		TS(III-IV)	4.3	4.3	4.3	4.3	4.3	4.3	4.4	4.4
		IV	-4.9	-4.9	-4.9	-4.9	-4.9	-4.9	-4.9	-4.9
THF		II	0.0	0.0	0.0	0.0	0.0	0.0	0.0	0.0
	path a	TS(II-III)	17.0	17.0	17.1	17.1	17.1	17.1	17.1	17.1
		III	-1.1	-1.1	-1.0	-1.0	-1.0	-1.0	-1.0	-1.0
		TS(III-IV)	6.2	6.2	6.2	6.2	6.3	6.3	6.3	6.3
		IV	-1.2	-1.2	-1.2	-1.2	-1.2	-1.2	-1.2	-1.2
	path b	TS(II-III)	16.2	16.2	16.2	16.2	16.2	16.2	16.2	16.2
		III	-0.5	-0.5	-0.5	-0.5	-0.5	-0.4	-0.4	-0.4
		TS(III-IV)	4.7	4.7	4.7	4.7	4.7	4.7	4.7	4.8
		IV	-4.4	-4.4	-4.4	-4.4	-4.4	-4.4	-4.4	-4.4
TFT		II	0.0	0.0	0.0	0.0	0.0	0.0	0.0	0.0
	path a	TS(II-III)	17.0	17.0	17.0	17.0	17.0	17.0	17.0	17.0
		III	-1.0	-1.0	-1.0	-1.0	-1.0	-1.0	-1.0	-1.0
		TS(III-IV)	6.2	6.2	6.2	6.2	6.3	6.3	6.3	6.3
		IV	-1.2	-1.2	-1.2	-1.2	-1.2	-1.2	-1.2	-1.2

path b	**TS(II-III)**	16.1	16.1	16.1	16.1	16.2	16.2	16.2	16.2
	**III**	-0.5	-0.5	-0.5	-0.5	-0.5	-0.4	-0.4	-0.4
	**TS(III-IV)**	4.7	4.7	4.7	4.7	4.7	4.7	4.8	4.8
	**IV**	-4.5	-4.5	-4.4	-4.4	-4.4	-4.4	-4.4	-4.4

14. Pictures of Reaction Set-up

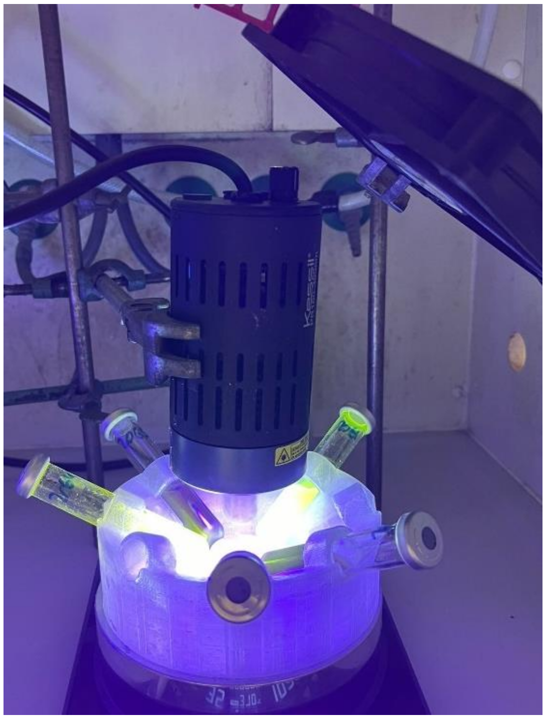

Figure S1. Set-up for 0.1 mmol scale reactions at 370, 390, 427 nm.

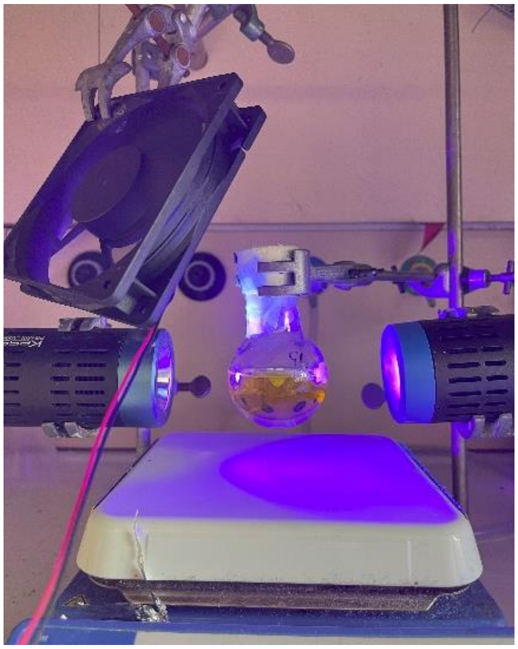

Figure S2. Set-up for gram-scale reactions at 390 nm.

15. References

(1) Li, B.; Ruffoni, A.; Leonori, D. A Photochemical Strategy for Ortho-Aminophenol Synthesis via Dearomative-Rearomative Coupling Between Aryl Azides and Alcohols. *Angew. Chem. Int. Ed.* **2023**, *62* (52), e202310540. https://doi.org/10.1002/anie.202310540.

(2) Yang, Z.; Du, F.-H.; Zhang, C.; Du, Y. Accessing Aryl Azides via Copper Powder-Catalyzed Cross-Coupling of Arylboronic Acids with the Hypervalent Azido-Iodine Reagent ABZ(I). *Org. Chem. Front.* **2023**, *10* (16), 4131–4138. https://doi.org/10.1039/D3QO00732D.

(3) Markiewicz, J. T.; Wiest, O.; Helquist, P. Synthesis of Primary Aryl Amines Through a Copper-Assisted Aromatic Substitution Reaction with Sodium Azide. *J. Org. Chem.* **2010**, *75* (14), 4887–4890. https://doi.org/10.1021/jo101002p.

(4) Cummings, S. P.; Le, T.-N.; Fernandez, G. E.; Quiambao, L. G.; Stokes, B. J. Tetrahydroxydiboron-Mediated Palladium-Catalyzed Transfer Hydrogenation and Deuteriation of Alkenes and Alkynes Using Water as the Stoichiometric H or D Atom Donor. *J. Am. Chem. Soc.* **2016**, *138* (19), 6107–6110. https://doi.org/10.1021/jacs.6b02132.

(5) Xu, S.; Zhuang, X.; Pan, X.; Zhang, Z.; Duan, L.; Liu, Y.; Zhang, L.; Ren, X.; Ding, K. 1-Phenyl-4-Benzoyl-1H-1,2,3-Triazoles as Orally Bioavailable Transcriptional Function Suppressors of Estrogen-Related Receptor α. *J. Med. Chem.* **2013**, *56* (11), 4631–4640. https://doi.org/10.1021/jm4003928.

(6) Teregulova, A. N.; Yarullin, A. R.; Chainikova, E. M.; Lobov, A. N.; Safiullin, R. L.; Khursan, S. L. Transformations of 4-R-6-Oxohexa-2,4-Diene Nitrile Oxides – Intermediates of Photooxidation of *Para*-R-C6H4N3, R = Ph, CH2Ph, OPh. *Tetrahedron* **2024**, *153*, 133849. https://doi.org/10.1016/j.tet.2024.133849.

(7) Pearson, T. J.; Shimazumi, R.; Driscoll, J. L.; Dherange, B. D.; Park, D.-I.; Levin, M. D. Aromatic Nitrogen Scanning by Ipso-Selective Nitrene Internalization. *Science* **2023**, *381* (6665), 1474–1479. https://doi.org/10.1126/science.adj5331.

(8) Zhou, F.; Driver, T. G. Efficient Synthesis of 3H-Indoles Enabled by the Lead-Mediated α-Arylation of β-Ketoesters or γ-Lactams Using Aryl Azides. *Org. Lett.* **2014**, *16* (11), 2916–2919. https://doi.org/10.1021/ol5010615.

(9) Cho, Y. A.; Kim, D.-S.; Ahn, H. R.; Canturk, B.; Molander, G. A.; Ham, J. Preparation of Potassium Azidoaryltrifluoroborates and Their Cross-Coupling with Aryl Halides. *Org. Lett.* **2009**, *11* (19), 4330–4333. https://doi.org/10.1021/ol901669k.

(10) Zuo, Z.; Liu, X.; Qian, X.; Zeng, T.; Sang, N.; Liu, H.; Zhou, Y.; Tao, L.; Zhou, X.; Su, N.; Yu, Y.; Chen, Q.; Luo, Y.; Zhao, Y. Bifunctional Naphtho[2,3-d][1,2,3]Triazole-4,9-Dione Compounds Exhibit Antitumor Effects In Vitro and In Vivo by Inhibiting Dihydroorotate Dehydrogenase and Inducing Reactive Oxygen Species Production. *J. Med. Chem.* **2020**, *63* (14), 7633–7652. https://doi.org/10.1021/acs.jmedchem.0c00512.

(11) Zhu, W.; Ma, D. Synthesis of Aryl Azides and Vinyl Azides via Proline-Promoted CuI-Catalyzed Coupling Reactions. *Chem. Commun.* **2004**, No. 7, 888–889. https://doi.org/10.1039/B400878B.

(12) Bertrand, H. C.; Schaap, M.; Baird, L.; Georgakopoulos, N. D.; Fowkes, A.; Thiollier, C.; Kachi, H.; Dinkova-Kostova, A. T.; Wells, G. Design, Synthesis, and Evaluation of Triazole Derivatives That Induce Nrf2 Dependent Gene Products and Inhibit the Keap1–Nrf2 Protein–Protein Interaction. *J. Med. Chem.* **2015**, *58* (18), 7186–7194. https://doi.org/10.1021/acs.jmedchem.5b00602.

(13) Bończak, B.; Lisowski, W.; Kamińska, A.; Hołdyński, M.; Fiałkowski, M. Gold Nanoparticles Functionalized with Fully Conjugated Fullerene C60 Derivatives as a Material

with Exceptional Capability of Absorbing Electrons. *J. Phys. Chem. C* **2019**, *123* (10), 6229–6240. https://doi.org/10.1021/acs.jpcc.8b10842.
(14) Powers, I. G.; Andjaba, J. M.; Luo, X.; Mei, J.; Uyeda, C. Catalytic Azoarene Synthesis from Aryl Azides Enabled by a Dinuclear Ni Complex. *J. Am. Chem. Soc.* **2018**, *140* (11), 4110–4118. https://doi.org/10.1021/jacs.8b00503.
(15) Berger, F.; Plutschack, M. B.; Riegger, J.; Yu, W.; Speicher, S.; Ho, M.; Frank, N.; Ritter, T. Site-Selective and Versatile Aromatic C−H Functionalization by Thianthrenation. *Nature* **2019**, *567* (7747), 223–228. https://doi.org/10.1038/s41586-019-0982-0.
(16) Aukland, M. H.; Šiaučiulis, M.; West, A.; Perry, G. J. P.; Procter, D. J. Metal-Free Photoredox-Catalysed Formal C–H/C–H Coupling of Arenes Enabled by Interrupted Pummerer Activation. *Nat. Catal.* **2020**, *3* (2), 163–169. https://doi.org/10.1038/s41929-019-0415-3.
(17) Kafuta, K.; Korzun, A.; Böhm, M.; Golz, C.; Alcarazo, M. Synthesis, Structure, and Reactivity of 5-(Aryl)Dibenzothiophenium Triflates. *Angew. Chem. Int. Ed.* **2020**, *59* (5), 1950–1955. https://doi.org/10.1002/anie.201912383.
(18) P. Hohenberg, W. Kohn, "Inhomogeneous Electron Gas," *Phys. Rev.*, **1964**, *136*, B864-B71. https://doi.org/10.1103/PhysRev.136.B864; W. Kohn, L. J. Sham, "Self-Consistent Equations Including Exchange and Correlation Effects," *Phys. Rev.*, **1965**, *140*, A1133-A38. https://doi.org/10.1103/PhysRev.140.A1133; R. G. Parr, W. Yang, *Density-functional theory of atoms and molecules* (Oxford Univ. Press, Oxford, 1989); *Challenge of d and f Electrons*, Ed. D. R. Salahub, M. C. Zerner (ACS, Washington, D.C., 1989). https://doi.org/10.1021/bk-1989-0394.
(19) M. J. Frisch, G. W. Trucks, H. B. Schlegel, G. E. Scuseria, M. A. Robb, J. R. Cheeseman, G. Scalmani, V. Barone, G. A. Petersson, H. Nakatsuji, X. Li, M. Caricato, A. V. Marenich, J. Bloino, B. G. Janesko, R. Gomperts, B. Mennucci, H. P. Hratchian, J. V. OrΘz, A. F. Izmaylov, J. L. Sonnenberg, Williams, F. Ding, F. Lipparini, F. Egidi, J. Goings, B. Peng, A. Petrone, T. Henderson, D. Ranasinghe, V. G. Zakrzewski, J. Gao, N. Rega, G. Zheng, W. Liang, M. Hada, M. Ehara, K. Toyota, R. Fukuda, J. Hasegawa, M. Ishida, T. Nakajima, Y. Honda, O. Kitao, H. Nakai, T. Vreven, K. Throssell, J. A. Montgomery Jr., J. E. Peralta, F. Ogliaro, M. J. Bearpark, J. J. Heyd, E. N. Brothers, K. N. Kudin, V. N. Staroverov, T. A. Keith, R. Kobayashi, J. Normand, K. Raghavachari, A. P. Rendell, J. C. Burant, S. S. Iyengar, J. Tomasi, M. Cossi, J. M. Millam, M. Klene, C. Adamo, R. Cammi, J. W. Ochterski, R. L. MarΘn, K. Morokuma, O. Farkas, J. B. Foresman, D. J. Fox, Wallingford, CT, 2016.
(20) Y. Zhao, D. G. Truhlar, "The M06 suite of density functionals for main group thermochemistry, thermochemical kinetics, noncovalent interactions, excited states, and transition elements: two new functionals and systematic testing of four M06-class functionals and 12 other functionals" *Theor. Chem. Acc.*, **2008**, *120*, 215-41. https://doi.org/10.1007/s00214-007-0310-x.
(21) F. Weigend, R. Ahlrichs, "Balanced basis sets of split valence, triple zeta valence and quadruple zeta valence quality for H to Rn: Design and assessment of accuracy," *Phys. Chem. Chem. Phys.*, **2005**, *7*, 3297-305. https://doi.org/10.1039/B508541A
(22) F. Weigend, R. Ahlrichs, "Balanced basis sets of split valence, triple zeta valence and quadruple zeta valence quality for H to Rn: Design and assessment of accuracy," *Phys. Chem. Chem. Phys.*, **2005**, *7*, 3297-305. https://doi.org/10.1039/B508541A
(23) A. V. Marenich, C. J. Cramer, D. G. Truhlar, "Universal solvation model based on solute electron density and a continuum model of the solvent defined by the bulk dielectric constant

and atomic surface tensions," *J. Phys. Chem. B*, **2009,** *113*, 6378-96. https://doi.org /10.1021/jp810292n
(24) Grimme, S. Chem. Eur. J. **2012**, *18*, 9955–9964 https://doi.org /10.1021/jp509921r

(25) Luchini, G.; Alegre-Requena, J. V.; Funes-Ardoiz, I.; Paton, R. S. GoodVibes: Automated Thermochemistry for Heterogeneous Computational Chemistry Data. F1000Research, 2020, 9, 291 https://doi.org /10.12688/f1000research.22758.1

16. NMR Spectra

S6a – ^{1}H NMR (600 MHz, $CDCl_3$)

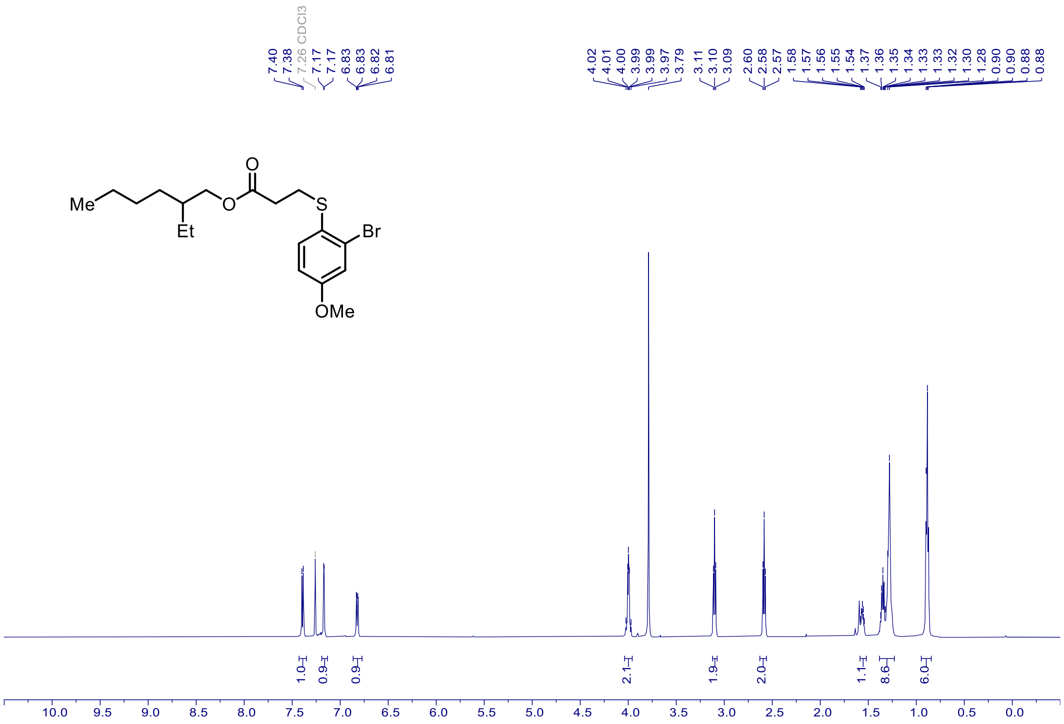

S6a – ^{13}C NMR (151 MHz, $CDCl_3$)

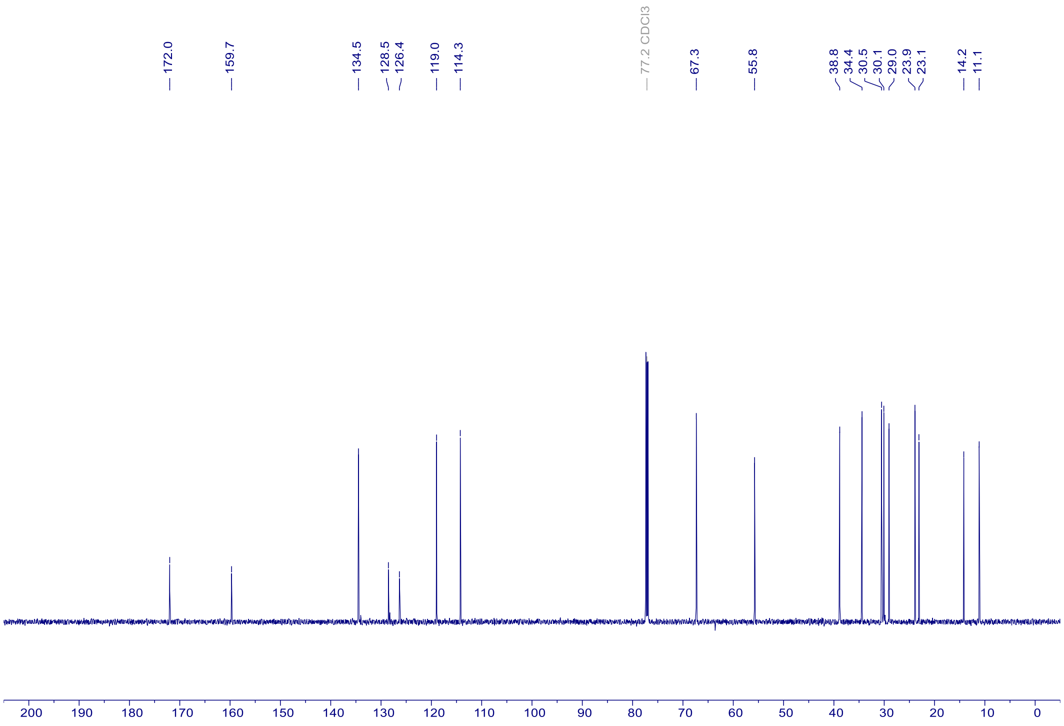

S6b – ^{19}F NMR (565 MHz, $CDCl_3$)

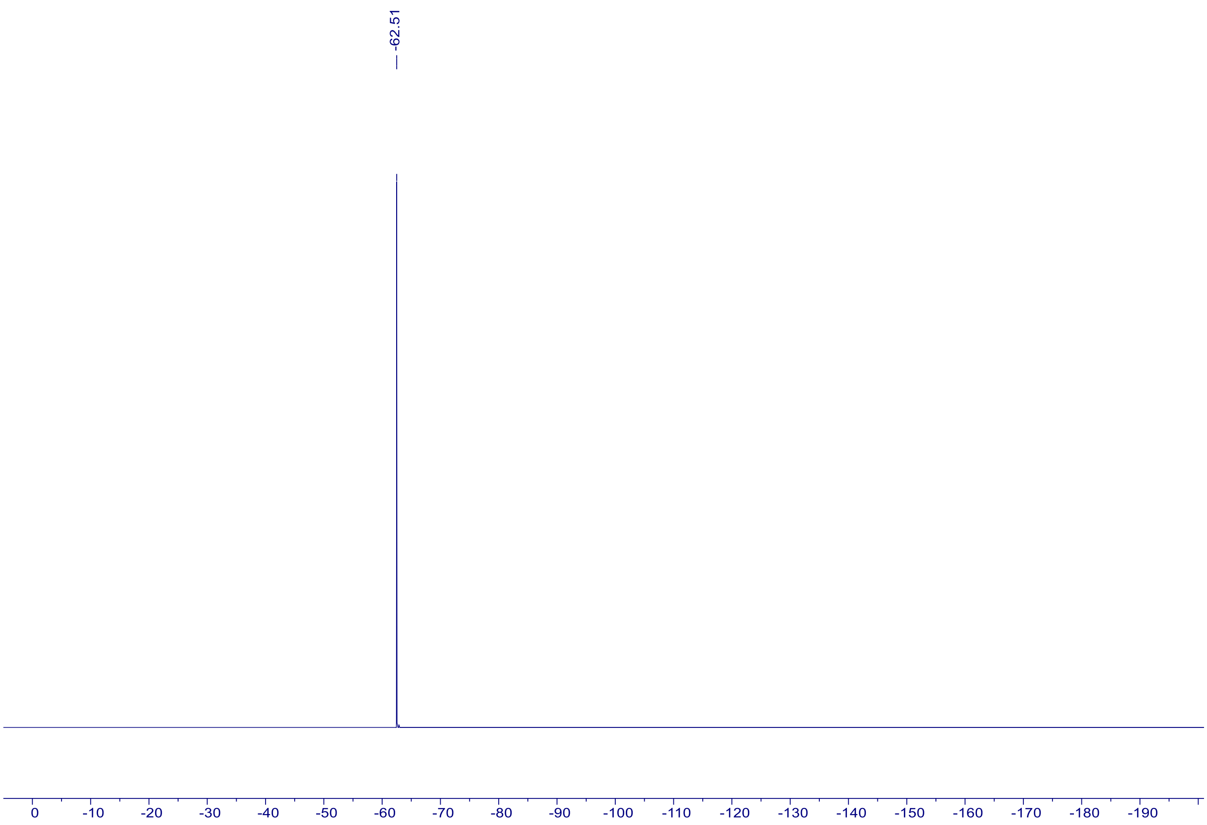

S6c – ^{1}H NMR (600 MHz, $CDCl_3$)

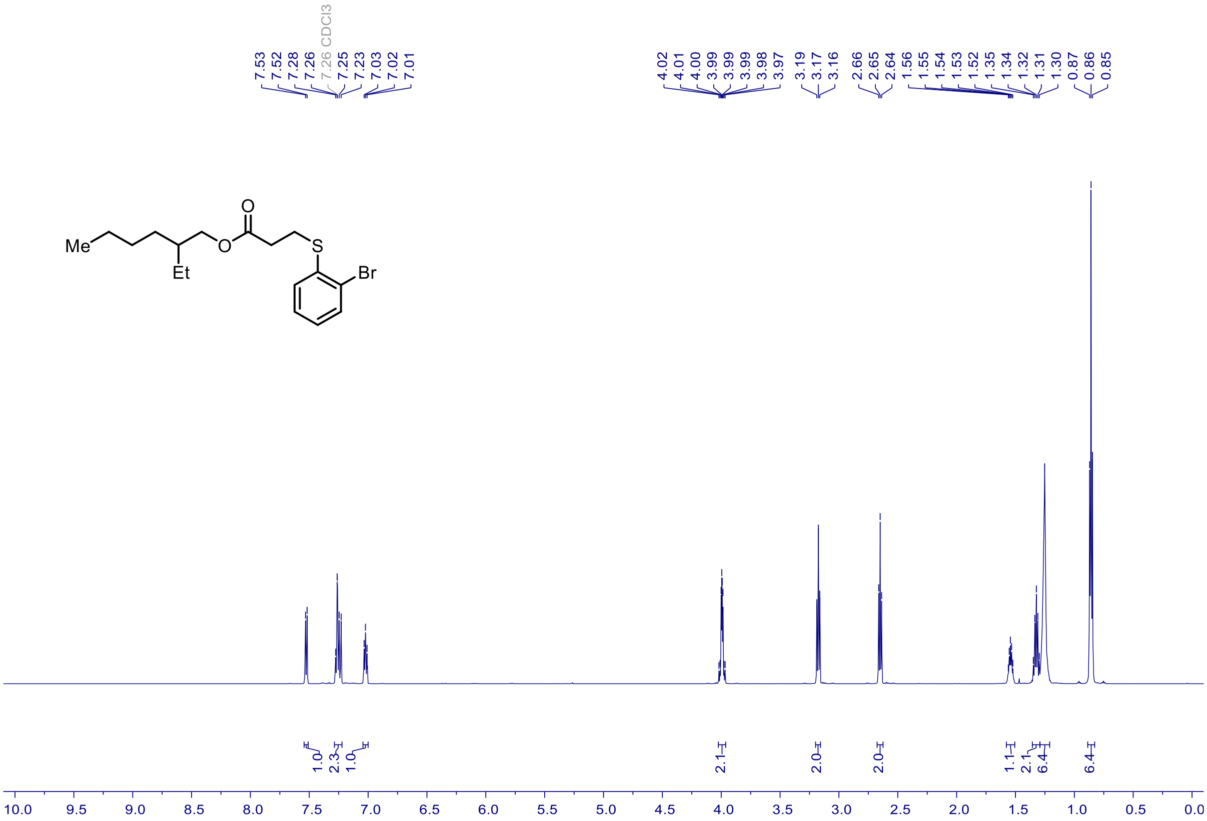

S6c – ^{13}C NMR (151 MHz, $CDCl_3$)

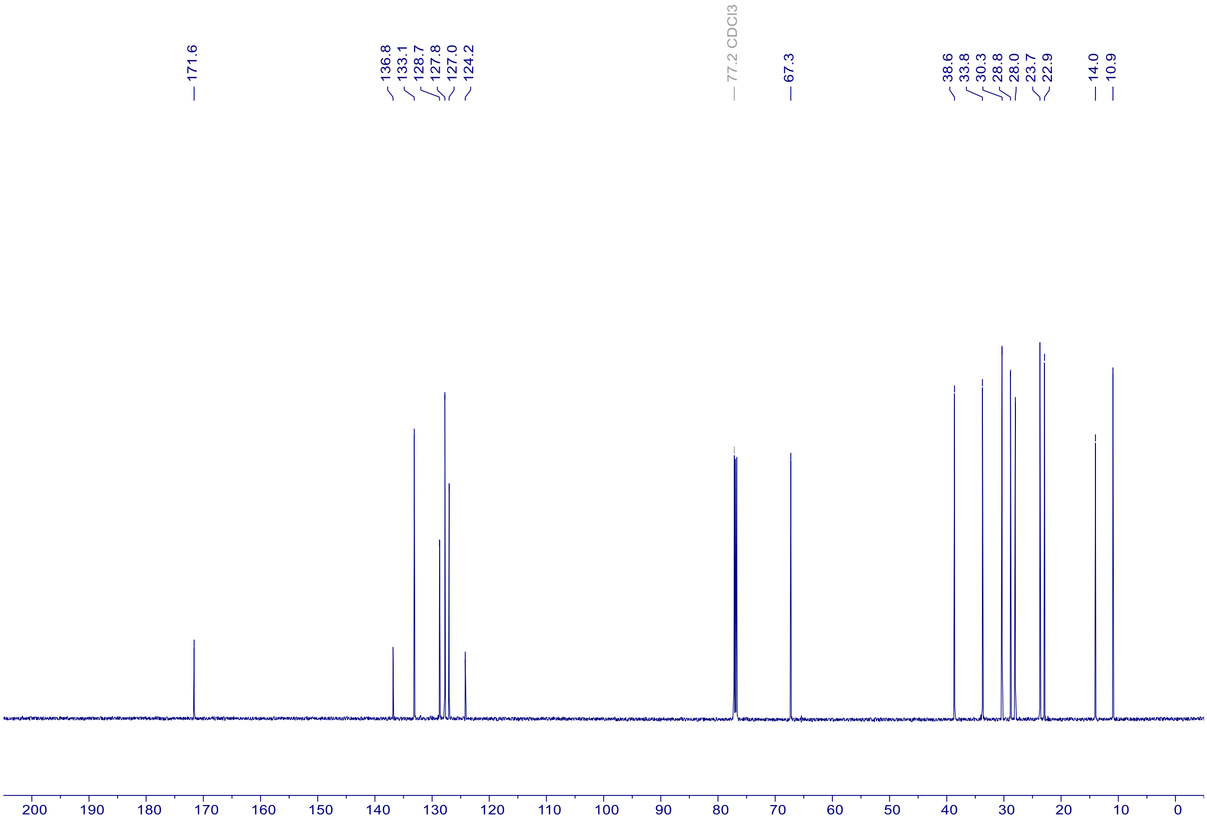

2d – ^{1}H NMR (600 MHz, $CDCl_3$)

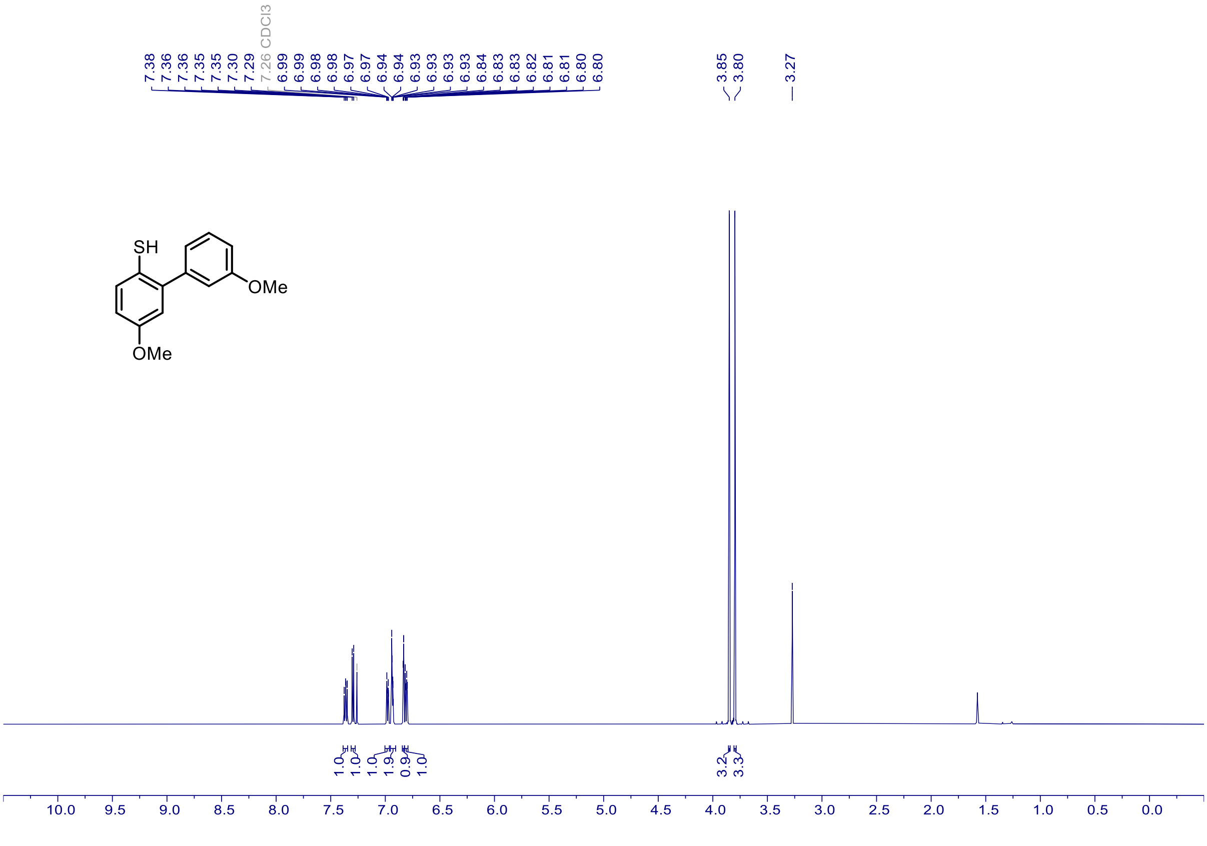

2d – ^{13}C NMR (151 MHz, $CDCl_3$)

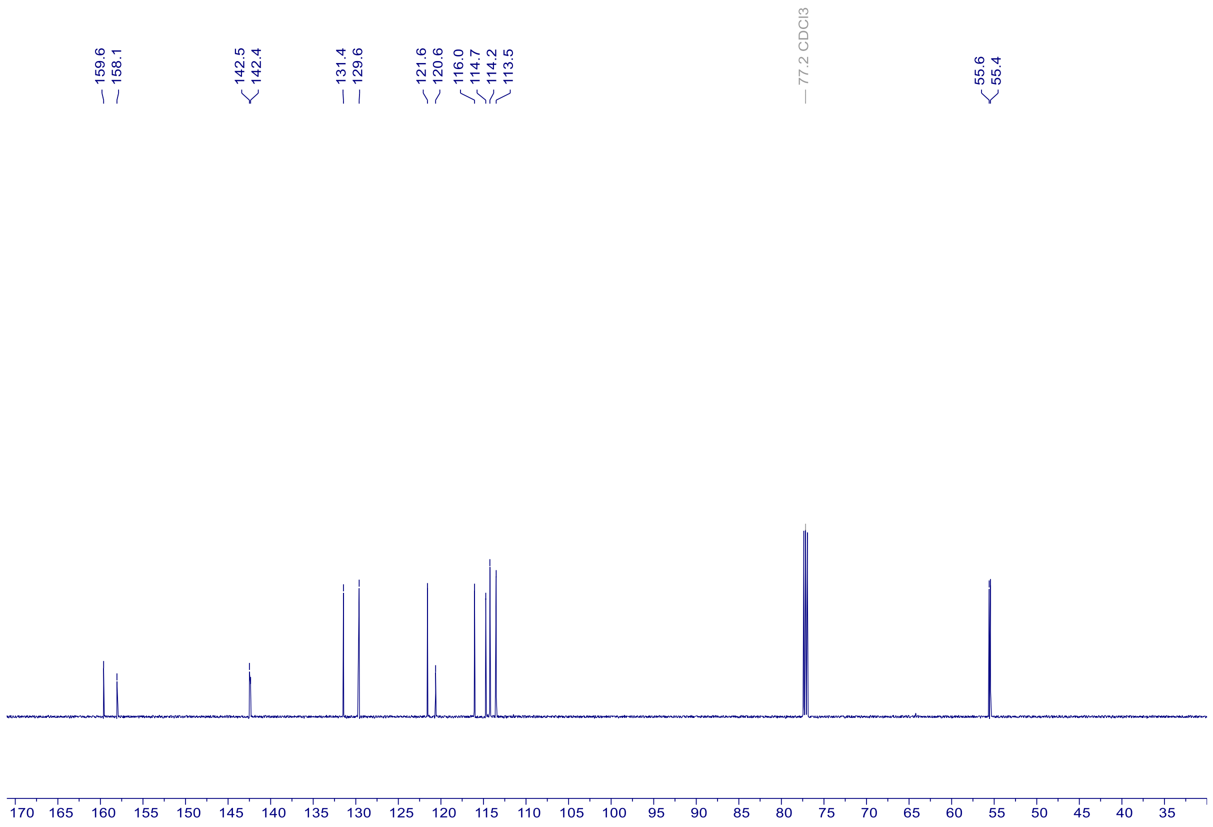

2e – ^{1}H NMR (600 MHz, $CDCl_3$)

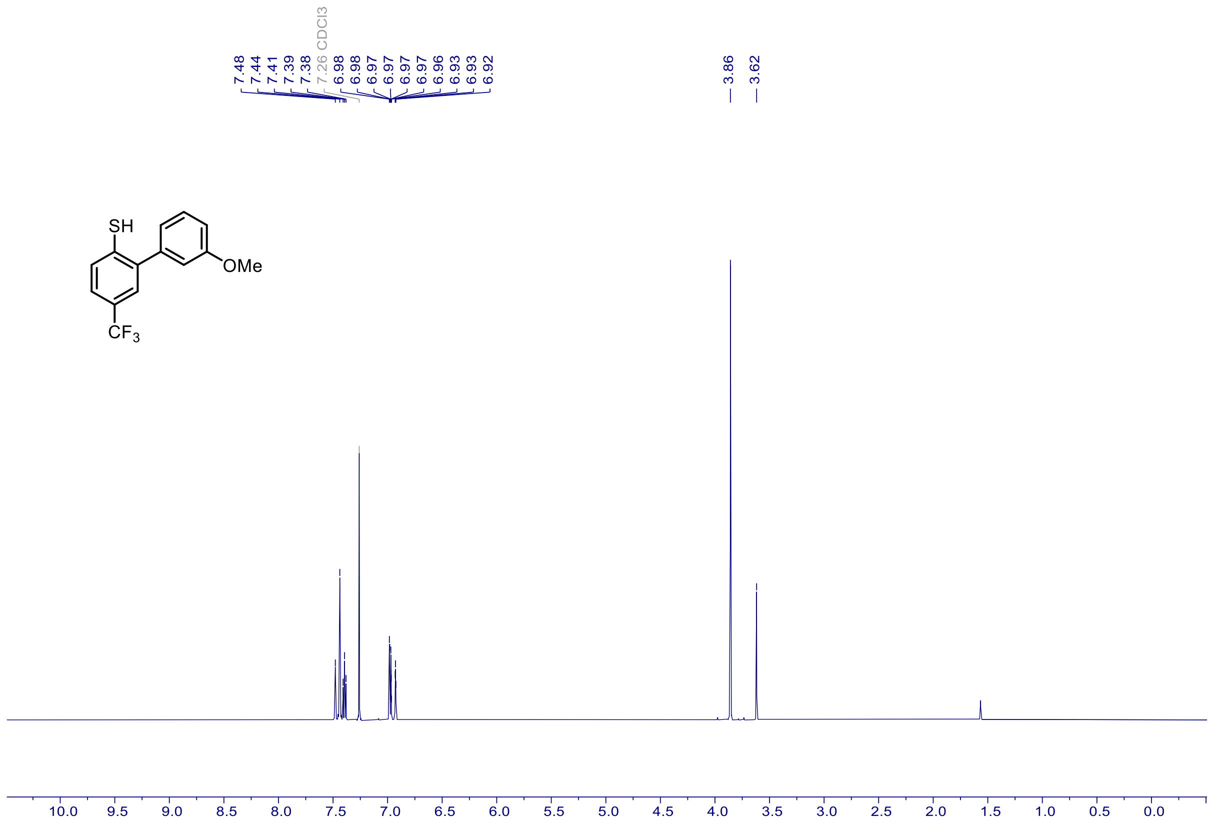

2e – ^{13}C NMR (151 MHz, CDCl$_3$)

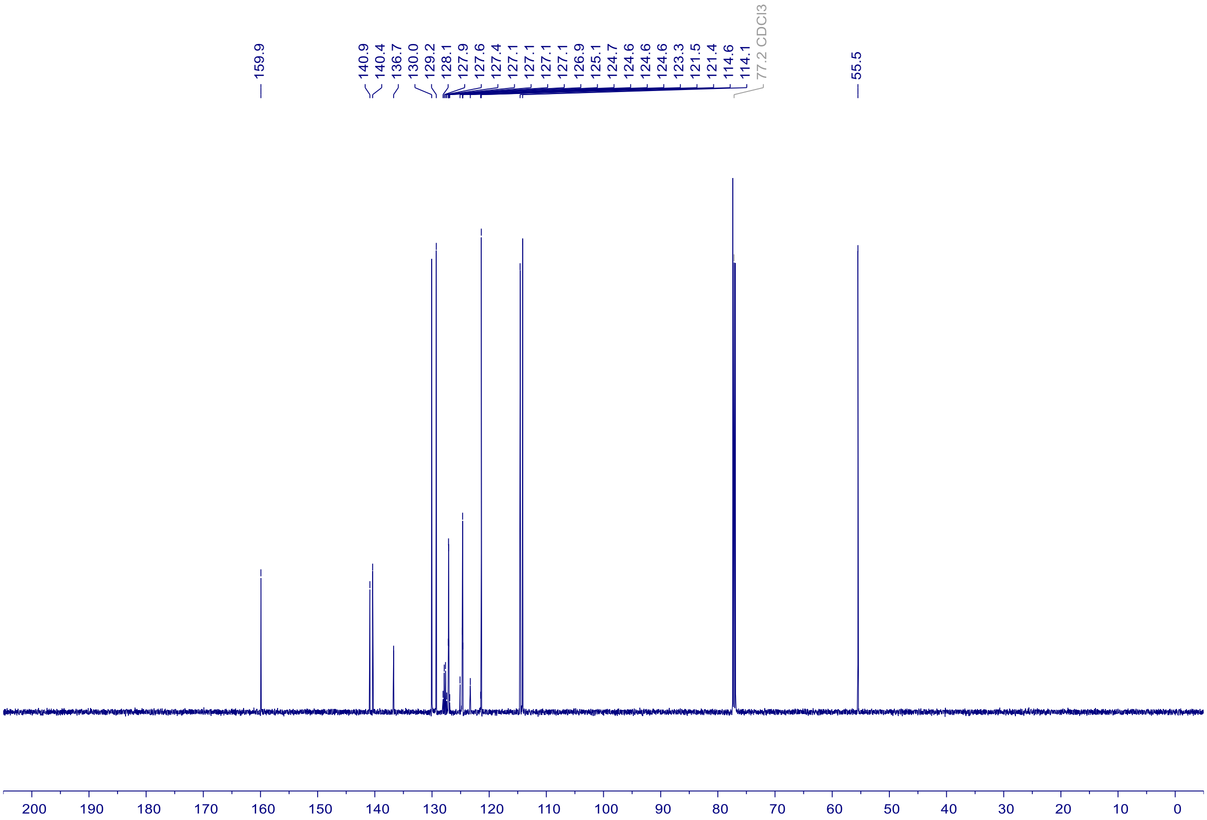

2e – ^{19}F NMR (565 MHz, CDCl$_3$)

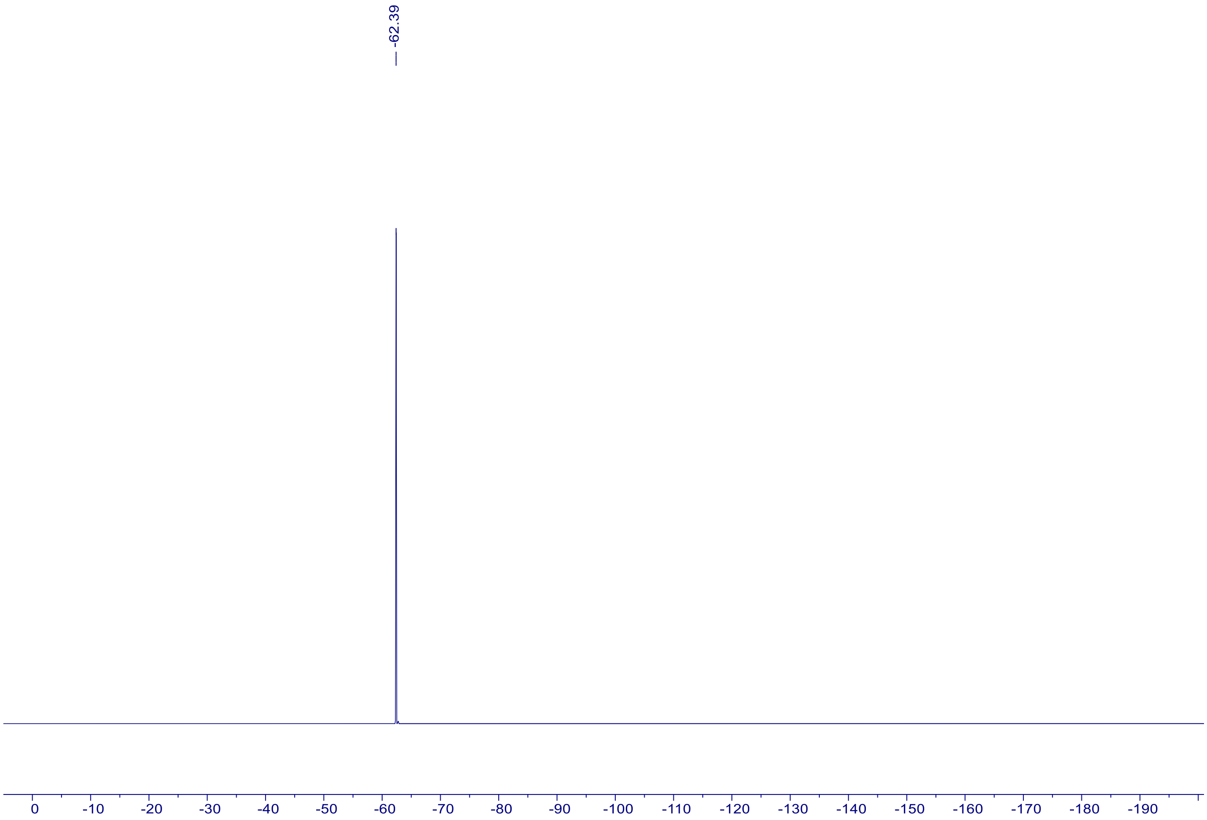

2f – ^{1}H NMR (600 MHz, $CDCl_3$)

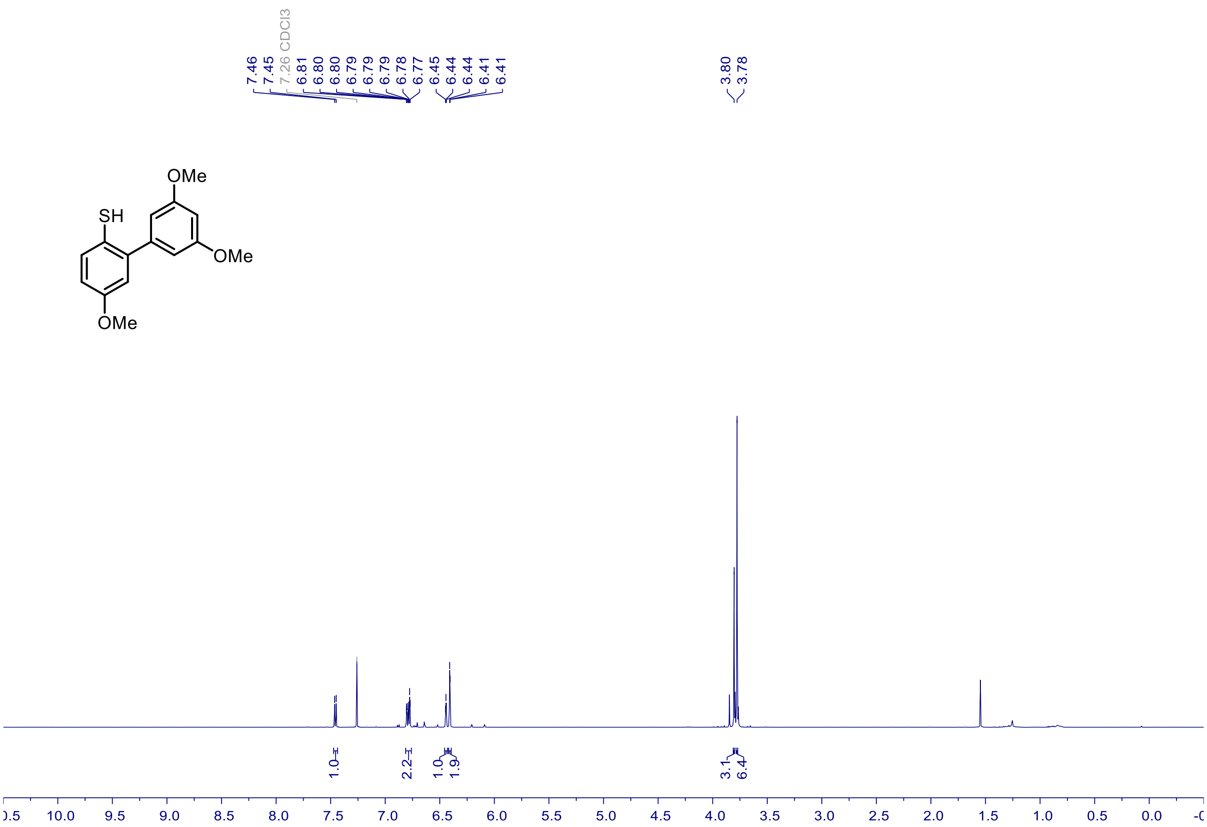

2f – ^{13}C NMR (151 MHz, $CDCl_3$)

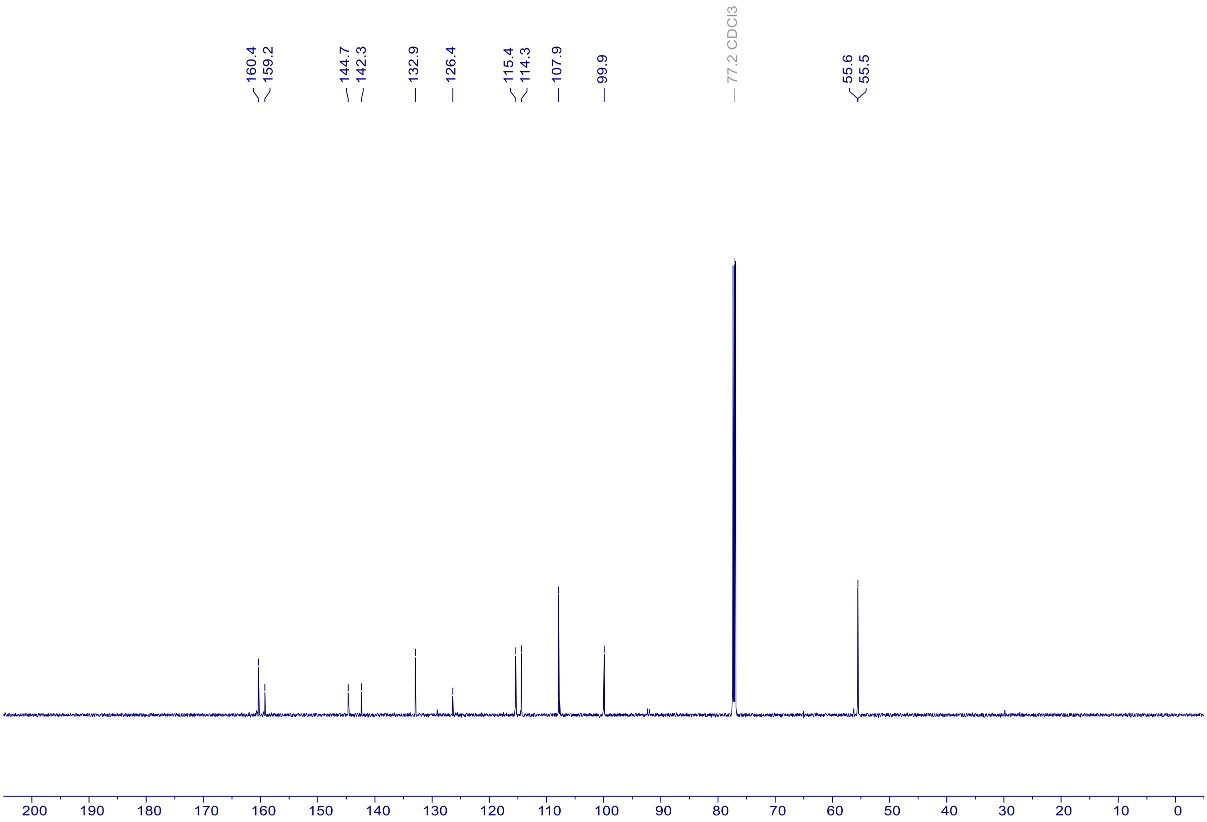

2g – ^{1}H NMR (600 MHz, $CDCl_3$)

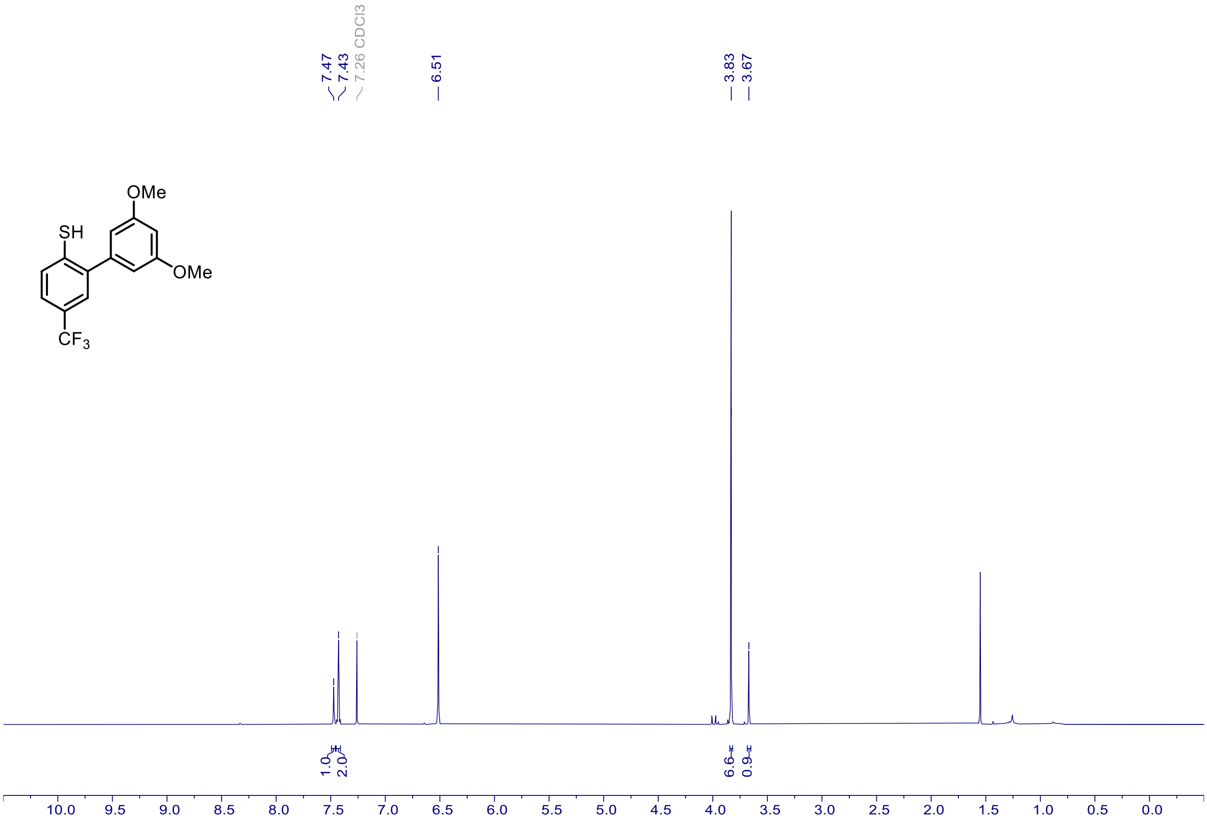

2g – ^{13}C NMR (151 MHz, $CDCl_3$)

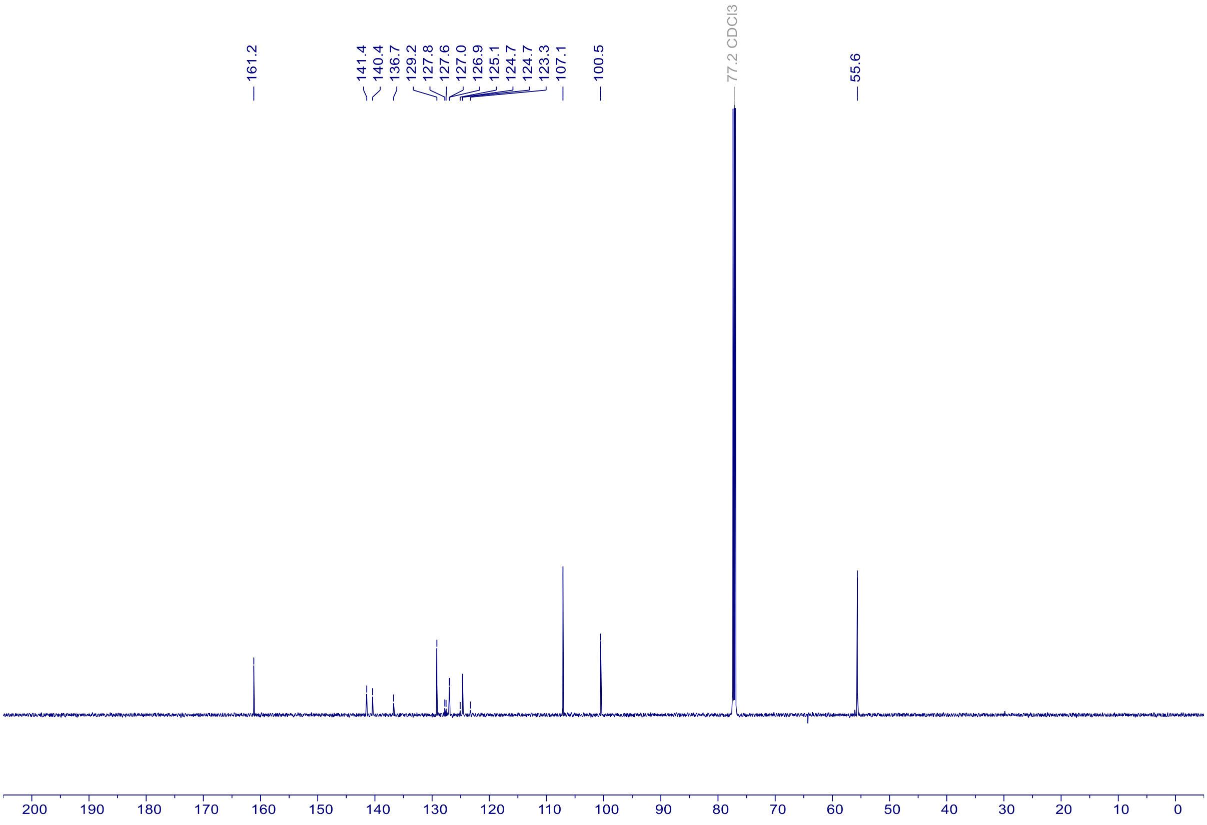

2g – ^{19}F NMR (565 MHz, $CDCl_3$)

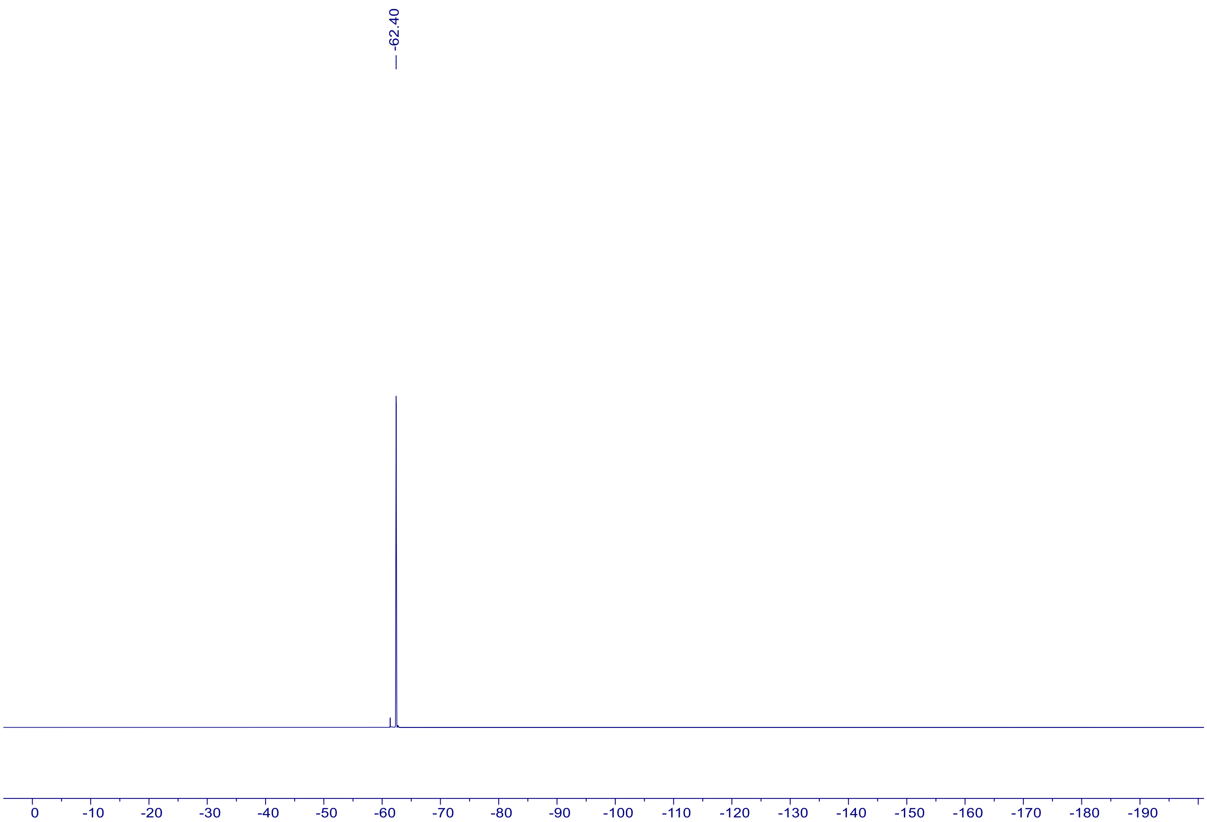

2h – ^{1}H NMR (600 MHz, $CDCl_3$)

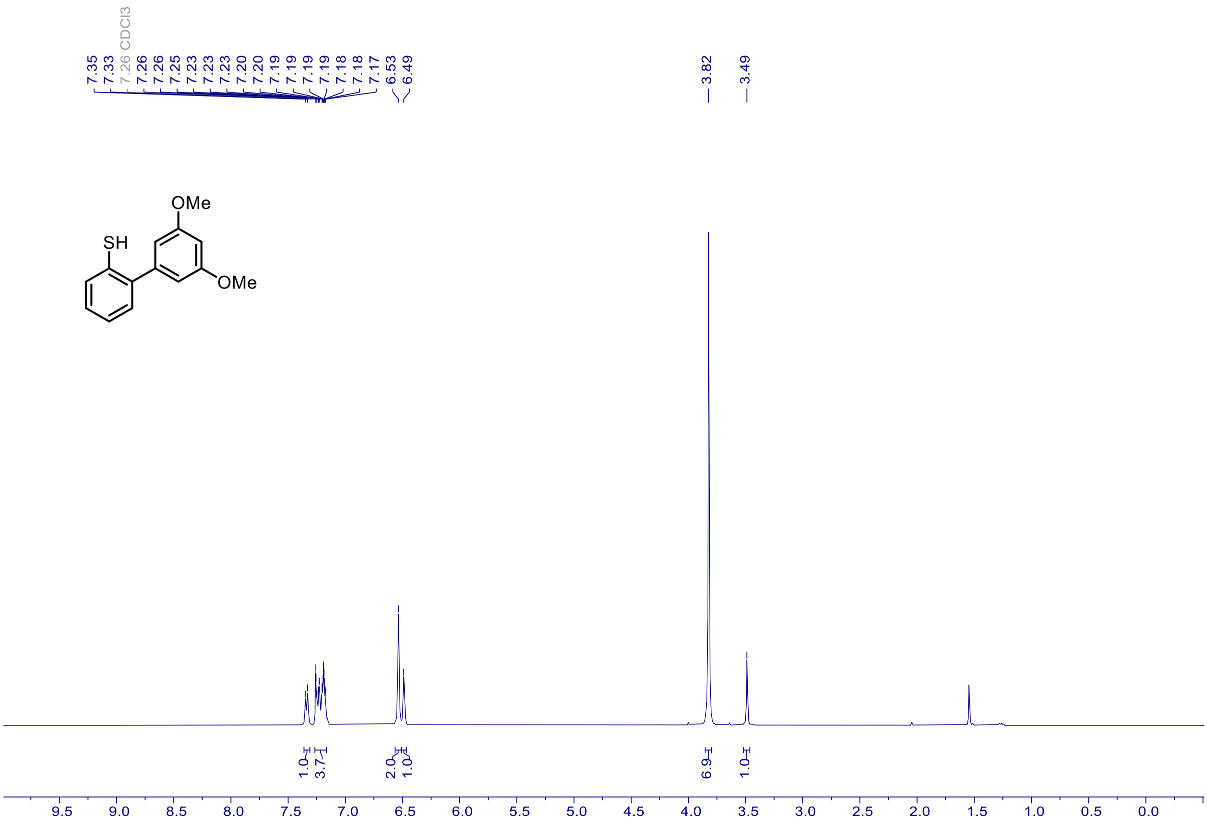

2h – ^{13}C NMR (151 MHz, CDCl$_3$)

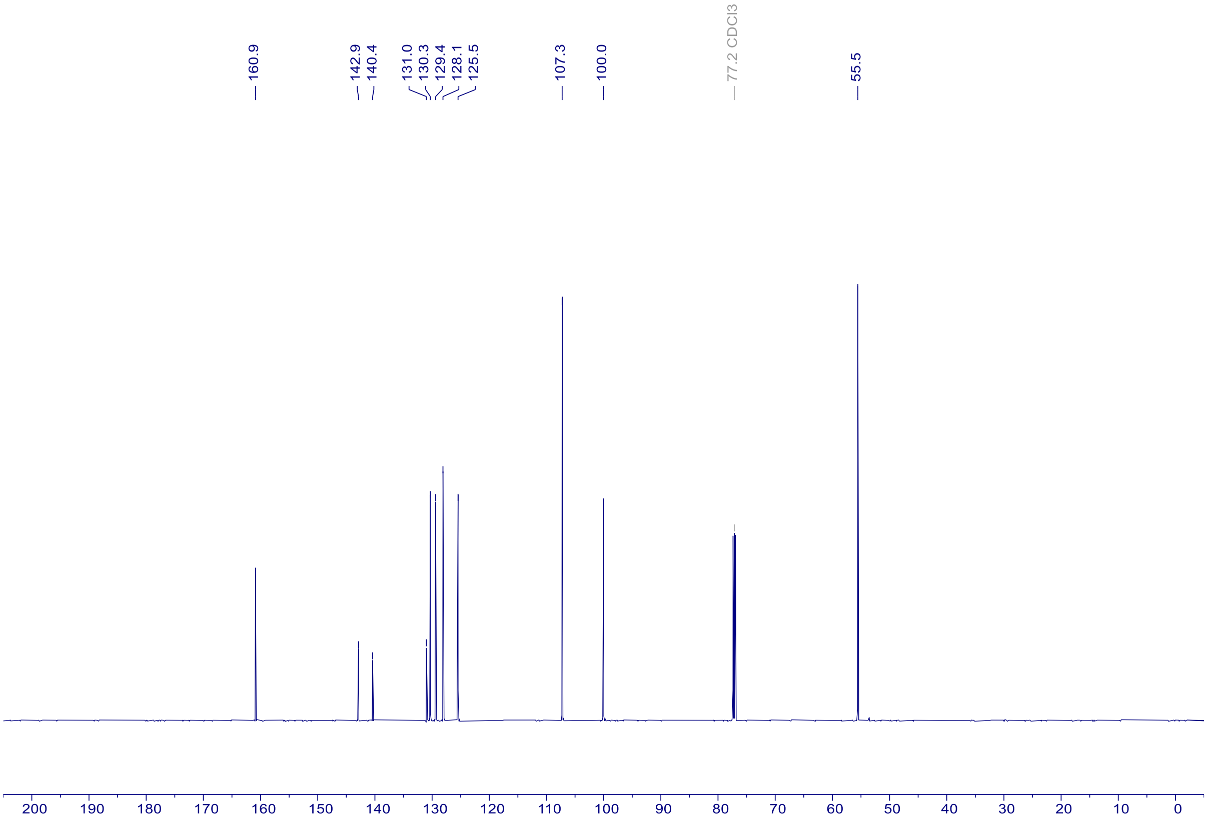

S1ah – ^{1}H NMR (600 MHz, CDCl$_3$)

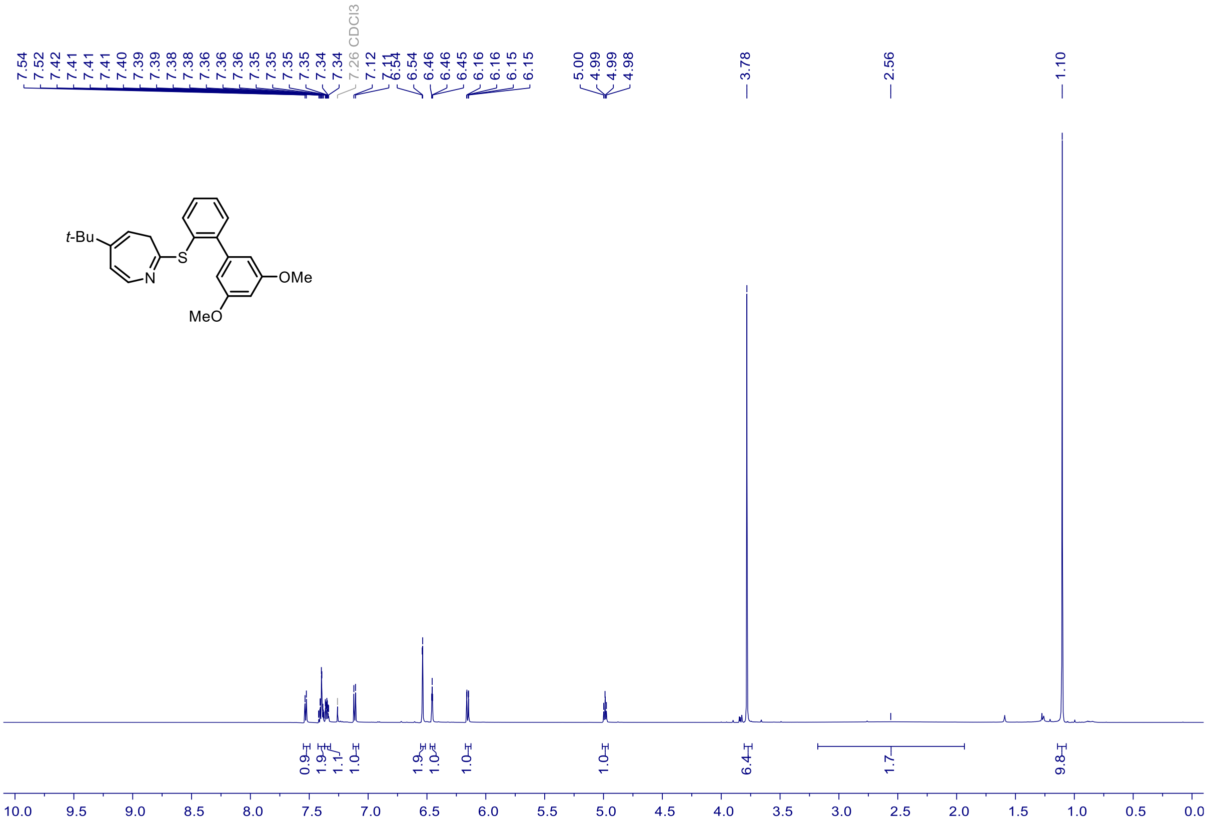

S1ah – ^{13}C NMR (151 MHz, $CDCl_3$)

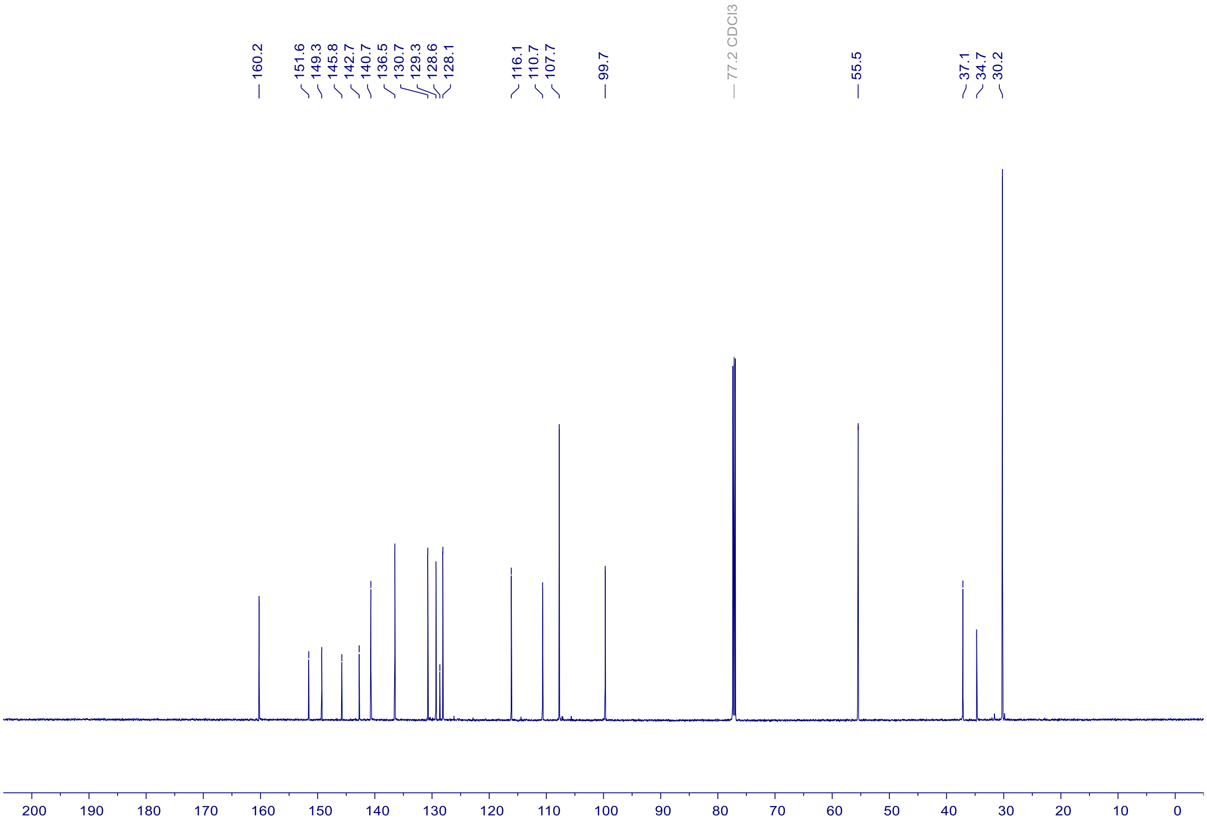

3ah – ^{1}H NMR (600 MHz, $CDCl_3$)

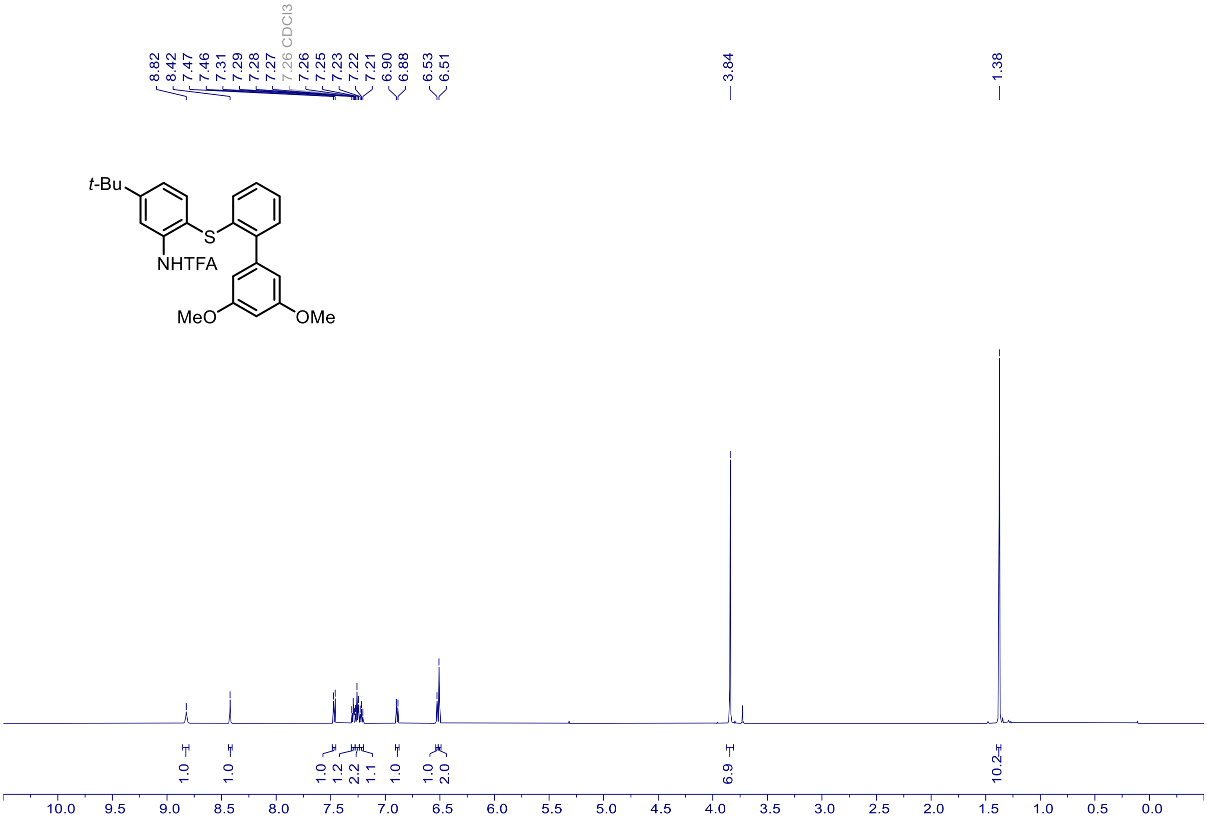

3ah – ^{13}C NMR (151 MHz, CDCl$_3$)

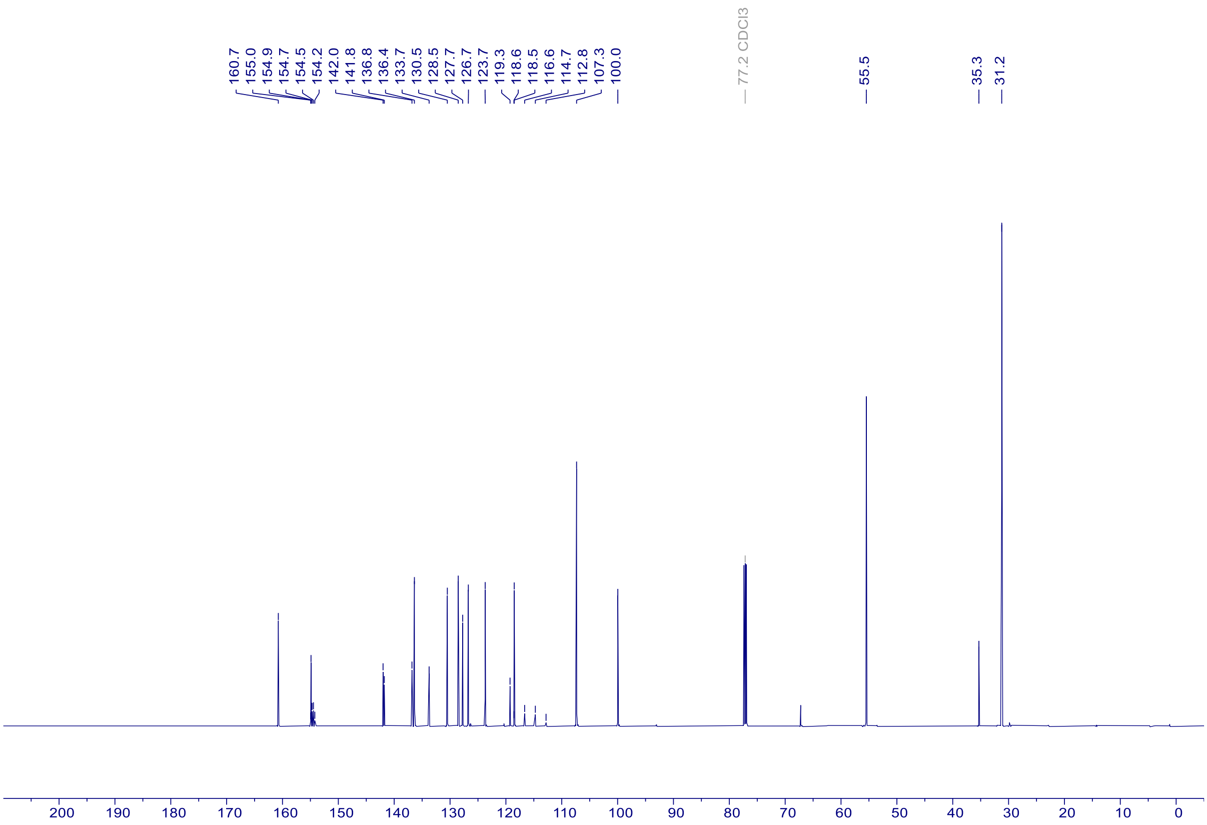

3ah – ^{19}F NMR (565 MHz, CDCl$_3$)

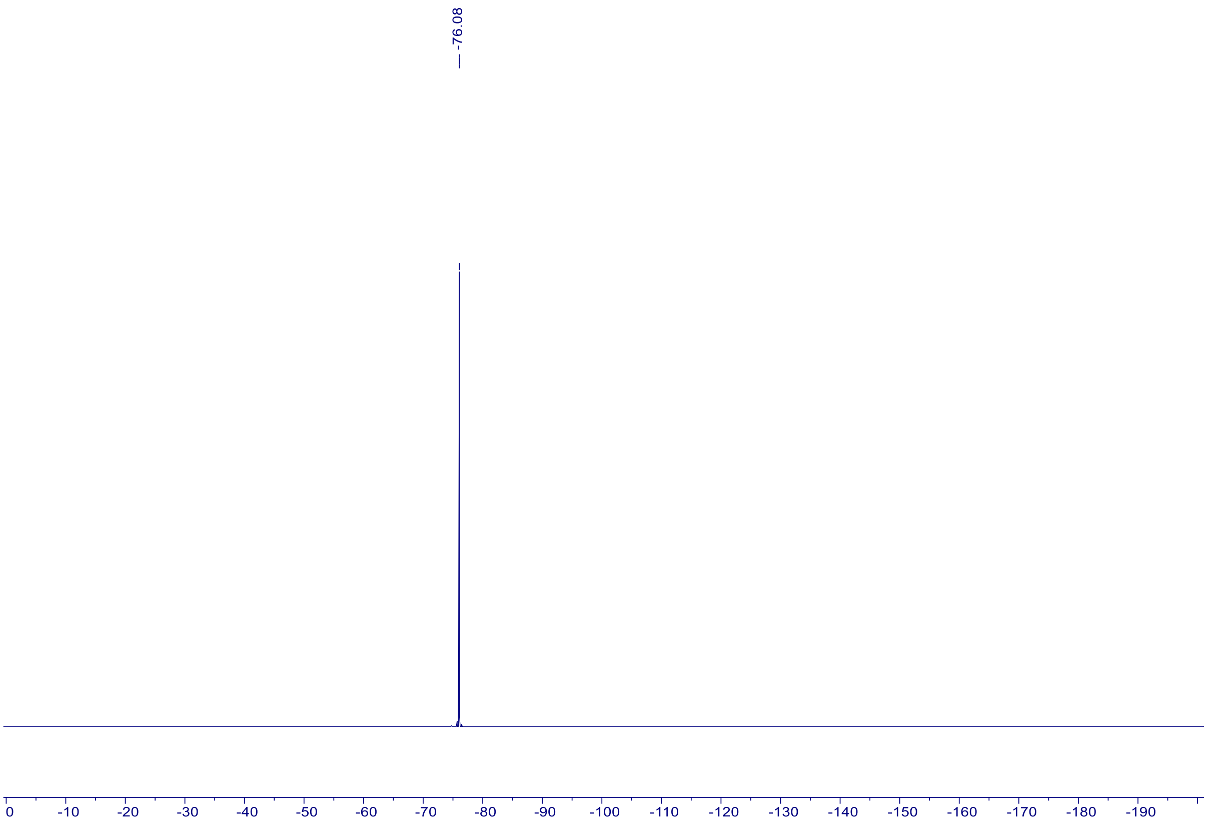

4ah – ^{1}H NMR (600 MHz, $CDCl_3$)

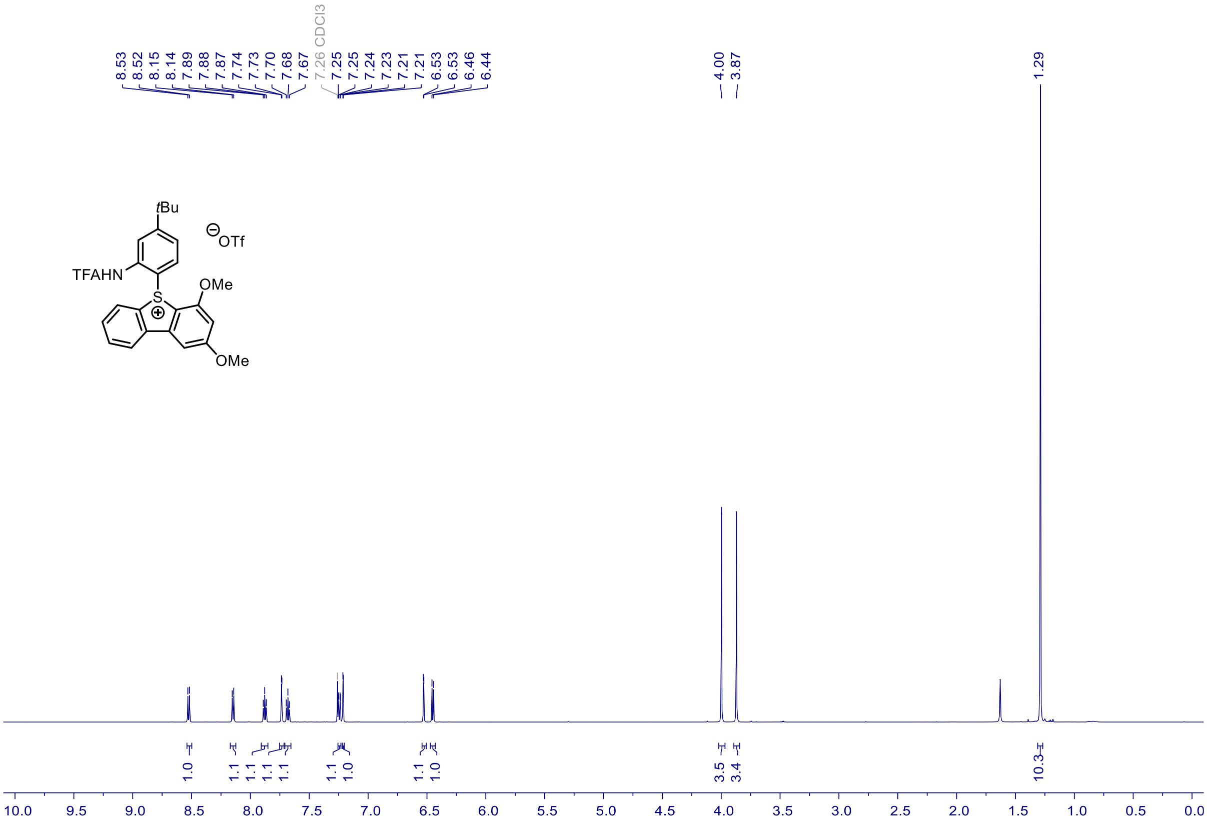

4ah – ^{13}C NMR (151 MHz, $CDCl_3$)

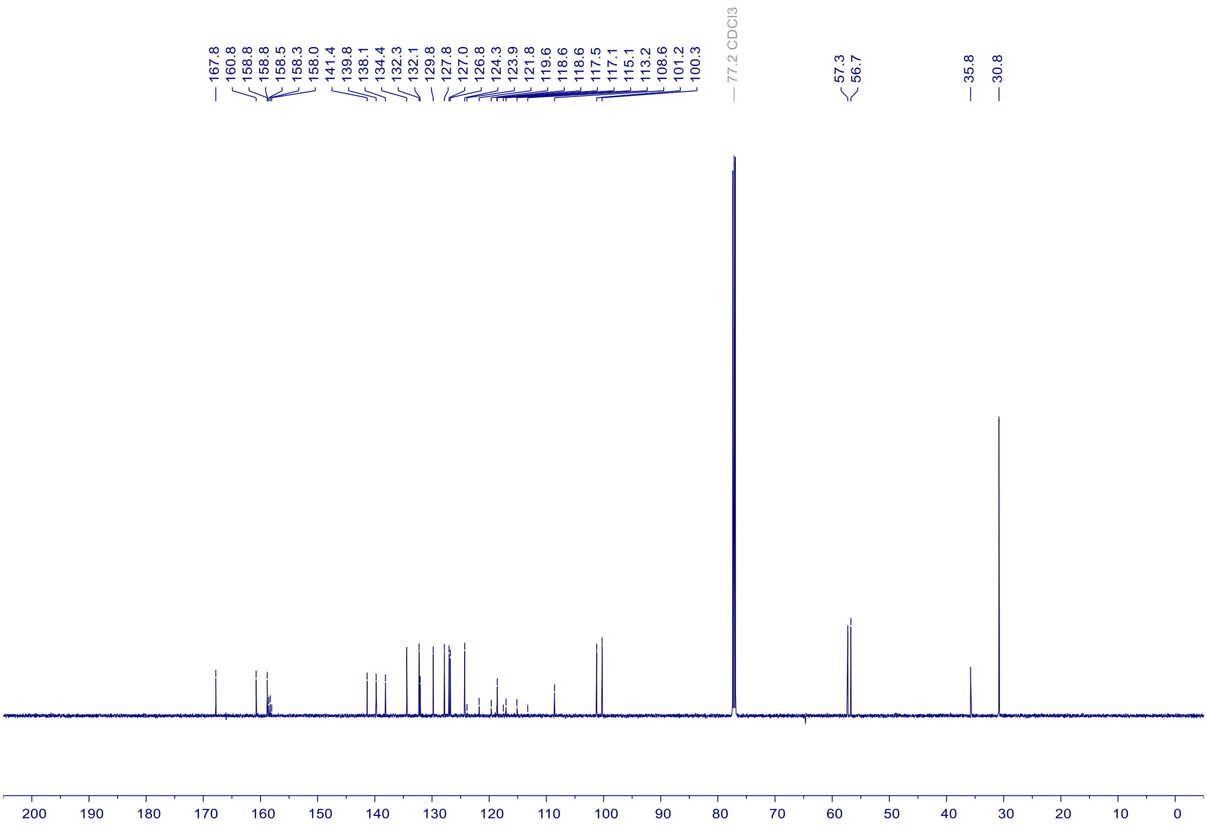

4ah – ^{19}F NMR (565 MHz, $CDCl_3$)

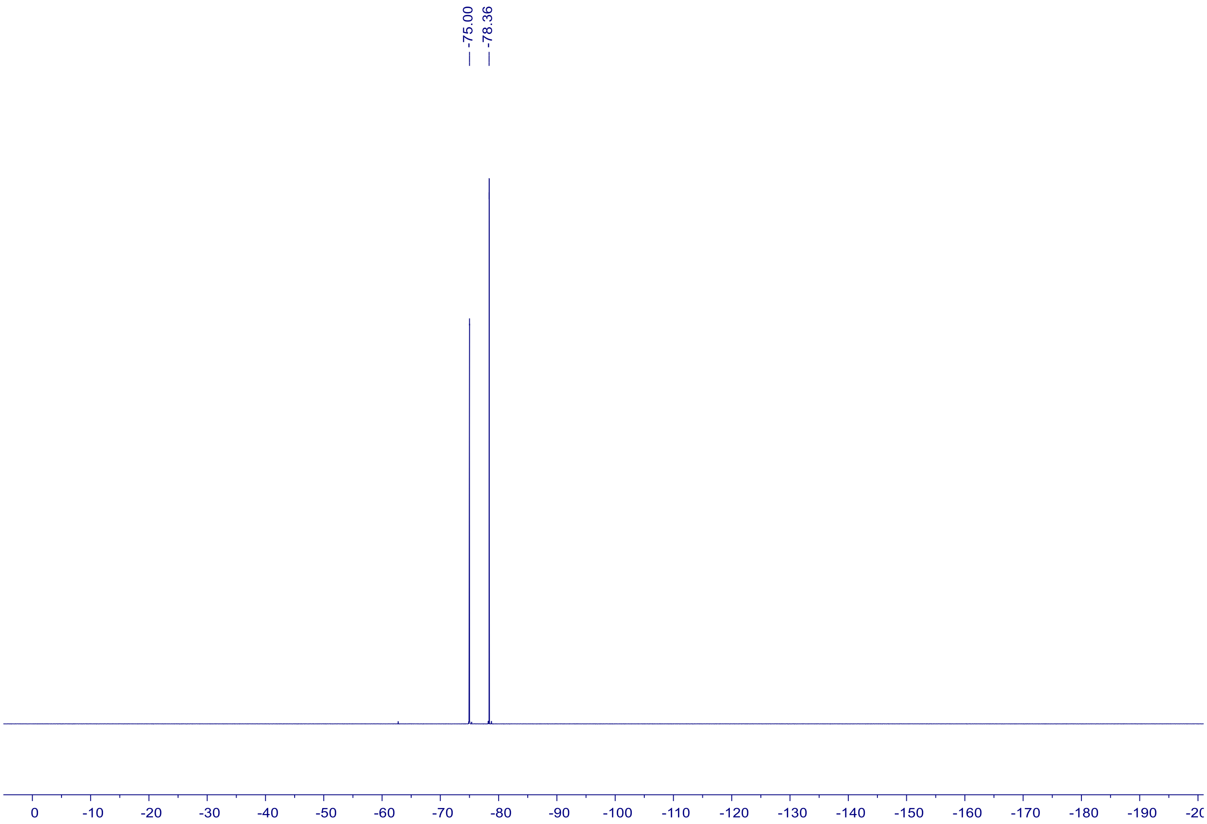

5a – ^{1}H NMR (600 MHz, $CDCl_3$)

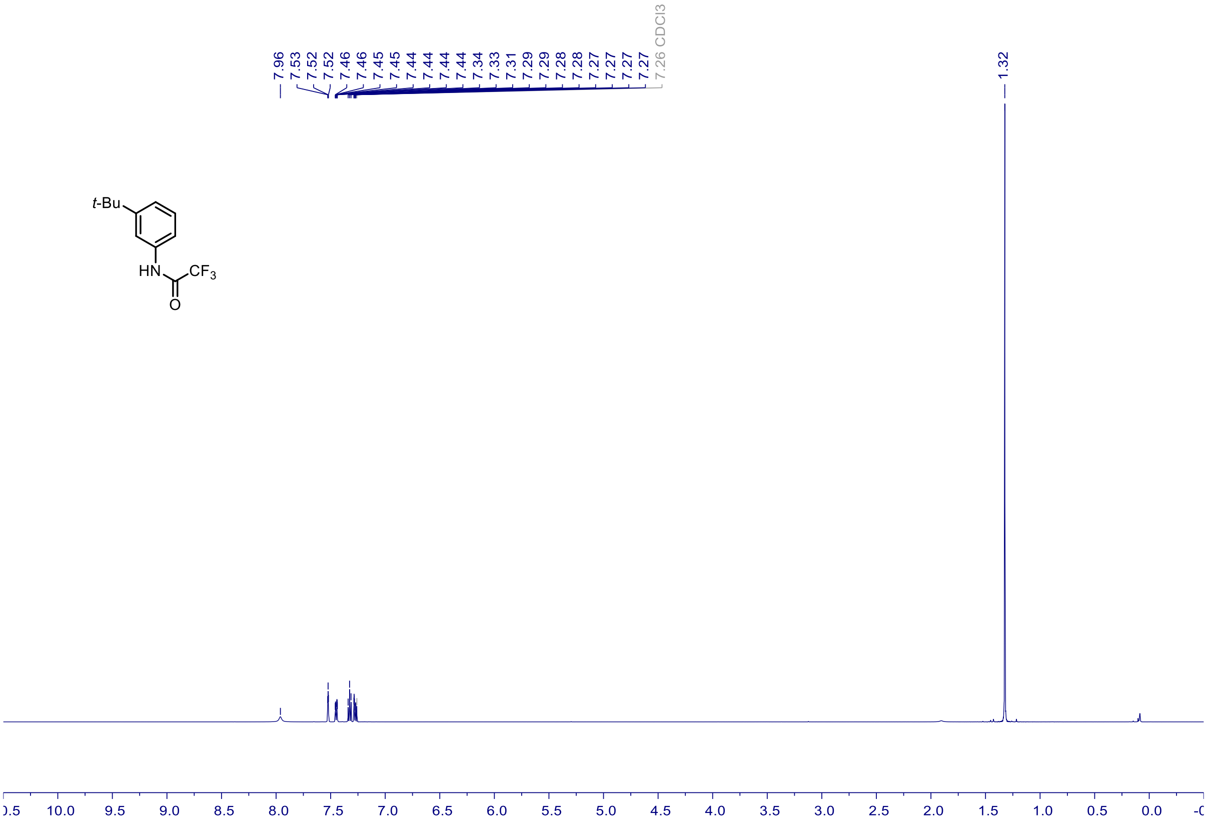

5a – ^{13}C NMR (151 MHz, $CDCl_3$)

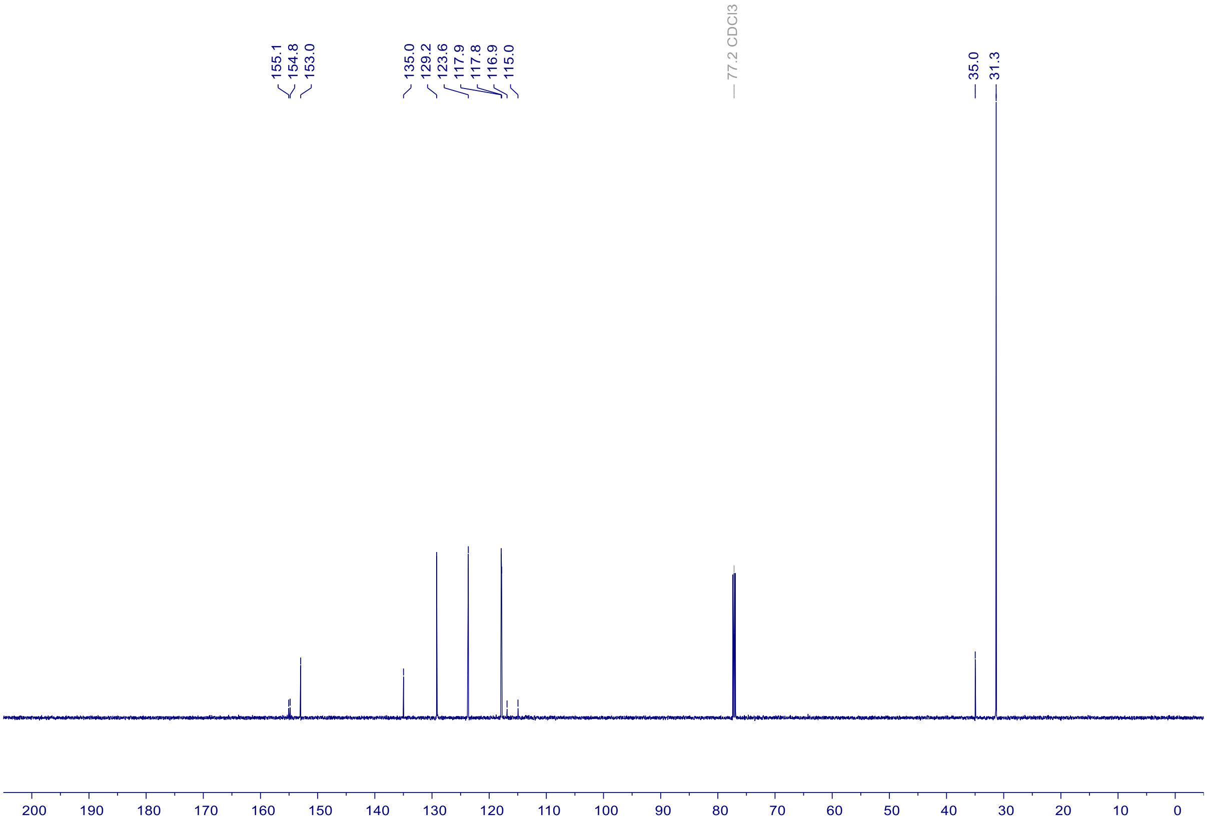

5a – ^{19}F NMR (565 MHz, $CDCl_3$)

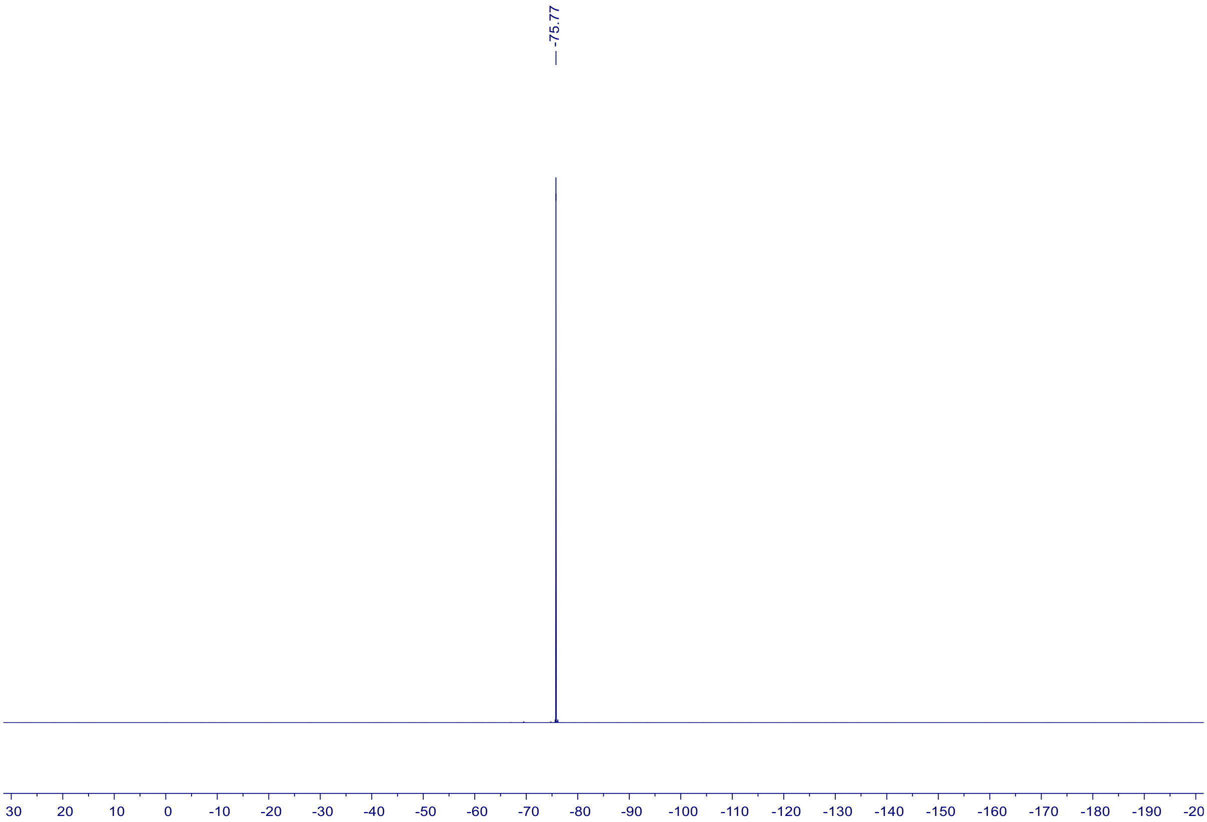

3ab – ¹H NMR (600 MHz, $CDCl_3$)

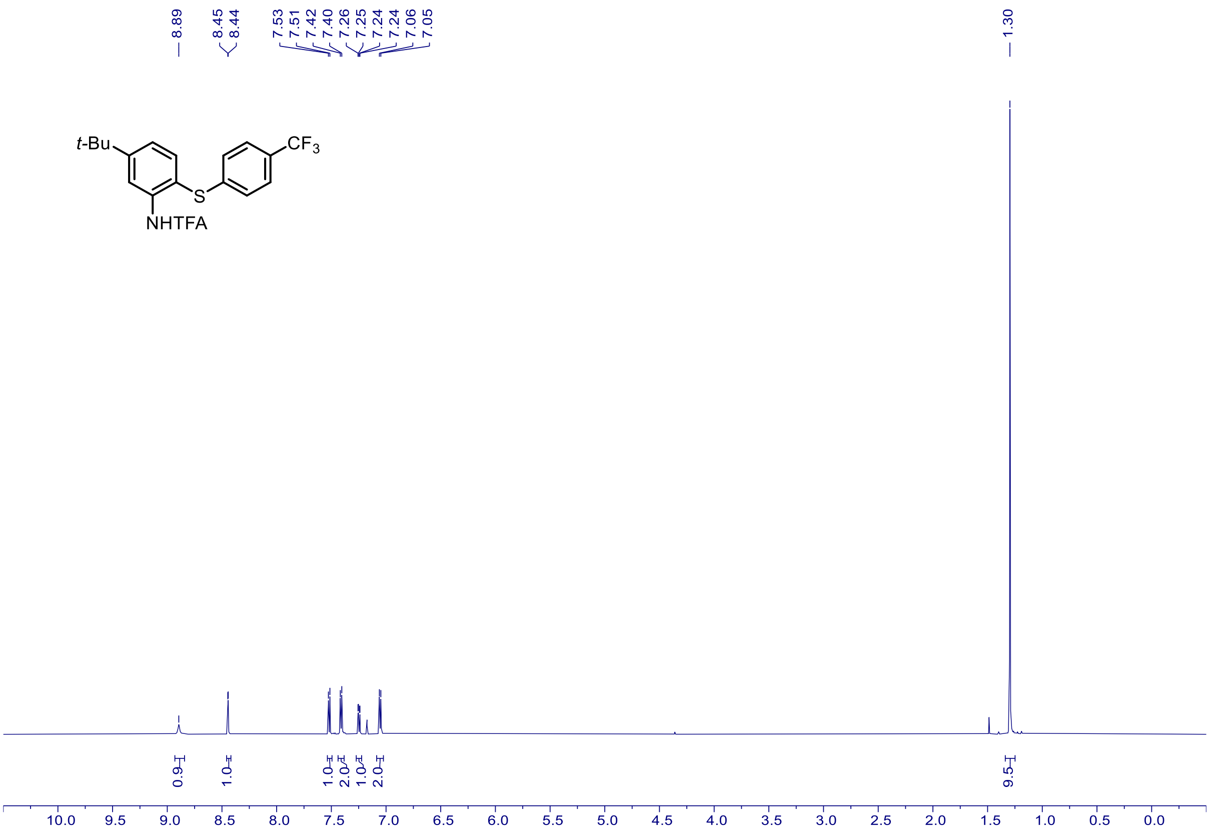

3ab – ¹³C NMR (151 MHz, $CDCl_3$)

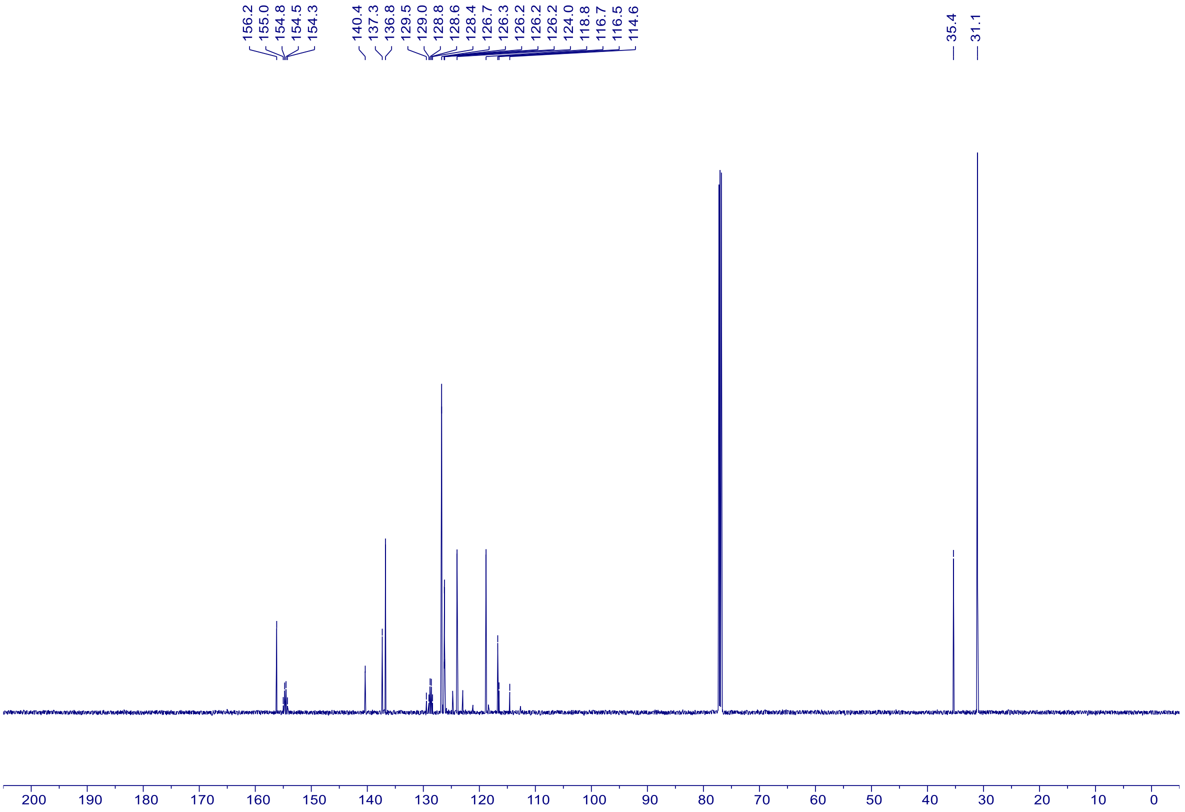

3ab – ^{19}F NMR (151 MHz, $CDCl_3$)

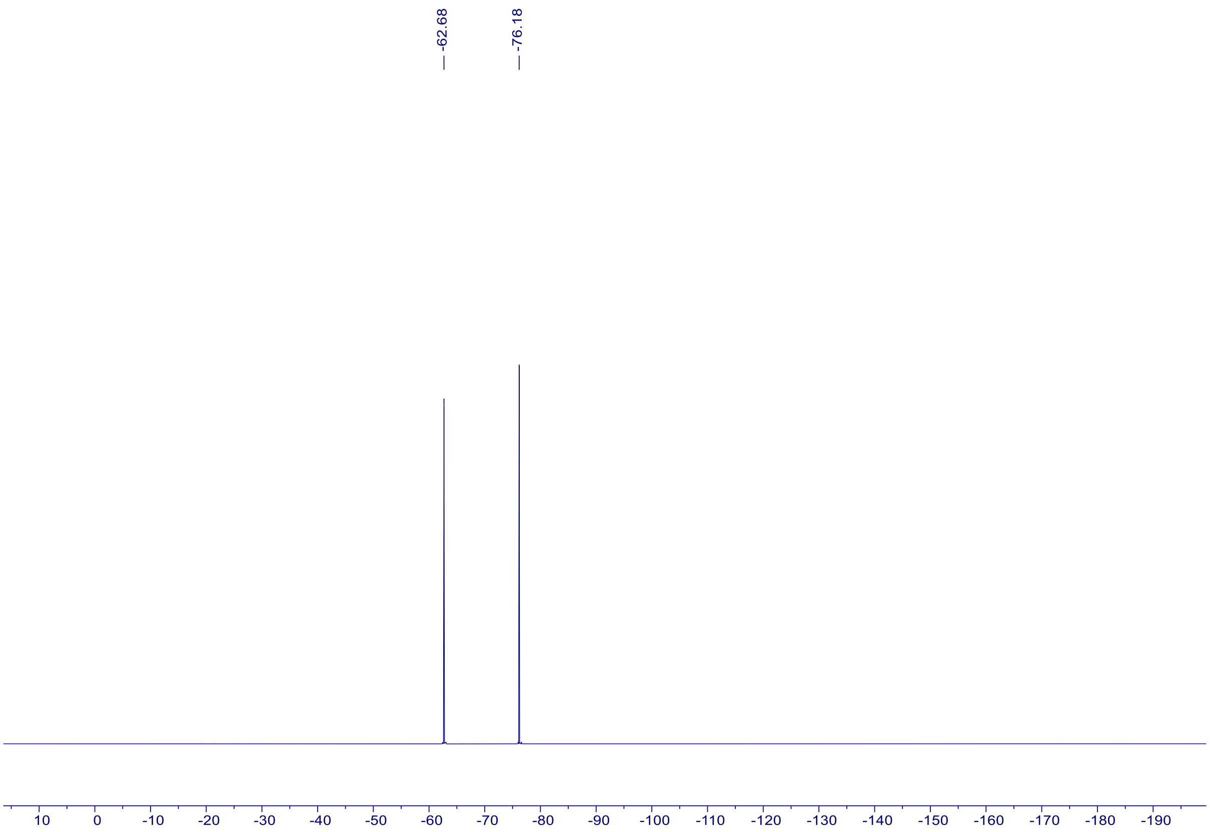

3ad – ^{1}H NMR (600 MHz, $CDCl_3$)

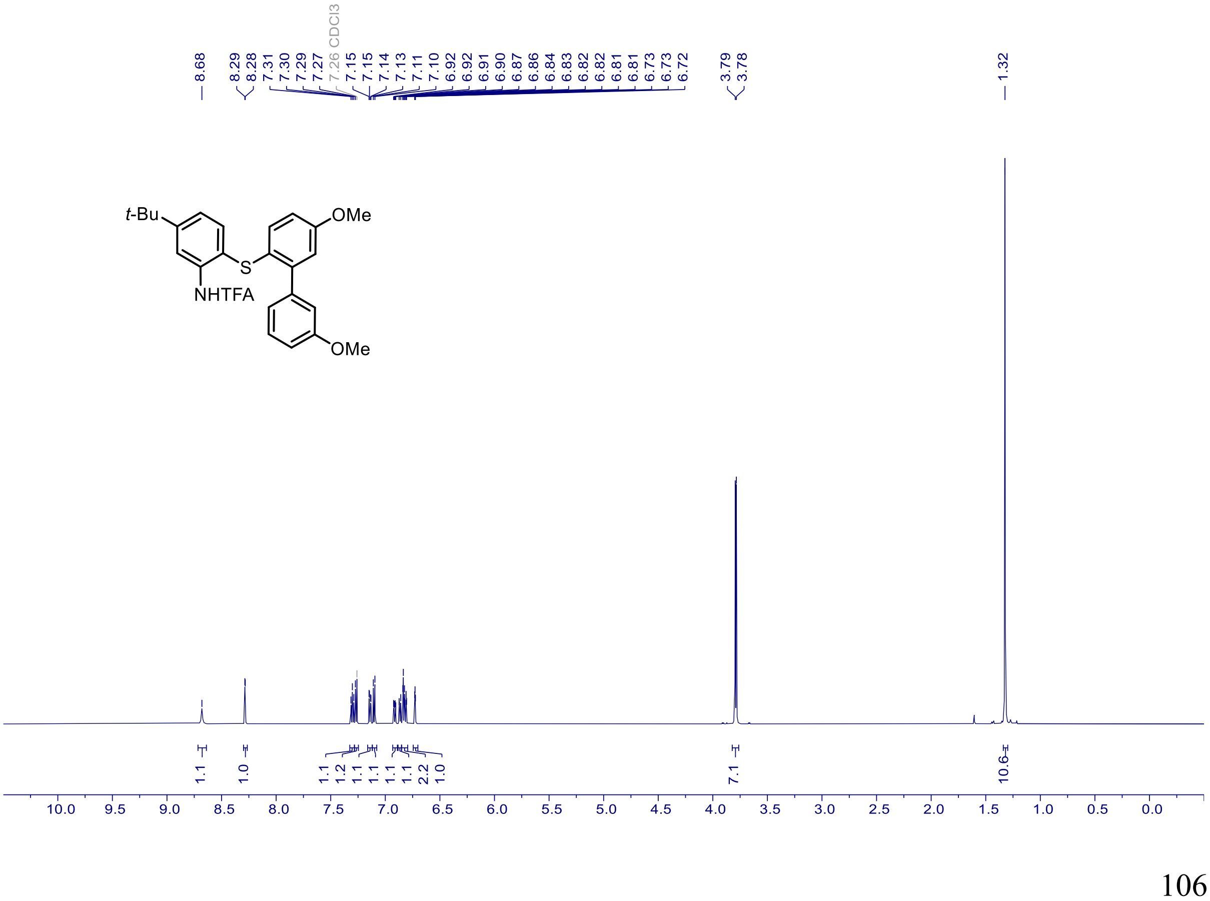

3ad – ^{13}C NMR (151 MHz, $CDCl_3$)

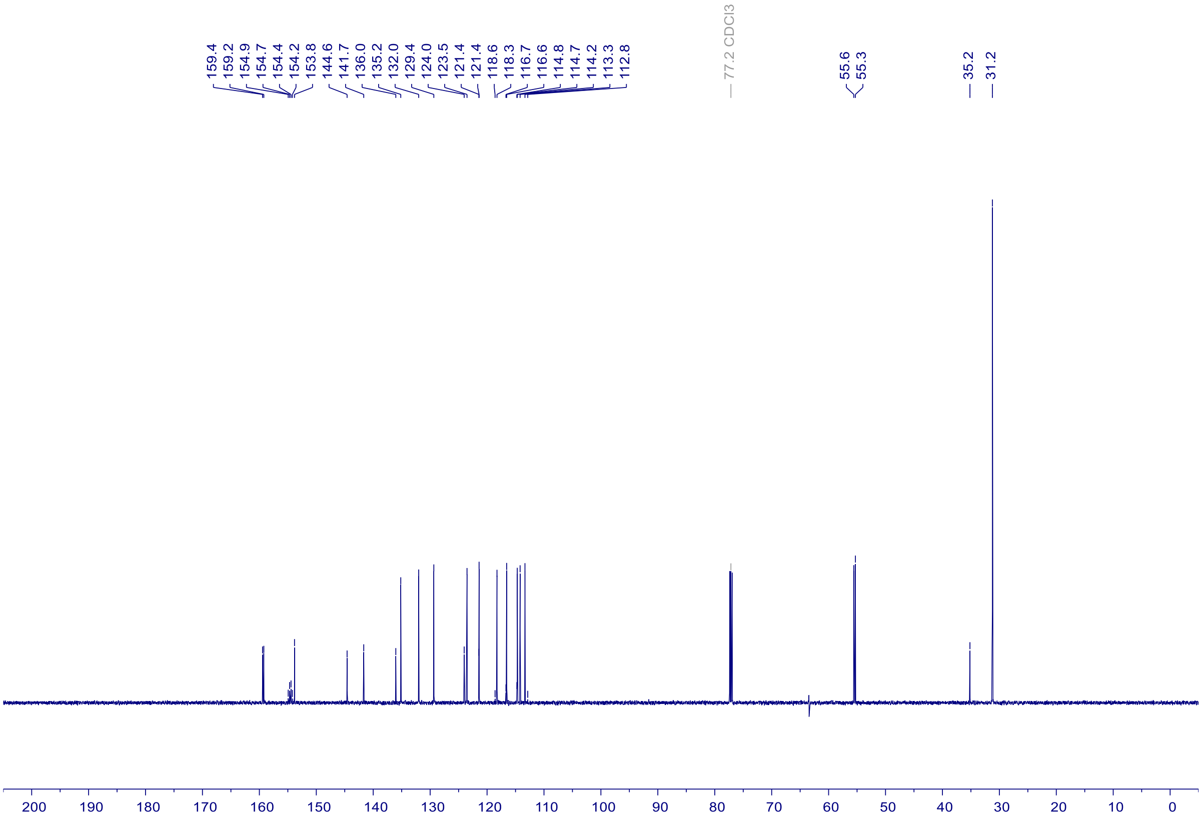

3ad – ^{19}F NMR (565 MHz, $CDCl_3$)

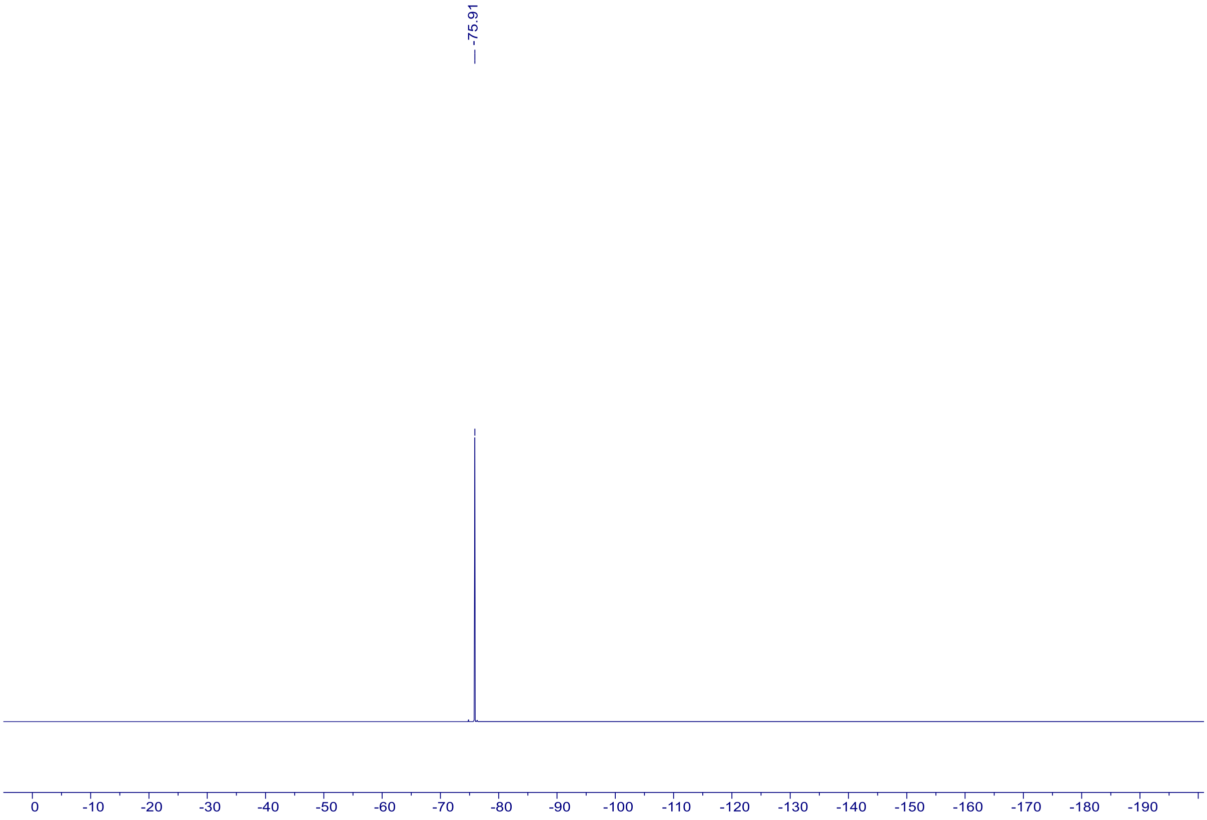

3ae – ^{1}H NMR (600 MHz, $CDCl_3$)

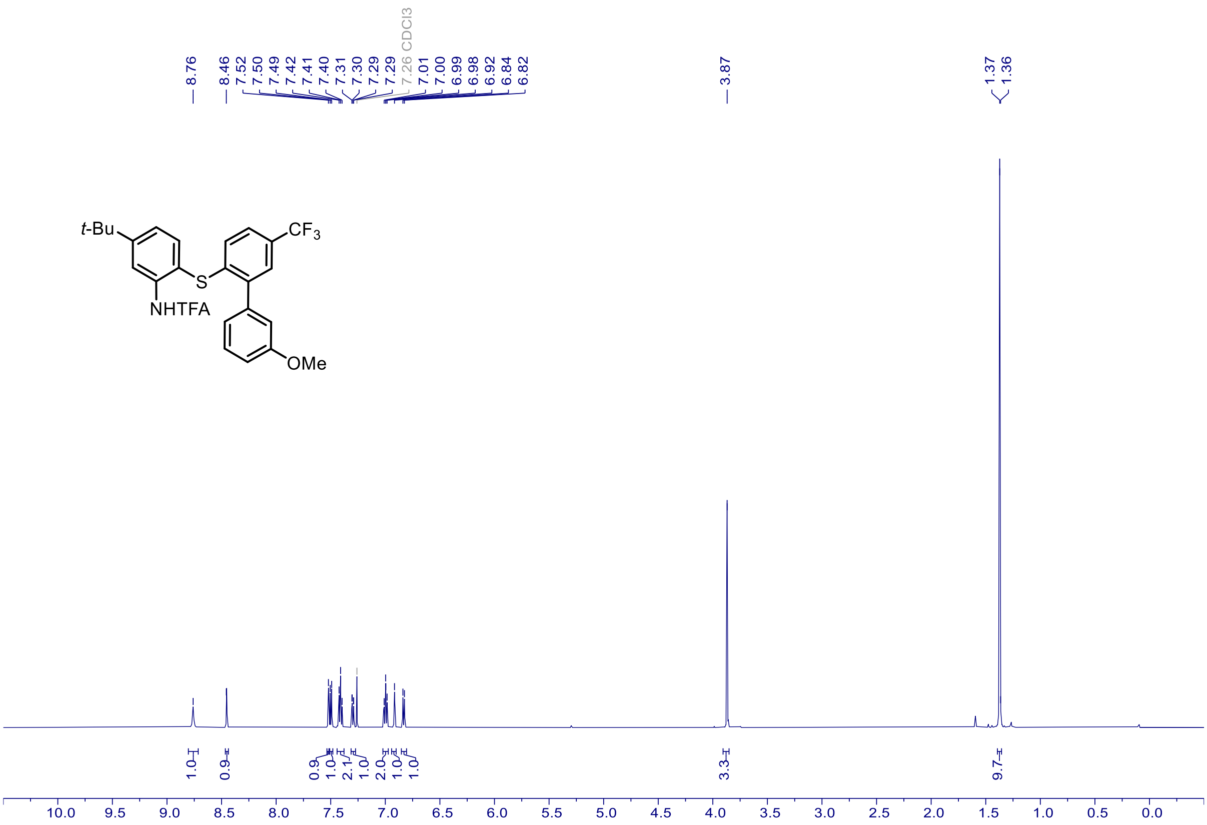

3ae – ^{13}C NMR (151 MHz, $CDCl_3$)

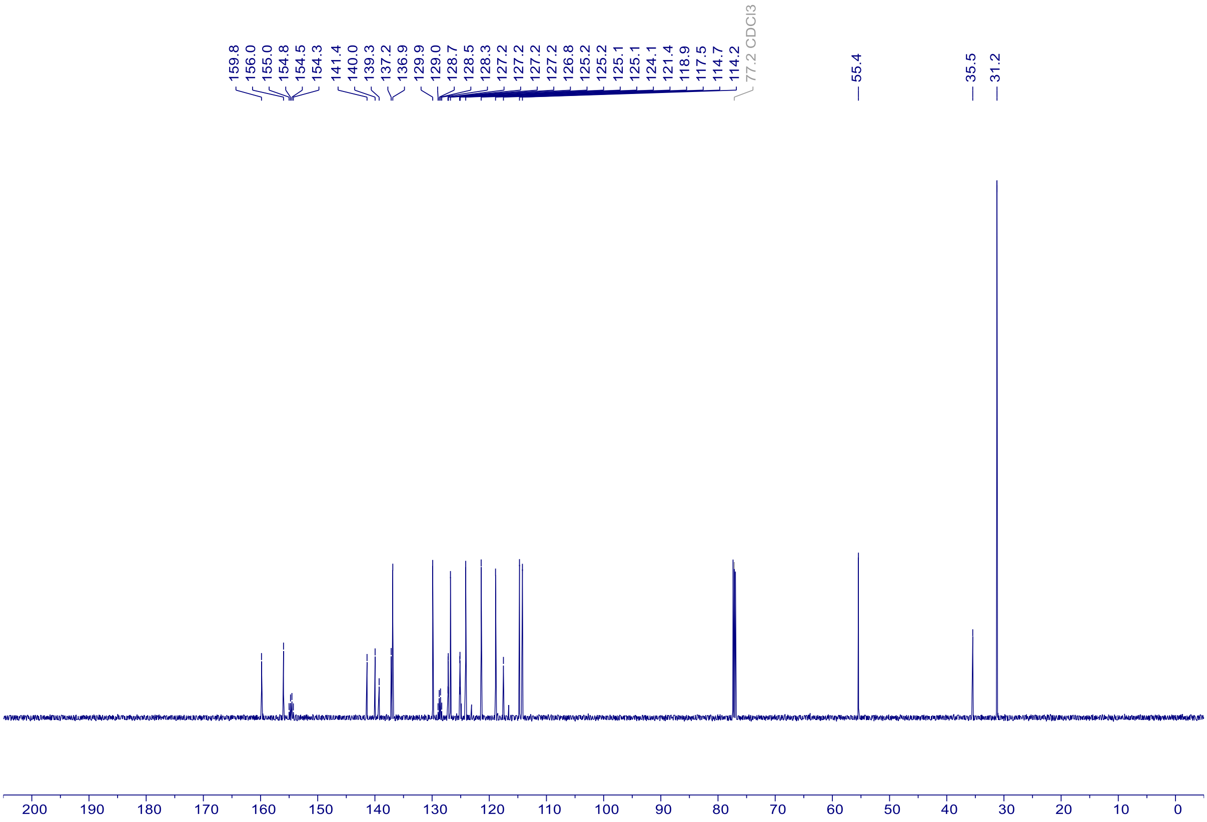

3ae – ^{19}F NMR (565 MHz, $CDCl_3$)

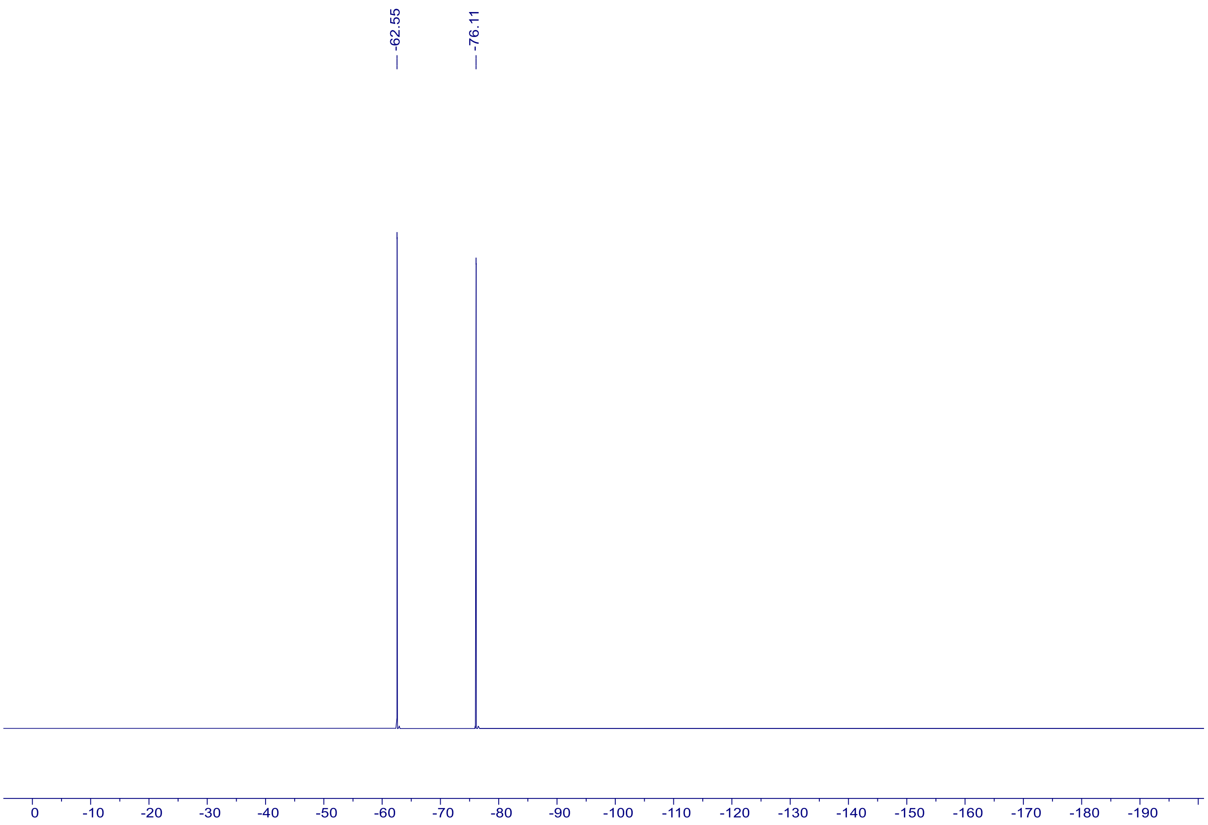

3af – ^{1}H NMR (600 MHz, $CDCl_3$)

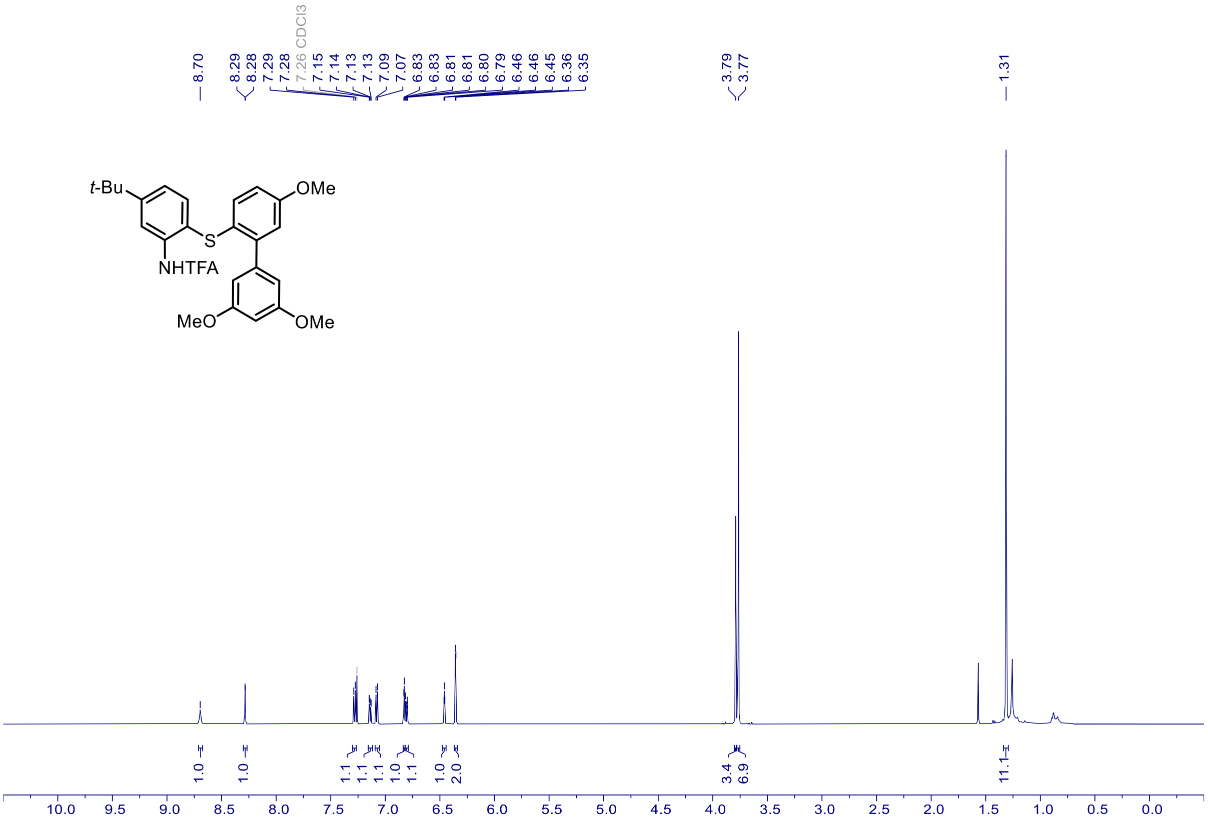

3af – ^{13}C NMR (151 MHz, $CDCl_3$)

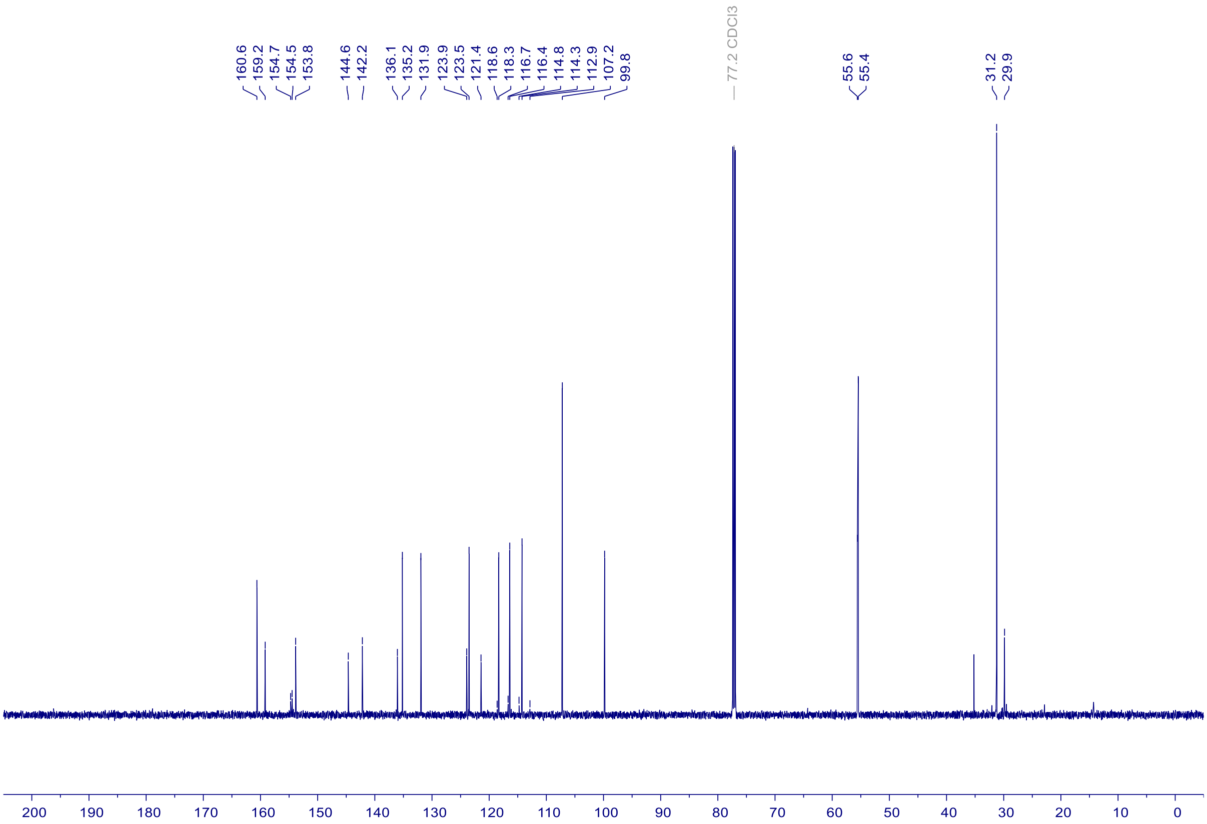

3af – ^{19}F NMR (565 MHz, $CDCl_3$)

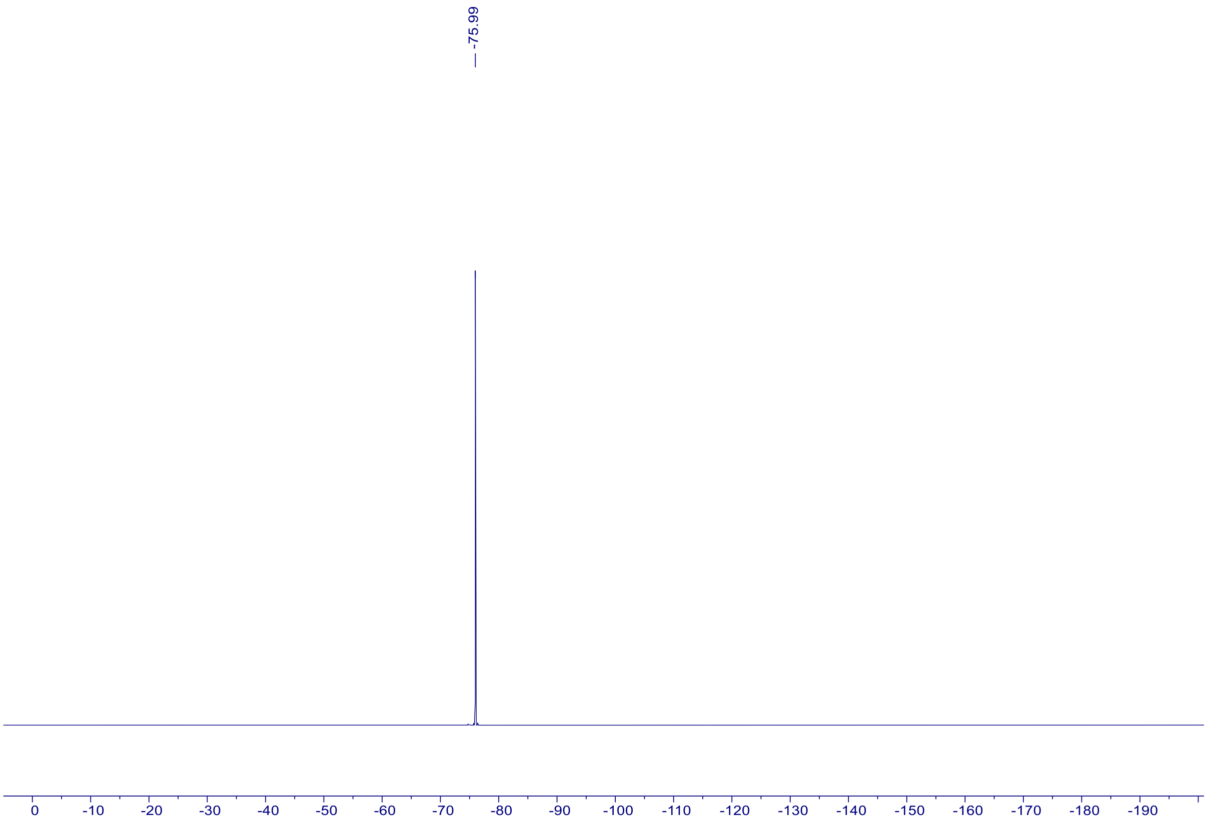

3ag – ^{1}H NMR (600 MHz, CDCl3)

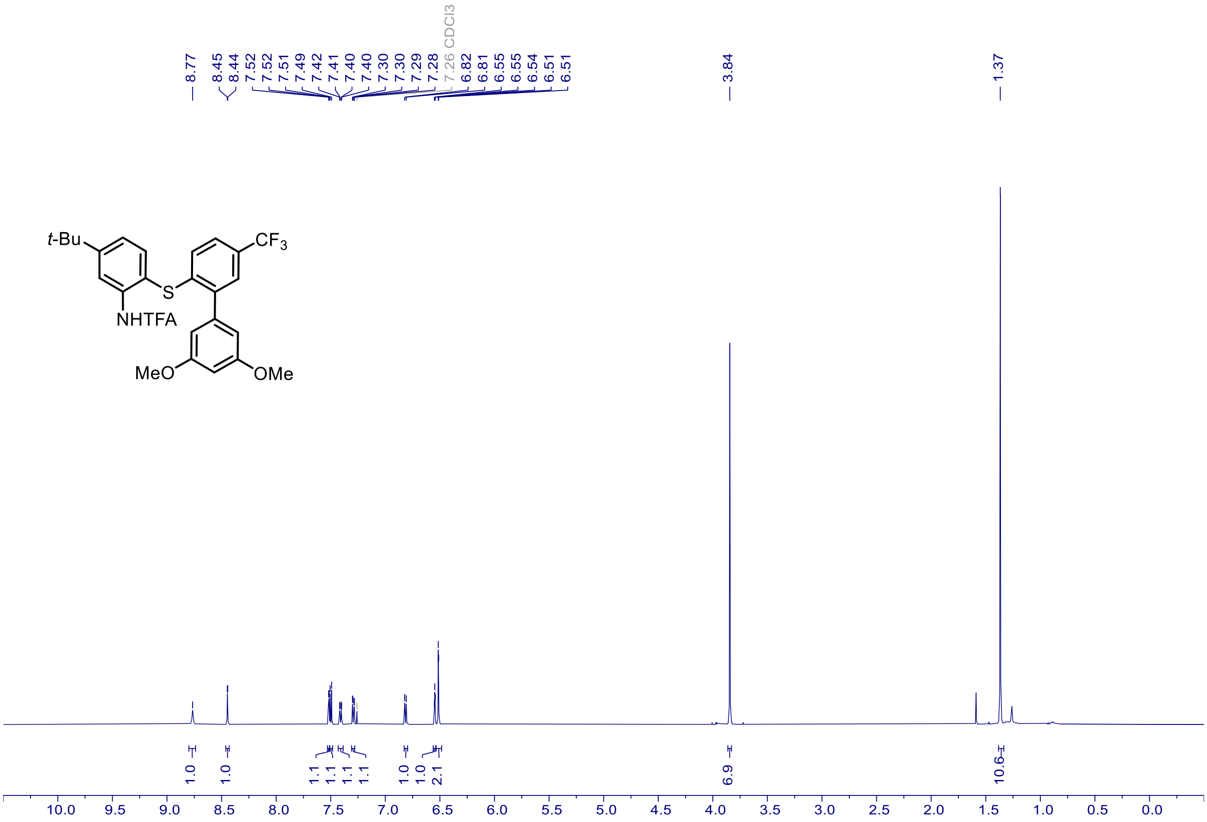

3ag – ^{13}C NMR (151 MHz, CDCl3)

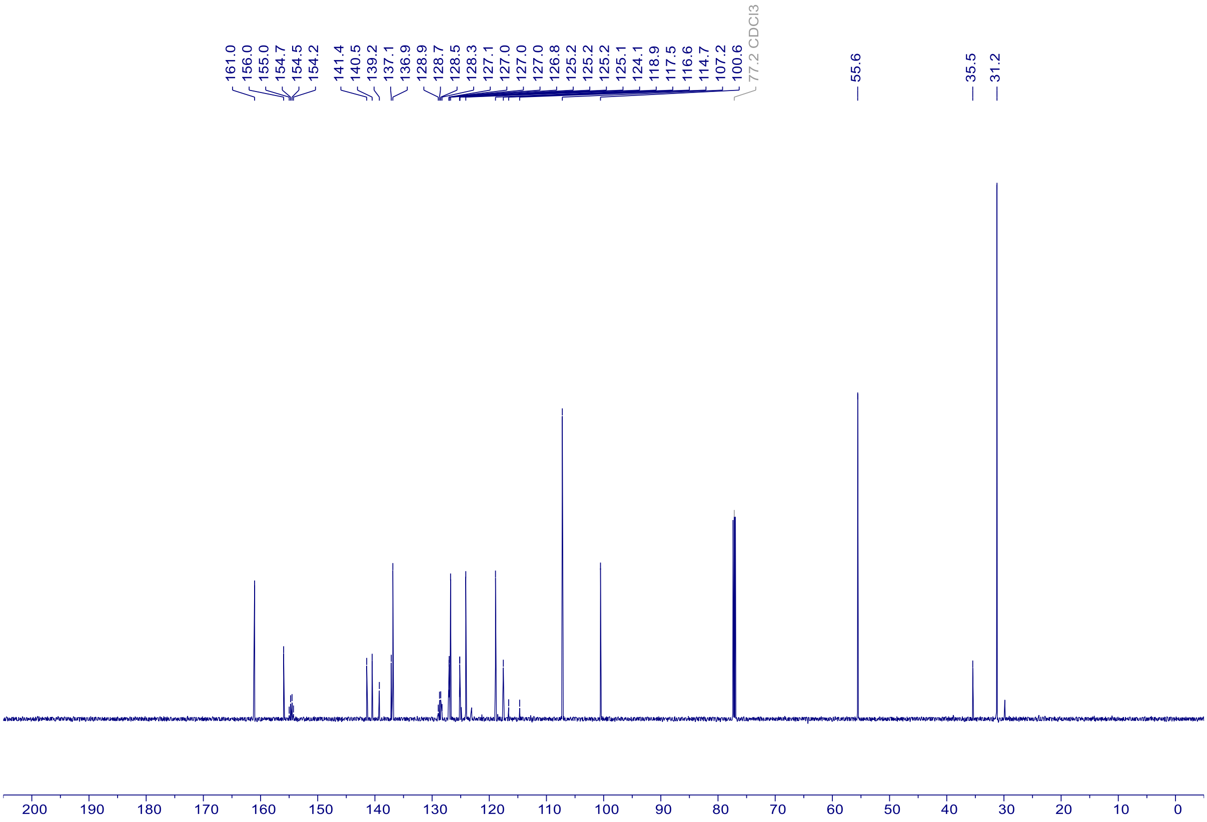

3ag – ^{19}F NMR (565 MHz, $CDCl_3$)

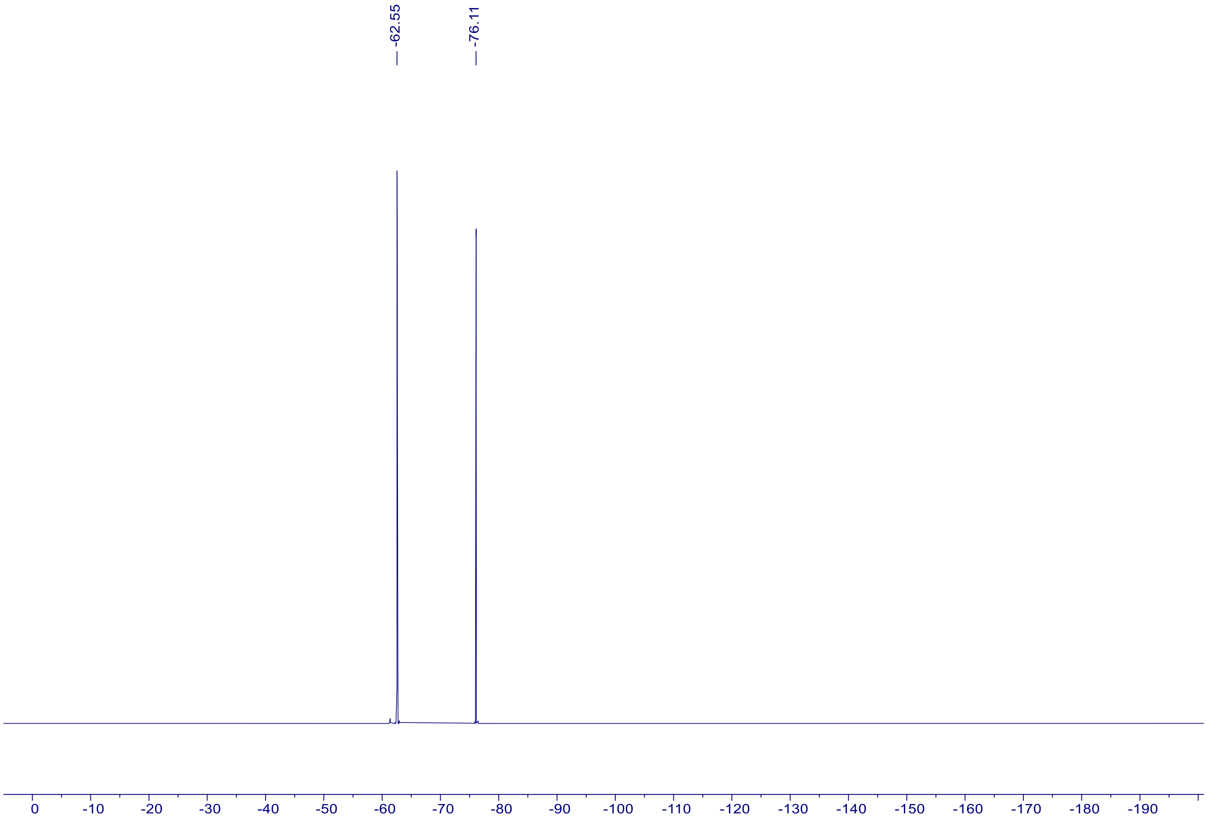

1a – ^{1}H NMR (600 MHz, $CDCl_3$)

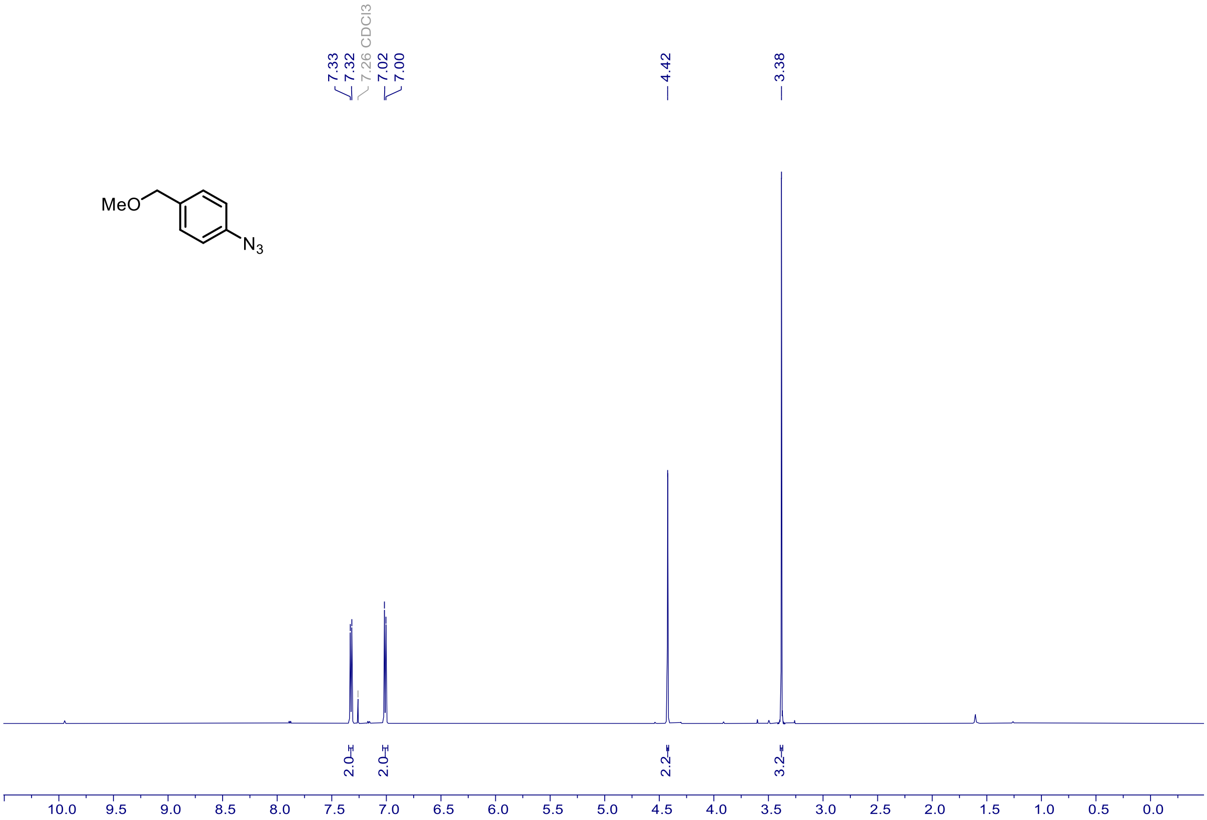

1a – ^{13}C NMR (151 MHz, $CDCl_3$)

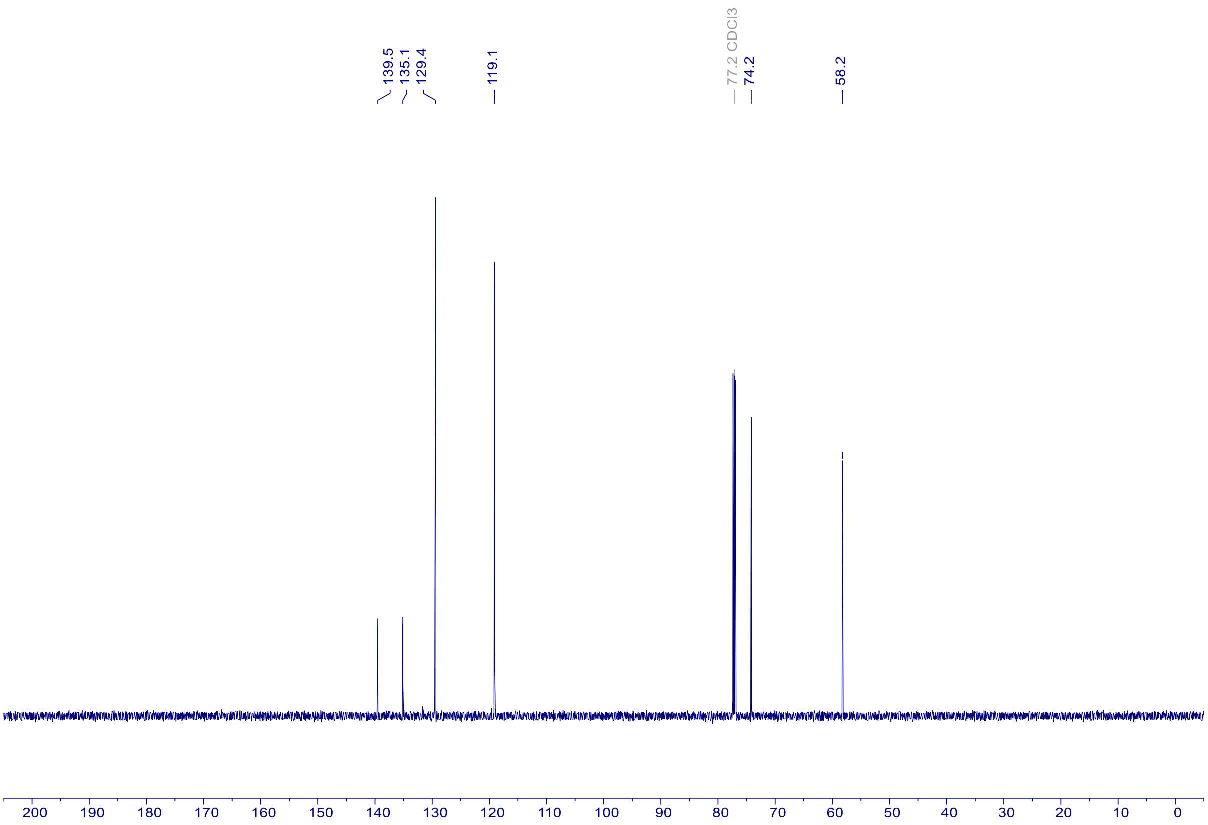

1j – ^{1}H NMR (600 MHz, $CDCl_3$)

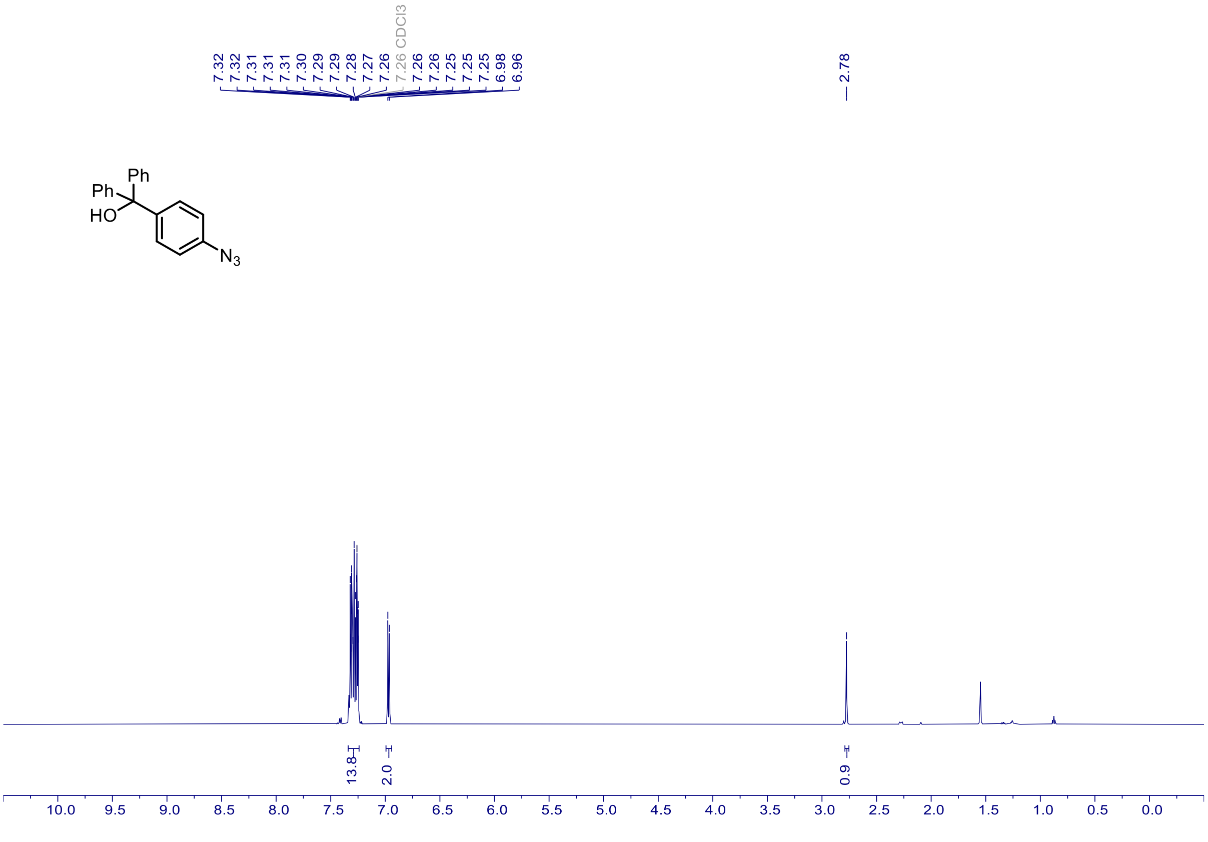

1j – ^{13}C NMR (151 MHz, $CDCl_3$)

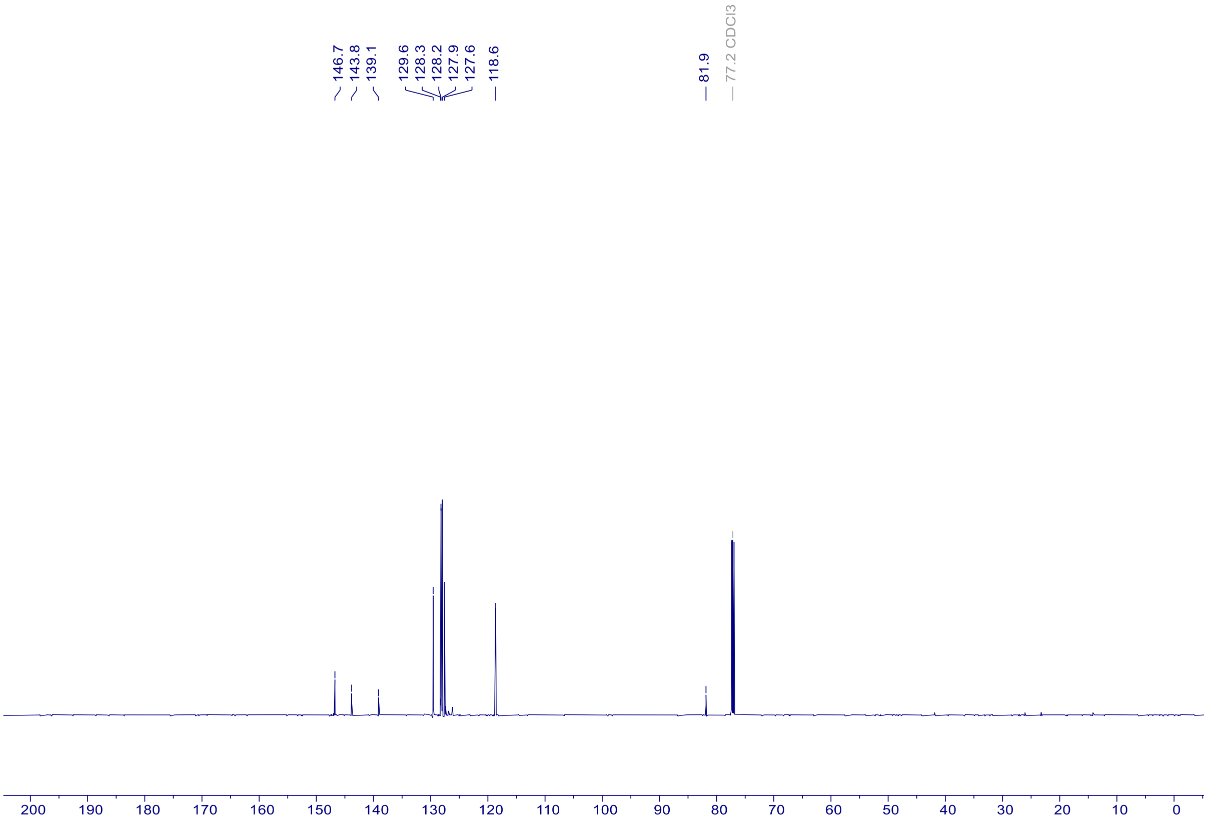

1o – ^{1}H NMR (600 MHz, $CDCl_3$)

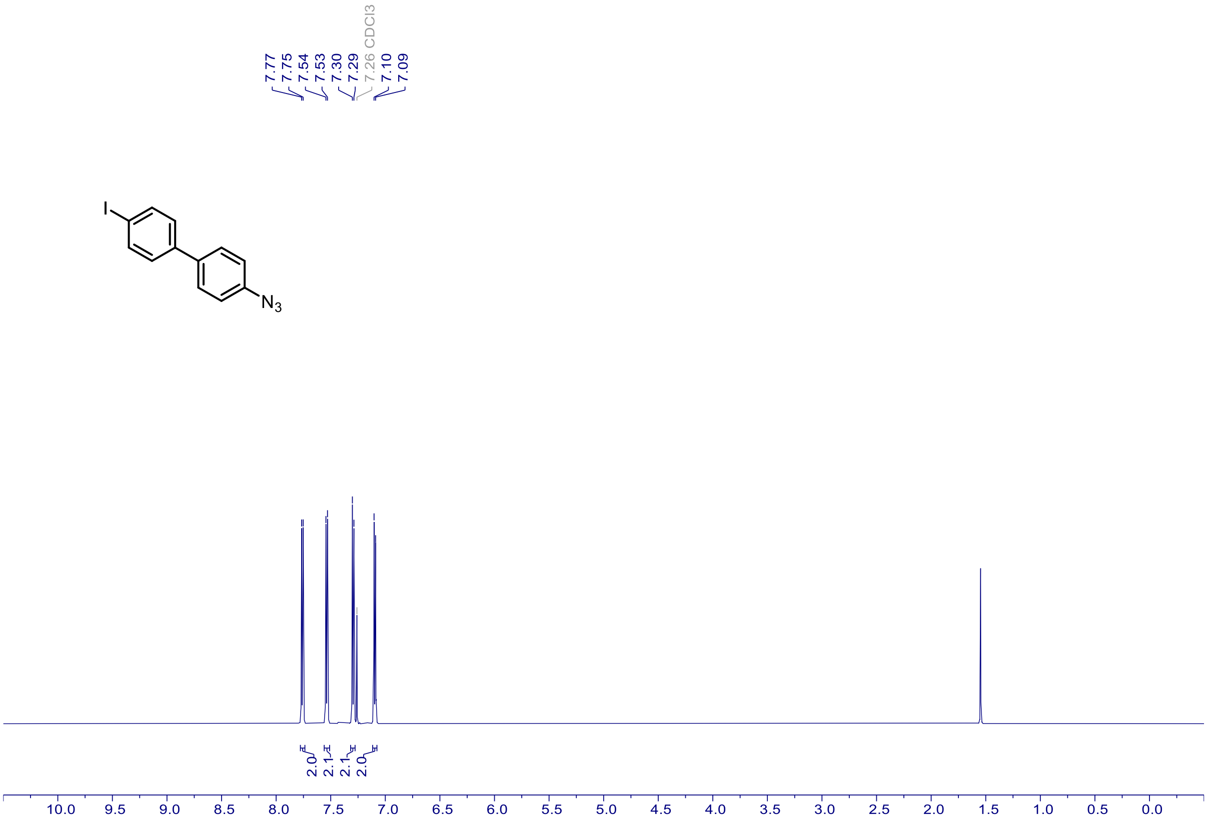

1o – ^{13}C NMR (151 MHz, $CDCl_3$)

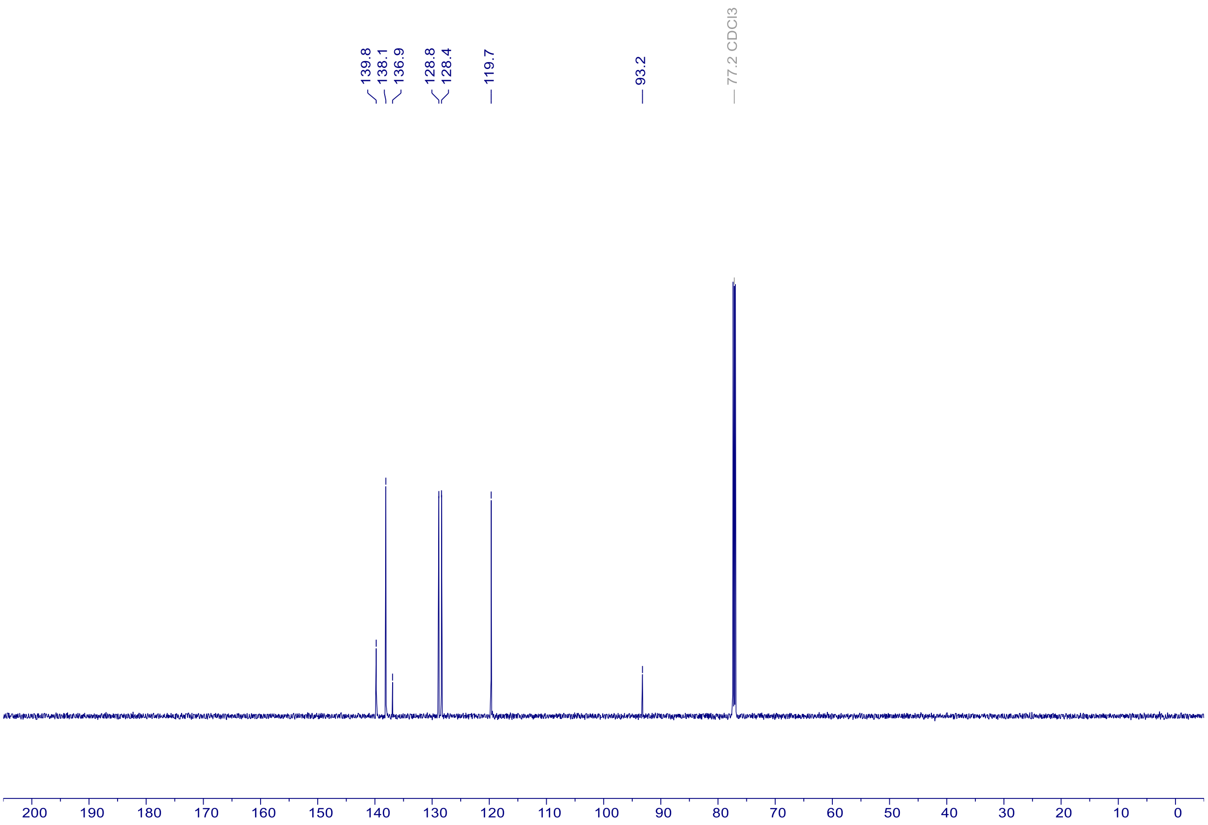

3bh – ^{1}H NMR (600 MHz, $CDCl_3$)

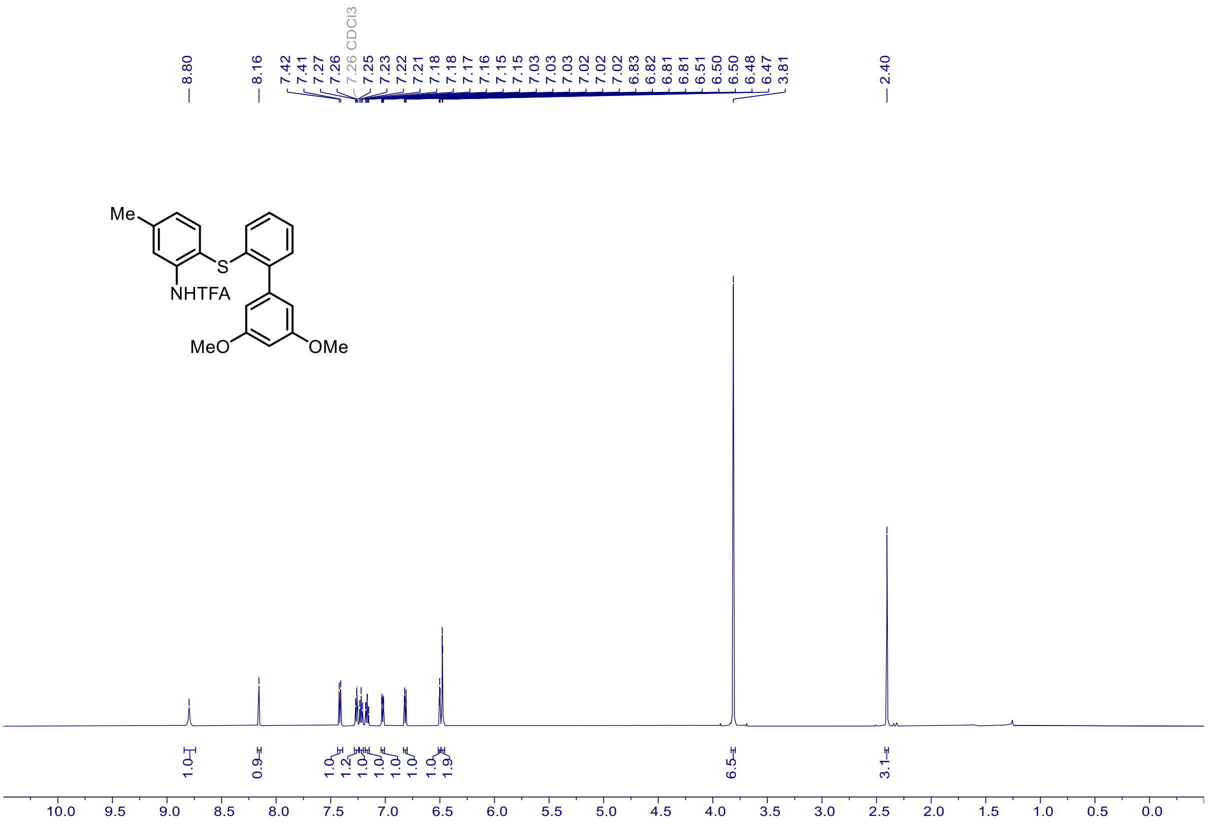

3bh – ^{13}C NMR (151 MHz, CDCl$_3$)

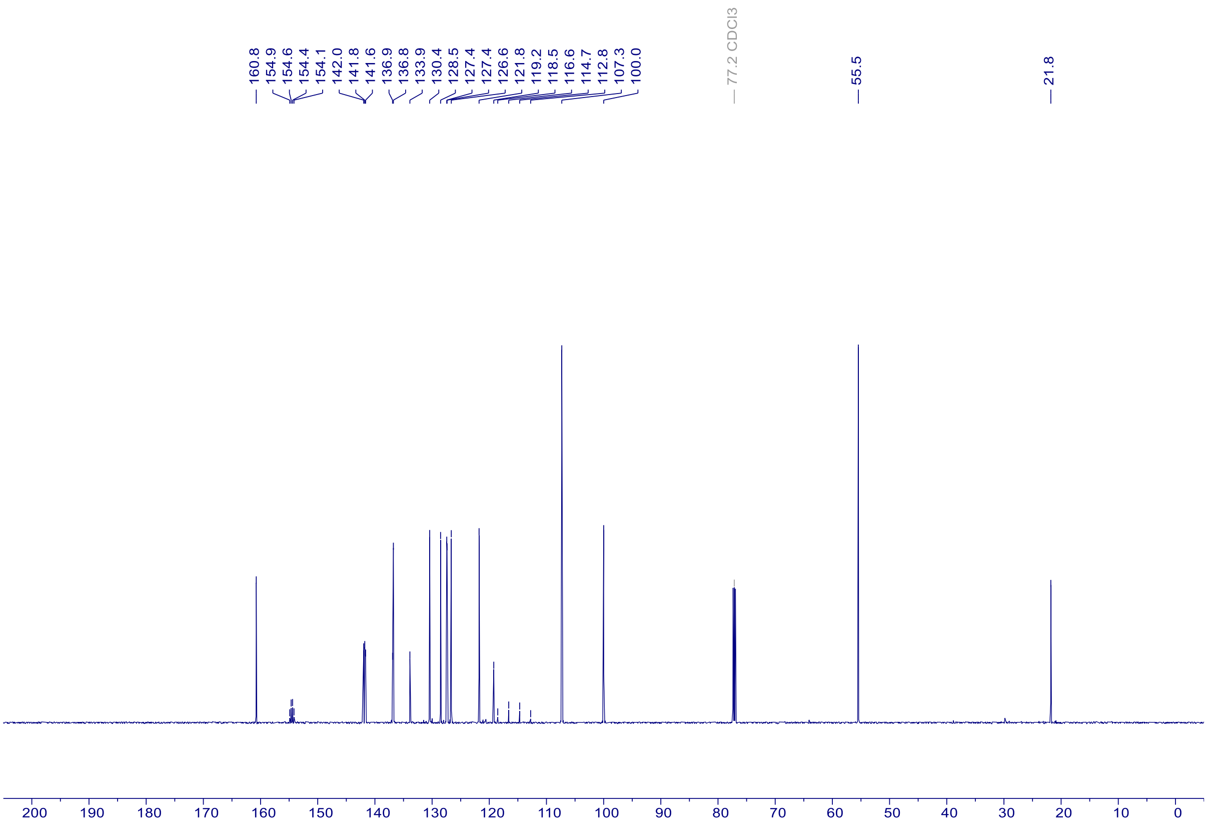

3bh – ^{19}F NMR (565 MHz, CDCl$_3$)

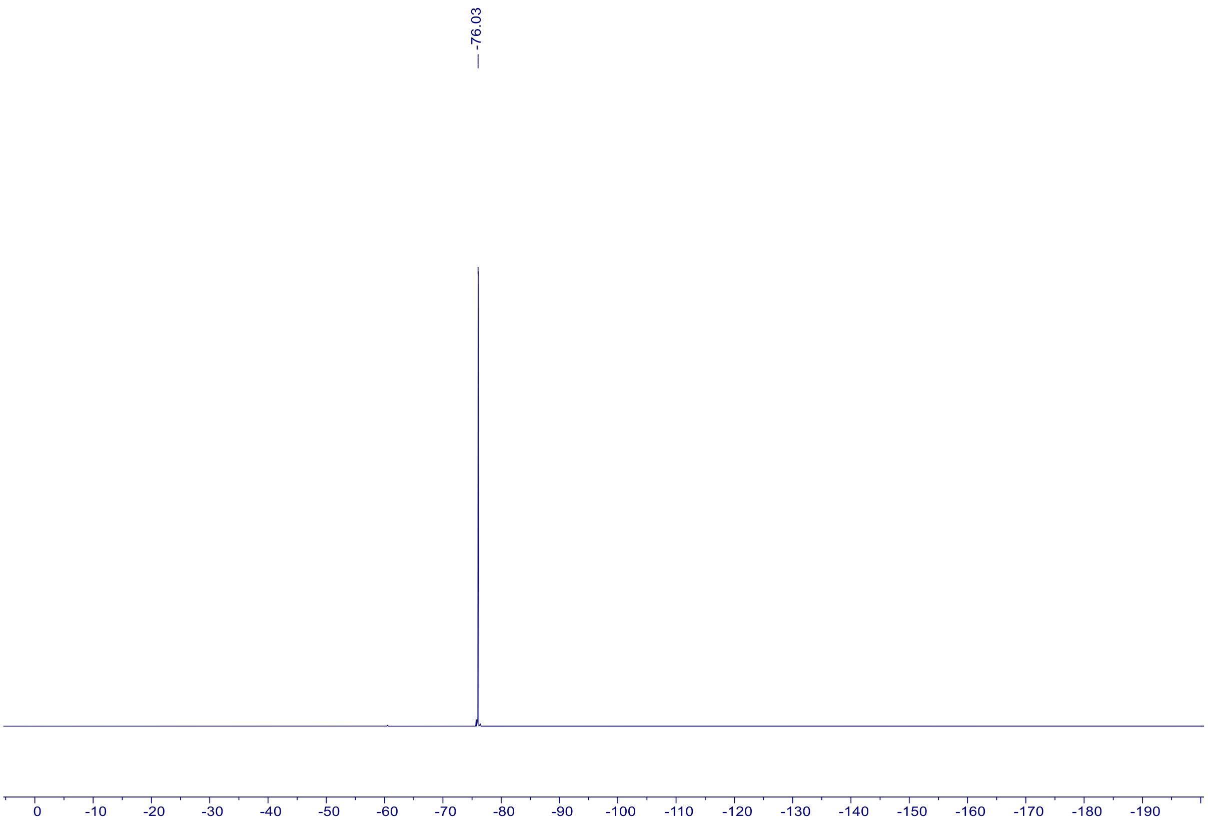

3ch – ^{1}H NMR (600 MHz, $CDCl_3$)

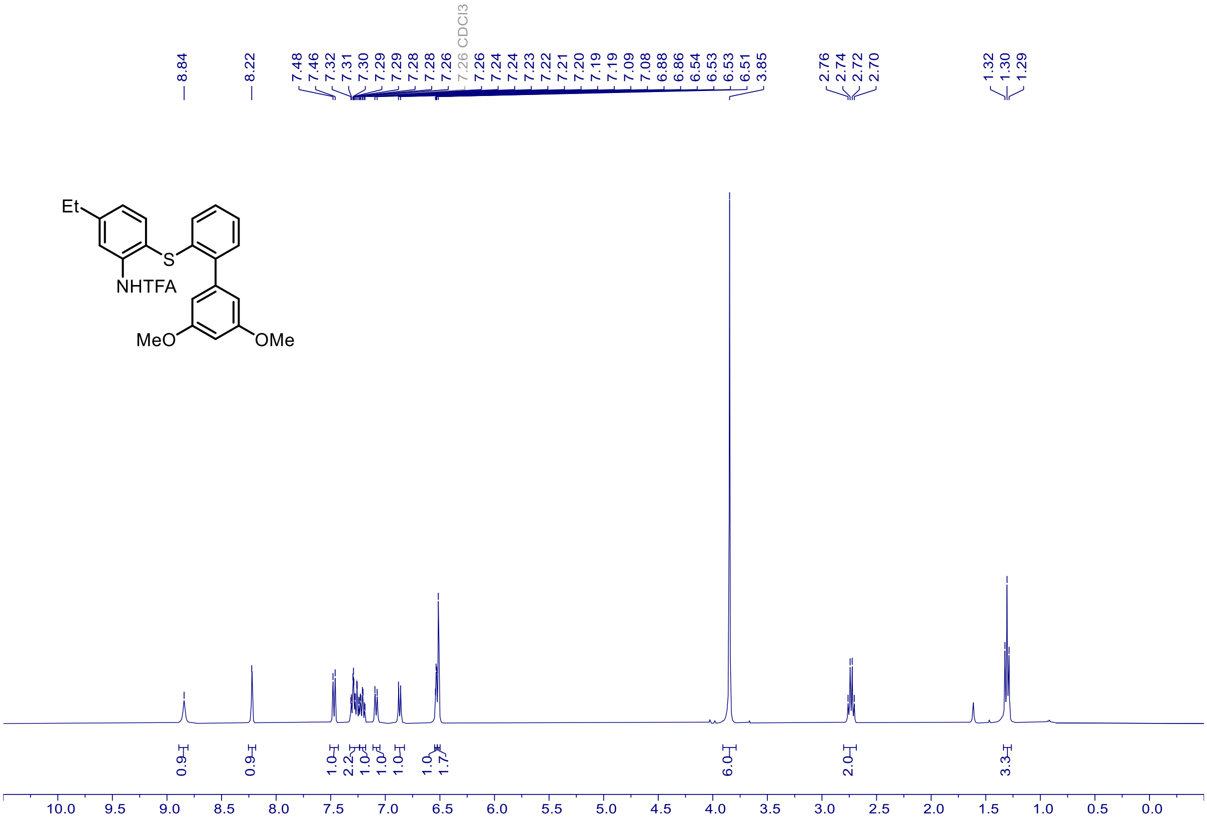

3ch – ^{13}C NMR (151 MHz, $CDCl_3$)

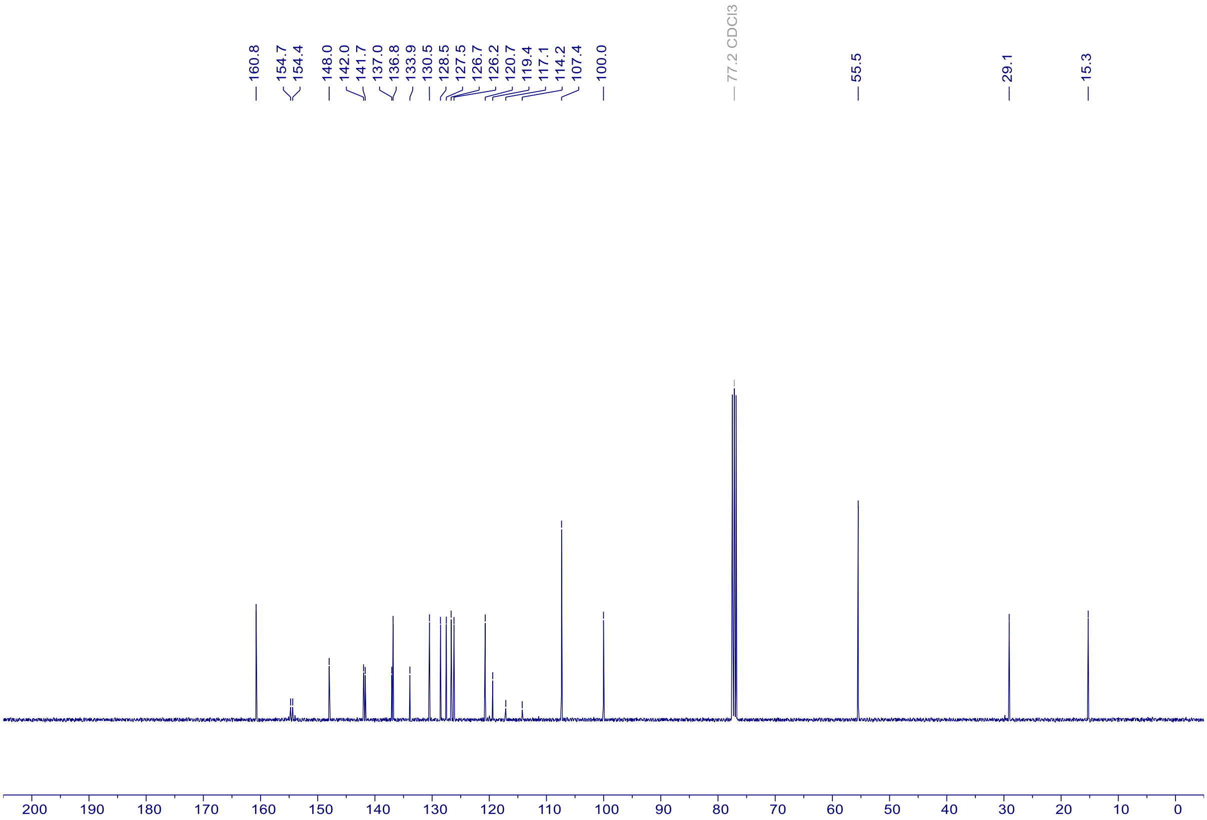

3ch – ^{19}F NMR (565 MHz, $CDCl_3$)

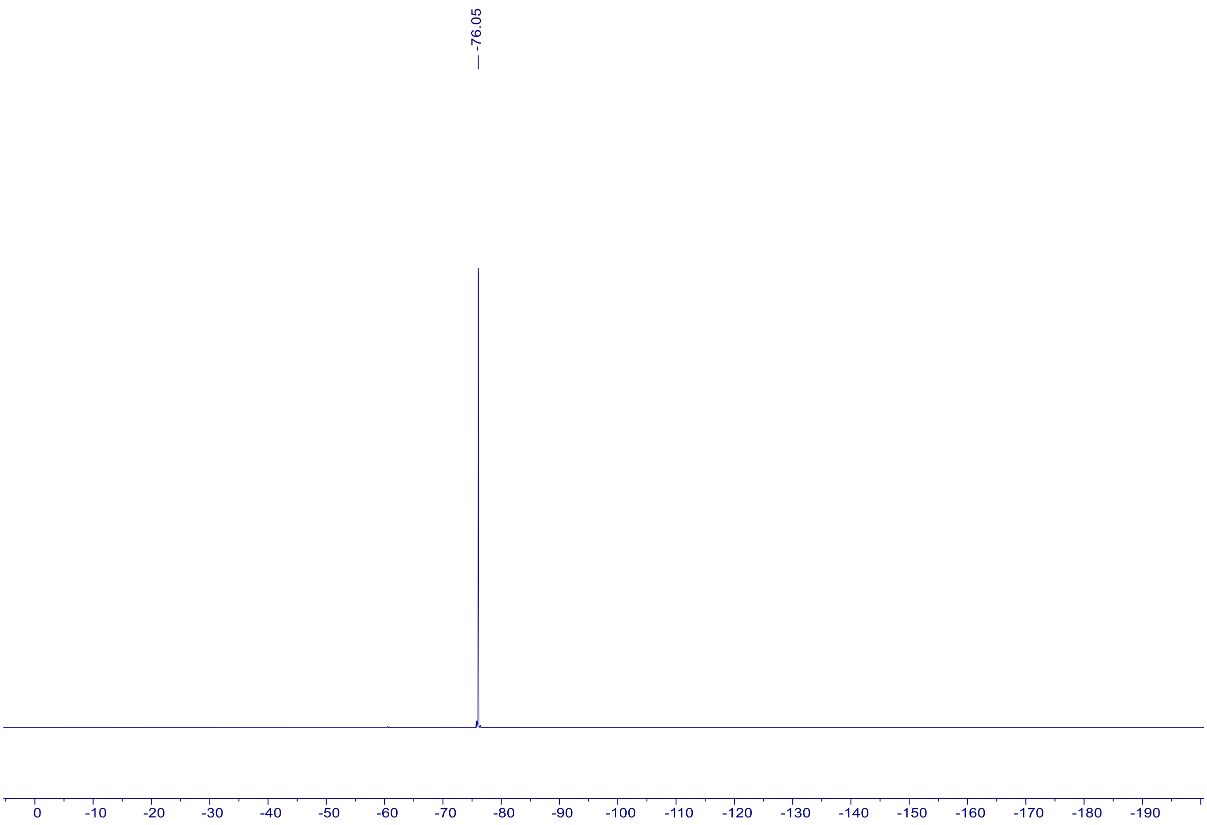

3dh – ^{1}H NMR (600 MHz, $CDCl_3$)

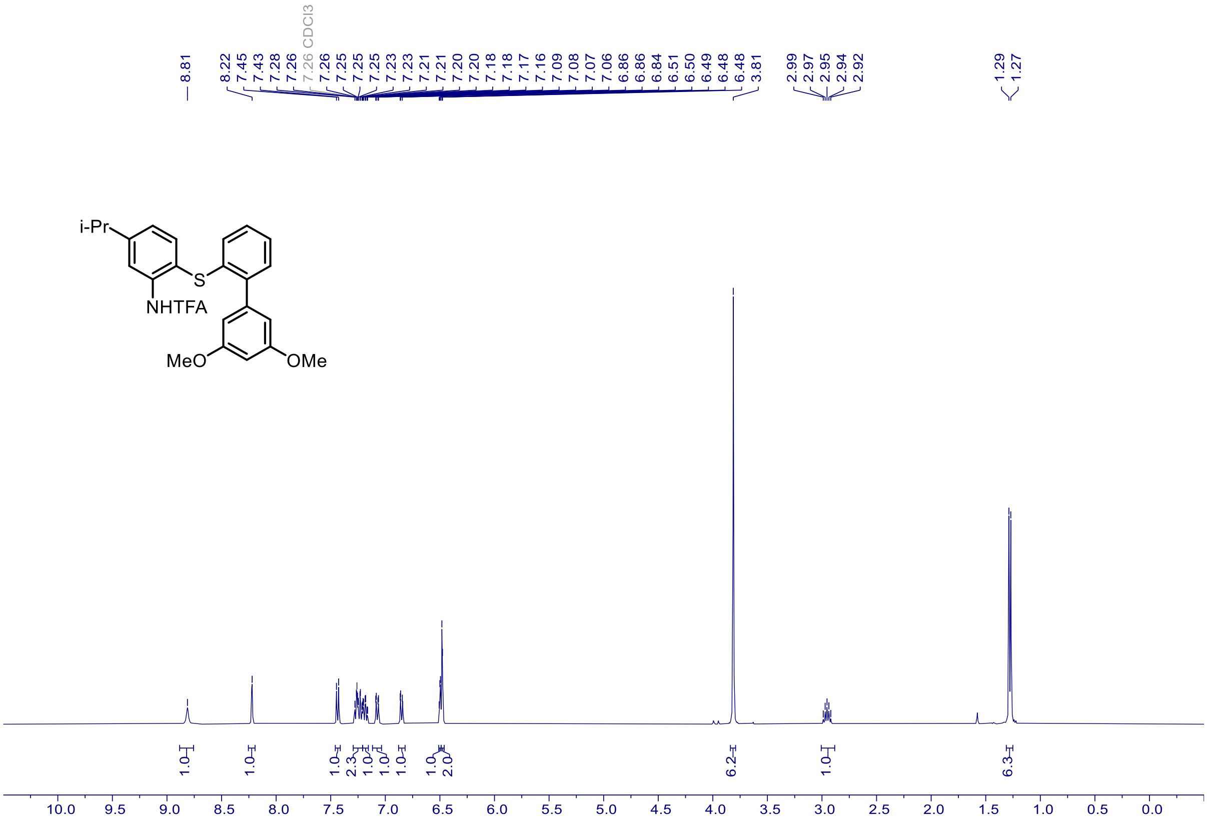

3dh – ^{13}C NMR (151 MHz, $CDCl_3$)

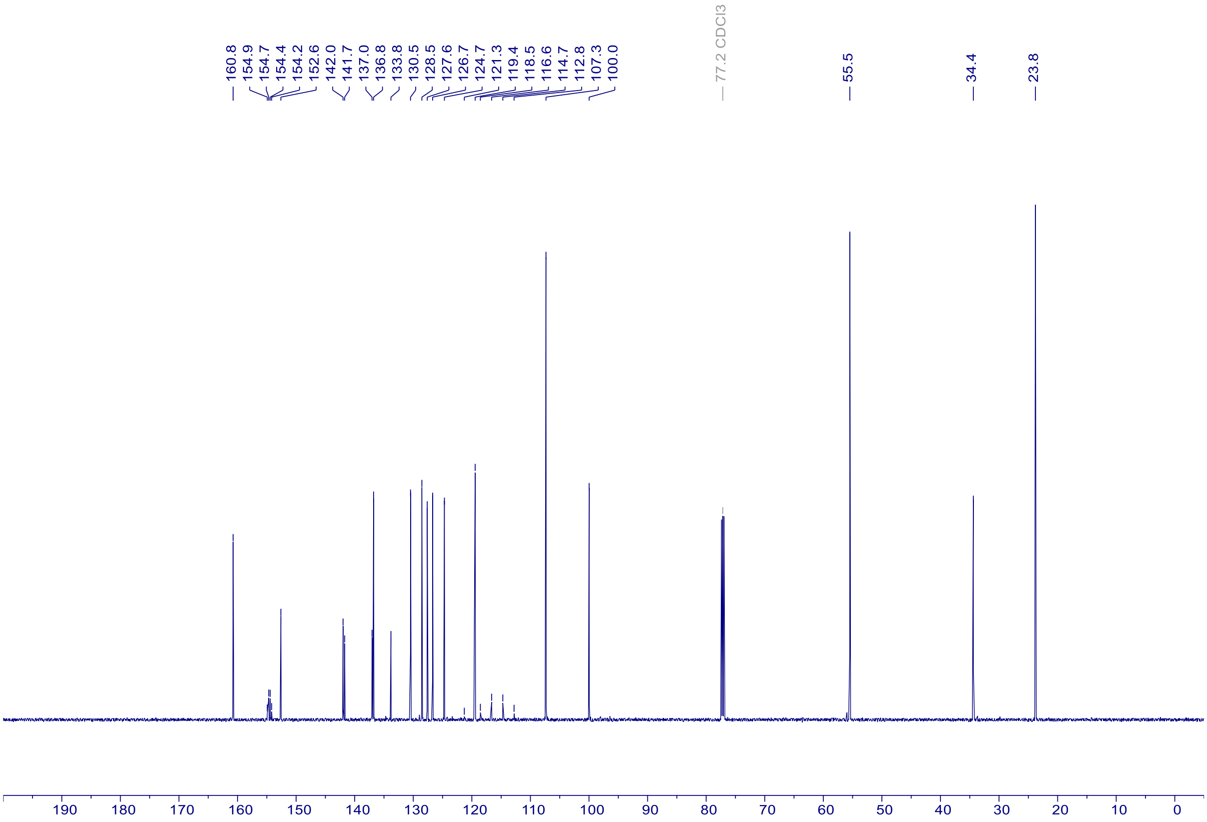

3dh – ^{19}F NMR (565 MHz, $CDCl_3$)

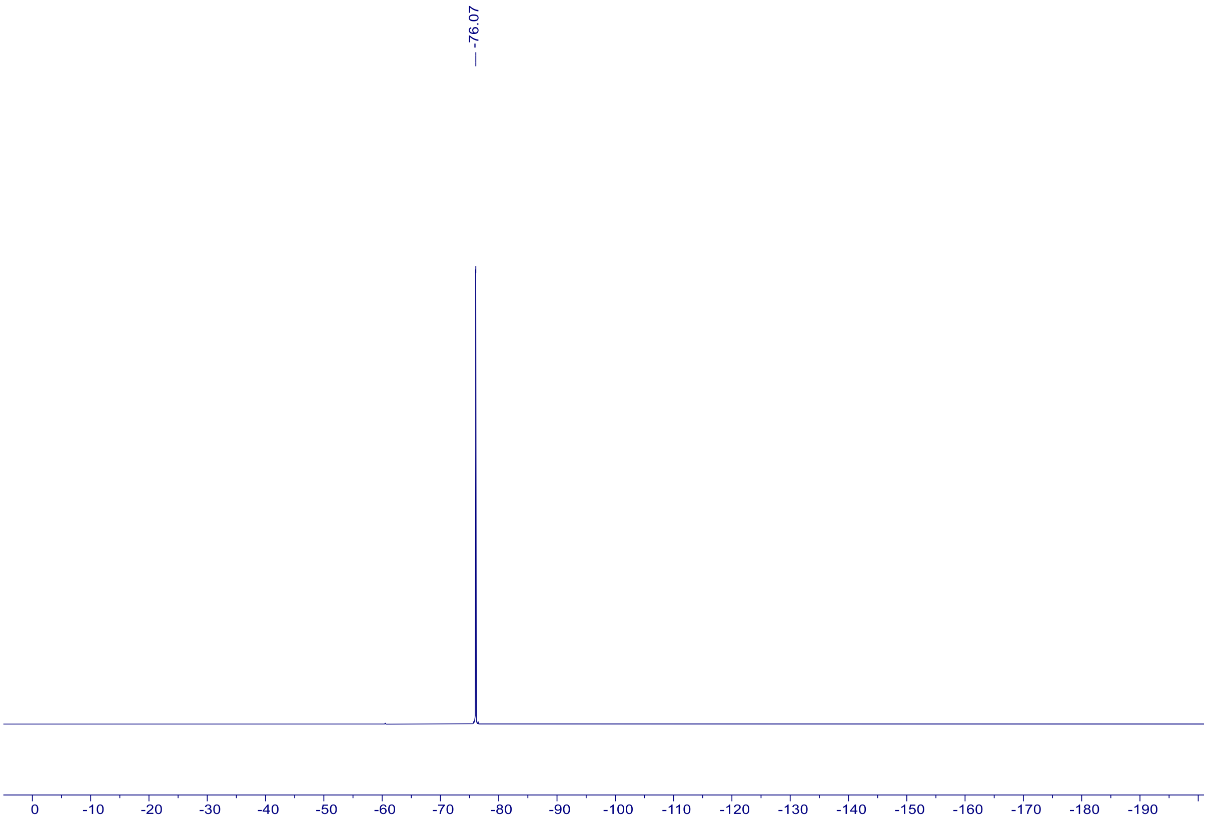

3eh – ^{1}H NMR (600 MHz, $CDCl_3$)

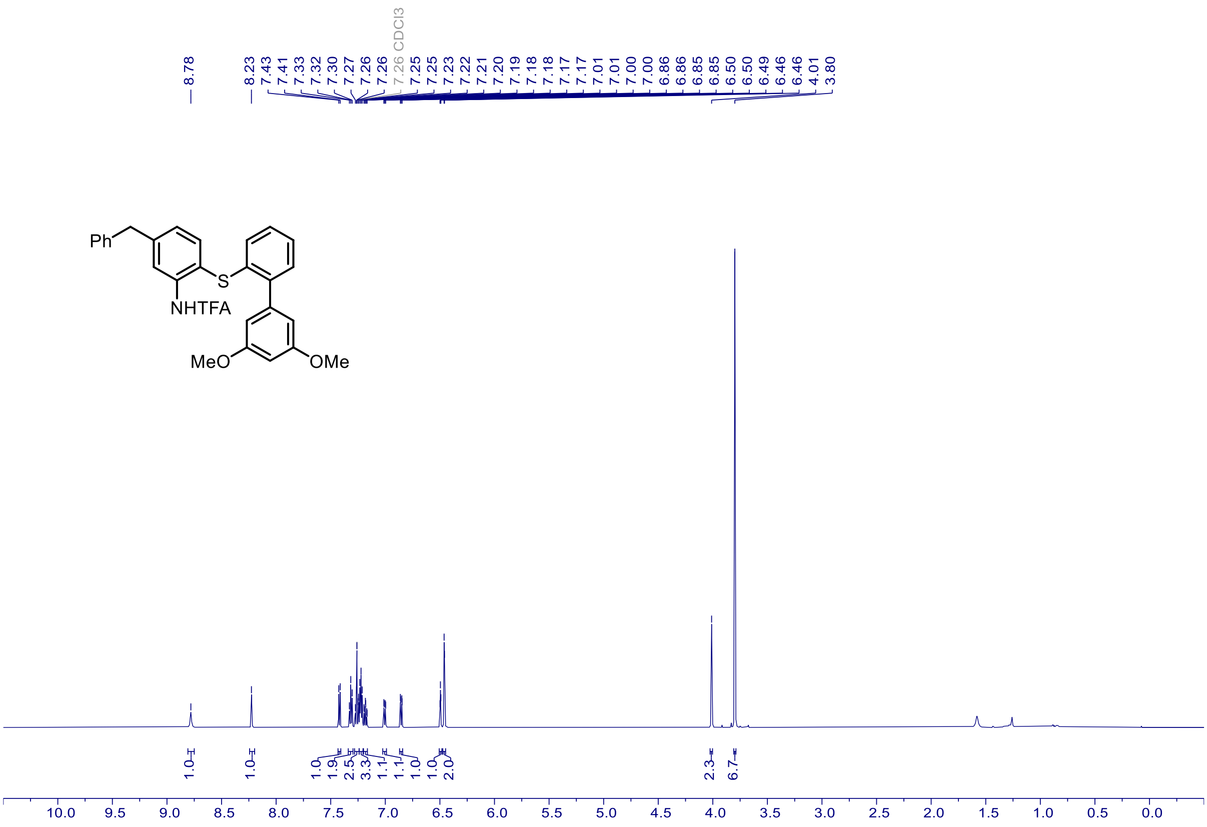

3eh – ^{13}C NMR (151 MHz, $CDCl_3$)

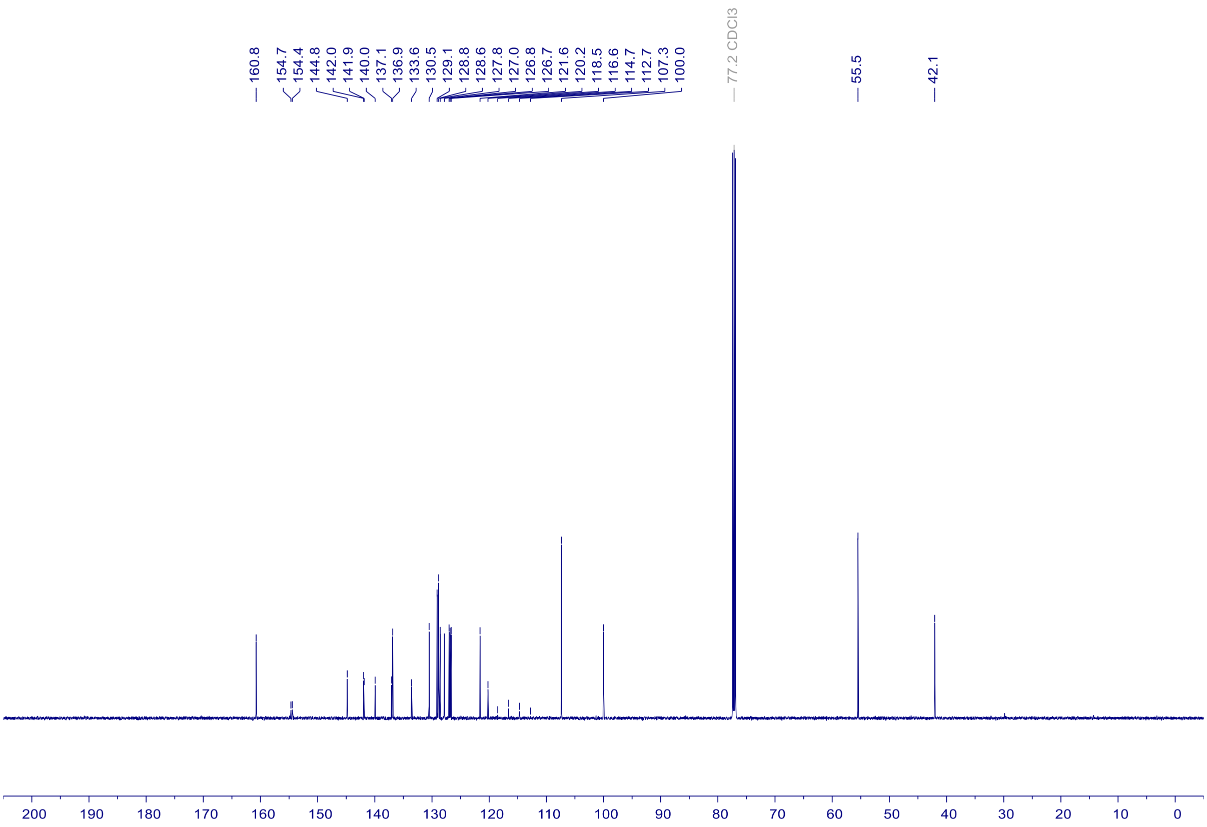

3eh – ^{19}F NMR (565 MHz, $CDCl_3$)

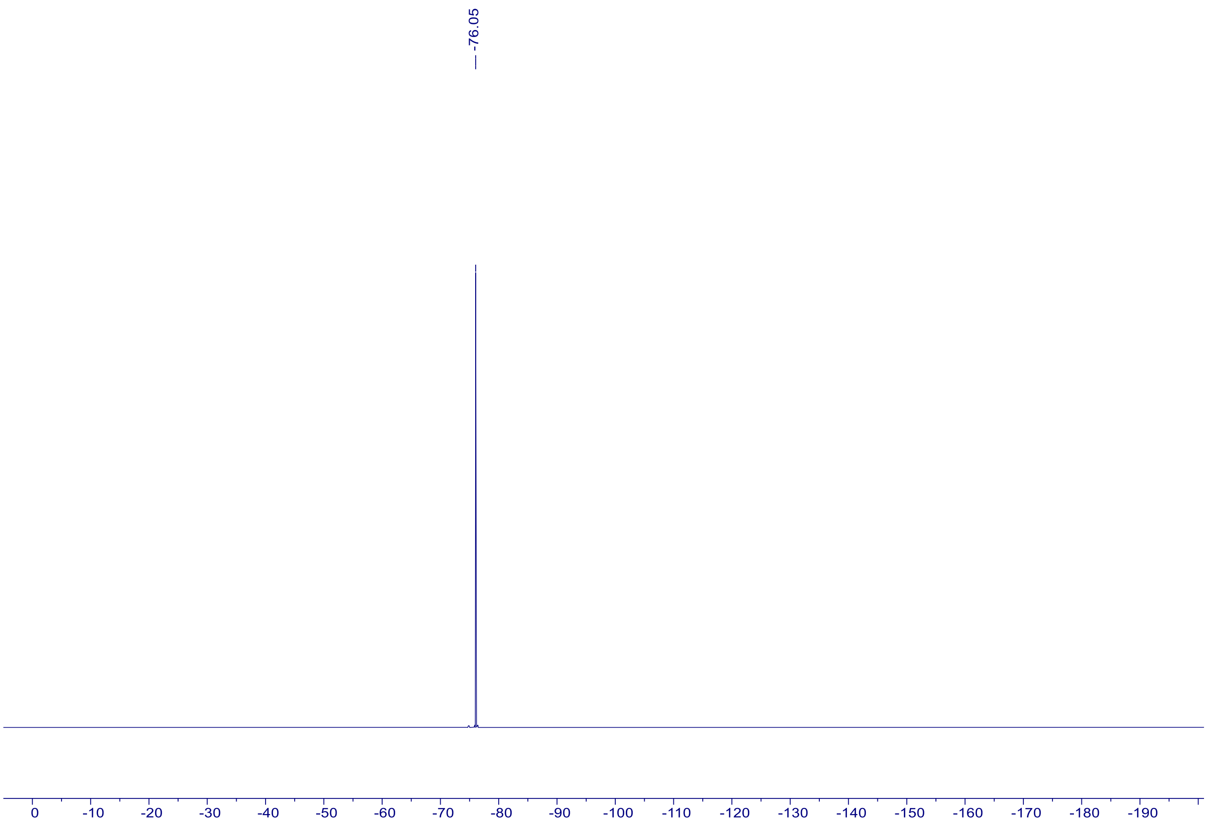

3fh – ^{1}H NMR (600 MHz, $CDCl_3$)

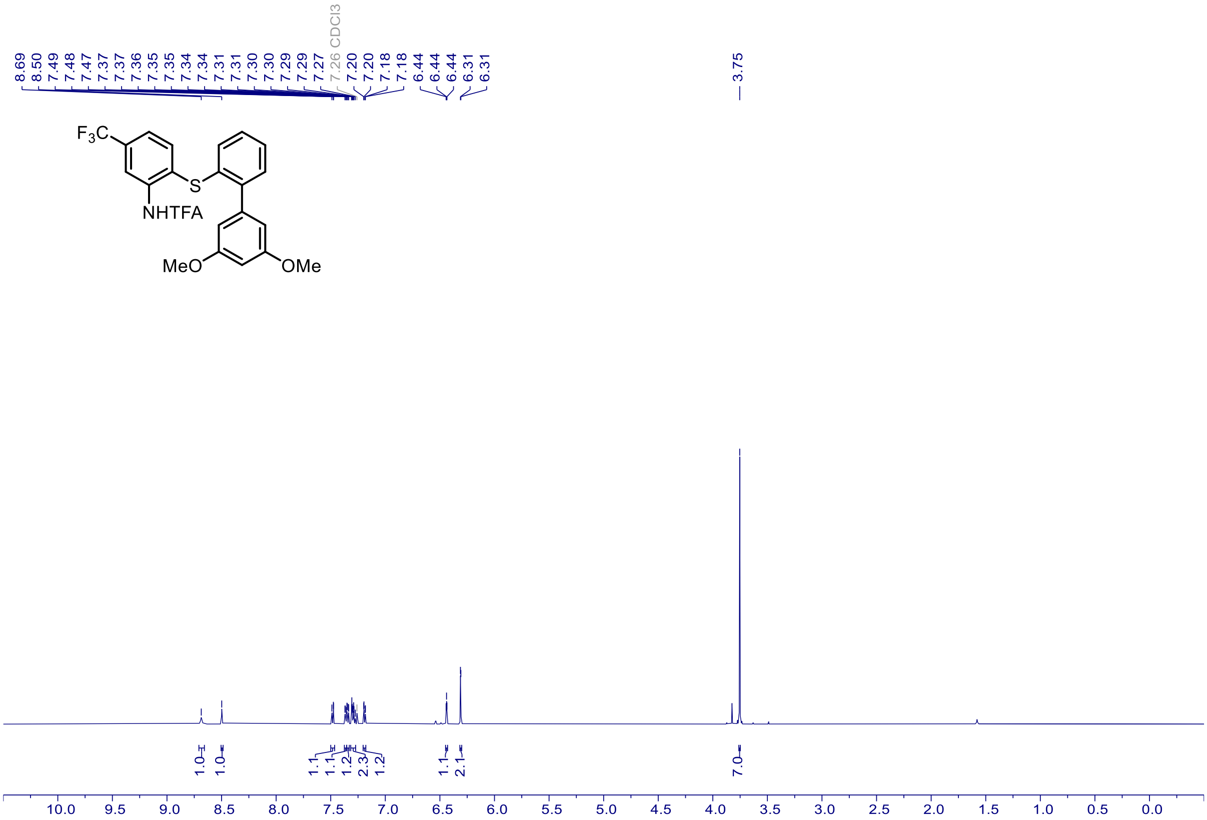

3fh – ^{13}C NMR (151 MHz, $CDCl_3$)

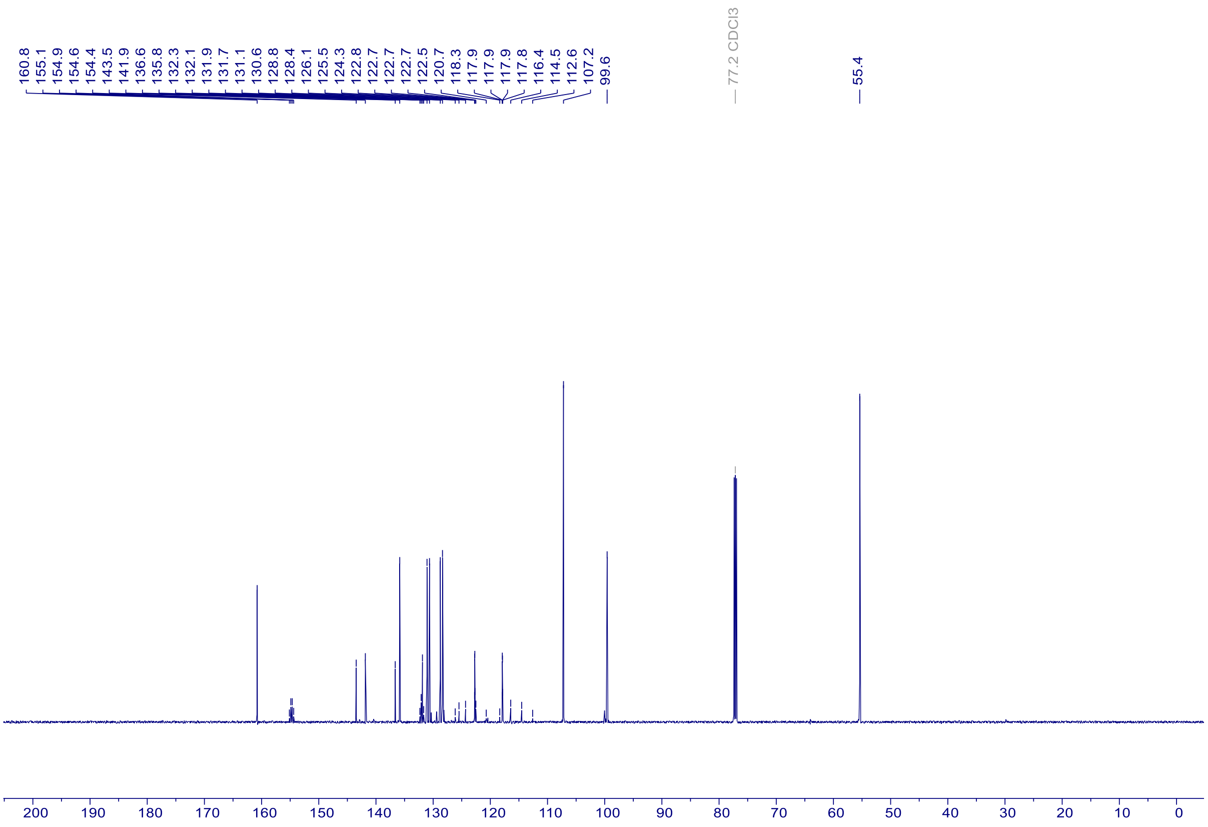

3fh – ^{19}F NMR (565 MHz, $CDCl_3$)

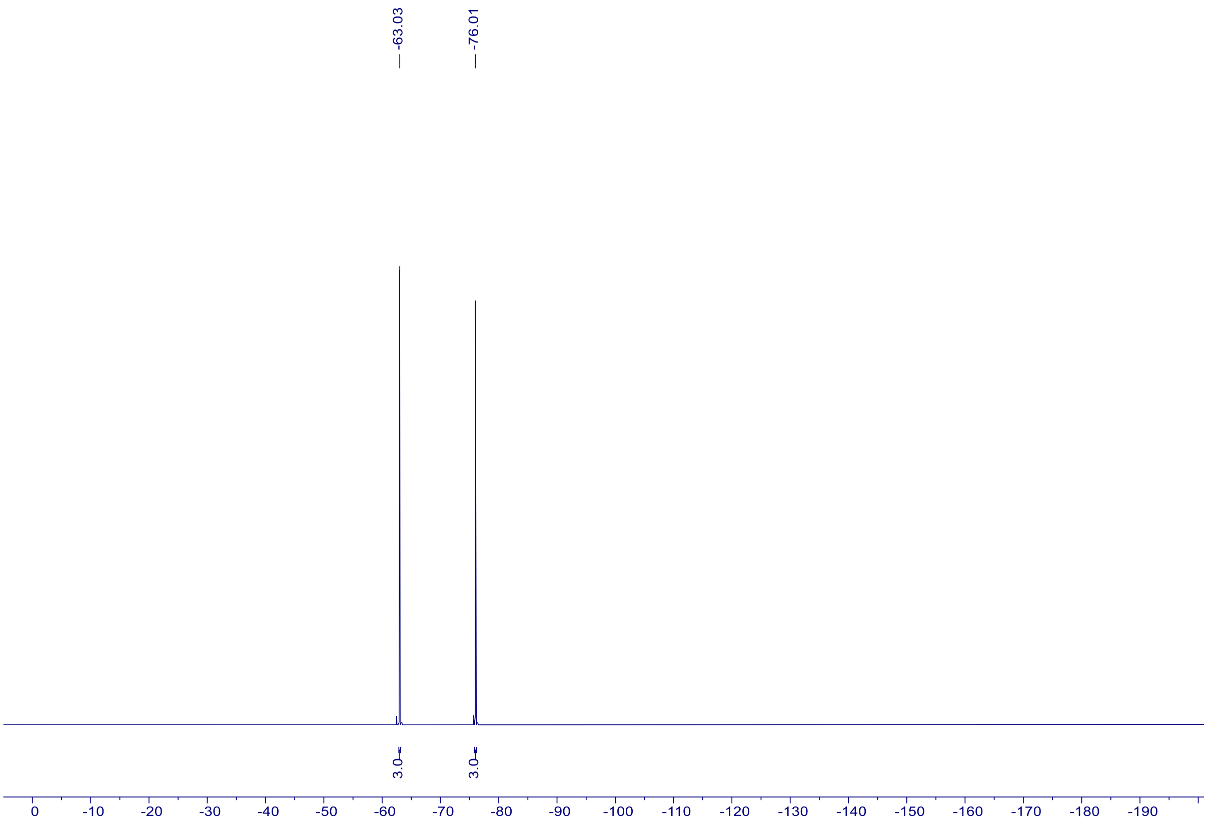

3gh – ^{1}H NMR (600 MHz, $CDCl_3$)

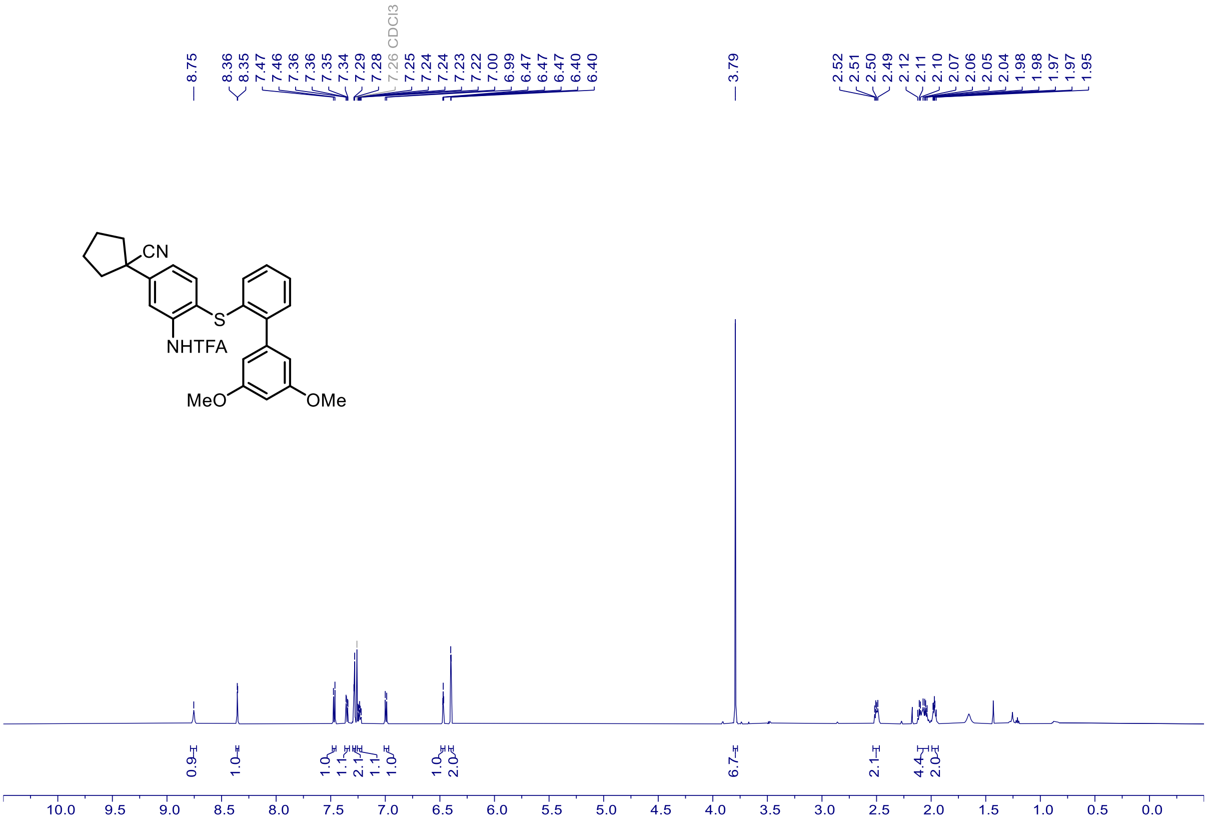

3gh – ^{13}C NMR (151 MHz, $CDCl_3$)

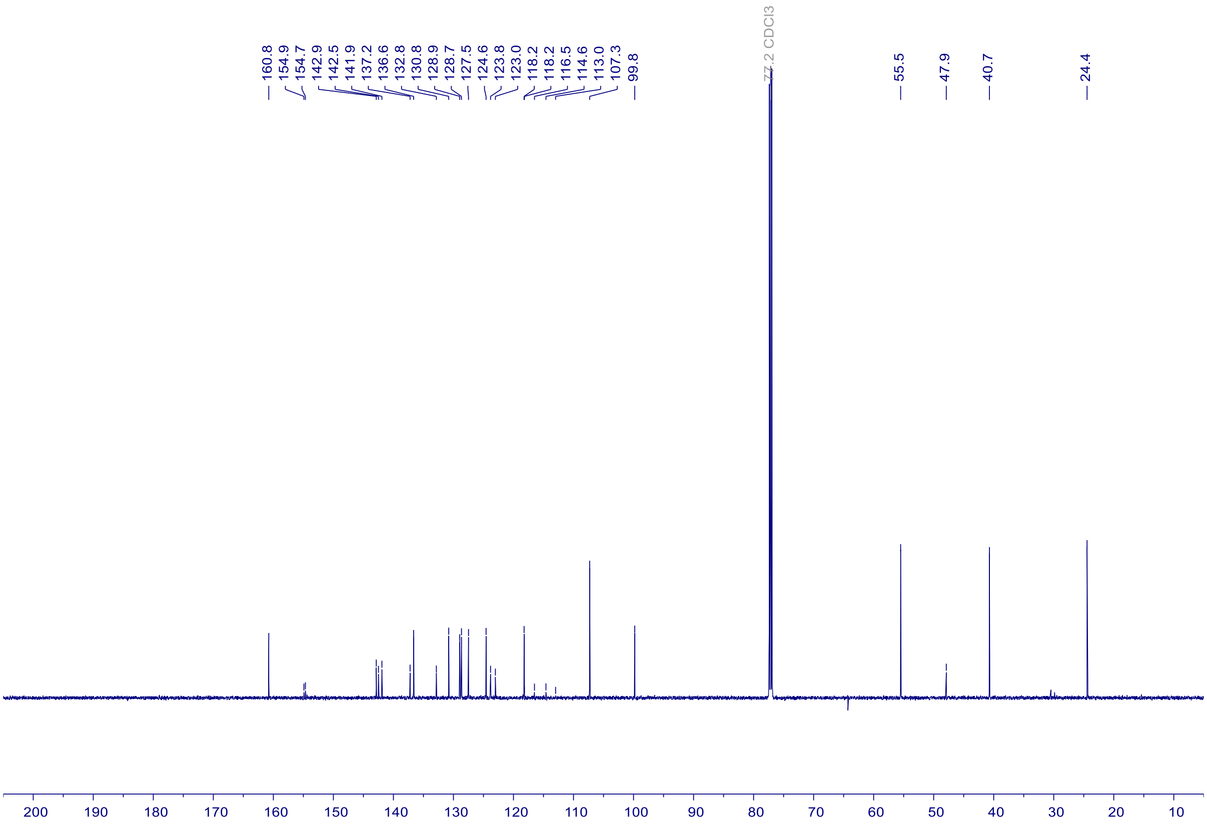

3gh – ^{19}F NMR (565 MHz, CDCl$_3$)

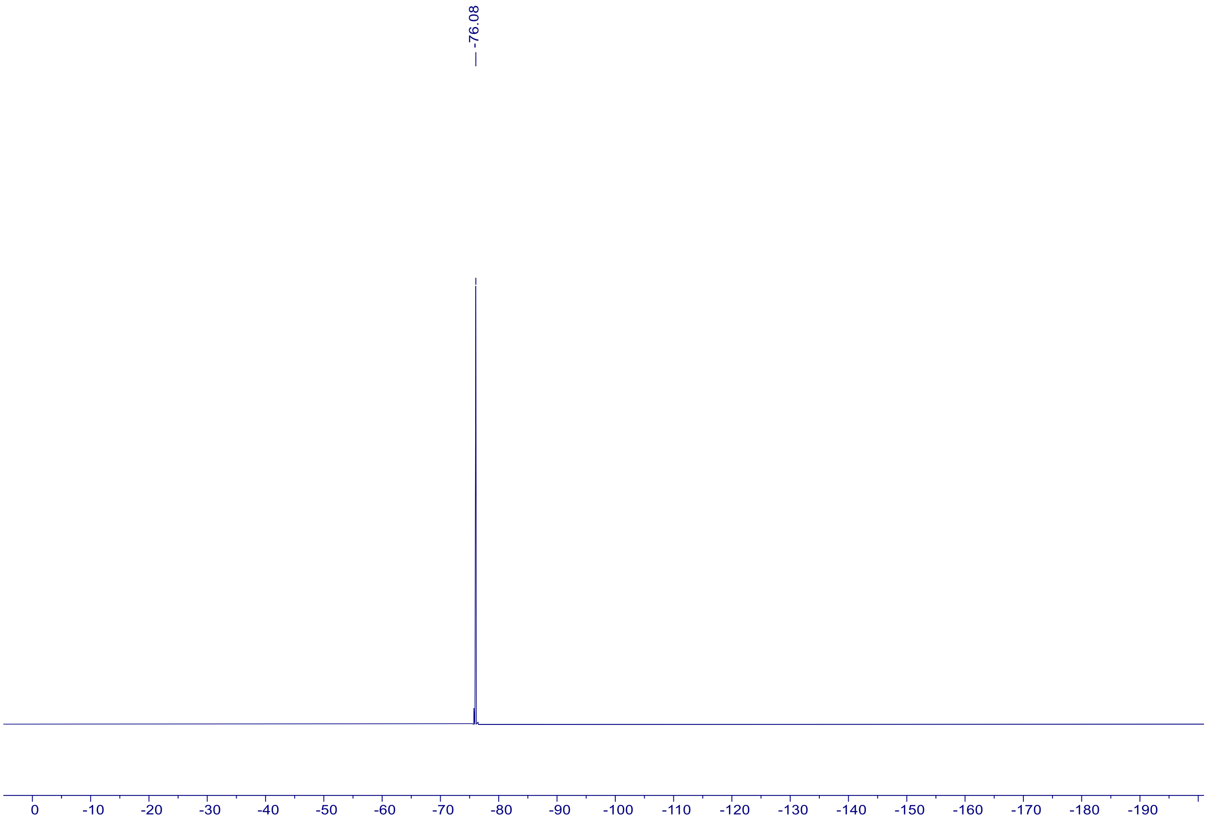

3hh – ^{1}H NMR (600 MHz, CDCl$_3$)

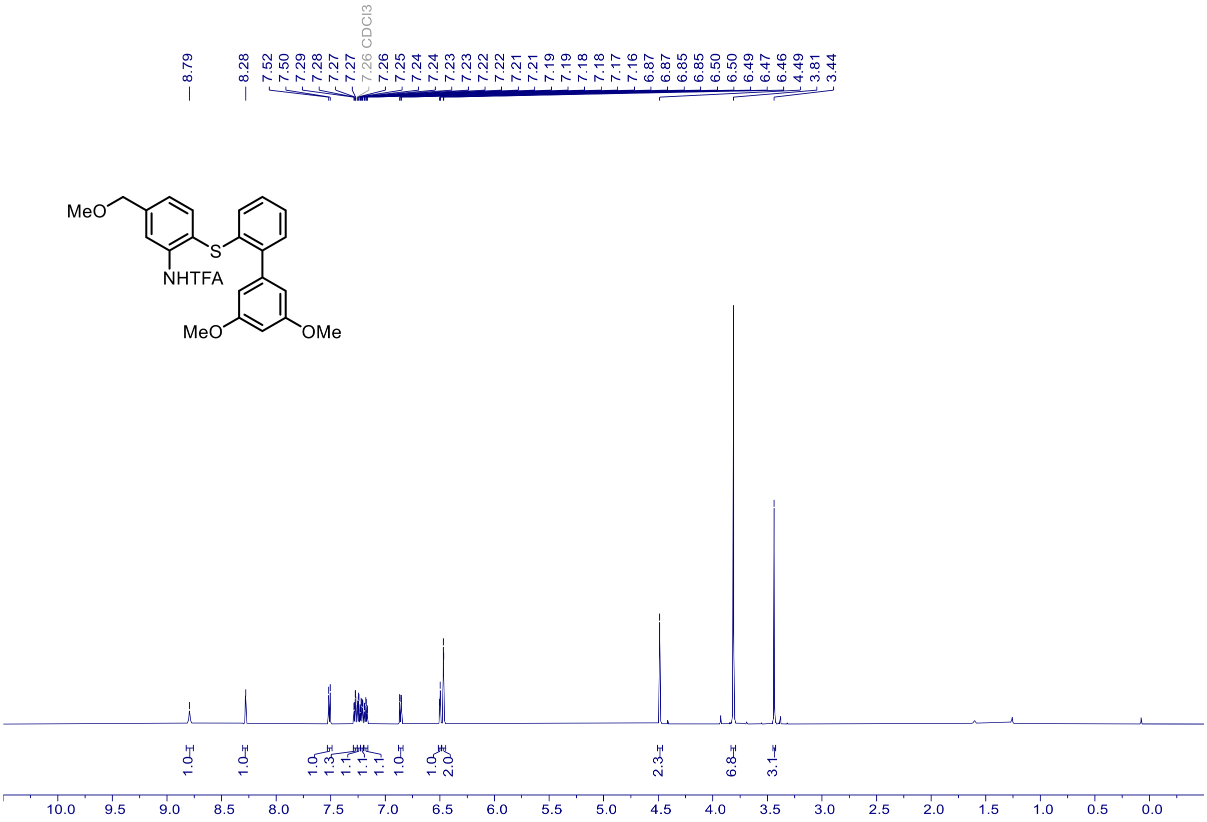

3hh – ^{13}C NMR (151 MHz, $CDCl_3$)

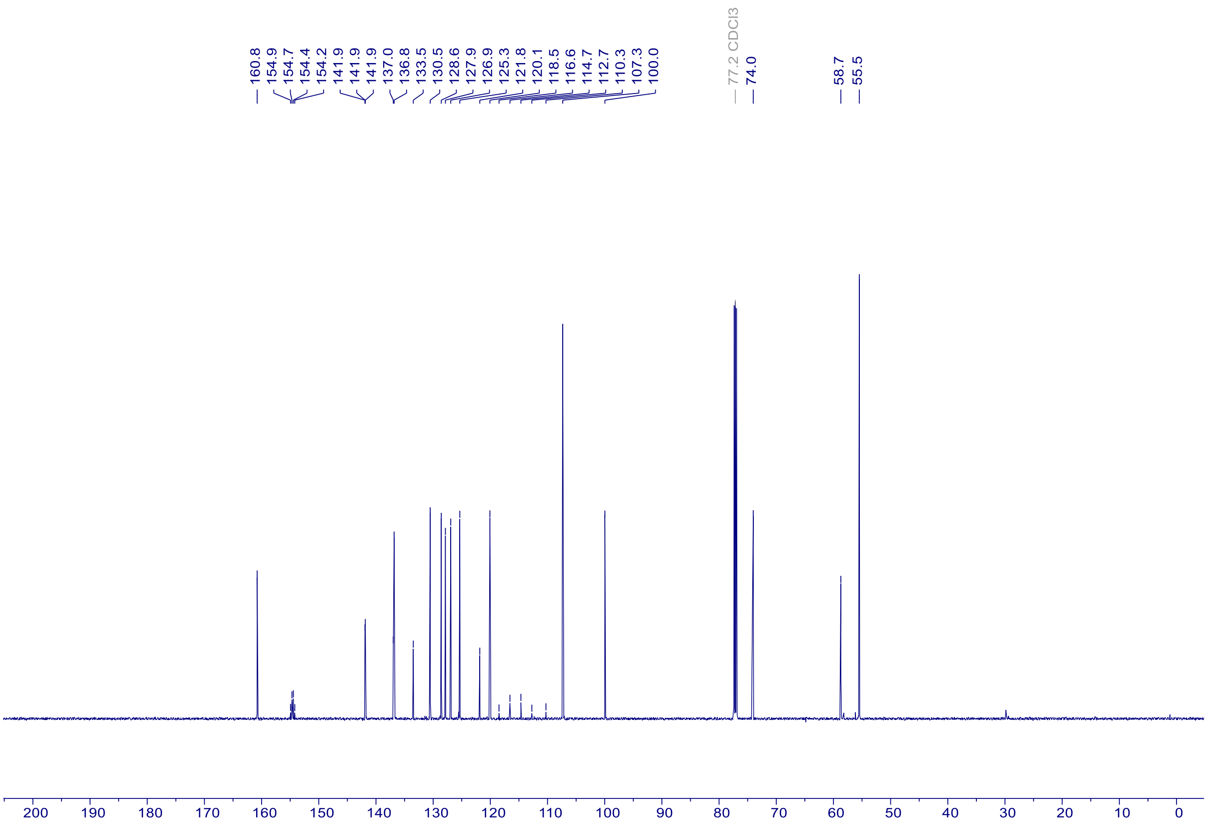

3hh – ^{19}F NMR (565 MHz, $CDCl_3$)

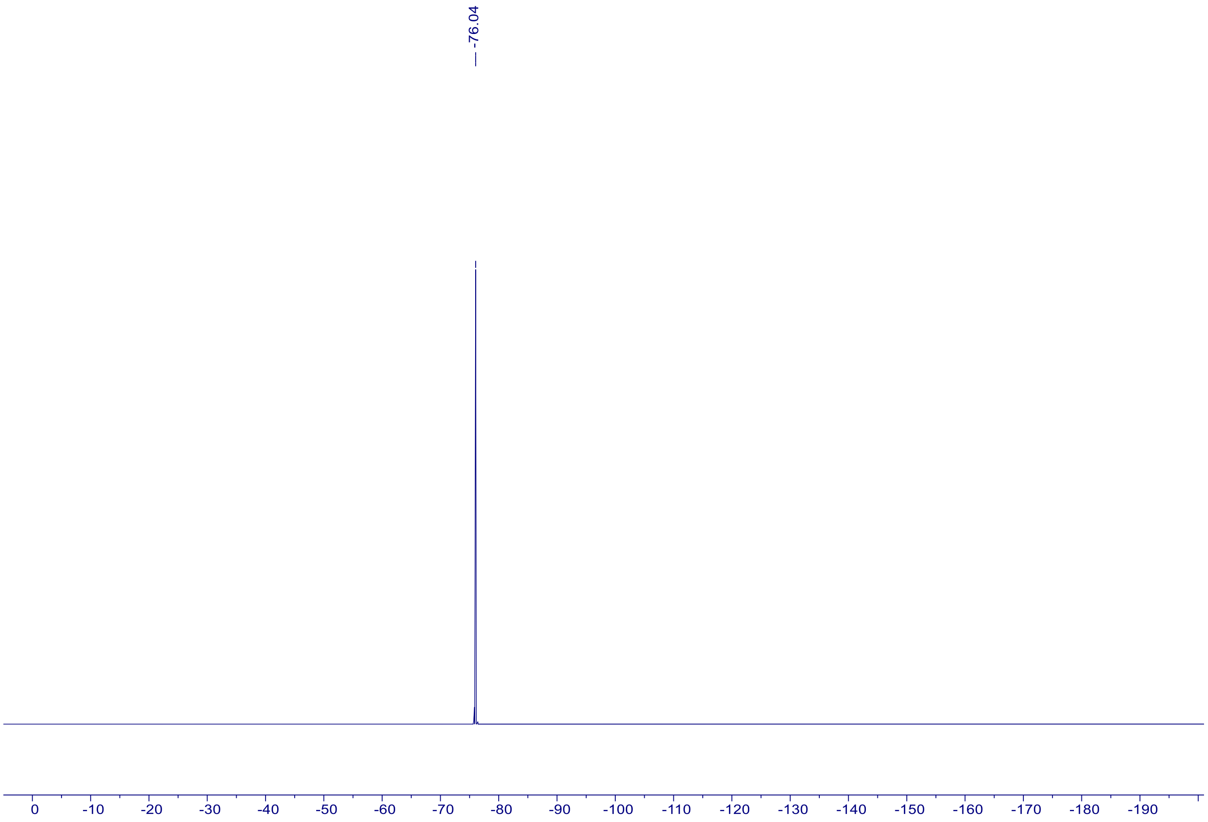

3ih – ^{1}H NMR (600 MHz, CDCl$_3$)

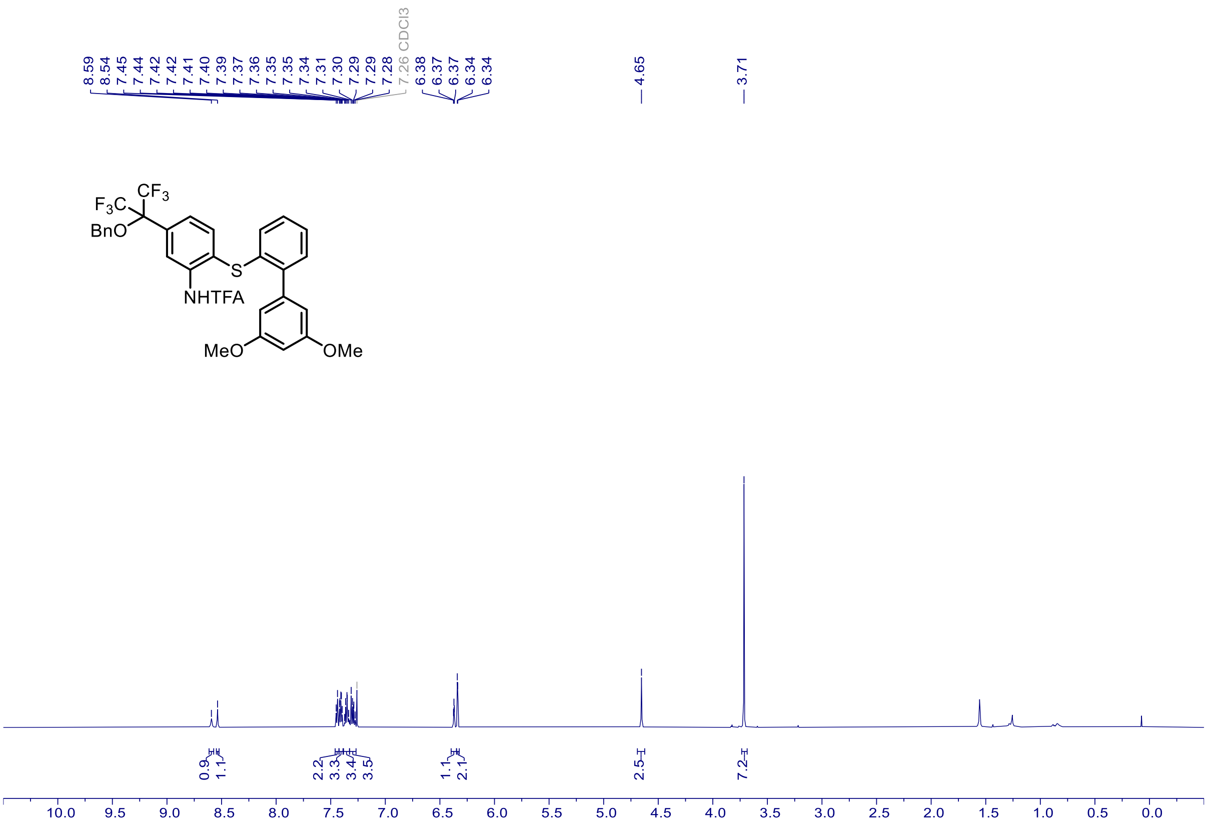

3ih – ^{13}C NMR (151 MHz, CDCl$_3$)

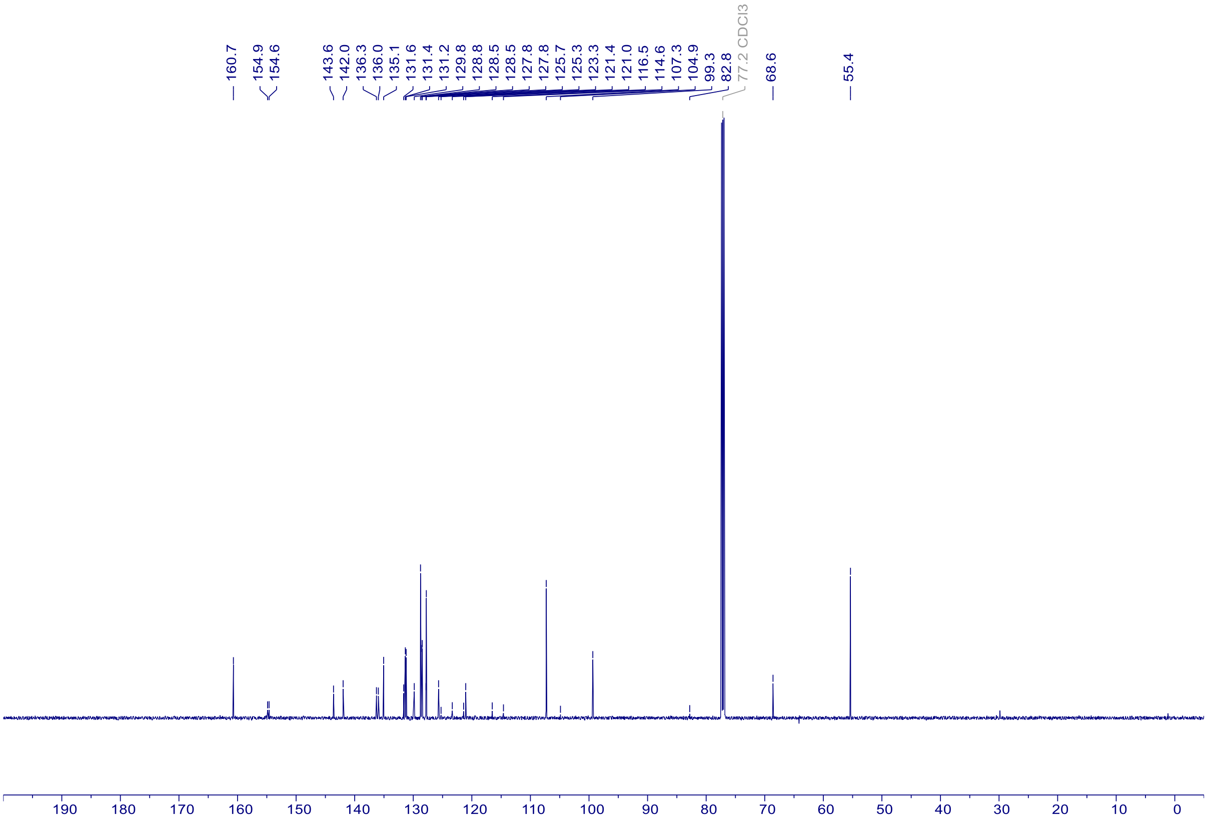

3ih – ^{19}F NMR (565 MHz, $CDCl_3$)

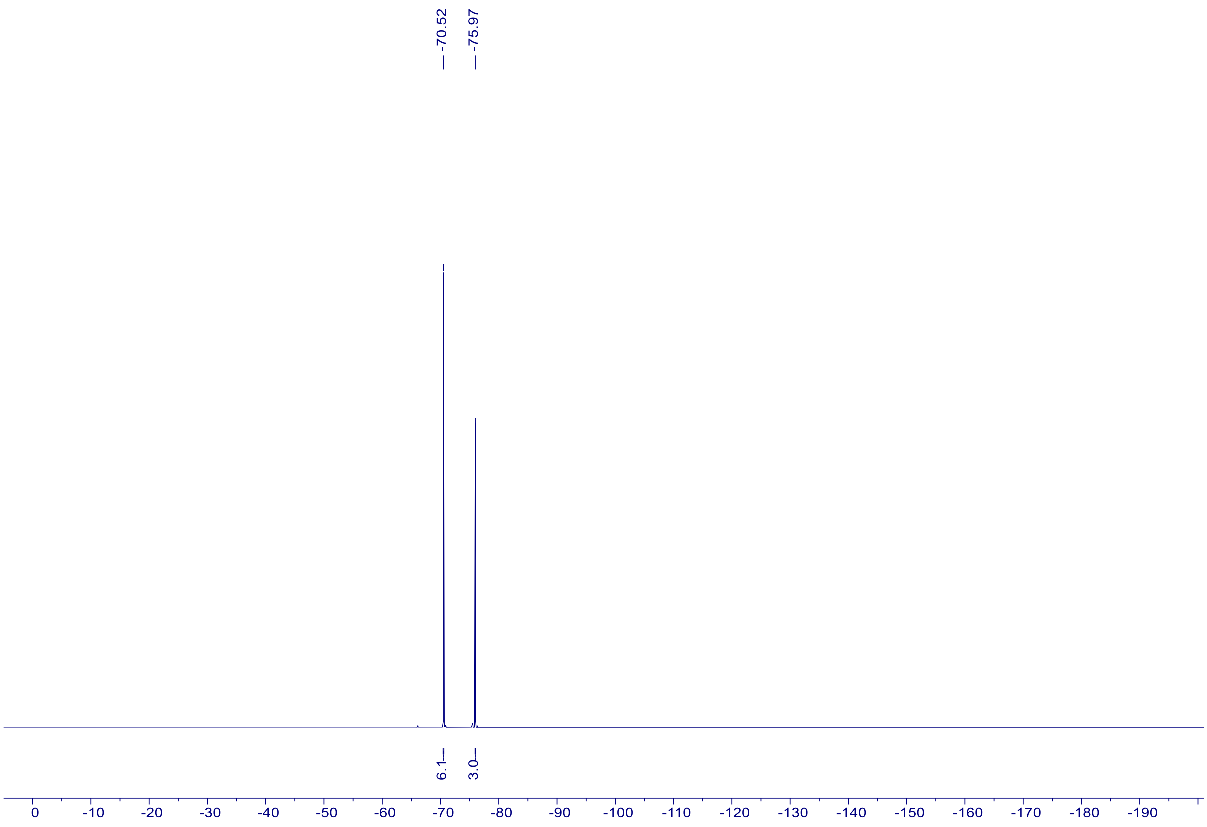

3jh – ^{1}H NMR (600 MHz, $CDCl_3$)

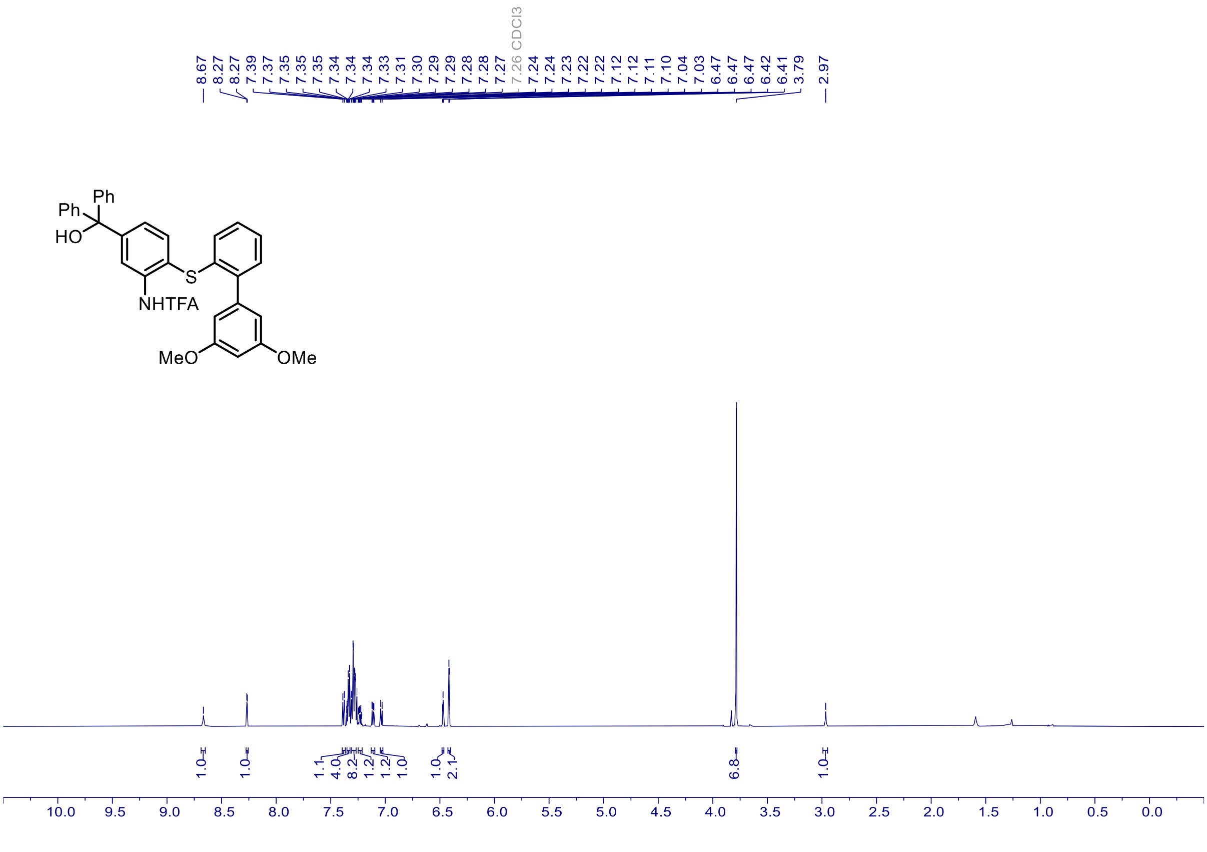

3jh – ^{13}C NMR (151 MHz, $CDCl_3$)

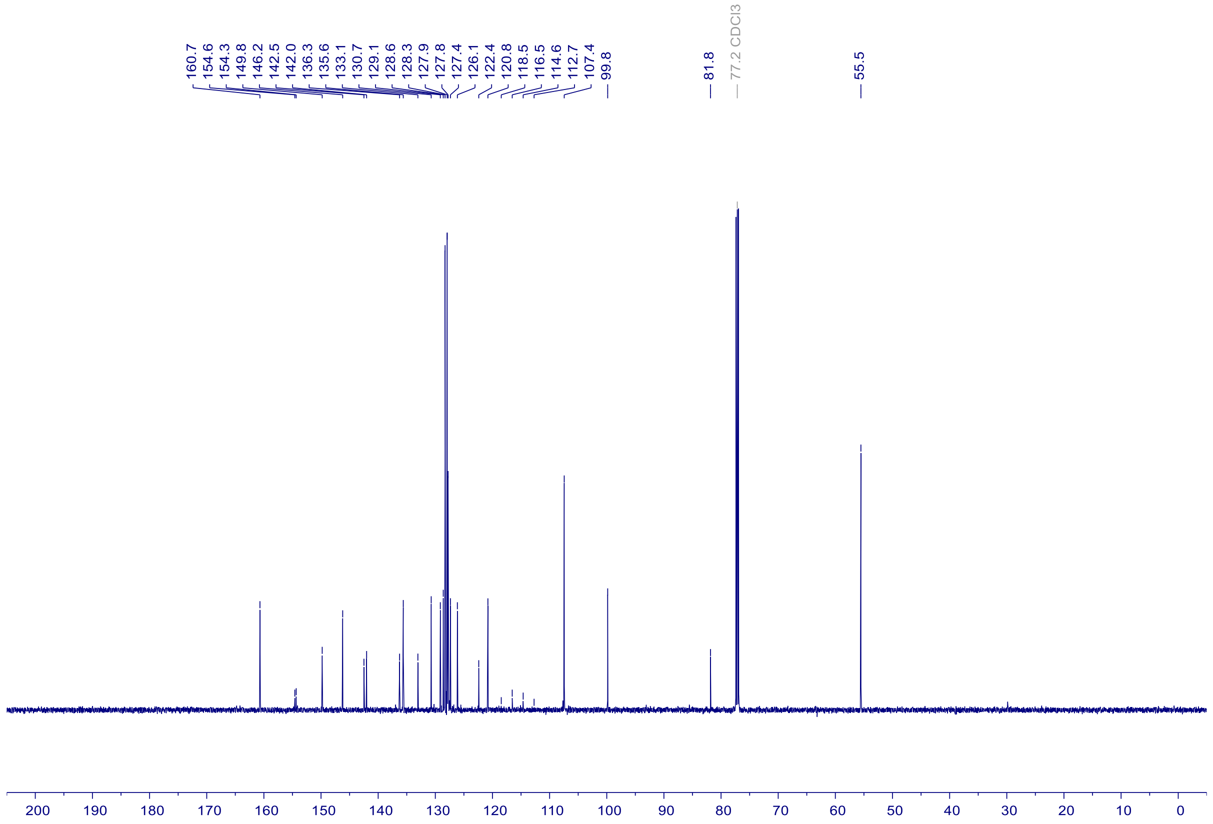

3jh – ^{19}F NMR (565 MHz, $CDCl_3$)

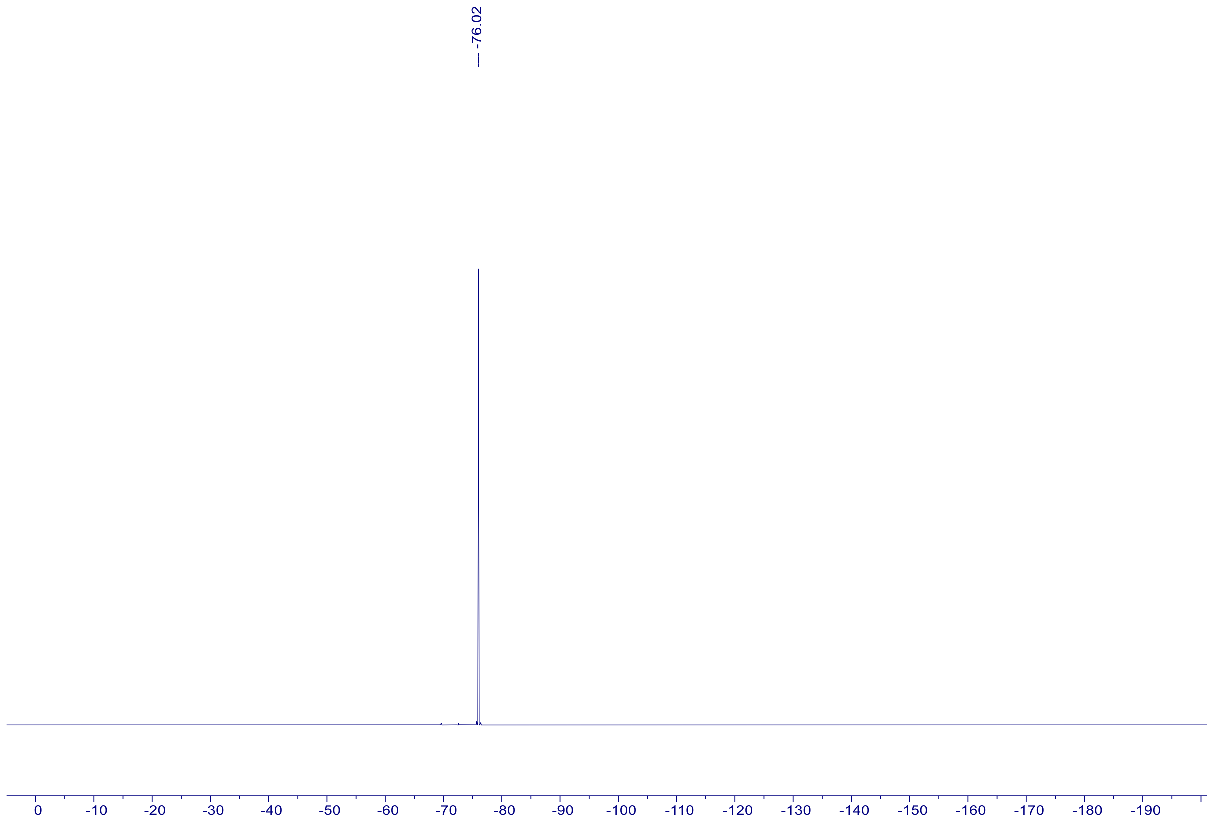

4kh – ^{1}H NMR (600 MHz, $CDCl_3$)

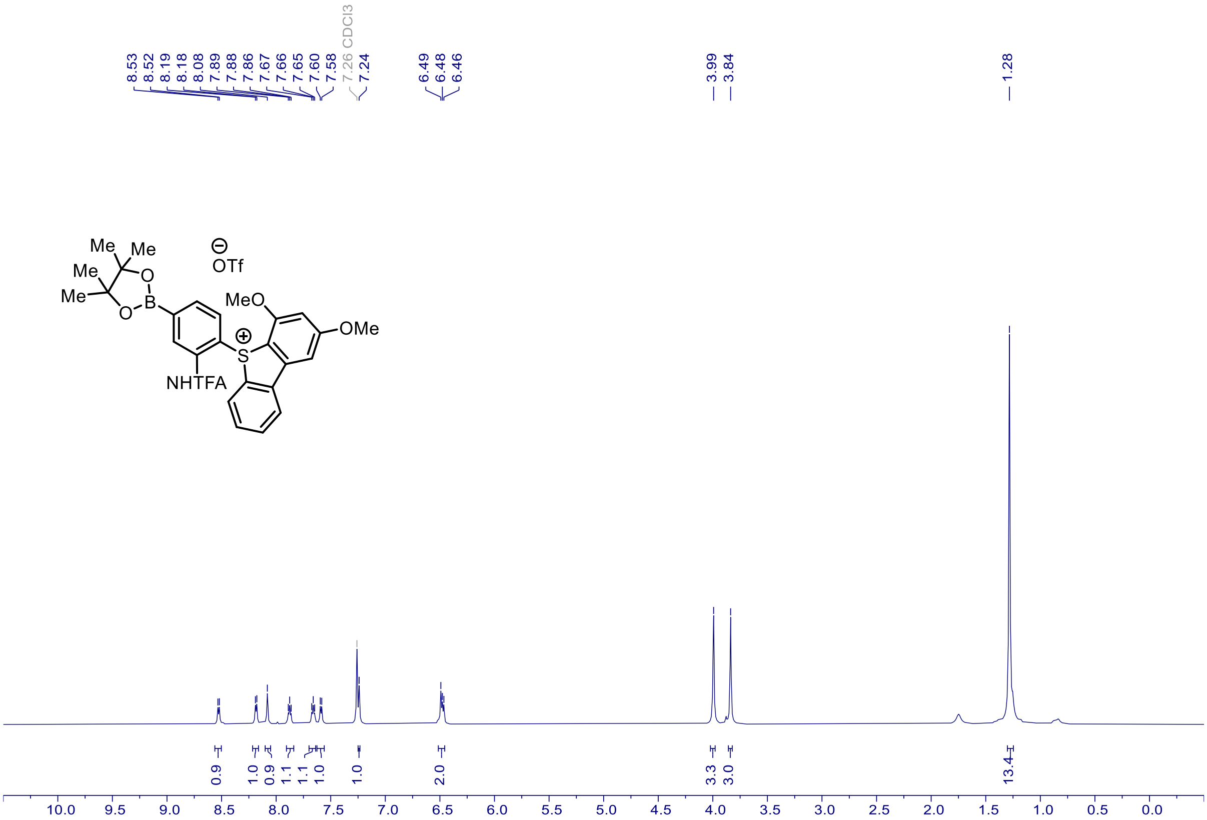

4kh – ^{13}C NMR (151 MHz, $CDCl_3$)

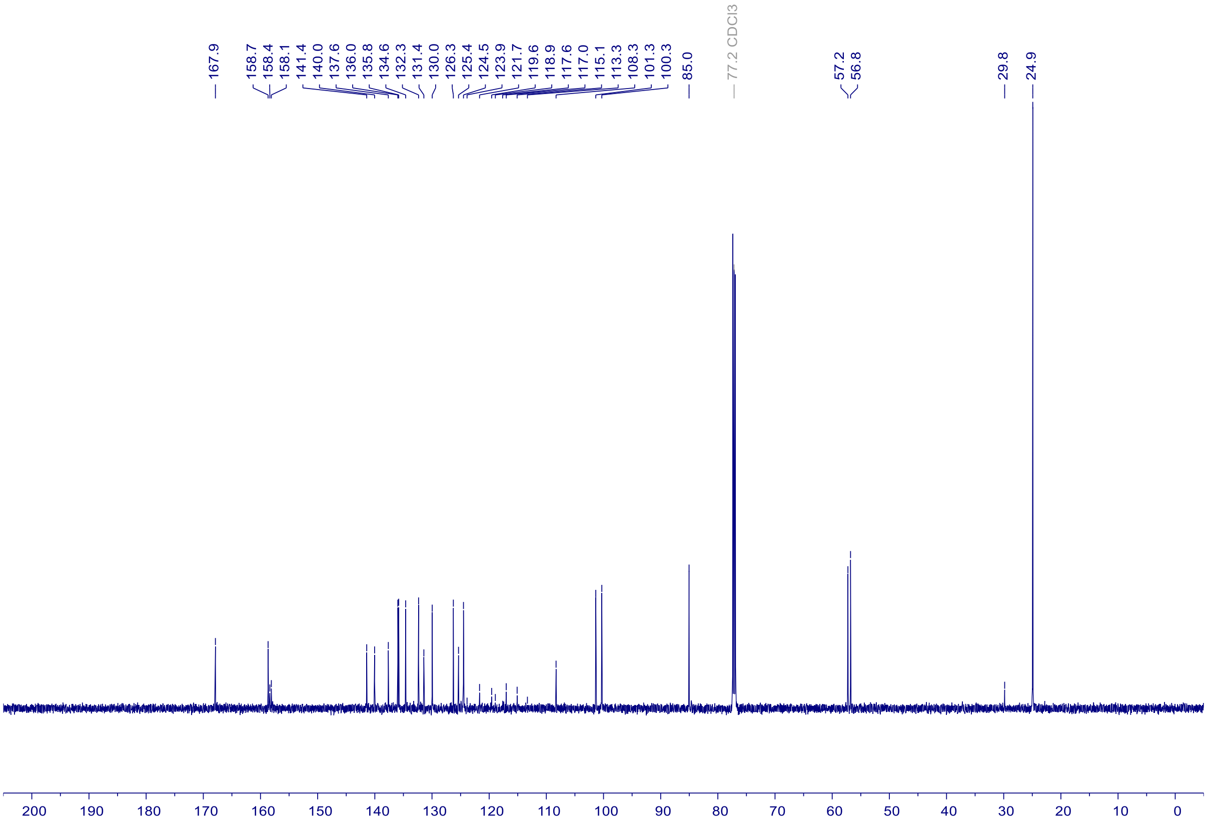

4kh – ^{19}F NMR (565 MHz, $CDCl_3$)

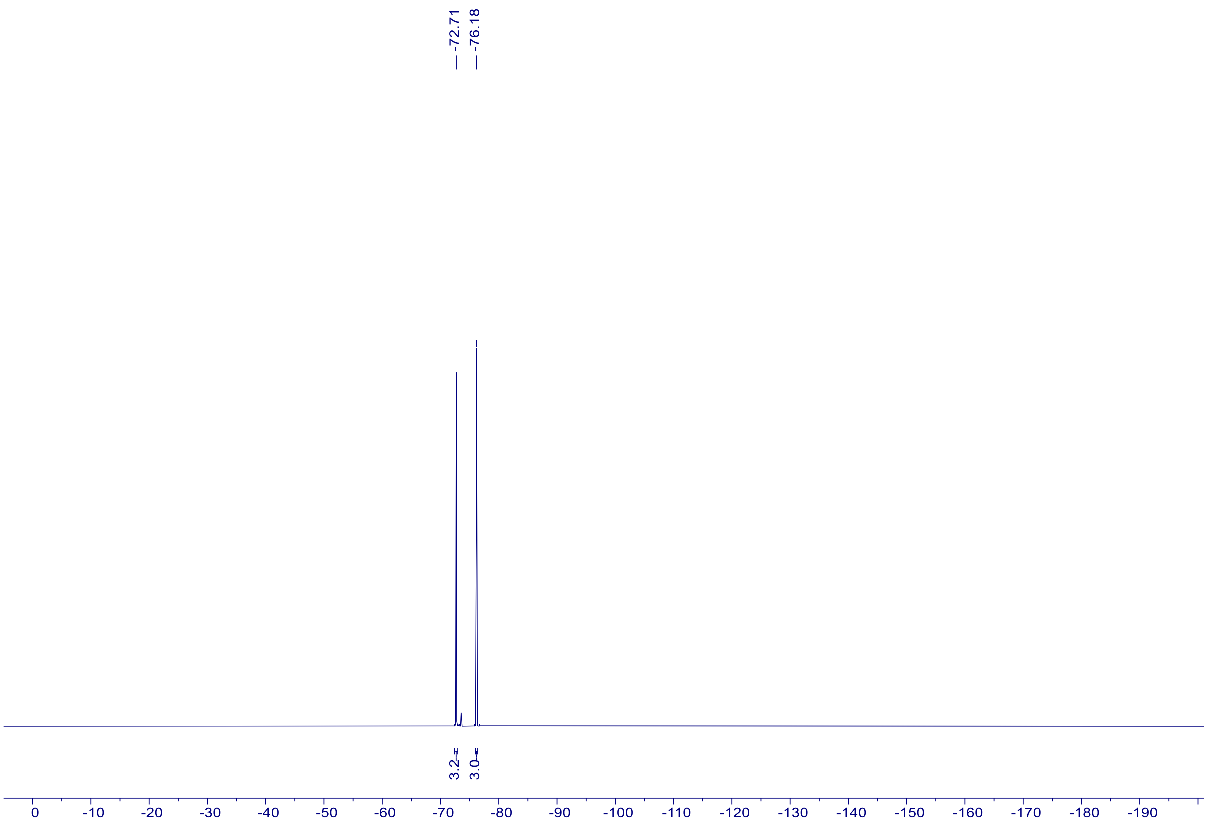

3lh – ^{1}H NMR (600 MHz, $CDCl_3$)

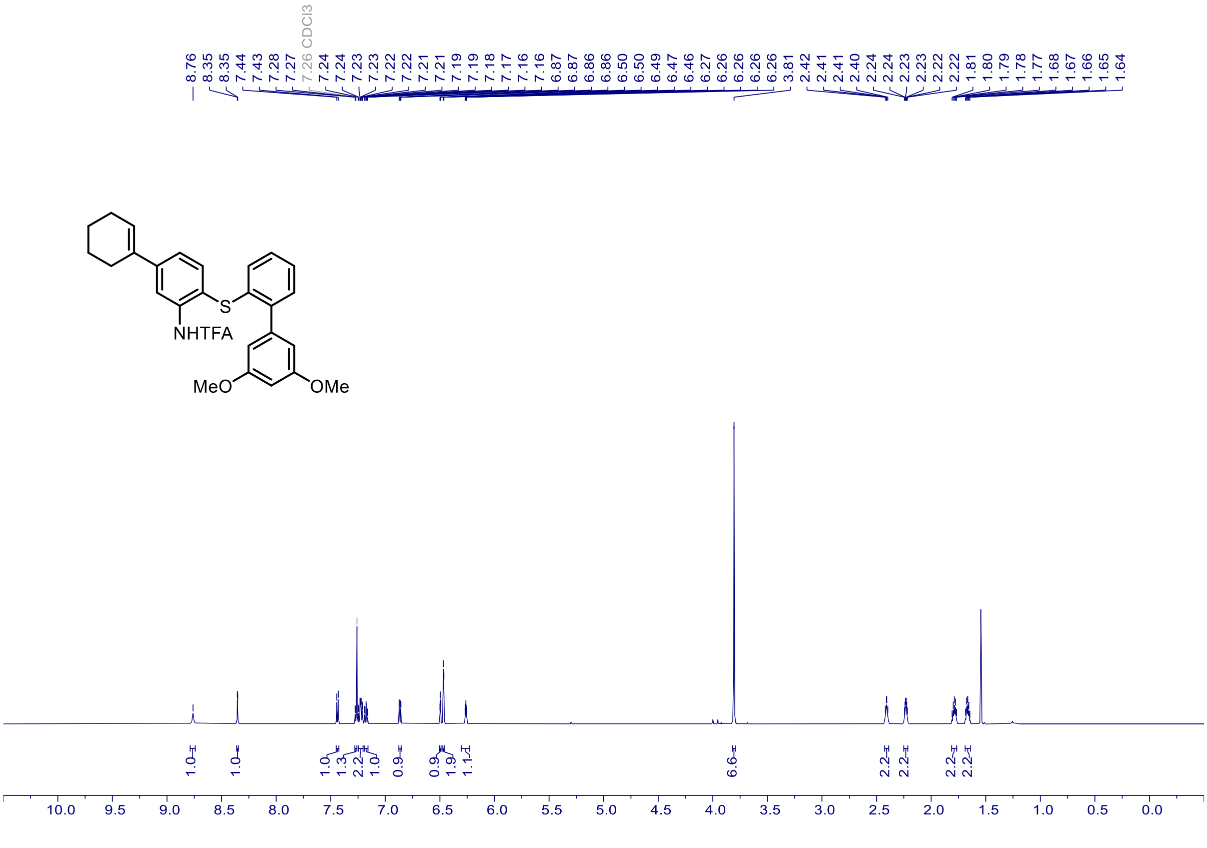

3lh – ^{13}C NMR (151 MHz, $CDCl_3$)

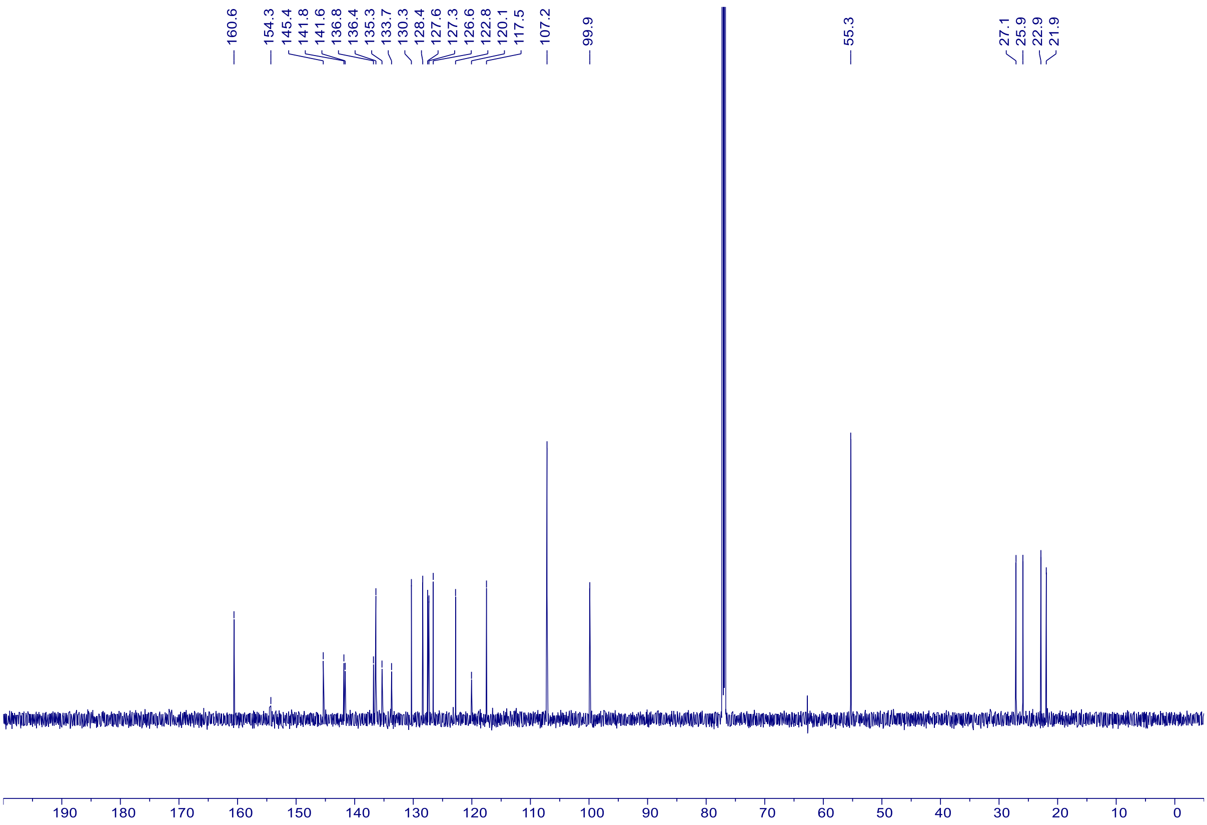

3lh – ^{19}F NMR (565 MHz, $CDCl_3$)

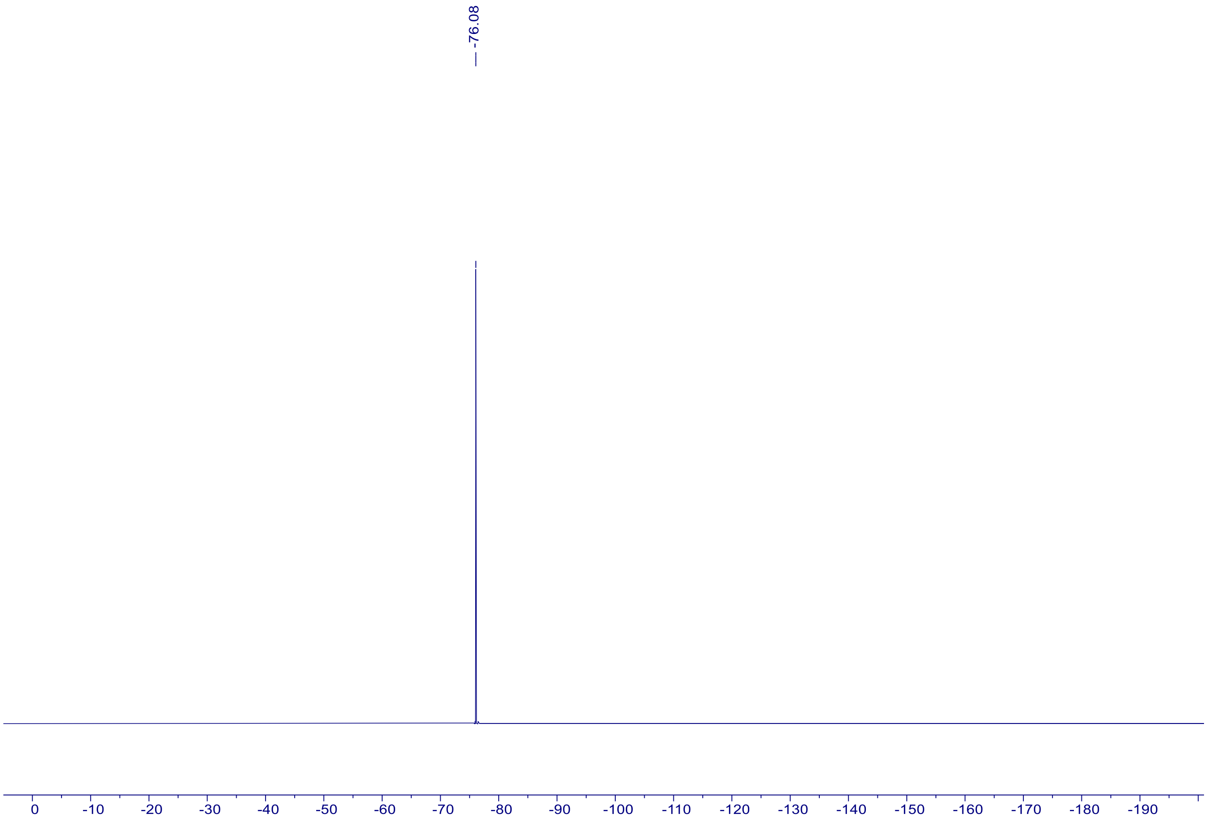

3mh – ^{1}H NMR (600 MHz, $CDCl_3$)

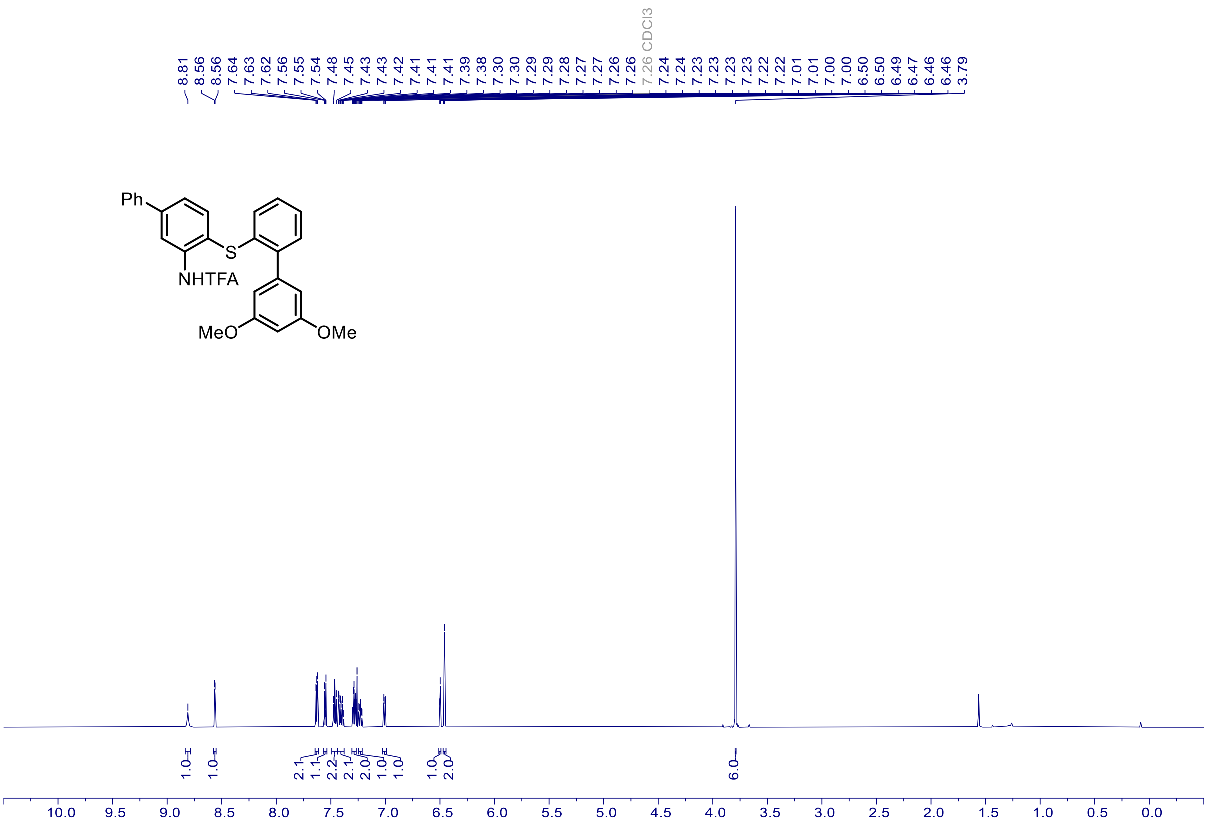

3mh – ^{13}C NMR (151 MHz, $CDCl_3$)

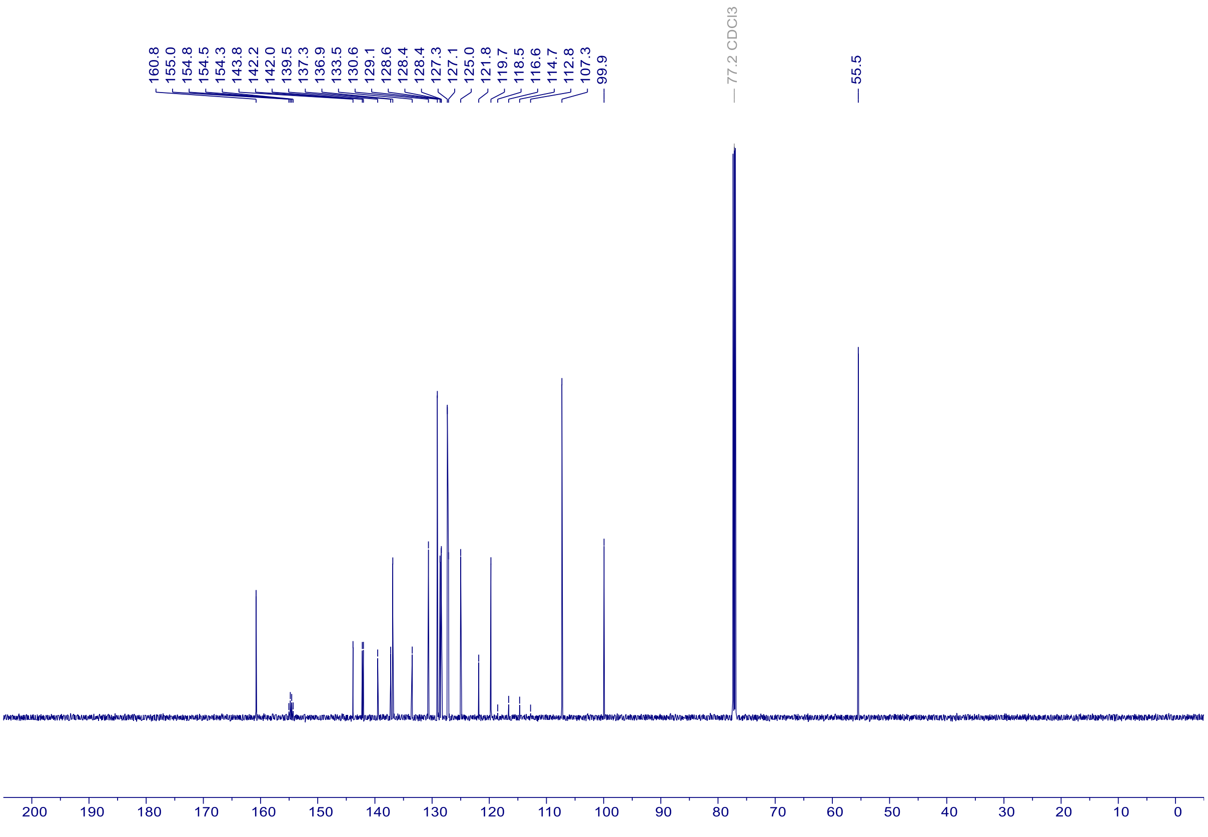

3mh – ^{19}F NMR (565 MHz, CDCl$_3$)

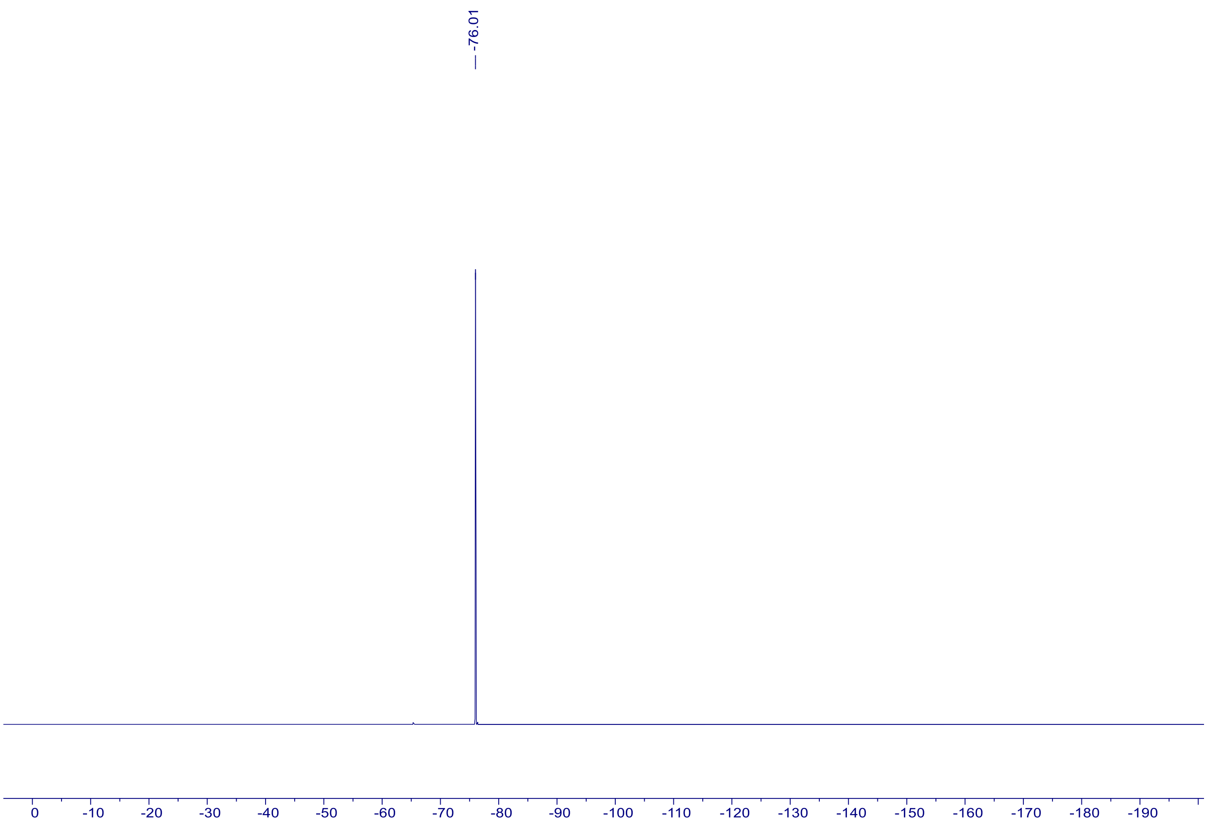

3nh – ^{1}H NMR (600 MHz, CDCl$_3$)

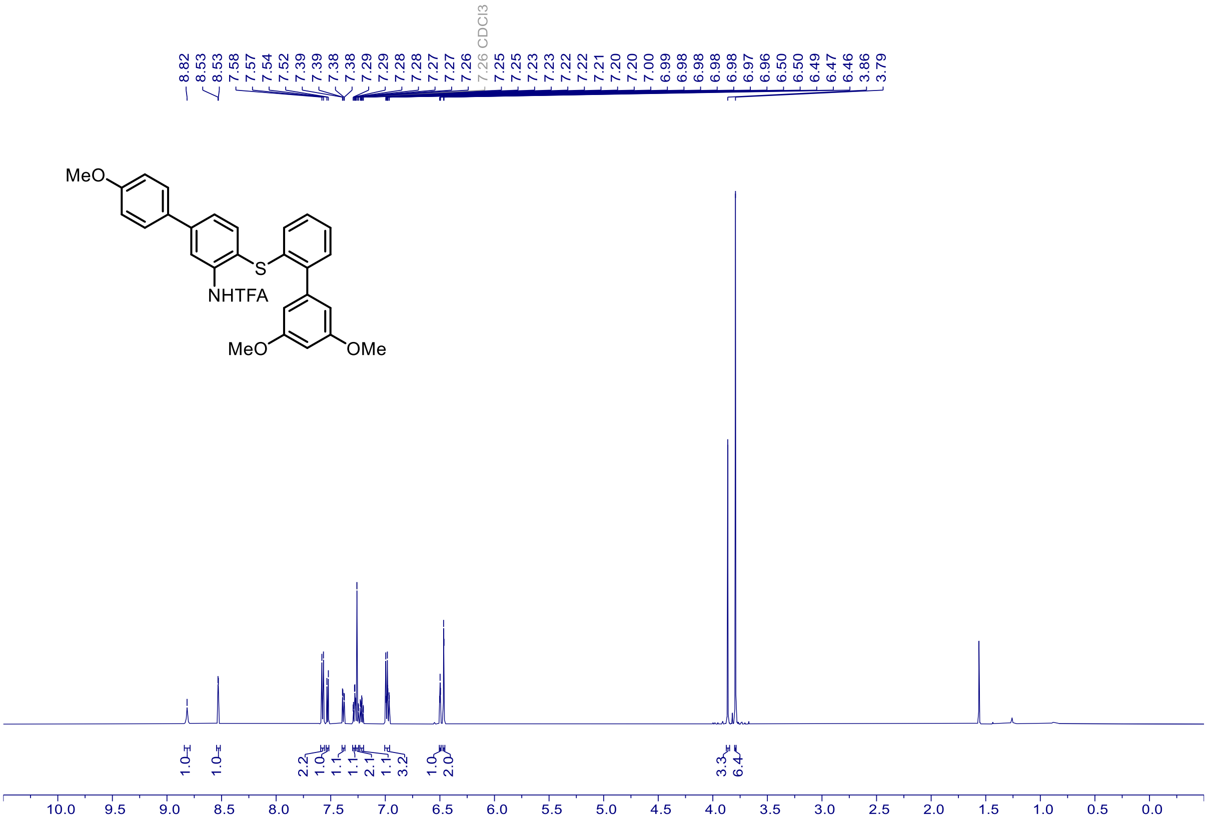

3nh – ^{13}C NMR (151 MHz, CDCl$_3$)

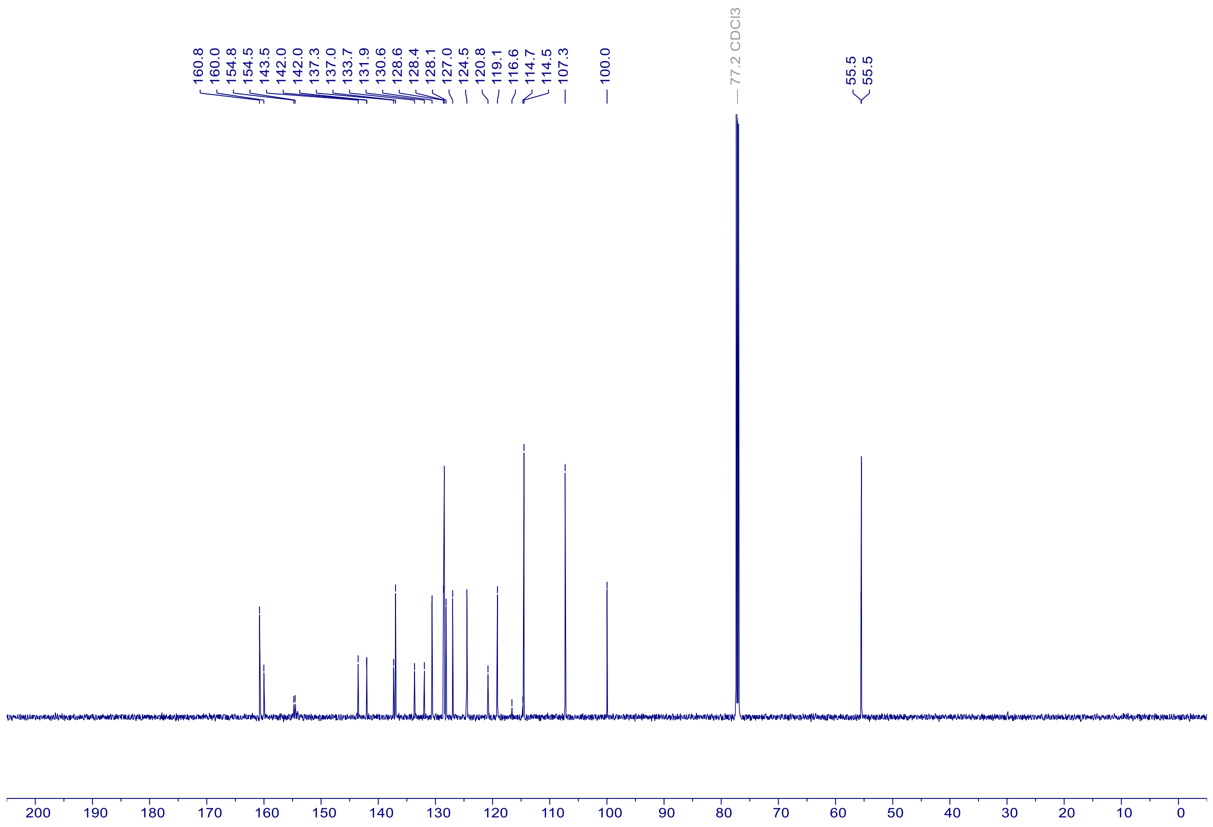

3nh – ^{19}F NMR (565 MHz, CDCl$_3$)

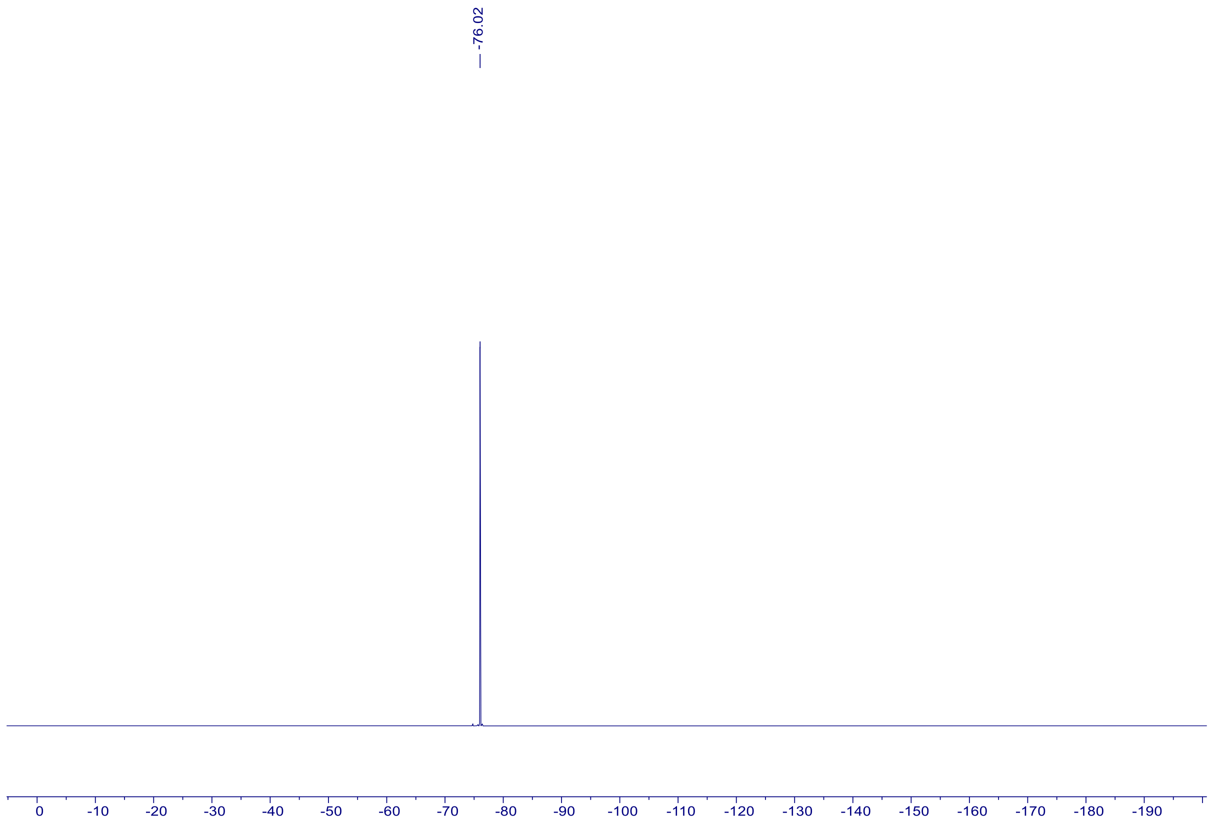

3oh – ^{1}H NMR (600 MHz, $CDCl_3$)

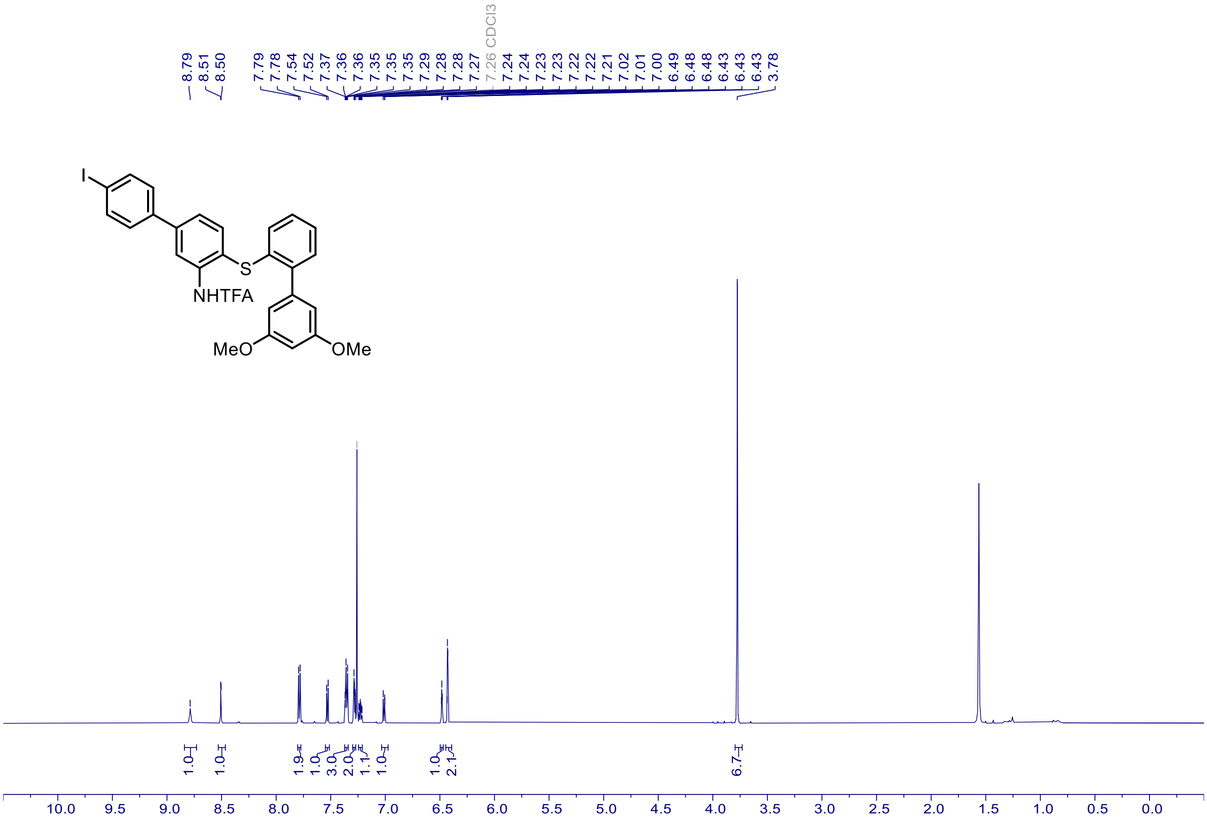

3oh – ^{13}C NMR (151 MHz, $CDCl_3$)

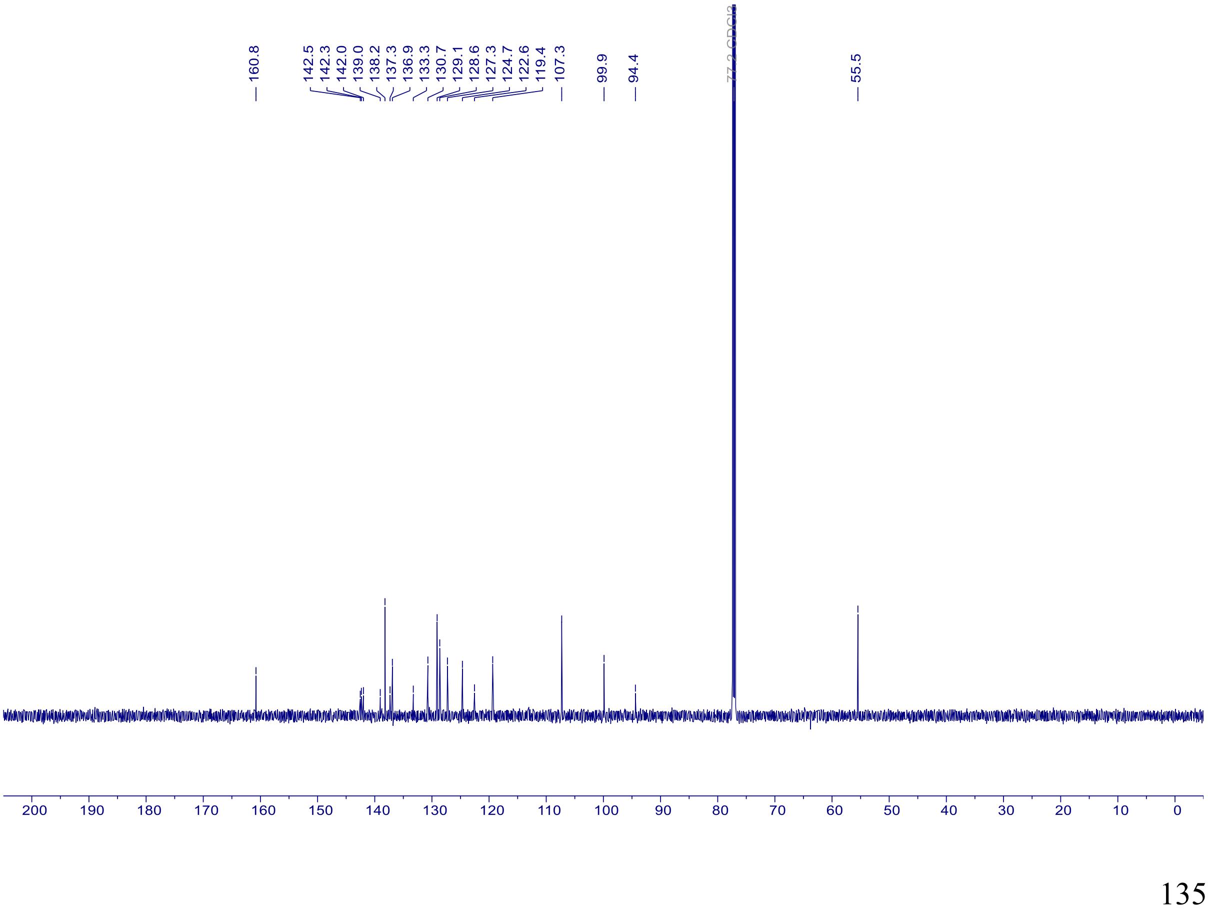

3oh – ^{19}F NMR (565 MHz, $CDCl_3$)

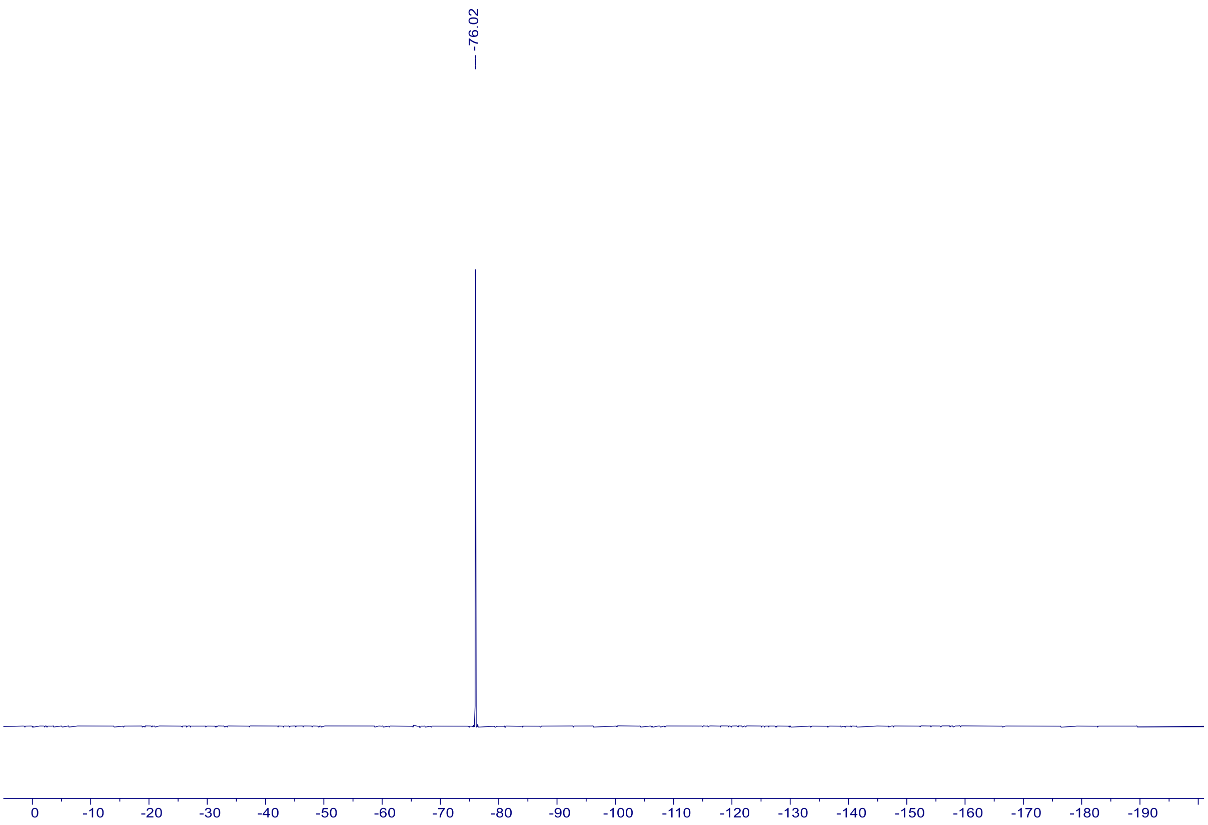

3ph – ^{1}H NMR (600 MHz, $CDCl_3$)

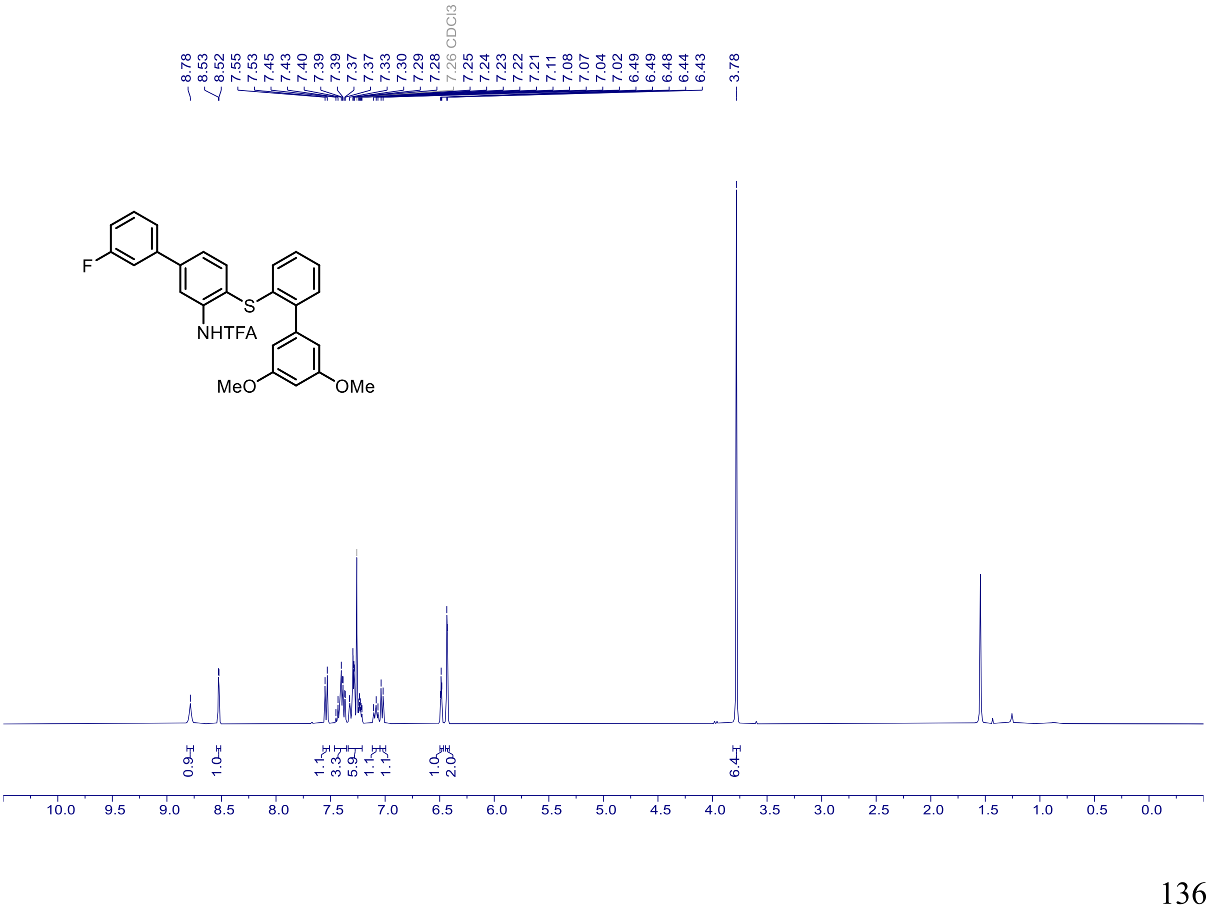

3ph – ^{13}C NMR (151 MHz, $CDCl_3$)

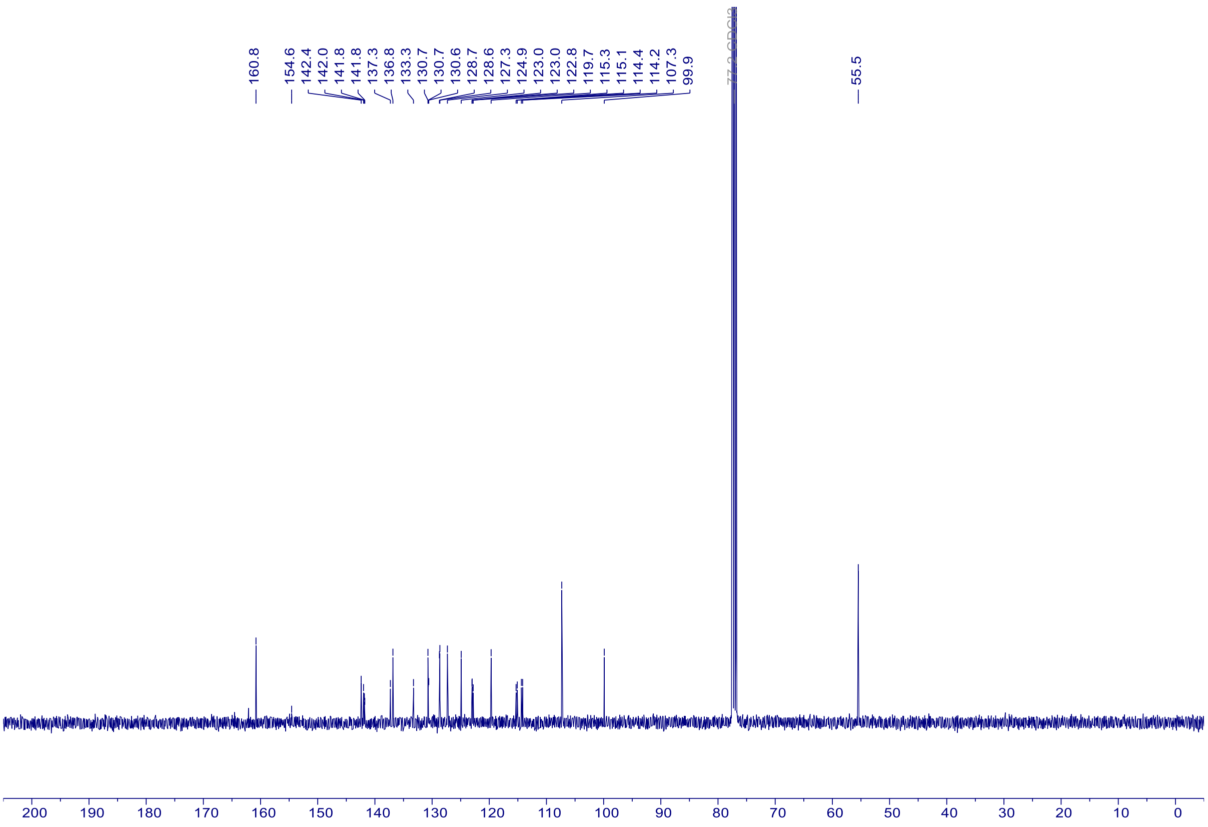

3ph – ^{19}F NMR (565 MHz, $CDCl_3$)

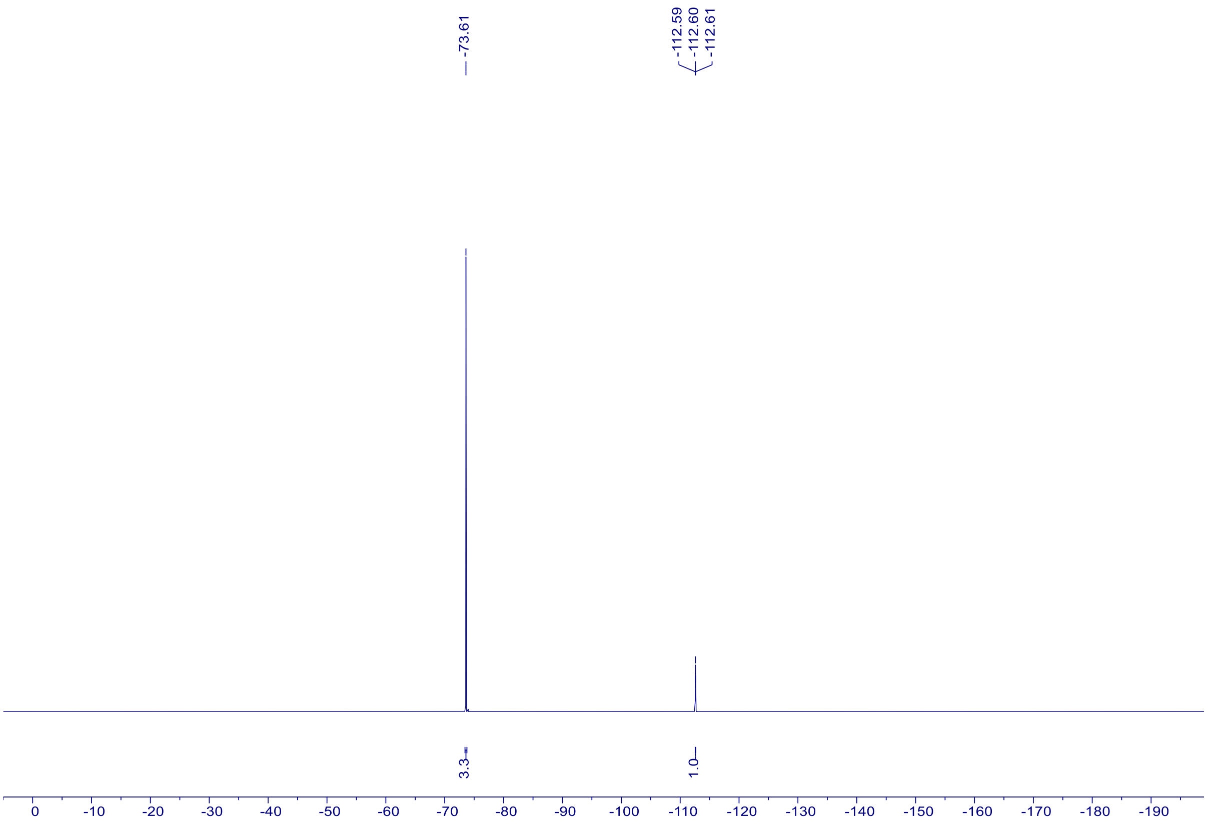

3qh´ – ^{1}H NMR (600 MHz, $CDCl_3$)

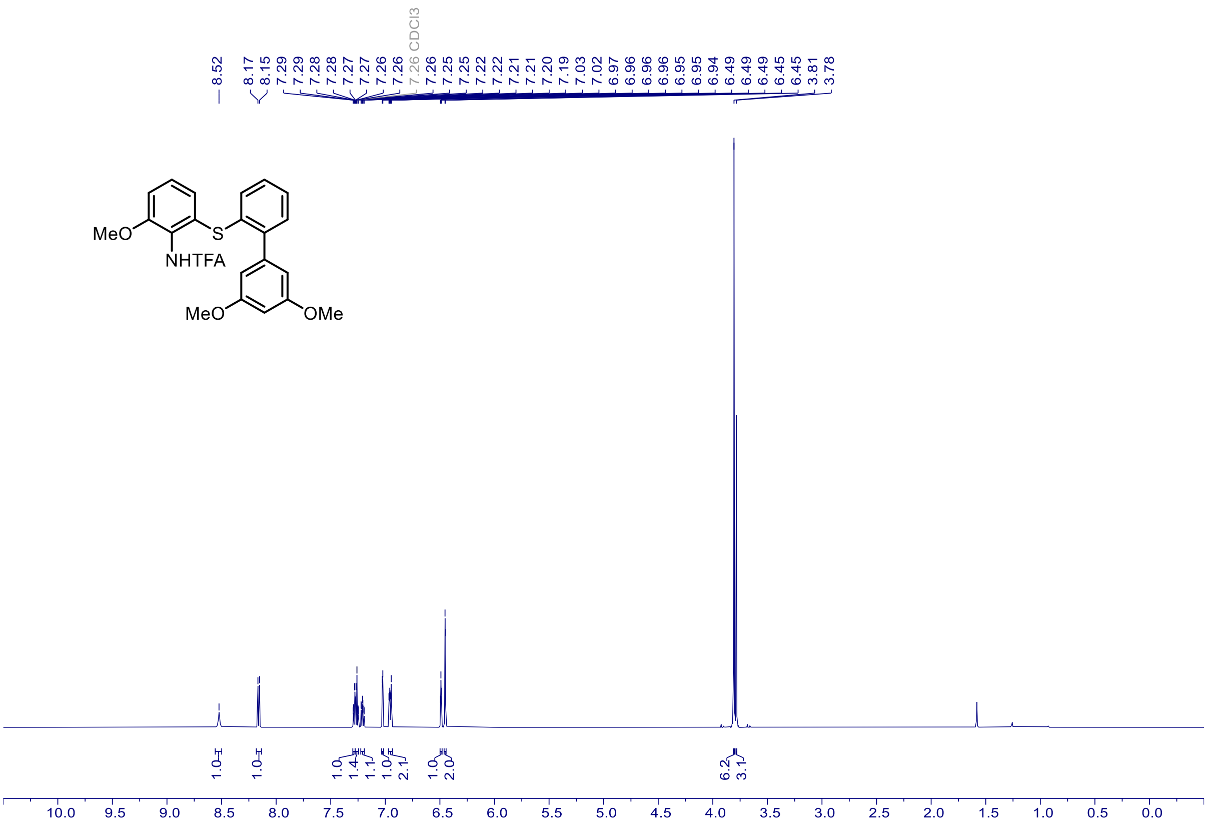

3qh´ – ^{13}C NMR (151 MHz, $CDCl_3$)

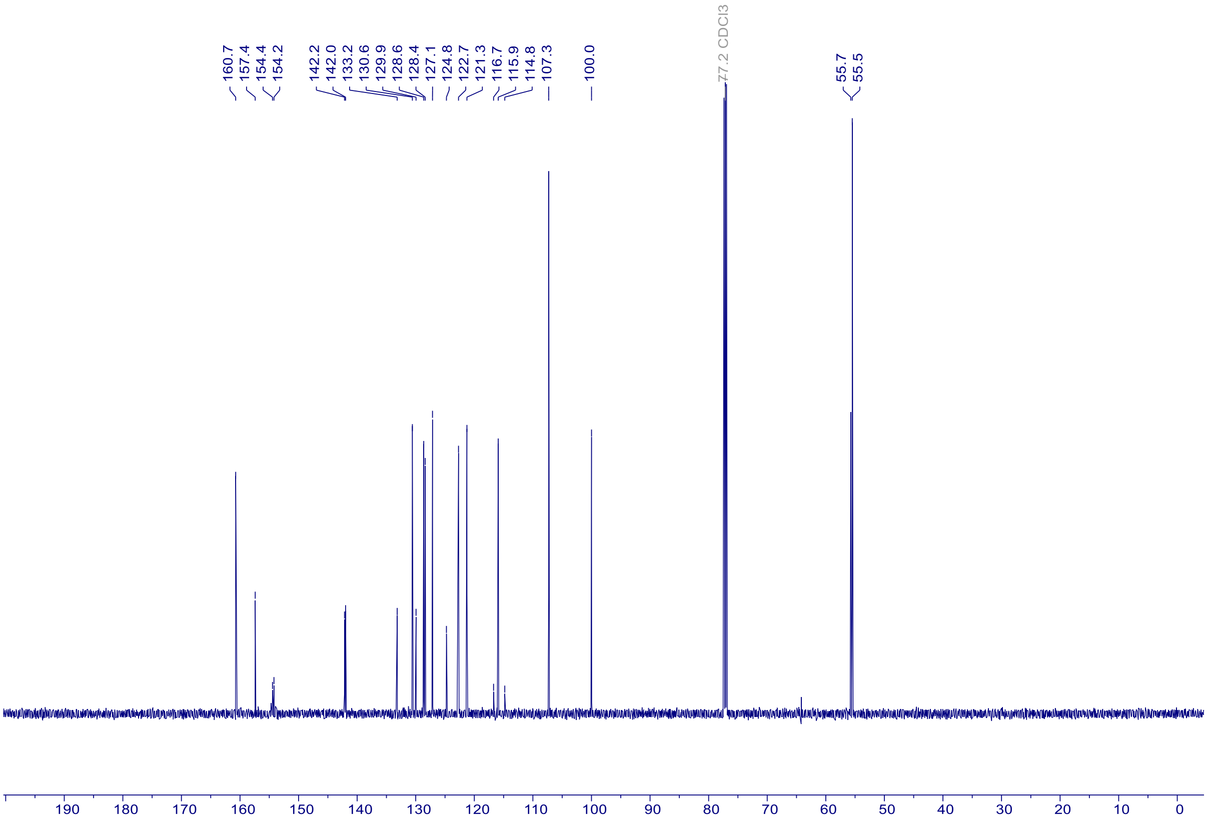

3qh´ – ^{19}F NMR (565 MHz, $CDCl_3$)

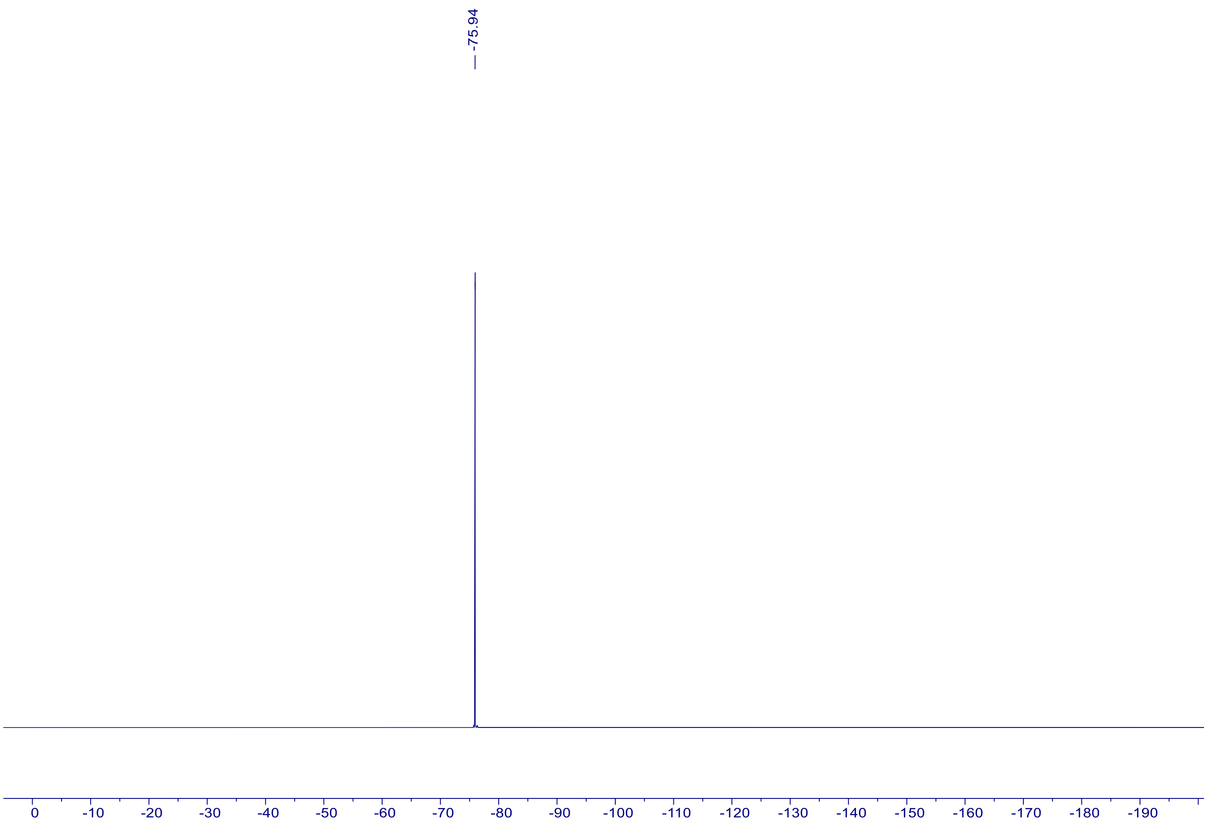

3rh – ^{1}H NMR (600 MHz, $CDCl_3$)

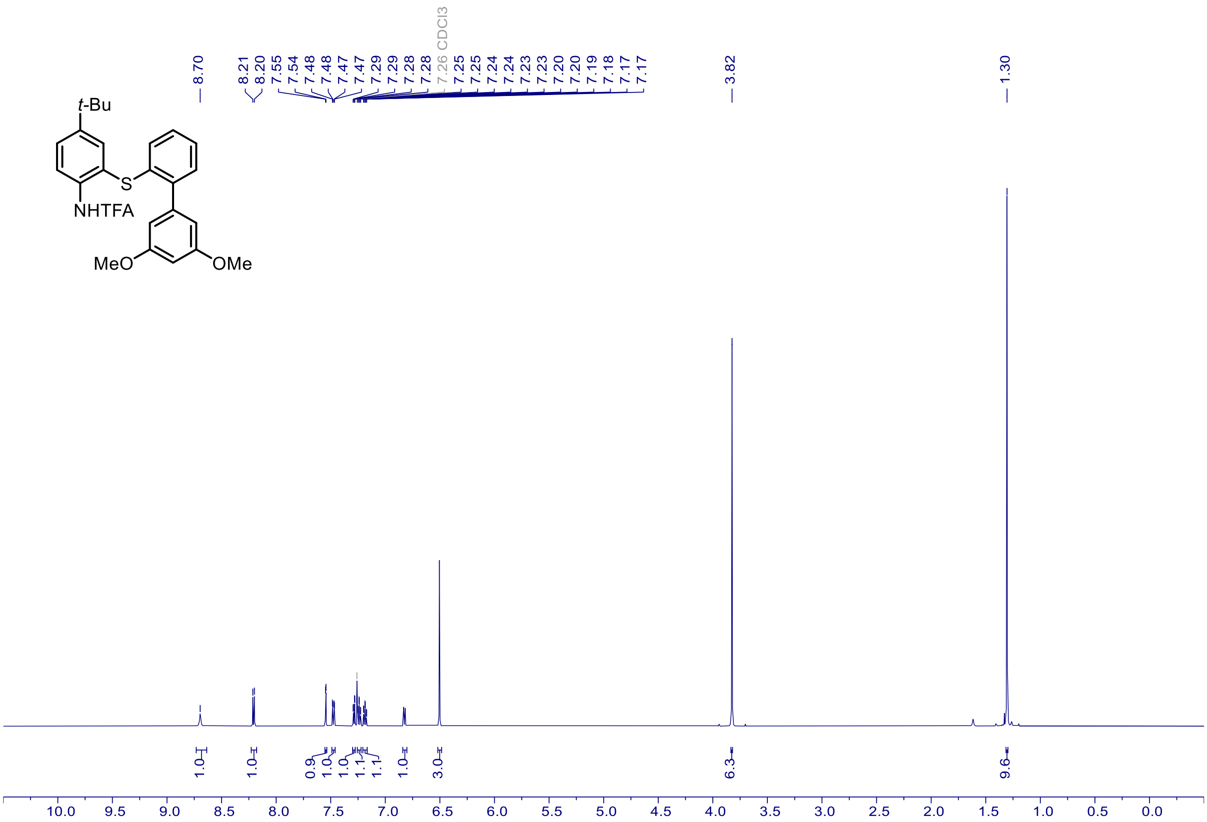

3rh – ^{13}C NMR (151 MHz, $CDCl_3$)

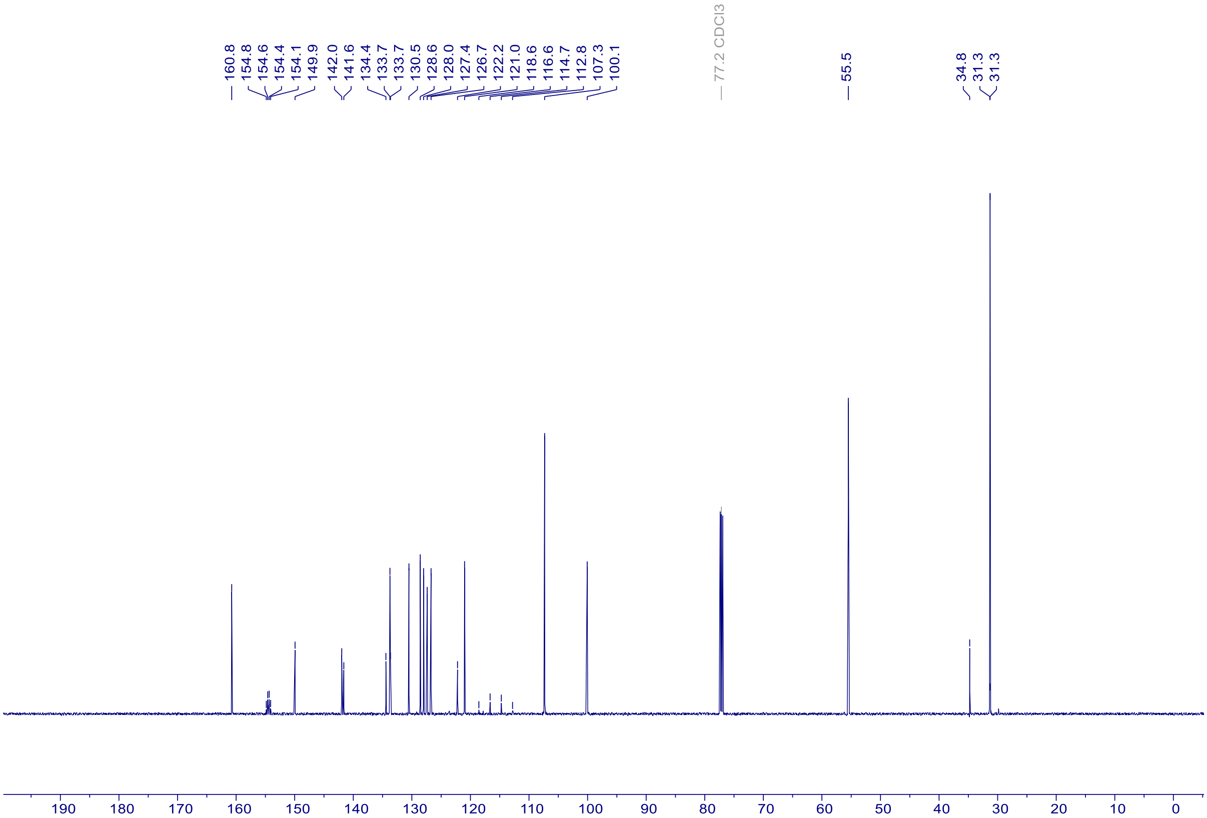

3rh – ^{19}F NMR (565 MHz, $CDCl_3$)

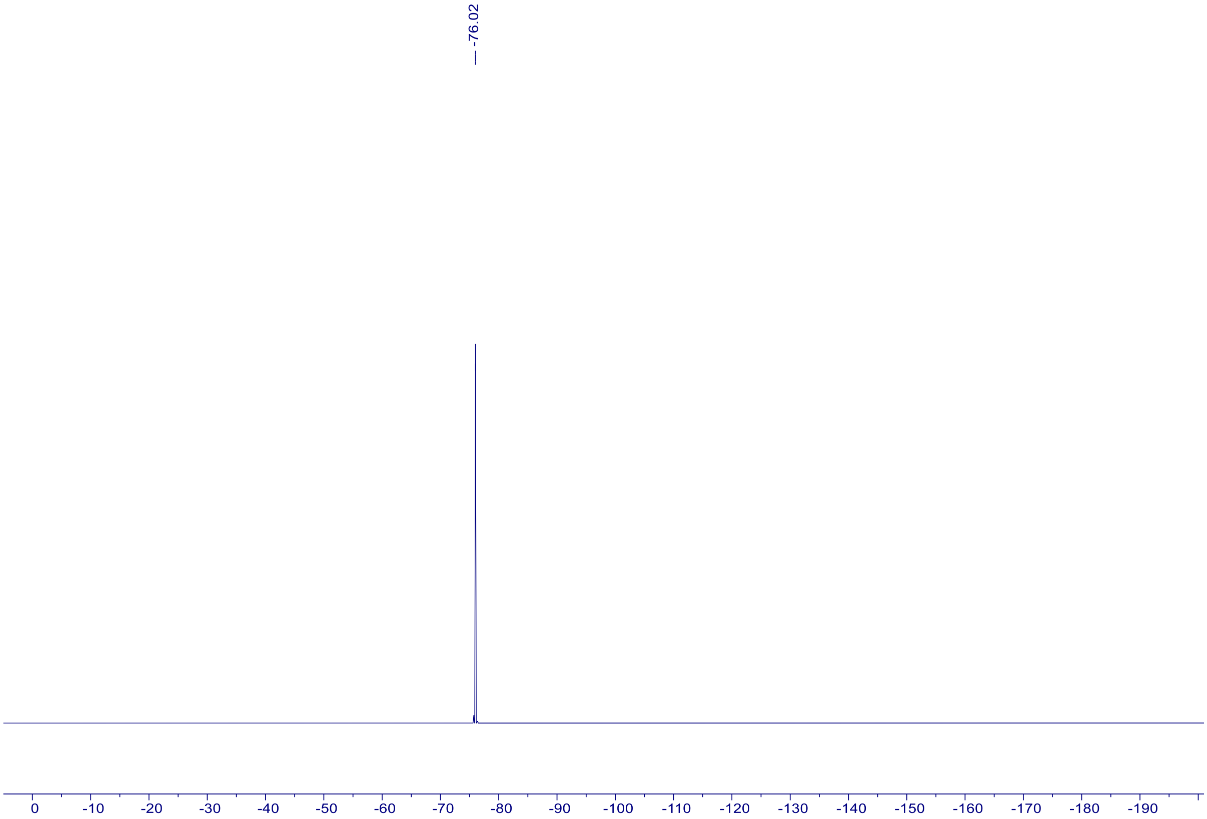

3rh′ – ^{1}H NMR (600 MHz, $CDCl_3$)

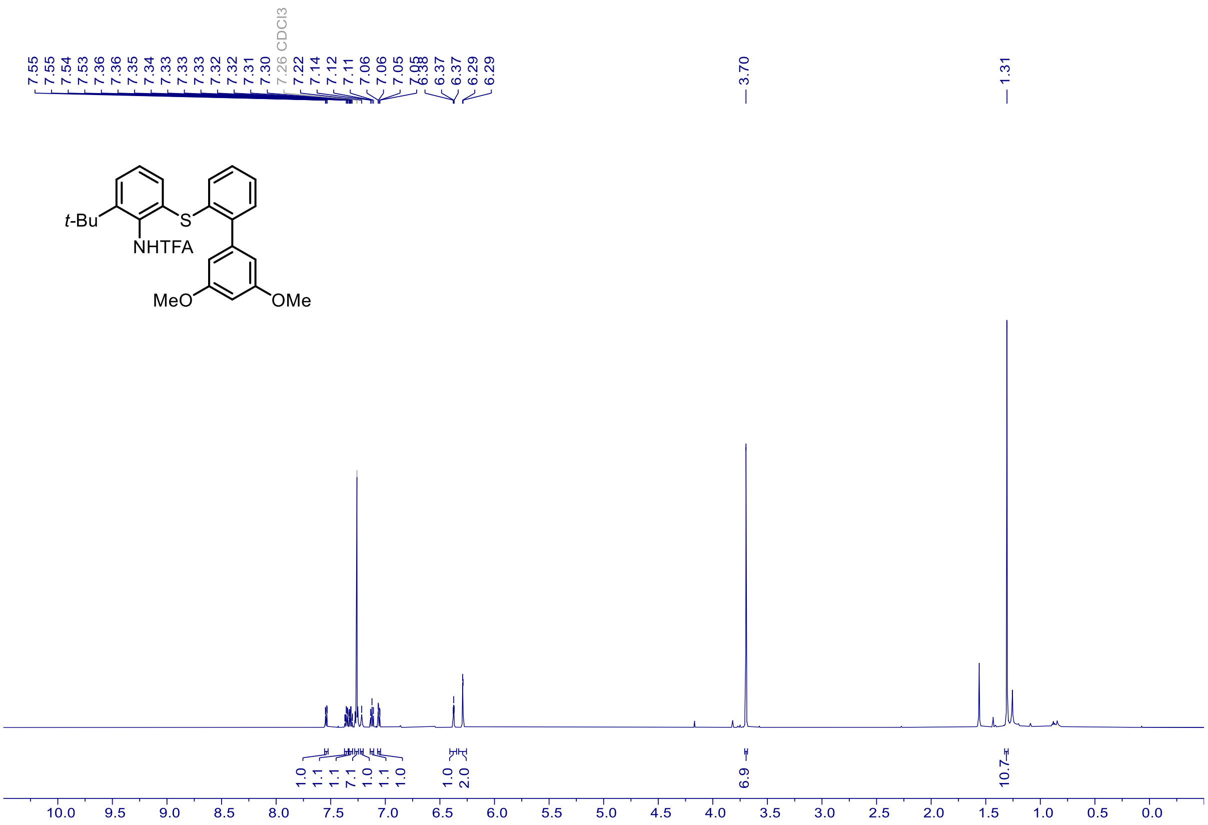

3rh′ – ^{13}C NMR (151 MHz, $CDCl_3$)

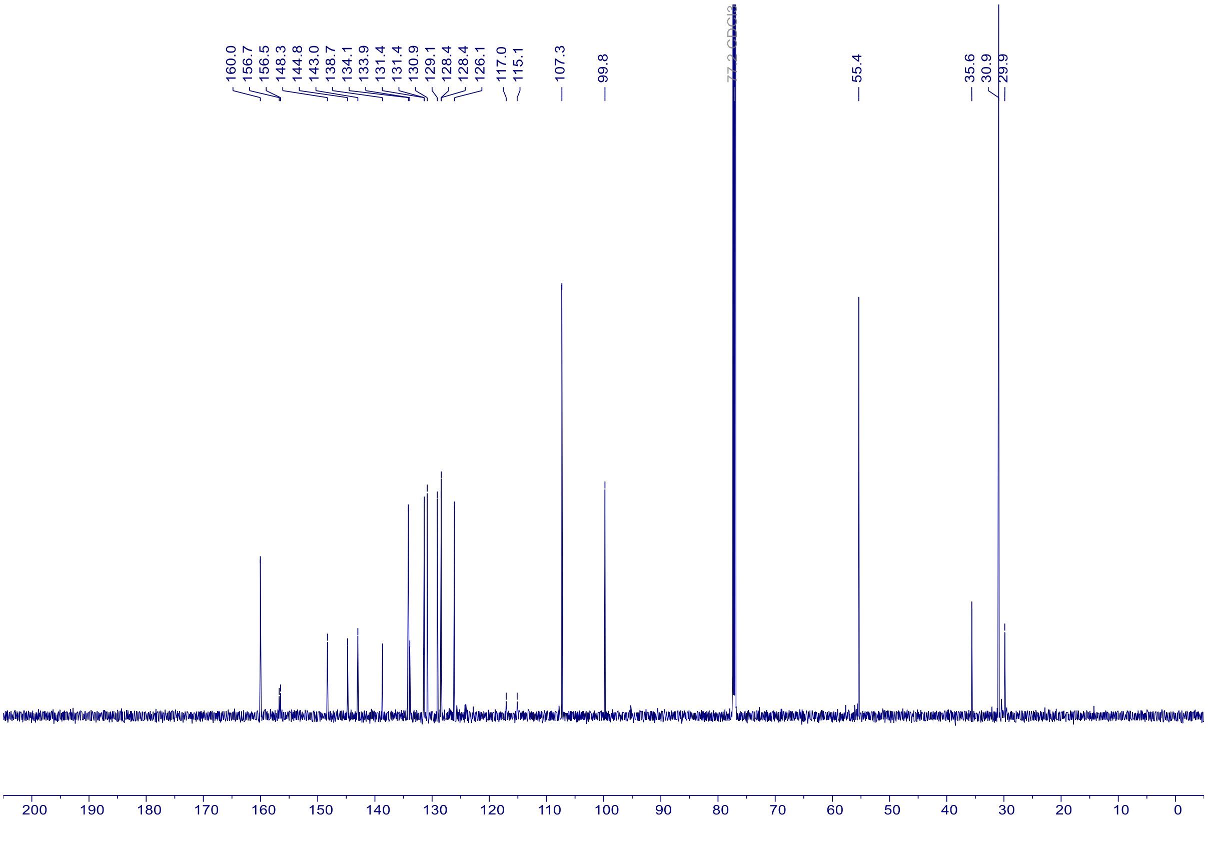

3rh′ – ^{19}F NMR (565 MHz, $CDCl_3$)

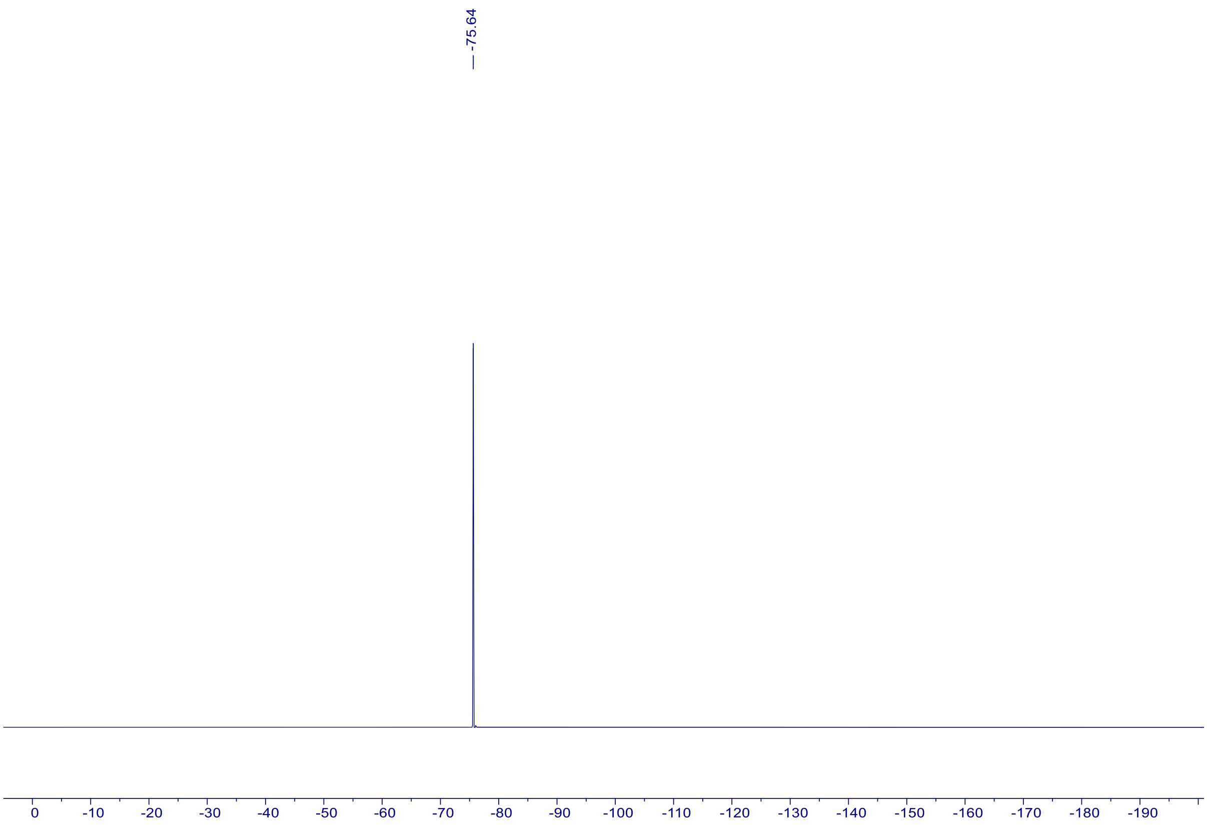

3sh – ^{1}H NMR (600 MHz, $CDCl_3$)

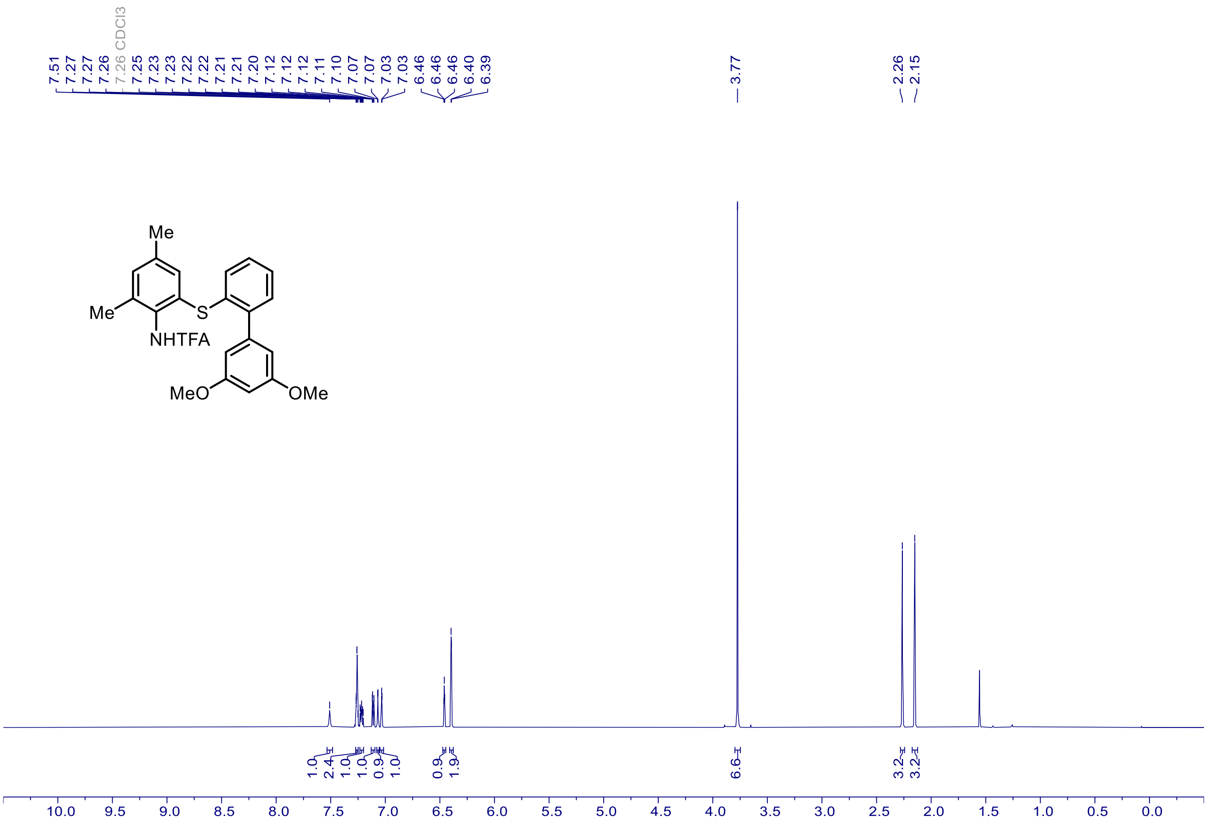

3sh – ^{13}C NMR (151 MHz, $CDCl_3$)

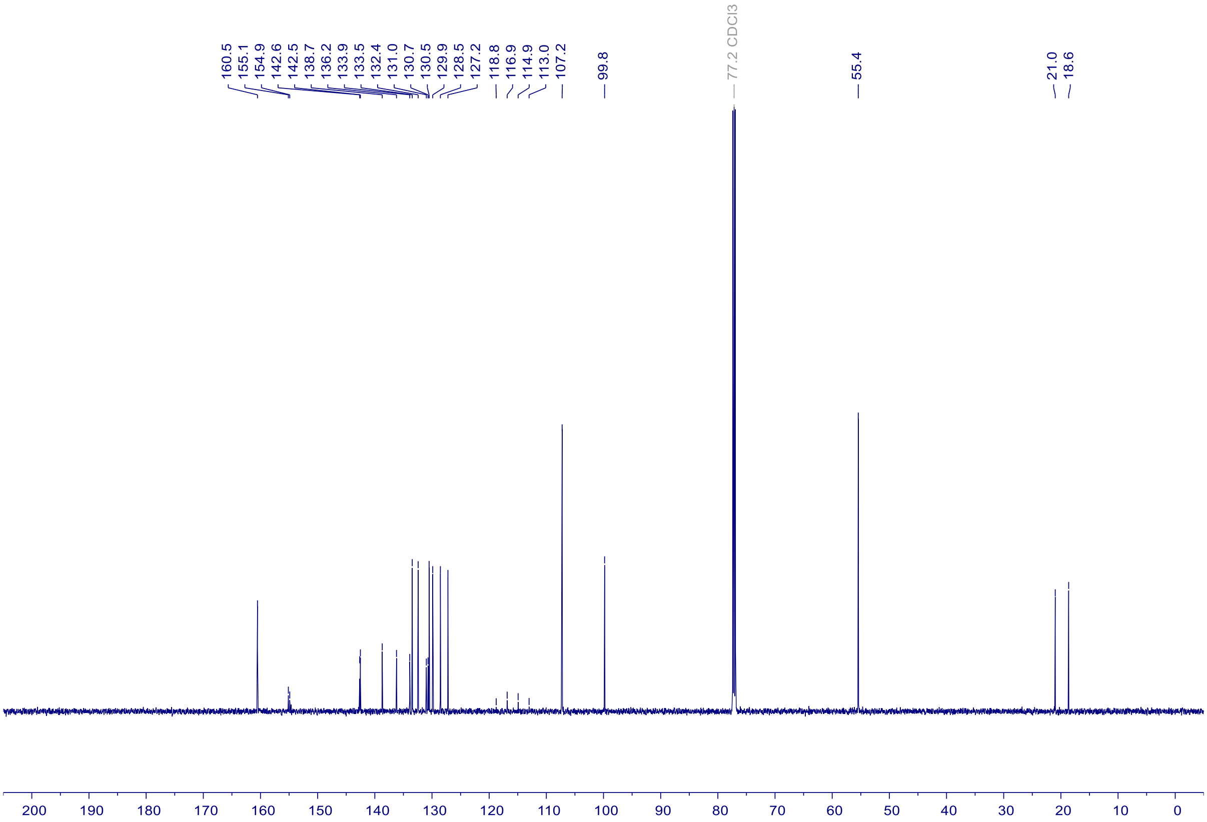

3sh – ^{19}F NMR (565 MHz, $CDCl_3$)

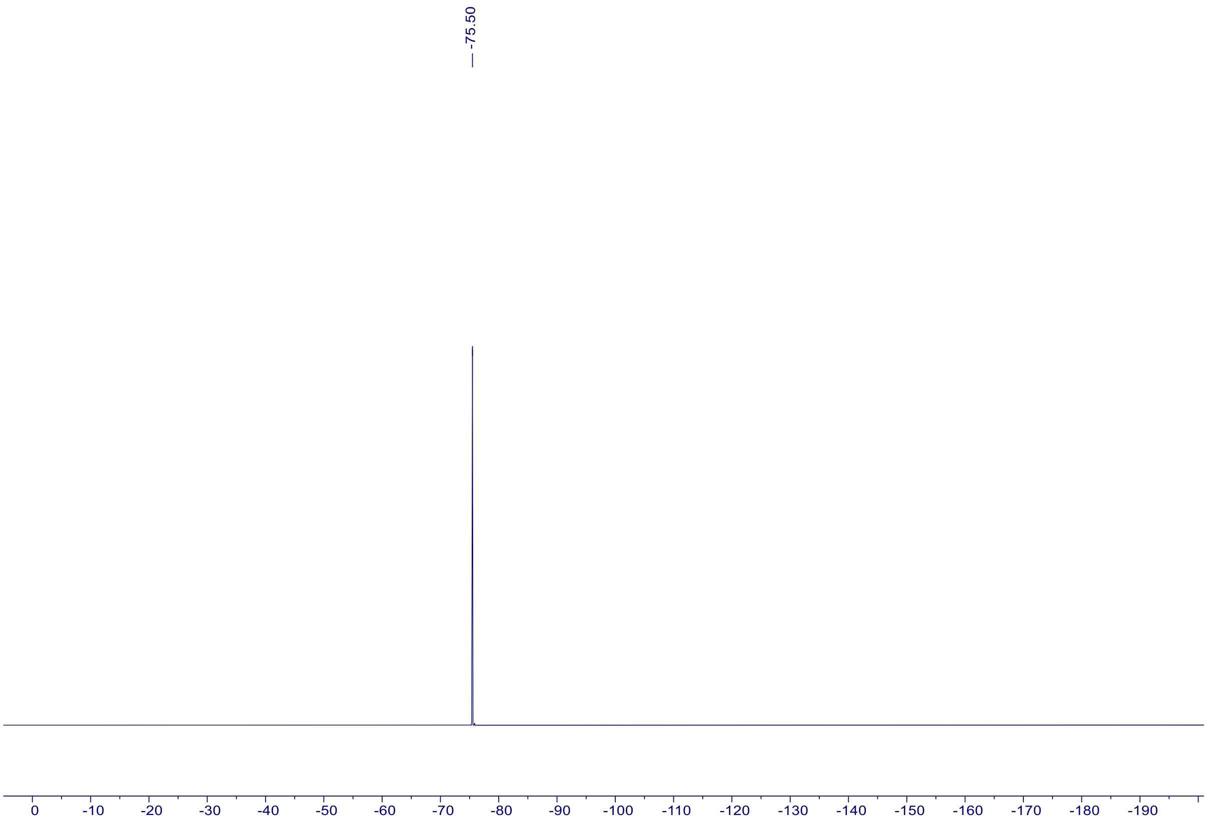

3th – ^{1}H NMR (600 MHz, $CDCl_3$)

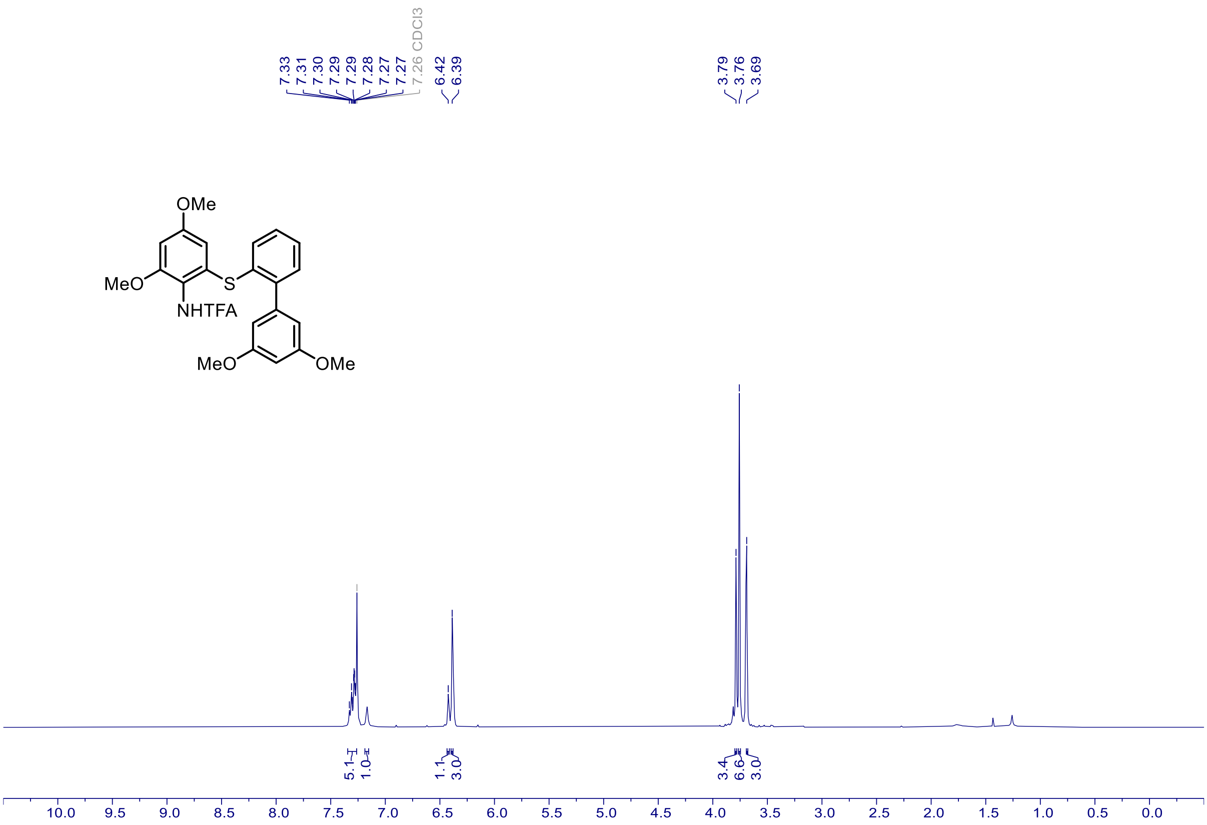

3th – ^{13}C NMR (151 MHz, $CDCl_3$)

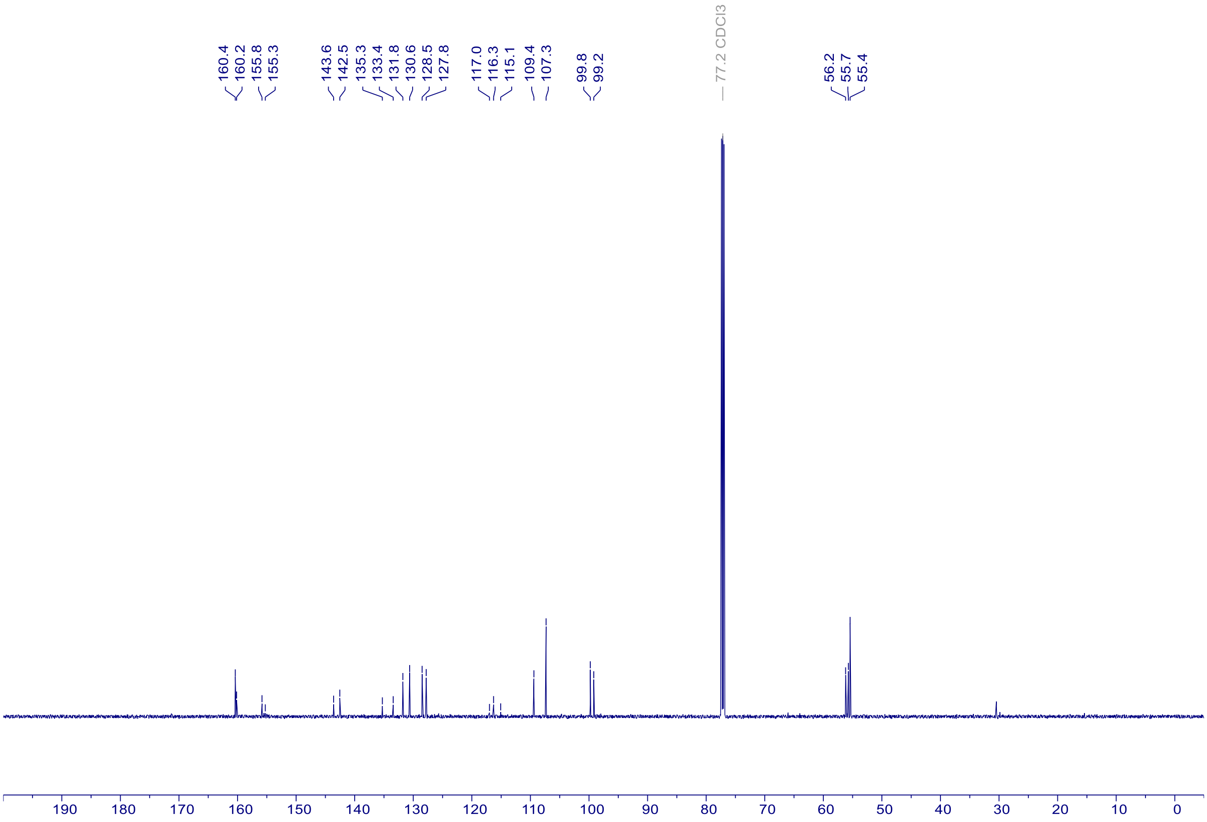

3th – ^{19}F NMR (565 MHz, $CDCl_3$)

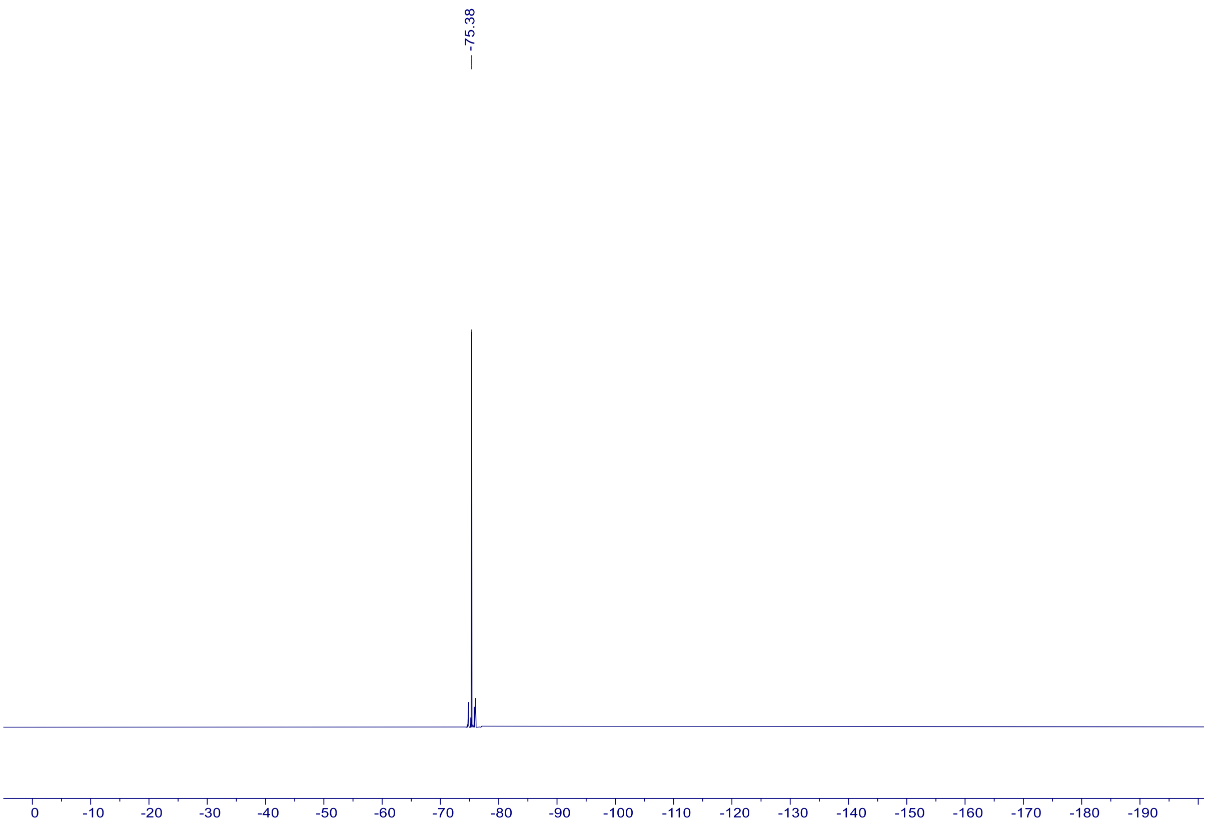

3uh – ^{1}H NMR (600 MHz, $CDCl_3$)

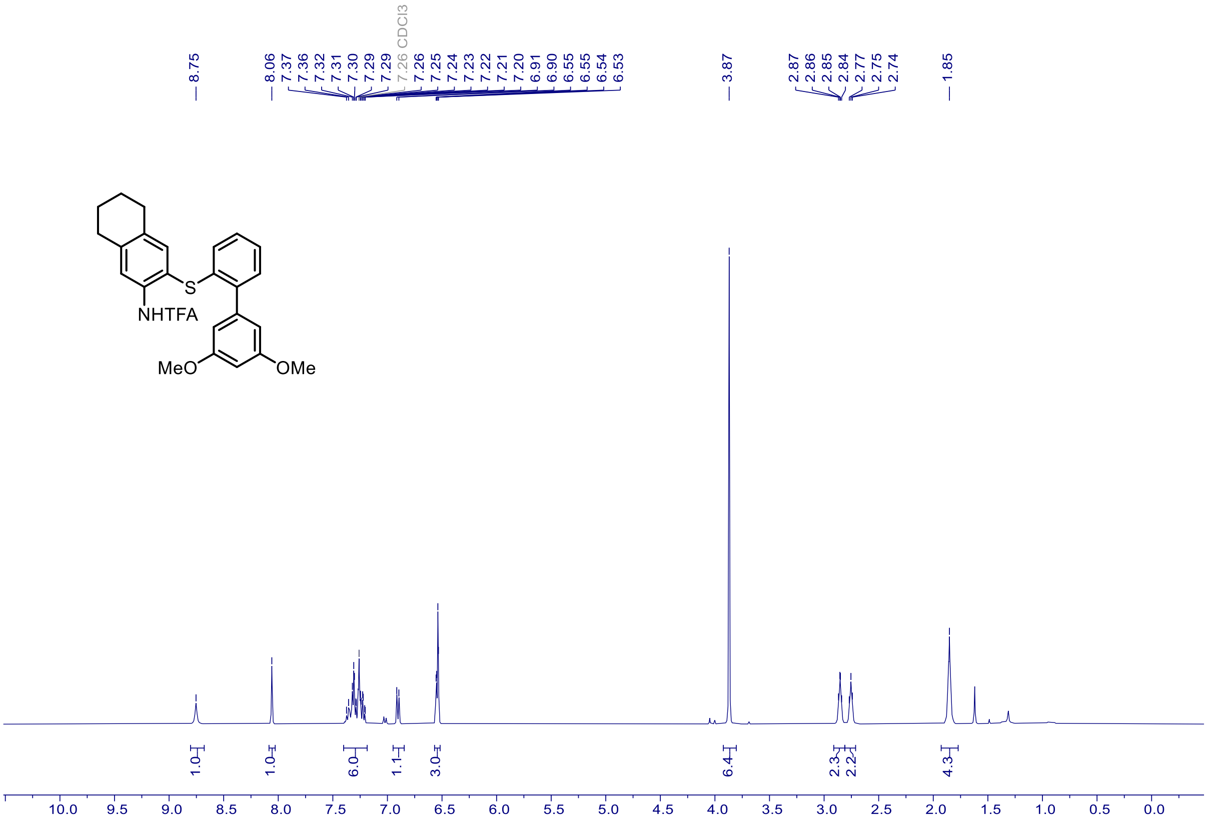

3uh – ^{13}C NMR (151 MHz, CDCl$_3$)

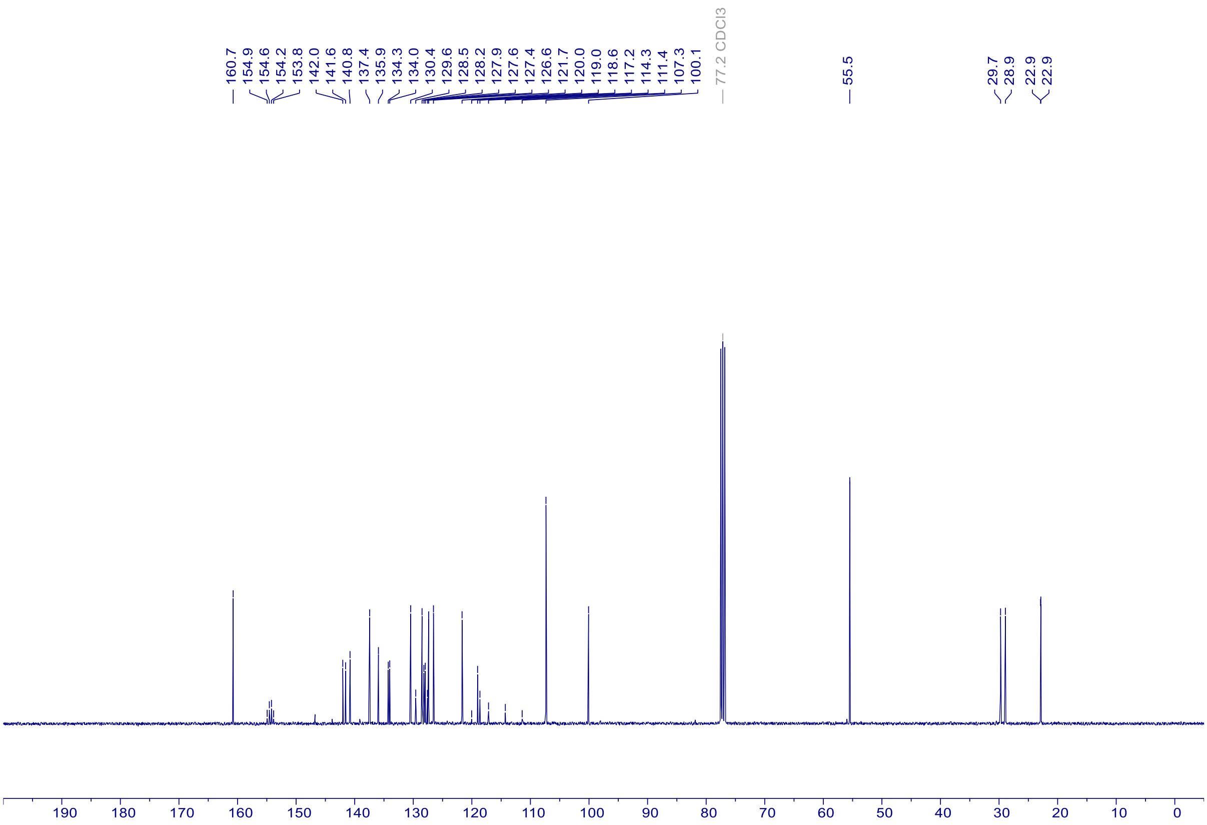

3uh – ^{19}F NMR (565 MHz, CDCl$_3$)

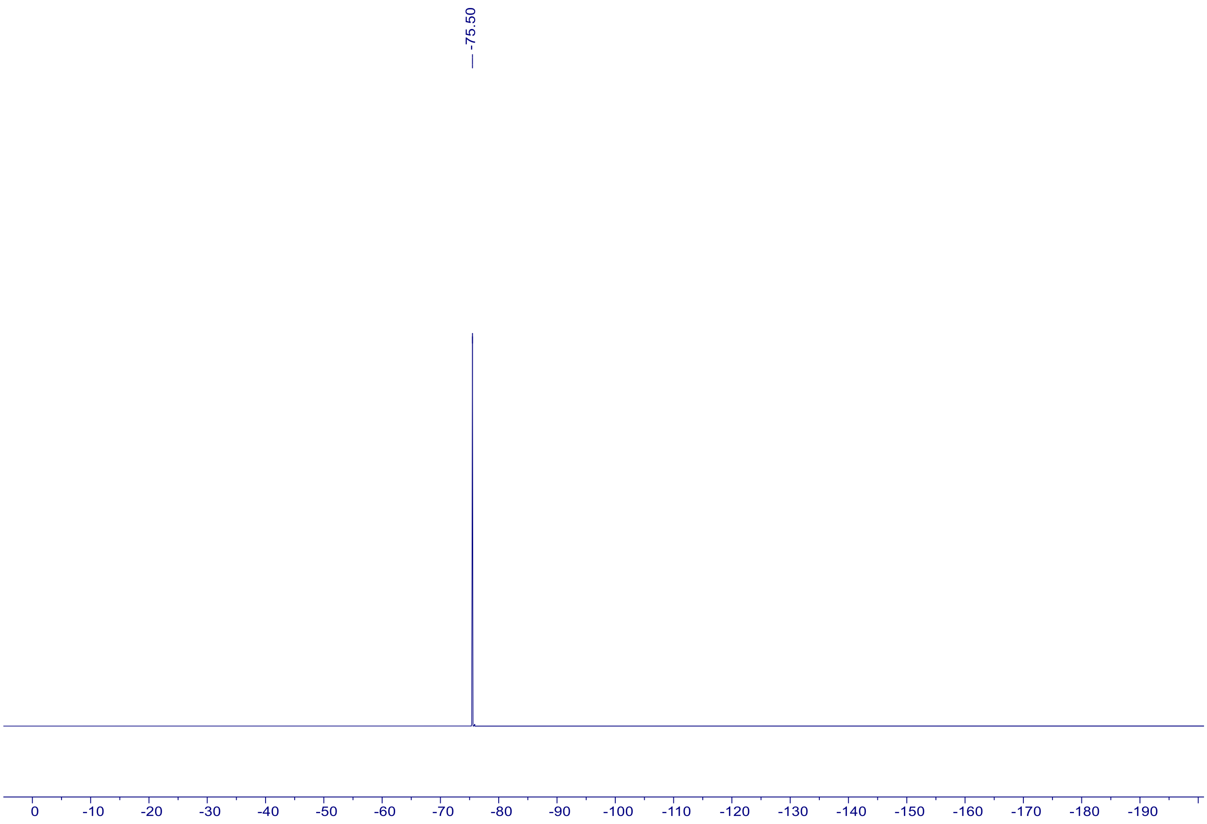

3uh´ – ^{1}H NMR (600 MHz, $CDCl_3$)

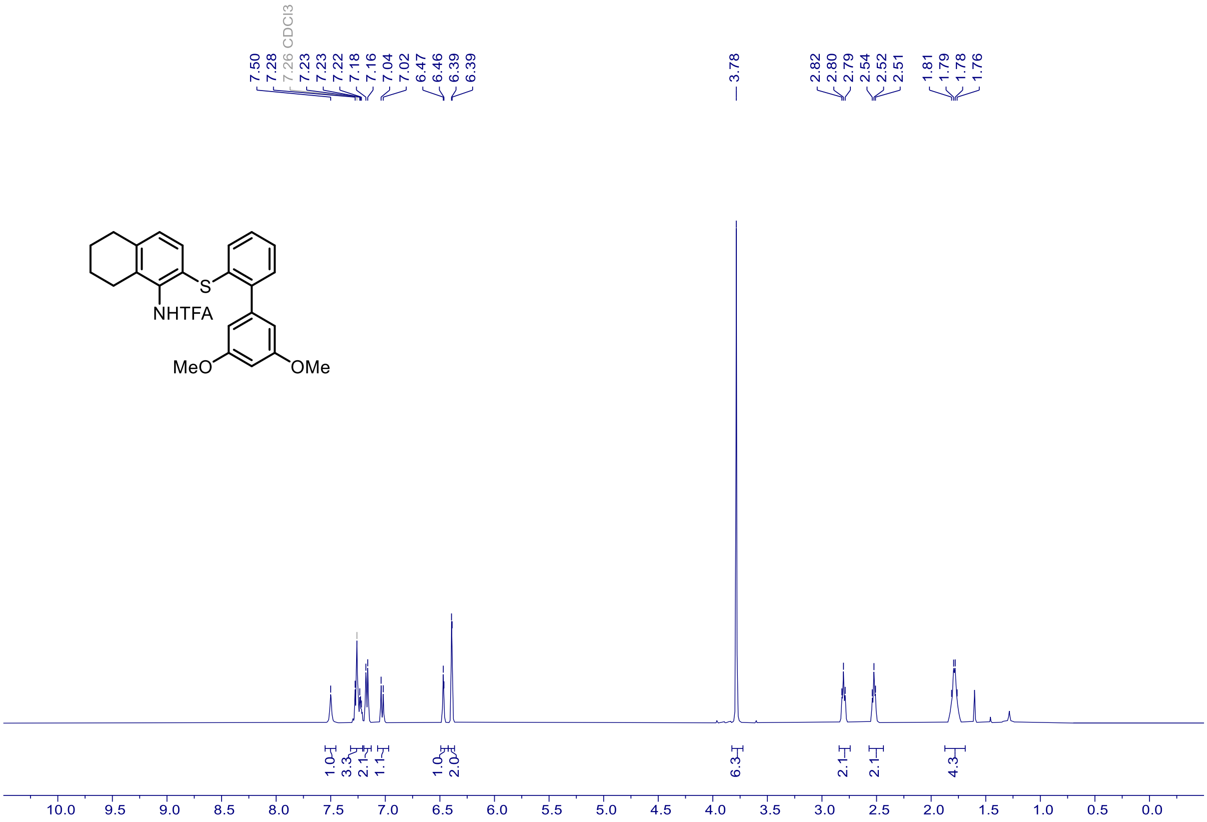

3uh´ – ^{13}C NMR (151 MHz, $CDCl_3$)

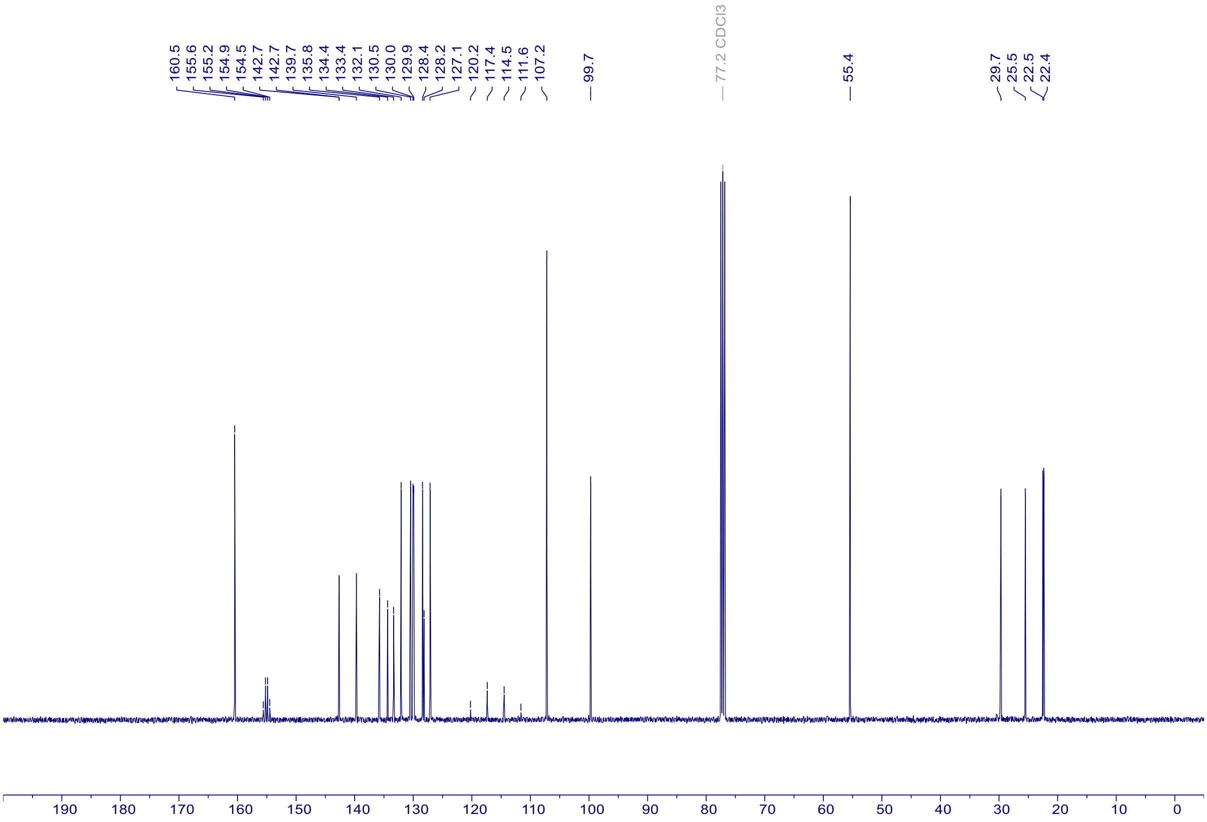

3uh′ – ^{19}F NMR (565 MHz, $CDCl_3$)

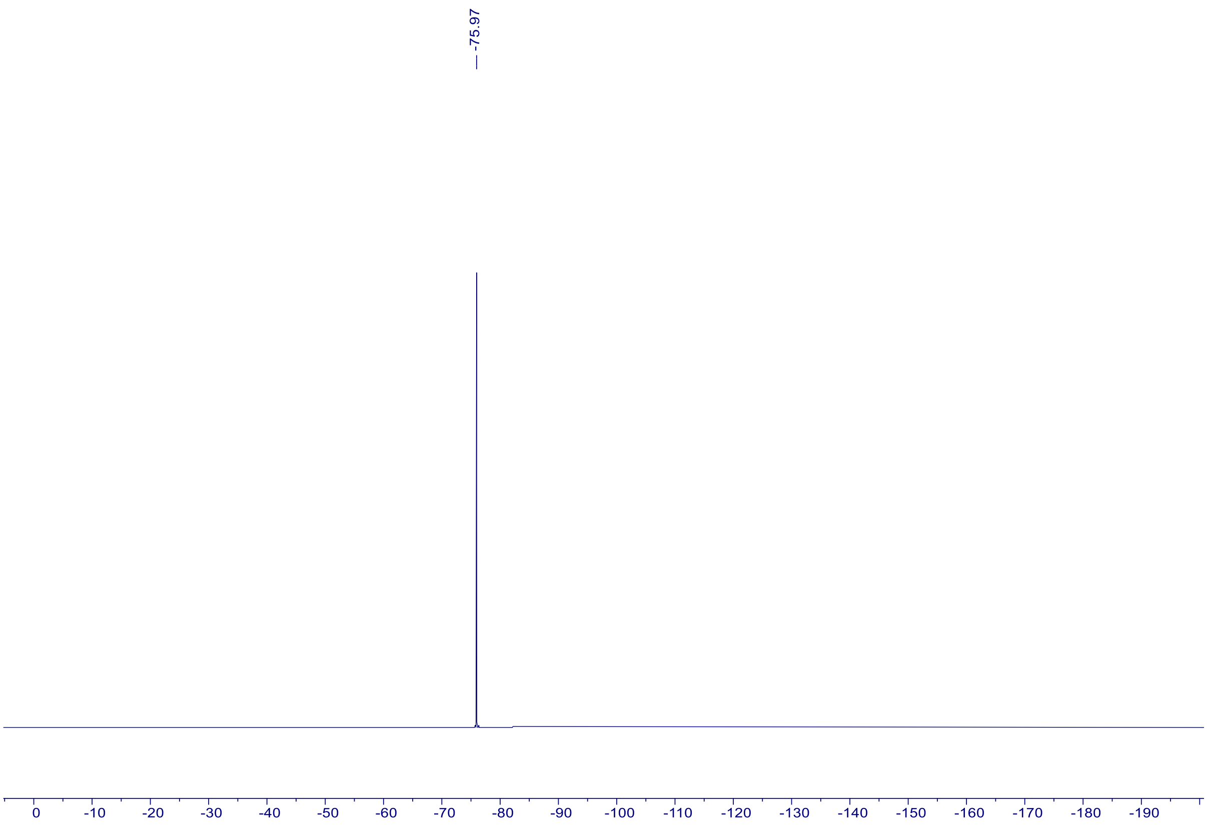

3vh – ^{1}H NMR (600 MHz, $CDCl_3$)

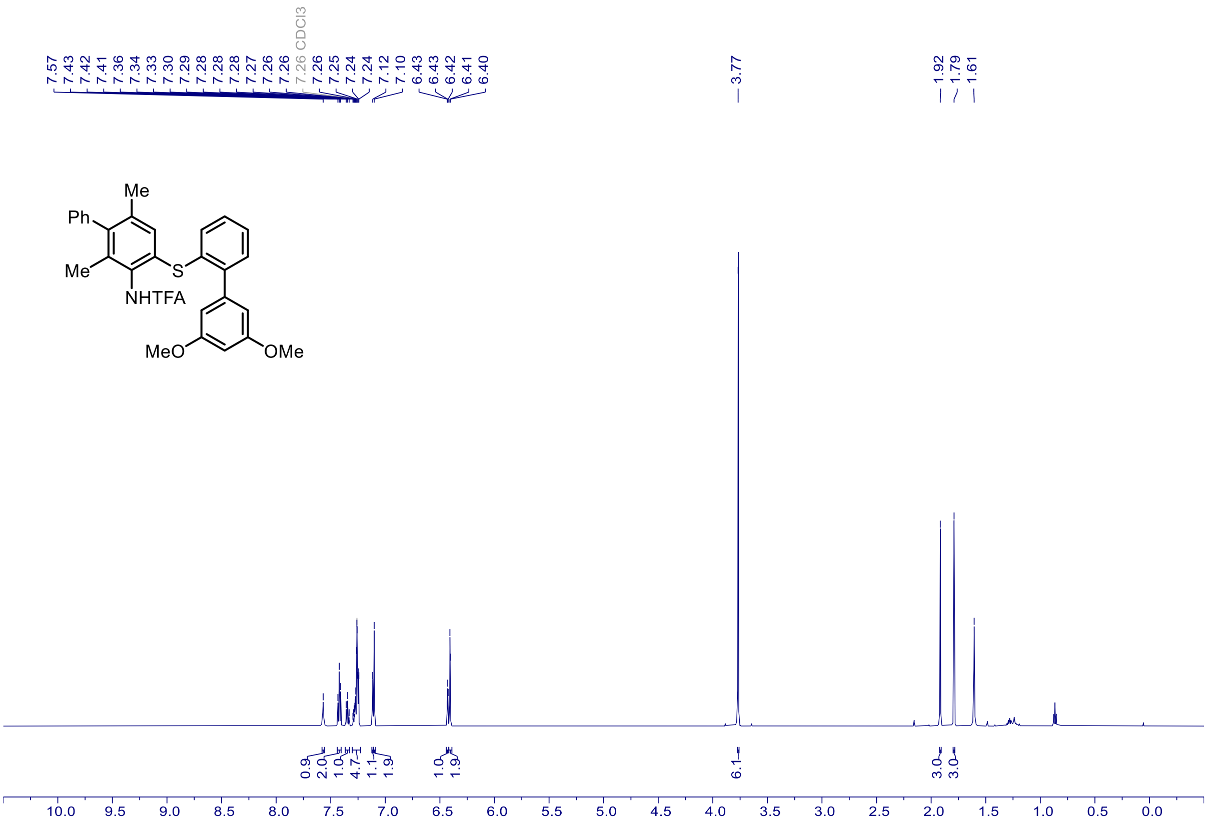

3vh – ^{13}C NMR (151 MHz, $CDCl_3$)

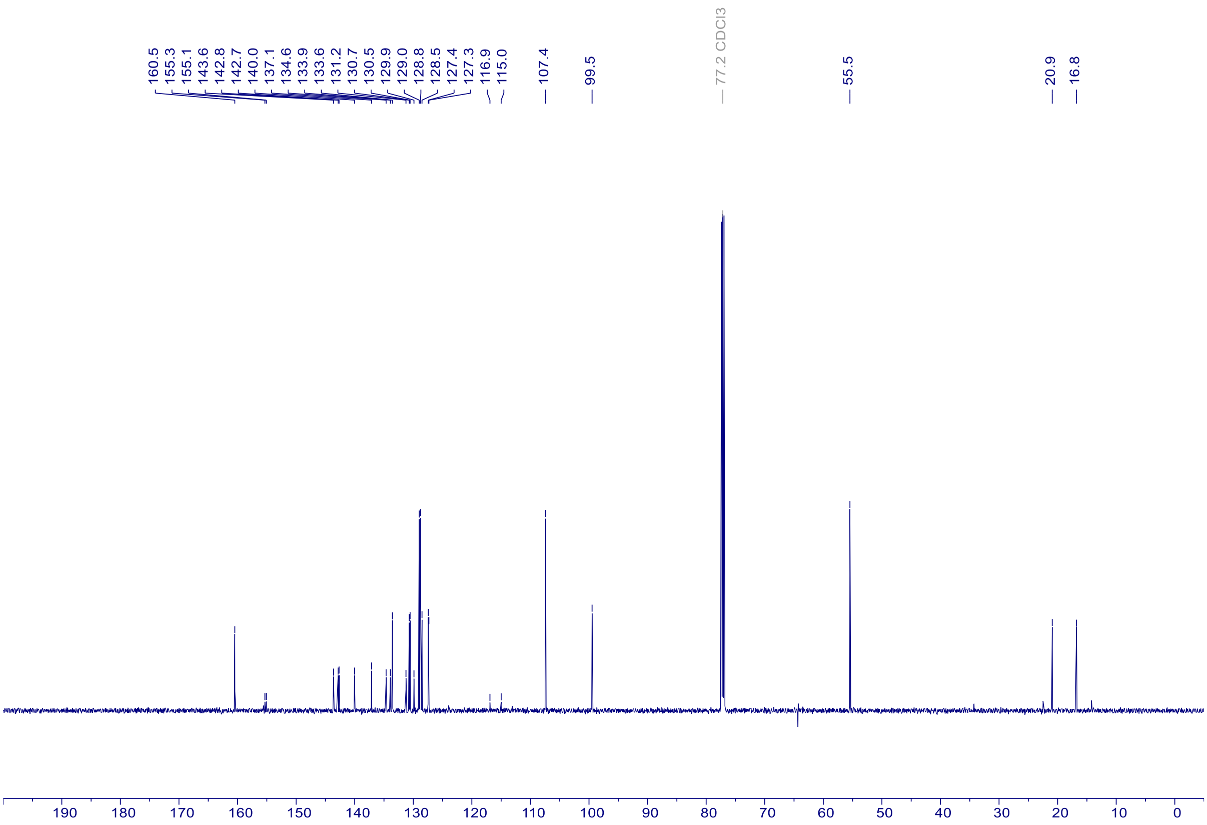

3vh – ^{19}F NMR (565 MHz, $CDCl_3$)

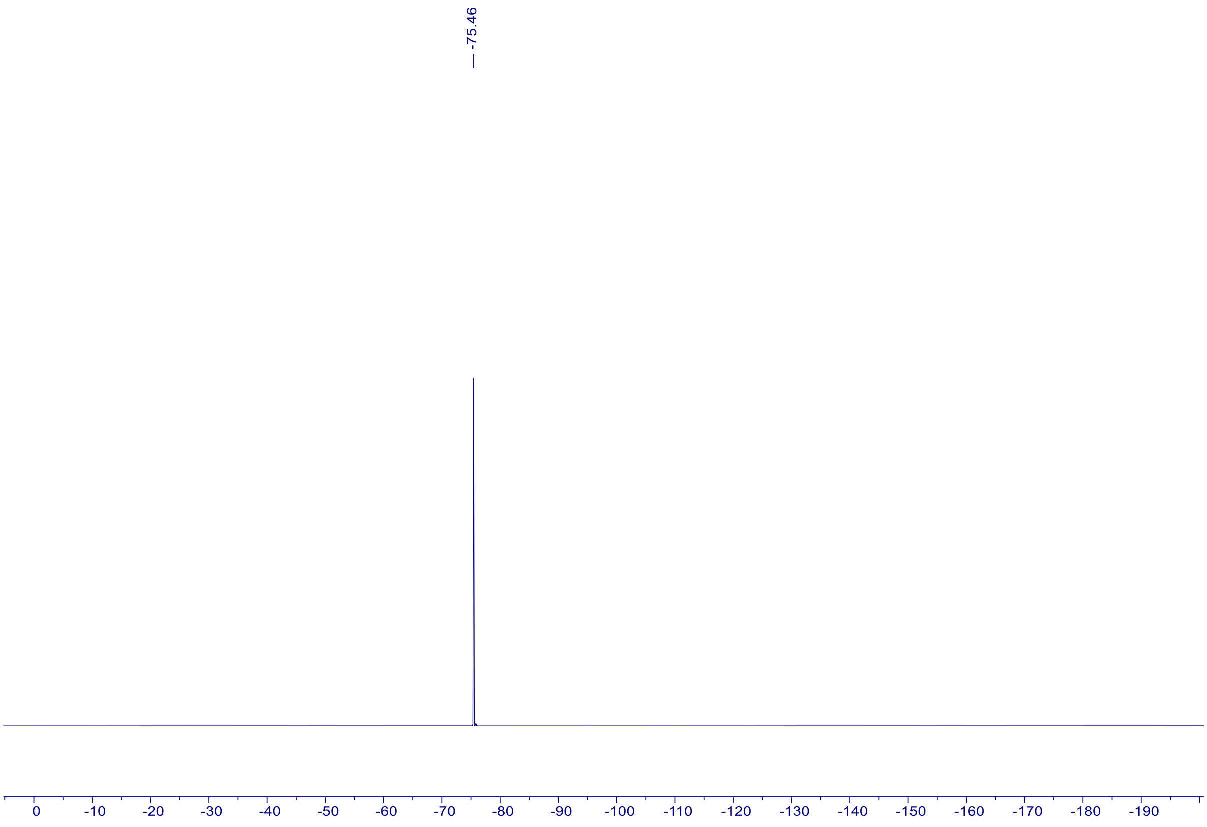

3wh – ^{1}H NMR (600 MHz, $CDCl_3$)

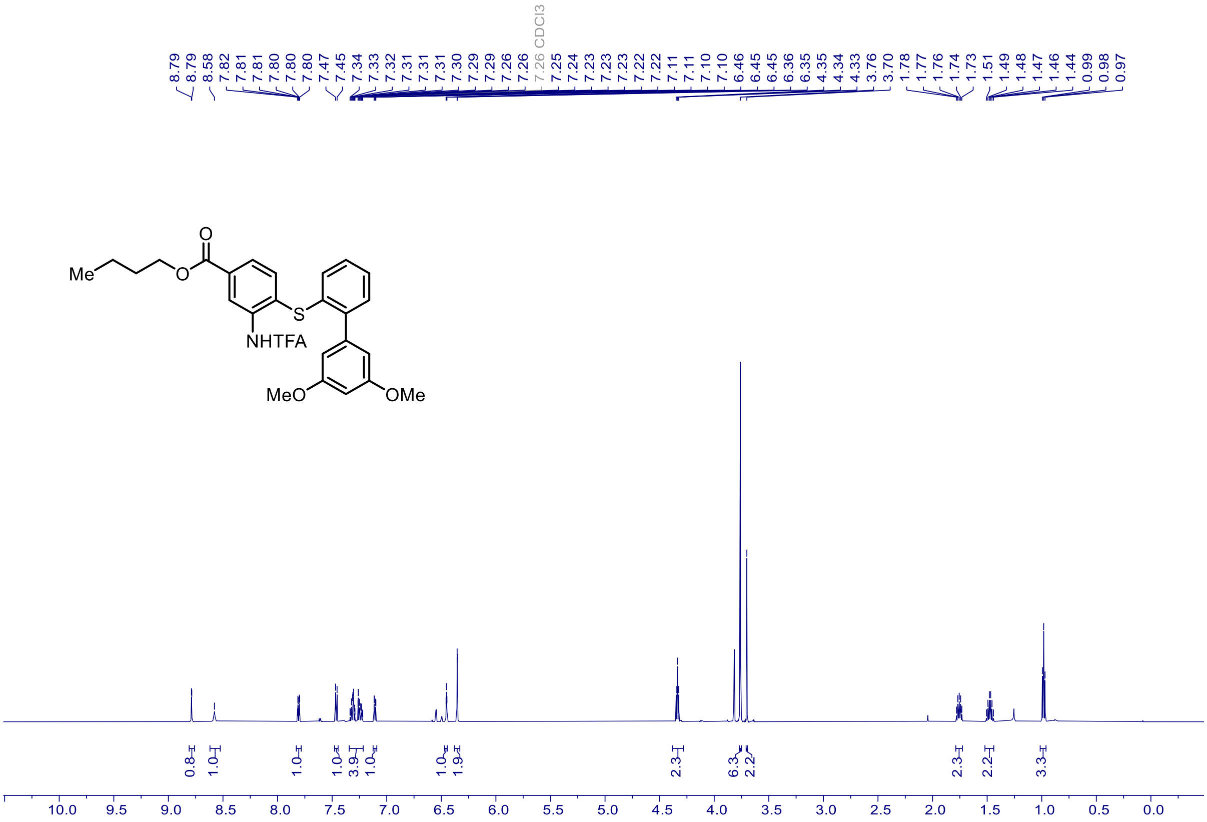

3wh – ^{13}C NMR (151 MHz, $CDCl_3$)

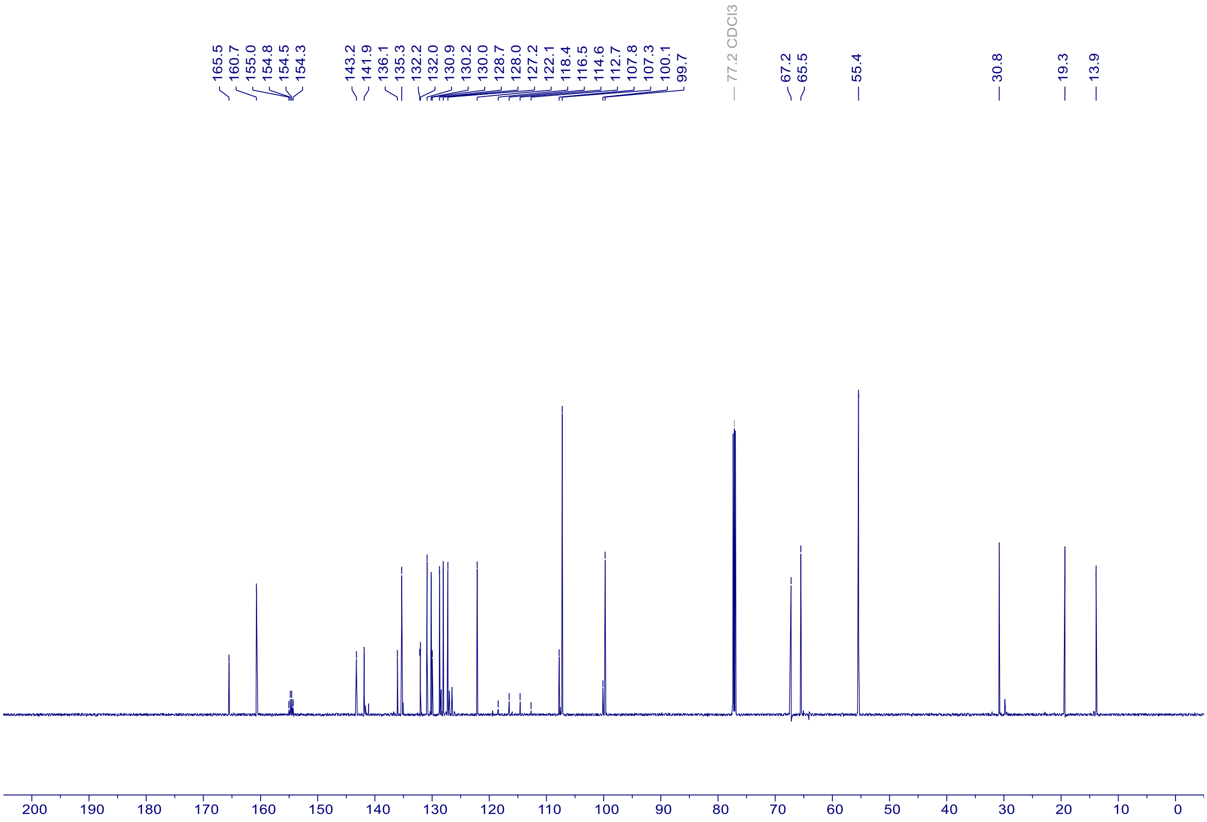

3wh – ^{19}F NMR (565 MHz, $CDCl_3$)

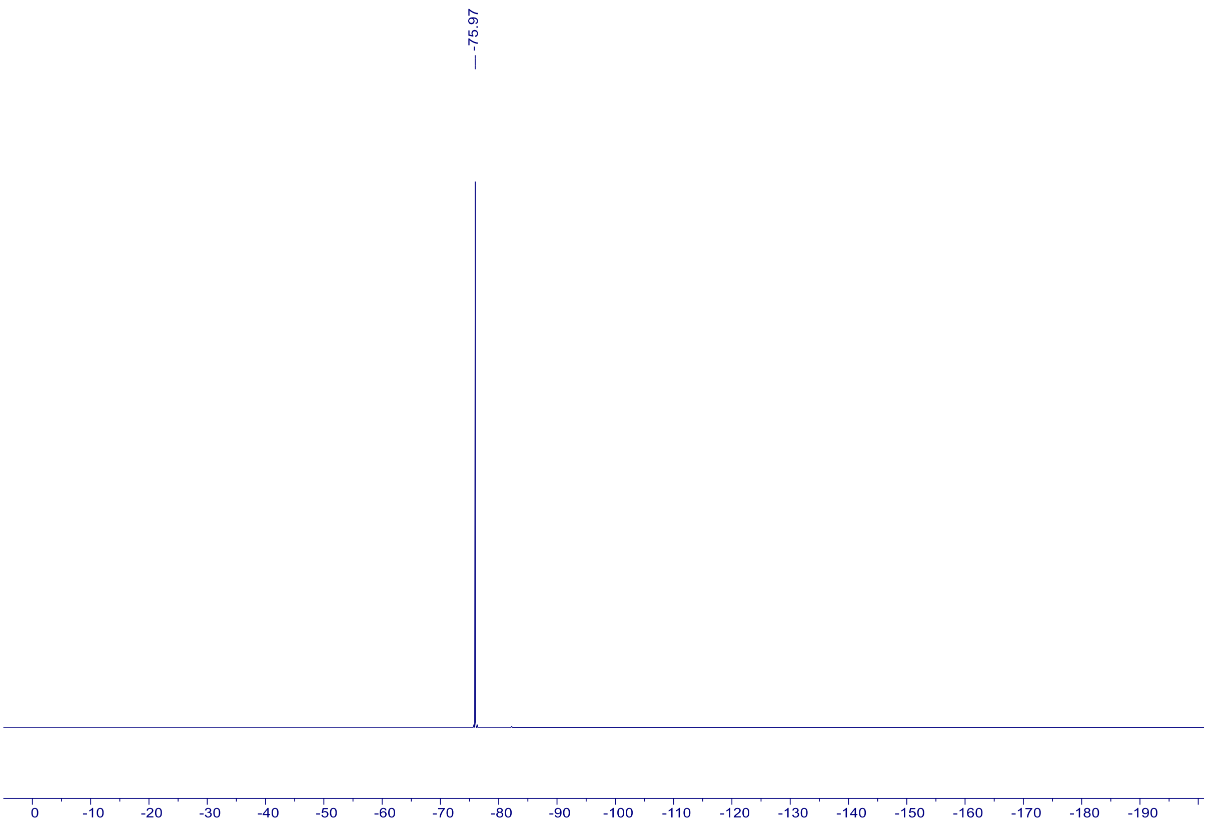

3xh – ^{1}H NMR (600 MHz, $CDCl_3$)

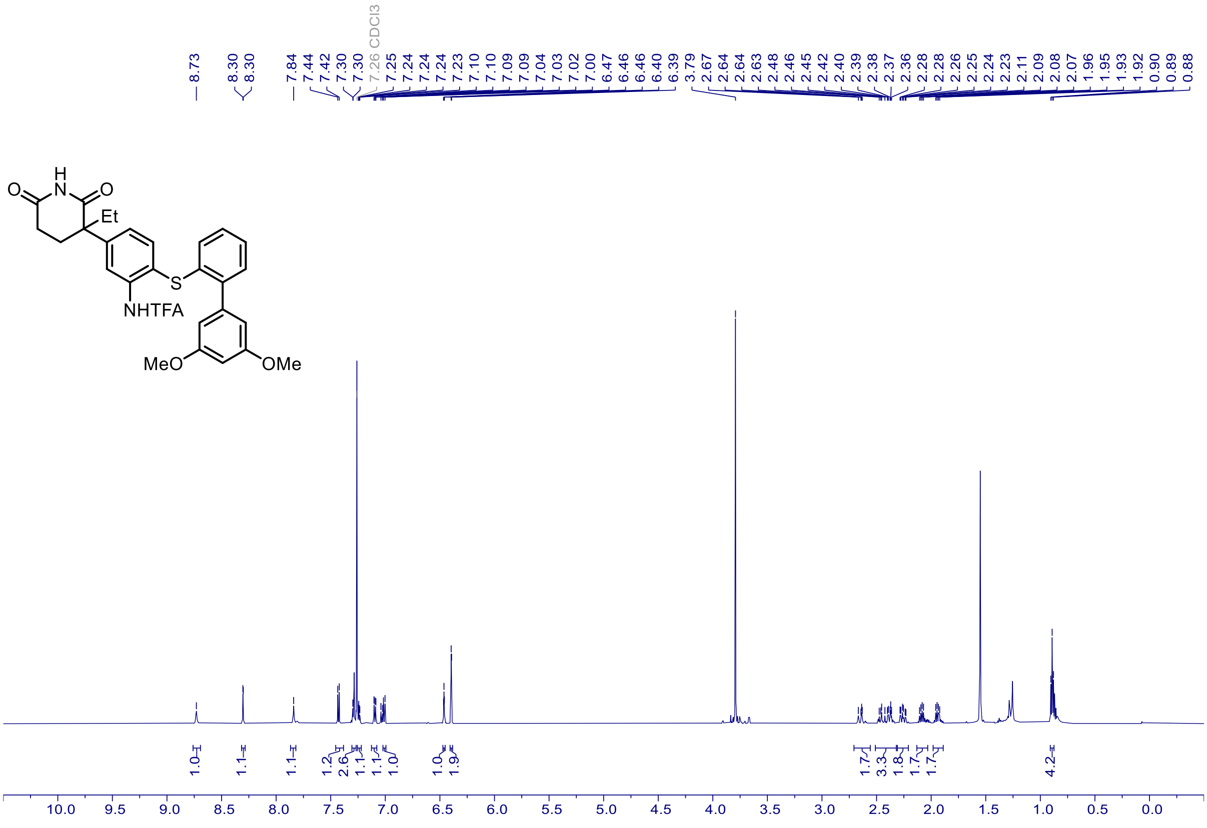

3xh – ^{13}C NMR (151 MHz, $CDCl_3$)

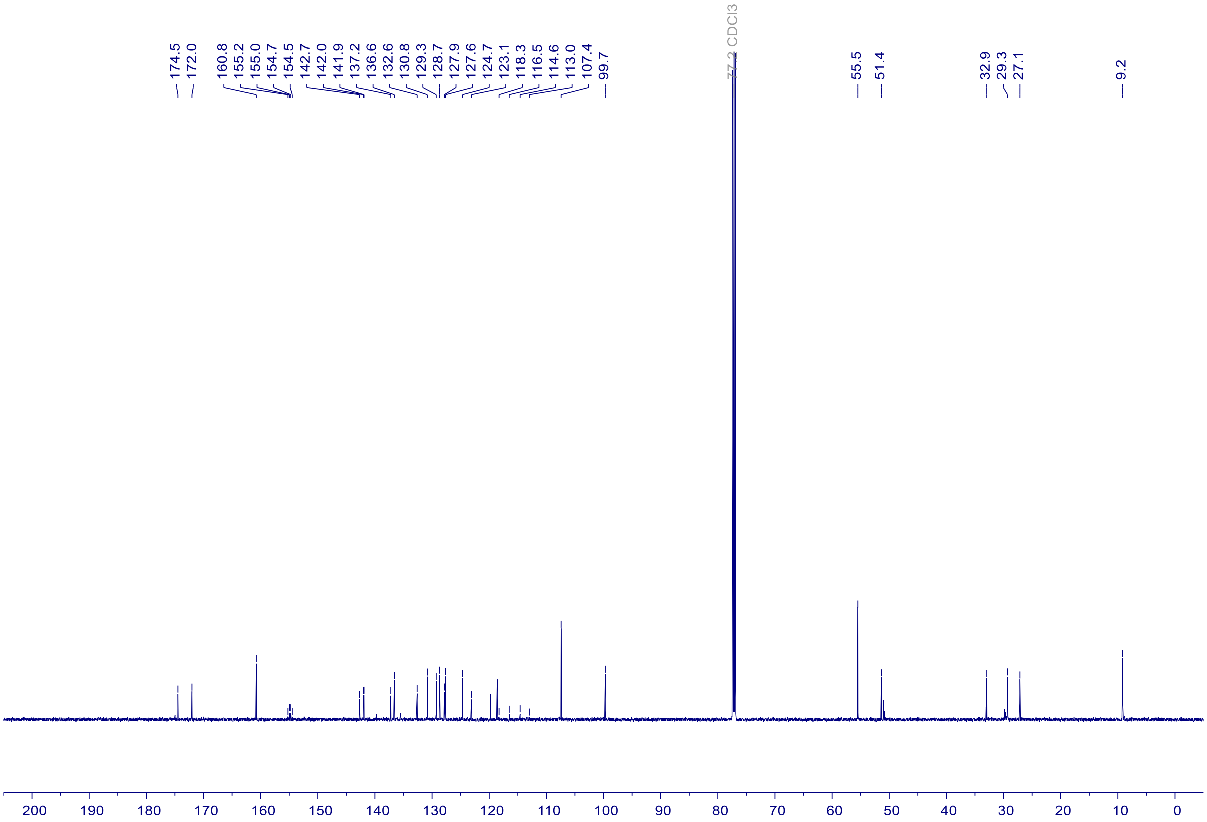

3xh – ^{19}F NMR (565 MHz, $CDCl_3$)

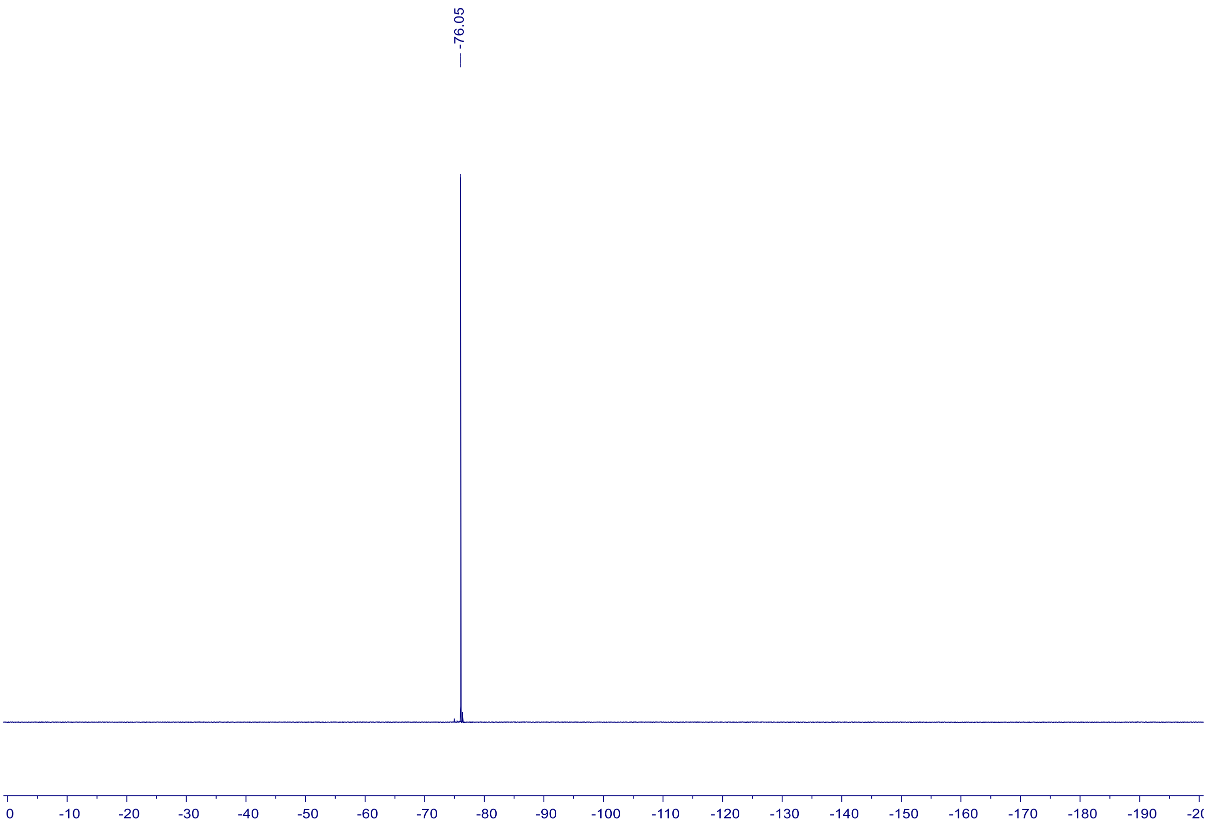

3yh – ^{1}H NMR (600 MHz, CDCl$_3$)

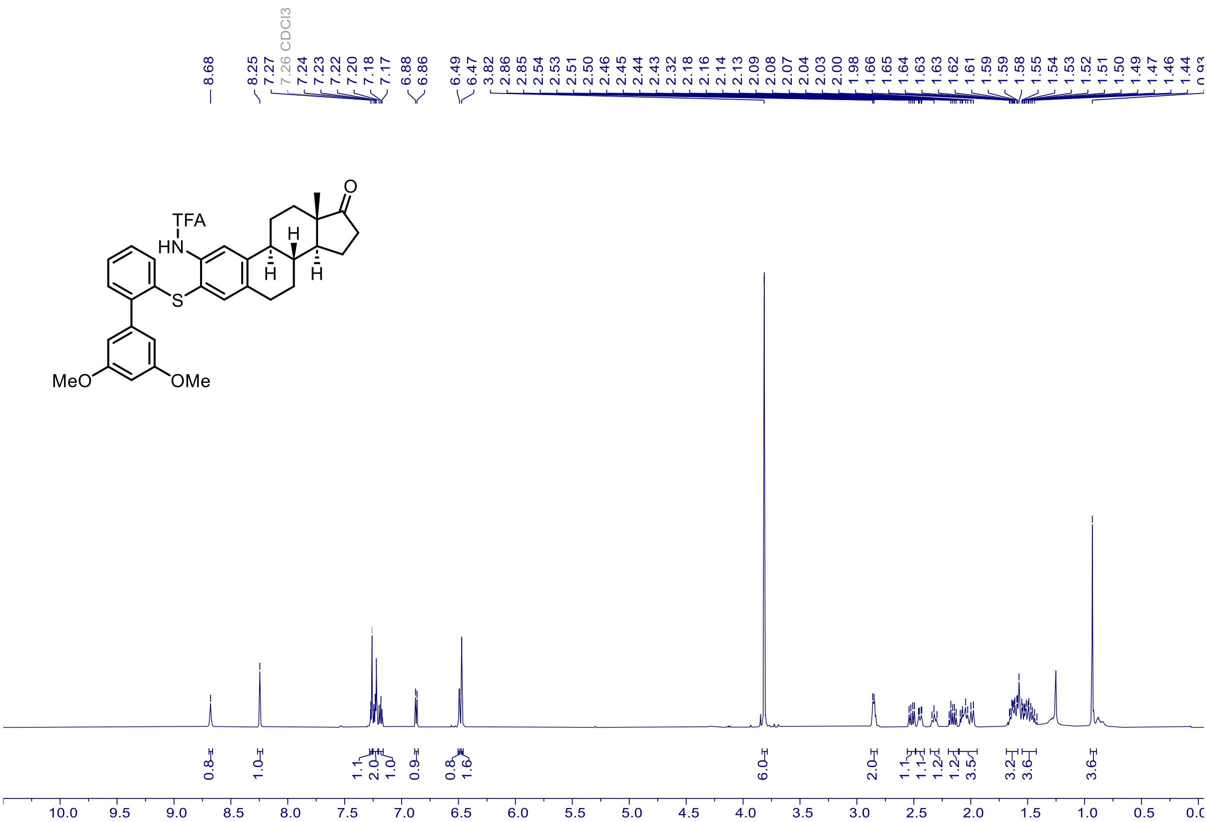

3yh – ^{13}C NMR (151 MHz, CDCl$_3$)

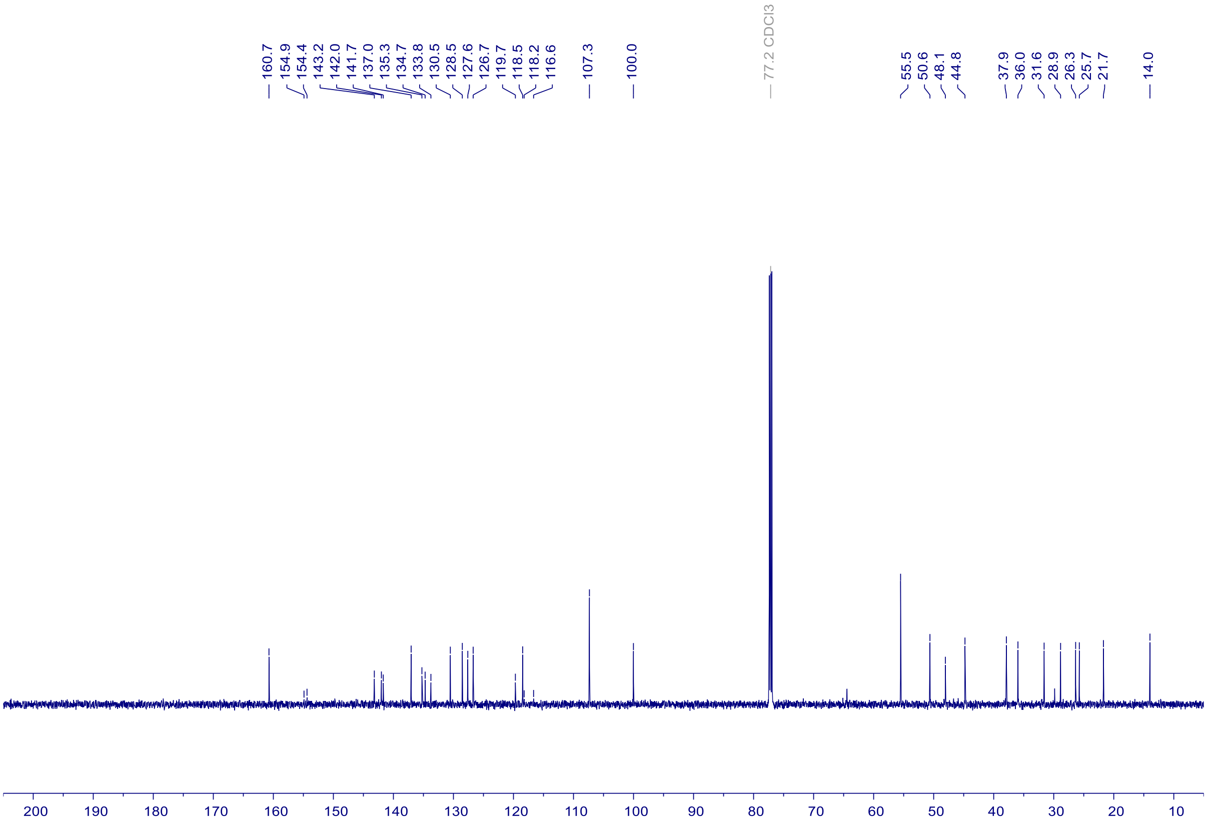

3yh – ^{19}F NMR (565 MHz, $CDCl_3$)

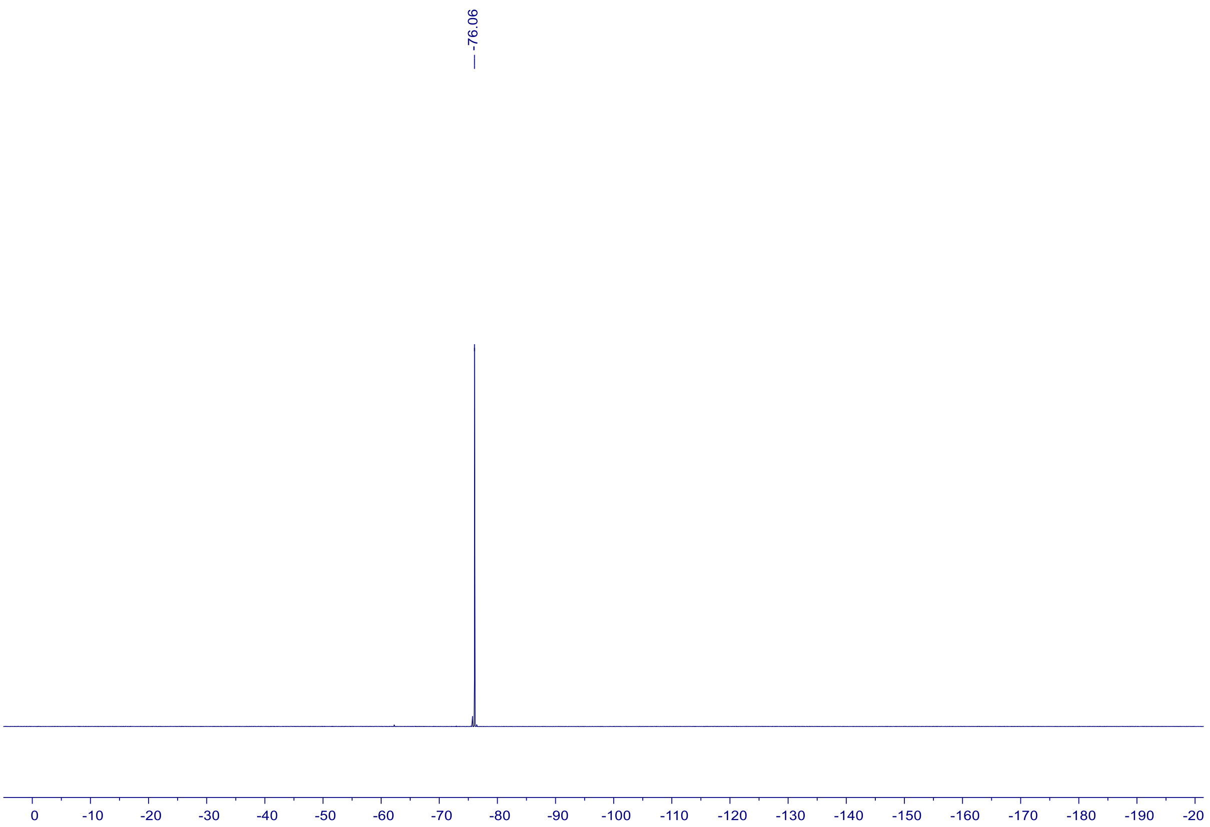

3yh´ – ^{1}H NMR (600 MHz, $CDCl_3$)

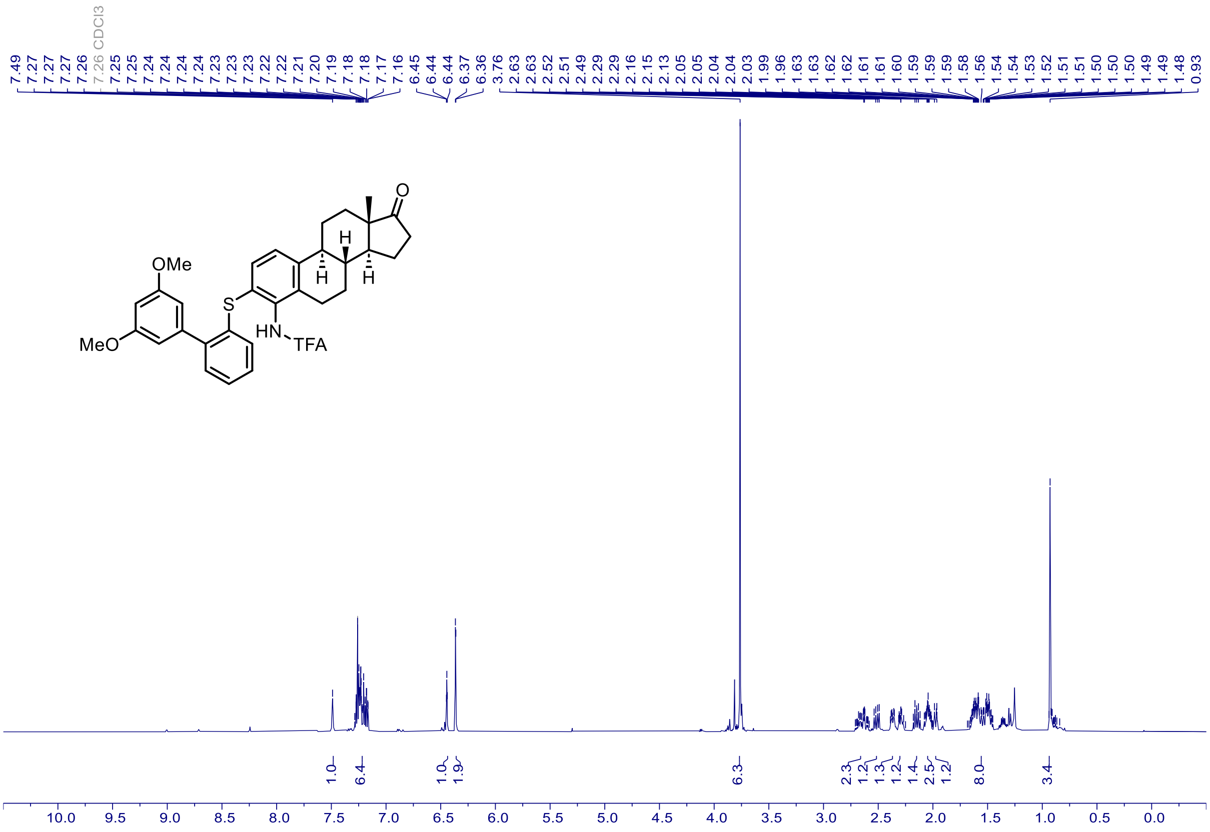

3yh′ – ^{13}C NMR (151 MHz, $CDCl_3$)

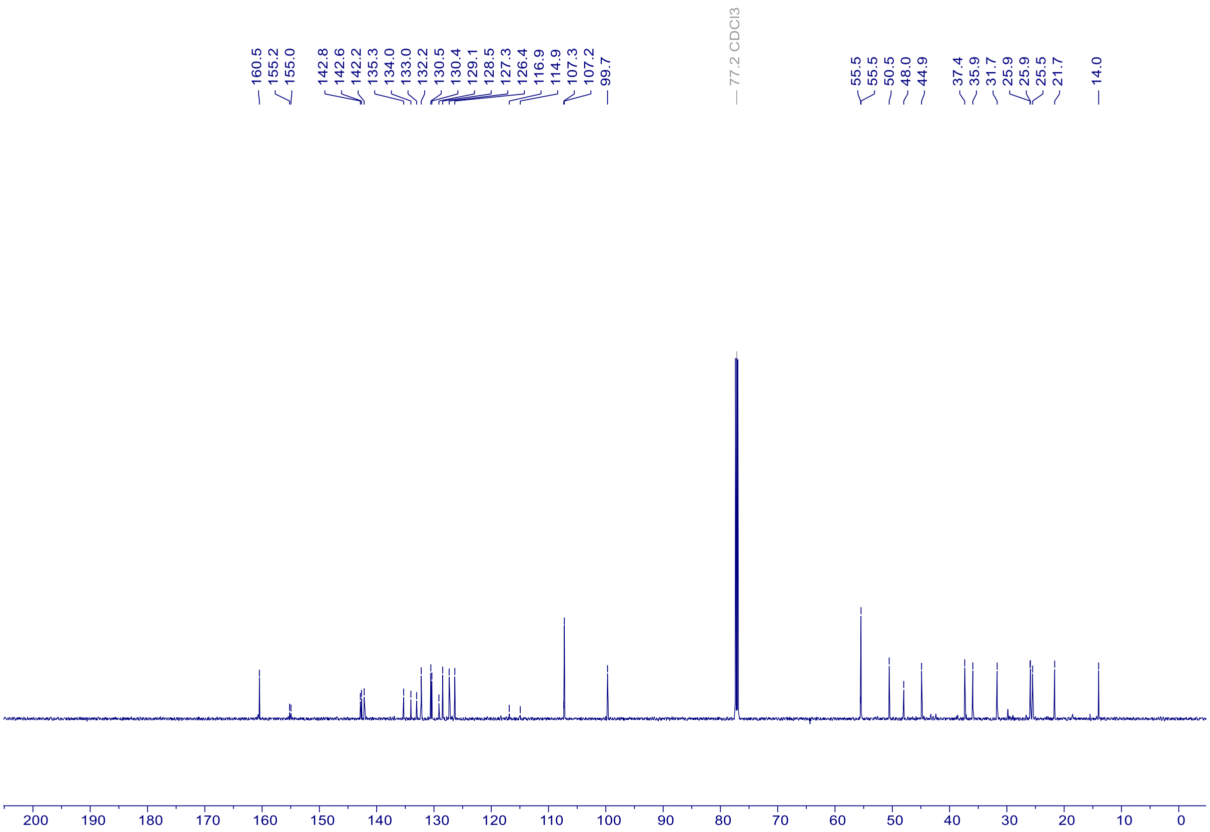

3yh′ – ^{19}F NMR (565 MHz, $CDCl_3$)

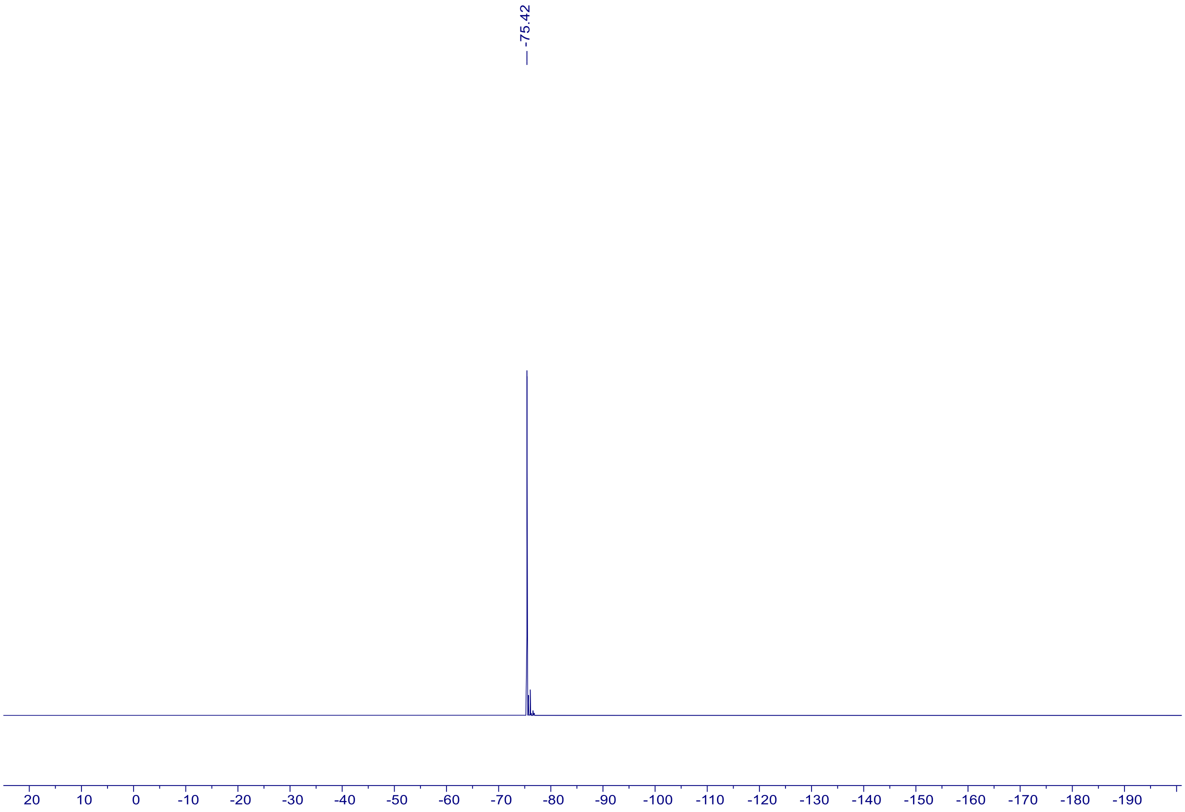

5b – ^{1}H NMR (600 MHz, $CDCl_3$)

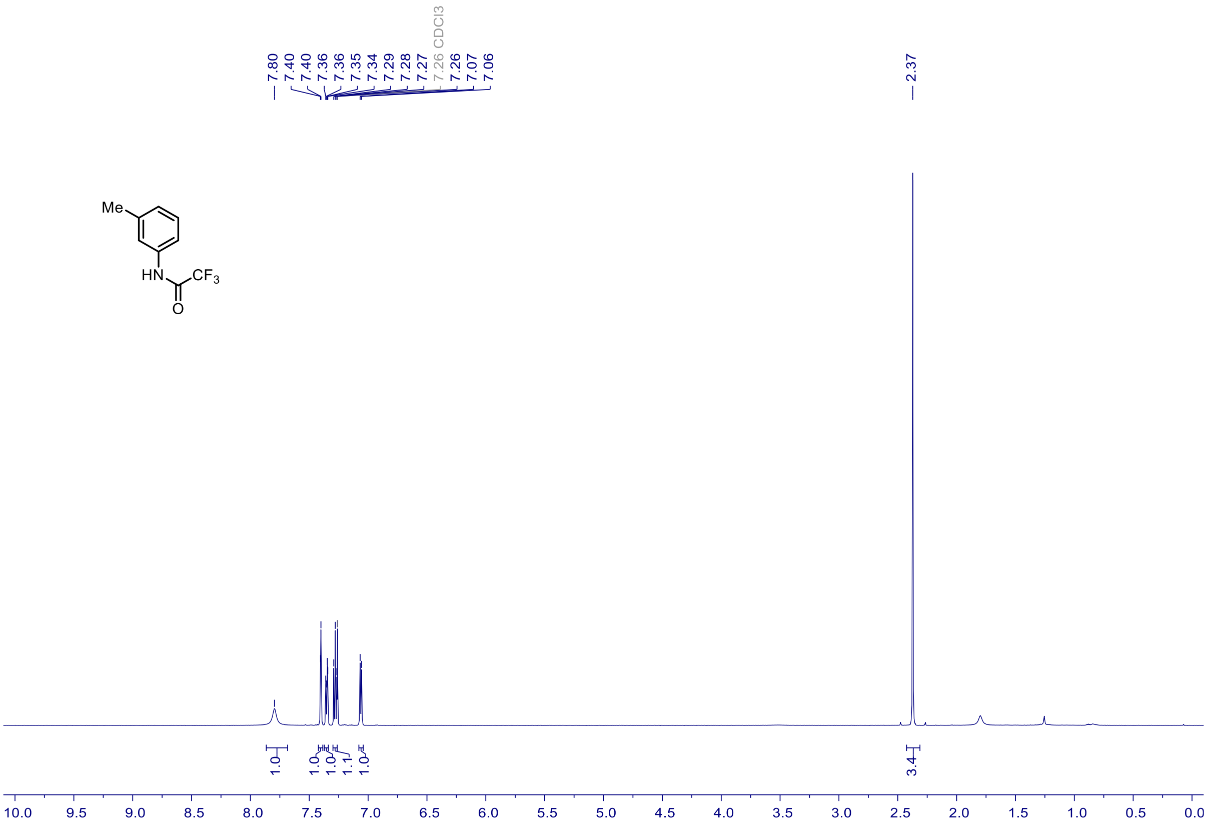

5b – ^{13}C NMR (151 MHz, $CDCl_3$)

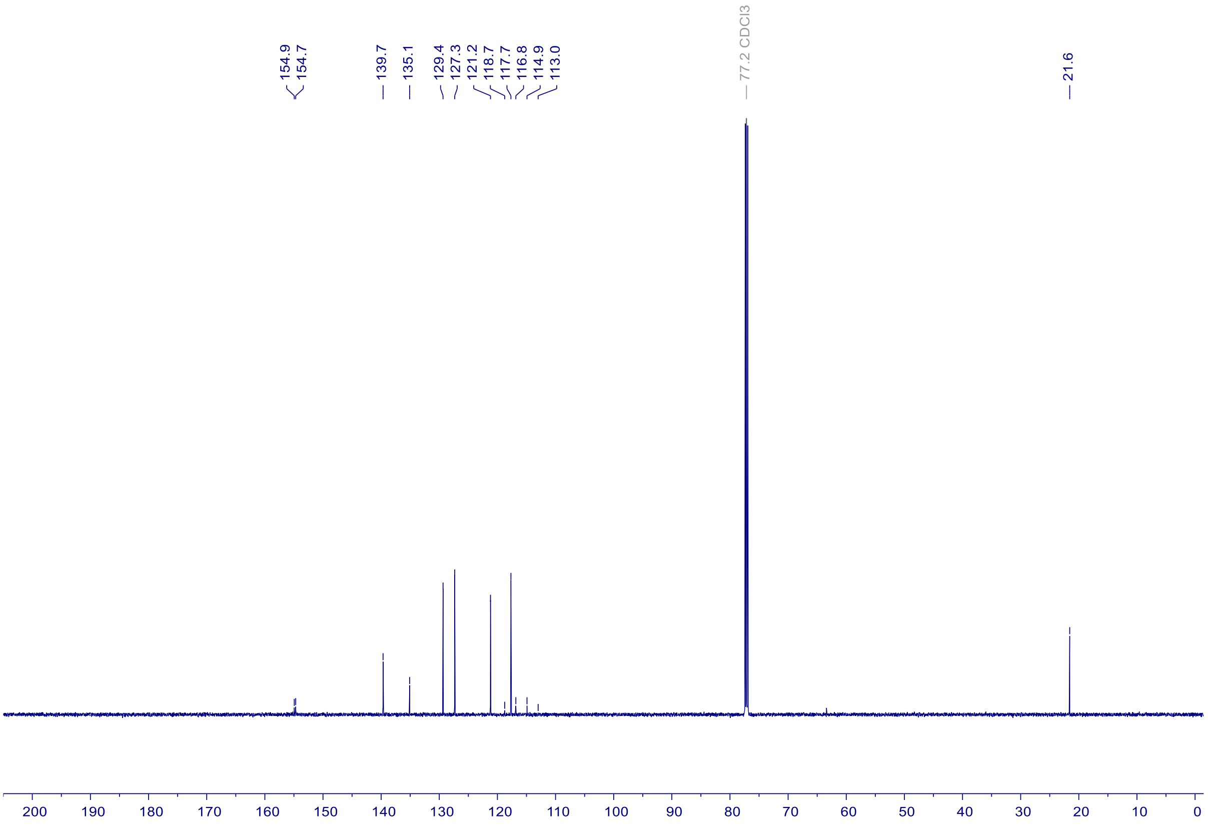

5b – ^{19}F NMR (565 MHz, $CDCl_3$)

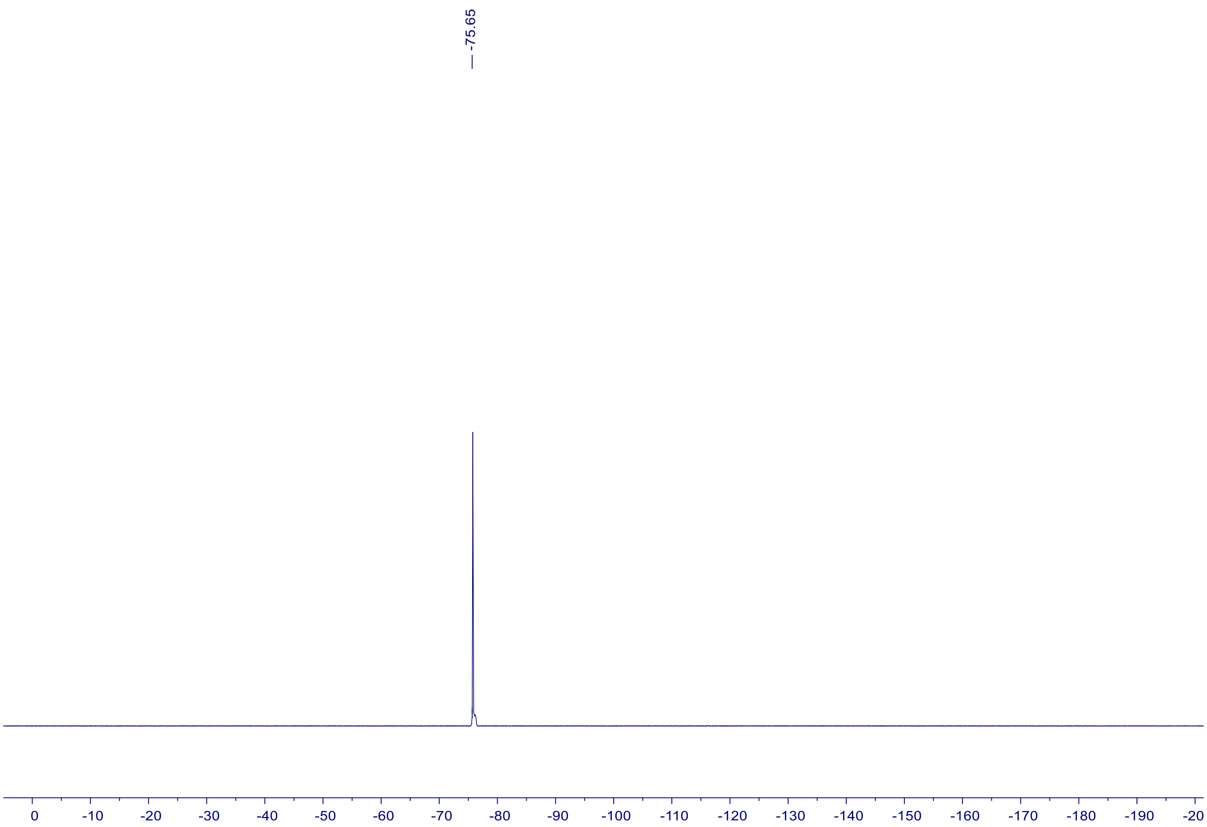

5c – ^{1}H NMR (600 MHz, $CDCl_3$)

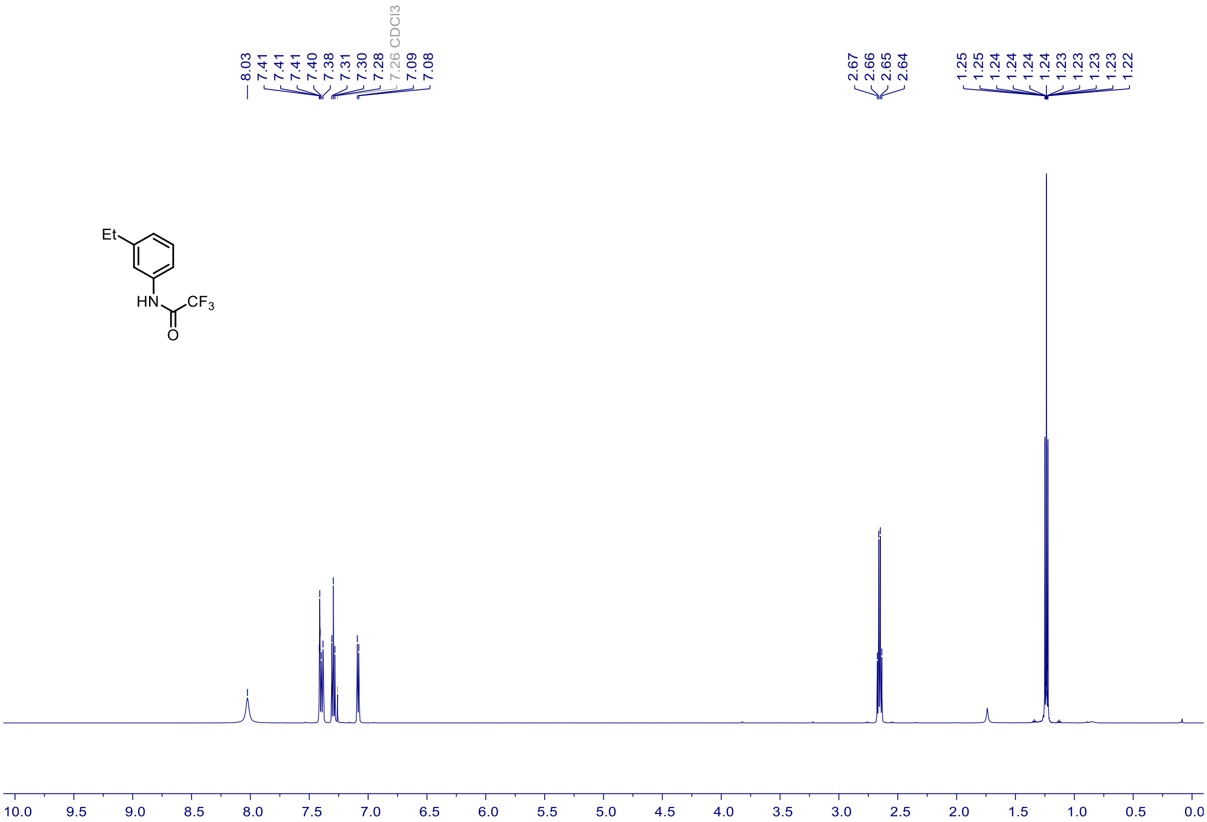

5c – ^{13}C NMR (151 MHz, $CDCl_3$)

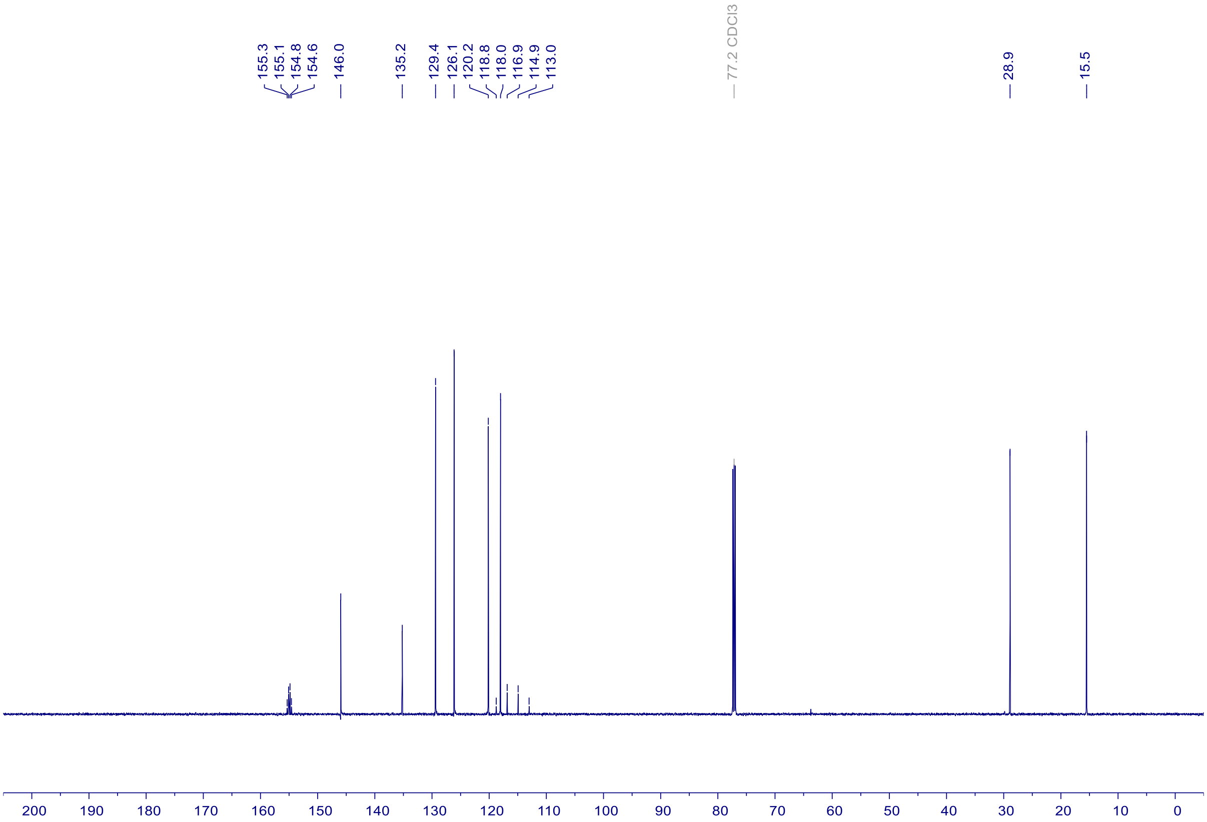

5c – ^{19}F NMR (565 MHz, $CDCl_3$)

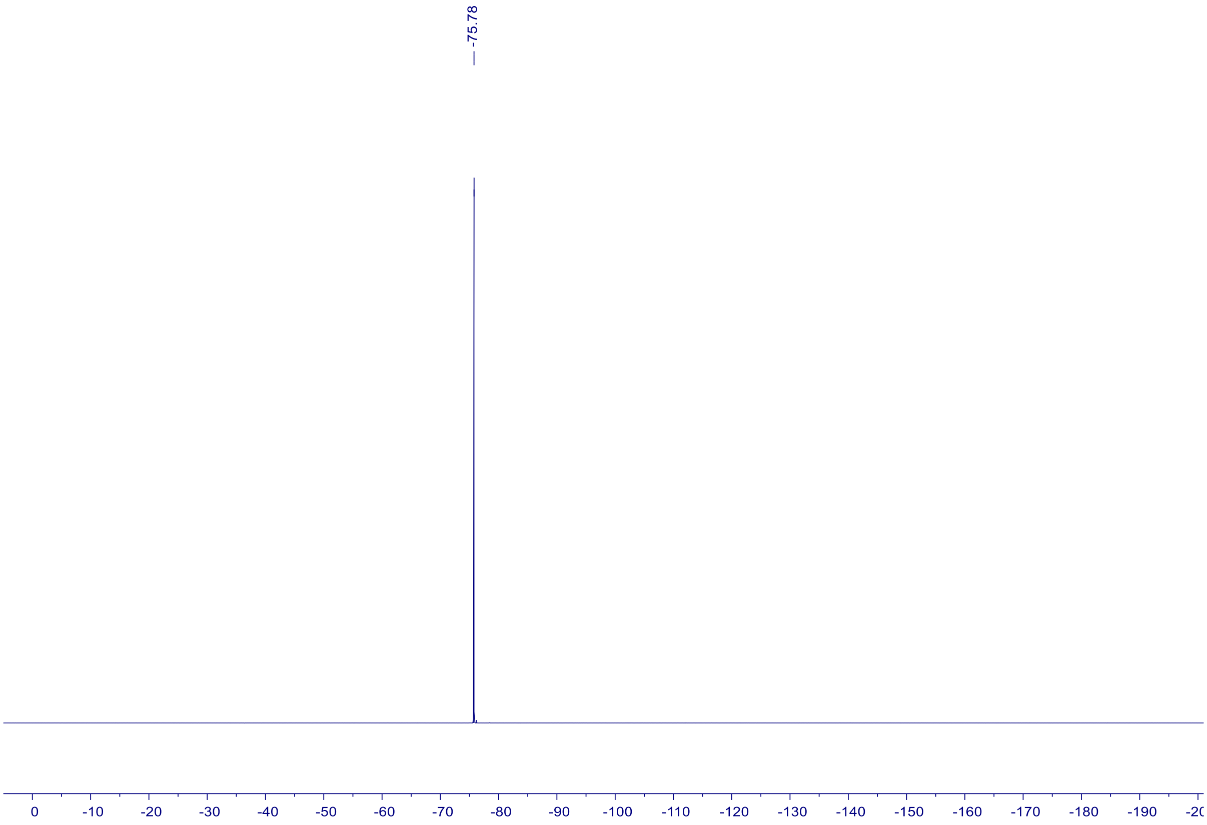

5d – ^{1}H NMR (600 MHz, $CDCl_3$)

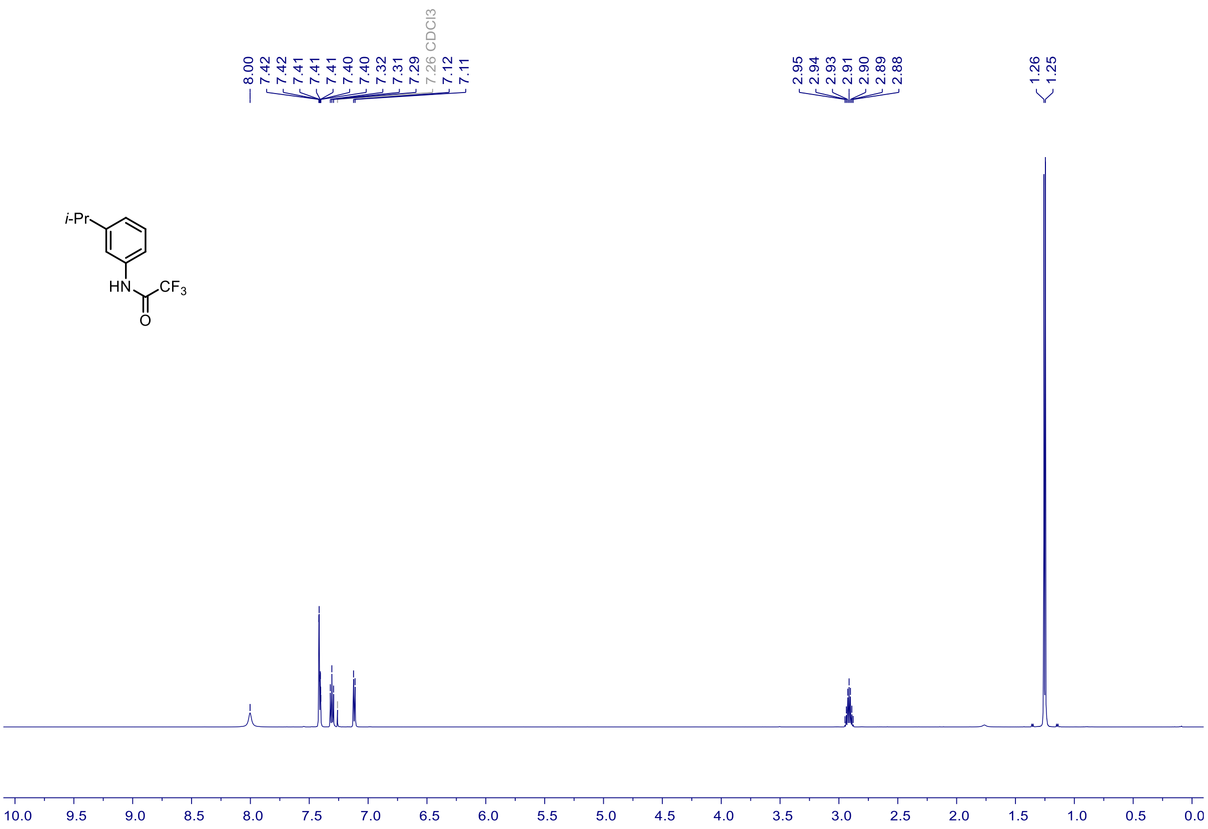

5d – ^{13}C NMR (151 MHz, $CDCl_3$)

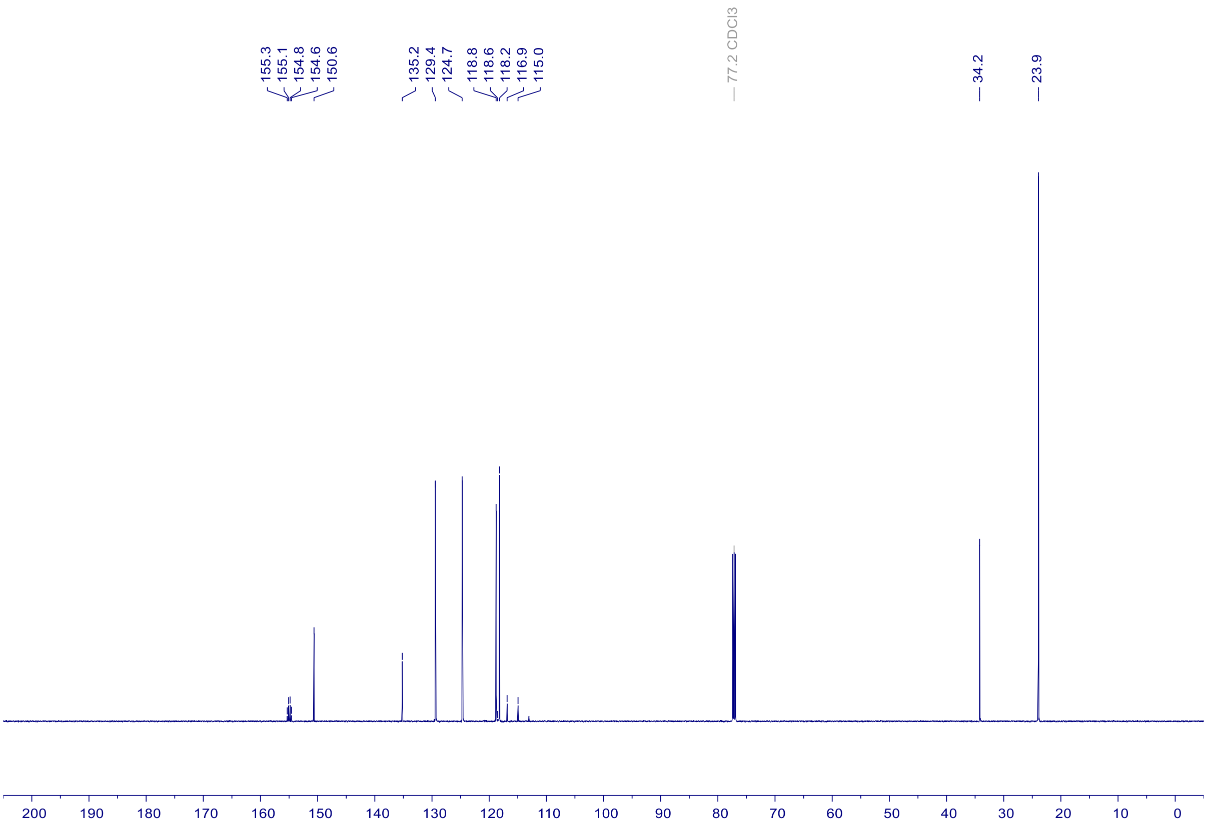

5d – ^{19}F NMR (565 MHz, $CDCl_3$)

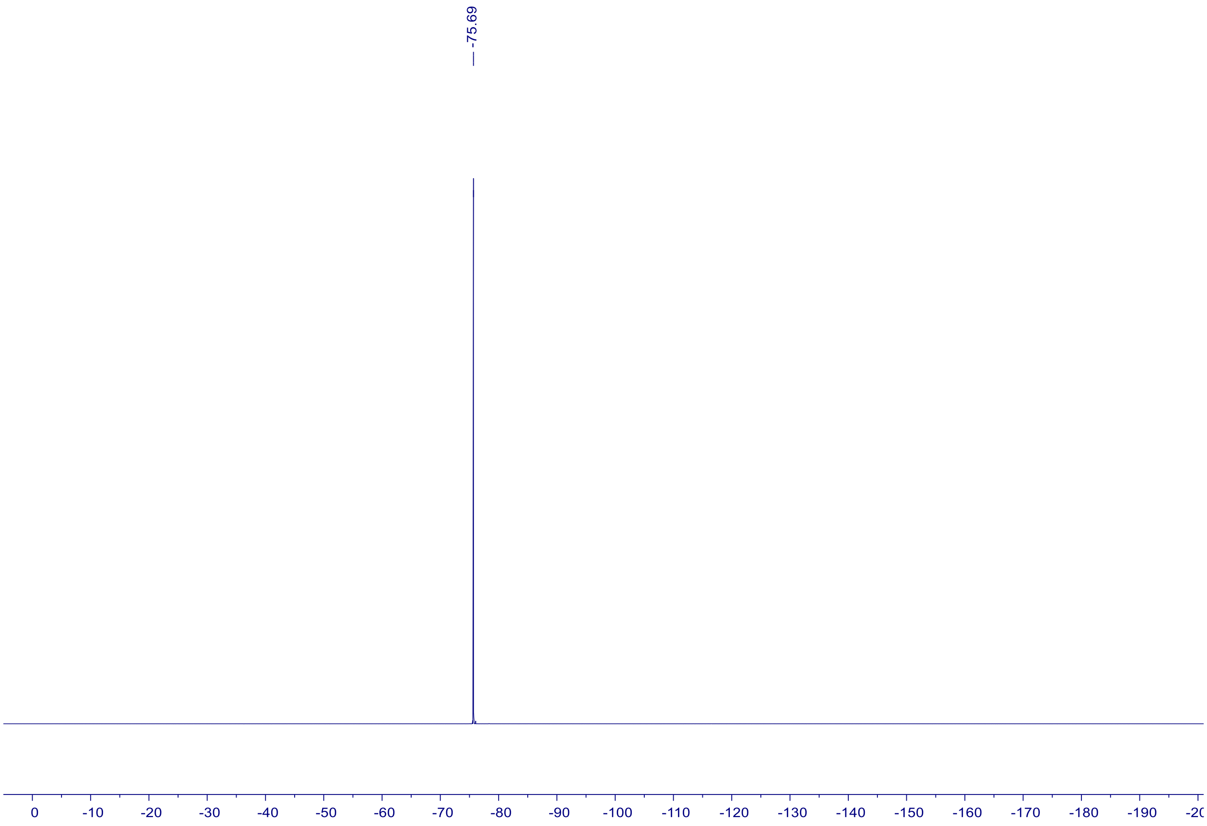

5e – ^{1}H NMR (600 MHz, $CDCl_3$)

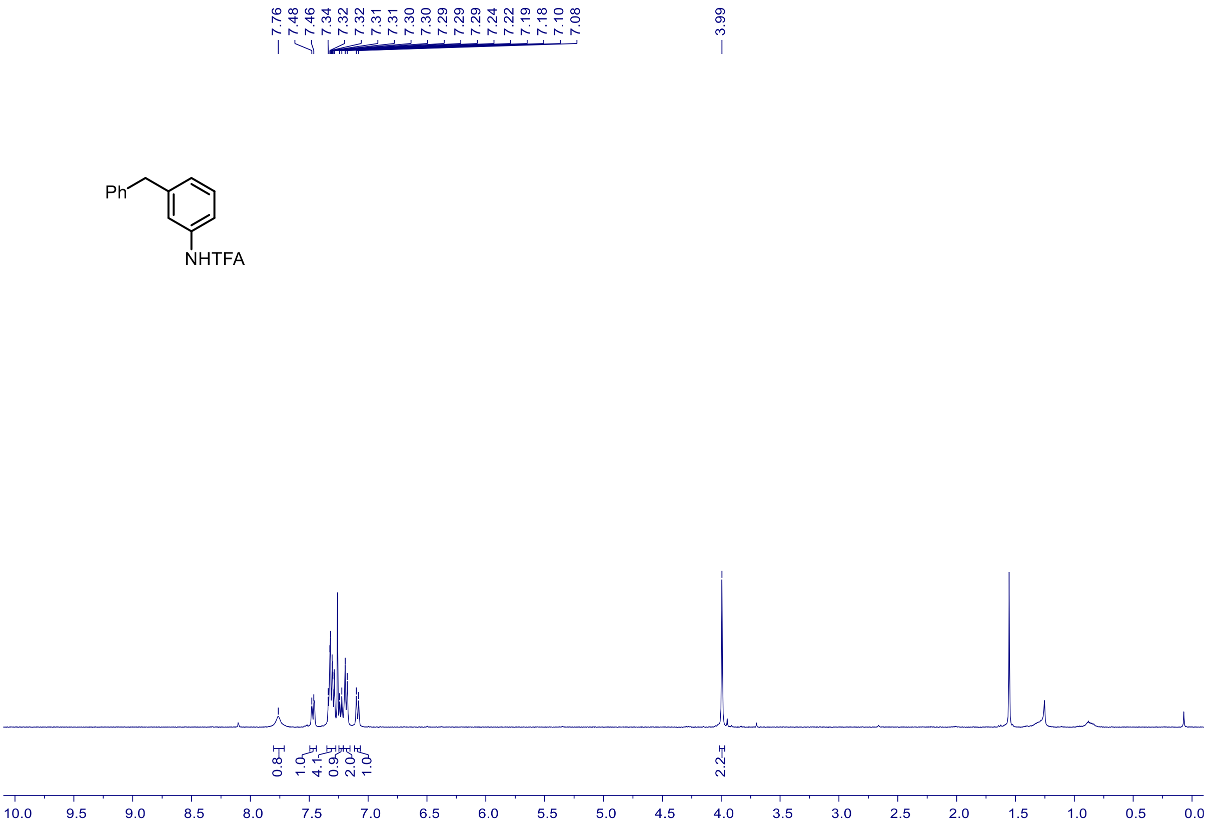

5e – ^{13}C NMR (151 MHz, $CDCl_3$)

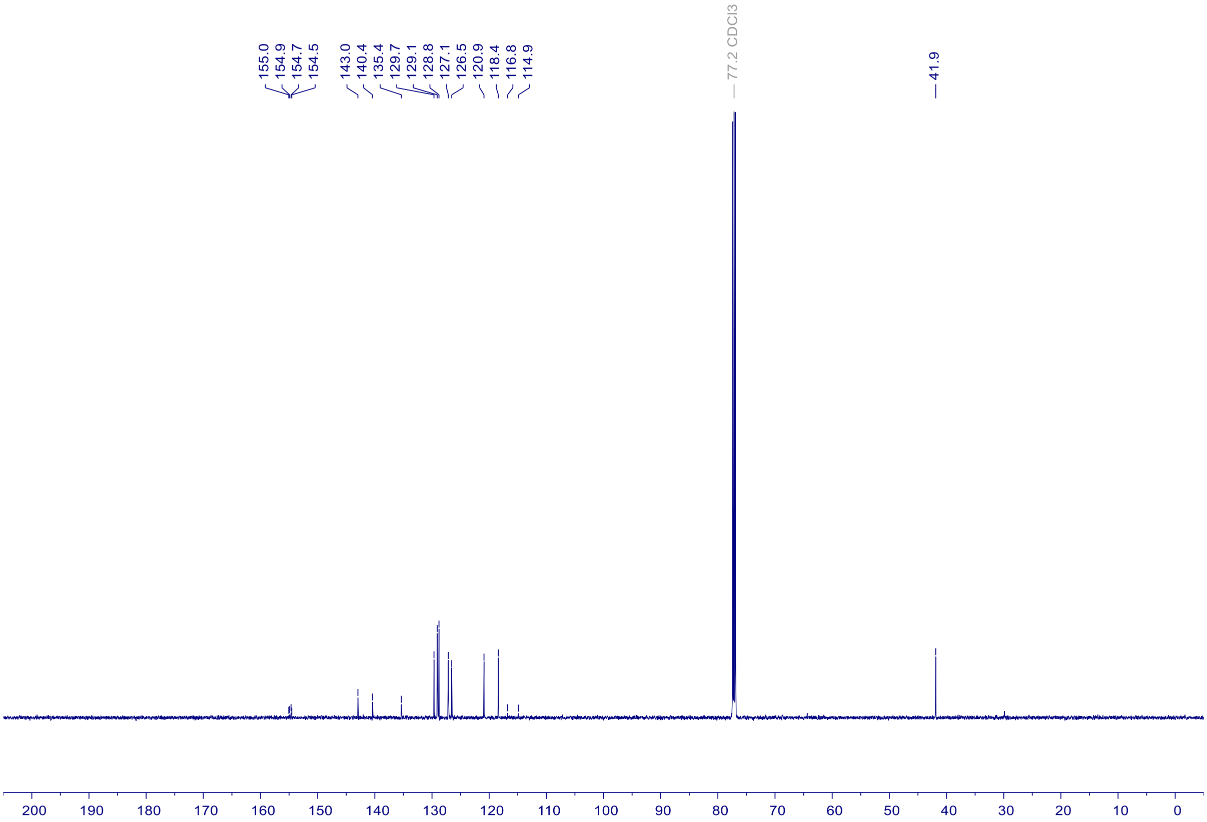

5e – ^{19}F NMR (565 MHz, $CDCl_3$)

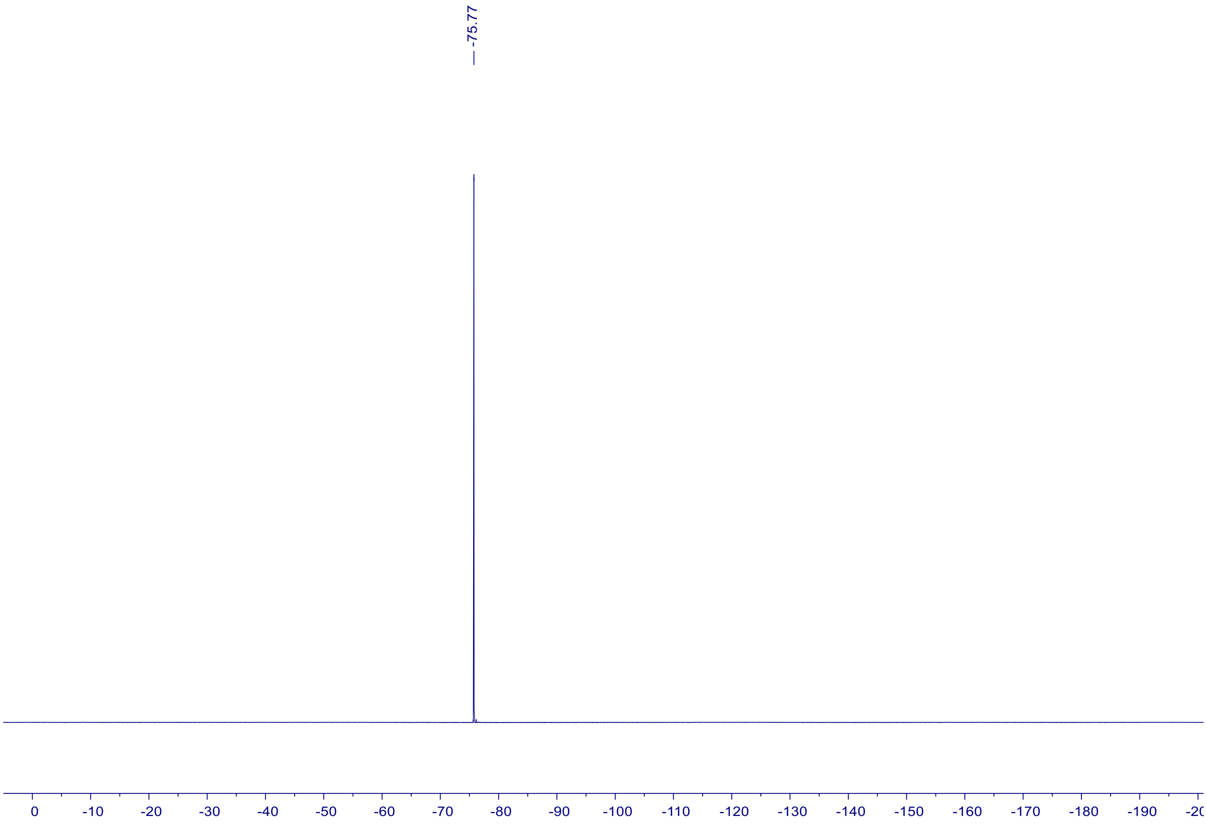

5f – ^{1}H NMR (600 MHz, $CDCl_3$)

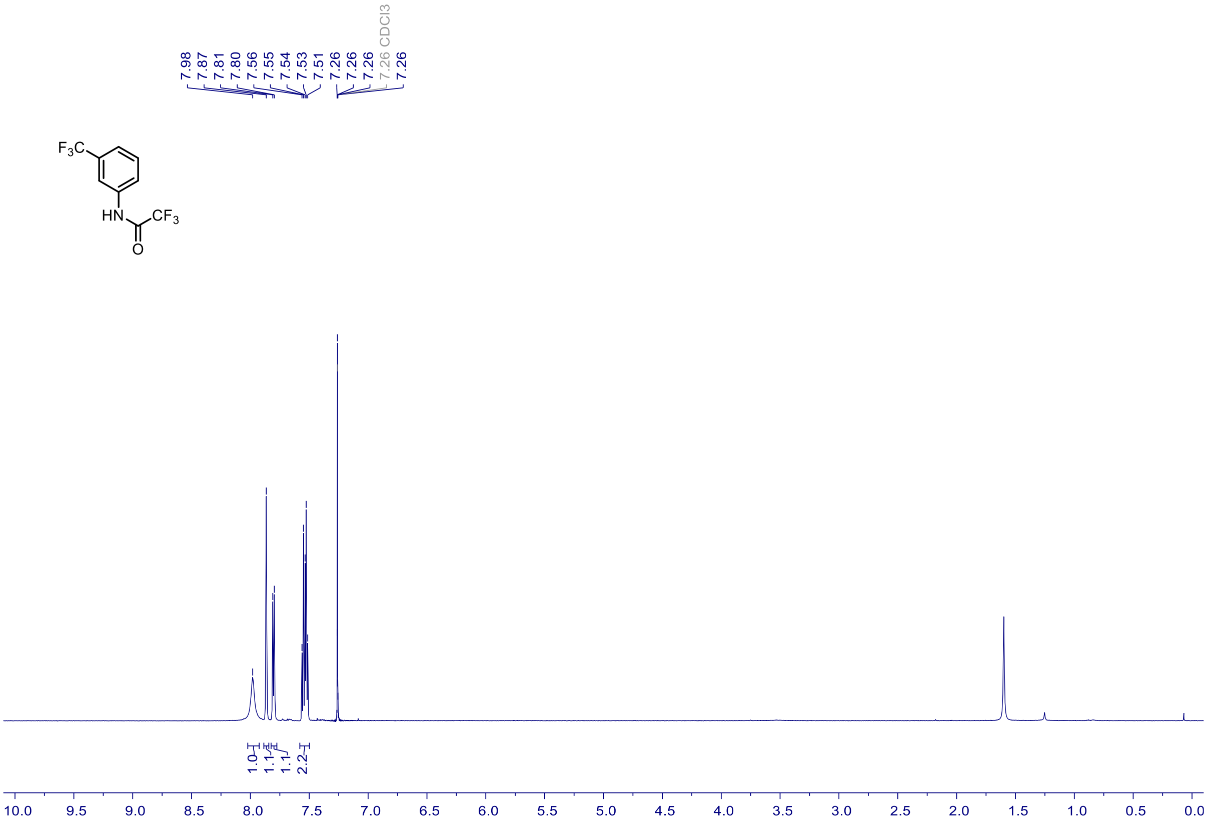

5f – ^{13}C NMR (151 MHz, $CDCl_3$)

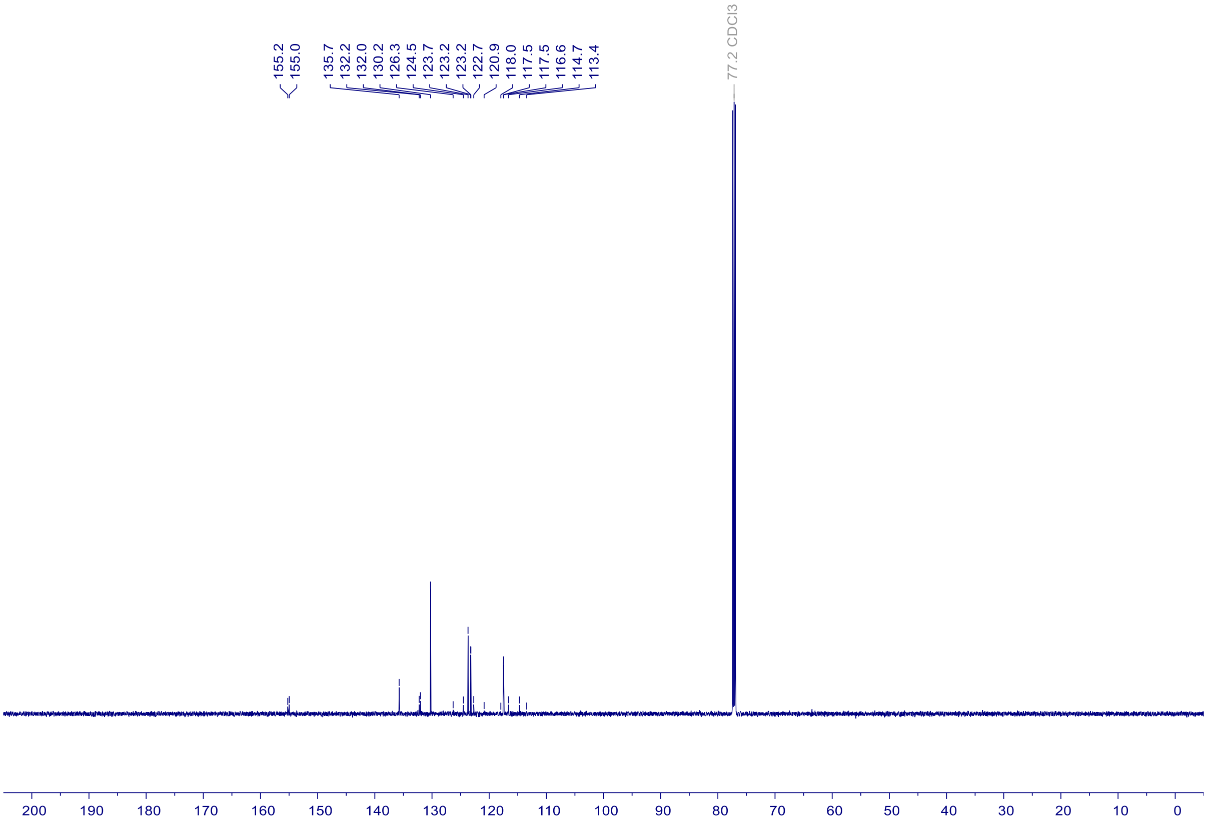

5f – ^{19}F NMR (565 MHz, $CDCl_3$)

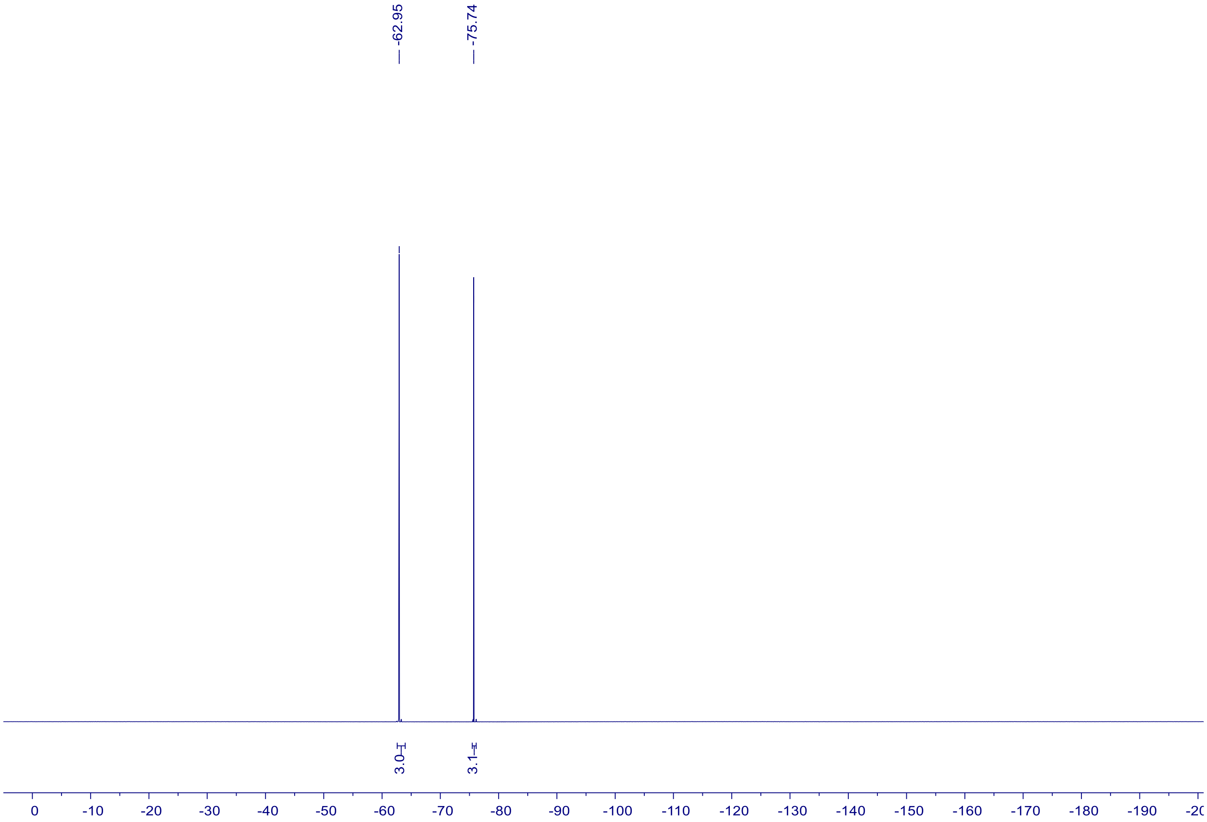

5g – ^{1}H NMR (600 MHz, $CDCl_3$)

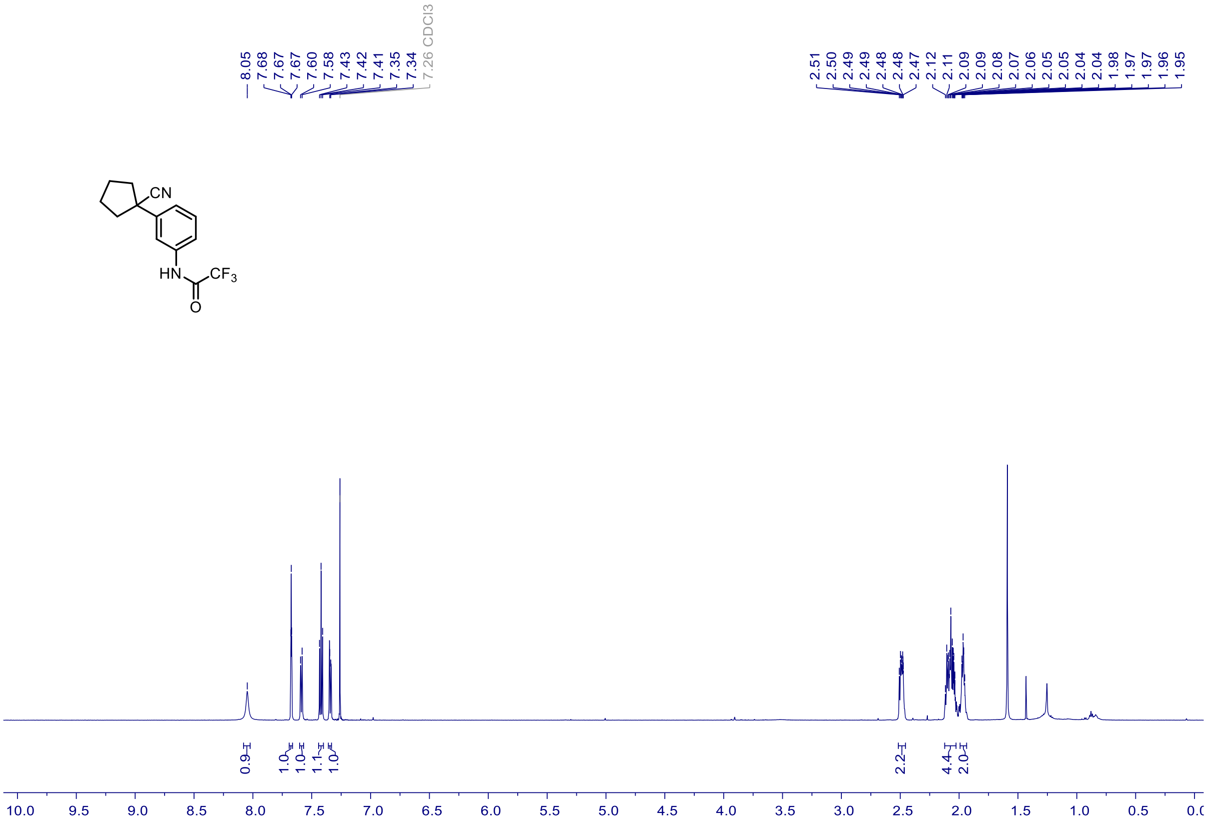

5g – ^{13}C NMR (151 MHz, $CDCl_3$)

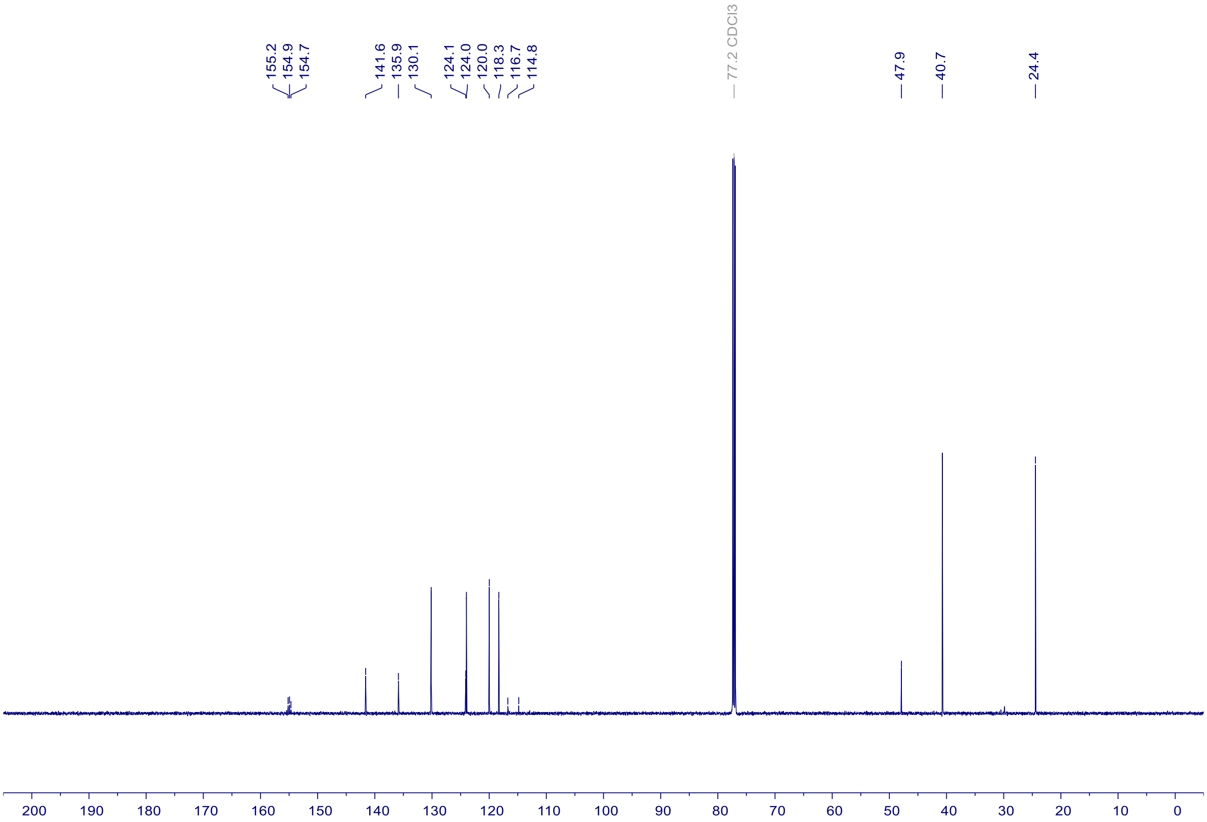

5g – ^{19}F NMR (565 MHz, $CDCl_3$)

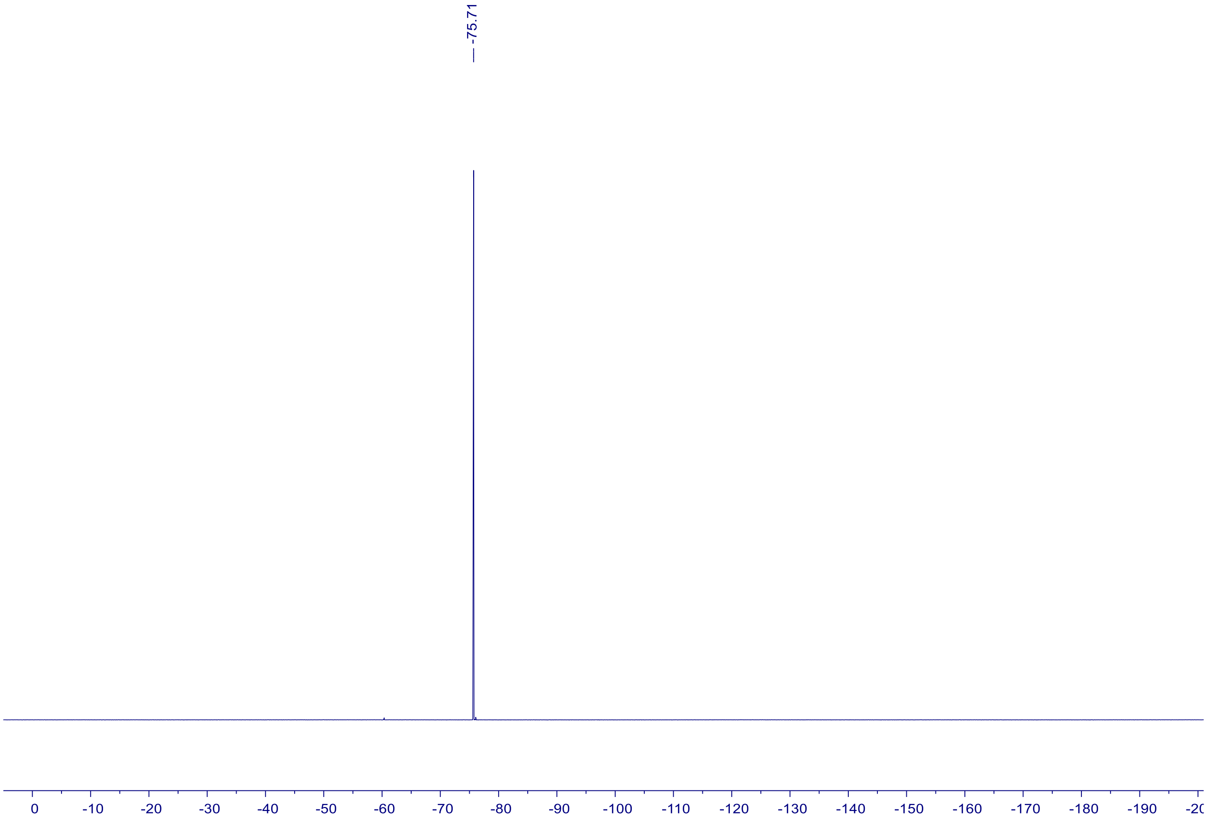

5h – ^{1}H NMR (600 MHz, $CDCl_3$)

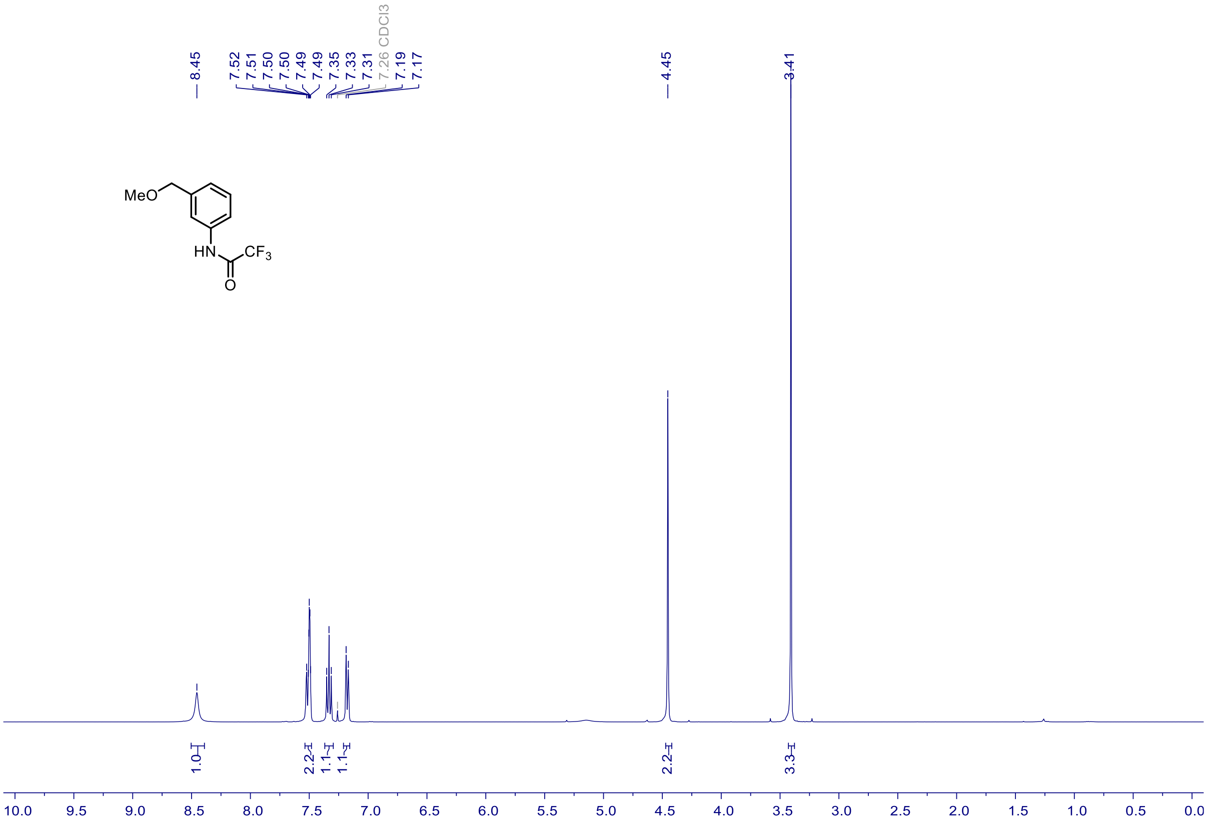

5h – ^{13}C NMR (151 MHz, $CDCl_3$)

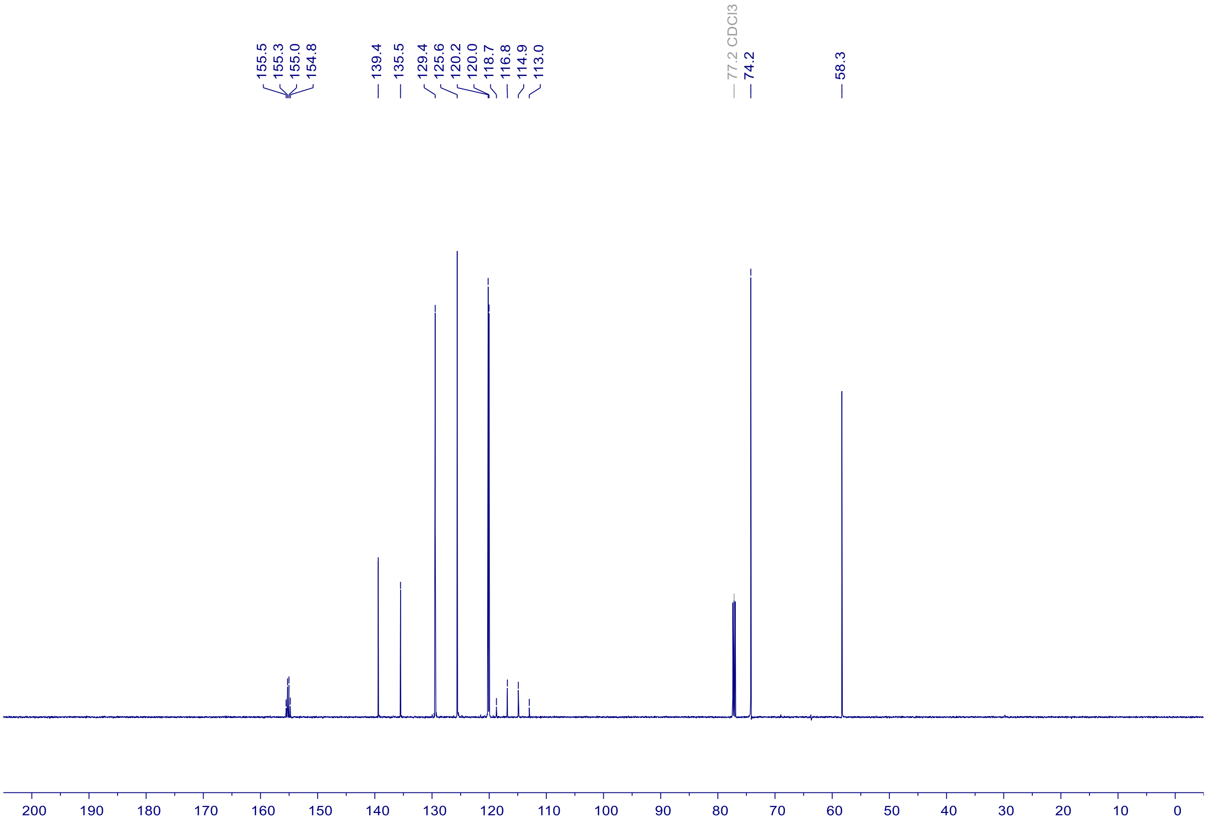

5h – ^{19}F NMR (565 MHz, $CDCl_3$)

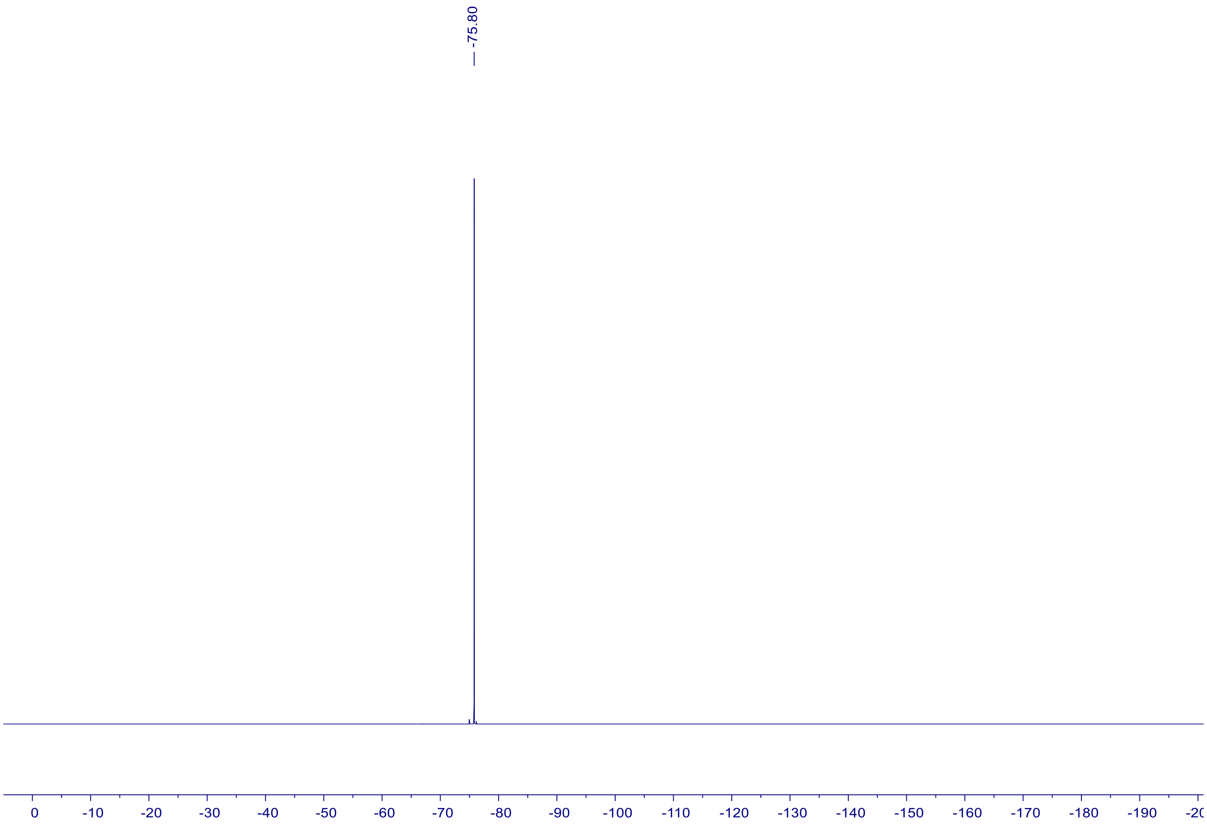

5i – ^{1}H NMR (600 MHz, $CDCl_3$)

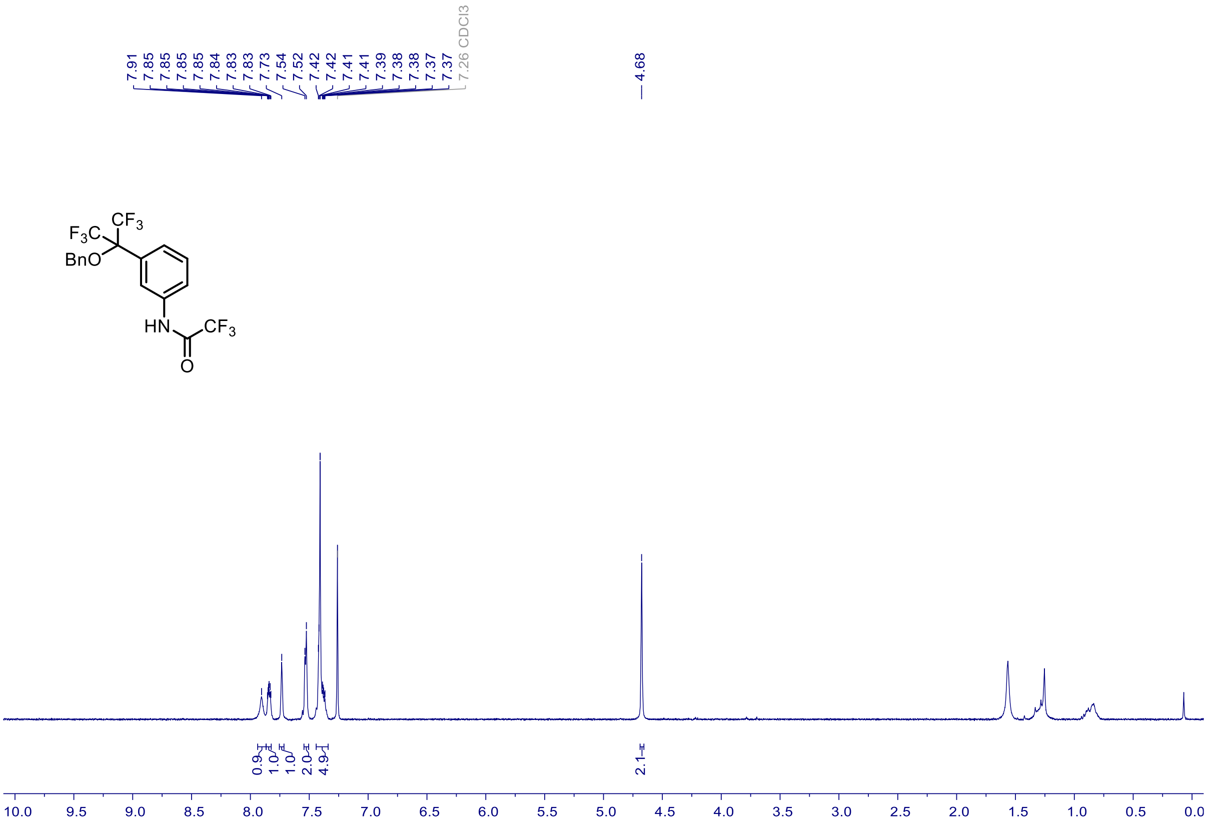

5i – ^{13}C NMR (151 MHz, $CDCl_3$)

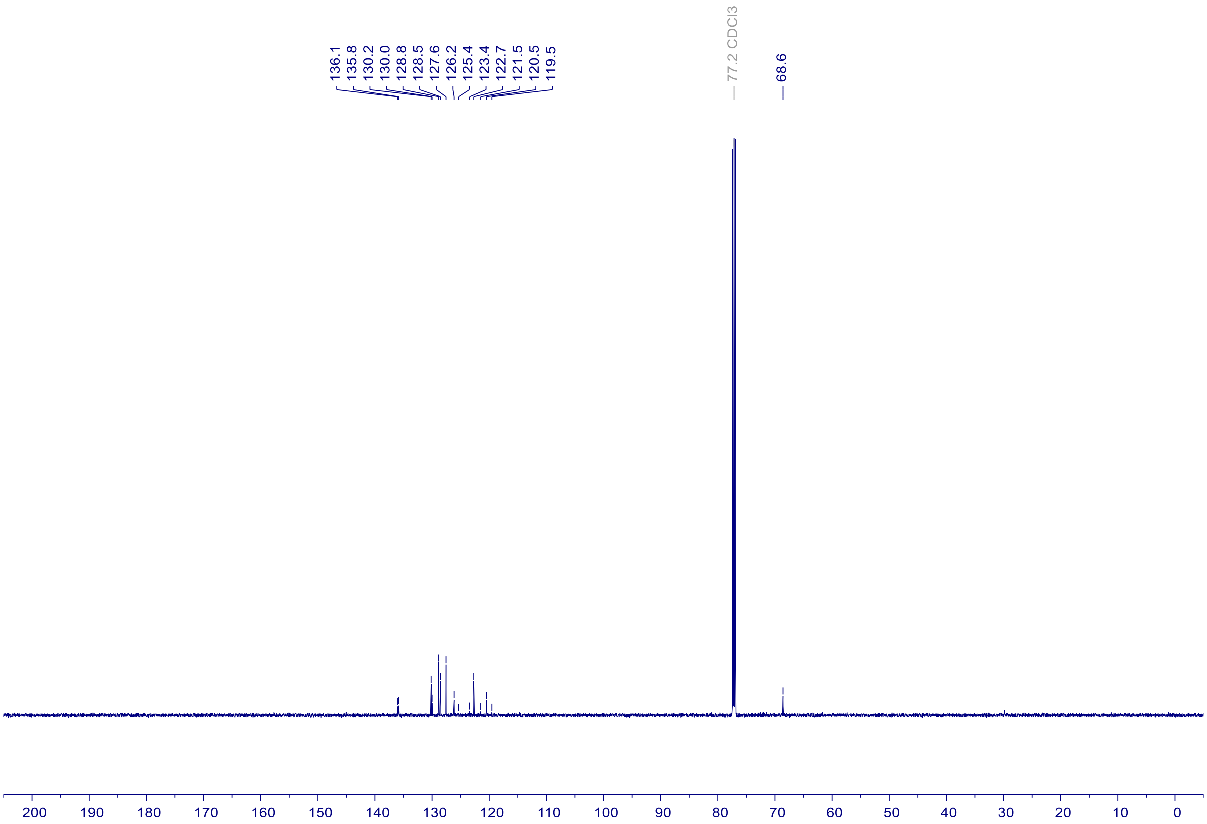

5i – ^{19}F NMR (565 MHz, $CDCl_3$)

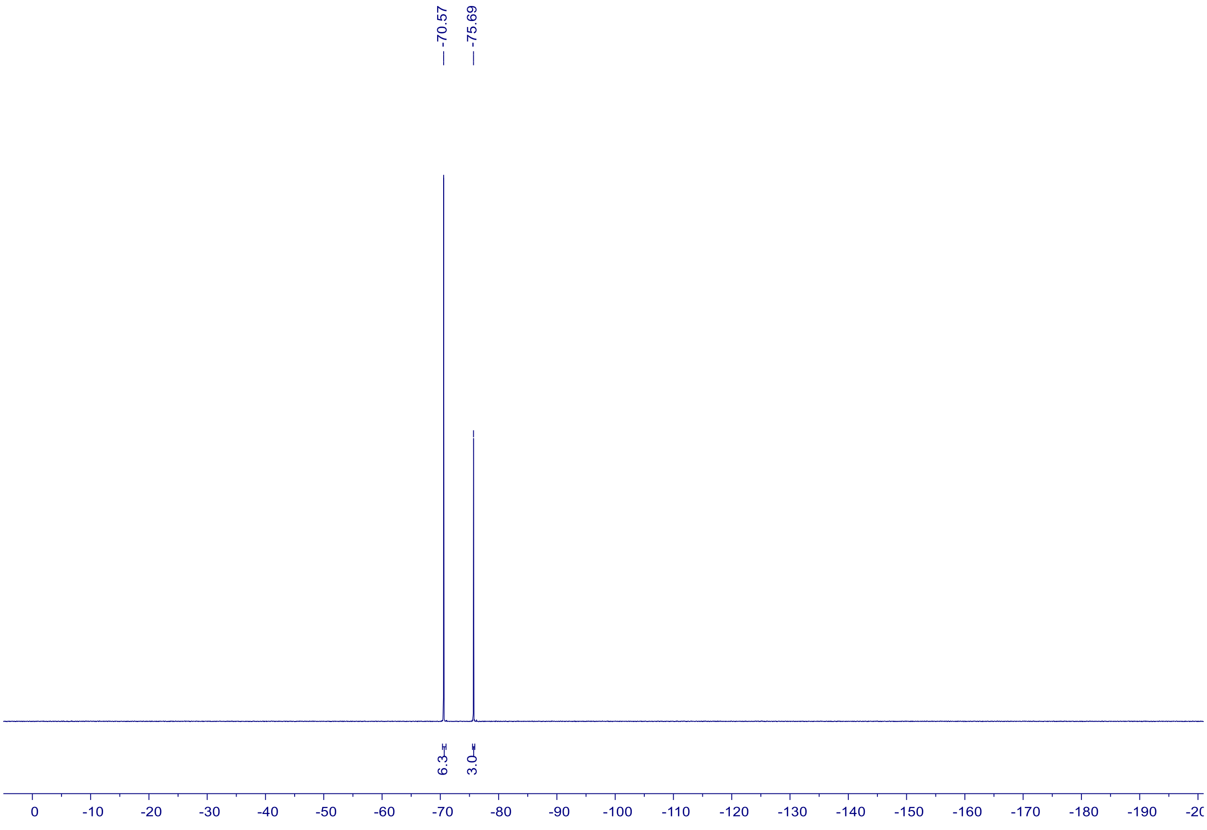

5j – ^{1}H NMR (600 MHz, $CDCl_3$)

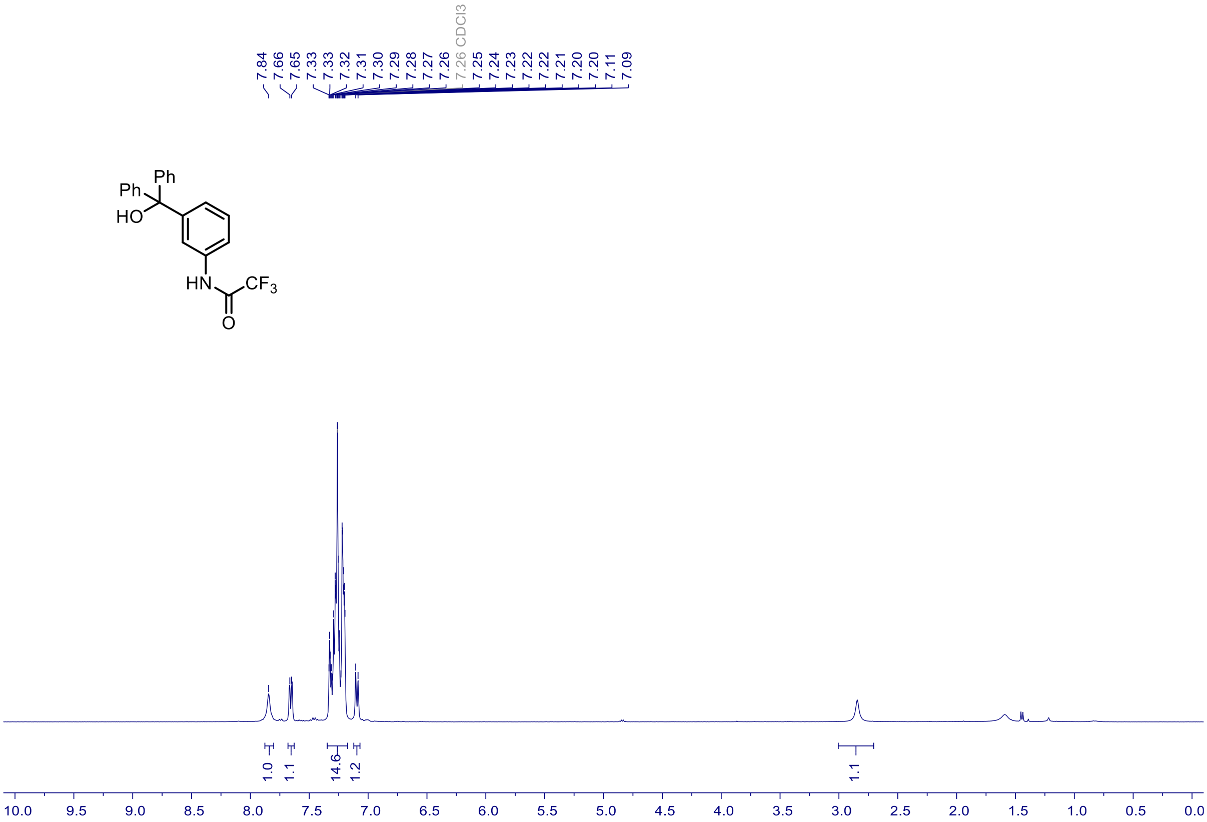

5j – ^{13}C NMR (151 MHz, $CDCl_3$)

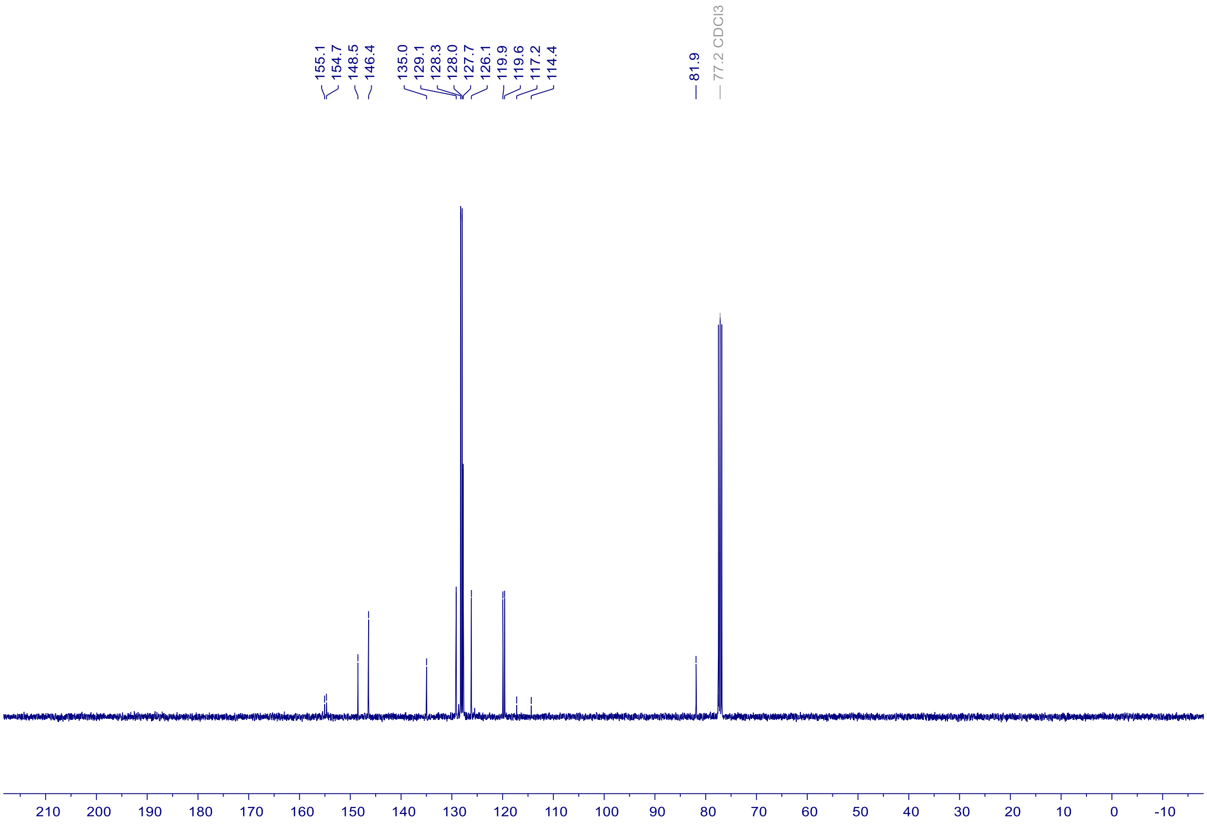

5j – ^{19}F NMR (565 MHz, $CDCl_3$)

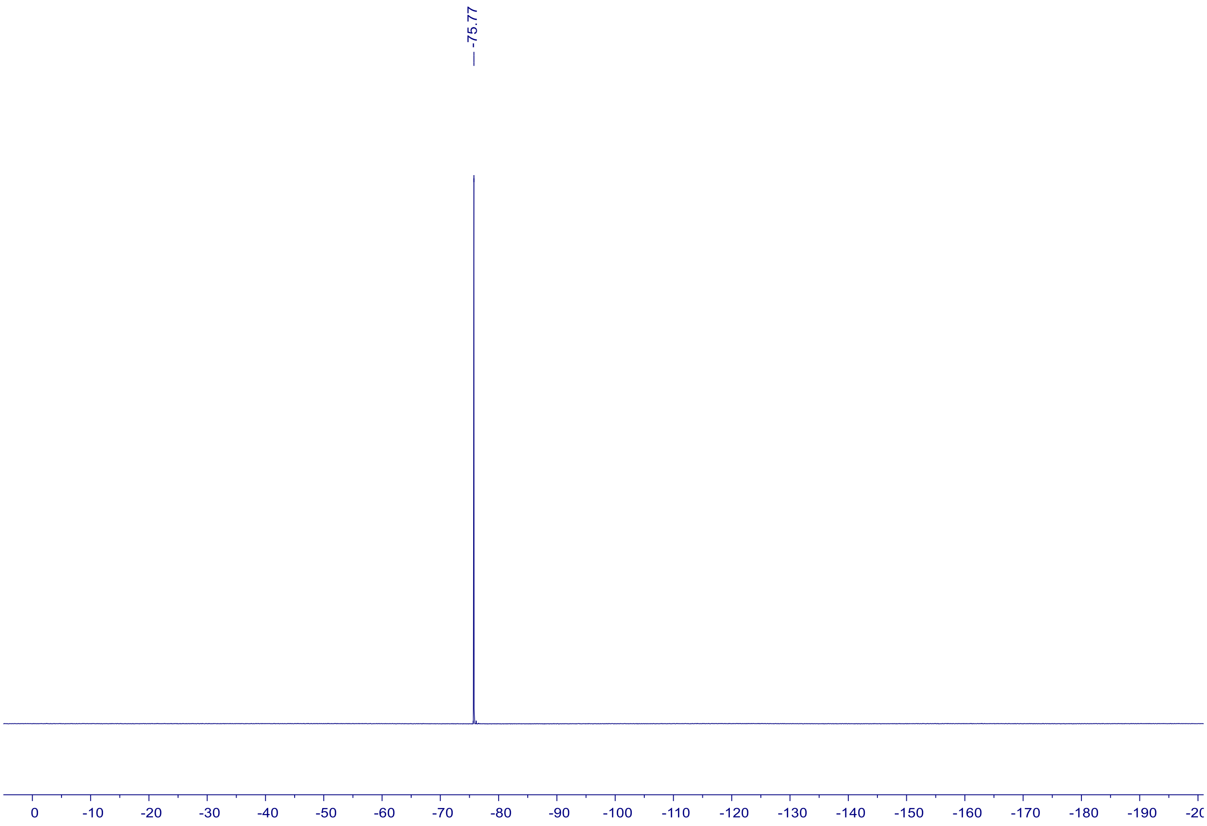

5k – ^{1}H NMR (600 MHz, $CDCl_3$)

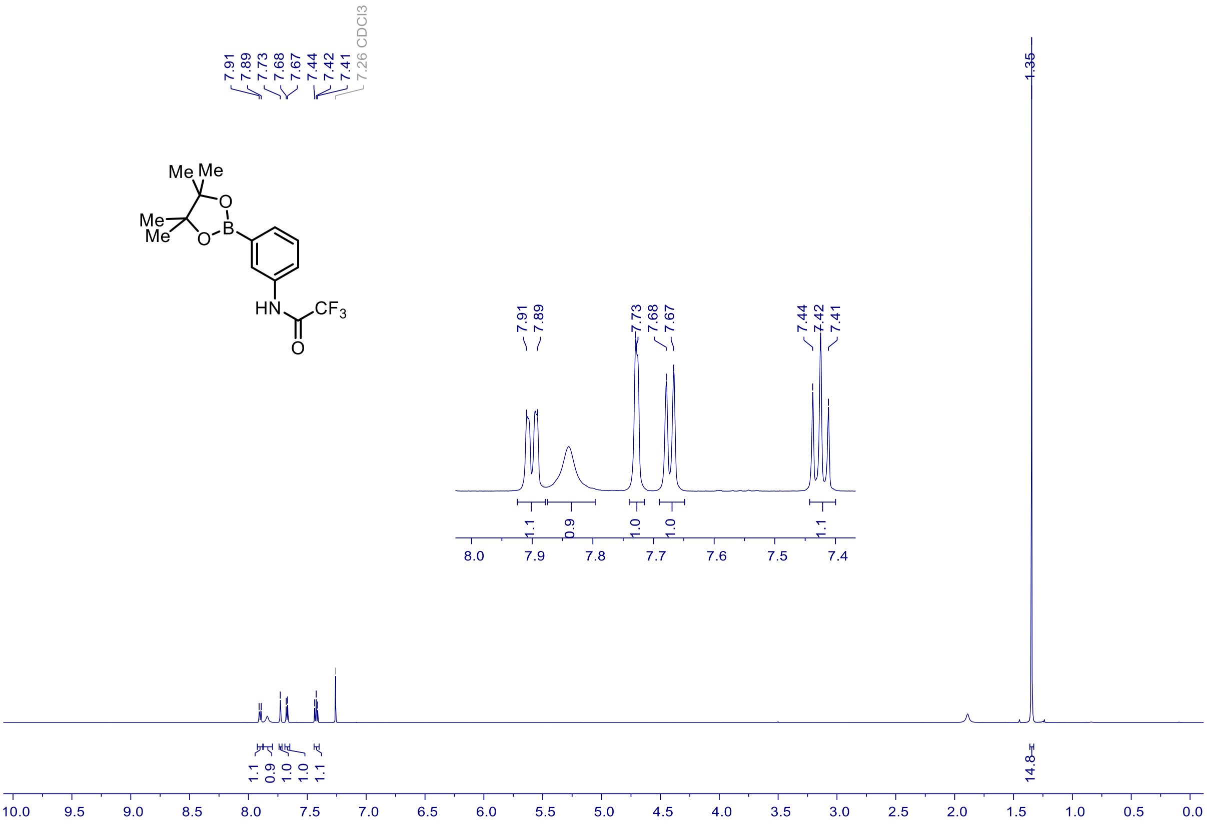

5k – ^{13}C NMR (151 MHz, $CDCl_3$)

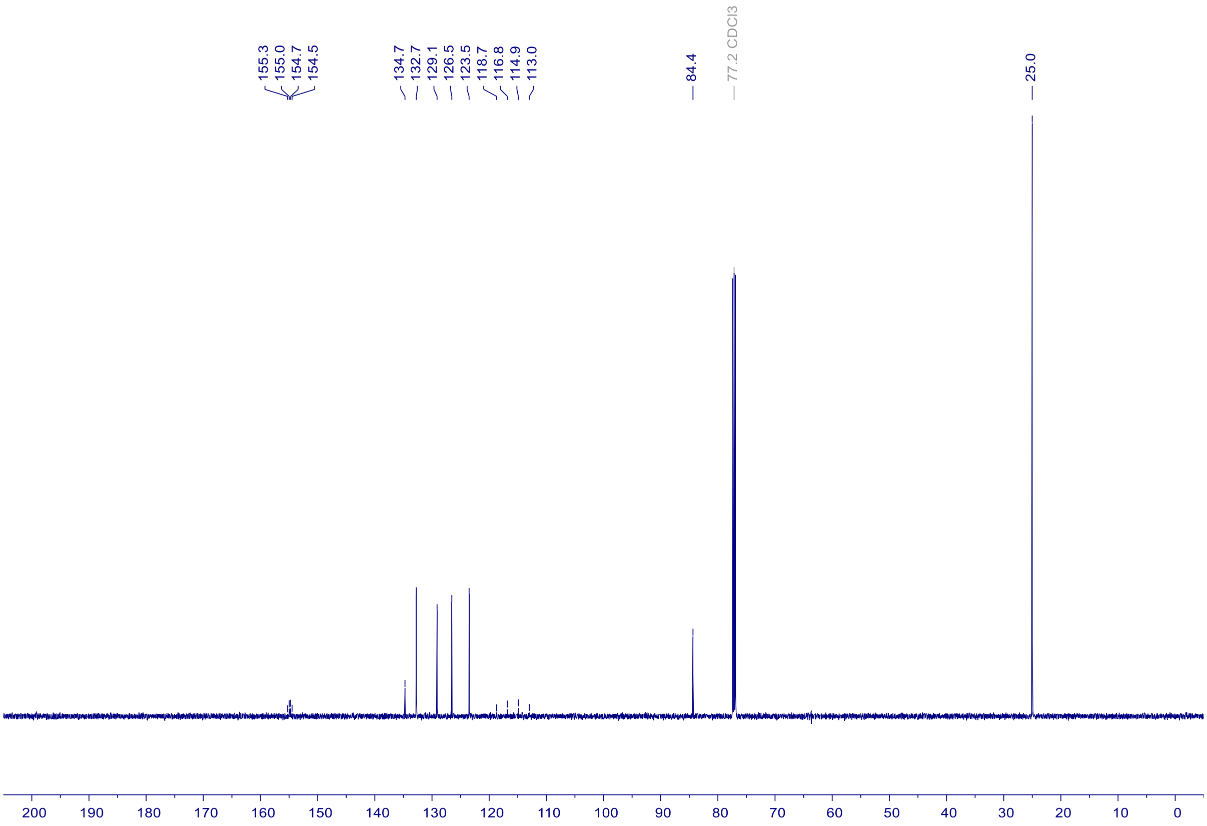

5k – ^{19}F NMR (565 MHz, $CDCl_3$)

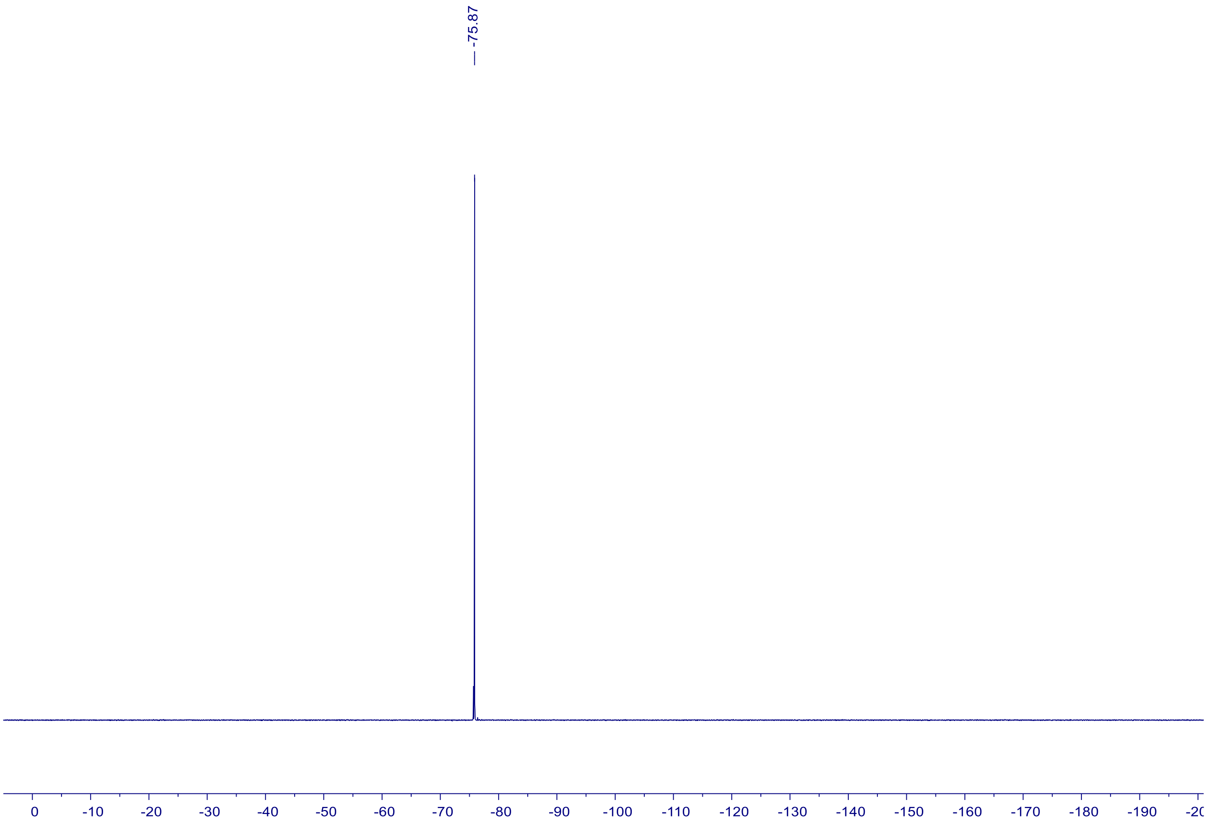

5l – ^{1}H NMR (600 MHz, $CDCl_3$)

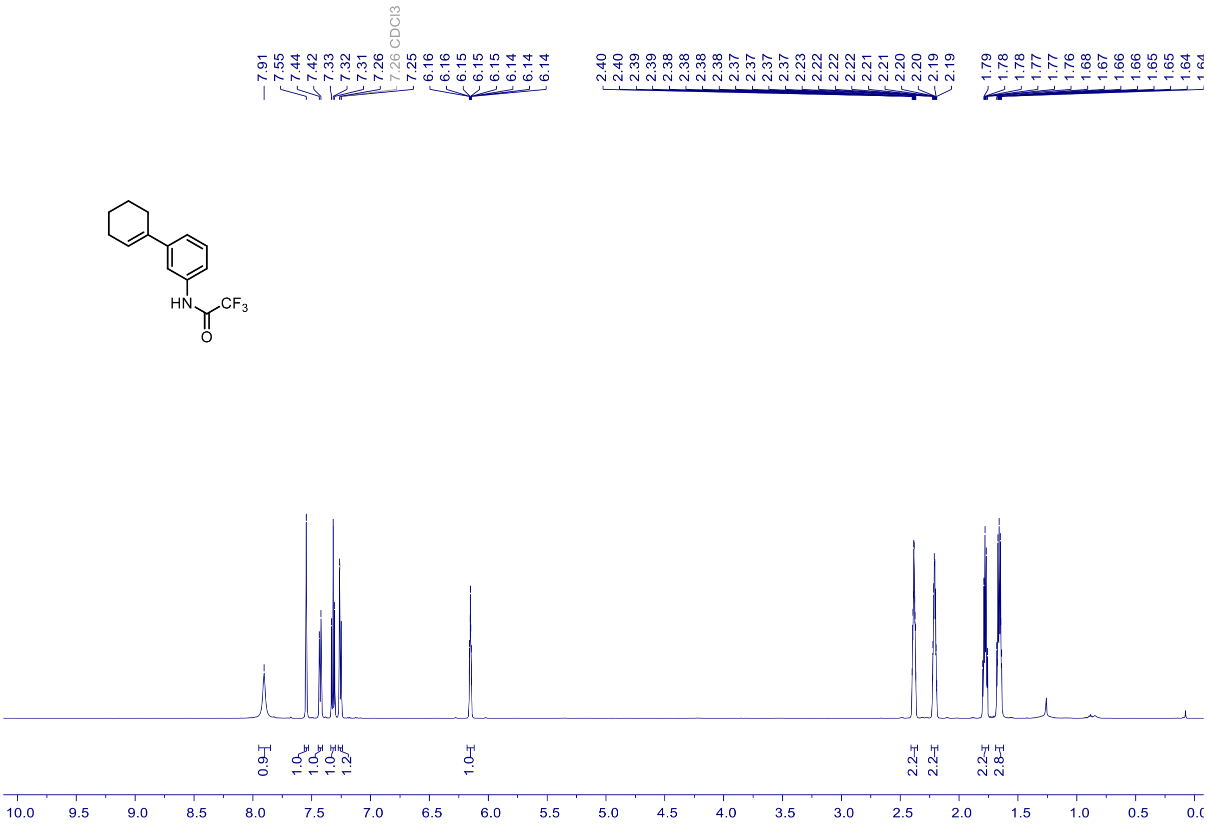

5l – ^{13}C NMR (151 MHz, $CDCl_3$)

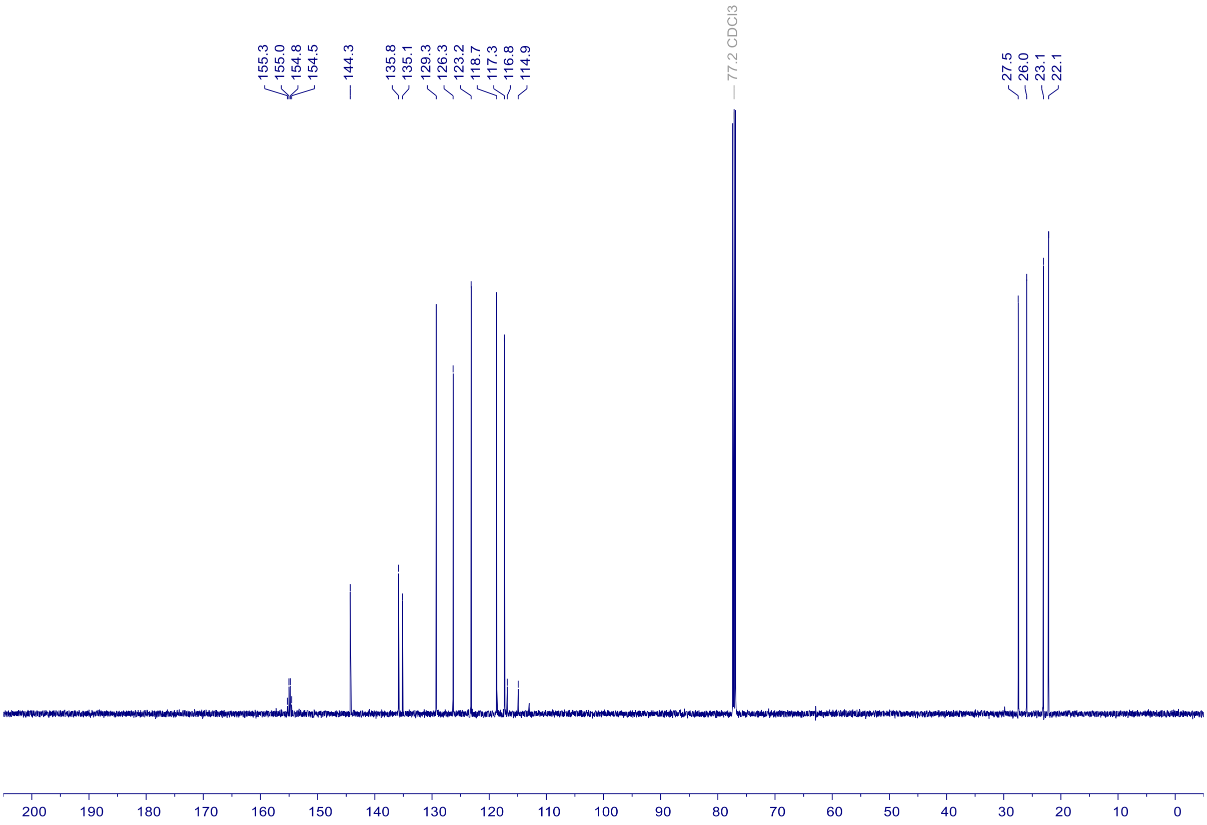

5l – ^{19}F NMR (565 MHz, $CDCl_3$)

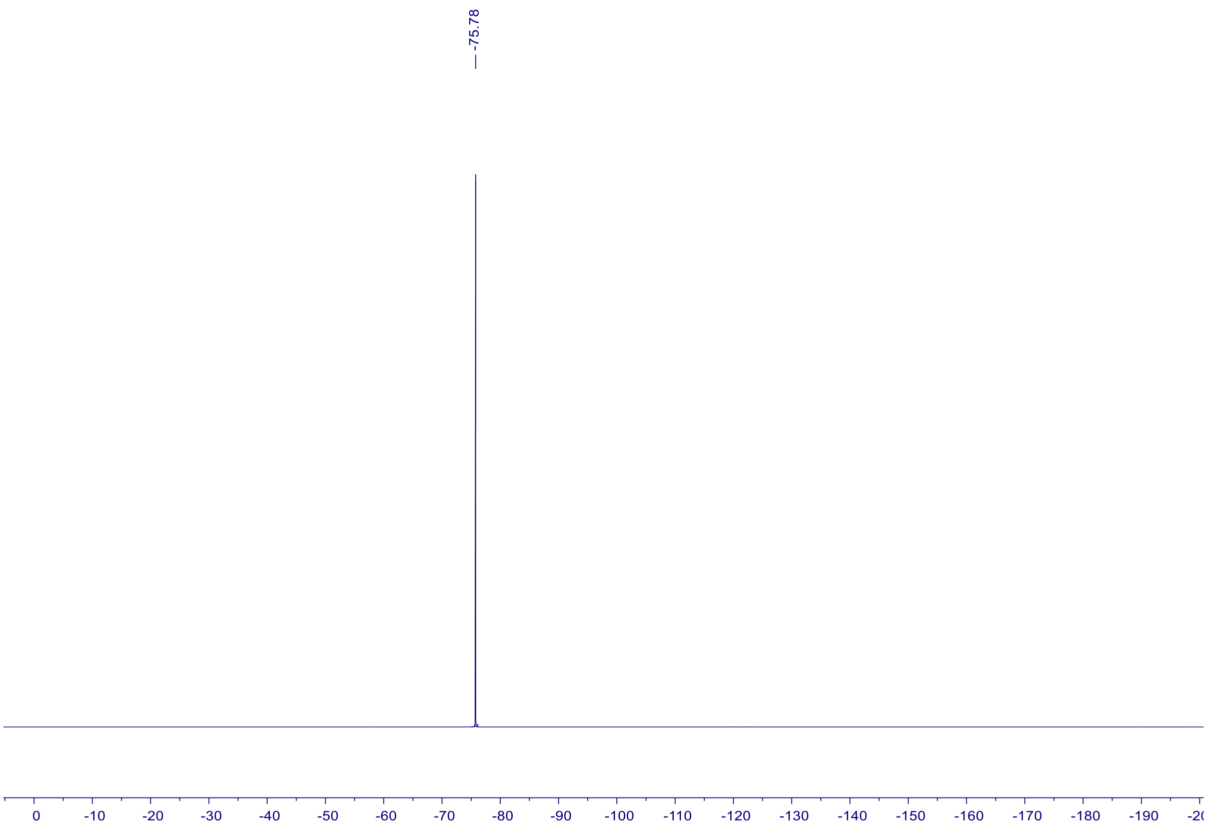

5m – ^{1}H NMR (600 MHz, $CDCl_3$)

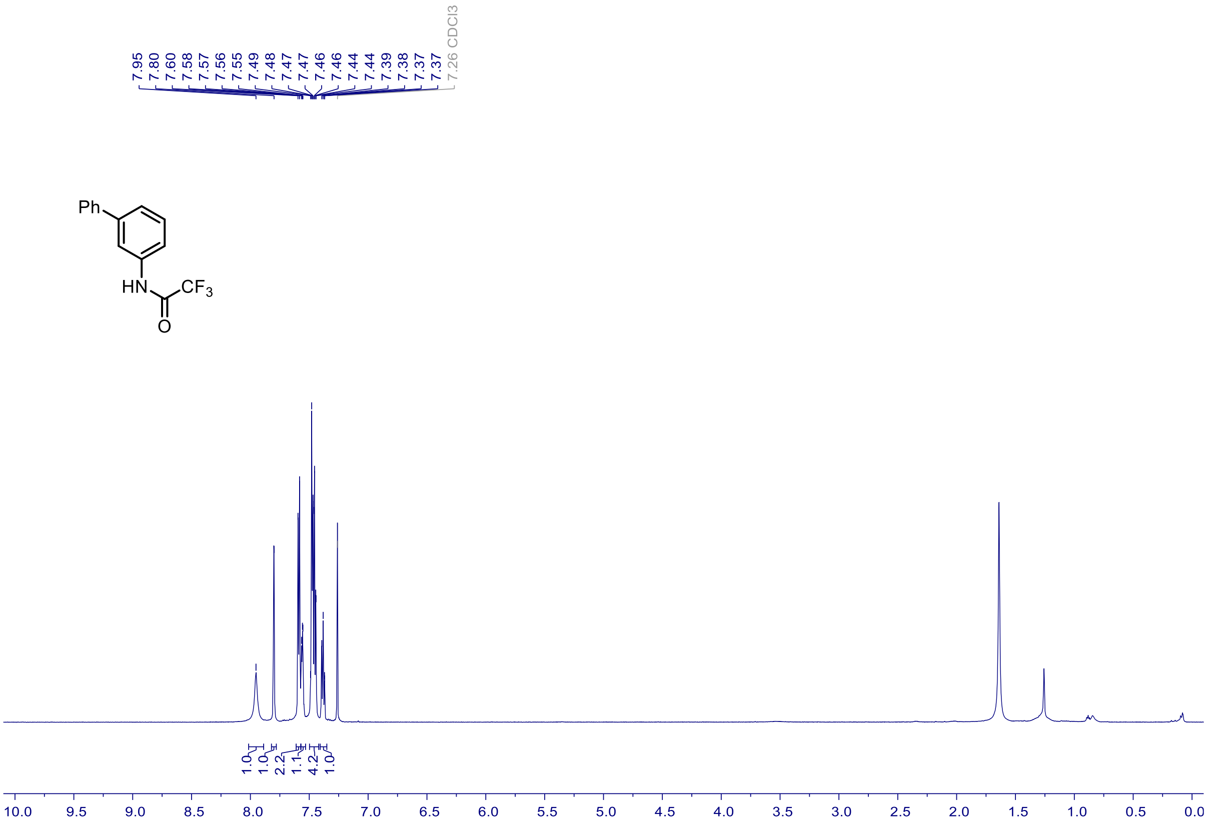

5m – ^{13}C NMR (151 MHz, $CDCl_3$)

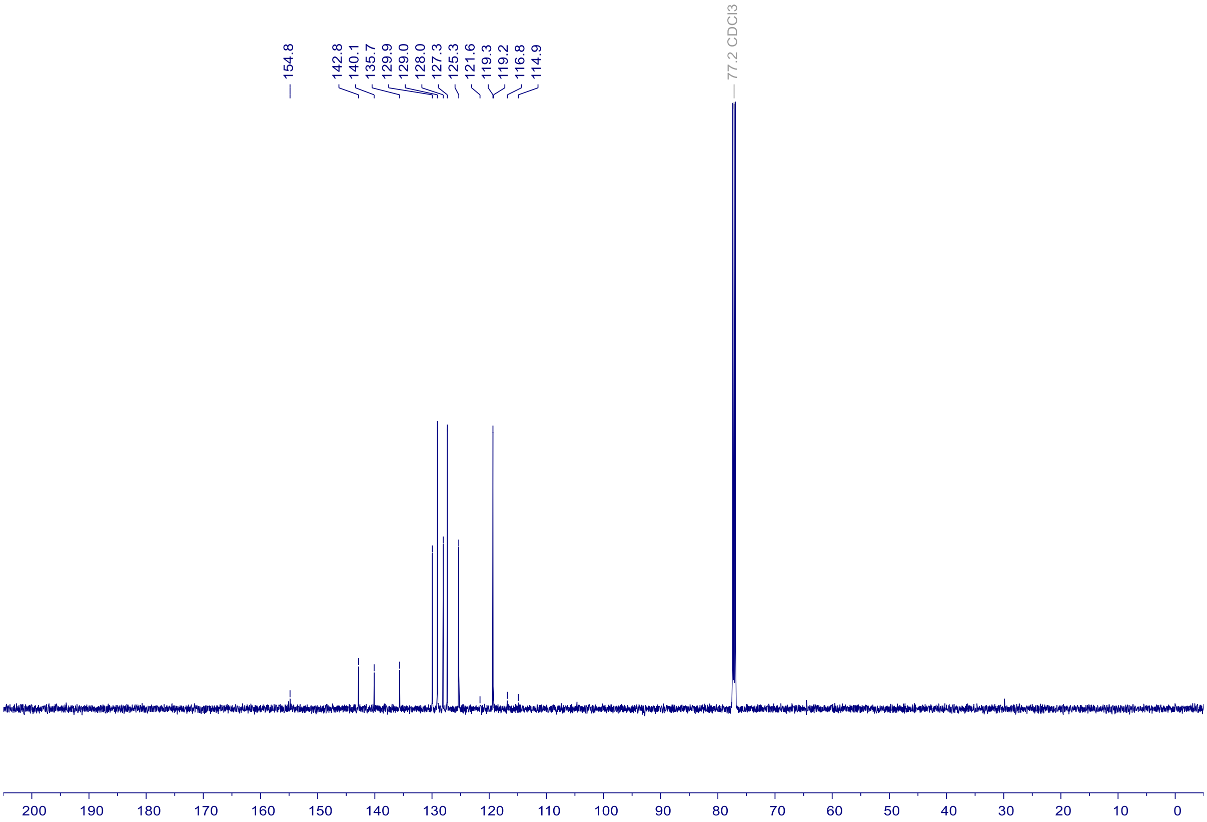

5m – ^{19}F NMR (565 MHz, $CDCl_3$)

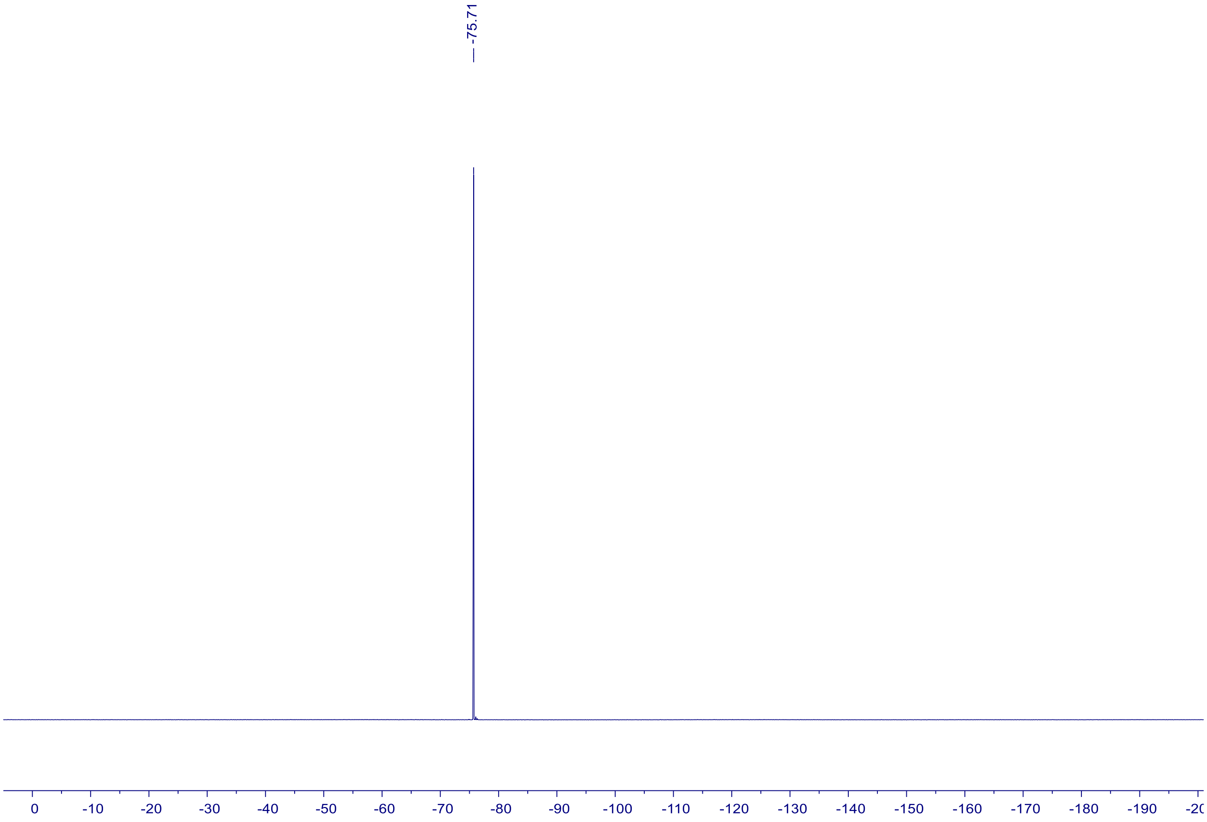

5n – ^{1}H NMR (600 MHz, $CDCl_3$)

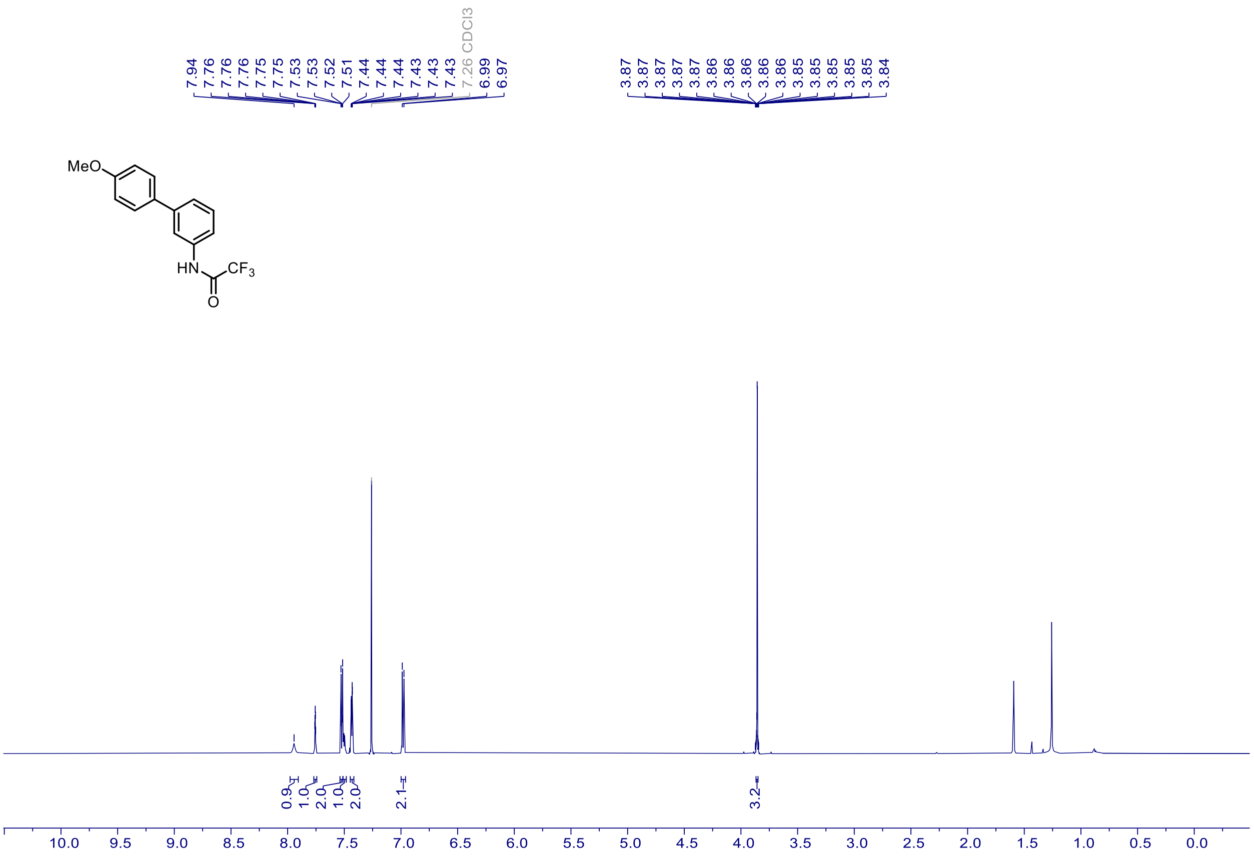

5n – ^{13}C NMR (151 MHz, $CDCl_3$)

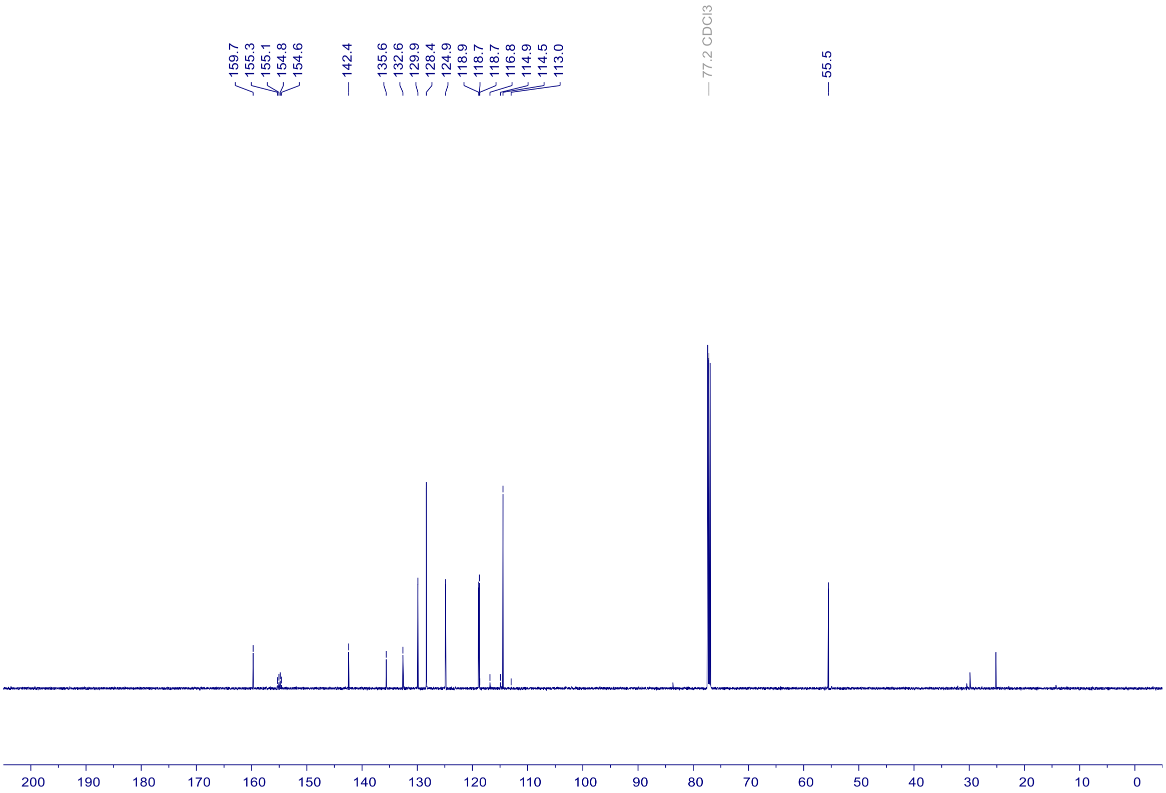

5n – ^{19}F NMR (565 MHz, $CDCl_3$)

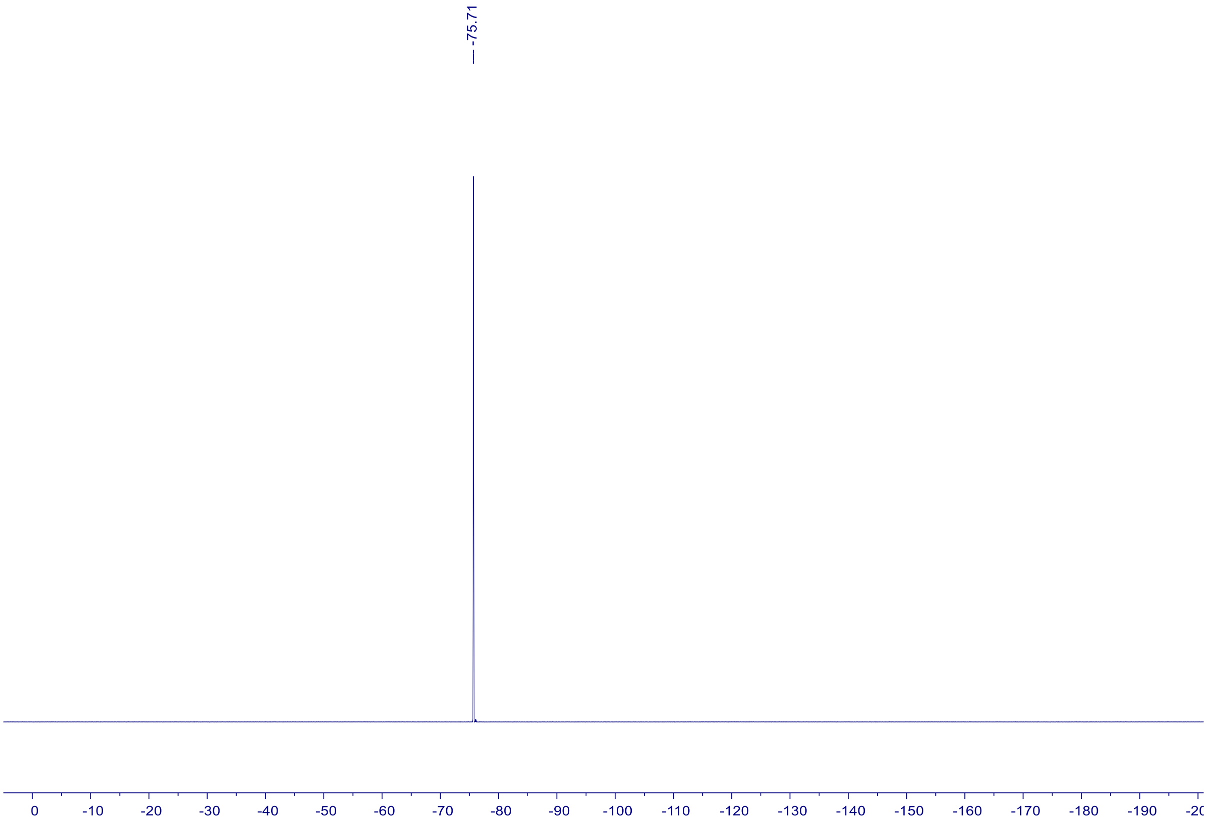

5o – ^{1}H NMR (600 MHz, $CDCl_3$)

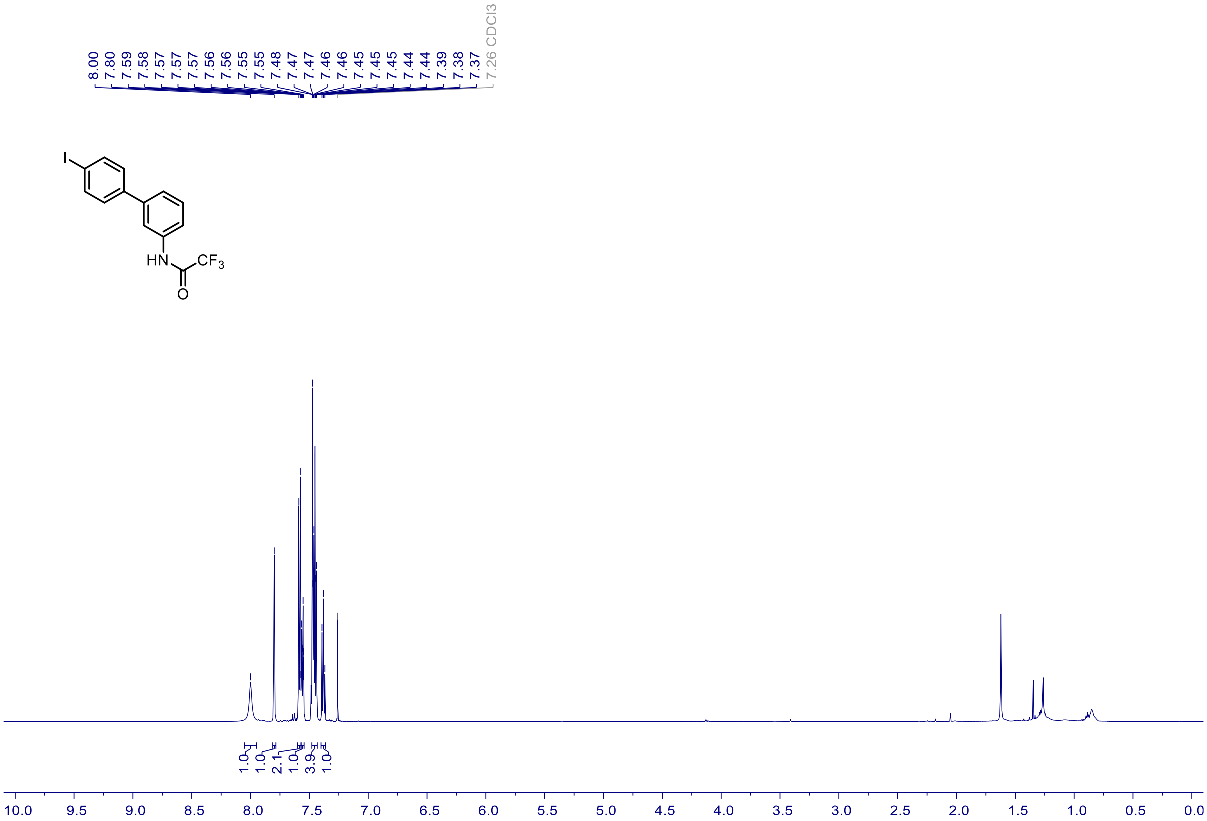

5o – ^{13}C NMR (151 MHz, $CDCl_3$)

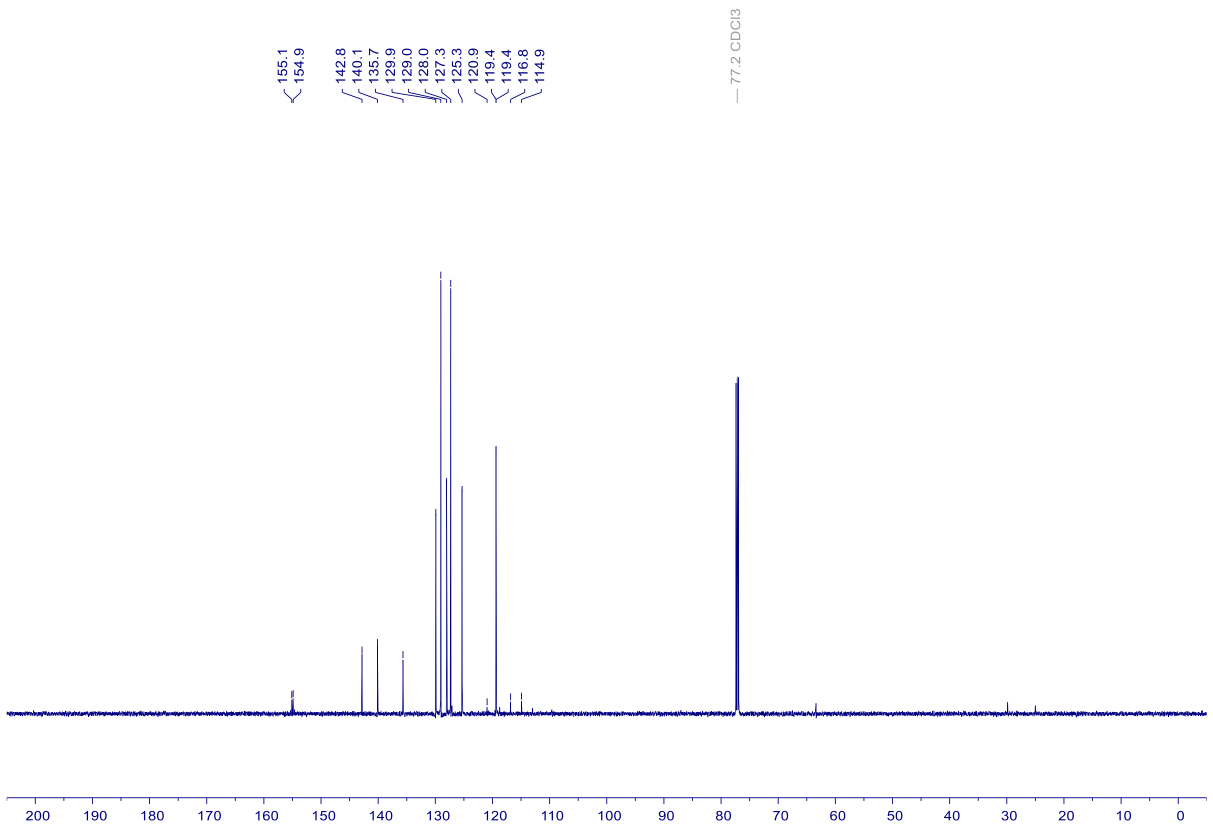

5o – ^{19}F NMR (565 MHz, $CDCl_3$)

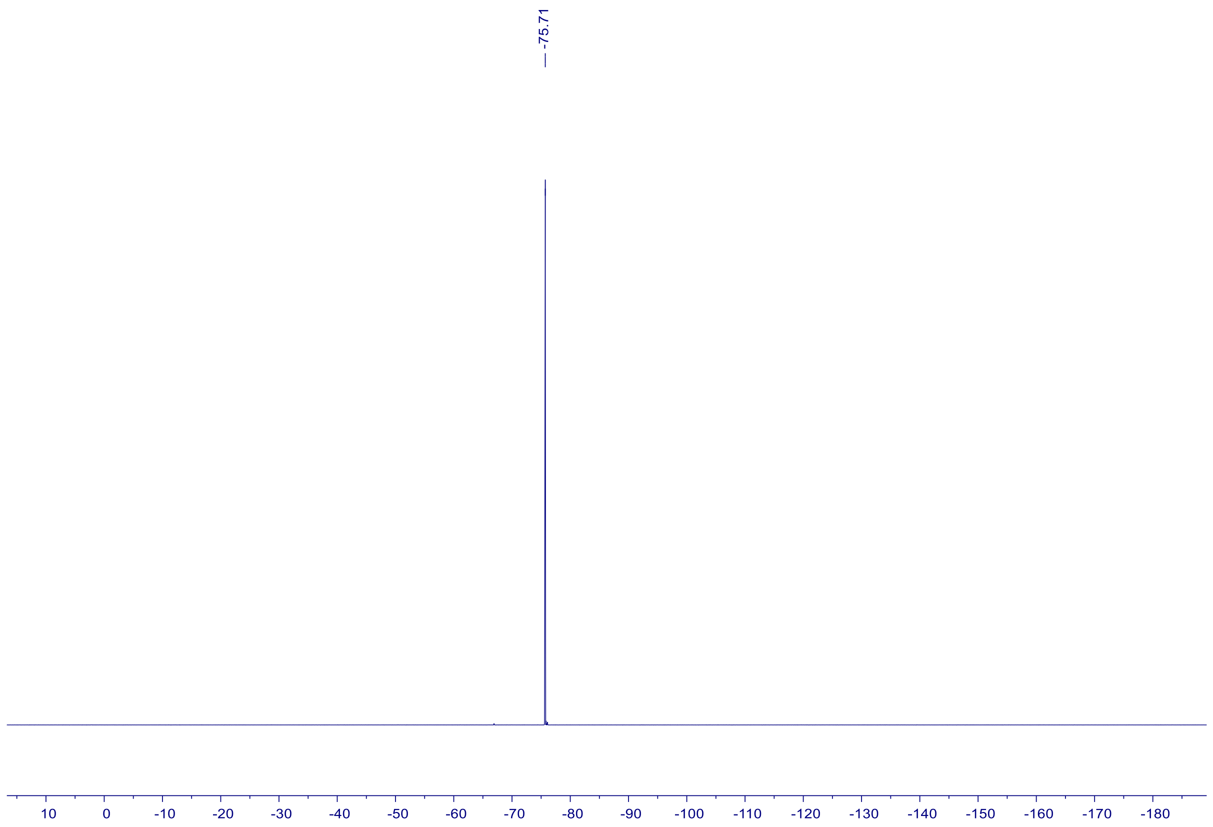

5p – ^{1}H NMR (600 MHz, $CDCl_3$)

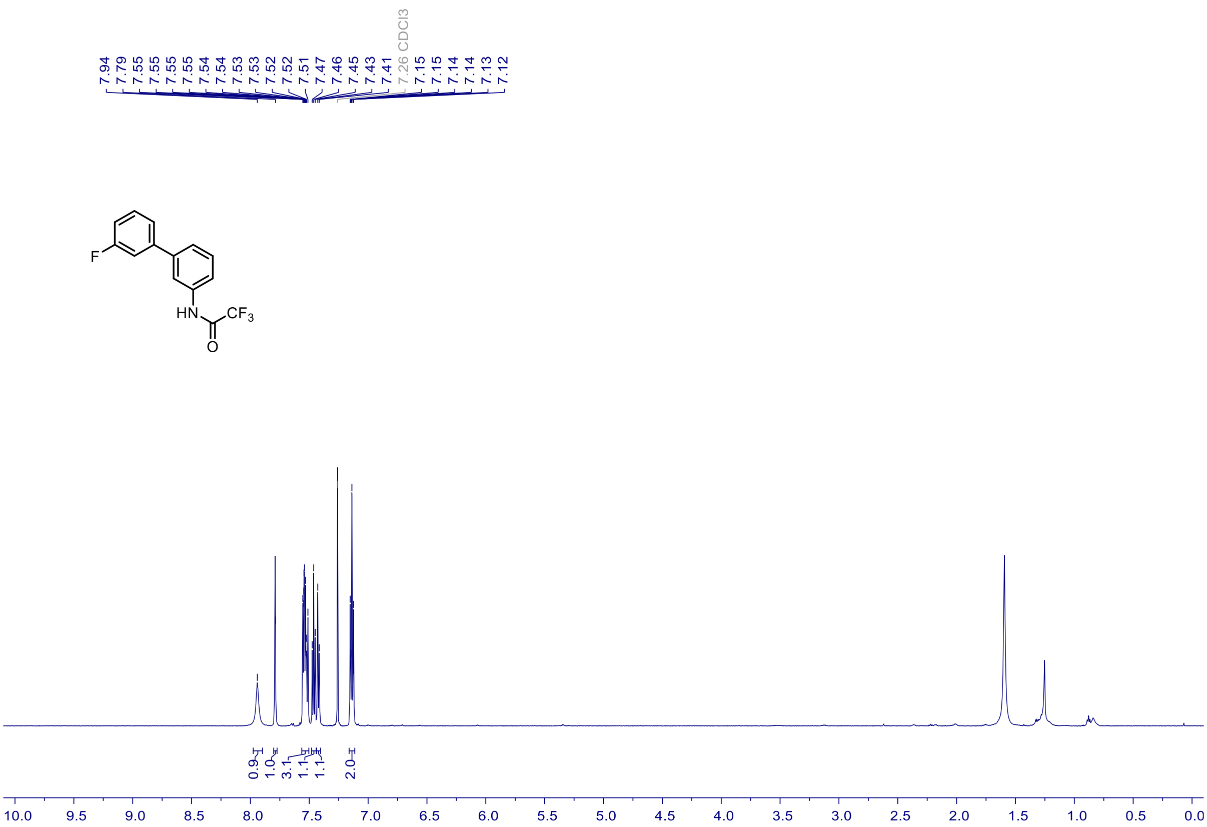

5p – ^{13}C NMR (151 MHz, $CDCl_3$)

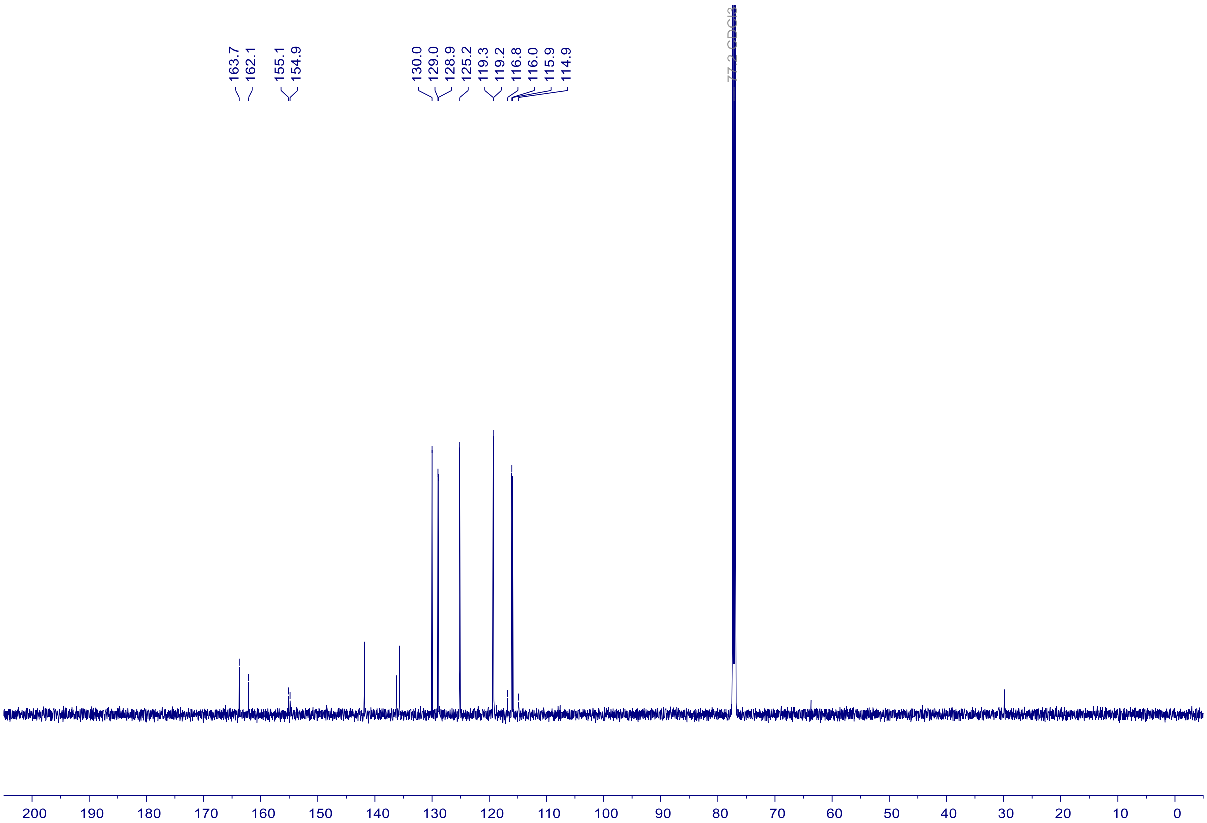

5p – ^{19}F NMR (565 MHz, $CDCl_3$)

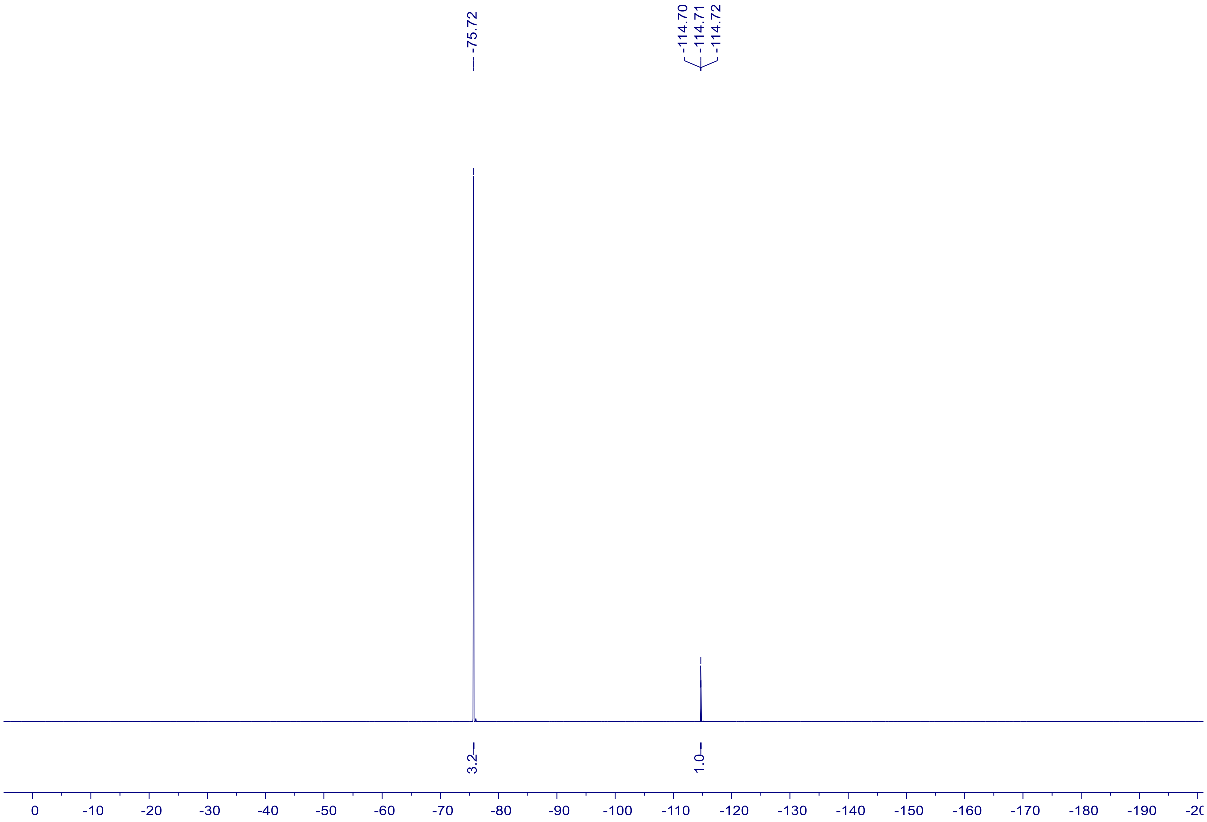

5q´ – ^{1}H NMR (600 MHz, $CDCl_3$)

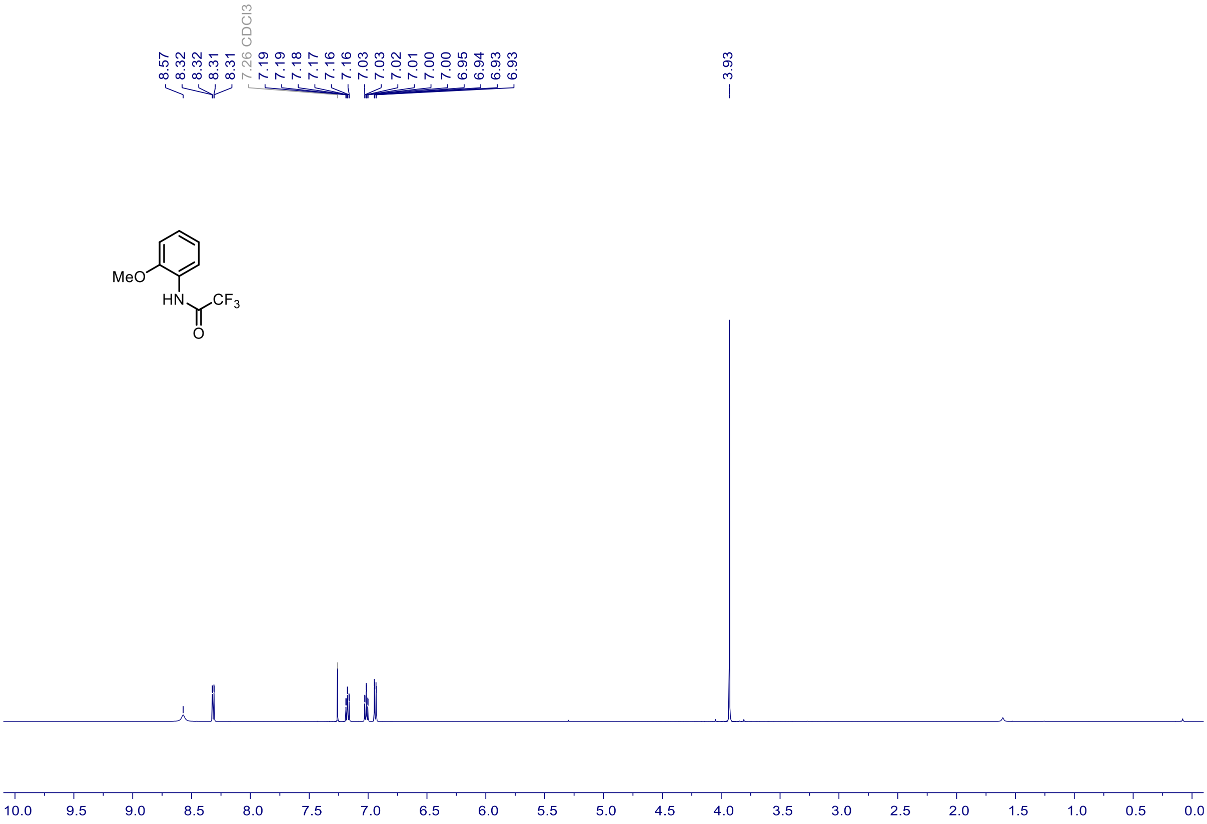

5q′ – ^{13}C NMR (151 MHz, $CDCl_3$)

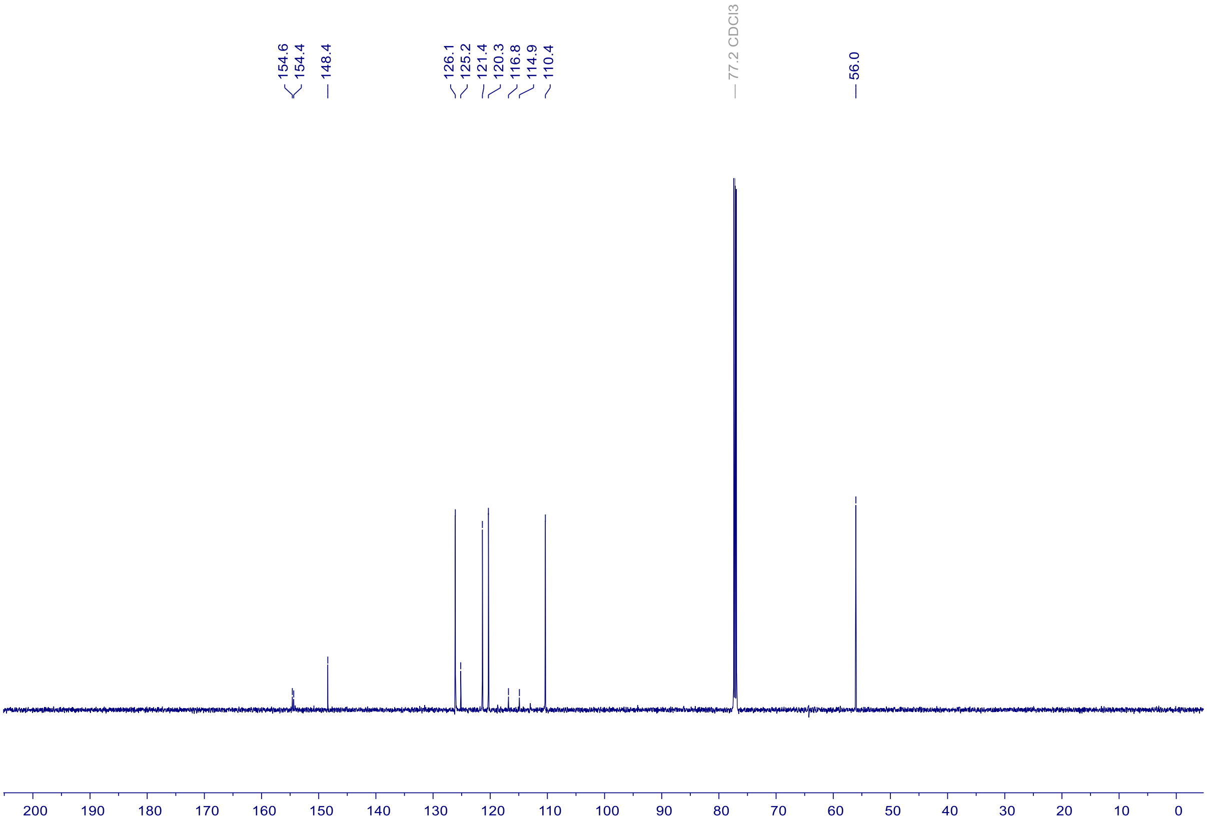

5q′ – ^{19}F NMR (565 MHz, $CDCl_3$)

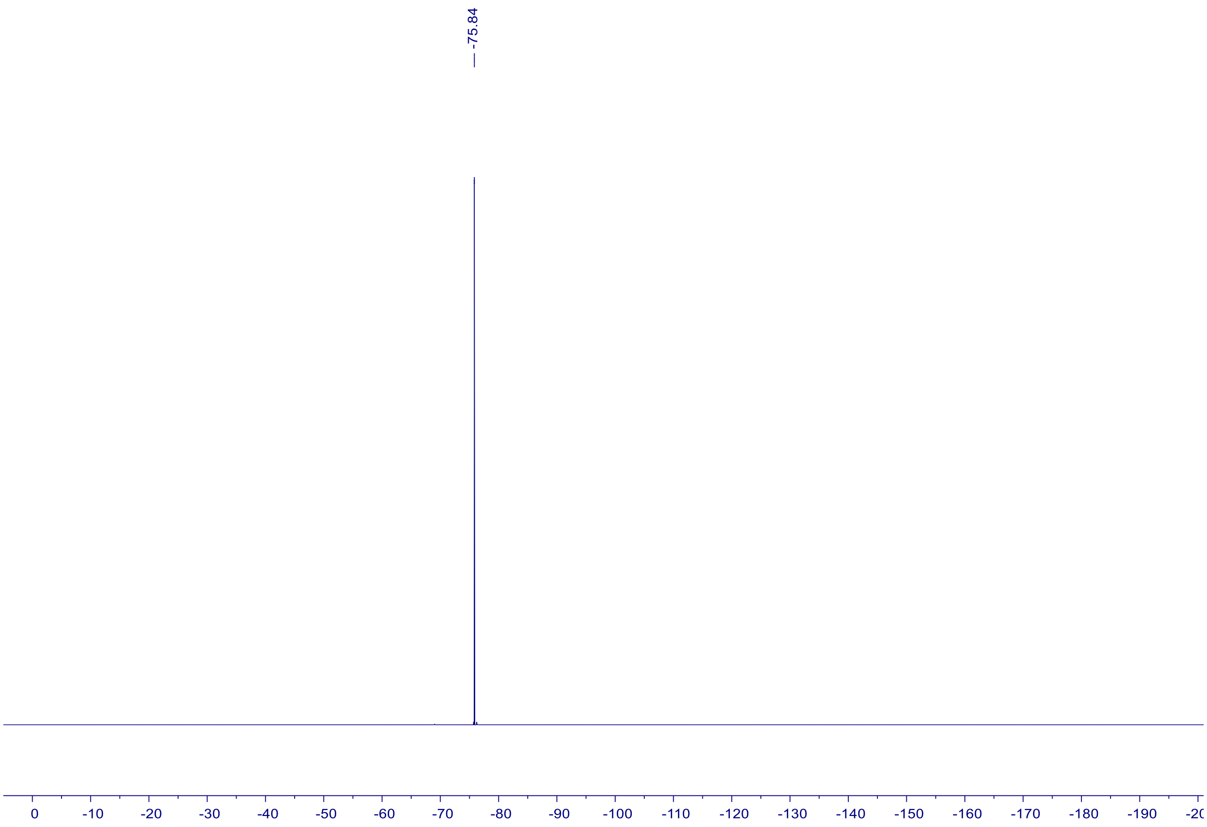

5r – ^{1}H NMR (600 MHz, $CDCl_3$)

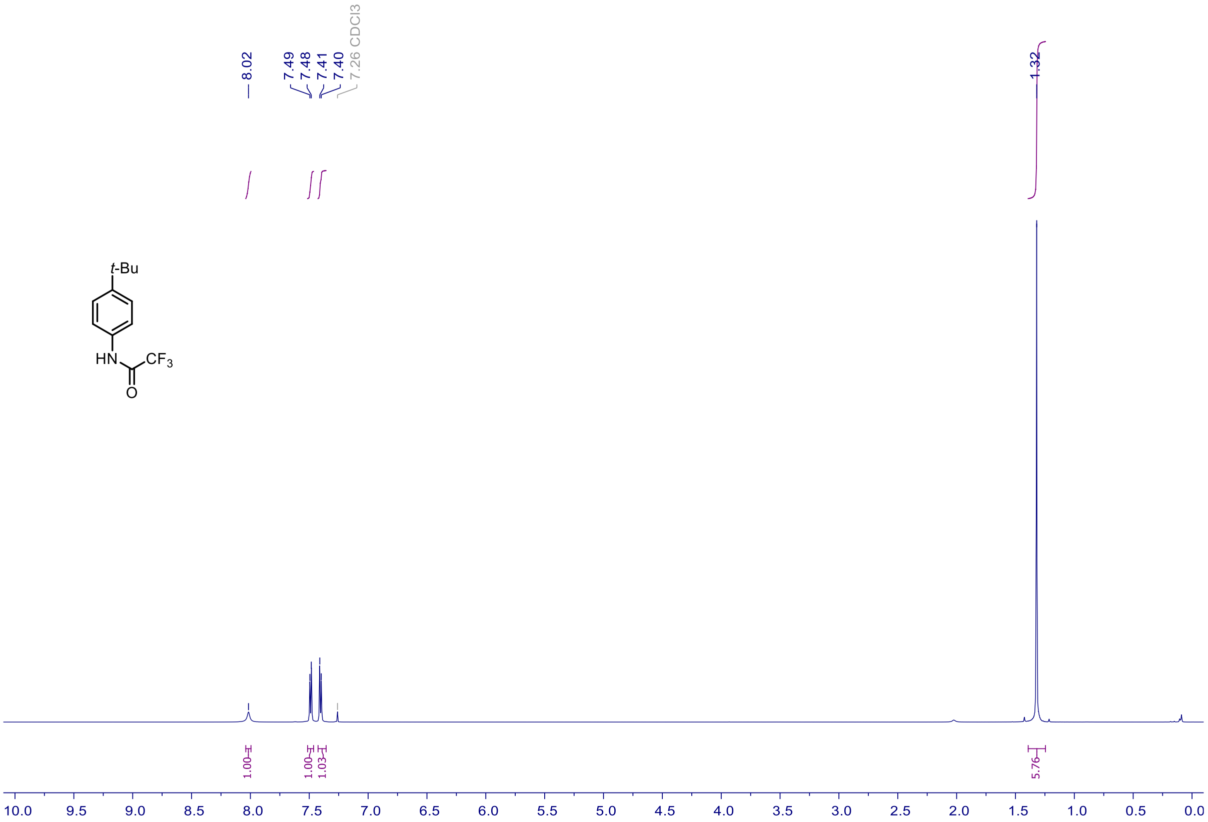

5r – ^{13}C NMR (151 MHz, $CDCl_3$)

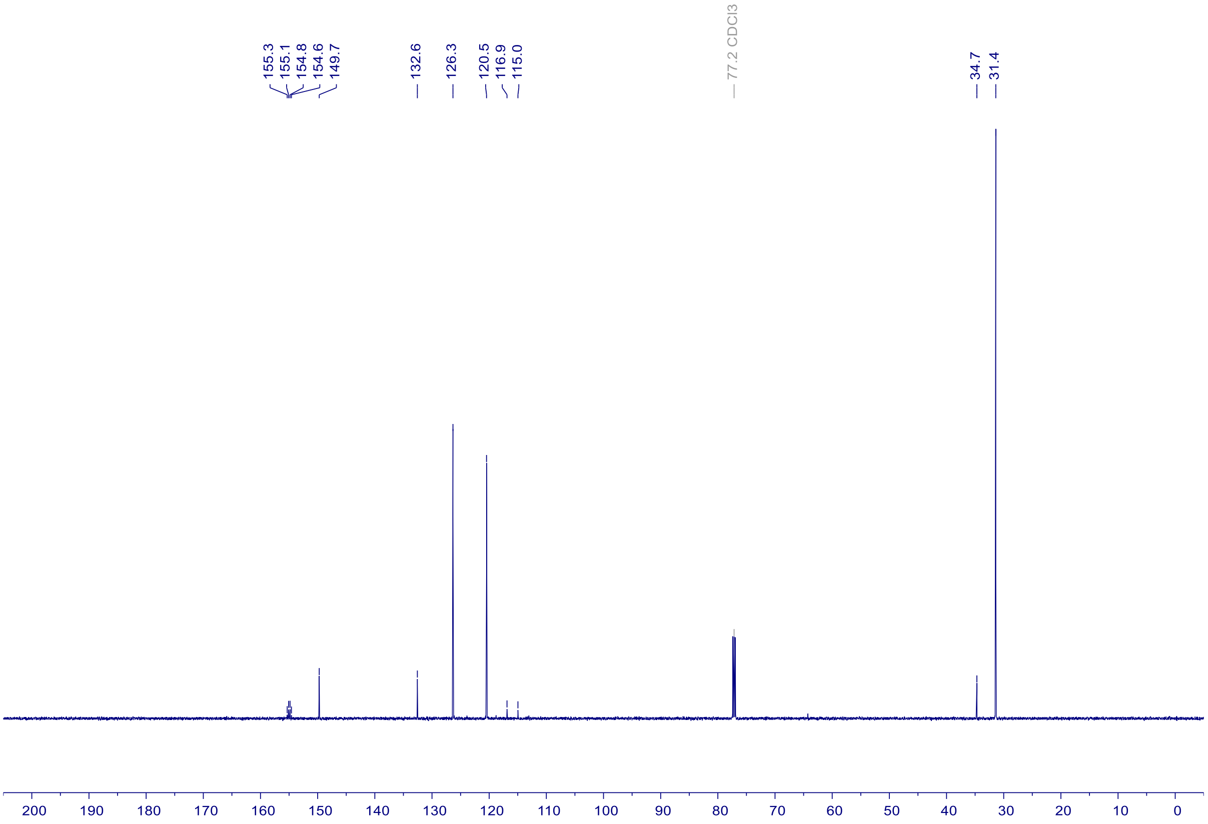

5r – ^{19}F NMR (565 MHz, $CDCl_3$)

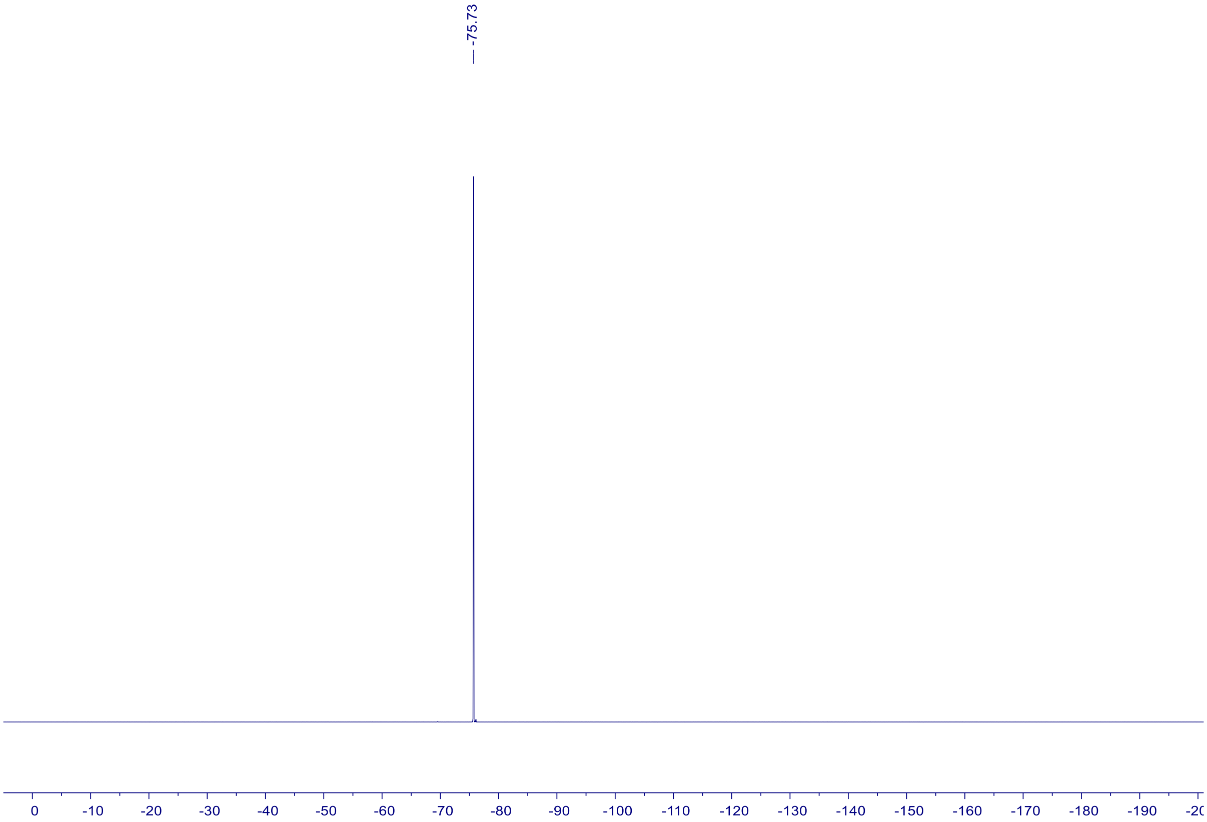

5r′ – ^{1}H NMR (600 MHz, $CDCl_3$)

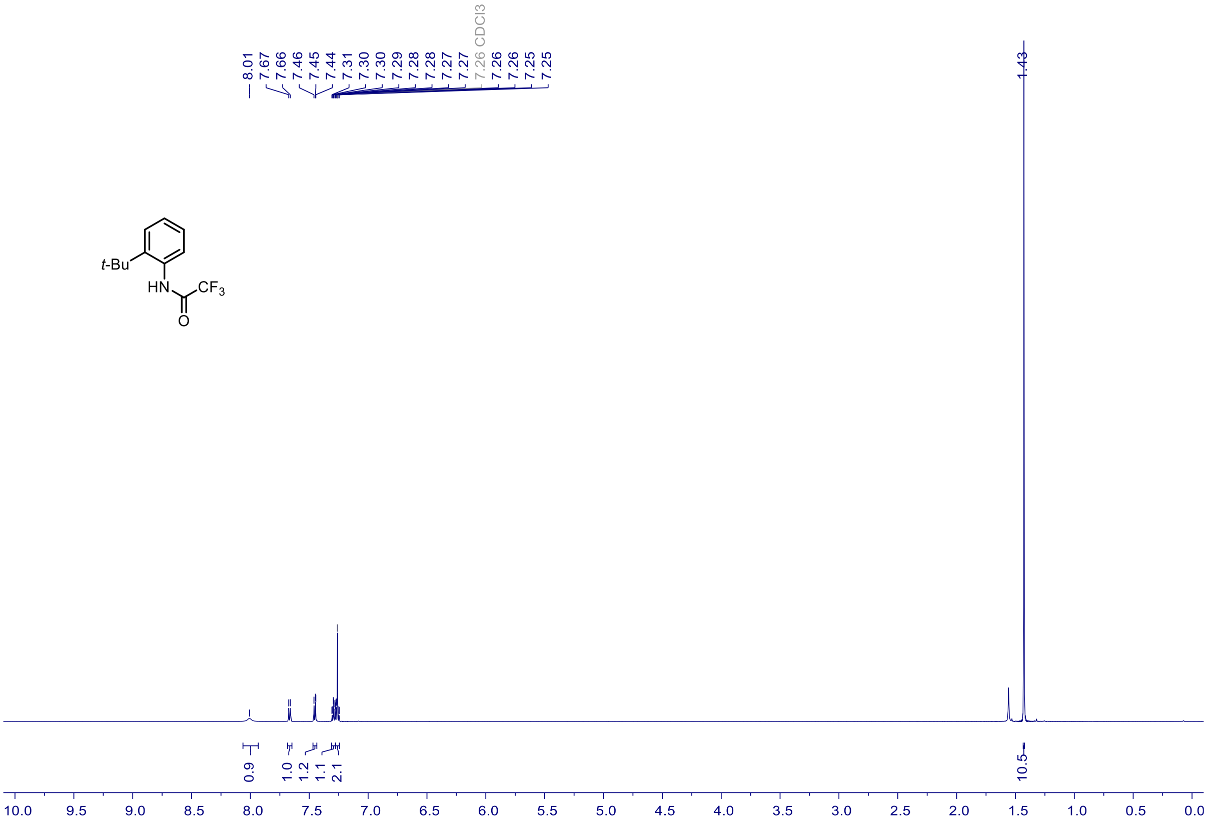

5r′ – ^{13}C NMR (151 MHz, $CDCl_3$)

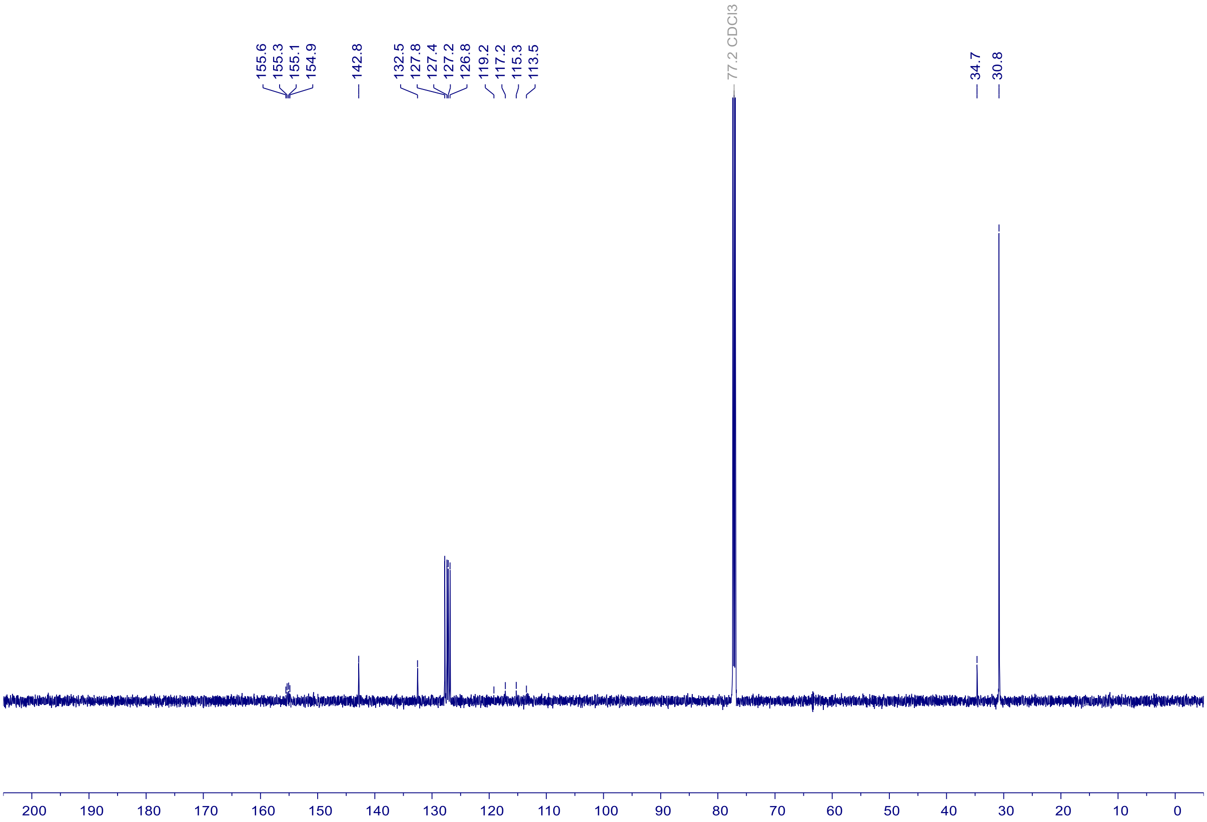

5r′ – ^{19}F NMR (565 MHz, $CDCl_3$)

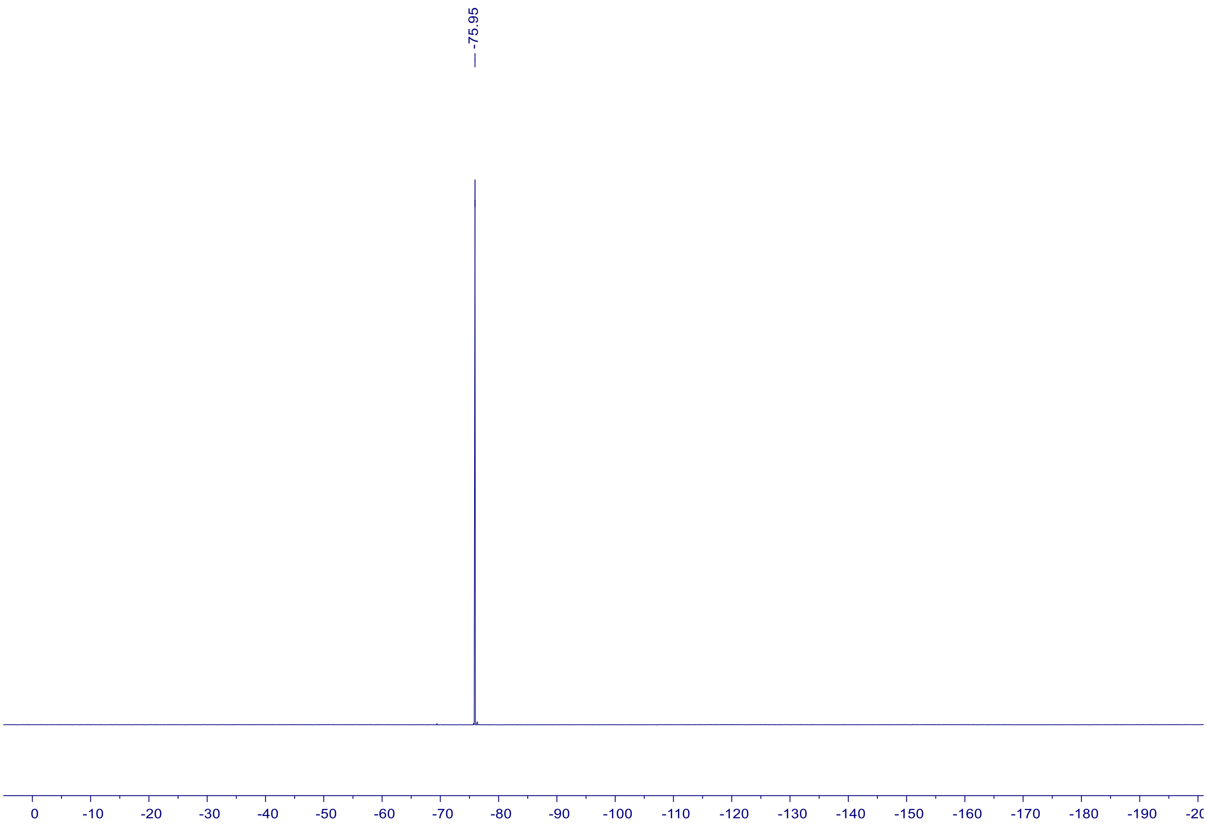

5s – ^{1}H NMR (600 MHz, $CDCl_3$)

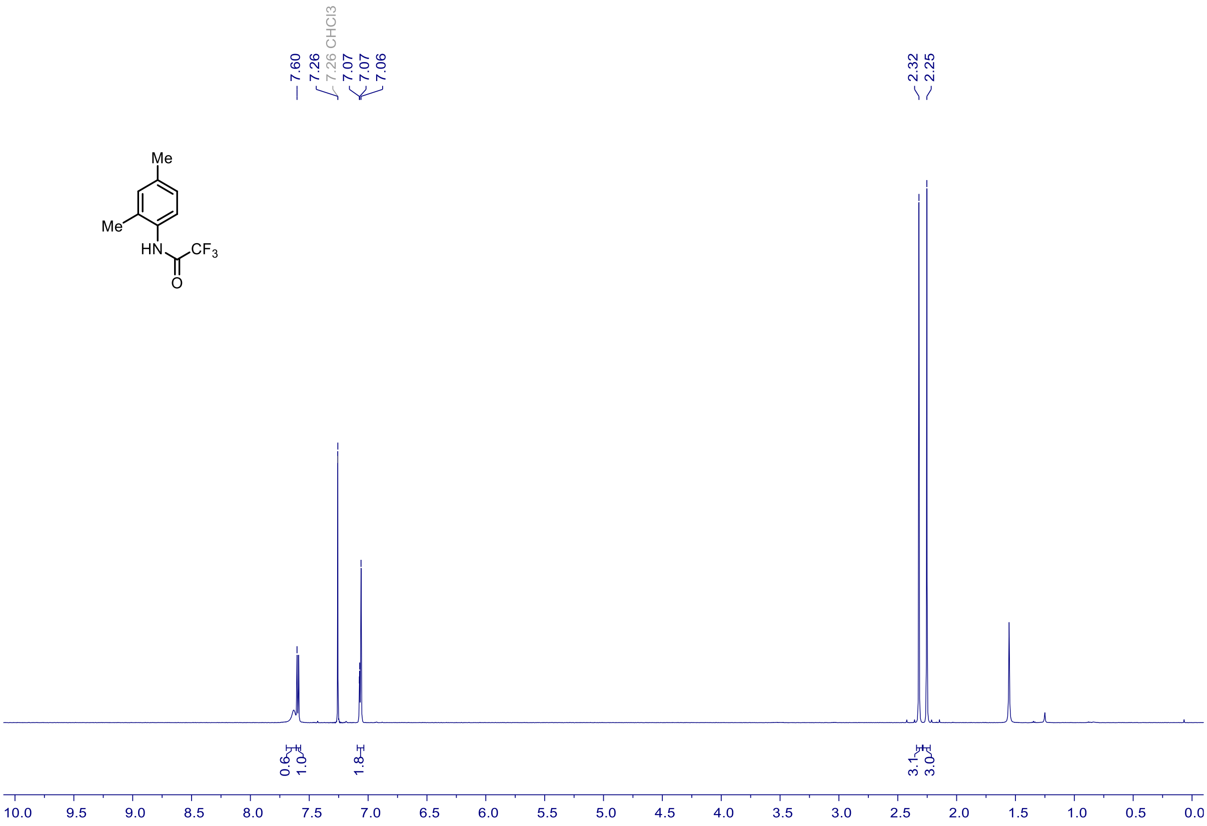

5s – ^{13}C NMR (151 MHz, $CDCl_3$)

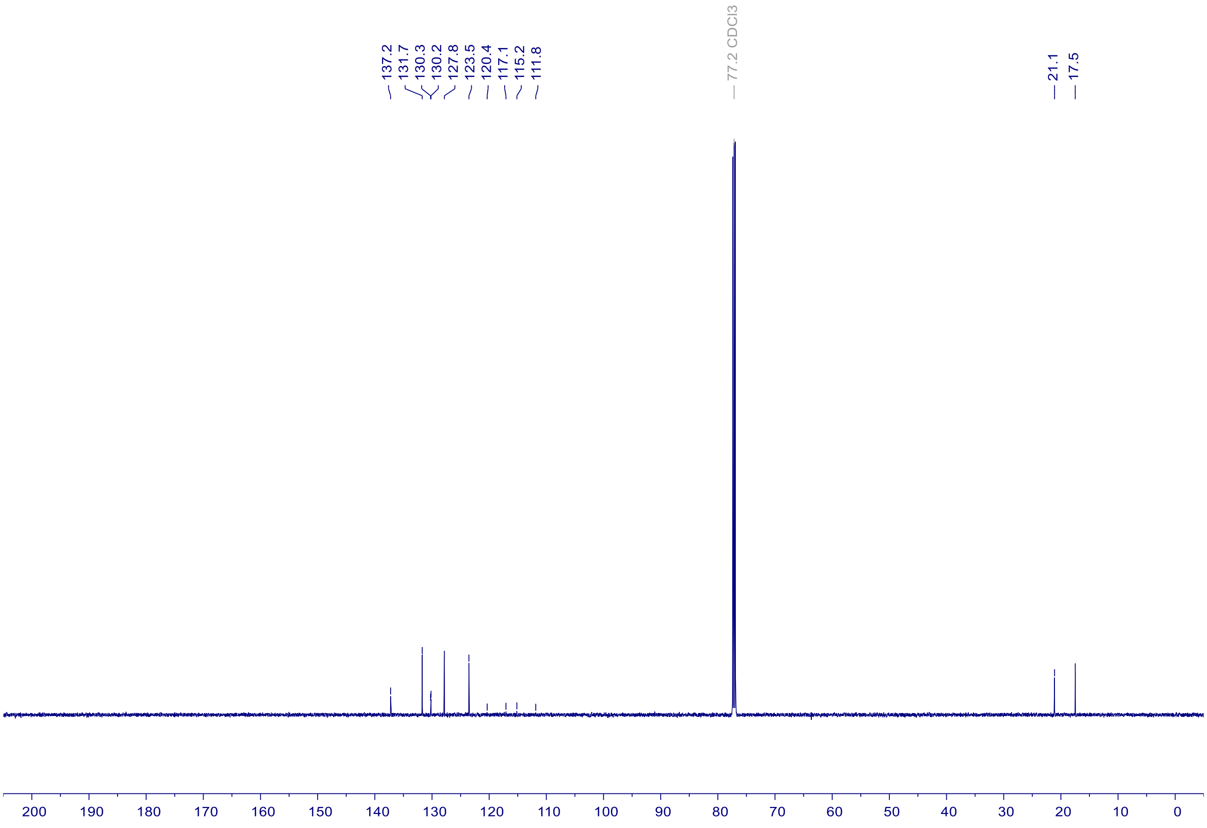

5s – ^{19}F NMR (565 MHz, $CDCl_3$)

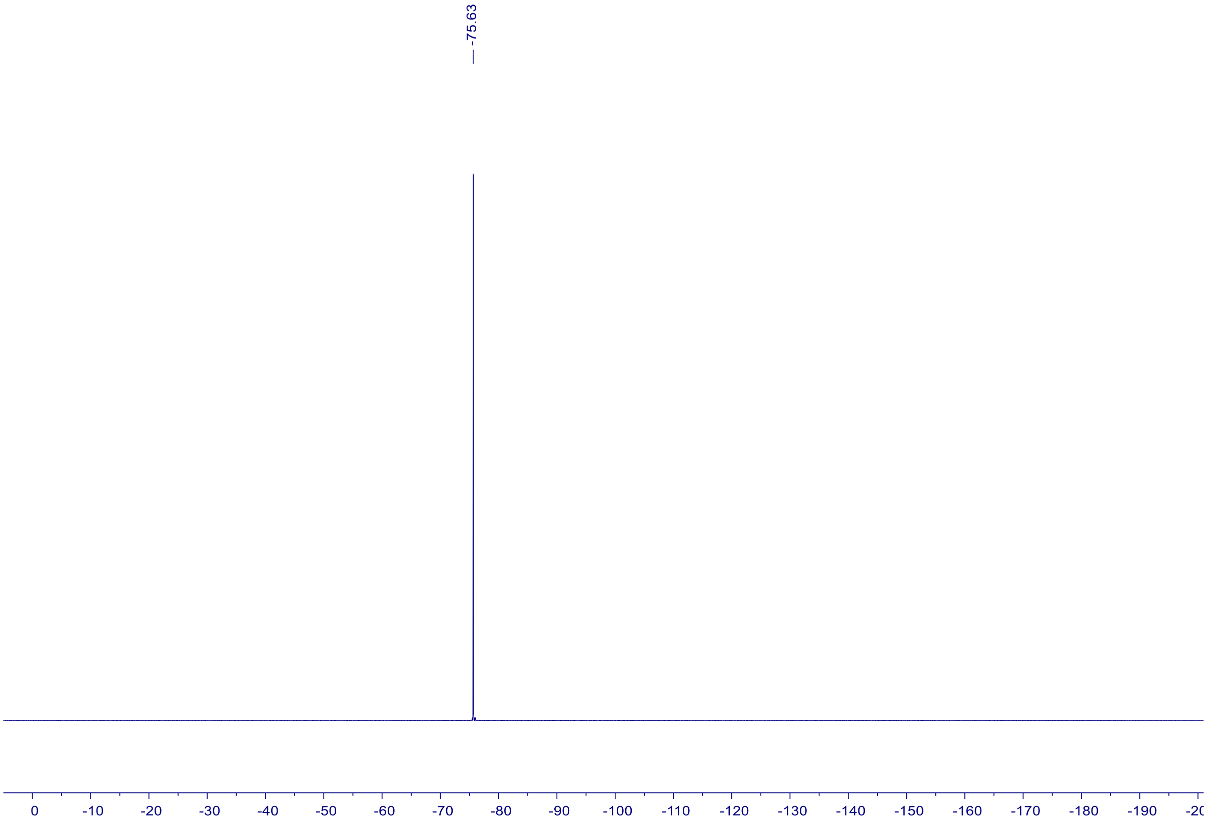

5t – ^{1}H NMR (600 MHz, $CDCl_3$)

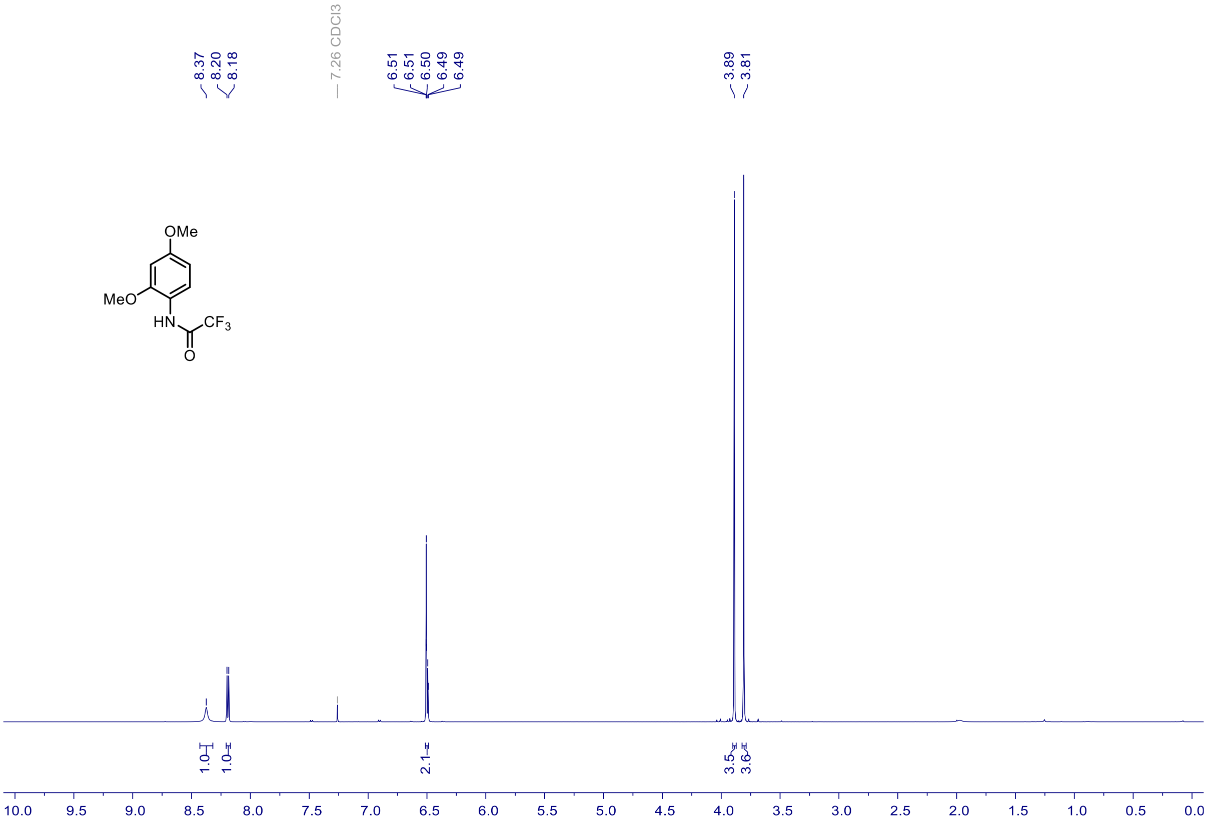

5t – ^{13}C NMR (151 MHz, CDCl$_3$)

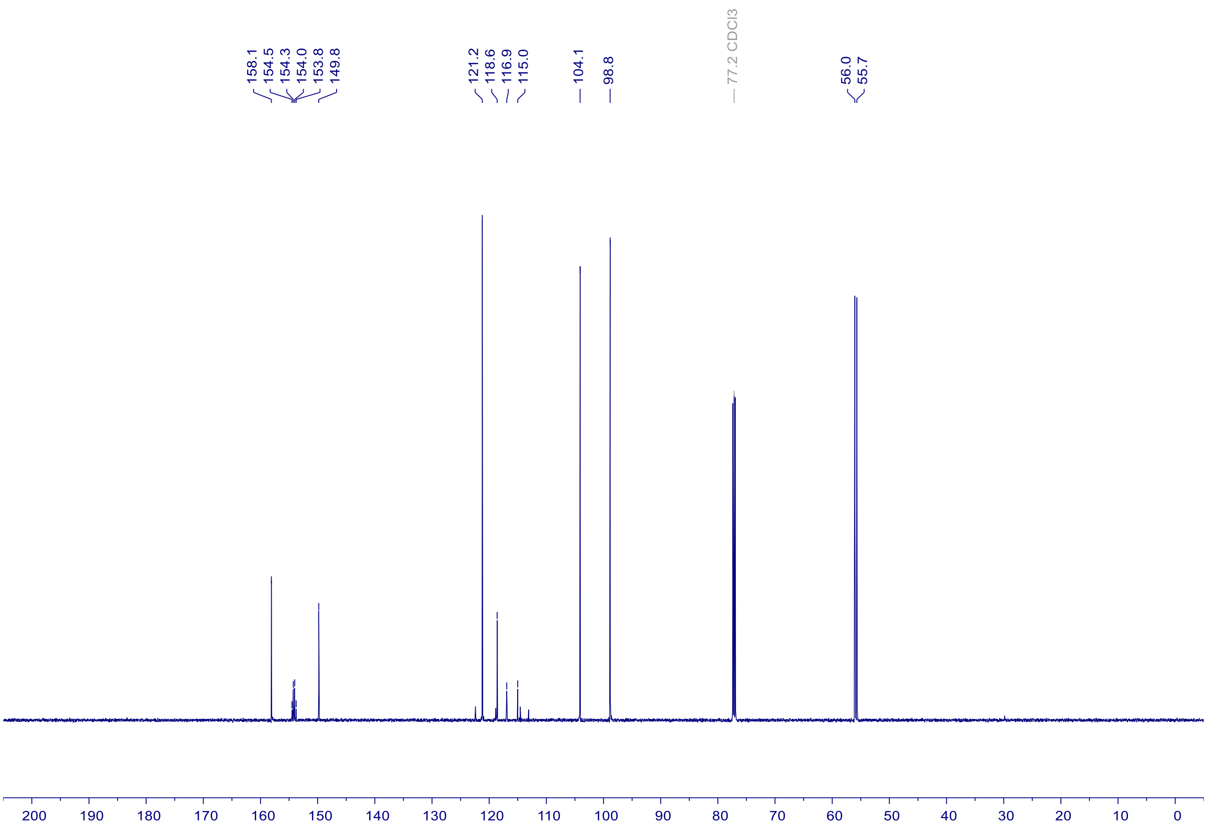

5t – ^{19}F NMR (565 MHz, CDCl$_3$)

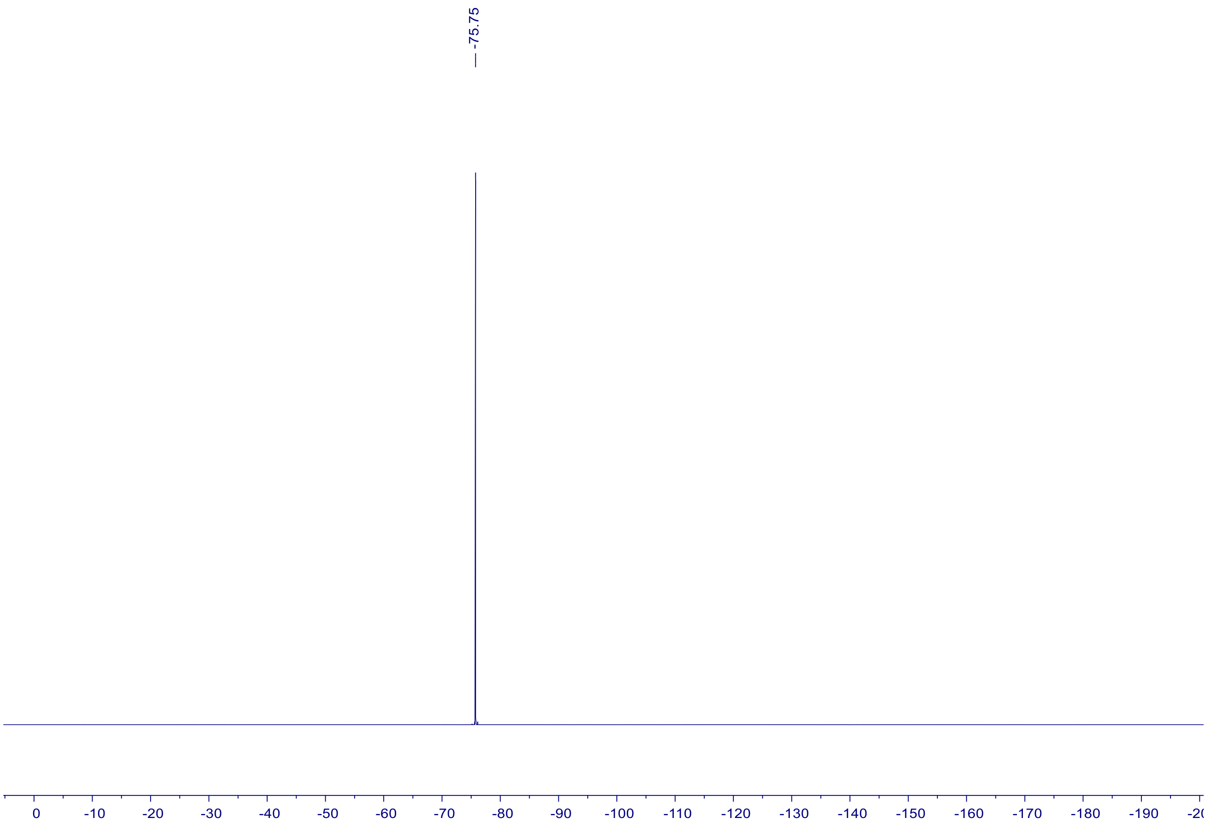

5u – ^{1}H NMR (600 MHz, $CDCl_3$)

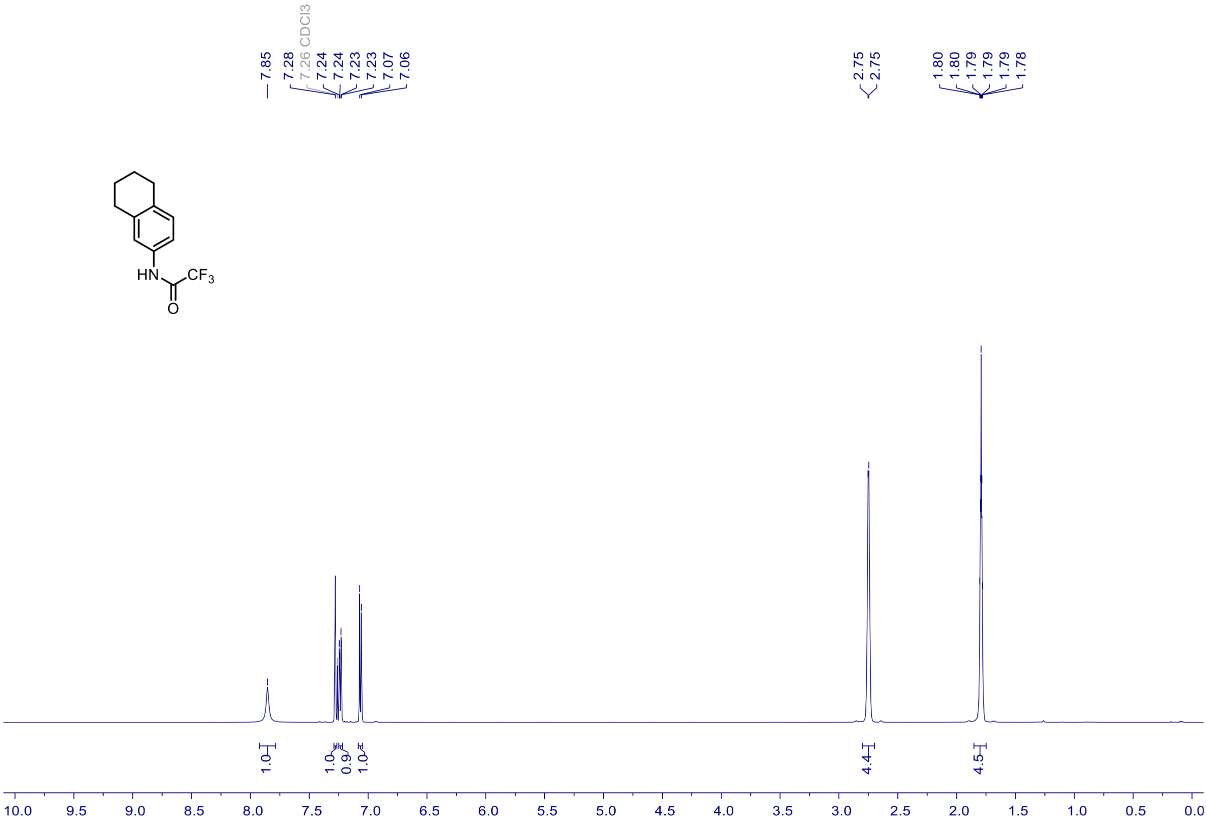

5u – ^{13}C NMR (151 MHz, $CDCl_3$)

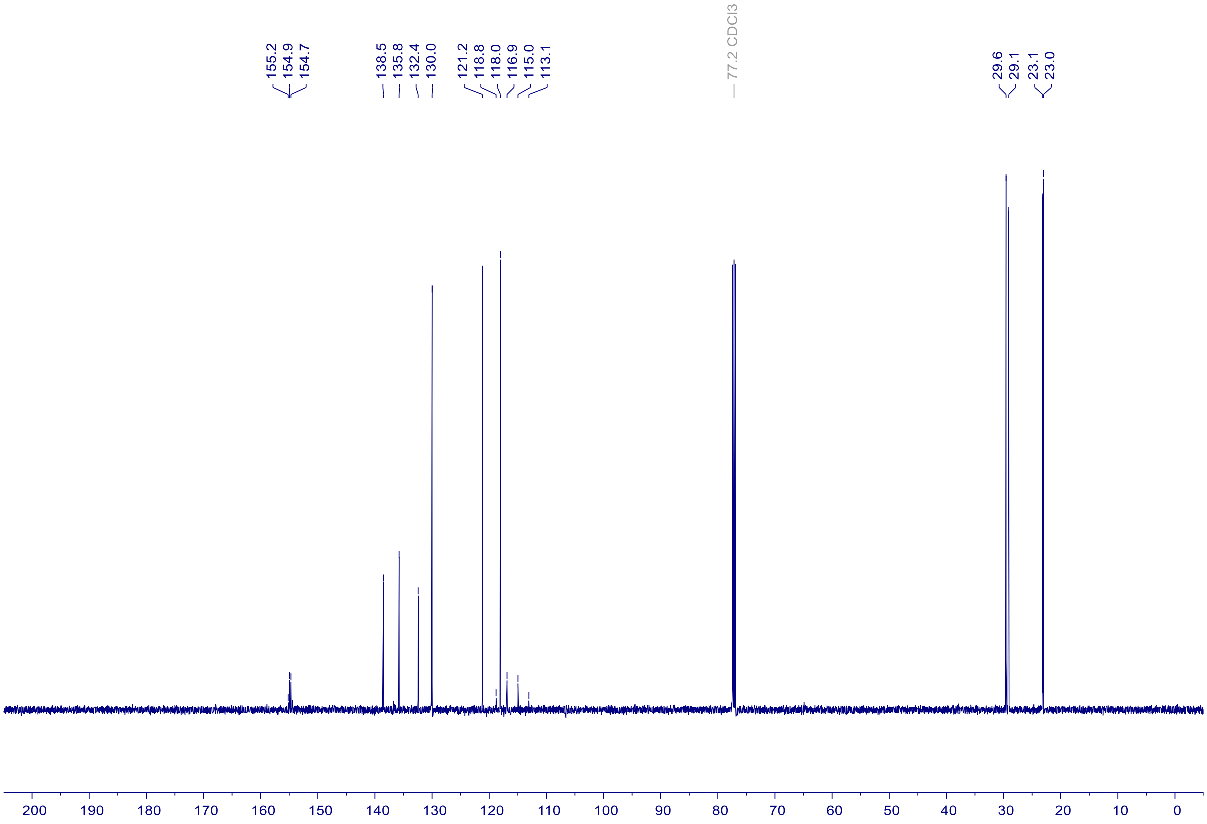

5u – ^{19}F NMR (565 MHz, $CDCl_3$)

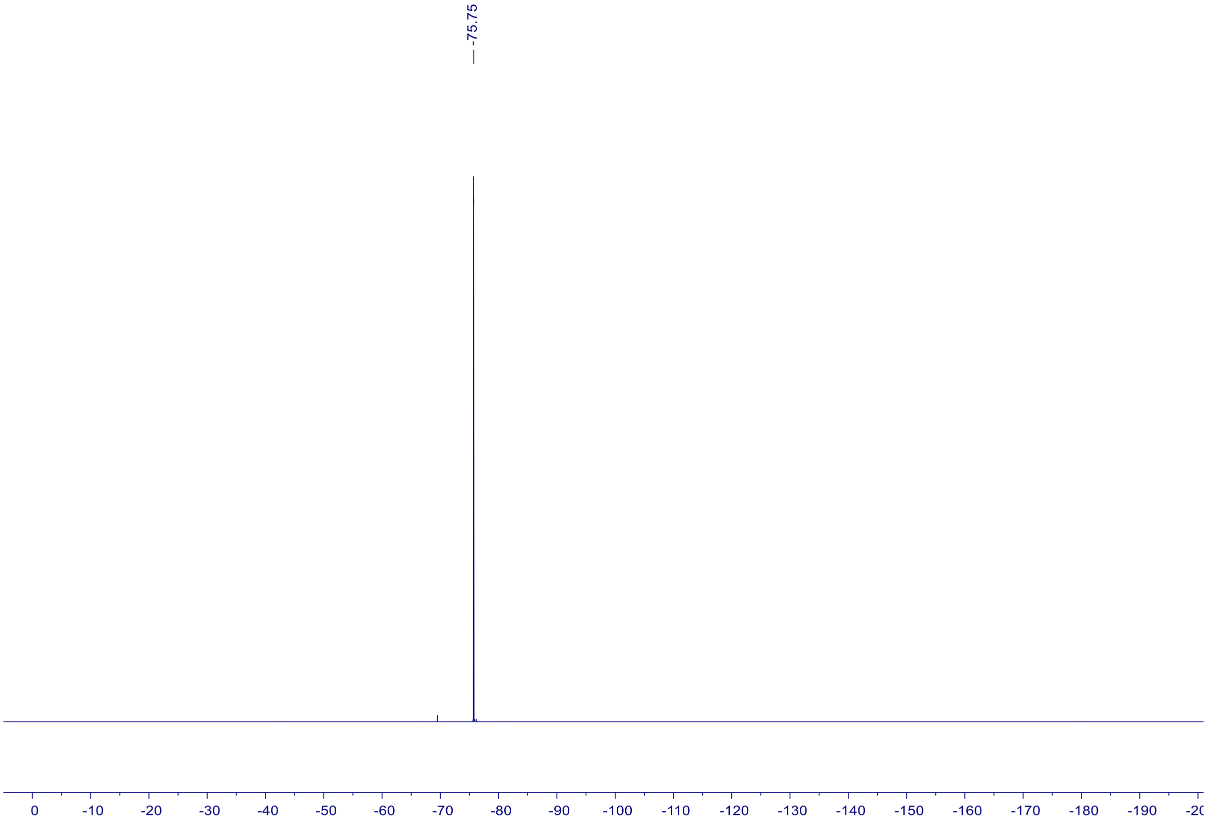

5u′– ^{1}H NMR (600 MHz, $CDCl_3$)

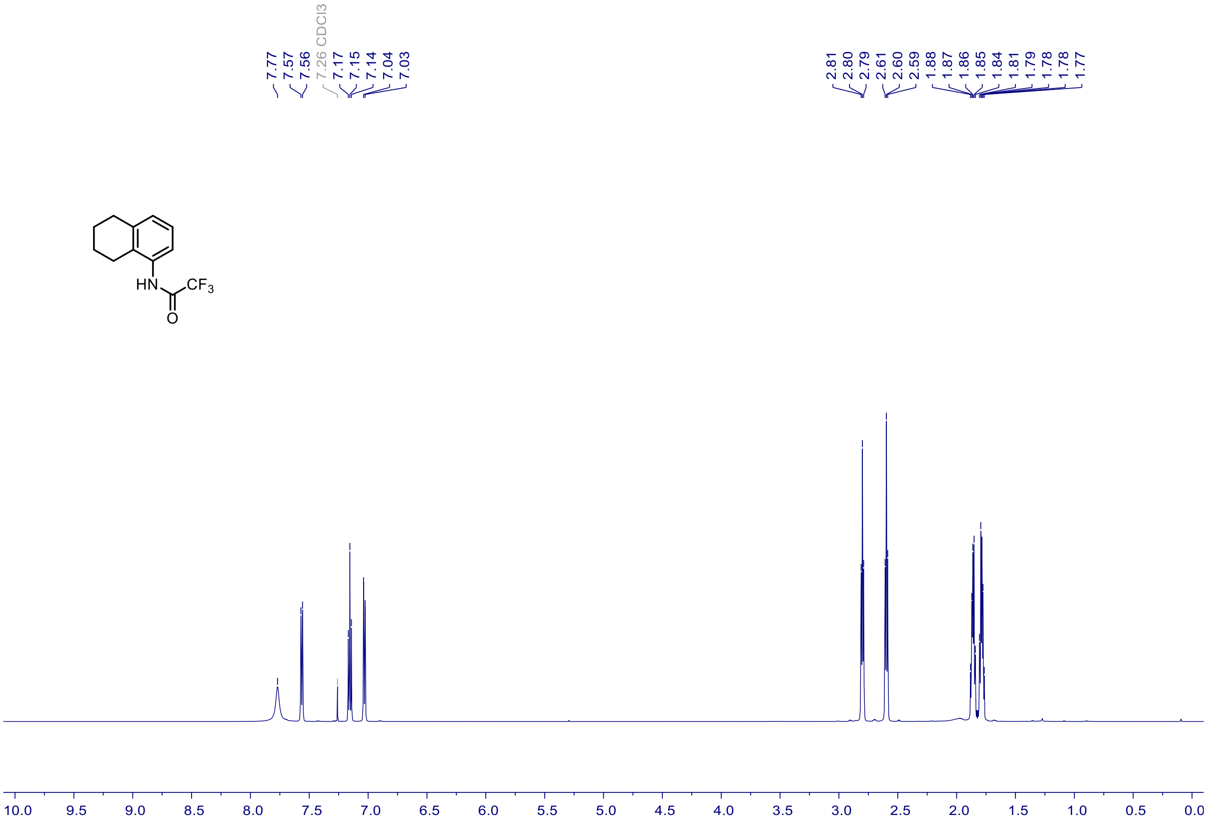

5u′ – ^{13}C NMR (151 MHz, CDCl$_3$)

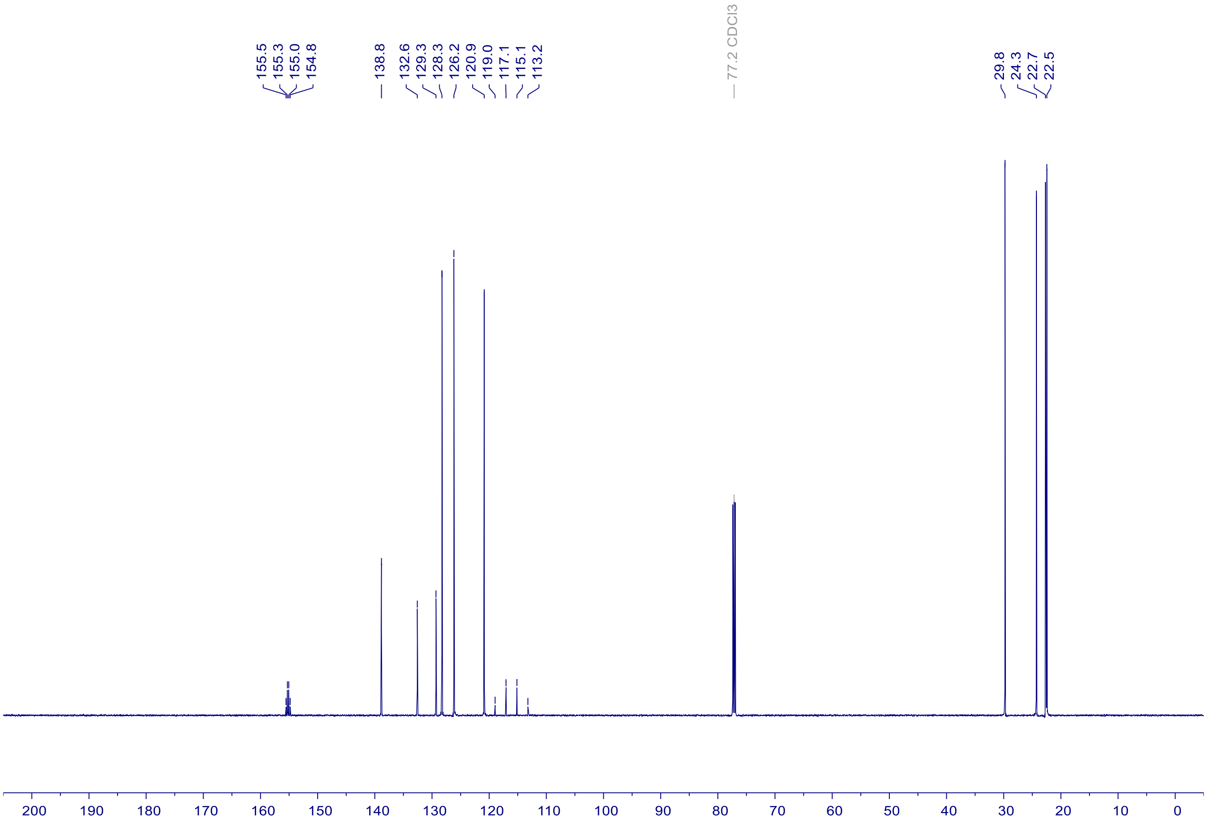

5u′ – ^{19}F NMR (565 MHz, CDCl$_3$)

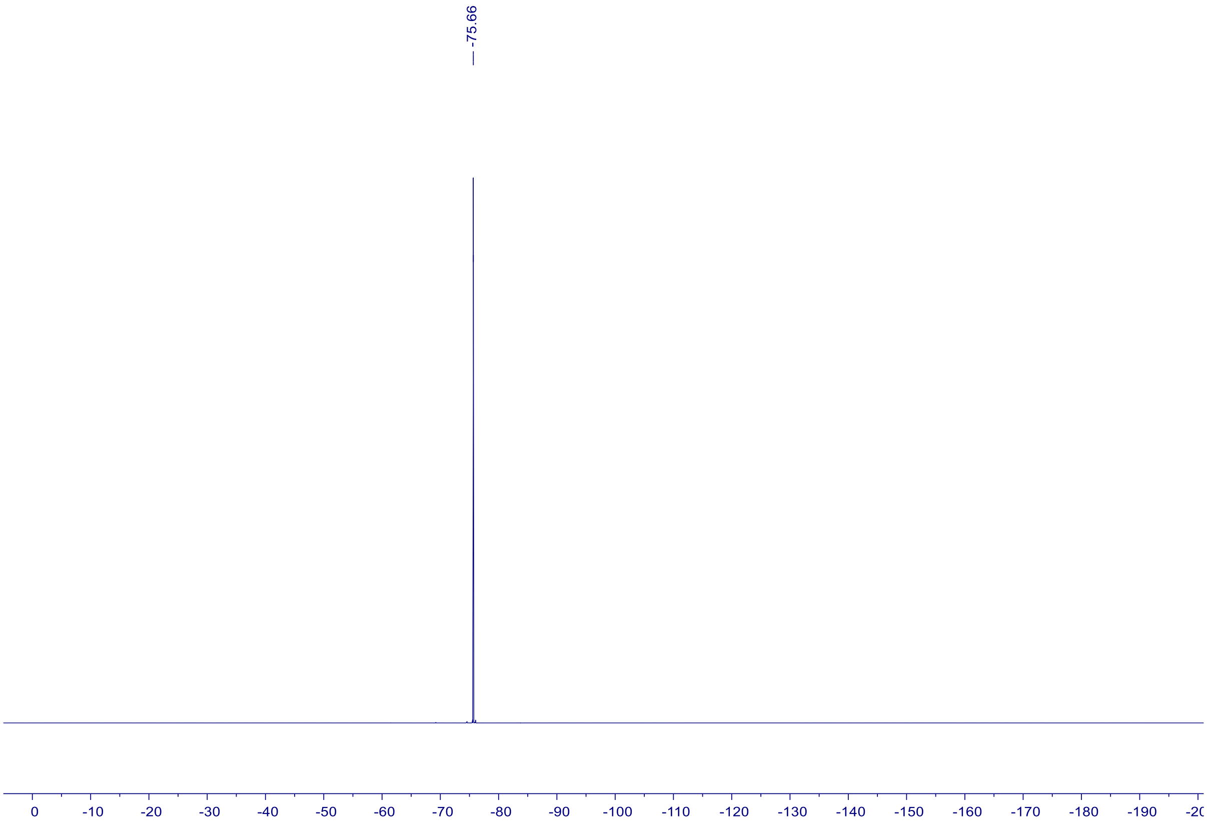

5v – ^{1}H NMR (600 MHz, $CDCl_3$)

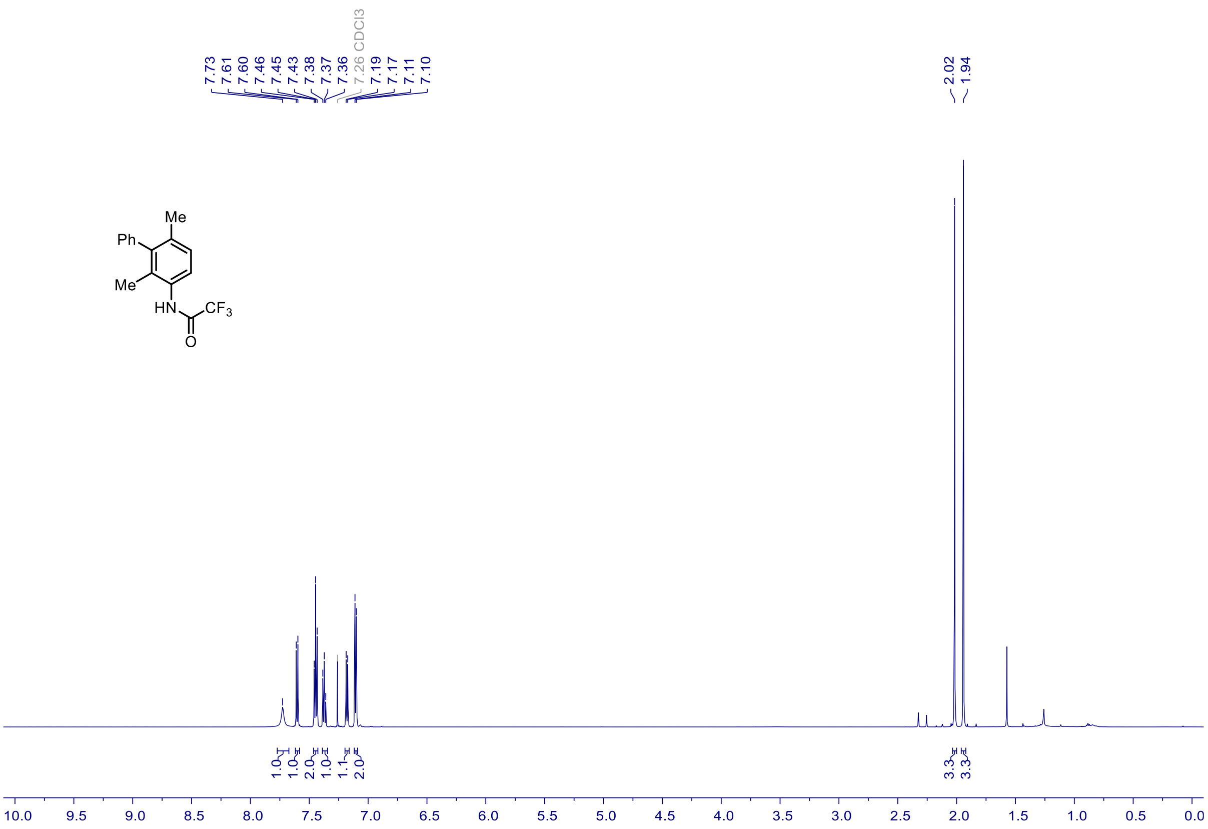

5v – ^{13}C NMR (151 MHz, $CDCl_3$)

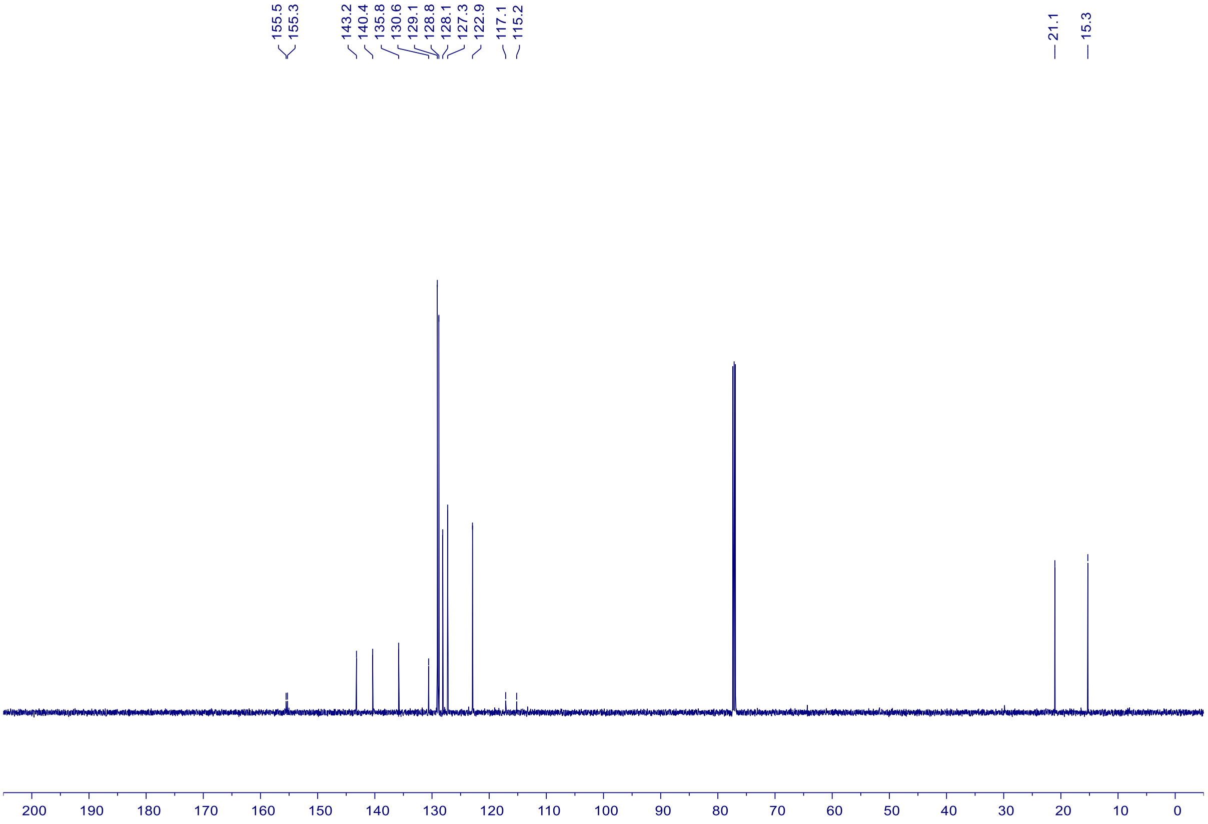

5v – ^{19}F NMR (565 MHz, $CDCl_3$)

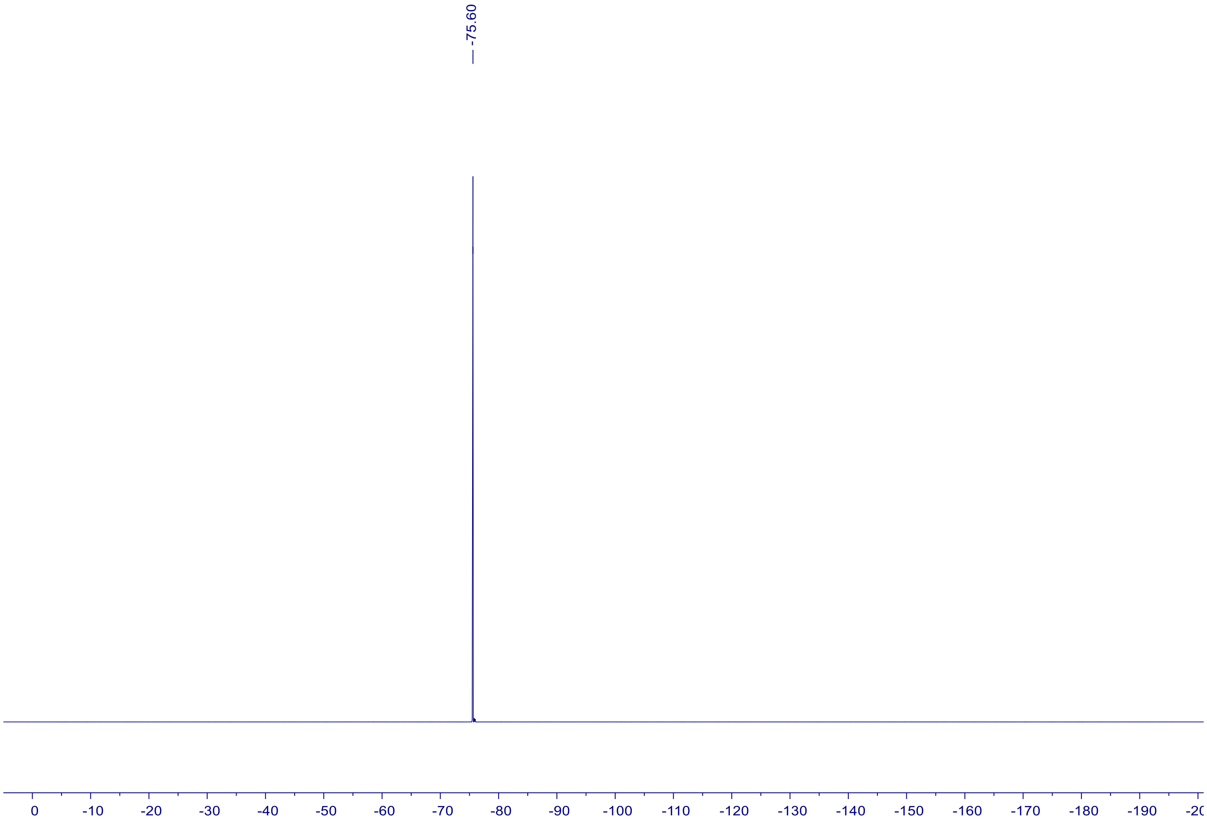

5w – ^{1}H NMR (600 MHz, $CDCl_3$)

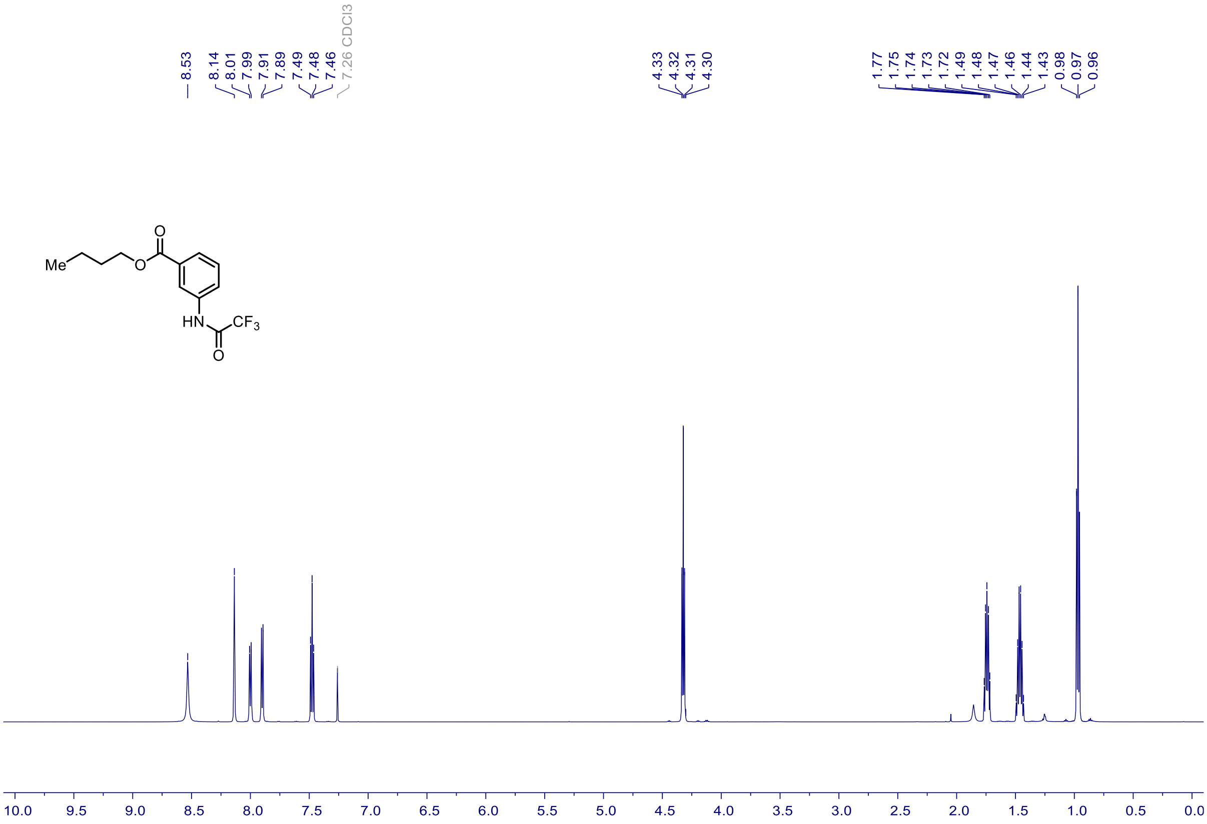

5w – ^{13}C NMR (151 MHz, $CDCl_3$)

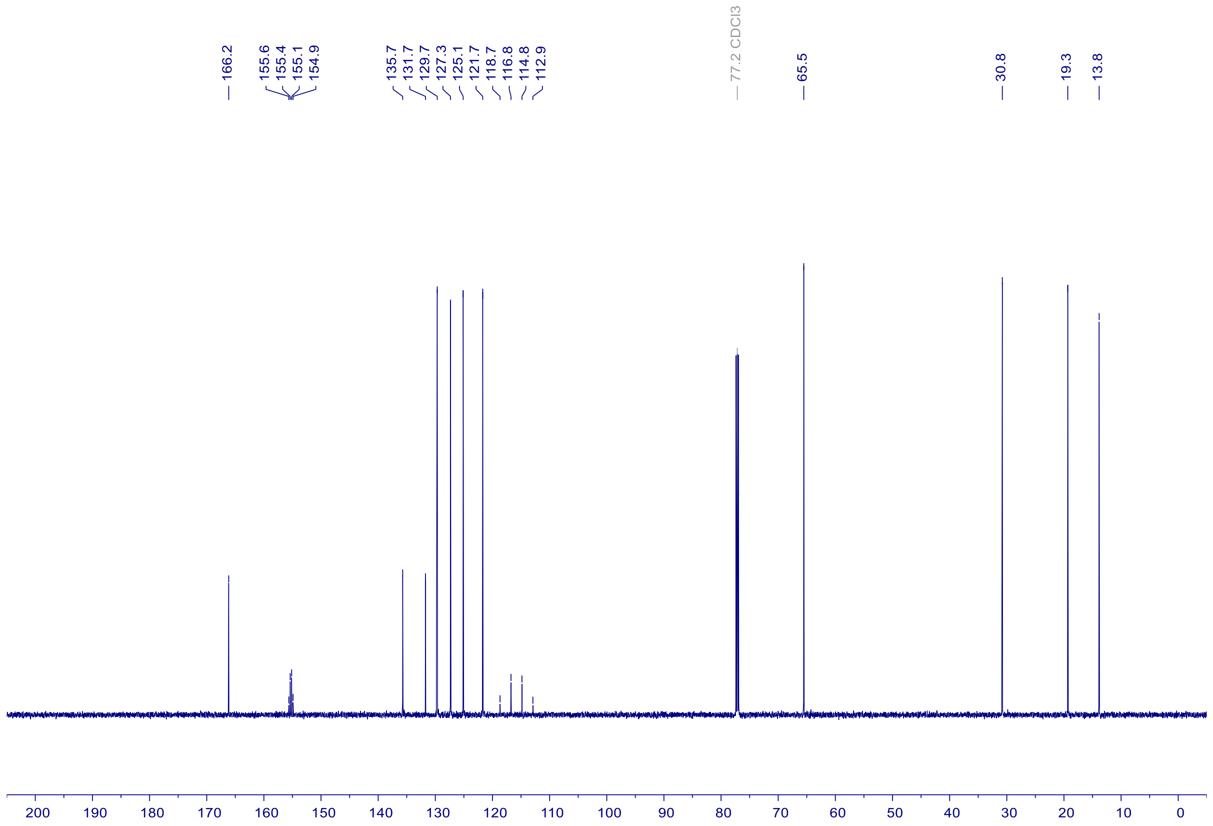

5w – ^{19}F NMR (565 MHz, $CDCl_3$)

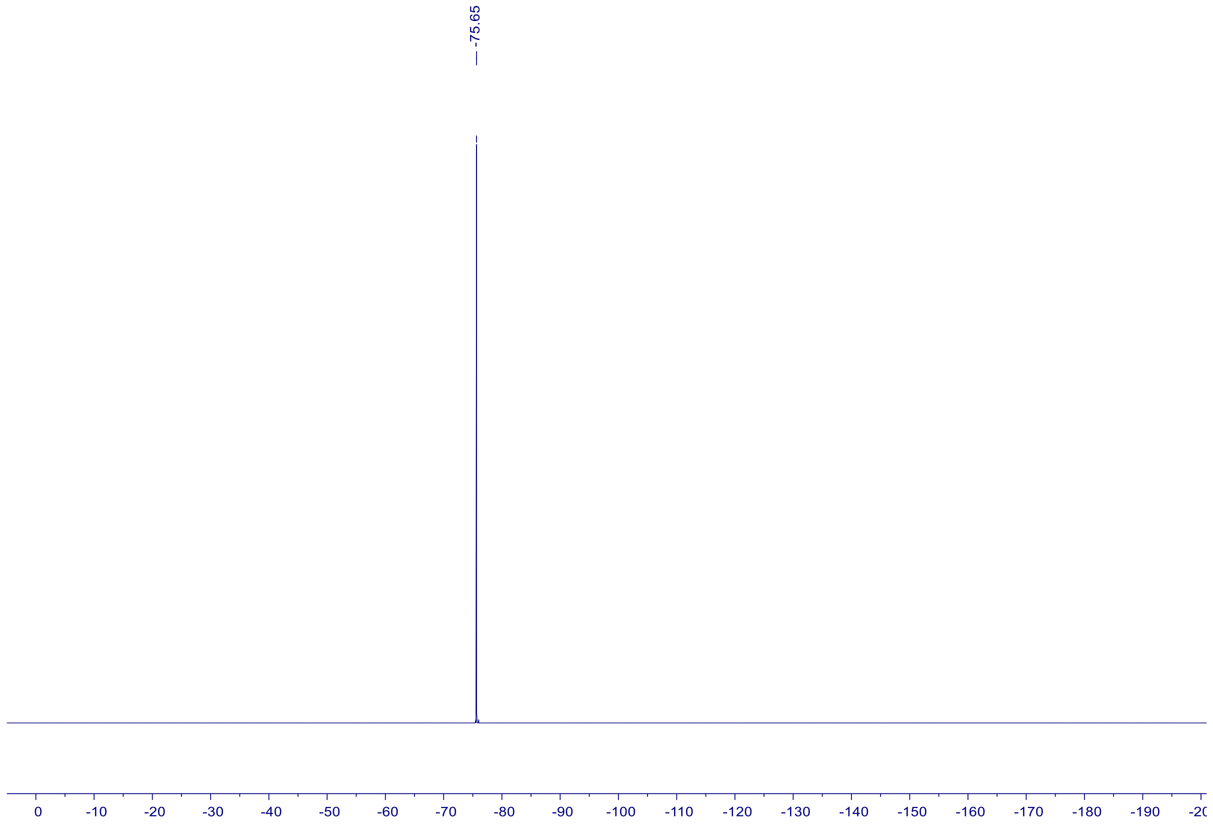

5x – ^{1}H NMR (600 MHz, $CDCl_3$)

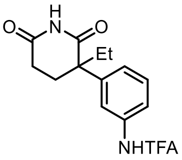

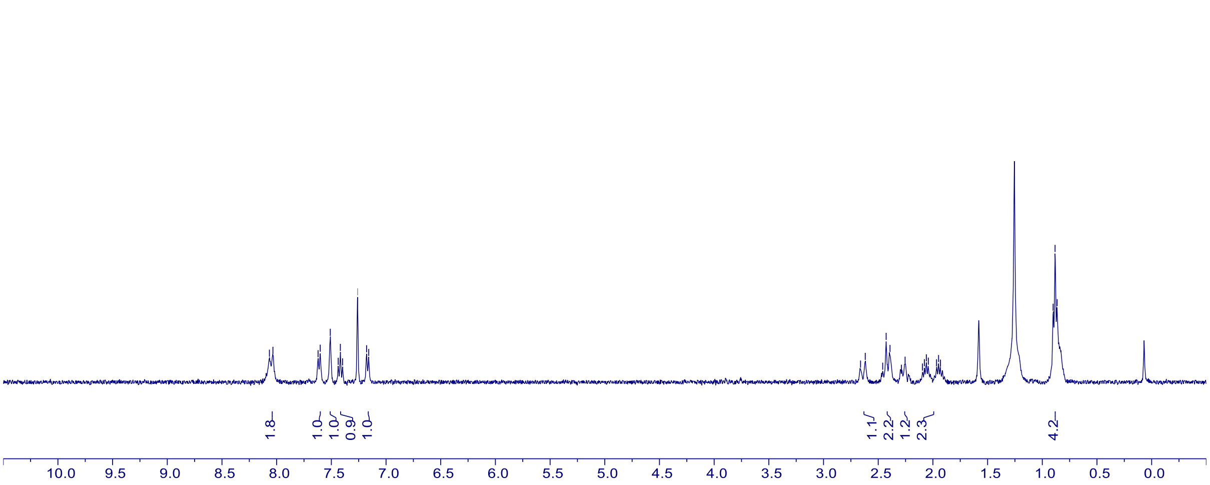

5x – ^{13}C NMR (151 MHz, $CDCl_3$)

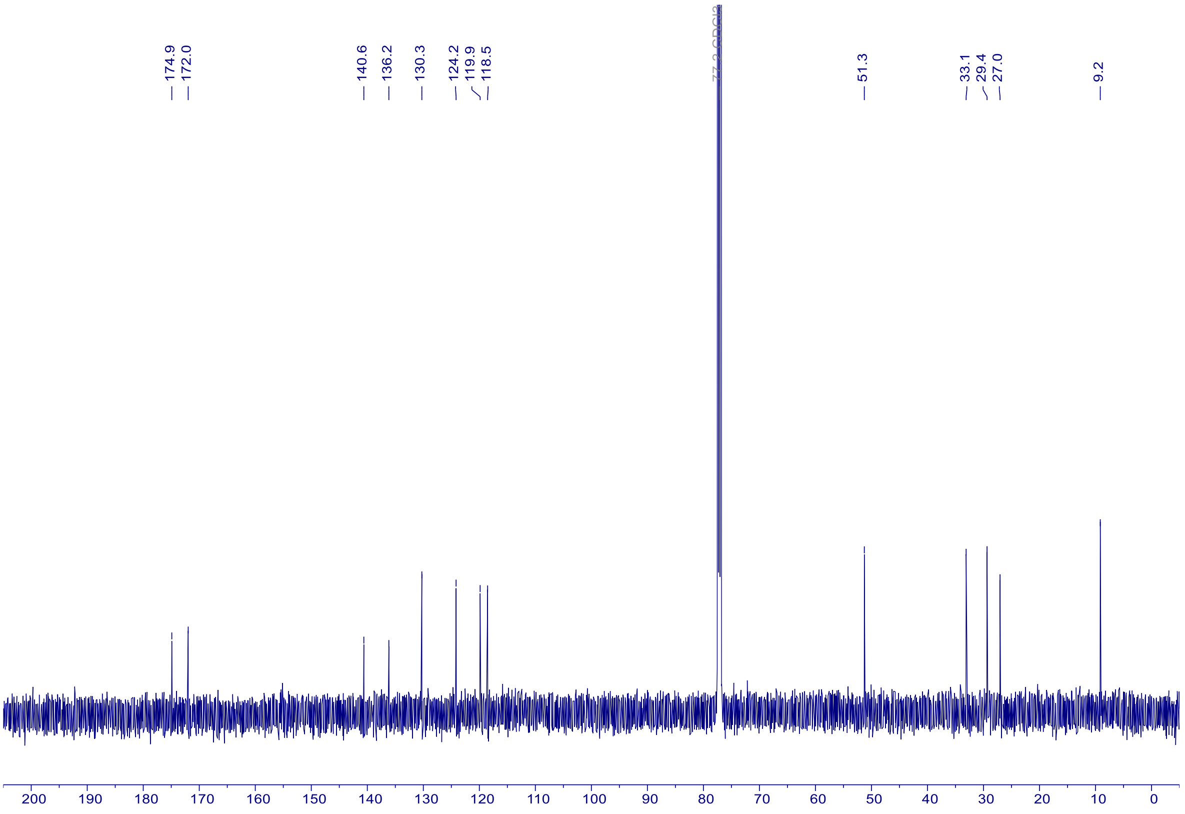

5x – ^{19}F NMR (565 MHz, $CDCl_3$)

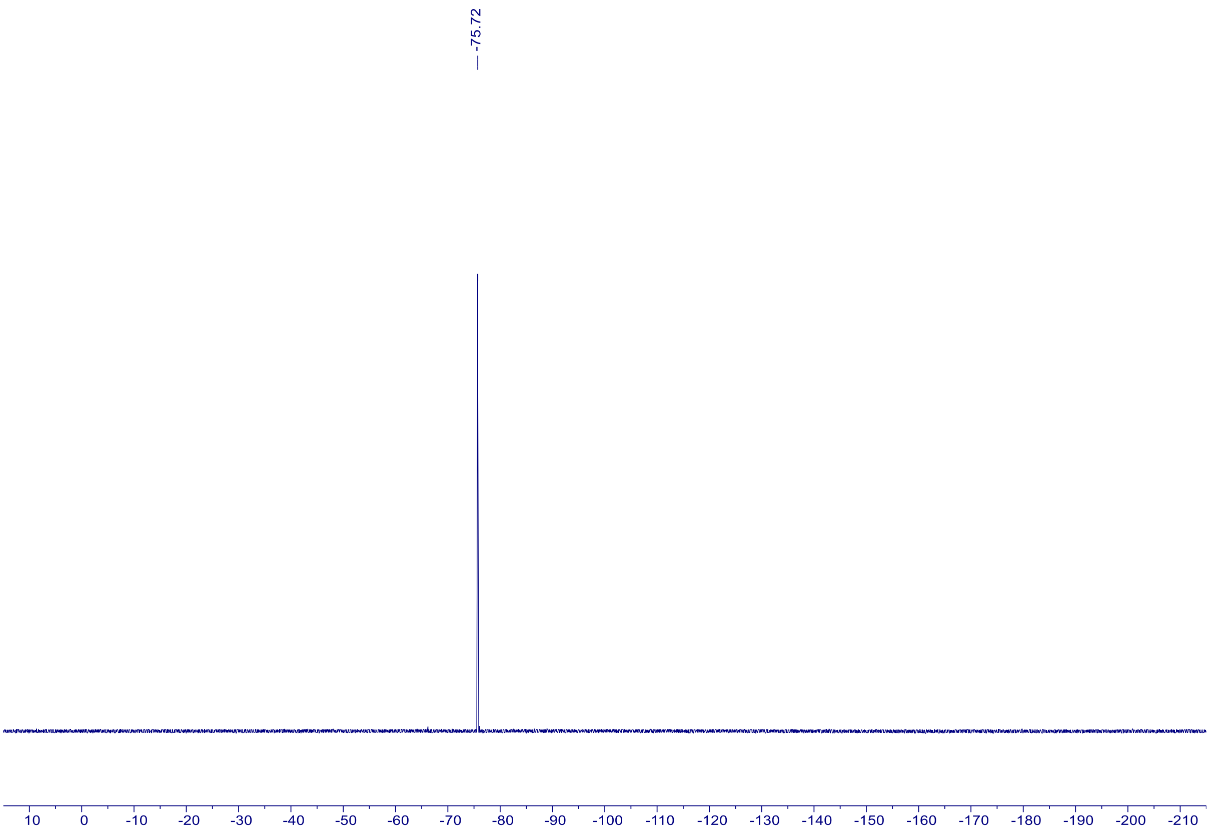

5y – ^{1}H NMR (600 MHz, $CDCl_3$)

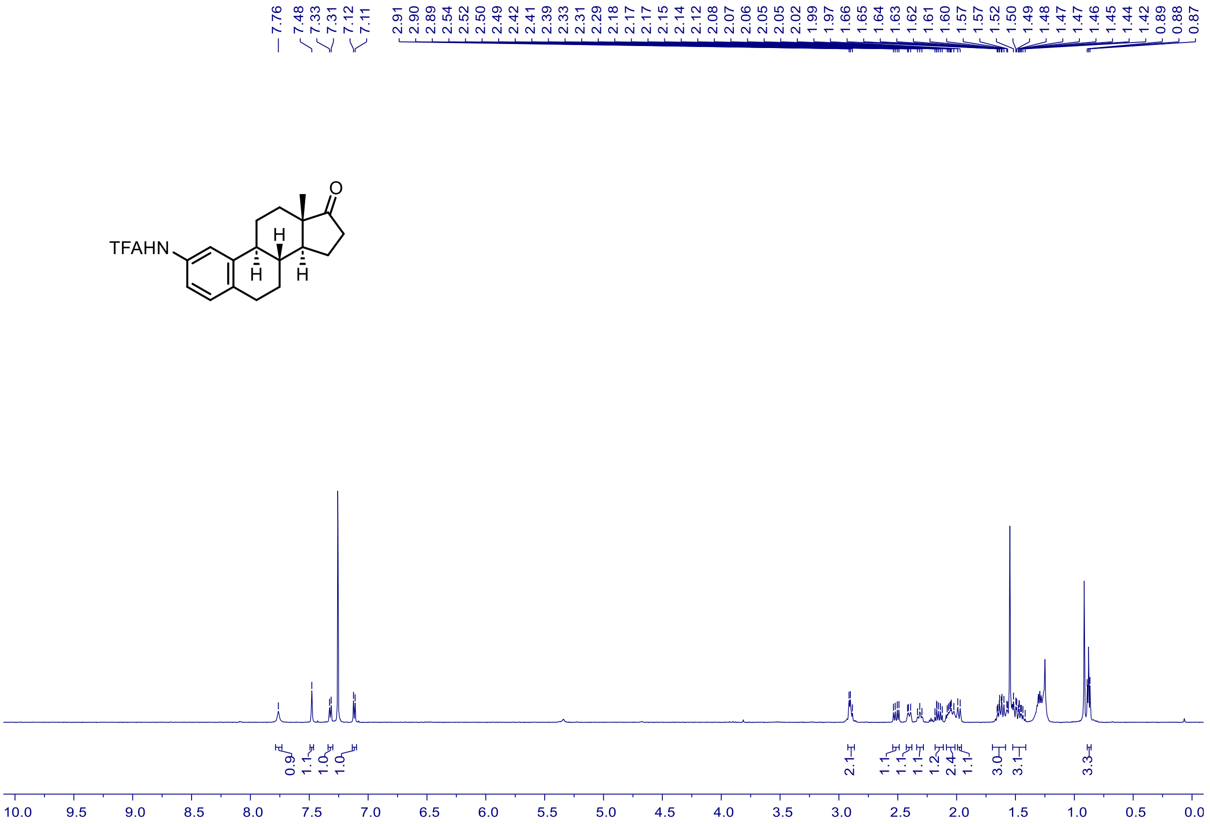

5y – ^{13}C NMR (151 MHz, $CDCl_3$)

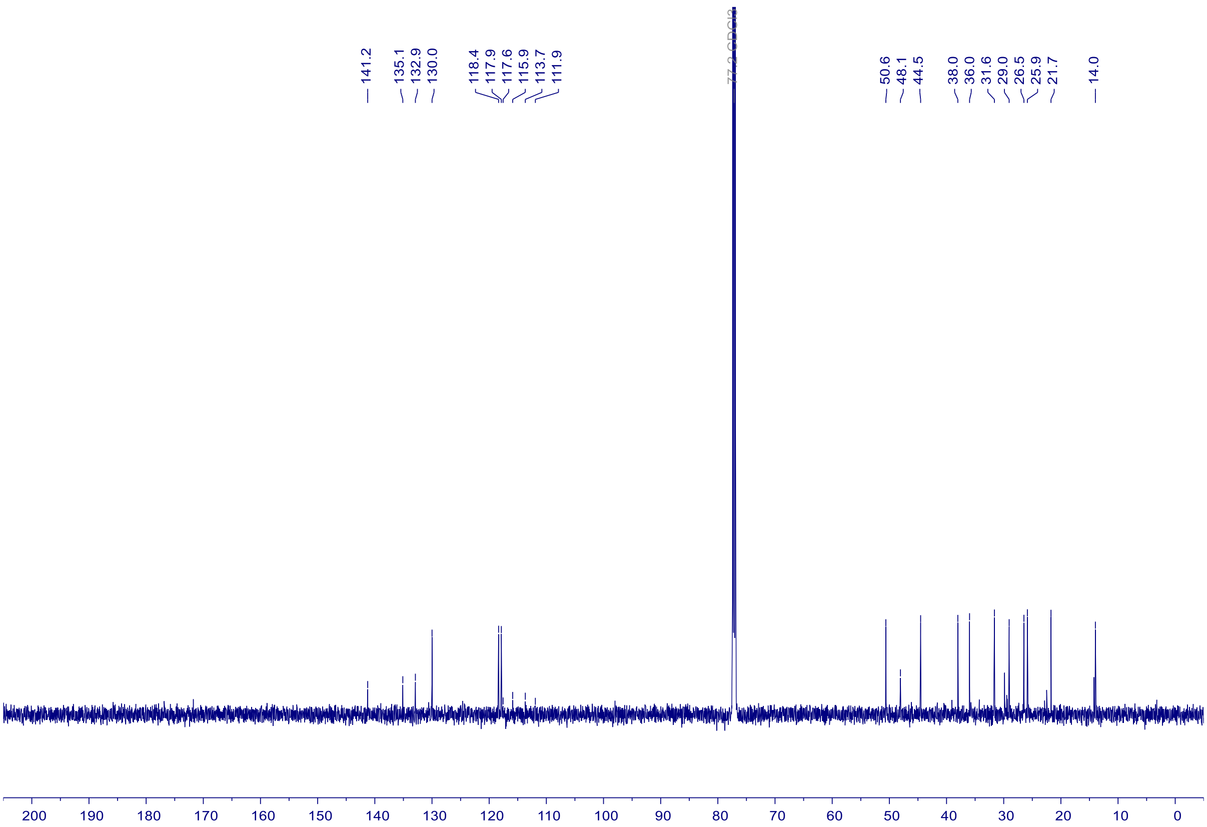

5y – ^{19}F NMR (565 MHz, $CDCl_3$)

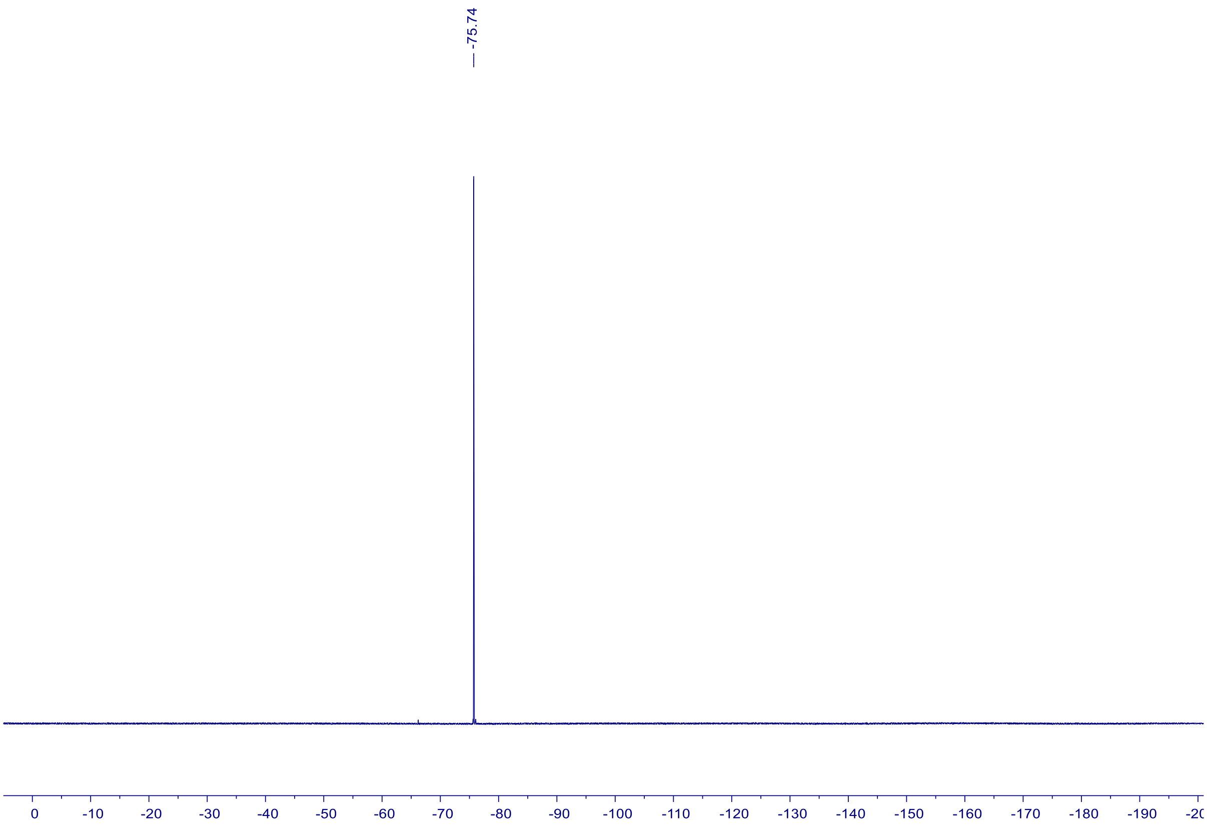

4qh – ^{1}H NMR (600 MHz, $CDCl_3$)

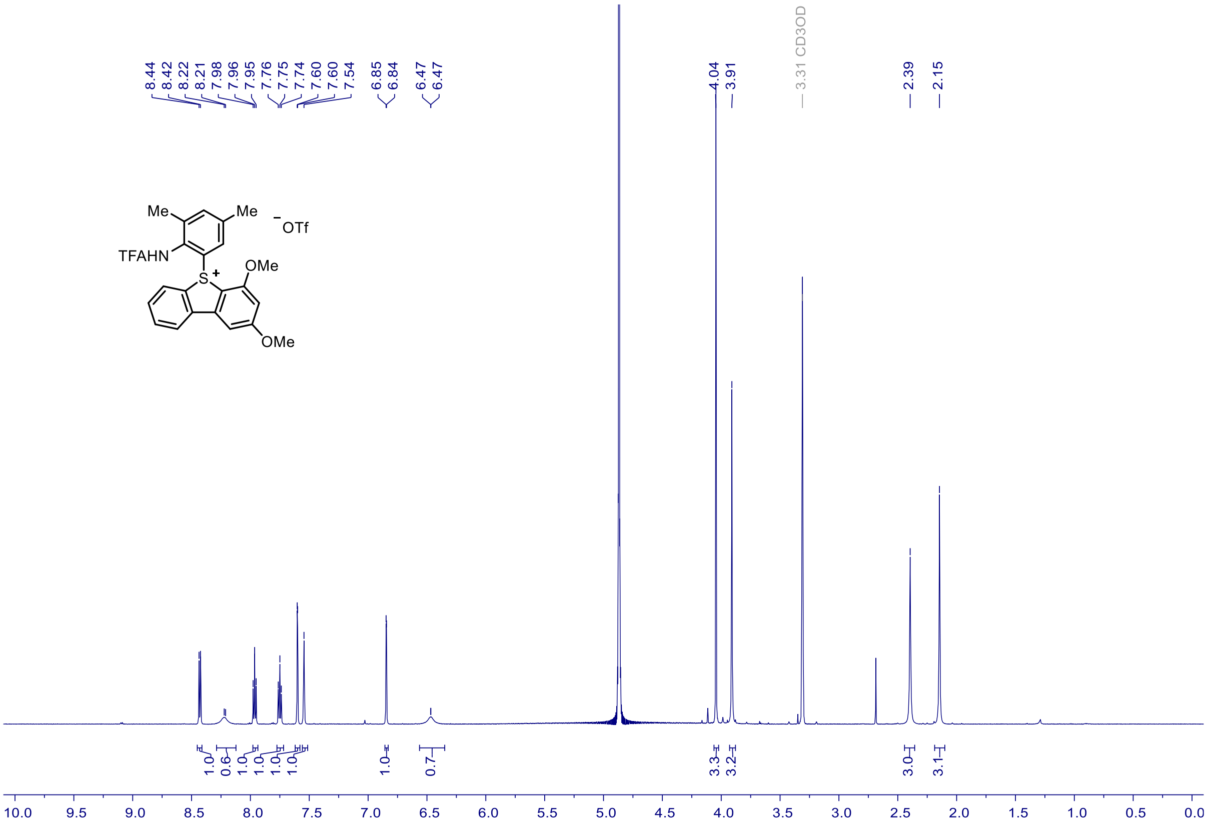

4qh – ^{13}C NMR (151 MHz, $CDCl_3$)

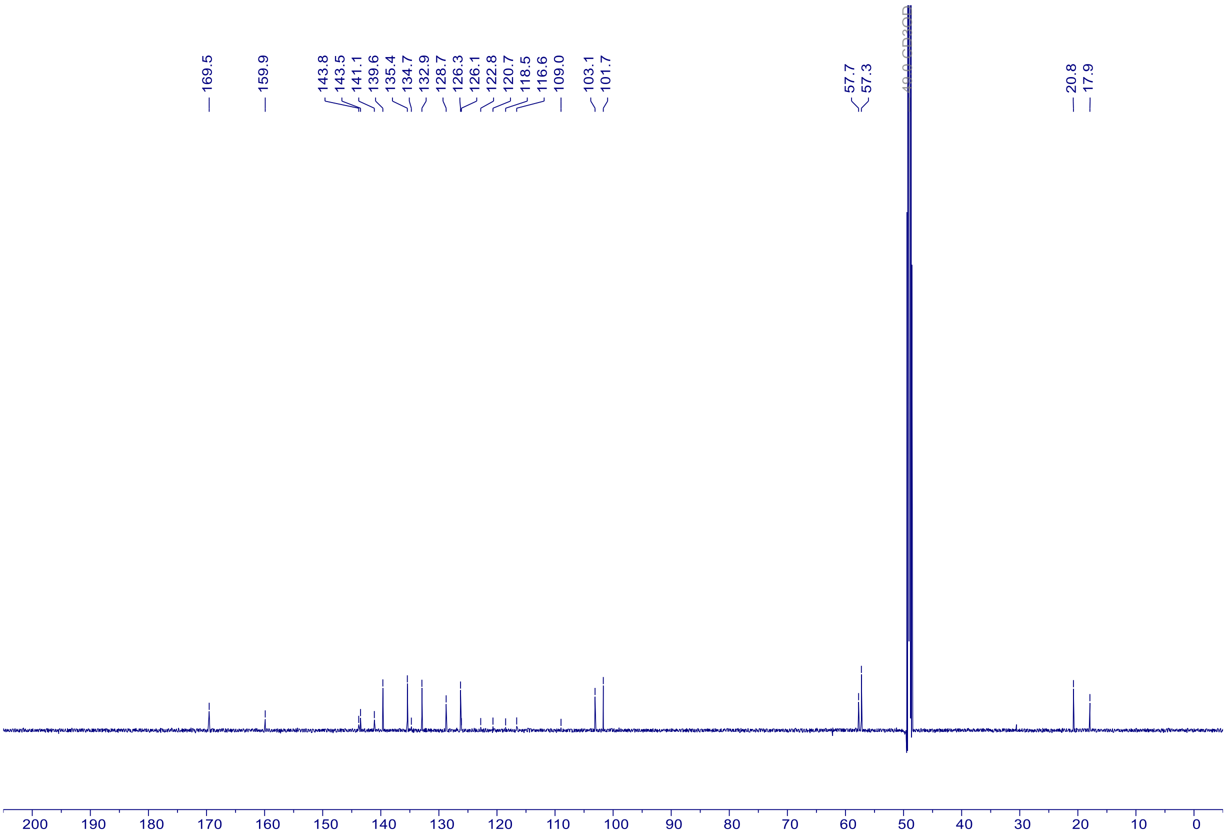

4qh – ^{19}F NMR (565 MHz, $CDCl_3$)

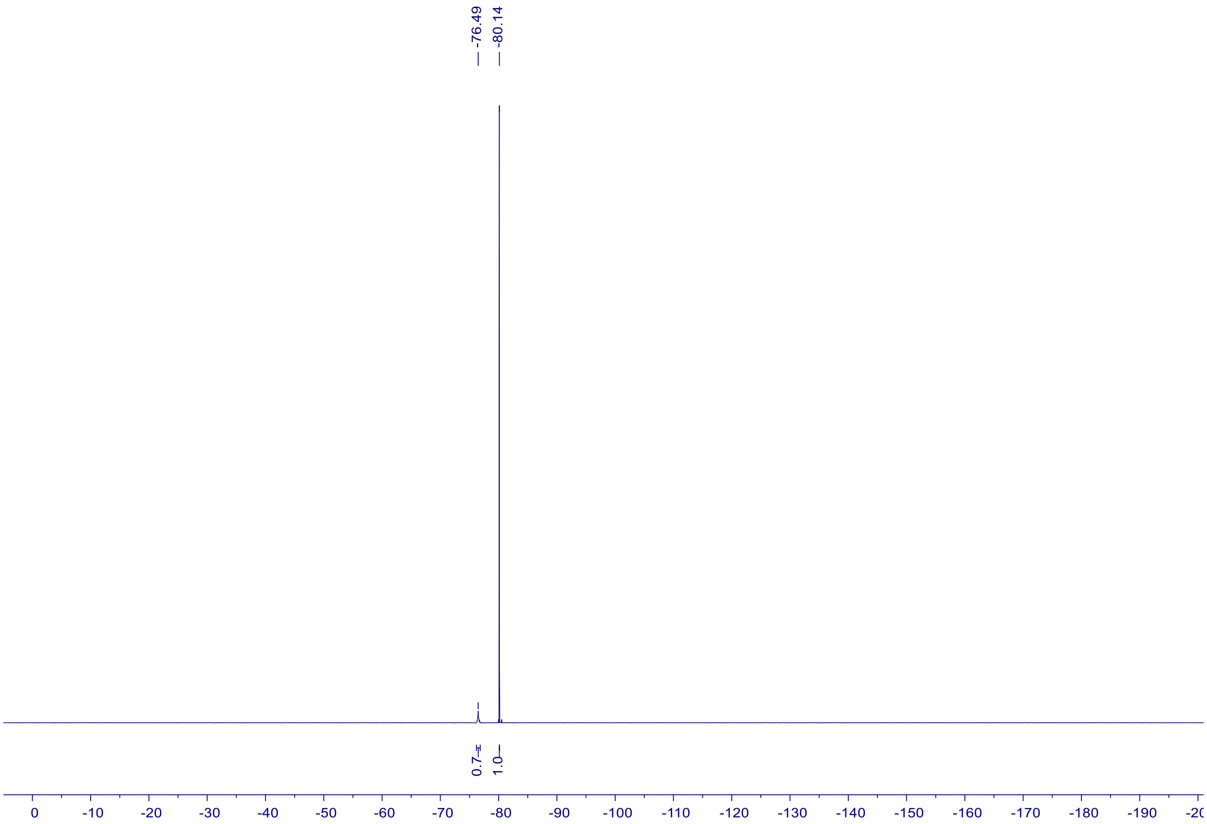

6a– ^{1}H NMR (600 MHz, $CDCl_3$)

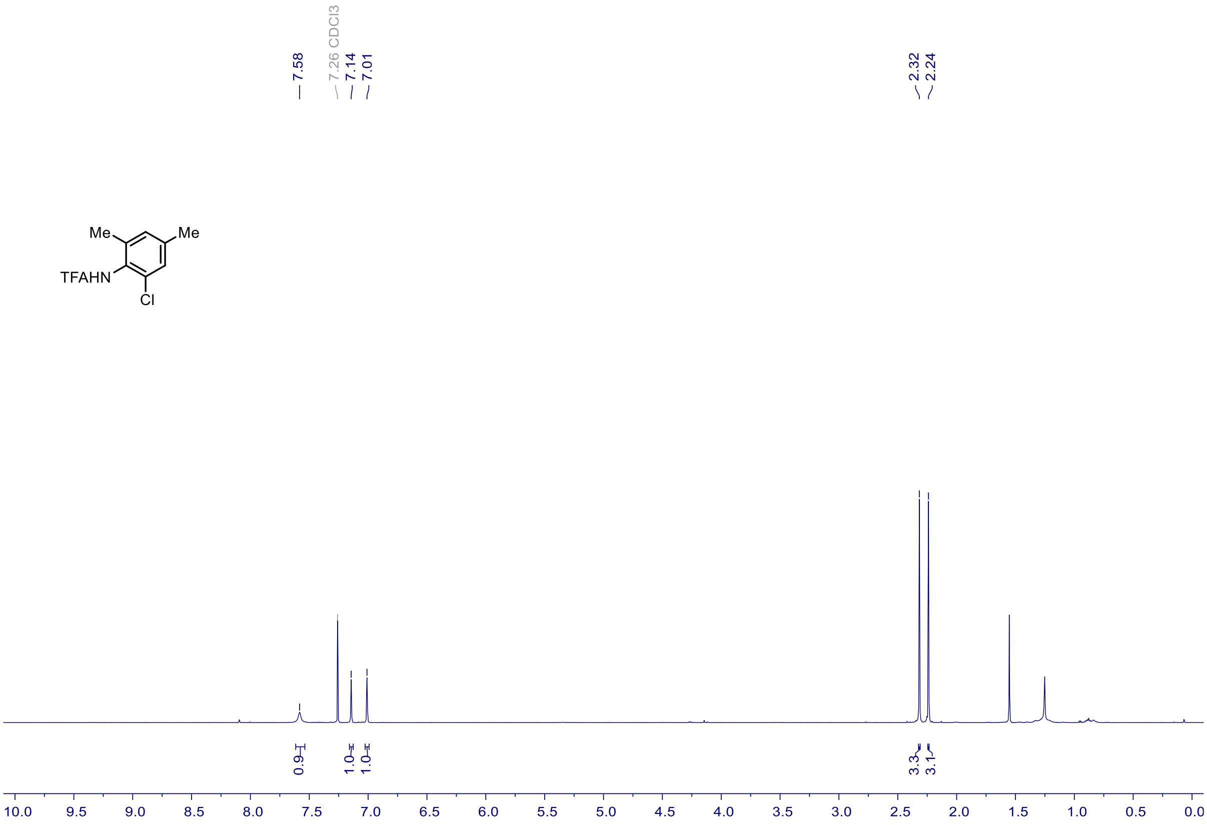

6a – ^{13}C NMR (151 MHz, $CDCl_3$)

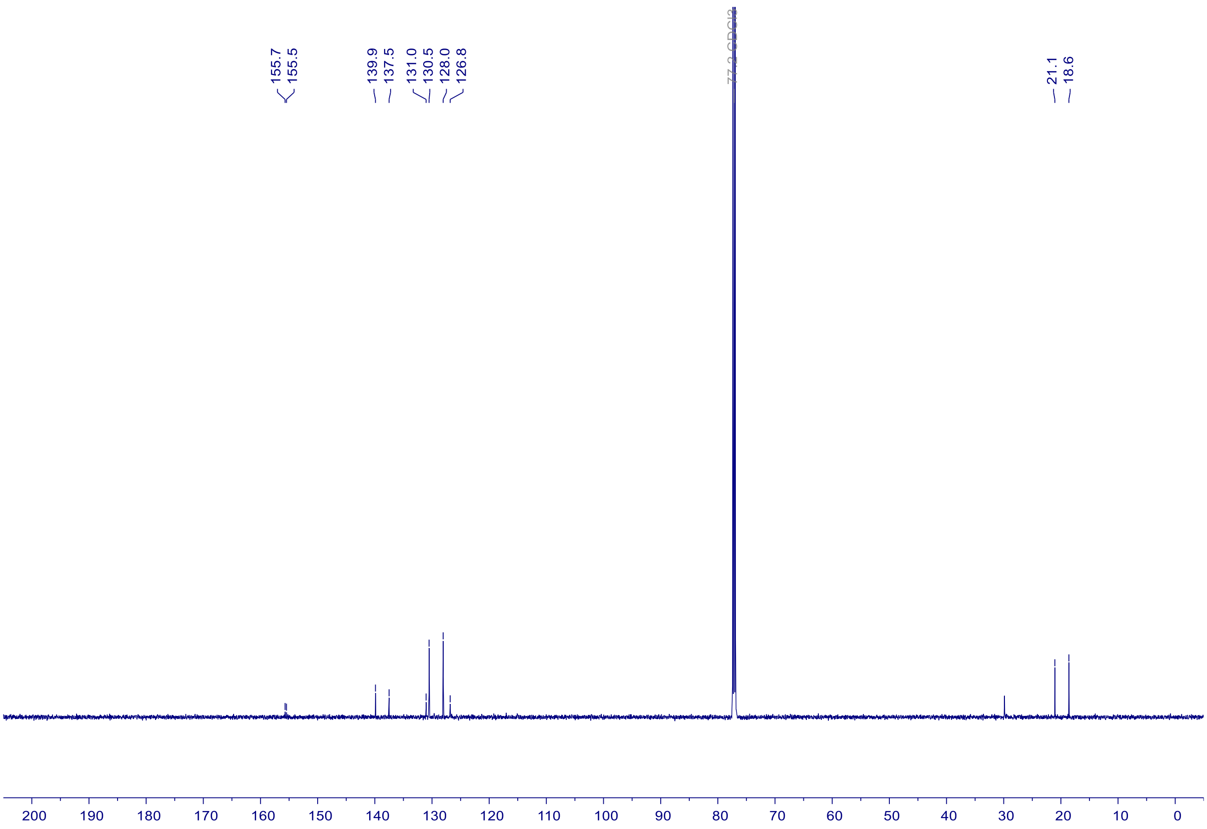

6a – ^{19}F NMR (565 MHz, $CDCl_3$)

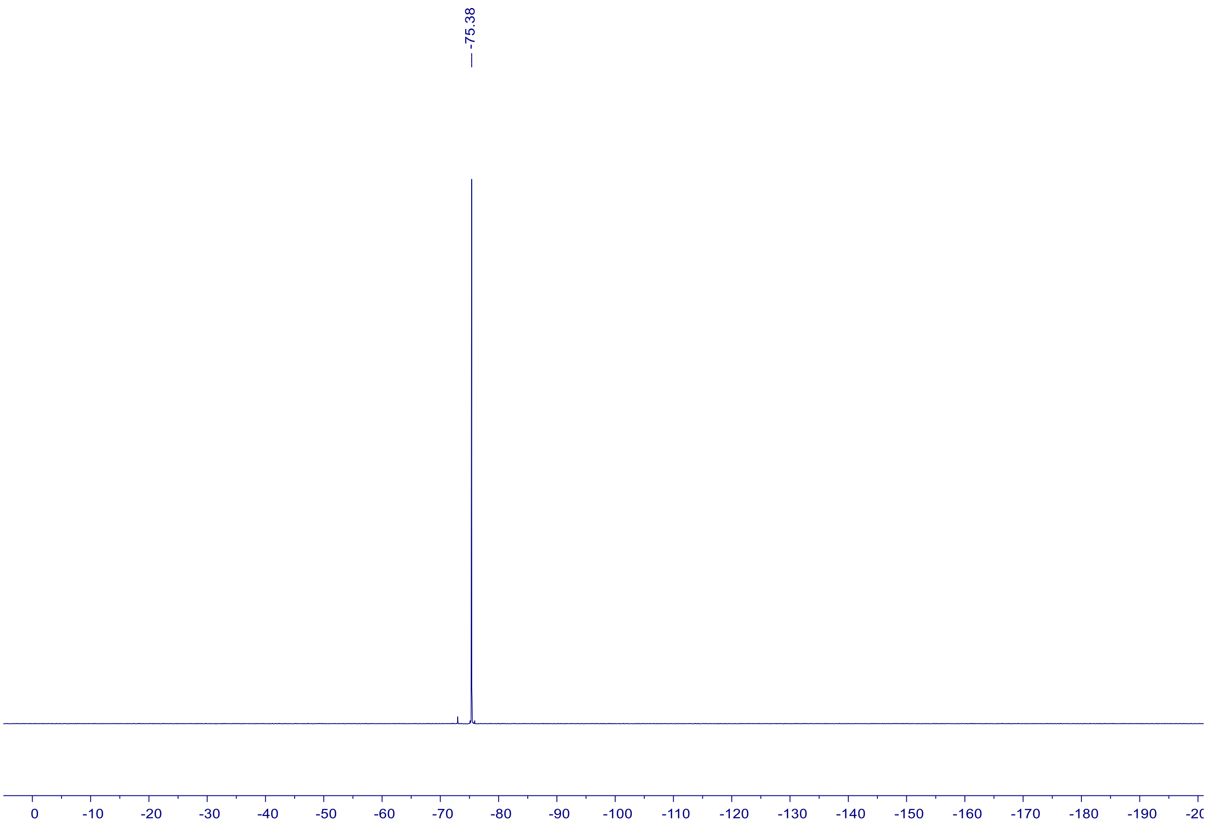

6b – ^{1}H NMR (600 MHz, $CDCl_3$)

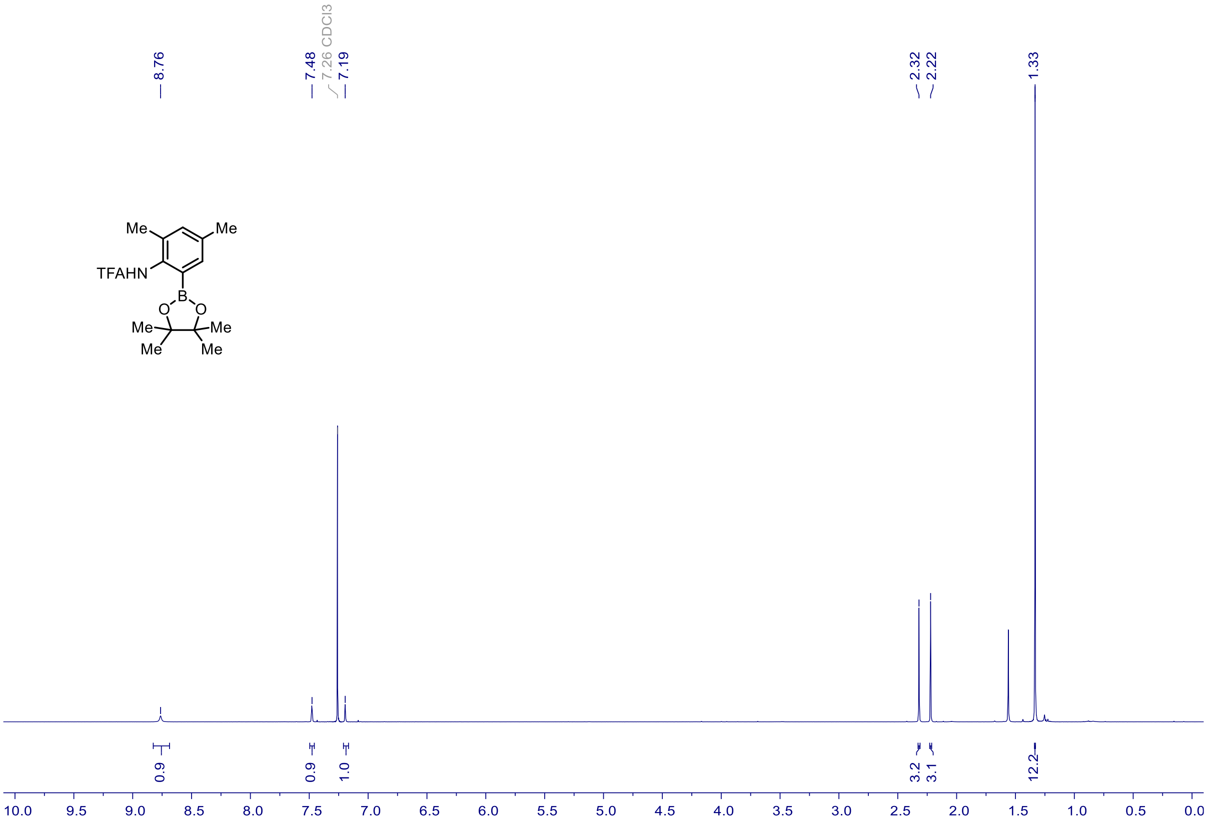

6b – ^{13}C NMR (151 MHz, $CDCl_3$)

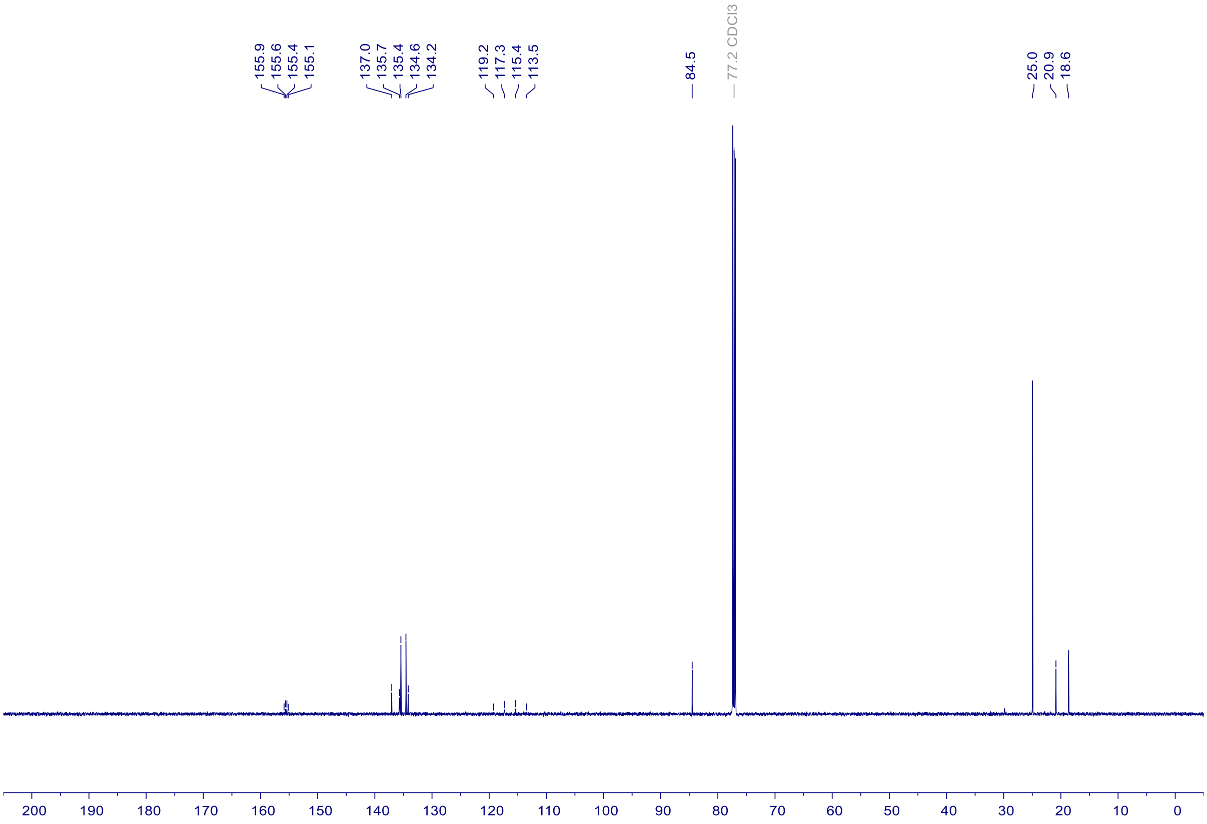

6b – ^{19}F NMR (565 MHz, CDCl$_3$)

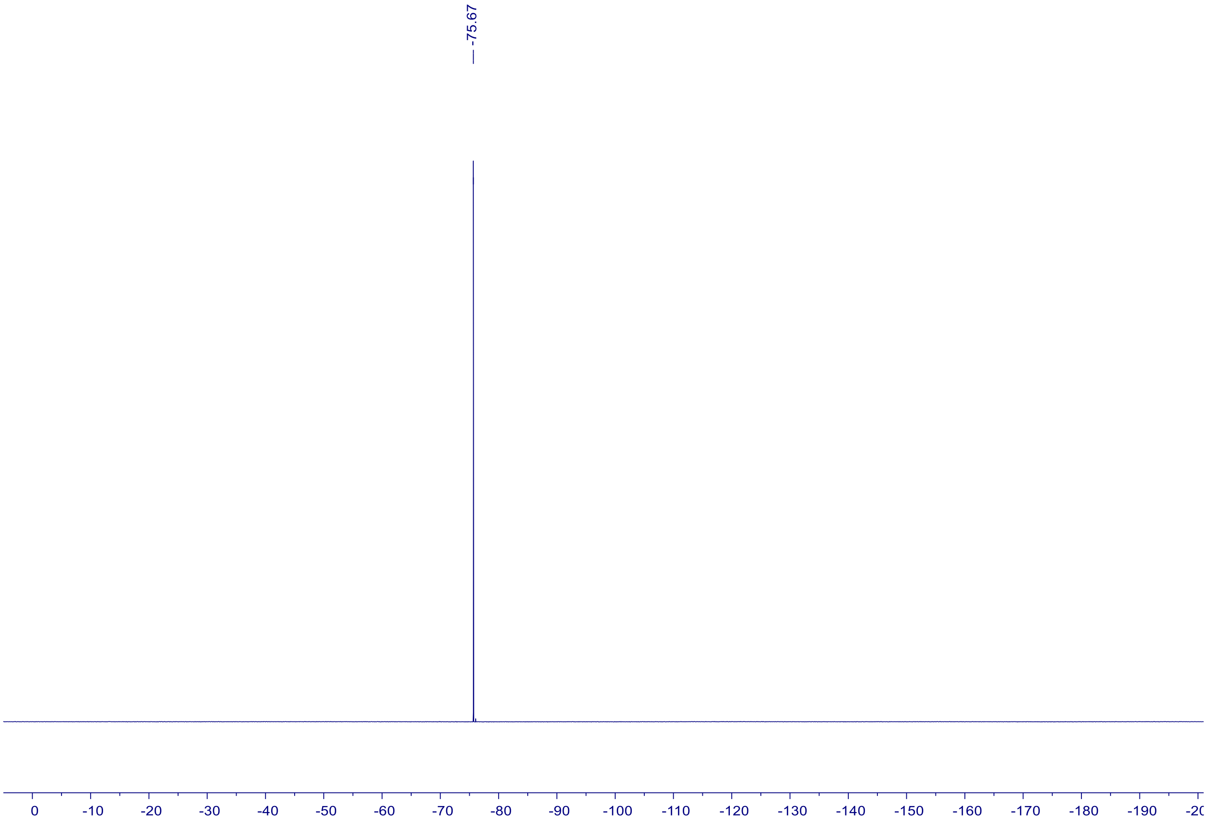

6c – ^{1}H NMR (600 MHz, CDCl$_3$)

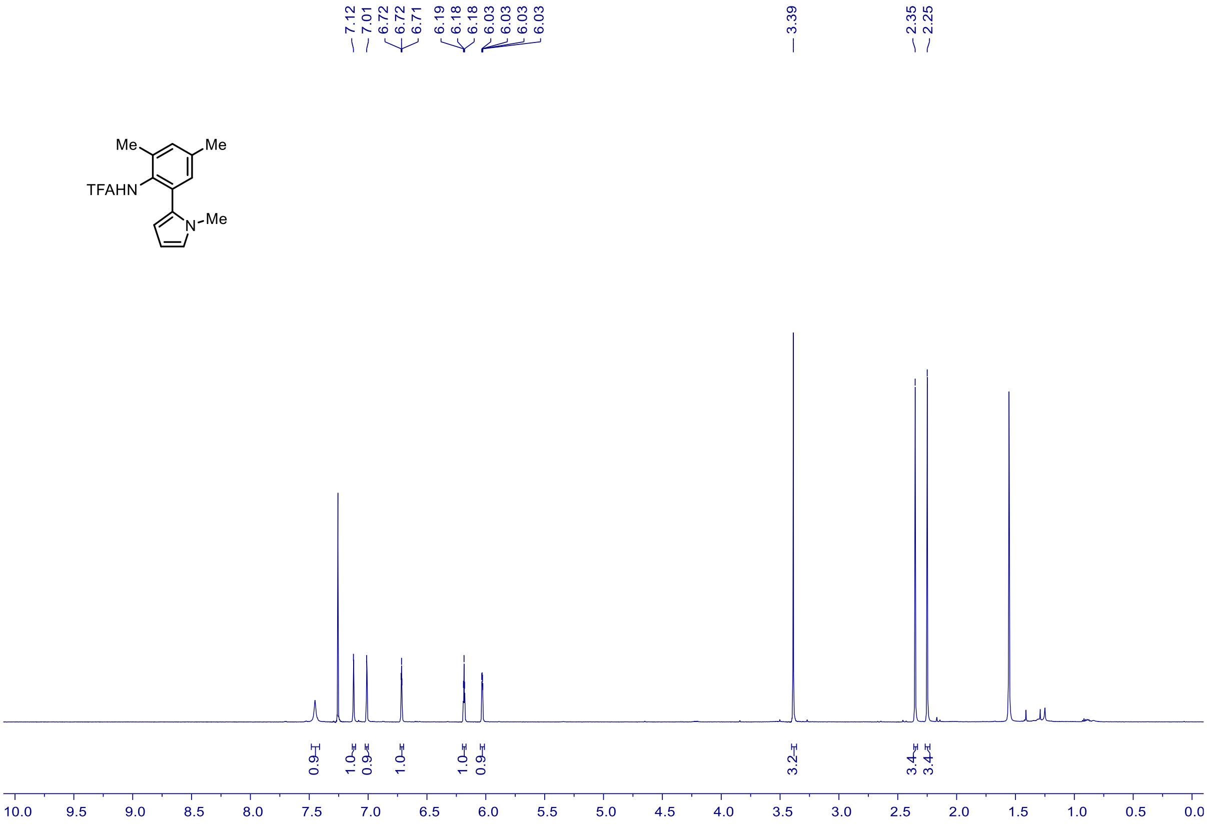

6c – ^{13}C NMR (151 MHz, CDCl$_3$)

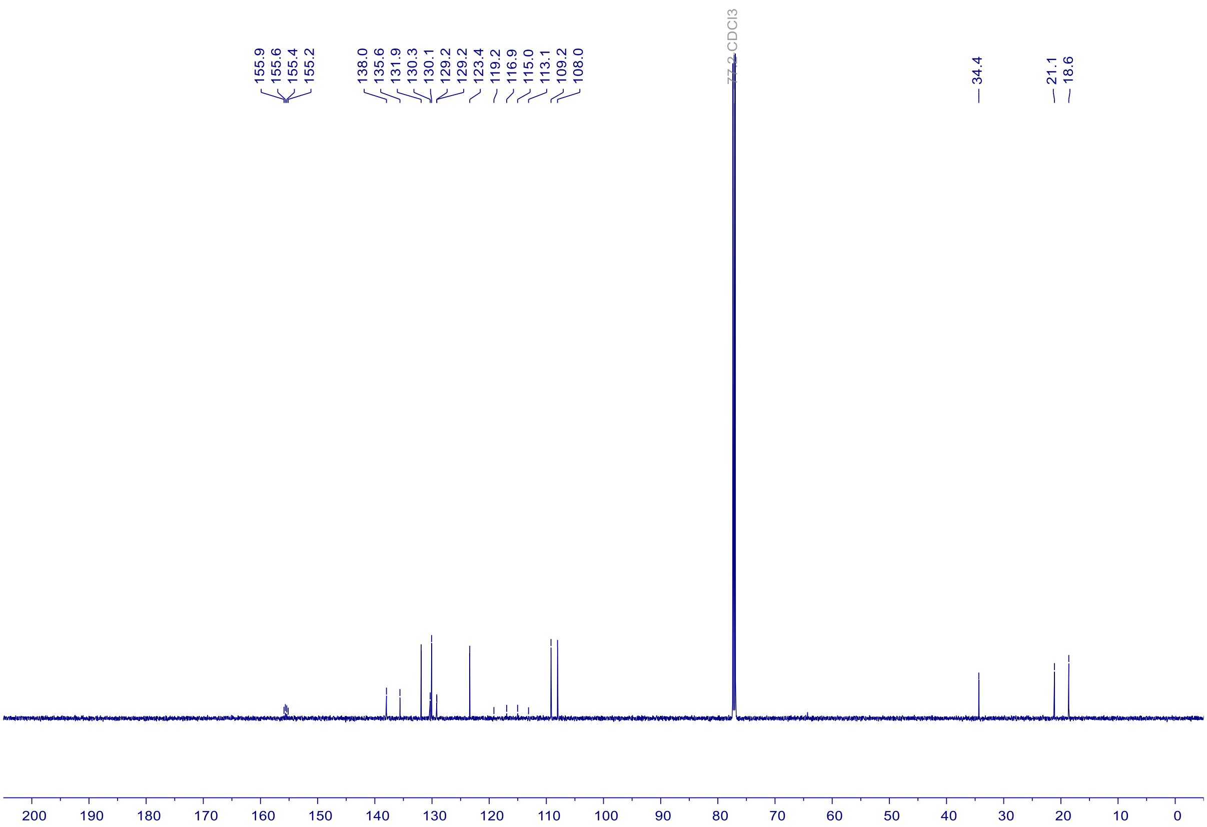

6c – ^{19}F NMR (565 MHz, CDCl$_3$)

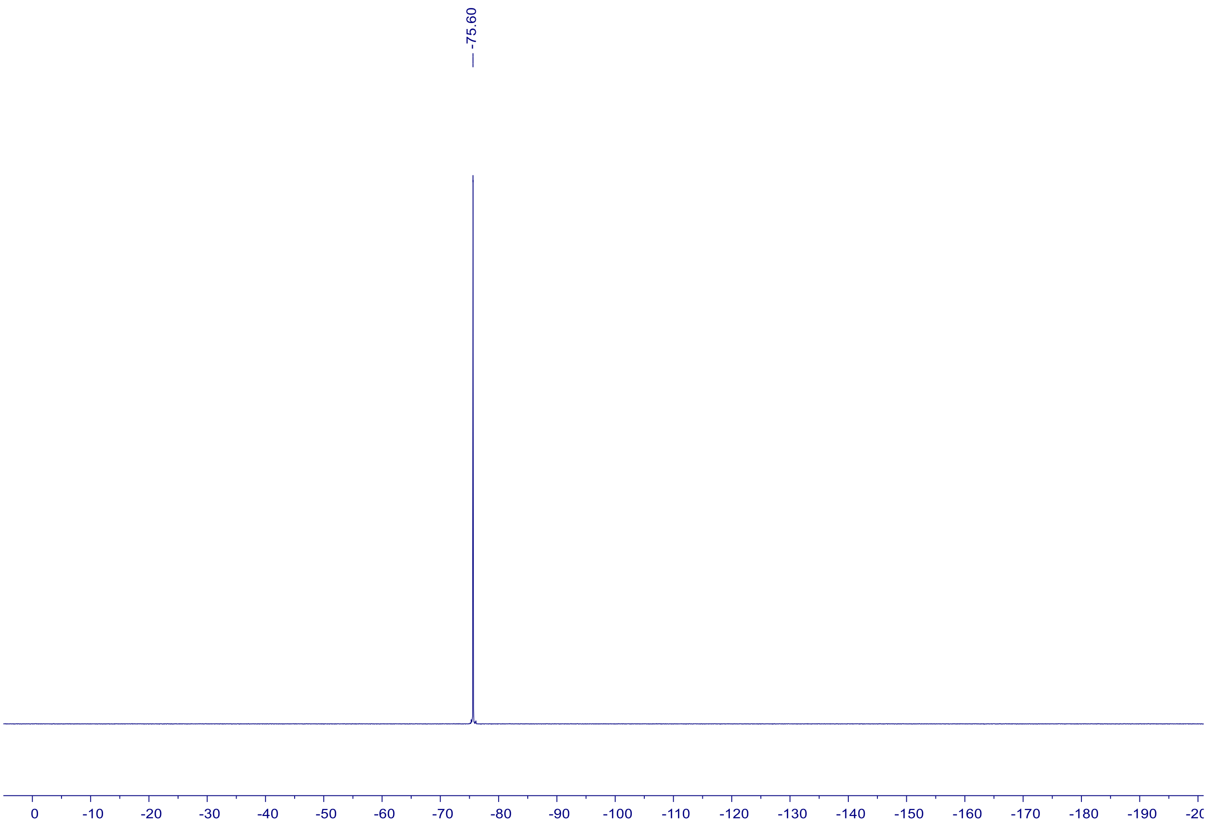

6d – ^{1}H NMR (600 MHz, $CDCl_3$)

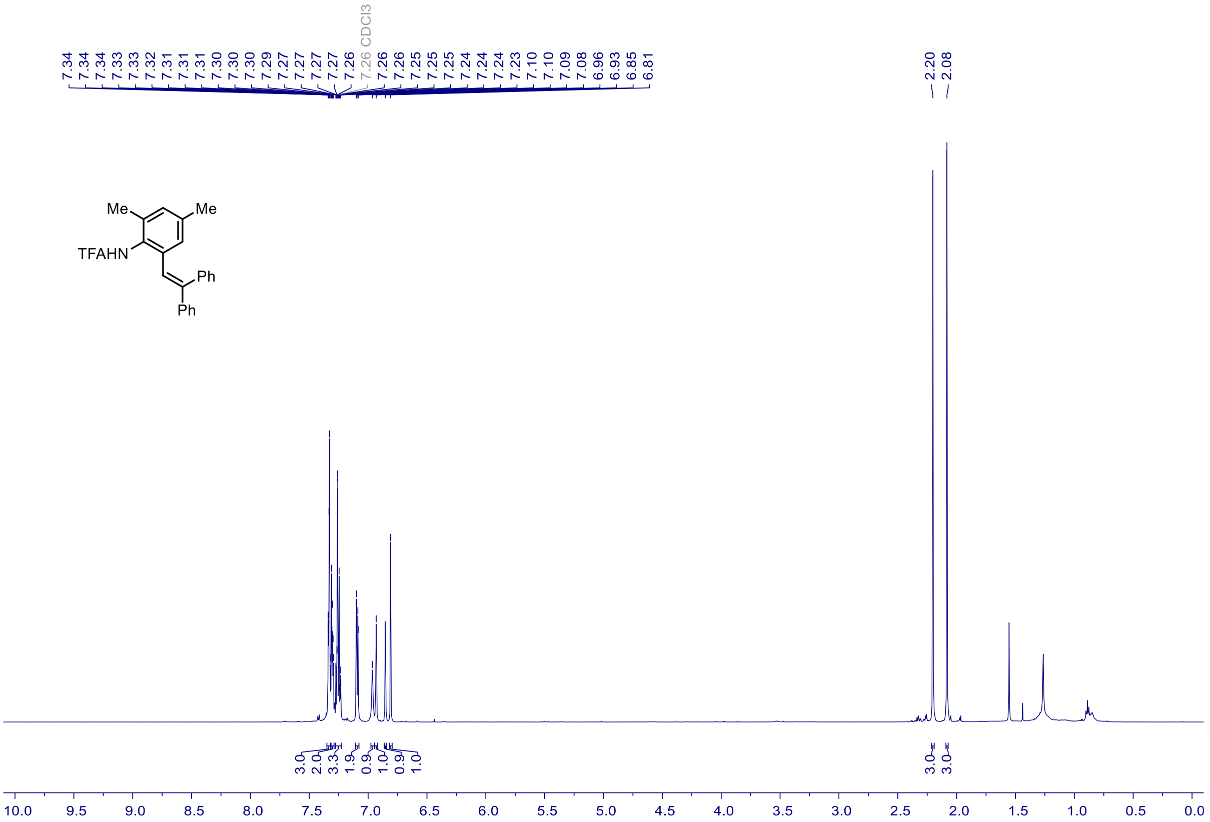

6d – ^{13}C NMR (151 MHz, $CDCl_3$)

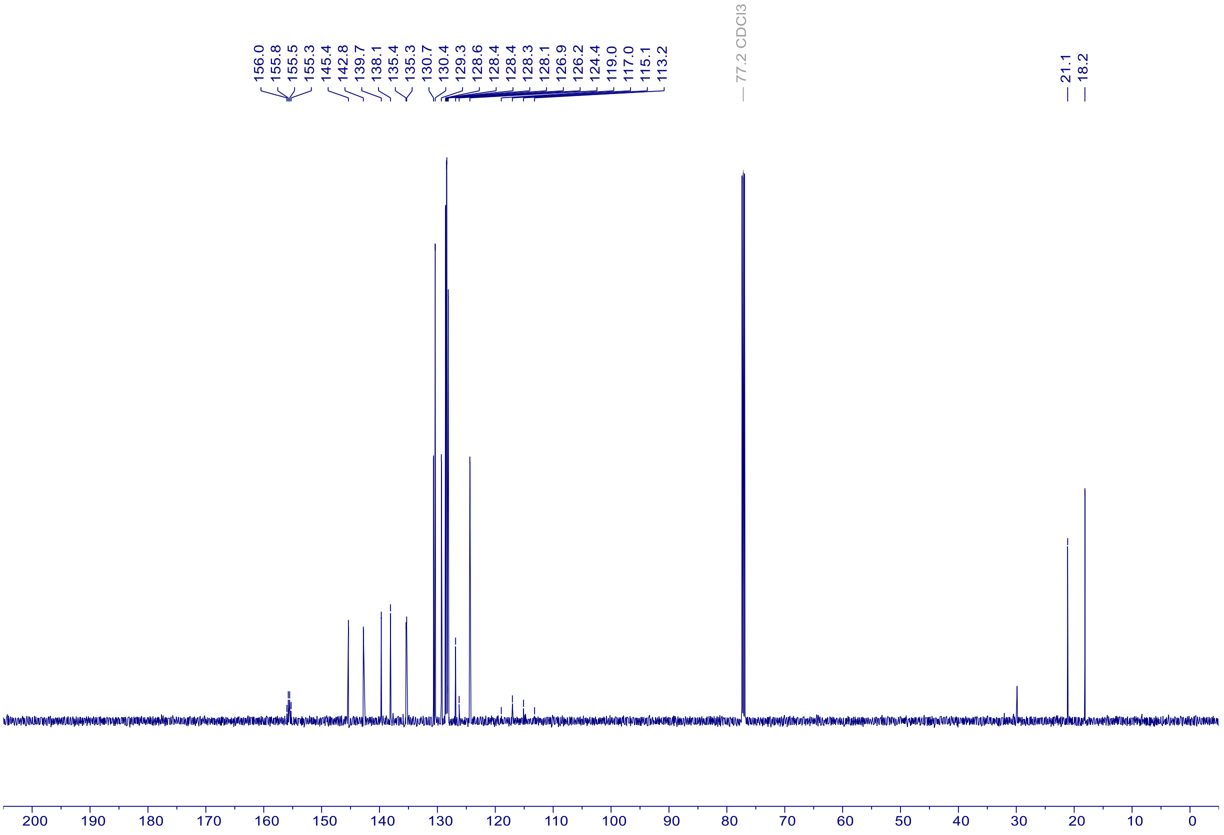

6d – ^{19}F NMR (565 MHz, $CDCl_3$)

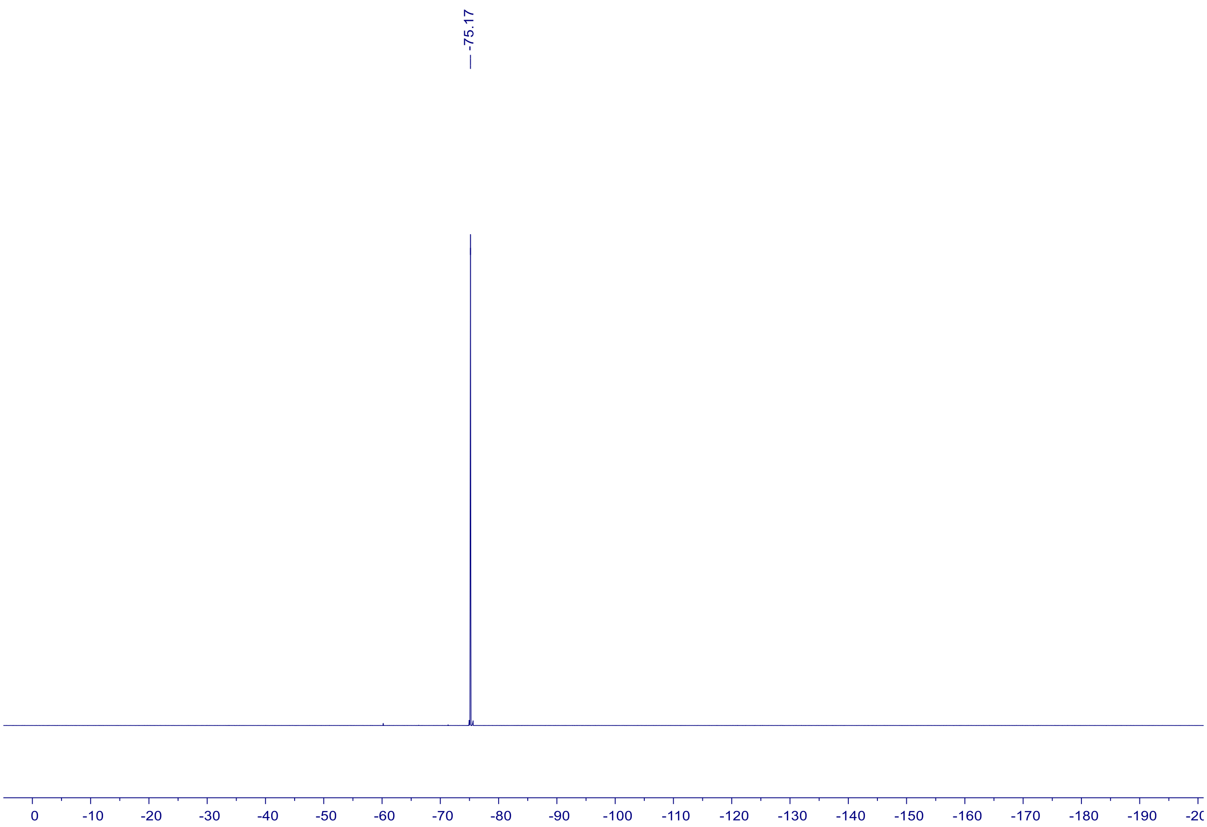

6e – ^{1}H NMR (600 MHz, $CDCl_3$)

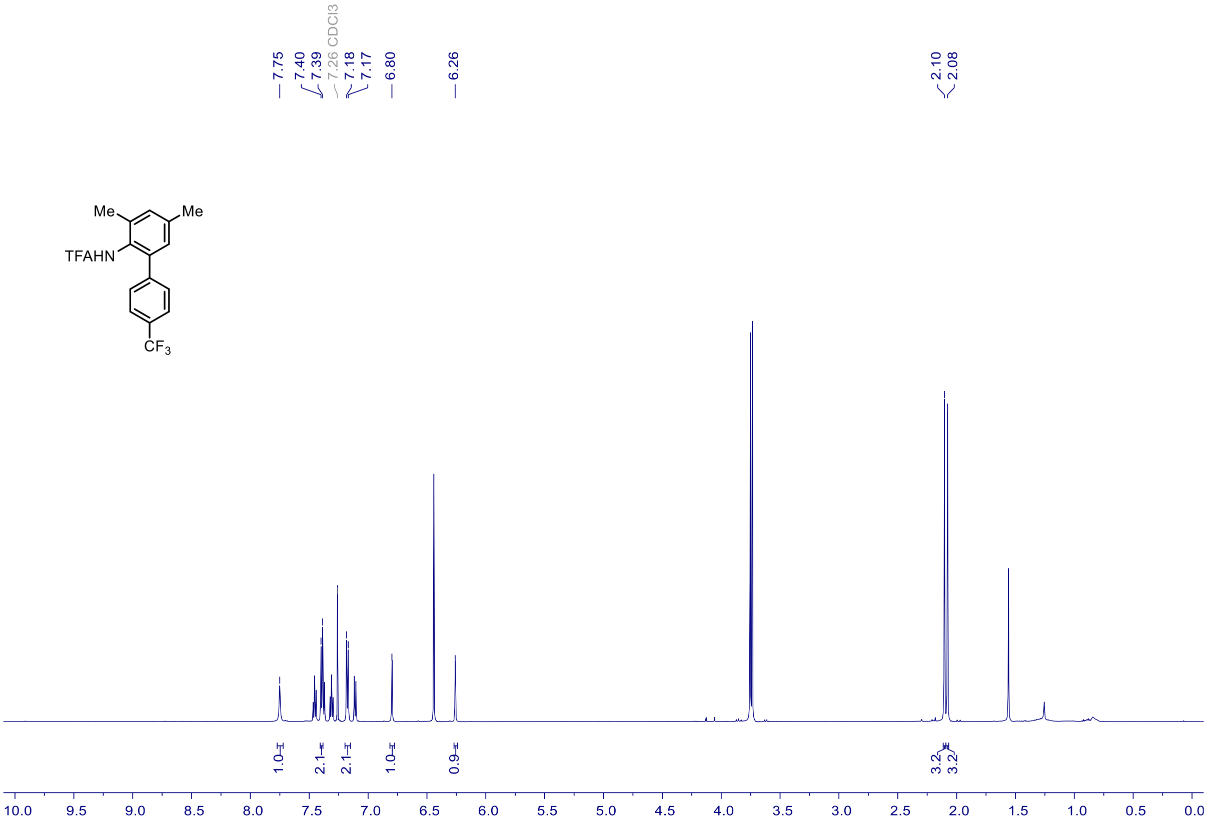

6e – ^{13}C NMR (151 MHz, $CDCl_3$)

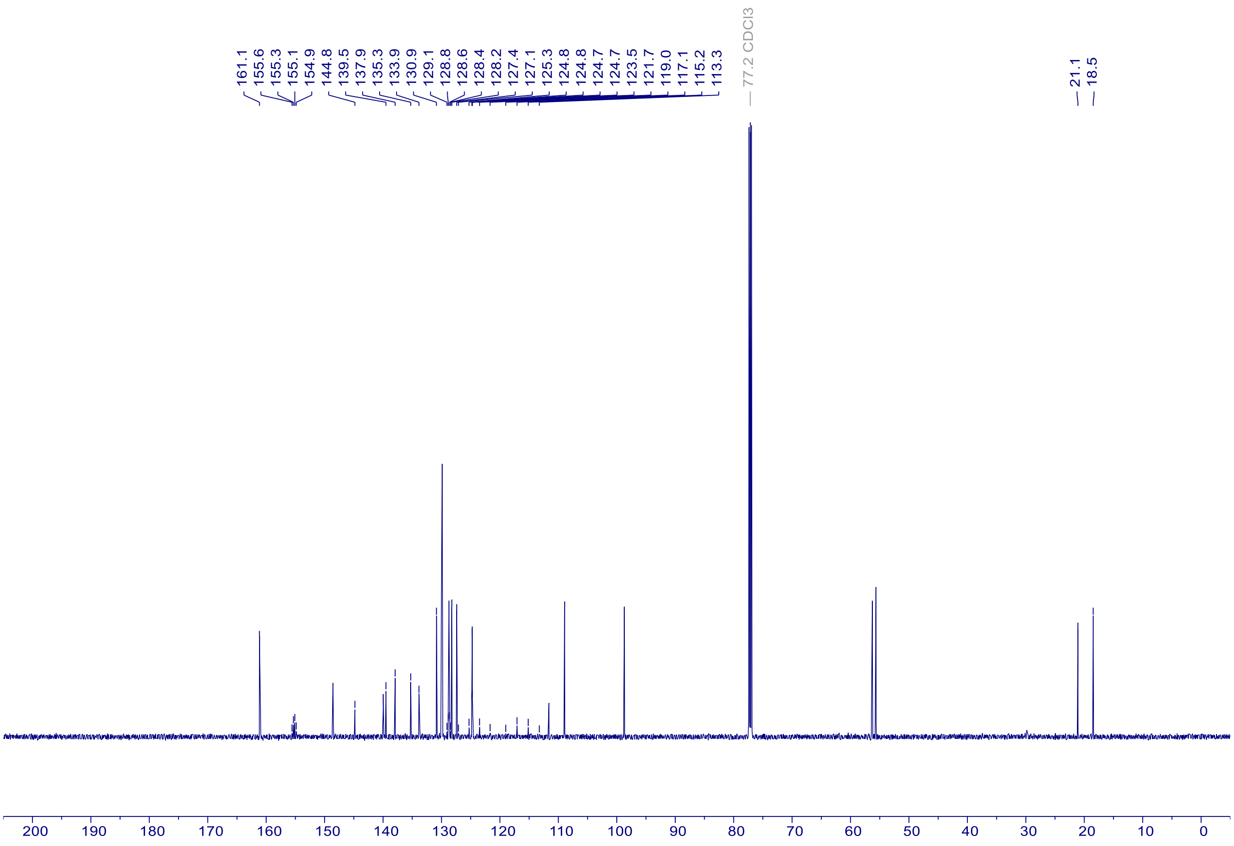

6e – ^{19}F NMR (565 MHz, $CDCl_3$)

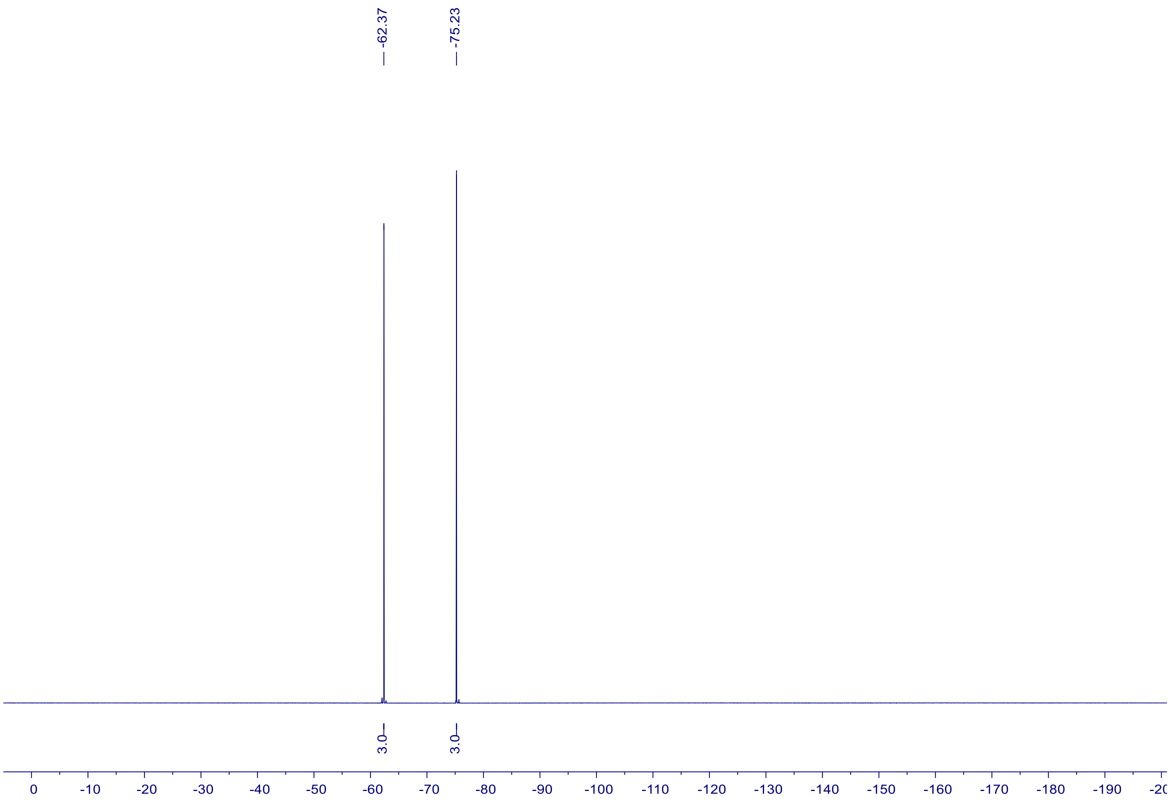

Supplement: SC-016-D5SC05329C-s002 [file SC-016-D5SC05329C-s002.pdf]
